# Supplementary material for: Comparative Venomics of the Cryptic Cone Snail Species Virroconus ebraeus and Virroconus judaeus
Source: Mar Drugs. 2022 Feb 17;20(2):149. doi: 10.3390/md20020149 (PMC8875821; doi:10.3390/md20020149)
Supplement: Supplementary file 1 [file marinedrugs-20-00149-s001.zip › Supplementary Figures.pdf]

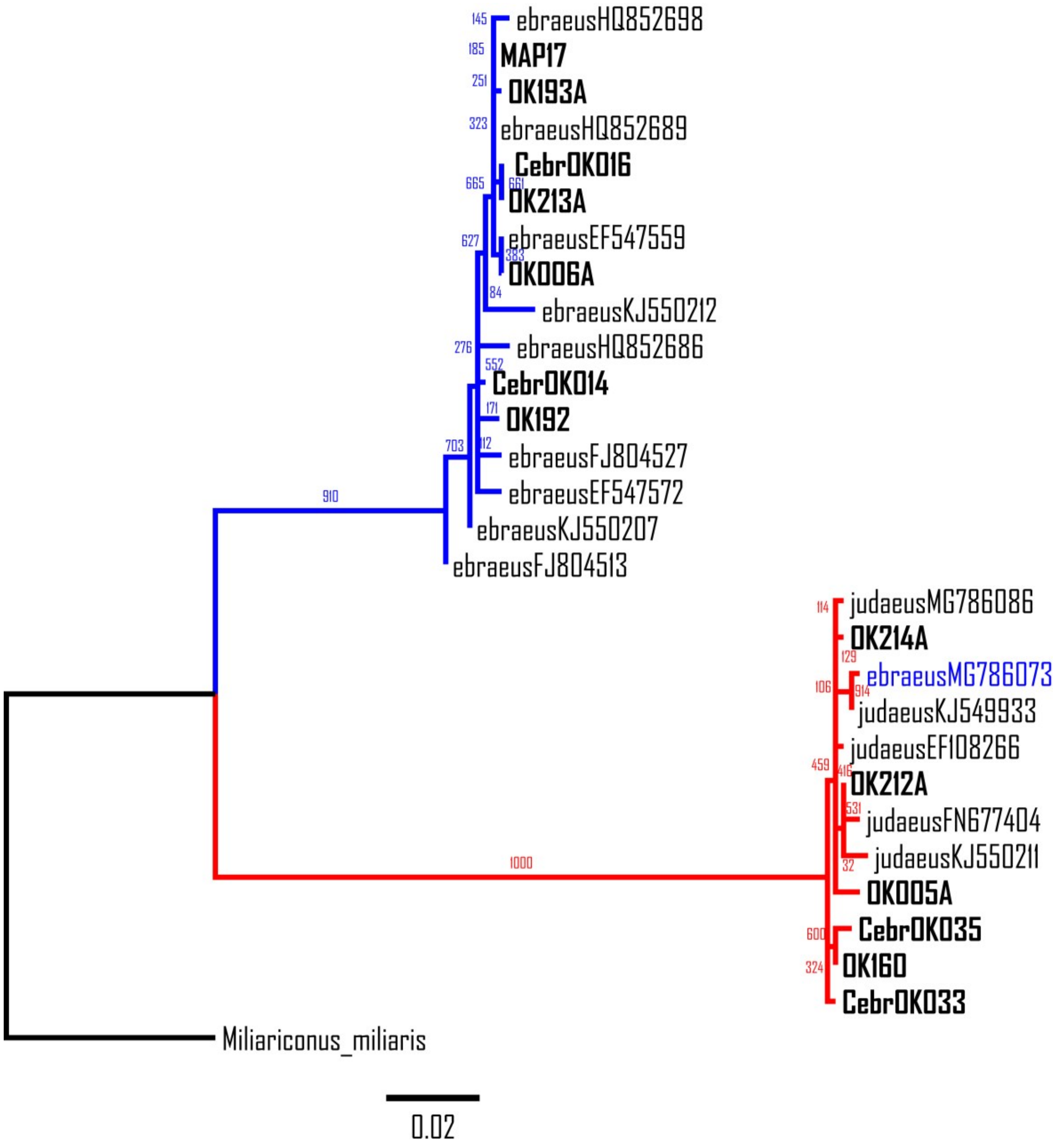

Supplementary Figure S1. Reconstructed maximum likelihood phylogeny based on barcoding *cox1* fragment of *V. ebraeus* and *V. judaeus* samples analyzed in this work plus entries retrieved from GenBank. Node values represent bootstraps (1000 replicates). Blue clade is the *V. ebraeus* whereas the red clade is *V. judaeus*. The samples analyzed in this work are marked in bold. In blue is highlighted a misidentified sample of *Virroconus judaeus*.

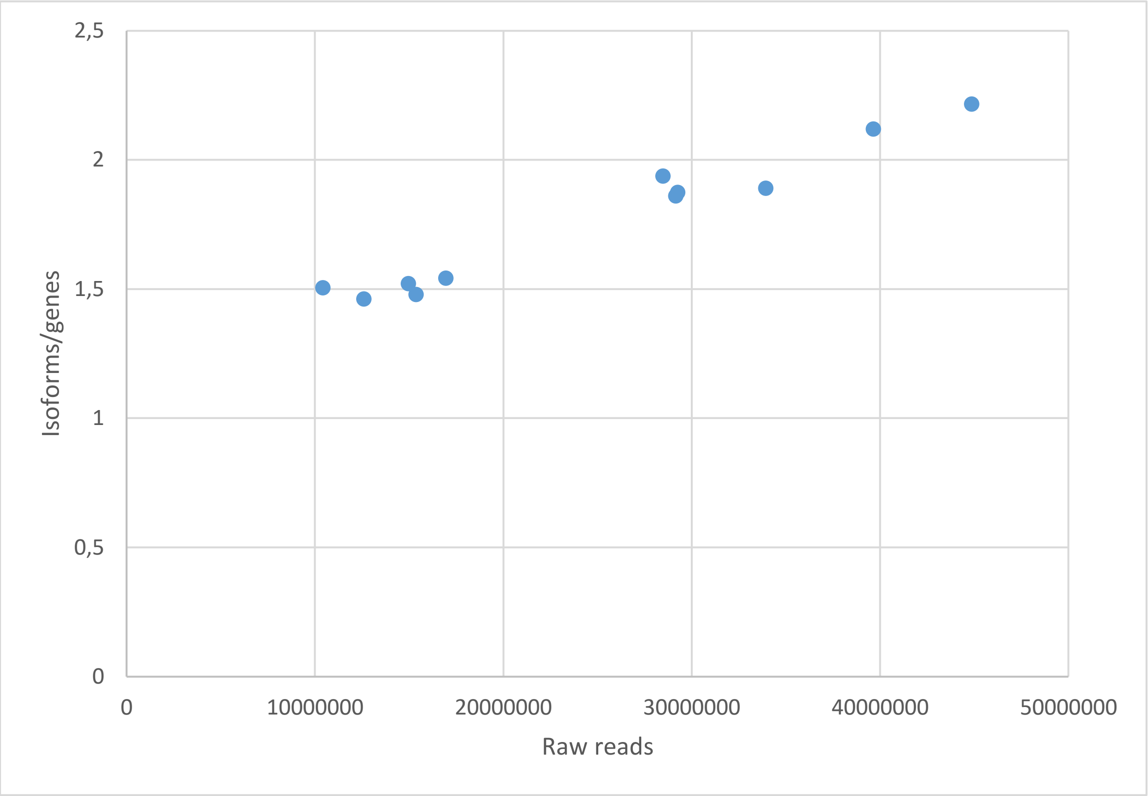

Supplementary Figure S2. In the X axis is represented the number of raw reads of the 11 samples analyzed in this work. The Y axis represents the ratio between the number of isoforms and genes assembled by Trinity for each sample. The *p*-value for the regression was $9x10^{-8}$.

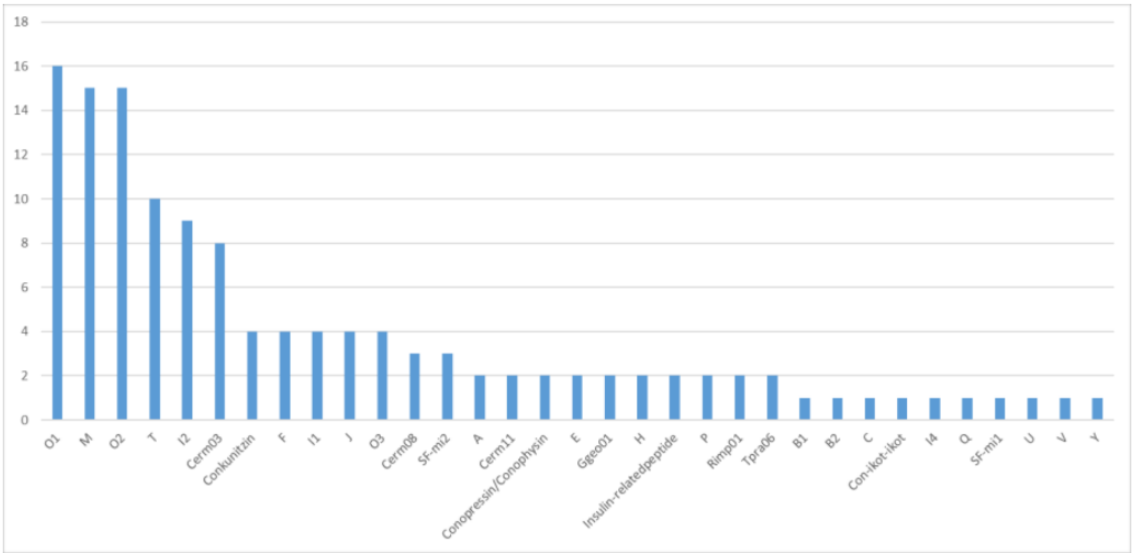

Supplementary Figure S3. Conotoxin precursor superfamilies and hormones common to both *V. ebraeus* and *V. judaeus*. Bars represent the number of common peptides.

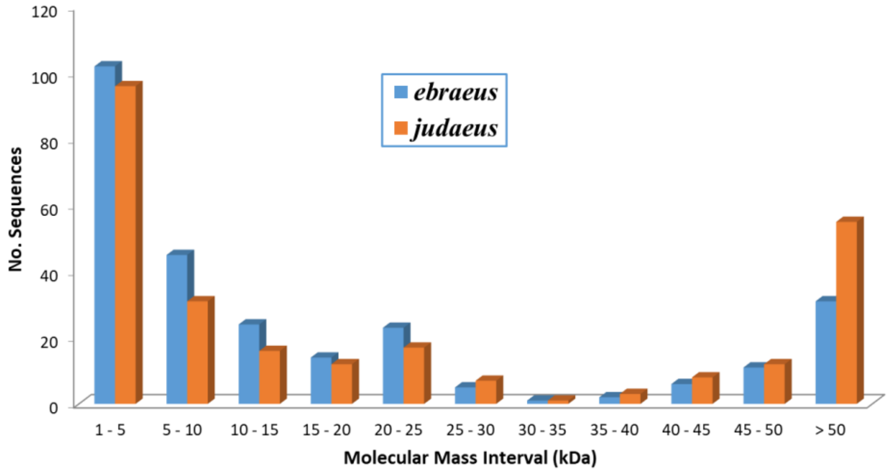

Supplementary Figure S4. Molecular mass distribution of the peptidic components of the venom duct extracts of *V. ebraeus* (blue) and *V. judaeus* (red).

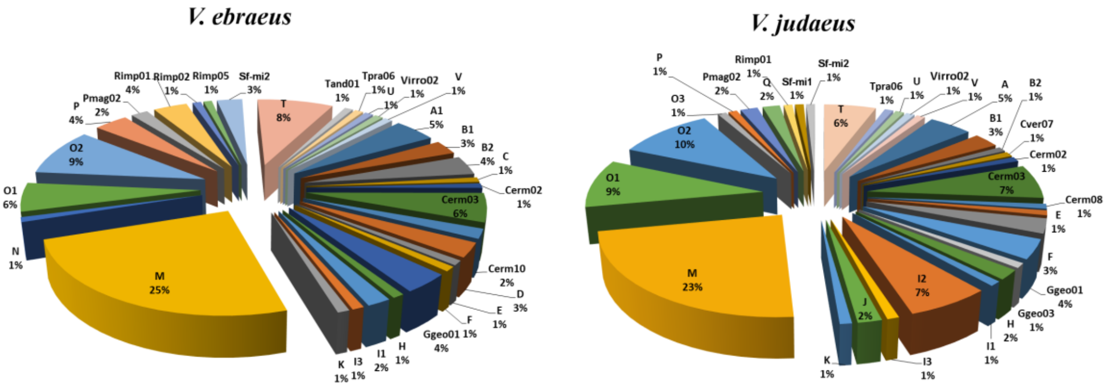

Supplementary Figure S5. Distribution in superfamilies of conotoxins identified by proteomic analysis in the venom duct extracts of *V. ebraeus* and *V. judaeus*.

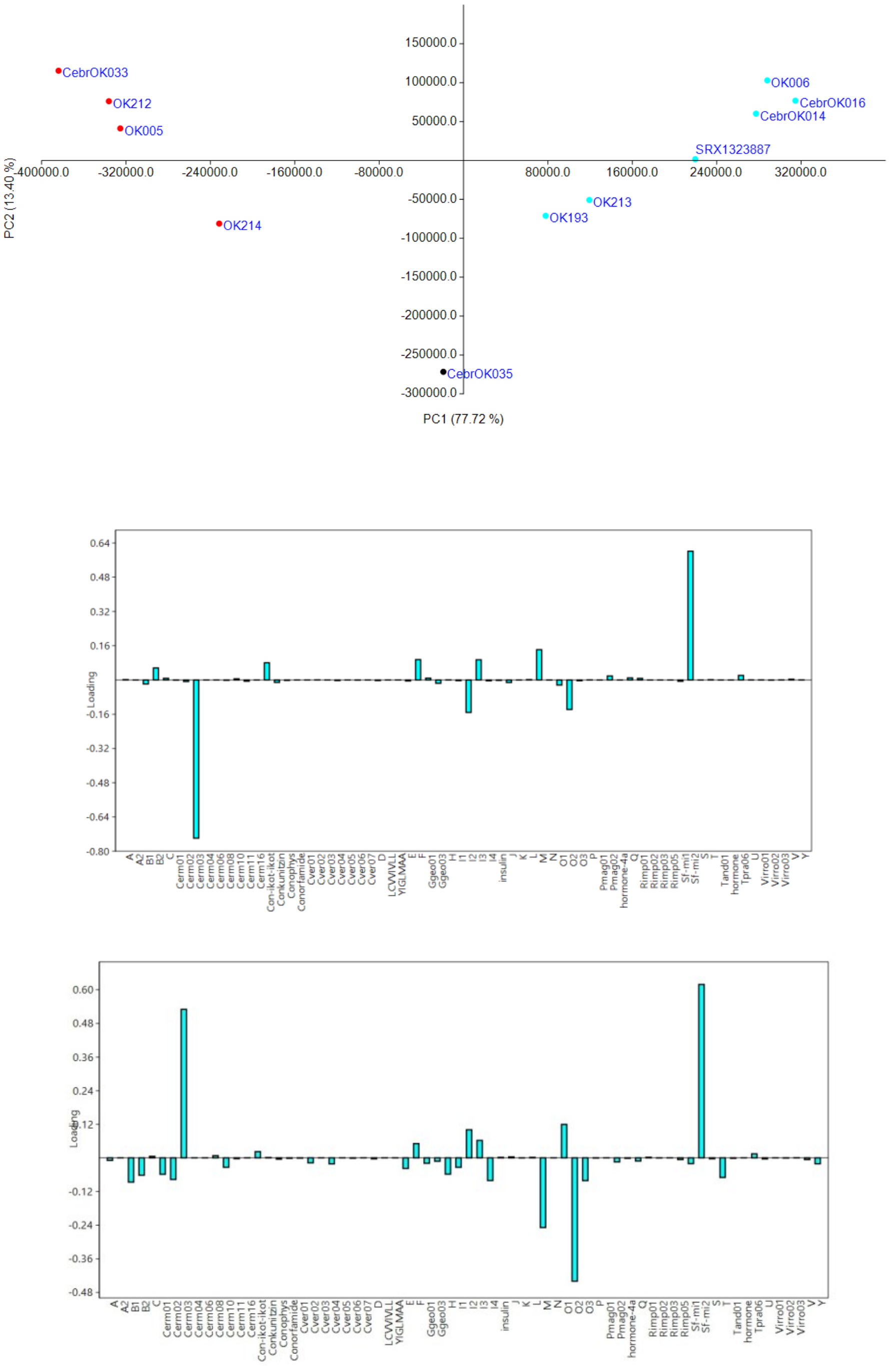

Supplementary Figure S6. PCA comparing venom composition of *V. ebraeus* (blue) and *V. judaeus* (red). Top: 2D PCA scatter plot. Venom composition was defined as the relative expression levels (TPMs) of the different superfamilies. Percentages of the variance for each of the axis (PC1 and PC2) are indicated. Bottom: Loading plots of the first two principal components for the PCA of the venom composition, defined as superfamily conotoxin abundance, among individuals of *V. ebraeus* and *V. judaeus*.

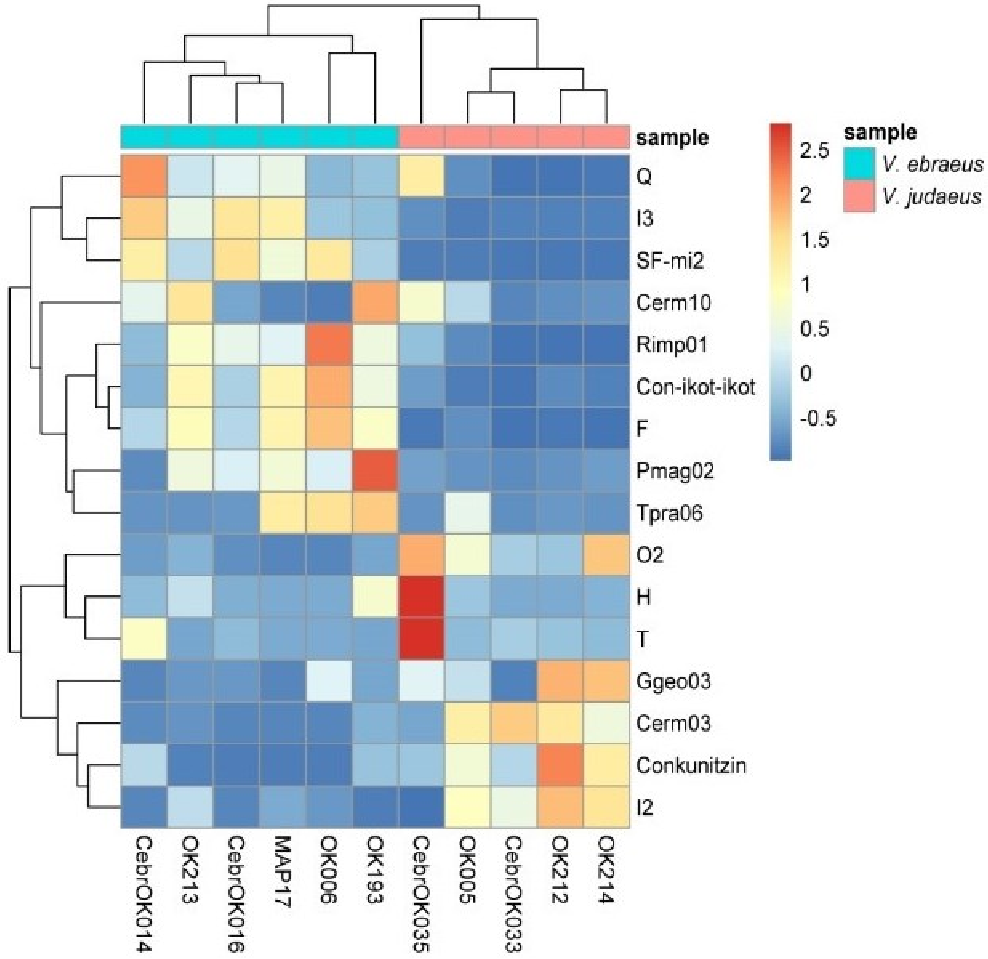

Supplementary Figure S7. Cluster analysis of differentially expressed superfamilies of conotoxin precursors and hormones between the eleven samples of *V. ebraeus* and *V. judaeus* species. Color gradient from dark blue to dark red, represents the Z-score normalized values of the TPMs.

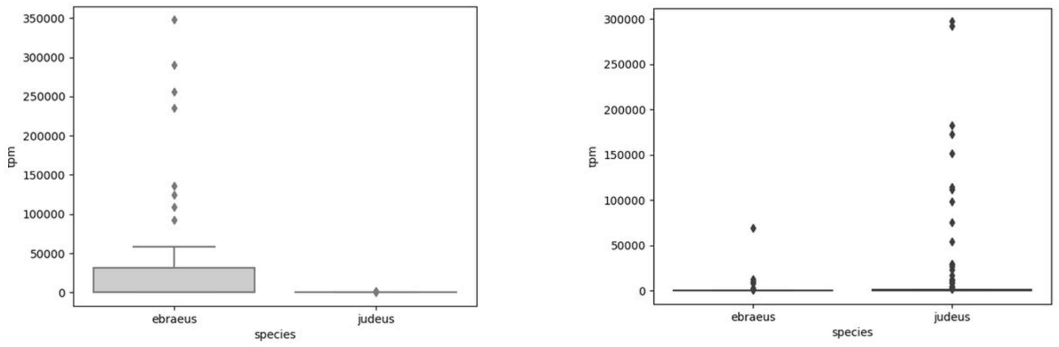

Sf-mi2 **Cerm03**

Supplementary Figure S8. Boxplot with the distribution of the TPM values of all transcripts belonging to superfamilies Sf-mi2 and Cerm03.

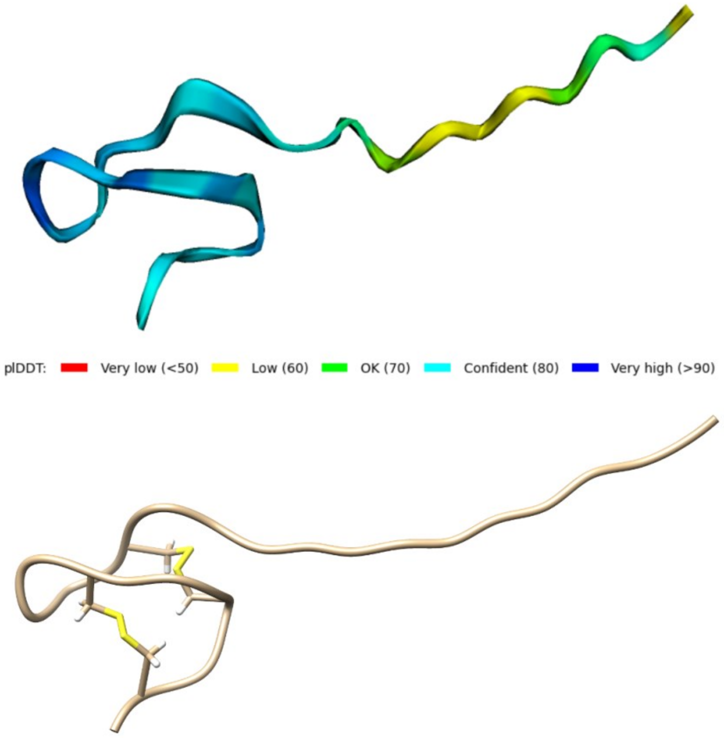

Supplementary Figure S9. Molecular modeling of sequence OK005_J_237 (Cerm03): A) AlphaFold2 result. B) Schematic representation showing the cysteine disulfide bridges.

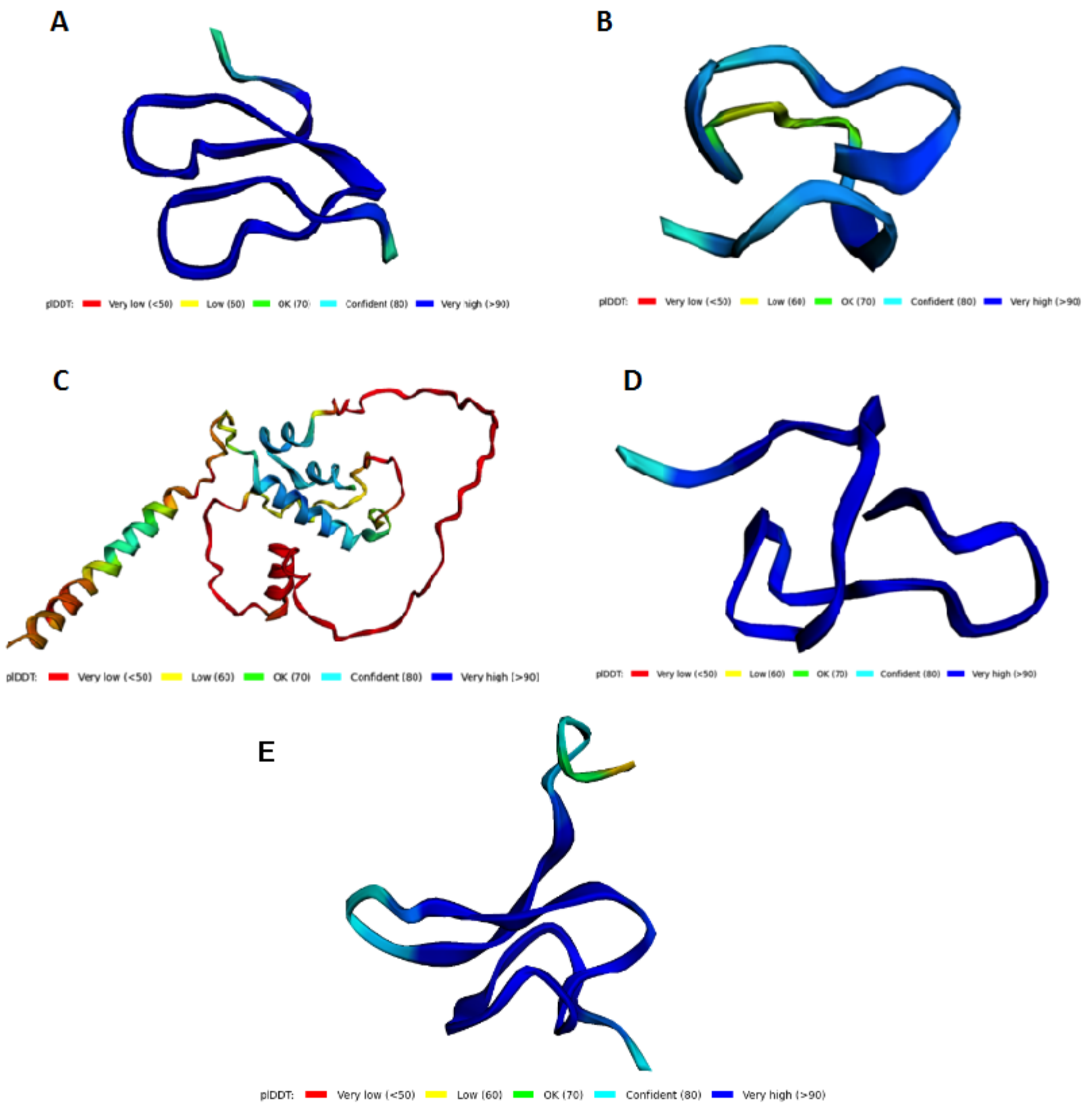

Supplementary Figure S10. Molecular modeling using AlphaFold2 of relevant venom proteins: (A) OK006_E_244 (Sf-mi2); (B) OK193_E_124 (O1); (C) OK213_E_290 (insulin); (D) Eb6.9 (O1); and (E) CebrOK033_J_232 (Virro03).
