# Supplementary material for: Comparative Venomics of the Cryptic Cone Snail Species Virroconus ebraeus and Virroconus judaeus
Source: Mar Drugs. 2022 Feb 17;20(2):149. doi: 10.3390/md20020149 (PMC8875821; doi:10.3390/md20020149)
Supplement: Supplementary file 1 [file marinedrugs-20-00149-s001.zip › Supplementary File S5.pdf]

Summary

1. Notes

2. Result Statistics

Figure S1. False discovery rate (FDR) curve. X axis is the number of peptides being kept. Y axis is the corresponding FDR.

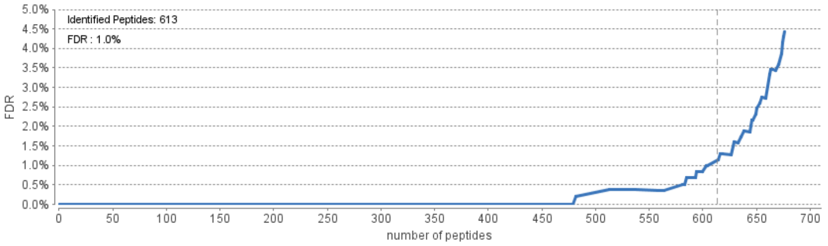

Figure S2. PSM score distribution. **(a)** Distribution of PEAKS peptide score; **(b)** Scatterplot of PEAKS peptide score versus precursor mass error.

(a) **(b)**

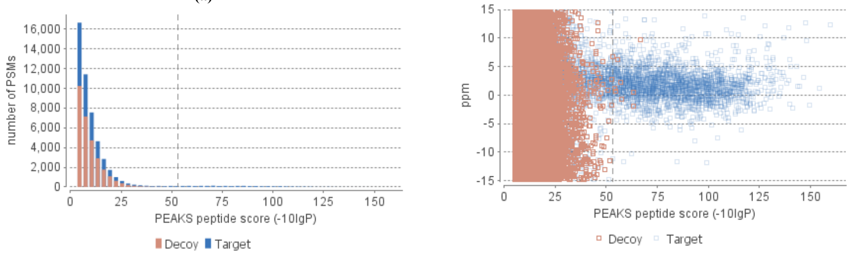

Table S1. Statistics of data.

	#Scans		#Features	Identified			#Peptides	#Sequences	#Proteins*		
	MS1	MS/MS		#PSMs	#Scans	#Features**			Groups	All	Top
Total	0	134759	0	1973	1973	0	613	528	144	594	419
ebraeus	0	52068	0	754	754	0	342	308	106	389	300
judaeus	0	82691	0	1219	1219	0	458	390	101	441	287

* proteins with significant peptides are used in counts.
** features are identified by DB search only.

Figure S3. Sample overlap for Proteins and Peptides (up to 8 samples). **(a)** All Proteins; **(b)** Top Proteins; **(c)** Peptides.

(a) **(b)** **(c)**

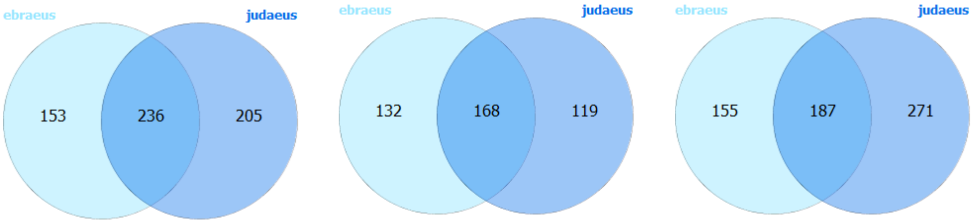

Figure S4. Distribution of peptide feature detection. **(a)** Feature m/z distribution; **(b)** Feature RT distribution.

(a)
No available data for figure

(b)
No available data for figure

Figure S5. Distribution of identified peptide features. **(a)** Feature abundance distribution; **(b)** *De novo* sequencing validation.

(a)
No available data for figure

(b)

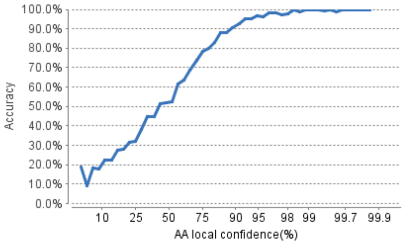

Table S2. Result filtration parameters.

Peptide -10lgP	≥53.3
PTM ion intensity	≥5%
Protein Group FDR	1.0%
Proteins unique peptides	≥1
De novo score(%)	≥80%

Table S3. Statistics of filtered result.

Table S4. PTM profile.

Name	ΔMass	Position	#PSM	-10lgP	Abundance	AScore
Oxidation	15.99	M	204	135.06		1000.00
Carbamidomethyl	57.02	C	137	139.43		1000.00
Pyro-glu from Q	-17.03	N-term	55	112.62		1000.00
Amidation	-.98	C-term	12	105.16		1000.00

FDR (Peptide-Spectrum Matches)	0.4%
FDR (Peptide Sequences)	1.0%
FDR (Protein Group)	1.4%
De Novo Only Spectra	5791

Pyro-glu from E	-18.01	N-term	11	95.20	1000.00

3. Experiment Control

Figure S6. Precursor mass error of peptide-spectrum matches (PSM) in filtered result. **(a)** Distribution of precursor mass error in ppm; **(b)** Scatterplot of precursor m/z versus precursor mass error in ppm.

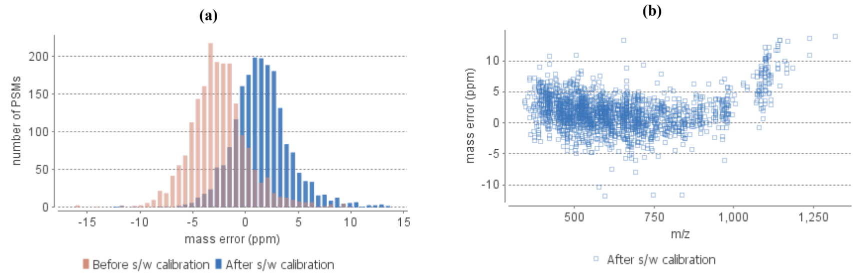

Table S5. Number of identified peptides in each sample by the number of missed cleavages.

Missed Cleavages	0	1	2	3	4+
ebraeus	311	30	1	0	0
judaeus	379	69	7	3	0

4. Other Information

Table S6. Search parameters.

PEAKS Version: PEAKS Studio 10.6 build 20201221
Search Engine Name: PEAKS
Parent Mass Error Tolerance: 15.0 ppm
Fragment Mass Error Tolerance: 0.5 Da
Precursor Mass Search Type: monoisotopic
Enzyme: Trypsin
Max Missed Cleavages: 3
Digest Mode: Specific
Fixed Modifications:
  Carbamidomethylation: 57.02
Variable Modifications:
  Amidation: -0.98
  Oxidation (M): 15.99
  Proline oxidation to pyroglutamic acid: 13.98
  Pyro-glu from E: -18.01
  Pyro-glu from Q: -17.03
Max Variable PTM Per Peptide: 3
Database: CTX-Jul21-final
Taxon: All
Searched Entry: 20085
FDR Estimation: Enabled
Merge Options: no merge
Precursor Options: corrected
Charge Options: no correction
Filter Charge: 2 - 8
Process: true
Associate chimera: yes

Table S7. Instrument parameters.

Fractions: 20210611-08_2021021-OK192A_815_5.3.236.mgf, 20210611-09_2021021-OK160A_816_5.3.236.mgf
Ion Source: ESI(nano-spray)
Fragmentation Mode: CID, CAD(y and b ions)
MS Scan Mode: FT-ICR/Orbitrap
MS/MS Scan Mode: Linear Ion Trap

Protein List

Protein Accession Contains:
Protein Description Contains:
Protein Sample Area >=
Protein PTM Contains:

Protein Group	Protein ID	Accession	-10lgP	Coverage (%)	Coverage (%) ebraeus	Coverage (%) judaeus	Area ebraeus	Area judaeus	#Peptides	#Unique	#Spec ebraeus	#Spec judaeus	PTM	Avg. Mass	Description
**10**	179579	OK193_E_272_PDI	464.87	63	60	50	0		32	3	67	112	Y	56649	OK193_E_272_PDI
**10**	179580	SRX1323887_E_247_PDI	464.87	63	60	50	0		32	3	67	112	Y	56649	SRX1323887_E_247_PDI
**10**	179581	OK006_E_295_PDI	464.87	63	60	50	0		32	3	67	112	Y	56649	OK006_E_295_PDI
**10**	179582	OK213_E_326_PDI	464.87	63	60	50	0		32	3	67	112	Y	56649	OK213_E_326_PDI
**10**	179583	CebrOK016_E_159_PDI	464.87	63	60	50	0		32	3	67	112	Y	56649	CebrOK016_E_159_PDI
**10**	179584	CebrOK014_E_145_PDI	464.87	63	60	50	0		32	3	67	112	Y	56649	CebrOK014_E_145_PDI
**38**	179572	V_1278_121_Protein	420.52	57	55	35	0		28	1	58	71	Y	56331	Disulfide Isomerase (PDI-A2)
**38**	179573	V_1302_228_Protein	420.52	57	55	35	0		28	1	58	71	Y	56331	Disulfide Isomerase (PDI-A2)
**38**	179574	V_1258_220_Protein	420.52	57	55	35	0		28	1	58	71	Y	56331	Disulfide Isomerase (PDI-A2)
**38**	179575	V_CG13_261_Protein	420.52	57	55	35	0		28	1	58	71	Y	56331	Disulfide Isomerase (PDI-A2)
**73**	179696	P_145_Actin	412.38	42	36	39	0	0	15	15	49	53	Y	54744	P_145_Actin
**96**	179739	OK212_J_286_Conoporin	405.70	54	5	54		0	12	2	4	62	Y	27240	OK212_J_286_Conoporin
**94**	179736	CebrOK033_J_240_Conoporin	400.36	52	5	52		0	12	2	4	64	Y	27319	CebrOK033_J_240_Conoporin
**98**	179734	OK213_E_299_Conoporin	367.15	54	54	12	0	0	13	11	55	8	Y	27163	OK213_E_299_Conoporin
**98**	179735	OK193_E_246_Conoporin	367.15	54	54	12	0	0	13	11	55	8	Y	27163	OK193_E_246_Conoporin
**103**	179699	CebrOK035_J_368_PDI	335.41	32	10	32	0	0	16	5	11	33	Y	58117	CebrOK035_J_368_PDI
**103**	179700	OK212_J_325_PDI	335.41	32	10	32	0	0	16	5	11	33	Y	58117	OK212_J_325_PDI
**107**	179809	CebrOK035_J_344_Conoporin	333.95	36	0	36		0	7	1	0	34	Y	26968	CebrOK035_J_344_Conoporin
**132**	179708	P_033D_033_Arginine	331.28	23	1	23	0	0	14	14	2	17	N	79345	kinase
**112**	179834	CebrOK035_J_343_Prohormone-4a	305.11	22	11	22		0	7	4	7	22	N	38910	CebrOK035_J_343_Prohormone-4a
**112**	179835	OK214_J_305_Prohormone-4a	305.11	20	10	20		0	7	4	7	22	N	42816	OK214_J_305_Prohormone-4a
**112**	179836	OK212_J_281_Prohormone-4a	305.11	20	10	20		0	7	4	7	22	N	42829	OK212_J_281_Prohormone-4a
**112**	179837	OK212_J_284_Prohormone-4a	305.11	19	10	19		0	7	4	7	22	N	44814	OK212_J_284_Prohormone-4a
**112**	179838	OK212_J_282_Prohormone-4a	305.11	18	9	18		0	7	4	7	22	N	46869	OK212_J_282_Prohormone-4a
**112**	179839	CebrOK035_J_340_Prohormone-4a	305.11	18	9	18		0	7	4	7	22	N	48222	CebrOK035_J_340_Prohormone-4a
**112**	179840	OK212_J_283_Prohormone-4a	305.11	17	9	17		0	7	4	7	22	N	48873	OK212_J_283_Prohormone-4a
**112**	179841	CebrOK033_J_239_Prohormone-4a	305.11	17	9	17		0	7	4	7	22	N	48914	CebrOK033_J_239_Prohormone-4a
**112**	179842	OK214_J_306_Prohormone-4a	305.11	17	8	17		0	7	4	7	22	N	50917	OK214_J_306_Prohormone-4a
**112**	179843	OK005_J_296_Prohormone-4a	305.11	17	8	17		0	7	4	7	22	N	50930	OK005_J_296_Prohormone-4a
**112**	179844	CebrOK035_J_339_Prohormone-4a	305.11	17	8	17		0	7	4	7	22	N	50912	CebrOK035_J_339_Prohormone-4a

total 419 proteins

Protein Group	Protein ID	Accession	-10lgP	Coverage (%)	Coverage (%) ebraeus	Coverage (%) judaeus	Area ebraeus	Area judaeus	#Peptides	#Unique	#Spec ebraeus	#Spec judaeus	PTM	Avg. Mass	Description
**112**	179845	CebrOK035_J_341_Prohormone-4a	305.11	16	8	16		0	7	4	7	22	N	54694	CebrOK035_J_341_Prohormone-4a
**112**	179846	CebrOK035_J_342_Prohormone-4a	305.11	15	8	15		0	7	4	7	22	N	54945	CebrOK035_J_342_Prohormone-4a
**122**	179811	P_109D_104_Gelsolin-like	302.25	24	11	24	0	0	7	7	4	20	Y	36438	P_109D_104_Gelsolin-like
**110**	179741	gene:107296774	300.43	14	11	14	0	0	9	9	14	16	Y	72155	transcript:XP015681610.1 DBNLINE|14034|
**118**	179758	CebrOK033_J_270_PDI	299.60	25	10	25		0	8	1	8	18	N	47799	CebrOK033_J_270_PDI
**118**	179759	OK214_J_340_PDI	299.60	25	10	25		0	8	1	8	18	N	47799	OK214_J_340_PDI
**118**	179756	OK005_J_322_PDI	299.60	25	10	25		0	8	1	8	18	N	47773	OK005_J_322_PDI
**118**	179757	OK212_J_328_PDI	299.60	25	10	25		0	8	1	8	18	N	47822	OK212_J_328_PDI
**118**	179762	OK214_J_341_PDI	299.60	25	10	25		0	8	1	8	18	N	47773	OK214_J_341_PDI
**123**	179760	OK006_E_298_PDI	297.69	25	12	23	0		8	1	9	14	N	47800	OK006_E_298_PDI
**123**	179761	OK193_E_275_PDI	297.69	25	12	23	0		8	1	9	14	N	47800	OK193_E_275_PDI
**123**	179763	SRX1323887_E_251_PDI	297.69	25	12	23	0		8	1	9	14	N	47814	SRX1323887_E_251_PDI
**108**	245416	CV8_286_Protein	288.64	21	10	19	0		10	1	12	19	N	57800	Disulfide Isomerase
**108**	179714	V_CG13_228_Protein	288.64	21	10	19	0		10	1	12	19	N	57929	Disulfide Isomerase (PDI-A3)
**108**	179715	A_1387_037	288.64	21	11	19	0		10	1	12	19	N	56522	Protein Disulfide Isomerase (PDI-A3)
**108**	179716	A_0025_374_Protein	288.64	22	11	19	0		10	1	12	19	N	56025	Disulfide Isomerase (PDI-A3)
**108**	179720	A_0885_194_Protein	288.64	21	10	19	0		10	1	12	19	N	57808	Disulfide Isomerase (PDI-A3)
**108**	179717	A_0855_188_Protein	288.64	21	10	19	0		10	1	12	19	N	57698	Disulfide Isomerase (PDI-A3)
**108**	179719	A_0048_031_Protein	288.64	21	10	19	0		10	1	12	19	N	57810	Disulfide Isomerase (PDI-A3)
**135**	179812	D_070_Calreticulin-like	284.54	22	2	22	0	0	8	8	1	17	N	47502	D_070_Calreticulin-like
**131**	179747	OK212_J_296_Ferritin	283.95	58	23	58	0	0	7	3	5	15	N	19702	OK212_J_296_Ferritin
**131**	179748	CebrOK033_J_246_Ferritin	283.95	58	23	58	0	0	7	3	5	15	N	19675	CebrOK033_J_246_Ferritin
**131**	179749	OK214_J_315_Ferritin	283.95	58	23	58	0	0	7	3	5	15	N	19675	OK214_J_315_Ferritin
**131**	179750	OK005_J_302_Ferritin	283.95	58	23	58	0	0	7	3	5	15	N	19702	OK005_J_302_Ferritin
**131**	179765	CebrOK035_J_351_Ferritin	283.95	58	23	58	0	0	7	3	5	15	N	19689	CebrOK035_J_351_Ferritin
**131**	179766	SRX1323887_E_233_Ferritin	283.95	58	23	58	0	0	7	3	5	15	N	19689	SRX1323887_E_233_Ferritin
**131**	179767	OK006_E_274_Ferritin	283.95	58	23	58	0	0	7	3	5	15	N	19689	OK006_E_274_Ferritin
**131**	179768	OK193_E_251_Ferritin	283.95	58	23	58	0	0	7	3	5	15	N	19689	OK193_E_251_Ferritin
**131**	179799	OK213_E_305_Ferritin	283.95	58	23	58	0	0	7	3	5	15	N	19675	OK213_E_305_Ferritin
**131**	179800	CebrOK014_E_138_Ferritin	283.95	58	23	58	0	0	7	3	5	15	N	19675	CebrOK014_E_138_Ferritin
**136**	179832	D_041_Thioredoxin	282.56	33	23	33		0	6	1	5	13	Y	22326	peroxidase 2
**102**	179896	D_085_FK506-binding	279.94	19	19	19	0	0	8	8	15	32	Y	30452	protein 2-like
**130**	179764	P_002D_003_Thioredoxin-like	277.08	14	1	14	0	0	7	7	2	18	Y	67946	P_002D_003_Thioredoxin-like
**117**	179732	K_0010_152_Protein	275.19	17	8	17		0	8	1	9	17	N	57456	Disulfide Isomerase (PDI-A3)
**121**	179871	CebrOK035_J_332_Tpra06	270.66	48	0	48		0	5	5	0	24	Y	15017	CebrOK035_J_332_Tpra06
**121**	179879	OK212_J_274_Tpra06	270.66	48	0	48		0	5	5	0	24	Y	15043	OK212_J_274_Tpra06
**121**	179880	CebrOK033_J_231_Tpra06	270.66	48	0	48		0	5	5	0	24	Y	15043	CebrOK033_J_231_Tpra06
**149**	179786	CebrOK016_E_148_Ferritin	260.29	48	6	48		0	8	6	1	9	Y	19836	CebrOK016_E_148_Ferritin
**149**	179787	OK213_E_304_Ferritin	260.29	48	6	48		0	8	6	1	9	Y	19836	OK213_E_304_Ferritin
**149**	179788	OK006_E_273_Ferritin	260.29	48	6	48		0	8	6	1	9	Y	19836	OK006_E_273_Ferritin
**149**	179789	OK005_J_301_Ferritin	260.29	48	6	48		0	8	6	1	9	Y	19836	OK005_J_301_Ferritin
**149**	179790	OK212_J_294_Ferritin	260.29	48	6	48		0	8	6	1	9	Y	19836	OK212_J_294_Ferritin
**149**	179791	OK193_E_250_Ferritin	260.29	48	6	48		0	8	6	1	9	Y	19836	OK193_E_250_Ferritin
**149**	179792	K_0010_064_Ferritin	260.29	48	6	48		0	8	6	1	9	Y	19836	K_0010_064_Ferritin
**149**	179793	CebrOK014_E_137_Ferritin	260.29	48	6	48		0	8	6	1	9	Y	19836	CebrOK014_E_137_Ferritin
**149**	179794	SRX1323887_E_232_Ferritin	260.29	48	6	48		0	8	6	1	9	Y	19836	SRX1323887_E_232_Ferritin
**149**	179795	OK214_J_314_Ferritin	260.29	48	6	48		0	8	6	1	9	Y	19836	OK214_J_314_Ferritin
**149**	179796	CebrOK035_J_350_Ferritin	260.29	48	6	48		0	8	6	1	9	Y	19836	CebrOK035_J_350_Ferritin
**149**	179797	CebrOK033_J_243_Ferritin	260.29	48	6	48		0	8	6	1	9	Y	19836	CebrOK033_J_243_Ferritin
**149**	179801	OK212_J_295_Ferritin	260.29	48	6	48		0	8	6	1	9	Y	19870	OK212_J_295_Ferritin
**149**	179802	V_1278_120_Ferritin	260.29	48	6	48		0	8	6	1	9	Y	19820	V_1278_120_Ferritin
**149**	179803	V_1302_266_Ferritin	260.29	48	6	48		0	8	6	1	9	Y	19820	V_1302_266_Ferritin
**149**	179804	V_1258_223_Ferritin	260.29	48	6	48		0	8	6	1	9	Y	19820	V_1258_223_Ferritin
**149**	179805	V_CG13_283_Ferritin	260.29	48	6	48		0	8	6	1	9	Y	19820	V_CG13_283_Ferritin
**93**	179972	OK212_J_014_B2	256.30	45	20	45		0	5	1	5	63	Y	14832	OK212_J_014_B2
**93**	179973	OK214_J_010_B2	256.30	45	20	45		0	5	1	5	63	Y	14832	OK214_J_010_B2
**93**	179974	OK005_J_013_B2	256.30	45	20	45		0	5	1	5	63	Y	14832	OK005_J_013_B2
**93**	179975	CebrOK035_J_018_B2	256.30	45	20	45		0	5	1	5	63	Y	14832	CebrOK035_J_018_B2
**93**	179976	CebrOK033_J_011_B2	256.30	45	20	45		0	5	1	5	63	Y	14832	CebrOK033_J_011_B2
**141**	179744	OK213_E_318_PPI	252.53	37	0	37		0	7	5	0	14	N	23008	OK213_E_318_PPI
**141**	179745	SRX1323887_E_240_PPI	252.53	37	0	37		0	7	5	0	14	N	23063	SRX1323887_E_240_PPI
**141**	179746	CebrOK035_J_257_PPI	252.53	41	0	41		0	7	5	0	14	N	20894	CebrOK035_J_257_PPI
**141**	179751	CebrOK033_J_256_PPI	252.53	37	0	37		0	7	5	0	14	N	23077	CebrOK033_J_256_PPI
**141**	179752	OK005_J_311_PPI	252.53	37	0	37		0	7	5	0	14	N	23077	OK005_J_311_PPI
**141**	179753	OK006_E_286_PPI	252.53	37	0	37		0	7	5	0	14	N	23077	OK006_E_286_PPI
**141**	179754	OK193_E_264_PPI	252.53	37	0	37		0	7	5	0	14	N	23077	OK193_E_264_PPI
**141**	179755	OK214_J_328_PPI	252.53	37	0	37		0	7	5	0	14	N	23077	OK214_J_328_PPI
**141**	179774	OK212_J_315_PPI	252.53	37	0	37		0	7	5	0	14	N	23063	OK212_J_315_PPI
**104**	179740	gene:107297086	248.77	30	30	28	0	0	8	8	23	22	Y	27632	transcript:XP015681930.1 DBNLINE|14330|
**133**	179848	OK193_E_277_Thioredoxin-like	244.15	26	22	26		0	6	1	5	14	Y	22827	protein
**114**	179864	gene:107292429	238.49	32	32	32	0	0	6	6	14	14	Y	16854	transcript:XP015677000.1 DBNLINE|9931|
**116**	179806	P_096_Elongation	236.77	11	9	11	0	0	5	5	13	14	N	50496	factor
**124**	123	Pvau2_100	233.02	21	15	21	0	0	4	3	4	19	Y	24456	Pvau2_100
**101**	179977	OK213_E_018_B2	229.35	47	45	22	0		5	1	7	42	Y	14399	OK213_E_018_B2
**109**	179932	OK006_E_036_F	224.91	69	69	30	0	0	4	4	30	1	Y	7573	OK006_E_036_F
**109**	179933	OK213_E_040_F	224.91	69	69	30	0	0	4	4	30	1	Y	7573	OK213_E_040_F
**109**	179934	SRX1323887_E_036_F	224.91	69	69	30	0	0	4	4	30	1	Y	7573	SRX1323887_E_036_F
**109**	179935	CebrOK014_E_011_F	224.91	69	69	30	0	0	4	4	30	1	Y	7573	CebrOK014_E_011_F
**109**	179936	OK214_J_037_F	224.91	69	69	30	0	0	4	4	30	1	Y	7573	OK214_J_037_F
**109**	179937	OK005_J_033_F	224.91	69	69	30	0	0	4	4	30	1	Y	7573	OK005_J_033_F
**109**	179938	OK193_E_033_F	224.91	69	69	30	0	0	4	4	30	1	Y	7633	OK193_E_033_F
**109**	179939	CebrOK016_E_013_F	224.91	69	69	30	0	0	4	4	30	1	Y	7633	CebrOK016_E_013_F
**109**	179940	OK213_E_041_F	224.91	69	69	30	0	0	4	4	30	1	Y	7633	OK213_E_041_F
**109**	179941	CebrOK014_E_010_F	224.91	69	69	30	0	0	4	4	30	1	Y	7633	CebrOK014_E_010_F
**109**	179942	SRX1323887_E_035_F	224.91	69	69	30	0	0	4	4	30	1	Y	7633	SRX1323887_E_035_F
**145**	179830	gene:107300483	224.02	28	11	28	0	0	5	5	2	9	N	22752	transcript:XP015685299.1 DBNLINE|17459|
**138**	179849	OK214_J_343_CAP	223.90	26	6	26	0	0	4	4	2	15	N	21613	OK214_J_343_CAP
**138**	179856	CebrOK033_J_271_CAP	223.90	26	6	26	0	0	4	4	2	15	N	21585	protein
**171**	179877	K_0010_139_Ferritin	208.54	30	8	30		0	4	1	1	4	N	15089	K_0010_139_Ferritin
**106**	181102	OK212_J_216_Cerm03	205.38	67	64	67		0	3	1	4	31	N	3510	OK212_J_216_Cerm03
**125**	179863	gene:107288075	203.91	34	16	34	0	0	4	4	6	16	Y	13996	transcript:XP015672174.1 DBNLINE|5735|
**125**	179865	gene:107298957	203.91	34	16	34	0	0	4	4	6	16	Y	13952	transcript:XP015683800.1 DBNLINE|16060|
**125**	179870	gene:107297087	203.91	34	16	34	0	0	4	4	6	16	Y	14050	transcript:XP015681932.1 DBNLINE|14331|
**126**	179978	CebrOK016_E_140_Tpra	203.65	30	30	0	0		4	4	22	0	Y	14047	06
**126**	179956	SRX1323887_E_218_Tpra06	203.65	29	29	0	0		4	4	22	0	Y	14906	SRX1323887_E_218_Tpra06
**126**	179957	CebrOK014_E_131_Tpra06	203.65	29	29	0	0		4	4	22	0	Y	14906	CebrOK014_E_131_Tpra06

total 419 proteins

Protein Group	Protein ID	Accession	-10lgP	Coverage (%)	Coverage (%) ebraeus	Coverage (%) judaeus	Area ebraeus	Area judaeus	#Peptides	#Unique	#Spec ebraeus	#Spec judaeus	PTM	Avg. Mass	Description
**126**	179958	OK213_E_279_Tpra06	203.65	29	29	0	0		4	4	22	0	Y	14906	OK213_E_279_Tpra06
**126**	179955	OK006_E_261_Tpra06	203.65	29	29	0	0		4	4	22	0	Y	14875	OK006_E_261_Tpra06
**126**	179964	OK006_E_262_Tpra06	203.65	29	29	0	0		4	4	22	0	Y	14873	OK006_E_262_Tpra06
**144**	179903	V_1278_106_Prohormone-4a	202.79	14	14	2	0		4	1	8	4	Y	42200	V_1278_106_Prohormone-4a
**144**	179904	V_CG13_205_Prohormone-4a	202.79	14	14	2	0		4	1	8	4	Y	42199	V_CG13_205_Prohormone-4a
**165**	179828	P_094D_091_Angiotensin-converting	202.24	6	4	4	0	0	4	4	3	3	N	81827	enzyme-like
**140**	179769	P_102D_097_Nucleobinding-2-like	199.47	13	11	7	0	0	6	6	5	9	N	48472	P_102D_097_Nucleobinding-2-lik e
**137**	179981	OK212_J_009_B1	197.15	51	11	51		0	3	1	2	16	N	8340	OK212_J_009_B1
**137**	179982	OK005_J_007_B1	197.15	51	11	51		0	3	1	2	16	N	8340	OK005_J_007_B1
**137**	180002	OK212_J_010_B1	197.15	41	9	41		0	3	1	2	16	N	10340	OK212_J_010_B1
**137**	179999	OK212_J_008_B1	197.15	53	11	53		0	3	1	2	16	N	7953	OK212_J_008_B1
**137**	180000	OK005_J_006_B1	197.15	53	11	53		0	3	1	2	16	N	7953	OK005_J_006_B1
**120**	182188	OK212_J_220_Cerm03	196.20	53	41	53		0	2	1	2	23	N	3523	OK212_J_220_Cerm03
**134**	179983	OK214_J_005_B1	195.80	51	11	51		0	3	1	2	17	N	8354	OK214_J_005_B1
**134**	180001	OK214_J_004_B1	195.80	53	11	53		0	3	1	2	17	N	7967	OK214_J_004_B1
**128**	179965	V_1278_048_E-1	190.86	44	44	30	0	0	4	4	15	6	Y	8480	V_1278_048_E-1
**128**	179966	V_CG13_066_E-1	190.86	44	44	30	0	0	4	4	15	6	Y	8480	V_CG13_066_E-1
**139**	180539	SRX1323887_E_199_Pmag02	189.56	32	14	18		0	3	2	7	9	Y	8081	SRX1323887_E_199_Pmag02
**139**	180540	SRX1323887_E_200_Pmag02	189.56	32	14	18		0	3	2	7	9	Y	8081	SRX1323887_E_200_Pmag02
**127**	180454	OK212_J_265_Pmag02	187.94	32	18	32		0	3	1	9	13	Y	8090	OK212_J_265_Pmag02
**127**	180915	CebrOK035_J_309_Pmag02	187.94	32	18	32		0	3	1	9	13	Y	8123	CebrOK035_J_309_Pmag02
**127**	181080	CebrOK035_J_308_Pmag02	187.94	32	18	32		0	3	1	9	13	Y	8091	CebrOK035_J_308_Pmag02
**127**	181081	CebrOK033_J_219_Pmag02	187.94	32	18	32		0	3	1	9	13	Y	8091	CebrOK033_J_219_Pmag02
**127**	181082	OK214_J_271_Pmag02	187.94	32	18	32		0	3	1	9	13	Y	8091	OK214_J_271_Pmag02
**127**	181083	OK005_J_275_Pmag02	187.94	32	18	32		0	3	1	9	13	Y	8116	OK005_J_275_Pmag02
**127**	181084	OK212_J_264_Pmag02	187.94	32	18	32		0	3	1	9	13	Y	8091	OK212_J_264_Pmag02
**127**	181085	OK214_J_272_Pmag02	187.94	32	18	32		0	3	1	9	13	Y	8130	OK214_J_272_Pmag02
**105**	179930	gene:107299479	185.85	23	23	23	0	0	4	4	22	13	N	20702	transcript:XP015684320.1 DBNLI NE|16549|
**176**	179994	SRX1323887_E_190_Ggeo01	184.26	61	61	0	0		3	1	5	0	N	7219	SRX1323887_E_190_Ggeo01
**176**	179995	CebrOK014_E_119_Ggeo01	184.26	61	61	0	0		3	1	5	0	N	7193	CebrOK014_E_119_Ggeo01
**176**	179996	OK005_J_264_Ggeo01	184.26	61	61	0	0		3	1	5	0	N	7161	OK005_J_264_Ggeo01
**176**	179997	OK213_E_251_Ggeo01	184.26	61	61	0	0		3	1	5	0	N	7161	OK213_E_251_Ggeo01
**159**	180503	P_022_Coagulation	184.02	15	15	8	0	0	3	3	6	2	Y	23382	factor
**162**	179929	D_059_Immunoglobulin-like	183.37	9	2	9	0	0	3	3	3	4	Y	41965	domain
**169**	179915	OK214_J_329_PPI	178.01	17	0	17		0	3	1	0	5	N	23008	OK214_J_329_PPI
**169**	179916	OK212_J_316_PPI	178.01	17	0	17		0	3	1	0	5	N	23008	OK212_J_316_PPI
**201**	179998	SRX1323887_E_191_Ggeo01	174.73	54	54	0	0		3	1	3	0	N	8172	SRX1323887_E_191_Ggeo01
**212**	180302	OK193_E_234_Tand01	172.52	27	27	0	0		2	2	2	0	N	12931	OK193_E_234_Tand01
**212**	180303	OK213_E_278_Tand01	172.52	27	27	0	0		2	2	2	0	N	12931	OK213_E_278_Tand01
**212**	180304	OK006_E_260_Tand01	172.52	27	27	0	0		2	2	2	0	N	12931	OK006_E_260_Tand01
**212**	180305	SRX1323887_E_217_Tand01	172.52	27	27	0	0		2	2	2	0	N	12931	SRX1323887_E_217_Tand01
**143**	179831	P_079D_079_Metallopeptidase	172.45	6	2	6	0	0	4	4	1	11	N	64734	P_079D_079_Metallopeptidase
**166**	179833	gene:107297064	168.85	5	0	5		0	4	4	0	6	N	91127	transcript:XP015681908.1 DBNLI NE|14308|
**167**	179914	gene:107289560	168.59	15	7	15	0	0	3	3	2	4	N	29134	transcript:XP015673826.1 DBNLI NE|7176|
**154**	179921	OK193_E_248_Conodipine	165.82	23	23	0	0		4	2	9	0	Y	20340	OK193_E_248_Conodipine
**154**	179922	SRX1323887_E_230_Conodipine	165.82	23	23	0	0		4	2	9	0	Y	20344	SRX1323887_E_230_Conodipine
**154**	179924	OK213_E_301_Conodipine	165.82	23	23	0	0		4	2	9	0	Y	20477	OK213_E_301_Conodipine
**154**	179925	OK006_E_272_Conodipine	165.82	23	23	0	0		4	2	9	0	Y	20477	OK006_E_272_Conodipine
**148**	180083	V_1302_329_T-3-WF	160.40	38	38	38	0	0	2	1	9	2	Y	5742	V_1302_329_T-3-WF
**148**	181103	V_1302_150_T-3-WF	160.40	42	42	42	0	0	2	1	9	2	Y	5242	V_1302_150_T-3-WF
**148**	181104	V_1258_142_T-3-WF	160.40	42	42	42	0	0	2	1	9	2	Y	5242	V_1258_142_T-3-WF
**148**	181105	V_1302_151_T-3-WF	160.40	40	40	40	0	0	2	1	9	2	Y	5727	V_1302_151_T-3-WF
**148**	181106	V_1258_074_T-3-WF	160.40	38	38	38	0	0	2	1	9	2	Y	5924	V_1258_074_T-3-WF
**168**	179920	gene:107292247	156.53	3	0	3		0	3	3	0	5	N	103037	transcript:XP015676813.1 DBNLI NE|9754|
**184**	180553	OK212_J_239_Cerm08	152.79	29	0	29		0	2	2	0	4	Y	10110	OK212_J_239_Cerm08
**184**	180554	CebrOK033_J_197_Cerm08	152.79	29	0	29		0	2	2	0	4	Y	10110	CebrOK033_J_197_Cerm08
**177**	179989	gene:107285995	152.00	25	8	25	0	0	3	3	2	3	N	20697	transcript:XP015669766.1 DBNLI NE|3721|
**150**	181079	V_CG13_085_B2	150.40	40	40	0	0		2	2	10	0	Y	15163	V_CG13_085_B2
**155**	182213	OK214_J_237_Cerm03	149.89	55	0	55		0	4	4	0	9	N	3355	OK214_J_237_Cerm03
**190**	180098	OK212_J_300_Thioredoxin	148.61	8	0	8		0	2	2	0	3	N	43581	domain-containing protein 5
**190**	180099	OK005_J_306_Thioredoxin	148.61	8	0	8		0	2	2	0	3	N	43581	domain-containing protein 5
**181**	179986	SRX1323887_E_229_Conodipine	145.93	20	20	0	0		3	1	4	0	Y	20361	SRX1323887_E_229_Conodipine
**181**	179987	OK193_E_247_Conodipine	145.93	20	20	0	0		3	1	4	0	Y	20357	OK193_E_247_Conodipine
**160**	181116	gene:107298953	143.17	37	7	37	0	0	2	2	2	6	N	10526	transcript:XP015683796.1 DBNLI NE|16056|
**142**	181101	OK212_J_224_Cerm03	136.93	70	23	70		0	2	1	2	12	N	3285	OK212_J_224_Cerm03
**111**	181087	OK213_E_102_M	136.34	58	58	58	0	0	2	2	18	12	N	3551	OK213_E_102_M
**191**	180813	V_CG13_016_Conkunitzin-12	134.58	16	16	0	0		2	2	3	0	N	12555	V_CG13_016_Conkunitzin-12
**191**	181052	V_1302_039_Conkunitzin-12	134.58	16	16	0	0		2	2	3	0	N	12597	V_1302_039_Conkunitzin-12
**147**	180171	OK213_E_009_B1	133.77	32	32	11	0		2	1	6	5	N	8454	OK213_E_009_B1
**147**	180145	OK213_E_008_B1	133.77	33	33	11	0		2	1	6	5	N	8008	OK213_E_008_B1
**147**	180188	SRX1323887_E_010_B1	133.77	33	33	11	0		2	1	6	5	N	8011	SRX1323887_E_010_B1
**147**	180189	OK193_E_008_B1	133.77	33	33	11	0		2	1	6	5	N	8011	OK193_E_008_B1
**147**	180190	OK213_E_010_B1	133.77	33	33	11	0		2	1	6	5	N	8011	OK213_E_010_B1
**147**	180191	CebrOK014_E_003_B1	133.77	33	33	11	0		2	1	6	5	N	8011	CebrOK014_E_003_B1
**151**	181112	OK193_E_026_Conkunitzin	128.50	30	30	0	0		2	2	10	0	Y	6959	OK193_E_026_Conkunitzin
**151**	181113	OK006_E_028_Conkunitzin	128.50	30	30	0	0		2	2	10	0	Y	6959	OK006_E_028_Conkunitzin
**151**	181114	SRX1323887_E_026_Conkunitzin	128.50	30	30	0	0		2	2	10	0	Y	6959	SRX1323887_E_026_Conkunitzin
**151**	181115	OK213_E_030_Conkunitzin	128.50	30	30	0	0		2	2	10	0	Y	6959	OK213_E_030_Conkunitzin
**161**	179913	gene:107288180	128.31	4	4	4	0	0	2	2	2	5	N	54031	transcript:XP015672252.1 DBNLI NE|5833|
**173**	181530	OK212_J_025_Conkunitzin	127.65	32	0	32		0	1	1	0	5	Y	7081	OK212_J_025_Conkunitzin
**173**	181531	OK212_J_026_Conkunitzin	127.65	32	0	32		0	1	1	0	5	Y	7065	OK212_J_026_Conkunitzin
**215**	180086	gene:107288688	123.35	15	0	15		0	2	2	0	2	Y	21782	transcript:XP015672813.1 DBNLI NE|6321|
**183**	218	Pvau2_22	112.72	5	0	5		0	2	2	0	4	N	52333	Pvau2_22
**179**	180112	gene:107294617	111.81	2	2	2	0	0	1	1	2	2	N	91720	transcript:XP015679347.1 DBNLI NE|12030|
**180**	180096	P_027D_029_Neuroendocrine	111.72	2	0	2		0	1	1	0	4	N	74870	convertase
**249**	182205	OK193_E_214_Ggeo01	108.40	32	32	0	0		1	1	1	0	N	4628	OK193_E_214_Ggeo01
**244**	182045	V_CG13_188_D	105.56	35	35	0	0		1	1	1	0	Y	5103	V_CG13_188_D
**178**	182050	OK193_E_115_M	105.36	34	34	0	0		2	2	5	0	Y	3277	OK193_E_115_M
**185**	180408	V_CG13_073_Rimp	104.60	11	11	11	0	0	1	1	3	1	N	12577	01
**164**	182107	OK213_E_049_H	103.98	29	29	0	0		1	1	7	0	N	6417	OK213_E_049_H
**188**	180013	A_0025_010_60S	102.93	22	0	22		0	2	2	0	3	N	12416	ribosomal protein L10
**188**	180014	A_1387_362	102.93	22	0	22		0	2	2	0	3	N	12416	60S ribosomal protein L10
**188**	180015	A_0885_182_60S	102.93	22	0	22		0	2	2	0	3	N	12416	ribosomal protein L10
**188**	180016	A_0055_259_60S	102.93	22	0	22		0	2	2	0	3	N	12416	ribosomal protein L10
**188**	180017	A_0520_023_60S	102.93	22	0	22		0	2	2	0	3	N	12416	ribosomal protein L10
**188**	180018	A_0048_318_60S	102.93	22	0	22		0	2	2	0	3	N	12416	ribosomal protein L10

total 419 proteins

Protein Group	Protein ID	Accession	-10lgP	Coverage (%)	Coverage (%) ebraeus	Coverage (%) judaeus	Area ebraeus	Area judaeus	#Peptides	#Unique	#Spec ebraeus	#Spec judaeus	PTM	Avg. Mass	Description
**188**	180019	A_0031_033_60S	102.93	22	0	22		0	2	2	0	3	N	12416	ribosomal protein L10
**188**	180020	A_0855_173_60S	102.93	22	0	22		0	2	2	0	3	N	12416	ribosomal protein L10
**188**	180021	K_0010_065_60S	102.93	22	0	22		0	2	2	0	3	N	12442	ribosomal protein L10
**188**	180022	A_0239_021_60S	102.93	22	0	22		0	2	2	0	3	N	12416	ribosomal protein L10
**188**	180023	A_0875_147_60S	102.93	22	0	22		0	2	2	0	3	N	12416	ribosomal protein L10
**188**	180024	A_0039_214_60S	102.93	22	0	22		0	2	2	0	3	N	12416	ribosomal protein L10
**186**	181095	OK212_J_022_Con-ikot-ikot	102.53	22	0	22		0	2	2	0	4	Y	10743	OK212_J_022_Con-ikot-ikot
**186**	181096	OK214_J_017_Con-ikot-ikot	102.53	22	0	22		0	2	2	0	4	Y	10743	OK214_J_017_Con-ikot-ikot
**186**	181097	CebrOK035_J_031_Con-ikot-ikot	102.53	22	0	22		0	2	2	0	4	Y	10743	CebrOK035_J_031_Con-ikot-ikot
**186**	181098	OK214_J_018_Con-ikot-ikot	102.53	21	0	21		0	2	2	0	4	Y	10848	OK214_J_018_Con-ikot-ikot
**199**	182215	V_1258_008_M-5-WF	102.49	27	27	0	0		2	2	3	0	N	5707	V_1258_008_M-5-WF
**199**	182216	V_1302_055_M-5-WF	102.49	19	19	0	0		2	2	3	0	N	7934	V_1302_055_M-5-WF
**262**	182206	SRX1323887_E_029_D	101.45	30	30	0	0		1	1	1	0	Y	4789	SRX1323887_E_029_D
**262**	182207	OK213_E_034_D	101.45	30	30	0	0		1	1	1	0	Y	4723	OK213_E_034_D
**156**	181120	V_CG13_175_T-3-WF	99.73	33	33	15	0		2	1	8	1	N	5152	V_CG13_175_T-3-WF
**202**	181107	OK006_E_057_I1	98.47	44	44	0	0		2	2	3	0	Y	4930	OK006_E_057_I1
**202**	181108	OK213_E_051_I1	98.47	44	44	0	0		2	2	3	0	Y	4930	OK213_E_051_I1
**202**	181109	CebrOK014_E_015_I1	98.47	44	44	0	0		2	2	3	0	Y	4930	CebrOK014_E_015_I1
**202**	181110	OK193_E_044_I1	98.47	44	44	0	0		2	2	3	0	Y	4929	OK193_E_044_I1
**202**	181111	SRX1323887_E_041_I1	98.47	44	44	0	0		2	2	3	0	Y	4929	SRX1323887_E_041_I1
**216**	182208	OK006_E_265_Conopressin	98.21	10	0	10		0	1	1	0	2	N	12783	/ Conophysin
**216**	182209	CebrOK035_J_333_Conopressin/Conophysin	98.21	9	0	9		0	1	1	0	2	N	13777	CebrOK035_J_333_Conopressin/ Conophysin
**220**	181117	CebrOK033_J_111_O1	95.69	51	0	51		0	2	2	0	2	N	4194	CebrOK033_J_111_O1
**174**	181516	OK213_E_029_Conkunitzin	94.00	15	15	0	0		2	2	5	0	N	7109	OK213_E_029_Conkunitzin
**256**	181962	OK213_E_035_D	93.29	30	30	0	0		1	1	1	0	Y	4845	OK213_E_035_D
**256**	181963	OK193_E_028_D	93.29	30	30	0	0		1	1	1	0	Y	4845	OK193_E_028_D
**256**	182212	CebrOK014_E_009_D	93.29	76	76	0	0		1	1	1	0	Y	1983	CebrOK014_E_009_D
**194**	180942	V_1258_145_T-3	89.65	46	46	0	0		2	2	3	0	N	4768	V_1258_145_T-3
**194**	180943	V_1302_153_T-3	89.65	46	46	0	0		2	2	3	0	N	4768	V_1302_153_T-3
**194**	181123	V_1302_154_T-3	89.65	46	46	0	0		2	2	3	0	N	4785	V_1302_154_T-3
**194**	181124	V_1258_144_T-3	89.65	46	46	0	0		2	2	3	0	N	4785	V_1258_144_T-3
**194**	181125	V_CG13_170_T-3	89.65	46	46	0	0		2	2	3	0	N	4785	V_CG13_170_T-3
**194**	181126	V_CG13_174_T-3	89.65	46	46	0	0		2	2	3	0	N	4786	V_CG13_174_T-3
**245**	180605	gene:107288629	86.87	3	0	3		0	1	1	0	1	N	45804	transcript:XP015672753.1 DBNLI NE|6264|
**205**	182210	CebrOK035_J_218_Q	86.20	36	0	36		0	1	1	0	3	Y	3782	CebrOK035_J_218_Q
**205**	182211	OK214_J_192_Q	86.20	36	0	36		0	1	1	0	3	Y	3782	OK214_J_192_Q
**209**	180185	gene:107290492	84.88	3	0	3		0	1	1	0	2	N	58417	transcript:XP015674860.1 DBNLI NE|8072|
**213**	180838	CebrOK016_E_005_B2	84.86	46	46	0	0		2	2	2	0	N	14210	CebrOK016_E_005_B2
**213**	181128	CebrOK014_E_004_B2	84.86	46	46	0	0		2	2	2	0	N	14180	CebrOK014_E_004_B2
**213**	181129	CebrOK016_E_004_B2	84.86	46	46	0	0		2	2	2	0	N	14180	CebrOK016_E_004_B2
**187**	182218	V_1278_082_T-3	84.64	24	24	24	0	0	1	1	3	1	N	4119	V_1278_082_T-3
**187**	1391	AXL95619_conotoxin_precursor_superfamily_T_Conus_ermineus	84.64	24	24	24	0	0	1	1	3	1	N	4119	AXL95619_conotoxin_precursor_s uperfamily_T_Conus_ermineus
**187**	182219	V_1278_081_T-3	84.64	24	24	24	0	0	1	1	3	1	N	4119	V_1278_081_T-3
**187**	1394	MATURECV10_022_Conotoxin_precursor_T_T_3	84.64	24	24	24	0	0	1	1	3	1	N	4119	MATURECV10_022_Conotoxin_pr ecursor_T_T_3
**187**	182220	V_1278_083_T-3	84.64	24	24	24	0	0	1	1	3	1	N	4118	V_1278_083_T-3
**172**	267	Pvau2_102	84.51	2	2	2	0	0	1	1	1	4	N	67029	Pvau2_102
**172**	324	Pvau2_101	84.51	2	2	2	0	0	1	1	1	4	N	65840	Pvau2_101
**196**	181836	OK213_E_130_M	83.34	24	24	0	0		1	1	3	0	N	6266	OK213_E_130_M
**196**	181837	OK193_E_113_M	83.34	24	24	0	0		1	1	3	0	N	6266	OK193_E_113_M
**196**	181838	SRX1323887_E_086_M	83.34	24	24	0	0		1	1	3	0	N	6266	SRX1323887_E_086_M
**196**	181839	OK006_E_111_M	83.34	24	24	0	0		1	1	3	0	N	6266	OK006_E_111_M
**175**	182271	SRX1323887_E_022_Con-ikot-ikot	82.56	10	10	0	0		1	1	5	0	Y	9256	SRX1323887_E_022_Con-ikot-iko t
**175**	182272	OK193_E_023_Con-ikot-ikot	82.56	10	10	0	0		1	1	5	0	Y	9242	OK193_E_023_Con-ikot-ikot
**175**	182273	OK006_E_022_Con-ikot-ikot	82.56	10	10	0	0		1	1	5	0	Y	9256	OK006_E_022_Con-ikot-ikot
**175**	182274	OK213_E_025_Con-ikot-ikot	82.56	10	10	0	0		1	1	5	0	Y	9256	OK213_E_025_Con-ikot-ikot
**175**	182275	OK006_E_021_Con-ikot-ikot	82.56	10	10	0	0		1	1	5	0	Y	9361	OK006_E_021_Con-ikot-ikot
**253**	1058	JAB84712_Conodipine_Vc2_prepropeptide_Conus_victoriae	81.19	6	6	0	0		1	1	1	0	N	19004	JAB84712_Conodipine_Vc2_prepr opeptide_Conus_victoriae
**222**	1379	Pneo_78	79.22	30	0	30		0	1	1	0	2	N	6326	Pneo_78
**263**	182214	OK213_E_290_Insulin-related	79.03	12	12	0	0		1	1	1	0	N	20850	peptide
**189**	181476	gene:107291672	77.88	10	10	10	0	0	1	1	1	2	N	10058	transcript:XP015676180.1 DBNLI NE|9211|
**203**	182221	K_0010_325_Conkunitzin-12	77.50	10	10	0	0		1	1	3	0	N	12568	K_0010_325_Conkunitzin-12
**261**	181980	gene:107293627	77.33	2	0	2		0	1	1	0	1	N	74837	transcript:XP015678272.1 DBNLI NE|11078|
**239**	216	Pvau2_11	76.52	3	0	3		0	1	1	0	1	N	65152	Pvau2_11
**239**	212	Pvau2_17	76.52	2	0	2		0	1	1	0	1	N	106303	Pvau2_17
**192**	181635	K_0010_003_Con-ikot-ikot-4	76.39	11	11	0	0		1	1	3	0	N	8773	K_0010_003_Con-ikot-ikot-4
**204**	182235	V_1278_027_I1-1	75.34	22	22	0	0		1	1	3	0	Y	4395	V_1278_027_I1-1
**204**	182231	V_1302_121_I1-1	75.34	22	22	0	0		1	1	3	0	Y	4477	V_1302_121_I1-1
**204**	182232	V_1258_040_I1-1	75.34	22	22	0	0		1	1	3	0	Y	4477	V_1258_040_I1-1
**204**	182233	A_0855_013_I1-1	75.34	22	22	0	0		1	1	3	0	Y	4362	A_0855_013_I1-1
**204**	182234	A_0885_033_I1-1	75.34	22	22	0	0		1	1	3	0	Y	4362	A_0885_033_I1-1
**204**	182236	A_0031_321_I1-1	75.34	22	22	0	0		1	1	3	0	Y	4392	A_0031_321_I1-1
**204**	182237	A_0055_009_I1-1	75.34	22	22	0	0		1	1	3	0	Y	4362	A_0055_009_I1-1
**204**	1230	AXL95521_conotoxin_precursor_superfamily_I1_Conus_ermineus	75.34	22	22	0	0		1	1	3	0	Y	4362	AXL95521_conotoxin_precursor_s uperfamily_I1_Conus_ermineus
**204**	182238	V_CG13_054_I1-1	75.34	22	22	0	0		1	1	3	0	Y	4436	V_CG13_054_I1-1
**204**	182239	A_0855_014_I1-1	75.34	21	21	0	0		1	1	3	0	Y	4655	A_0855_014_I1-1
**204**	182240	A_0520_205_I1-1	75.34	19	19	0	0		1	1	3	0	Y	5146	A_0520_205_I1-1
**230**	245780	CV8_288_Protein	74.95	1	0	1		0	1	1	0	1	N	86303	Disulfide Isomerase
**230**	180526	OK213_E_328_PDI	74.95	1	0	1		0	1	1	0	1	N	86454	OK213_E_328_PDI
**230**	180527	OK006_E_297_PDI	74.95	1	0	1		0	1	1	0	1	N	86468	OK006_E_297_PDI
**230**	180476	K_0010_438_Protein	74.95	1	0	1		0	1	1	0	1	N	77577	Disulfide Isomerase (PDI-A5)
**230**	180566	V_1258_178_Protein	74.95	2	0	2		0	1	1	0	1	N	71576	Disulfide Isomerase (PDI-A5)
**230**	180567	V_1302_231_Protein	74.95	2	0	2		0	1	1	0	1	N	71576	Disulfide Isomerase (PDI-A5)
**230**	180821	CebrOK033_J_269_PDI	74.95	1	0	1		0	1	1	0	1	N	82079	CebrOK033_J_269_PDI
**230**	180822	SRX1323887_E_250_PDI	74.95	1	0	1		0	1	1	0	1	N	86454	SRX1323887_E_250_PDI
**230**	180823	OK214_J_339_PDI	74.95	1	0	1		0	1	1	0	1	N	86501	OK214_J_339_PDI
**230**	180824	OK212_J_327_PDI	74.95	1	0	1		0	1	1	0	1	N	86501	OK212_J_327_PDI
**230**	180825	OK193_E_274_PDI	74.95	1	0	1		0	1	1	0	1	N	86466	OK193_E_274_PDI
**230**	180826	OK005_J_321_PDI	74.95	1	0	1		0	1	1	0	1	N	86501	OK005_J_321_PDI
**230**	180918	A_0855_170_Protein	74.95	2	0	2		0	1	1	0	1	N	57402	Disulfide Isomerase (PDI-A5)
**230**	180919	A_0039_255_Protein	74.95	2	0	2		0	1	1	0	1	N	58543	Disulfide Isomerase (PDI-A5)
**230**	180920	A_0239_037_Protein	74.95	2	0	2		0	1	1	0	1	N	58472	Disulfide Isomerase (PDI-A5)
**230**	180882	V_1278_149_Protein	74.95	2	0	2		0	1	1	0	1	N	71544	Disulfide Isomerase (PDI-A5)
**230**	180883	A_0039_254_Protein	74.95	2	0	2		0	1	1	0	1	N	71664	Disulfide Isomerase (PDI-A5)
**230**	180884	V_CG13_297_Protein	74.95	2	0	2		0	1	1	0	1	N	71510	Disulfide Isomerase (PDI-A5)
**230**	180885	A_0855_171_Protein	74.95	2	0	2		0	1	1	0	1	N	71605	Disulfide Isomerase (PDI-A5)
**230**	180886	A_0055_279_Protein	74.95	2	0	2		0	1	1	0	1	N	71735	Disulfide Isomerase (PDI-A5)

total 419 proteins

Protein Group	Protein ID	Accession	-10lgP	Coverage (%)	Coverage (%) ebraeus	Coverage (%) judaeus	Area ebraeus	Area judaeus	#Peptides	#Unique	#Spec ebraeus	#Spec judaeus	PTM	Avg. Mass	Description
**230**	180917	A_0875_155_Protein	74.95	3	0	3		0	1	1	0	1	N	38496	Disulfide Isomerase (PDI-A5)
**230**	180531	A_0885_186_Protein	74.95	2	0	2		0	1	1	0	1	N	71651	Disulfide Isomerase (PDI-A5)
**210**	182227	OK193_E_118_N	73.45	22	22	0	0		1	1	2	0	N	4239	OK193_E_118_N
**210**	182228	SRX1323887_E_088_N	73.45	22	22	0	0		1	1	2	0	N	4239	SRX1323887_E_088_N
**210**	182229	OK213_E_135_N	73.45	22	22	0	0		1	1	2	0	N	4239	OK213_E_135_N
**210**	182230	OK193_E_117_N	73.45	22	22	0	0		1	1	2	0	N	4239	OK193_E_117_N
**266**	182242	V_CG13_125_A-1	72.97	44	44	0	0		1	1	1	0	Y	1885	V_CG13_125_A-1
**197**	182225	OK006_E_107_M	72.81	43	0	43		0	1	1	0	3	N	2486	OK006_E_107_M
**223**	182217	OK213_E_203_Q	72.77	32	32	0	0		1	1	2	0	Y	4152	OK213_E_203_Q
**217**	182094	OK213_E_192_O2	71.13	17	17	0	0		1	1	2	0	N	5540	OK213_E_192_O2
**163**	182222	OK005_J_272_Ggeo01	70.99	25	0	25		0	1	1	0	7	Y	4059	OK005_J_272_Ggeo01
**163**	182223	CebrOK035_J_299_Ggeo01	70.99	25	0	25		0	1	1	0	7	Y	4059	CebrOK035_J_299_Ggeo01
**163**	182224	OK214_J_263_Ggeo01	70.99	25	0	25		0	1	1	0	7	Y	4059	OK214_J_263_Ggeo01
**231**	180692	gene:107285462	70.43	4	0	4		0	1	1	0	1	N	22635	transcript:XP015669131.1 DBNLINE|3200|
**252**	180948	K_0010_081_Rimp	69.05	11	11	0	0		1	1	1	0	N	12417	01
**258**	181503	OK006_E_029_Conkunitzin	69.01	9	9	0	0		1	1	1	0	N	12057	OK006_E_029_Conkunitzin
**258**	181504	OK193_E_027_Conkunitzin	69.01	9	9	0	0		1	1	1	0	N	12057	OK193_E_027_Conkunitzin
**258**	181505	CebrOK016_E_012_Conkunitzin	69.01	9	9	0	0		1	1	1	0	N	12057	CebrOK016_E_012_Conkunitzin
**258**	181506	SRX1323887_E_028_Conkunitzin	69.01	9	9	0	0		1	1	1	0	N	12057	SRX1323887_E_028_Conkunitzin
**258**	181508	OK213_E_033_Conkunitzin	69.01	6	6	0	0		1	1	1	0	N	17313	OK213_E_033_Conkunitzin
**218**	182243	OK212_J_201_T	66.91	39	0	39		0	1	1	0	2	N	2492	OK212_J_201_T
**208**	180123	D_092_FK506-binding	66.80	4	4	4	0	0	1	1	1	1	N	24293	protein 2-like
**211**	181642	SRX1323887_E_137_P	66.31	28	28	0	0		1	1	2	0	N	3425	SRX1323887_E_137_P
**227**	180397	gene:107290974	65.83	2	0	2		0	1	1	0	1	N	56705	transcript:XP015675408.1 DBNLINE|8539|
**246**	181174	OK214_J_257_Cver07	65.46	6	0	6		0	1	1	0	1	N	20908	OK214_J_257_Cver07
**207**	180100	gene:107293638	65.35	2	0	2		0	1	1	0	2	N	38120	transcript:XP015678284.1 DBNLINE|11089|
**200**	182328	SRX1323887_E_222_Conorfamide	63.04	10	10	0	0		1	1	3	0	N	8297	SRX1323887_E_222_Conorfamide
**200**	182329	OK213_E_288_Conorfamide	63.04	10	10	0	0		1	1	3	0	N	8297	OK213_E_288_Conorfamide
**200**	182330	OK193_E_242_Conorfamide	63.04	10	10	0	0		1	1	3	0	N	8297	OK193_E_242_Conorfamide
**219**	182283	V_1258_138_Q-4	62.05	23	23	0	0		1	1	2	0	N	3751	V_1258_138_Q-4
**219**	182284	V_CG13_043_Q-4	62.05	23	23	0	0		1	1	2	0	N	3724	V_CG13_043_Q-4
**238**	180120	gene:107297725	61.56	2	0	2		0	1	1	0	1	N	40821	transcript:XP015682572.1 DBNLINE|14925|
**259**	182278	OK006_E_160_O2	61.28	26	26	0	0		1	1	1	0	N	3869	OK006_E_160_O2
**259**	182279	OK006_E_159_O2	61.28	26	26	0	0		1	1	1	0	N	3882	OK006_E_159_O2
**236**	182244	OK212_J_105_M	61.19	58	0	58		0	1	1	0	1	N	2580	OK212_J_105_M
**236**	182245	OK213_E_106_M	61.19	58	0	58		0	1	1	0	1	N	2580	OK213_E_106_M
**236**	182246	OK212_J_104_M	61.19	58	0	58		0	1	1	0	1	N	2580	OK212_J_104_M
**236**	182247	OK006_E_092_M	61.19	58	0	58		0	1	1	0	1	N	2580	OK006_E_092_M
**236**	182248	OK212_J_106_M	61.19	58	0	58		0	1	1	0	1	N	2580	OK212_J_106_M
**236**	182249	CebrOK033_J_077_M	61.19	58	0	58		0	1	1	0	1	N	2580	CebrOK033_J_077_M
**236**	182250	OK005_J_090_M	61.19	58	0	58		0	1	1	0	1	N	2580	OK005_J_090_M
**236**	182251	OK212_J_101_M	61.19	58	0	58		0	1	1	0	1	N	2580	OK212_J_101_M
**236**	182252	OK005_J_089_M	61.19	58	0	58		0	1	1	0	1	N	2580	OK005_J_089_M
**236**	182253	OK214_J_092_M	61.19	58	0	58		0	1	1	0	1	N	2580	OK214_J_092_M
**236**	182254	OK212_J_103_M	61.19	58	0	58		0	1	1	0	1	N	2580	OK212_J_103_M
**236**	182255	OK214_J_091_M	61.19	58	0	58		0	1	1	0	1	N	2580	OK214_J_091_M
**236**	182256	OK212_J_115_M	61.19	58	0	58		0	1	1	0	1	N	2596	OK212_J_115_M
**236**	182257	OK212_J_102_M	61.19	58	0	58		0	1	1	0	1	N	2580	OK212_J_102_M
**236**	182258	OK214_J_090_M	61.19	58	0	58		0	1	1	0	1	N	2580	OK214_J_090_M
**236**	182259	OK212_J_109_M	61.19	58	0	58		0	1	1	0	1	N	2580	OK212_J_109_M
**236**	182260	OK212_J_108_M	61.19	58	0	58		0	1	1	0	1	N	2580	OK212_J_108_M
**236**	182261	CebrOK035_J_095_M	61.19	58	0	58		0	1	1	0	1	N	2580	CebrOK035_J_095_M
**236**	182262	OK212_J_087_M	61.19	58	0	58		0	1	1	0	1	N	2580	OK212_J_087_M
**236**	182263	OK214_J_095_M	61.19	58	0	58		0	1	1	0	1	N	2624	OK214_J_095_M
**232**	601	DAC80618_TPA_inf_conodipine_partial_Conus_magus	60.77	5	5	0	0		1	1	1	0	N	18551	DAC80618_TPA_inf_conodipine_partial_Conus_magus
**232**	180309	OK212_J_293_Conodipine	60.77	4	4	0	0		1	1	1	0	N	20620	OK212_J_293_Conodipine
**232**	180367	OK212_J_292_Conodipine	60.77	4	4	0	0		1	1	1	0	N	20809	OK212_J_292_Conodipine
**232**	180682	V_CG13_220_Conodipine-2	60.77	5	5	0	0		1	1	1	0	N	17071	V_CG13_220_Conodipine-2
**232**	180624	V_1258_222_Conodipine-2	60.77	4	4	0	0		1	1	1	0	N	20525	V_1258_222_Conodipine-2
**232**	180639	V_1302_262_Conodipine-2	60.77	4	4	0	0		1	1	1	0	N	20361	V_1302_262_Conodipine-2
**232**	180625	V_1302_263_Conodipine-2	60.77	4	4	0	0		1	1	1	0	N	20531	V_1302_263_Conodipine-2
**232**	246068	CV8_259_Conodipine	60.77	4	4	0	0		1	1	1	0	N	20466	CV8_259_Conodipine
**232**	180756	OK212_J_290_Conodipine	60.77	4	4	0	0		1	1	1	0	N	20627	OK212_J_290_Conodipine
**232**	181511	OK212_J_291_Conodipine	60.77	4	4	0	0		1	1	1	0	N	20816	OK212_J_291_Conodipine
**237**	179993	gene:107302264	60.44	9	0	9		0	1	1	0	1	N	11204	transcript:XP015686849.1 DBNLINE|18974|
**237**	180003	gene:107302095	60.44	6	0	6		0	1	1	0	1	N	17676	transcript:XP015686704.1 DBNLINE|18836|
**214**	181139	A_0239_128_M-3a-WF	60.04	29	29	0	0		1	1	2	0	N	3379	A_0239_128_M-3a-WF
**214**	181140	A_0239_131_M-3a-WF	60.04	28	28	0	0		1	1	2	0	N	3507	A_0239_131_M-3a-WF
**214**	182295	OK214_J_079_M	60.04	40	40	0	0		1	1	2	0	N	2373	OK214_J_079_M
**214**	182296	A_1387_079	60.04	38	38	0	0		1	1	2	0	N	2602	M-3a-WF
**214**	182297	OK006_E_081_M	60.04	32	32	0	0		1	1	2	0	N	2967	OK006_E_081_M
**214**	182298	OK212_J_098_M	60.04	30	30	0	0		1	1	2	0	N	3140	OK212_J_098_M
**214**	182299	K_0010_334_M-3a	60.04	27	27	0	0		1	1	2	0	N	3609	K_0010_334_M-3a
**214**	182300	A_1387_077	60.04	26	26	0	0		1	1	2	0	N	3769	M-3a
**214**	182301	K_0010_335_M-3a-WF	60.04	24	24	0	0		1	1	2	0	N	4026	K_0010_335_M-3a-WF
**214**	1136	AXL95709_conotoxin_precursor_superfamily_M_partial_Conus	60.04	19	19	0	0		1	1	2	0	N	5023	AXL95709_conotoxin_precursor_superfamily_M_partial_Conus
**214**	1066	DAC80580_TPA_inf_conotoxin_superfamily_M_Conus	60.04	18	18	0	0		1	1	2	0	N	5313	DAC80580_TPA_inf_conotoxin_superfamily_M_Conus
**214**	1067	QFQ61048_conotoxin_superfamily_M_Conus_magus	60.04	18	18	0	0		1	1	2	0	N	5204	QFQ61048_conotoxin_superfamily_M_Conus_magus
**221**	181374	gene:107283272	59.84	7	7	7	0	0	1	1	1	1	N	10960	transcript:XP015666633.1 DBNLINE|1071|
**257**	181031	gene:107295193	58.82	4	0	4		0	1	1	0	1	N	26111	transcript:XP015679983.1 DBNLINE|12586|
**255**	182281	OK214_J_161_O2	58.28	32	0	32		0	1	1	0	1	N	3006	OK214_J_161_O2
**255**	182282	OK214_J_169_O2	58.28	25	0	25		0	1	1	0	1	N	3636	OK214_J_169_O2
**242**	182310	OK006_E_230_Ggeo01	58.10	22	22	0	0		1	1	1	0	N	4655	OK006_E_230_Ggeo01
**264**	1383	P08581	58.09	30	30	0	0		1	1	1	0	Y	5013	P08581
**264**	182285	OK193_E_233_Sf-mi2	58.09	30	30	0	0		1	1	1	0	Y	4981	OK193_E_233_Sf-mi2
**250**	182353	gene:107292229	58.05	12	0	12		0	1	1	0	1	N	15667	transcript:XP015676788.1 DBNLINE|9736|
**260**	181408	OK214_J_009_B2	58.02	13	13	0	0		1	1	1	0	N	10396	OK214_J_009_B2
**193**	182286	OK214_J_276_Rimp01	57.67	15	15	0	0		1	1	3	0	Y	7109	OK214_J_276_Rimp01
**193**	182288	OK006_E_239_Rimp01	57.67	9	9	0	0		1	1	3	0	Y	12321	OK006_E_239_Rimp01
**193**	182289	OK213_E_266_Rimp01	57.67	9	9	0	0		1	1	3	0	Y	12369	OK213_E_266_Rimp01
**193**	182290	CebrOK016_E_135_Rimp	57.67	9	9	0	0		1	1	3	0	Y	12321	01
**193**	182291	OK193_E_223_Rimp01	57.67	9	9	0	0		1	1	3	0	Y	12307	OK193_E_223_Rimp01
**193**	182292	SRX1323887_E_204_Rimp01	57.67	9	9	0	0		1	1	3	0	Y	12321	SRX1323887_E_204_Rimp01
**193**	182293	CebrOK014_E_124_Rimp01	57.67	9	9	0	0		1	1	3	0	Y	12321	CebrOK014_E_124_Rimp01
**total 419 proteins**															

Protein Group	Protein ID	Accession	-10lgP	Coverage (%)	Coverage (%) ebraeus	Coverage (%) judaeus	Area ebraeus	Area judaeus	#Peptides	#Unique	#Spec ebraeus	#Spec judaeus	PTM	Avg. Mass	Description
**198**	182339	OK213_E_267_Rimp01	57.66	9	0	9		0	1	1	0	3	N	8878	OK213_E_267_Rimp01
**198**	182346	OK212_J_266_Rimp01	57.66	6	0	6		0	1	1	0	3	N	12580	OK212_J_266_Rimp01
**198**	182347	CebrOK035_J_314_Rimp01	57.66	6	0	6		0	1	1	0	3	N	12580	CebrOK035_J_314_Rimp01
**198**	182348	OK005_J_279_Rimp01	57.66	6	0	6		0	1	1	0	3	N	12580	OK005_J_279_Rimp01
**198**	182345	OK214_J_274_Rimp01	57.66	6	0	6		0	1	1	0	3	N	12370	OK214_J_274_Rimp01
**198**	182349	OK214_J_273_Rimp01	57.66	6	0	6		0	1	1	0	3	N	12580	OK214_J_273_Rimp01
**198**	182350	CebrOK033_J_221_Rimp01	57.66	6	0	6		0	1	1	0	3	N	12580	CebrOK033_J_221_Rimp01
**total 419 proteins**															

OK193_E_272_PDI

back to list

| Protein Coverage | Supporting Peptides |

Protein Coverage:

Oxidation (M) (+15.99)

```
1    MKFSSCLVLT LLVFVSAEEV KREEDVYVLT TKNFDSFIAD NEFVLVEFYA PWCGHCKALA PEYAKAATAL EEQNLNVKLG
81   KVDATVEDSL AAKFEVRGYP TIKFFRKDKP KSPADYTGGR QSDDIINWLK KKTGPPAKEL KEKDDVKSFV EKDEVVVIGF
                                            198
161  FKDQESTGAL AFKKAAASID DIPFAITSED HVFKEYKMDK DGVVLLKKFD EGRNDFEGEF EEEAIVKHVR ENQLPLVVEF
241  TQESAQKIFG GEVKNHILLF LKKEGGEDTI EKFRGAAEDF KGKVLFIYLD TDNEENGRIT EFFGLKDDEI PAVRLIQLAE
321  DMSKYKPESS DLETATIKKF VQDFLDGKLK PHLMSEDVPD DWDAKPVKIL VGKNFKEVAM DKSKAVFVEF YAPWCGHCKQ
                                423
401  LAPIWDELGE KYKDSKDIVV AKMDATANEI EEVKVQSFPT LKYFPKDSEE AVDYNGERTL DAFVKFLESG GTEGAGVPED
481  EEEEEEDEEG DDEDLPRDEL
```

Supporting Peptides:

Peptide	Uniq	-10lgP	Mass	Length	ppm	m/z	z	RT	Fraction	Scan	Source File	Area ebraeus	Area judaeus	#Feature	#Feature ebraeus	#Feature judaeus	Start	End	PTM	AScore	Found By
K.AAASIDDIPFAITSEDHVFK.E	N	139.79	2146.0581	20	-1.5	716.3559	3	51.84	2	F2:121947	20210611-09_2021021-OK160A_816_5.3.236.mgf	0	0	0	0	0	175	194			PEAKS DB
K.YKPESSDLETATIK.K	N	126.50	1580.7933	14	3.8	791.4036	2	25.19	2	F2:75149	20210611-09_2021021-OK160A_816_5.3.236.mgf	0	0	0	0	0	325	338			PEAKS DB
R.ENQLPLVVEFTQESAQK.I	N	122.23	1958.9949	17	-2.6	654.0012	3	51.77	2	F2:121715	20210611-09_2021021-OK160A_816_5.3.236.mgf	0	0	0	0	0	231	247			PEAKS DB
K.AATALEEQNLNVK.L	N	120.90	1399.7307	13	4.4	700.8728	2	27.82	2	F2:79371	20210611-09_2021021-OK160A_816_5.3.236.mgf	0	0	0	0	0	66	78			PEAKS DB
K.LKPHLM(+15.99)SEDVPDDWDAKPVK.I	N	120.27	2335.1519	20	3.0	779.3903	3	29.56	2	F2:82514	20210611-09_2021021-OK160A_816_5.3.236.mgf	0	0	0	0	0	349	368		M6:Oxidation (M):1000.00	PEAKS DB
R.NDFEGEFEEEAIVK.H	N	117.74	1654.7362	14	1.2	828.3729	2	42.82	2	F2:106304	20210611-09_2021021-OK160A_816_5.3.236.mgf	0	0	0	0	0	214	227			PEAKS DB
K.DSEEAVDYNGER.T	N	116.33	1382.5586	12	-6.2	692.2794	2	19.77	2	F2:65987	20210611-09_2021021-OK160A_816_5.3.236.mgf	0	0	0	0	0	447	458			PEAKS DB
K.MDATANEIEEVK.V	N	113.62	1348.6180	12	2.7	675.3153	2	28.09	2	F2:79934	20210611-09_2021021-OK160A_816_5.3.236.mgf		0	0	0	0	423	434			PEAKS DB
R.ITEFFGLKDDEIPAVR.L	N	113.32	1848.9620	16	-0.6	617.3253	3	47.36	1	F1:45510	20210611-08_2021021-OK192A_815_5.3.236.mgf	0	0	0	0	0	299	314			PEAKS DB
K.KAAASIDDIPFAITSEDHVFK.E	N	110.26	2274.1531	21	0.5	759.0555	3	47.20	2	F2:113862	20210611-09_2021021-OK160A_816_5.3.236.mgf		0	0	0	0	174	194			PEAKS DB
K.LKPHLMSEDVPDDWDAKPVK.I	N	106.07	2319.1567	20	7.8	774.0623	3	33.33	2	F2:89240	20210611-09_2021021-OK160A_816_5.3.236.mgf	0	0	0	0	0	349	368			PEAKS DB
K.M(+15.99)DATANEIEEVK.V	N	99.38	1364.6129	12	-1.0	683.3102	2	25.80	2	F2:75953	20210611-09_2021021-OK160A_816_5.3.236.mgf	0	0	0	0	0	423	434	Oxidation (M)	M1:Oxidation (M):1000.00	PEAKS DB
R.LIQLAEDMSK.Y	N	97.87	1146.5955	10	2.2	574.3038	2	34.62	2	F2:91986	20210611-09_2021021-OK160A_816_5.3.236.mgf	0	0	0	0	0	315	324			PEAKS DB
K.KFVQDFLDGK.L	N	95.51	1195.6237	10	2.0	598.8178	2	34.51	2	F2:91583	20210611-09_2021021-OK160A_816_5.3.236.mgf		0	0	0	0	339	348			PEAKS DB
K.Q(-17.03)LAPIWDELGEK.Y	N	95.23	1380.6925	12	-1.5	691.3496	2	59.13	2	F2:131431	20210611-09_2021021-OK160A_816_5.3.236.mgf	0	0	0	0	0	400	411		Q1:Pyro-glu from Q:1000.00	PEAKS DB
R.LIQLAEDM(+15.99)SK.Y	N	94.94	1162.5903	10	1.4	582.3008	2	26.69	2	F2:77509	20210611-09_2021021-OK160A_816_5.3.236.mgf	0	0	0	0	0	315	324		M8:Oxidation (M):1000.00	PEAKS DB
K.DQESTGALAFK.K	N	94.65	1165.5615	11	4.3	583.7881	2	28.45	2	F2:80728	20210611-09_2021021-OK160A_816_5.3.236.mgf	0	0	0	0	0	163	173			PEAKS DB
K.REEDVYVLTTK.N	N	89.94	1351.6984	11	1.1	676.8544	2	26.32	2	F2:77138	20210611-09_2021021-OK160A_816_5.3.236.mgf	0	0	0	0	0	22	32			PEAKS DB
R.EEDVYVLTTK.N	N	89.68	1195.5972	10	1.5	598.8045	2	31.54	1	F1:27383	20210611-08_2021021-OK192A_815_5.3.236.mgf	0	0	0	0	0	23	32			PEAKS DB
K.VDATVEDSLAAK.F	Y	88.57	1217.6139	12	1.7	609.8129	2	26.56	1	F1:20640	20210611-08_2021021-OK192A_815_5.3.236.mgf	0		0	0	0	82	93			PEAKS DB
K.MDKDGVVLLK.K	N	87.62	1116.6212	10	1.6	559.3164	2	27.37	2	F2:78640	20210611-09_2021021-OK160A_816_5.3.236.mgf		0	0	0	0	198	207			PEAKS DB
K.NHILLFLK.K	N	86.51	996.6120	8	3.6	499.3132	2	39.74	1	F1:37317	20210611-08_2021021-OK192A_815_5.3.236.mgf	0	0	0	0	0	255	262			PEAKS DB
R.E(-18.01)NQLPLVVEFTQESAQK.I	N	86.27	1940.9843	17	-6.1	971.4894	2	58.57	2	F2:130865	20210611-09_2021021-OK160A_816_5.3.236.mgf		0	0	0	0	231	247		E1:Pyro-glu from E:1000.00	PEAKS DB
K.QLAPIWDELGEK.Y	N	82.76	1397.7190	12	0.5	699.8645	2	48.72	1	F1:46550	20210611-08_2021021-OK192A_815_5.3.236.mgf	0	0	0	0	0	400	411			PEAKS DB
K.M(+15.99)DKDGVVLLK.K	N	80.81	1132.6162	10	0.4	567.3132	2	24.19	2	F2:73149	20210611-09_2021021-OK160A_816_5.3.236.mgf		0	0	0	0	198	207	Oxidation (M)	M1:Oxidation (M):1000.00	PEAKS DB
K.FVQDFLDGK.L	N	79.93	1067.5287	9	1.0	534.7699	2	37.97	2	F2:97970	20210611-09_2021021-OK160A_816_5.3.236.mgf	0	0	0	0	0	340	348			PEAKS DB
K.NHILLFLKK.E	N	79.31	1124.7069	9	5.4	375.9102	3	30.94	1	F1:26438	20210611-08_2021021-OK192A_815_5.3.236.mgf	0	0	0	0	0	255	263			PEAKS DB
K.VQSFPTLK.Y	N	75.55	918.5175	8	3.7	460.2658	2	29.78	2	F2:82955	20210611-09_2021021-OK160A_816_5.3.236.mgf	0	0	0	0	0	435	442			PEAKS DB
K.FLESGGTEGAGVPEDEEEEEEDEEGDDEDLPR.D	N	73.31	3508.3921	32	13.2	1170.4823	3	38.27	1	F1:35747	20210611-08_2021021-OK192A_815_5.3.236.mgf	0		0	0	0	466	497			PEAKS DB
K.SPADYTGGR.Q	Y	73.22	922.4144	9	0.9	462.2132	2	14.63	1	F1:5853	20210611-08_2021021-OK192A_815_5.3.236.mgf	0		0	0	0	112	120			PEAKS DB
K.DDEIPAVR.L	N	71.69	913.4504	8	0.8	457.7310	2	24.89	2	F2:74398	20210611-09_2021021-OK160A_816_5.3.236.mgf	0	0	0	0	0	307	314			PEAKS DB
R.E(-18.01)EDVYVLTTK.N	N	69.76	1177.5867	10	4.8	589.8010	2	31.52	2	F2:85902	20210611-09_2021021-OK160A_816_5.3.236.mgf		0	0	0	0	23	32		E1:Pyro-glu from E:1000.00	PEAKS DB
R.ITEFFGLK.D	N	66.91	953.5222	8	0.9	477.7668	2	42.32	2	F2:105336	20210611-09_2021021-OK160A_816_5.3.236.mgf	0	0	0	0	0	299	306			PEAKS DB
R.QSDDIINWLK.K	Y	64.68	1230.6244	10	-0.3	616.3170	2	48.47	1	F1:46302	20210611-08_2021021-OK192A_815_5.3.236.mgf	0		0	0	0	121	130			PEAKS DB
R.TLDAFVK.F	N	64.57	792.4381	7	2.8	397.2258	2	28.98	2	F2:81609	20210611-09_2021021-OK160A_816_5.3.236.mgf	0	0	0	0	0	459	465			PEAKS DB
**total 39 peptides**																					

Peptide	Uniq	-10lgP	Mass	Length	ppm	m/z	z	RT	Fraction	Scan	Source File	Area ebraeus	Area judaeus	#Feature	#Feature ebraeus	#Feature judaeus	Start	End	PTM	AScore	Found By
K.ALAPEYAK.A	N	62.34	861.4596	8	3.5	431.7368	2	19.50	2	F2:65275	20210611-09_2021021-OK160A_816_5.3.236.mgf	0	0	0	0	0	58	65			PEAKS DB
K.DGVVLLK.K	N	61.62	742.4589	7	1.0	372.2355	2	30.45	2	F2:84009	20210611-09_2021021-OK160A_816_5.3.236.mgf	0	0	0	0	0	201	207			PEAKS DB
K.EGGEDTIEK.F	N	59.47	976.4349	9	3.4	489.2243	2	12.93	2	F2:56083	20210611-09_2021021-OK160A_816_5.3.236.mgf		0	0	0	0	264	272			PEAKS DB
K.IFGGEVK.N	N	59.09	748.4119	7	4.2	375.2134	2	21.69	1	F1:13485	20210611-08_2021021-OK192A_815_5.3.236.mgf	0	0	0	0	0	248	254			PEAKS DB
total 39 peptides																					

SRX1323887_E_247_PDI

back to list

| Protein Coverage | Supporting Peptides |

Protein Coverage:

O Oxidation (M) (+15.99)

```
1   MKFSSCLVLT LLVFVSAEEV KREEDVYVLT TKNFDSFIAD NEFVLVEFYA PWCGHCKALA PEYAKAATAL EEQNLNVKLG
81  KVDATVEDSL AAKFEVRGYP TIKFFRKDKP KSPADYTGGR QSDDIINWLK KKTGPPAKEL KEKDDVKSFV EKDEVVVIGF
                                         198
161 FKDQESTGAL AFKKAAASID DIPFAITSED HVFKEYKMDK DGVVLLKKFD EGRNDFEGEF EEEAIVKHVR ENQLPLVVEF
241 TQESAQKIFG GEVKNHILLF LKKEGGEDTI EKFRGAAEDF KGKVLFIYLD TDNEENGRIT EFFGLKDDEI PAVRLIQLAE
321 DMSKYKPESS DLETATIKKF VQDFLDGKLK PHLMSEDVPD DWDAKPVKIL VGKNFKEVAM DKSKAVFVEF YAPWCGHCKQ
                  423
401 LAPIWDELGE KYKDSKDIVV AKMDATANEI EEVKVQSFPT LKYFPKDSEE AVDYNGERTL DAFVKFLESG GTEGAGVPED
481 EEEEEEDEEG DDEDLPRDEL
```

Supporting Peptides:

Peptide	Uniq	-10lgP	Mass	Length	ppm	m/z	z	RT	Fraction	Scan	Source File	Area ebraeus	Area judaeus	#Feature	#Feature ebraeus	#Feature judaeus	Start	End	PTM	AScore	Found By
K.AAASIDDIPFAITSEDHVFK.E	N	139.79	2146.0581	20	-1.5	716.3559	3	51.84	2	F2:121947	20210611-09_2021021-OK160A_816_5.3.236.mgf	0	0	0	0	0	175	194			PEAKS DB
K.YKPESSDLETATIK.K	N	126.50	1580.7933	14	3.8	791.4036	2	25.19	2	F2:75149	20210611-09_2021021-OK160A_816_5.3.236.mgf	0	0	0	0	0	325	338			PEAKS DB
R.ENQLPLVVEFTQESAQK.I	N	122.23	1958.9949	17	-2.6	654.0012	3	51.77	2	F2:121715	20210611-09_2021021-OK160A_816_5.3.236.mgf	0	0	0	0	0	231	247			PEAKS DB
K.AATALEEQNLNVK.L	N	120.90	1399.7307	13	4.4	700.8728	2	27.82	2	F2:79371	20210611-09_2021021-OK160A_816_5.3.236.mgf	0	0	0	0	0	66	78			PEAKS DB
K.LKPHLM(+15.99)SEDVPDDWDAKPVK.I	N	120.27	2335.1519	20	3.0	779.3903	3	29.56	2	F2:82514	20210611-09_2021021-OK160A_816_5.3.236.mgf	0	0	0	0	0	349	368		M6:Oxidation (M):1000.00	PEAKS DB
R.NDFEGEFEEEAIVK.H	N	117.74	1654.7362	14	1.2	828.3729	2	42.82	2	F2:106304	20210611-09_2021021-OK160A_816_5.3.236.mgf	0	0	0	0	0	214	227			PEAKS DB
K.DSEEAVDYNGER.T	N	116.33	1382.5586	12	-6.2	692.2794	2	19.77	2	F2:65987	20210611-09_2021021-OK160A_816_5.3.236.mgf	0	0	0	0	0	447	458			PEAKS DB
K.MDATANEIEEVK.V	N	113.62	1348.6180	12	2.7	675.3153	2	28.09	2	F2:79934	20210611-09_2021021-OK160A_816_5.3.236.mgf		0	0	0	0	423	434			PEAKS DB
R.ITEFFGLKDDEIPAVR.L	N	113.32	1848.9620	16	-0.6	617.3253	3	47.36	1	F1:45510	20210611-08_2021021-OK192A_815_5.3.236.mgf	0	0	0	0	0	299	314			PEAKS DB
K.KAAASIDDIPFAITSEDHVFK.E	N	110.26	2274.1531	21	0.5	759.0555	3	47.20	2	F2:113862	20210611-09_2021021-OK160A_816_5.3.236.mgf		0	0	0	0	174	194			PEAKS DB
K.LKPHLMSEDVPDDWDAKPVK.I	N	106.07	2319.1567	20	7.8	774.0623	3	33.33	2	F2:89240	20210611-09_2021021-OK160A_816_5.3.236.mgf	0	0	0	0	0	349	368			PEAKS DB
K.M(+15.99)DATANEIEEVK.V	N	99.38	1364.6129	12	-1.0	683.3102	2	25.80	2	F2:75953	20210611-09_2021021-OK160A_816_5.3.236.mgf	0	0	0	0	0	423	434	Oxidation (M)	M1:Oxidation (M):1000.00	PEAKS DB
R.LIQLAEDMSK.Y	N	97.87	1146.5955	10	2.2	574.3038	2	34.62	2	F2:91986	20210611-09_2021021-OK160A_816_5.3.236.mgf	0	0	0	0	0	315	324			PEAKS DB
K.KFVQDFLDGK.L	N	95.51	1195.6237	10	2.0	598.8178	2	34.51	2	F2:91583	20210611-09_2021021-OK160A_816_5.3.236.mgf		0	0	0	0	339	348			PEAKS DB
K.Q(-17.03)LAPIWDELGEK.Y	N	95.23	1380.6925	12	-1.5	691.3496	2	59.13	2	F2:131431	20210611-09_2021021-OK160A_816_5.3.236.mgf	0	0	0	0	0	400	411		Q1:Pyro-glu from Q:1000.00	PEAKS DB
R.LIQLAEDM(+15.99)SK.Y	N	94.94	1162.5903	10	1.4	582.3008	2	26.69	2	F2:77509	20210611-09_2021021-OK160A_816_5.3.236.mgf	0	0	0	0	0	315	324		M8:Oxidation (M):1000.00	PEAKS DB
K.DQESTGALAFK.K	N	94.65	1165.5615	11	4.3	583.7881	2	28.45	2	F2:80728	20210611-09_2021021-OK160A_816_5.3.236.mgf	0	0	0	0	0	163	173			PEAKS DB
K.REEDVYVLTTK.N	N	89.94	1351.6984	11	1.1	676.8544	2	26.32	2	F2:77138	20210611-09_2021021-OK160A_816_5.3.236.mgf	0	0	0	0	0	22	32			PEAKS DB
R.EEDVYVLTTK.N	N	89.68	1195.5972	10	1.5	598.8045	2	31.54	1	F1:27383	20210611-08_2021021-OK192A_815_5.3.236.mgf	0	0	0	0	0	23	32			PEAKS DB
K.VDATVEDSLAAK.F	Y	88.57	1217.6139	12	1.7	609.8129	2	26.56	1	F1:20640	20210611-08_2021021-OK192A_815_5.3.236.mgf	0		0	0	0	82	93			PEAKS DB
K.MDKDGVVLLK.K	N	87.62	1116.6212	10	1.6	559.3164	2	27.37	2	F2:78640	20210611-09_2021021-OK160A_816_5.3.236.mgf		0	0	0	0	198	207			PEAKS DB
K.NHILLFLK.K	N	86.51	996.6120	8	3.6	499.3132	2	39.74	1	F1:37317	20210611-08_2021021-OK192A_815_5.3.236.mgf	0	0	0	0	0	255	262			PEAKS DB
R.E(-18.01)NQLPLVVEFTQESAQK.I	N	86.27	1940.9843	17	-6.1	971.4894	2	58.57	2	F2:130865	20210611-09_2021021-OK160A_816_5.3.236.mgf		0	0	0	0	231	247		E1:Pyro-glu from E:1000.00	PEAKS DB
K.QLAPIWDELGEK.Y	N	82.76	1397.7190	12	0.5	699.8645	2	48.72	1	F1:46550	20210611-08_2021021-OK192A_815_5.3.236.mgf	0	0	0	0	0	400	411			PEAKS DB
K.M(+15.99)DKDGVVLLK.K	N	80.81	1132.6162	10	0.4	567.3132	2	24.19	2	F2:73149	20210611-09_2021021-OK160A_816_5.3.236.mgf		0	0	0	0	198	207	Oxidation (M)	M1:Oxidation (M):1000.00	PEAKS DB
K.FVQDFLDGK.L	N	79.93	1067.5287	9	1.0	534.7699	2	37.97	2	F2:97970	20210611-09_2021021-OK160A_816_5.3.236.mgf	0	0	0	0	0	340	348			PEAKS DB
K.NHILLFLKK.E	N	79.31	1124.7069	9	5.4	375.9102	3	30.94	1	F1:26438	20210611-08_2021021-OK192A_815_5.3.236.mgf	0	0	0	0	0	255	263			PEAKS DB
K.VQSFPTLK.Y	N	75.55	918.5175	8	3.7	460.2658	2	29.78	2	F2:82955	20210611-09_2021021-OK160A_816_5.3.236.mgf	0	0	0	0	0	435	442			PEAKS DB
K.FLESGGTEGAGVPEDEEEEEEDEEGDDEDLPR.D	N	73.31	3508.3921	32	13.2	1170.4823	3	38.27	1	F1:35747	20210611-08_2021021-OK192A_815_5.3.236.mgf	0	0	0	0	0	466	497			PEAKS DB
K.SPADYTGGR.Q	Y	73.22	922.4144	9	0.9	462.2132	2	14.63	1	F1:5853	20210611-08_2021021-OK192A_815_5.3.236.mgf	0	0	0	0	0	112	120			PEAKS DB
K.DDEIPAVR.L	N	71.69	913.4504	8	0.8	457.7310	2	24.89	2	F2:74398	20210611-09_2021021-OK160A_816_5.3.236.mgf	0	0	0	0	0	307	314			PEAKS DB
R.E(-18.01)EDVYVLTTK.N	N	69.76	1177.5867	10	4.8	589.8010	2	31.52	2	F2:85902	20210611-09_2021021-OK160A_816_5.3.236.mgf		0	0	0	0	23	32		E1:Pyro-glu from E:1000.00	PEAKS DB
R.ITEFFGLK.D	N	66.91	953.5222	8	0.9	477.7668	2	42.32	2	F2:105336	20210611-09_2021021-OK160A_816_5.3.236.mgf	0	0	0	0	0	299	306			PEAKS DB
R.QSDDIINWLK.K	Y	64.68	1230.6244	10	-0.3	616.3170	2	48.47	1	F1:46302	20210611-08_2021021-OK192A_815_5.3.236.mgf	0	0	0	0	0	121	130			PEAKS DB
R.TLDAFVK.F	N	64.57	792.4381	7	2.8	397.2258	2	28.98	2	F2:81609	20210611-09_2021021-OK160A_816_5.3.236.mgf	0	0	0	0	0	459	465			PEAKS DB
total 39 peptides																					

Peptide	Uniq	-10lgP	Mass	Length	ppm	m/z	z	RT	Fraction	Scan	Source File	Area ebraeus	Area judaeus	#Feature	#Feature ebraeus	#Feature judaeus	Start	End	PTM	AScore	Found By
K.ALAPEYAK.A	N	62.34	861.4596	8	3.5	431.7368	2	19.50	2	F2:65275	20210611-09_2021021-OK160A_816_5.3.236.mgf	0	0	0	0	0	58	65			PEAKS DB
K.DGVVLLK.K	N	61.62	742.4589	7	1.0	372.2355	2	30.45	2	F2:84009	20210611-09_2021021-OK160A_816_5.3.236.mgf	0	0	0	0	0	201	207			PEAKS DB
K.EGGEDTIEK.F	N	59.47	976.4349	9	3.4	489.2243	2	12.93	2	F2:56083	20210611-09_2021021-OK160A_816_5.3.236.mgf		0	0	0	0	264	272			PEAKS DB
K.IFGGEVK.N	N	59.09	748.4119	7	4.2	375.2134	2	21.69	1	F1:13485	20210611-08_2021021-OK192A_815_5.3.236.mgf	0	0	0	0	0	248	254			PEAKS DB

total 39 peptides

OK006_E_295_PDI

back to list

| Protein Coverage | Supporting Peptides |

Protein Coverage:

1 MKFSSCLVLT LLVFVSAEEV KREEDVYVLT TKNFDSFIAD NEFVLVEFYA PWCGHCKALA PEYAKAATAL EEQNLNVKLG

Oxidation (M) (+15.99)

81 KVDATVEDSL AAKFEVRGYP TIKFFRKDKP KSPADYTGGR QSDDIINWLK KKTGPPAKEL KEKDDVKSFV EKDEVVVIGF

198

161 FKDQESTGAL AFKKAAASID DIPFAITSED HVFKEYKMDK DGVVLLKKFD EGRNDFEGEF EEEAIVKHVR ENQLPLVVEF

241 TQESAQKIFG GEVKNHILLF LKKEGGEDTI EKFRGAAEDF KGKVLFIYLD TDNEENGRIT EFFGLKDDEI PAVRLIQLAE

321 DMSKYKPESS DLETATIKKF VQDFLDGKLK PHLMSEDVPD DWDAKPVKIL VGKNFKEVAM DKSKAVFVEF YAPWCGHCKQ

423

401 LAPIWDELGE KYKDSKDIVV AKMDATANEI EEVKVQSFPT LKYFPKDSEE AVDYNGERTL DAFVKFLESG GTEGAGVPED

481 EEEEEEDEEG DDEDLPRDEL

Supporting Peptides:

Peptide	Uniq	-10lgP	Mass	Length	ppm	m/z	z	RT	Fraction	Scan	Source File	Area ebraeus	Area judaeus	#Feature	#Feature ebraeus	#Feature judaeus	Start	End	PTM	AScore	Found By
K.AAASIDDIPFAITSEDHVFK.E	N	139.79	2146.0581	20	-1.5	716.3559	3	51.84	2	F2:121947	20210611-09_2021021-OK160A_816_5.3.236.mgf	0	0	0	0	0	175	194			PEAKS DB
K.YKPESSDLETATIK.K	N	126.50	1580.7933	14	3.8	791.4036	2	25.19	2	F2:75149	20210611-09_2021021-OK160A_816_5.3.236.mgf	0	0	0	0	0	325	338			PEAKS DB
R.ENQLPLVVEFTQESAQK.I	N	122.23	1958.9949	17	-2.6	654.0012	3	51.77	2	F2:121715	20210611-09_2021021-OK160A_816_5.3.236.mgf	0	0	0	0	0	231	247			PEAKS DB
K.AATALEEQNLNVK.L	N	120.90	1399.7307	13	4.4	700.8728	2	27.82	2	F2:79371	20210611-09_2021021-OK160A_816_5.3.236.mgf	0	0	0	0	0	66	78			PEAKS DB
K.LKPHLM(+15.99)SEDVPDDWDAKPVK.I	N	120.27	2335.1519	20	3.0	779.3903	3	29.56	2	F2:82514	20210611-09_2021021-OK160A_816_5.3.236.mgf	0	0	0	0	0	349	368		M6:Oxidation (M):1000.00	PEAKS DB
R.NDFEGEFEEEAIVK.H	N	117.74	1654.7362	14	1.2	828.3729	2	42.82	2	F2:106304	20210611-09_2021021-OK160A_816_5.3.236.mgf	0	0	0	0	0	214	227			PEAKS DB
K.DSEEAVDYNGER.T	N	116.33	1382.5586	12	-6.2	692.2794	2	19.77	2	F2:65987	20210611-09_2021021-OK160A_816_5.3.236.mgf	0	0	0	0	0	447	458			PEAKS DB
K.MDATANEIEEVK.V	N	113.62	1348.6180	12	2.7	675.3153	2	28.09	2	F2:79934	20210611-09_2021021-OK160A_816_5.3.236.mgf		0	0	0	0	423	434			PEAKS DB
R.ITEFFGLKDDEIPAVR.L	N	113.32	1848.9620	16	-0.6	617.3253	3	47.36	1	F1:45510	20210611-08_2021021-OK192A_815_5.3.236.mgf	0	0	0	0	0	299	314			PEAKS DB
K.KAAASIDDIPFAITSEDHVFK.E	N	110.26	2274.1531	21	0.5	759.0555	3	47.20	2	F2:113862	20210611-09_2021021-OK160A_816_5.3.236.mgf		0	0	0	0	174	194			PEAKS DB
K.LKPHLMSEDVPDDWDAKPVK.I	N	106.07	2319.1567	20	7.8	774.0623	3	33.33	2	F2:89240	20210611-09_2021021-OK160A_816_5.3.236.mgf	0	0	0	0	0	349	368			PEAKS DB
K.M(+15.99)DATANEIEEVK.V	N	99.38	1364.6129	12	-1.0	683.3102	2	25.80	2	F2:75953	20210611-09_2021021-OK160A_816_5.3.236.mgf	0	0	0	0	0	423	434	Oxidation (M)	M1:Oxidation (M):1000.00	PEAKS DB
R.LIQLAEDMSK.Y	N	97.87	1146.5955	10	2.2	574.3038	2	34.62	2	F2:91986	20210611-09_2021021-OK160A_816_5.3.236.mgf	0	0	0	0	0	315	324			PEAKS DB
K.KFVQDFLDGK.L	N	95.51	1195.6237	10	2.0	598.8178	2	34.51	2	F2:91583	20210611-09_2021021-OK160A_816_5.3.236.mgf		0	0	0	0	339	348			PEAKS DB
K.Q(-17.03)LAPIWDELGEK.Y	N	95.23	1380.6925	12	-1.5	691.3496	2	59.13	2	F2:131431	20210611-09_2021021-OK160A_816_5.3.236.mgf	0	0	0	0	0	400	411		Q1:Pyro-glu from Q:1000.00	PEAKS DB
R.LIQLAEDM(+15.99)SK.Y	N	94.94	1162.5903	10	1.4	582.3008	2	26.69	2	F2:77509	20210611-09_2021021-OK160A_816_5.3.236.mgf	0	0	0	0	0	315	324		M8:Oxidation (M):1000.00	PEAKS DB
K.DQESTGALAFK.K	N	94.65	1165.5615	11	4.3	583.7881	2	28.45	2	F2:80728	20210611-09_2021021-OK160A_816_5.3.236.mgf	0	0	0	0	0	163	173			PEAKS DB
K.REEDVYVLTTK.N	N	89.94	1351.6984	11	1.1	676.8544	2	26.32	2	F2:77138	20210611-09_2021021-OK160A_816_5.3.236.mgf	0	0	0	0	0	22	32			PEAKS DB
R.EEDVYVLTTK.N	N	89.68	1195.5972	10	1.5	598.8045	2	31.54	1	F1:27383	20210611-08_2021021-OK192A_815_5.3.236.mgf	0	0	0	0	0	23	32			PEAKS DB
K.VDATVEDSLAAK.F	Y	88.57	1217.6139	12	1.7	609.8129	2	26.56	1	F1:20640	20210611-08_2021021-OK192A_815_5.3.236.mgf	0		0	0	0	82	93			PEAKS DB
K.MDKDGVVLLK.K	N	87.62	1116.6212	10	1.6	559.3164	2	27.37	2	F2:78640	20210611-09_2021021-OK160A_816_5.3.236.mgf		0	0	0	0	198	207			PEAKS DB
K.NHILLFLK.K	N	86.51	996.6120	8	3.6	499.3132	2	39.74	1	F1:37317	20210611-08_2021021-OK192A_815_5.3.236.mgf	0	0	0	0	0	255	262			PEAKS DB
R.E(-18.01)NQLPLVVEFTQESAQK.I	N	86.27	1940.9843	17	-6.1	971.4894	2	58.57	2	F2:130865	20210611-09_2021021-OK160A_816_5.3.236.mgf		0	0	0	0	231	247		E1:Pyro-glu from E:1000.00	PEAKS DB
K.QLAPIWDELGEK.Y	N	82.76	1397.7190	12	0.5	699.8645	2	48.72	1	F1:46550	20210611-08_2021021-OK192A_815_5.3.236.mgf	0	0	0	0	0	400	411			PEAKS DB
K.M(+15.99)DKDGVVLLK.K	N	80.81	1132.6162	10	0.4	567.3132	2	24.19	2	F2:73149	20210611-09_2021021-OK160A_816_5.3.236.mgf		0	0	0	0	198	207	Oxidation (M)	M1:Oxidation (M):1000.00	PEAKS DB
K.FVQDFLDGK.L	N	79.93	1067.5287	9	1.0	534.7699	2	37.97	2	F2:97970	20210611-09_2021021-OK160A_816_5.3.236.mgf	0	0	0	0	0	340	348			PEAKS DB
K.NHILLFLKK.E	N	79.31	1124.7069	9	5.4	375.9102	3	30.94	1	F1:26438	20210611-08_2021021-OK192A_815_5.3.236.mgf	0	0	0	0	0	255	263			PEAKS DB
K.VQSFPTLK.Y	N	75.55	918.5175	8	3.7	460.2658	2	29.78	2	F2:82955	20210611-09_2021021-OK160A_816_5.3.236.mgf	0	0	0	0	0	435	442			PEAKS DB
K.FLESGGTEGAGVPEDEEEEEEDEEGDDEDLPR.D	N	73.31	3508.3921	32	13.2	1170.4823	3	38.27	1	F1:35747	20210611-08_2021021-OK192A_815_5.3.236.mgf	0		0	0	0	466	497			PEAKS DB
K.SPADYTGGR.Q	Y	73.22	922.4144	9	0.9	462.2132	2	14.63	1	F1:5853	20210611-08_2021021-OK192A_815_5.3.236.mgf	0		0	0	0	112	120			PEAKS DB
K.DDEIPAVR.L	N	71.69	913.4504	8	0.8	457.7310	2	24.89	2	F2:74398	20210611-09_2021021-OK160A_816_5.3.236.mgf	0	0	0	0	0	307	314			PEAKS DB
R.E(-18.01)EDVYVLTTK.N	N	69.76	1177.5867	10	4.8	589.8010	2	31.52	2	F2:85902	20210611-09_2021021-OK160A_816_5.3.236.mgf		0	0	0	0	23	32		E1:Pyro-glu from E:1000.00	PEAKS DB
R.ITEFFGLK.D	N	66.91	953.5222	8	0.9	477.7668	2	42.32	2	F2:105336	20210611-09_2021021-OK160A_816_5.3.236.mgf	0	0	0	0	0	299	306			PEAKS DB
R.QSDDIINWLK.K	Y	64.68	1230.6244	10	-0.3	616.3170	2	48.47	1	F1:46302	20210611-08_2021021-OK192A_815_5.3.236.mgf	0		0	0	0	121	130			PEAKS DB
R.TLDAFVK.F	N	64.57	792.4381	7	2.8	397.2258	2	28.98	2	F2:81609	20210611-09_2021021-OK160A_816_5.3.236.mgf	0	0	0	0	0	459	465			PEAKS DB

total 39 peptides

Peptide	Uniq	-10lgP	Mass	Length	ppm	m/z	z	RT	Fraction	Scan	Source File	Area ebraeus	Area judaeus	#Feature	#Feature ebraeus	#Feature judaeus	Start	End	PTM	AScore	Found By
K.ALAPEYAK.A	N	62.34	861.4596	8	3.5	431.7368	2	19.50	2	F2:65275	20210611-09_2021021-OK160A_816_5.3.236.mgf	0	0	0	0	0	58	65			PEAKS DB
K.DGVVLLK.K	N	61.62	742.4589	7	1.0	372.2355	2	30.45	2	F2:84009	20210611-09_2021021-OK160A_816_5.3.236.mgf	0	0	0	0	0	201	207			PEAKS DB
K.EGGEDTIEK.F	N	59.47	976.4349	9	3.4	489.2243	2	12.93	2	F2:56083	20210611-09_2021021-OK160A_816_5.3.236.mgf		0	0	0	0	264	272			PEAKS DB
K.IFGGEVK.N	N	59.09	748.4119	7	4.2	375.2134	2	21.69	1	F1:13485	20210611-08_2021021-OK192A_815_5.3.236.mgf	0	0	0	0	0	248	254			PEAKS DB
**total 39 peptides**																					

OK213_E_326_PDI

back to list

| Protein Coverage | Supporting Peptides |

Protein Coverage:

```
  1 MKFSSCLVLT LLVFVSAEEV KREEDVYVLT TKNFDSFIAD NEFVLVEFYA PWCGHCKALA PEYAKAATAL EEQNLNVKLG
 81 KVDATVEDSL AAKFEVRGYP TIKFFRKDKP KSPADYTGGR QSDDIINWLK KKTGPPAKEL KEKDDVKSFV EKDEVVVIGF
                                             198
161 FKDQESTGAL AFKKAAASID DIPFAITSED HVFKEYKMDK DGVVLLKKFD EGRNDFEGEF EEEAIVKHVR ENQLPLVVEF
241 TQESAQKIFG GEVKNHILLF LKKEGGEDTI EKFRGAAEDF KGKVLFIYLD TDNEENGRIT EFFGLKDDEI PAVRLIQLAE
321 DMSKYKPESS DLETATIKKF VQDFLDGKLK PHLMSEDVPD DWDAKPVKIL VGKNFKEVAM DKSKAVFVEF YAPWCGHCKQ
                                423
401 LAPIWDELGE KYKDSKDIVV AKMDATANEI EEVKVQSFPT LKYFPKDSEE AVDYNGERTL DAFVKFLESG GTEGAGVPED
481 EEEEEEDEEG DDEDLPRDEL
```

O Oxidation (M) (+15.99)

Supporting Peptides:

Peptide	Uniq	-10lgP	Mass	Length	ppm	m/z	z	RT	Fraction	Scan	Source File	Area ebraeus	Area judaeus	#Feature	#Feature ebraeus	#Feature judaeus	Start	End	PTM	AScore	Found By
K.AAASIDDIPFAITSEDHVFK.E	N	139.79	2146.0581	20	-1.5	716.3559	3	51.84	2	F2:121947	20210611-09_2021021-OK160A_816_5.3.236.mgf	0	0	0	0	0	175	194			PEAKS DB
K.YKPESSDLETATIK.K	N	126.50	1580.7933	14	3.8	791.4036	2	25.19	2	F2:75149	20210611-09_2021021-OK160A_816_5.3.236.mgf	0	0	0	0	0	325	338			PEAKS DB
R.ENQLPLVVEFTQESAQK.I	N	122.23	1958.9949	17	-2.6	654.0012	3	51.77	2	F2:121715	20210611-09_2021021-OK160A_816_5.3.236.mgf	0	0	0	0	0	231	247			PEAKS DB
K.AATALEEQNLNVK.L	N	120.90	1399.7307	13	4.4	700.8728	2	27.82	2	F2:79371	20210611-09_2021021-OK160A_816_5.3.236.mgf	0	0	0	0	0	66	78			PEAKS DB
K.LKPHLM(+15.99)SEDVPDDWDAKPVK.I	N	120.27	2335.1519	20	3.0	779.3903	3	29.56	2	F2:82514	20210611-09_2021021-OK160A_816_5.3.236.mgf	0	0	0	0	0	349	368		M6:Oxidation (M):1000.00	PEAKS DB
R.NDFEGEFEEEAIVK.H	N	117.74	1654.7362	14	1.2	828.3729	2	42.82	2	F2:106304	20210611-09_2021021-OK160A_816_5.3.236.mgf	0	0	0	0	0	214	227			PEAKS DB
K.DSEEAVDYNGER.T	N	116.33	1382.5586	12	-6.2	692.2794	2	19.77	2	F2:65987	20210611-09_2021021-OK160A_816_5.3.236.mgf	0	0	0	0	0	447	458			PEAKS DB
K.MDATANEIEEVK.V	N	113.62	1348.6180	12	2.7	675.3153	2	28.09	2	F2:79934	20210611-09_2021021-OK160A_816_5.3.236.mgf		0	0	0	0	423	434			PEAKS DB
R.ITEFFGLKDDEIPAVR.L	N	113.32	1848.9620	16	-0.6	617.3253	3	47.36	1	F1:45510	20210611-08_2021021-OK192A_815_5.3.236.mgf	0	0	0	0	0	299	314			PEAKS DB
K.KAAASIDDIPFAITSEDHVFK.E	N	110.26	2274.1531	21	0.5	759.0555	3	47.20	2	F2:113862	20210611-09_2021021-OK160A_816_5.3.236.mgf		0	0	0	0	174	194			PEAKS DB
K.LKPHLMSEDVPDDWDAKPVK.I	N	106.07	2319.1567	20	7.8	774.0623	3	33.33	2	F2:89240	20210611-09_2021021-OK160A_816_5.3.236.mgf	0	0	0	0	0	349	368			PEAKS DB
K.M(+15.99)DATANEIEEVK.V	N	99.38	1364.6129	12	-1.0	683.3102	2	25.80	2	F2:75953	20210611-09_2021021-OK160A_816_5.3.236.mgf	0	0	0	0	0	423	434	Oxidation (M)	M1:Oxidation (M):1000.00	PEAKS DB
R.LIQLAEDMSK.Y	N	97.87	1146.5955	10	2.2	574.3038	2	34.62	2	F2:91986	20210611-09_2021021-OK160A_816_5.3.236.mgf	0	0	0	0	0	315	324			PEAKS DB
K.KFVQDFLDGK.L	N	95.51	1195.6237	10	2.0	598.8178	2	34.51	2	F2:91583	20210611-09_2021021-OK160A_816_5.3.236.mgf		0	0	0	0	339	348			PEAKS DB
K.Q(-17.03)LAPIWDELGEK.Y	N	95.23	1380.6925	12	-1.5	691.3496	2	59.13	2	F2:131431	20210611-09_2021021-OK160A_816_5.3.236.mgf	0	0	0	0	0	400	411		Q1:Pyro-glu from Q:1000.00	PEAKS DB
R.LIQLAEDM(+15.99)SK.Y	N	94.94	1162.5903	10	1.4	582.3008	2	26.69	2	F2:77509	20210611-09_2021021-OK160A_816_5.3.236.mgf	0	0	0	0	0	315	324		M8:Oxidation (M):1000.00	PEAKS DB
K.DQESTGALAFK.K	N	94.65	1165.5615	11	4.3	583.7881	2	28.45	2	F2:80728	20210611-09_2021021-OK160A_816_5.3.236.mgf	0	0	0	0	0	163	173			PEAKS DB
K.REEDVYVLTTK.N	N	89.94	1351.6984	11	1.1	676.8544	2	26.32	2	F2:77138	20210611-09_2021021-OK160A_816_5.3.236.mgf	0	0	0	0	0	22	32			PEAKS DB
R.EEDVYVLTTK.N	N	89.68	1195.5972	10	1.5	598.8045	2	31.54	1	F1:27383	20210611-08_2021021-OK192A_815_5.3.236.mgf	0	0	0	0	0	23	32			PEAKS DB
K.VDATVEDSLAAK.F	Y	88.57	1217.6139	12	1.7	609.8129	2	26.56	1	F1:20640	20210611-08_2021021-OK192A_815_5.3.236.mgf	0		0	0	0	82	93			PEAKS DB
K.MDKDGVVLLK.K	N	87.62	1116.6212	10	1.6	559.3164	2	27.37	2	F2:78640	20210611-09_2021021-OK160A_816_5.3.236.mgf		0	0	0	0	198	207			PEAKS DB
K.NHILLFLK.K	N	86.51	996.6120	8	3.6	499.3132	2	39.74	1	F1:37317	20210611-08_2021021-OK192A_815_5.3.236.mgf	0	0	0	0	0	255	262			PEAKS DB
R.E(-18.01)NQLPLVVEFTQESAQK.I	N	86.27	1940.9843	17	-6.1	971.4894	2	58.57	2	F2:130865	20210611-09_2021021-OK160A_816_5.3.236.mgf		0	0	0	0	231	247		E1:Pyro-glu from E:1000.00	PEAKS DB
K.QLAPIWDELGEK.Y	N	82.76	1397.7190	12	0.5	699.8645	2	48.72	1	F1:46550	20210611-08_2021021-OK192A_815_5.3.236.mgf	0	0	0	0	0	400	411			PEAKS DB
K.M(+15.99)DKDGVVLLK.K	N	80.81	1132.6162	10	0.4	567.3132	2	24.19	2	F2:73149	20210611-09_2021021-OK160A_816_5.3.236.mgf		0	0	0	0	198	207	Oxidation (M)	M1:Oxidation (M):1000.00	PEAKS DB
K.FVQDFLDGK.L	N	79.93	1067.5287	9	1.0	534.7699	2	37.97	2	F2:97970	20210611-09_2021021-OK160A_816_5.3.236.mgf	0	0	0	0	0	340	348			PEAKS DB
K.NHILLFLKK.E	N	79.31	1124.7069	9	5.4	375.9102	3	30.94	1	F1:26438	20210611-08_2021021-OK192A_815_5.3.236.mgf	0	0	0	0	0	255	263			PEAKS DB
K.VQSFPTLK.Y	N	75.55	918.5175	8	3.7	460.2658	2	29.78	2	F2:82955	20210611-09_2021021-OK160A_816_5.3.236.mgf	0	0	0	0	0	435	442			PEAKS DB
K.FLESGGTEGAGVPEDEEEEEEDEEGDDEDLPR.D	N	73.31	3508.3921	32	13.2	1170.4823	3	38.27	1	F1:35747	20210611-08_2021021-OK192A_815_5.3.236.mgf	0		0	0	0	466	497			PEAKS DB
K.SPADYTGGR.Q	Y	73.22	922.4144	9	0.9	462.2132	2	14.63	1	F1:5853	20210611-08_2021021-OK192A_815_5.3.236.mgf	0		0	0	0	112	120			PEAKS DB
K.DDEIPAVR.L	N	71.69	913.4504	8	0.8	457.7310	2	24.89	2	F2:74398	20210611-09_2021021-OK160A_816_5.3.236.mgf	0	0	0	0	0	307	314			PEAKS DB
R.E(-18.01)EDVYVLTTK.N	N	69.76	1177.5867	10	4.8	589.8010	2	31.52	2	F2:85902	20210611-09_2021021-OK160A_816_5.3.236.mgf		0	0	0	0	23	32		E1:Pyro-glu from E:1000.00	PEAKS DB
R.ITEFFGLK.D	N	66.91	953.5222	8	0.9	477.7668	2	42.32	2	F2:105336	20210611-09_2021021-OK160A_816_5.3.236.mgf	0	0	0	0	0	299	306			PEAKS DB
R.QSDDIINWLK.K	Y	64.68	1230.6244	10	-0.3	616.3170	2	48.47	1	F1:46302	20210611-08_2021021-OK192A_815_5.3.236.mgf	0		0	0	0	121	130			PEAKS DB
R.TLDAFVK.F	N	64.57	792.4381	7	2.8	397.2258	2	28.98	2	F2:81609	20210611-09_2021021-OK160A_816_5.3.236.mgf	0	0	0	0	0	459	465			PEAKS DB
**total 39 peptides**																					

Peptide	Uniq	-10lgP	Mass	Length	ppm	m/z	z	RT	Fraction	Scan	Source File	Area ebraeus	Area judaeus	#Feature	#Feature ebraeus	#Feature judaeus	Start	End	PTM	AScore	Found By
K.ALAPEYAK.A	N	62.34	861.4596	8	3.5	431.7368	2	19.50	2	F2:65275	20210611-09_2021021-OK160A_816_5.3.236.mgf	0	0	0	0	0	58	65			PEAKS DB
K.DGVVLLK.K	N	61.62	742.4589	7	1.0	372.2355	2	30.45	2	F2:84009	20210611-09_2021021-OK160A_816_5.3.236.mgf	0	0	0	0	0	201	207			PEAKS DB
K.EGGEDTIEK.F	N	59.47	976.4349	9	3.4	489.2243	2	12.93	2	F2:56083	20210611-09_2021021-OK160A_816_5.3.236.mgf		0	0	0	0	264	272			PEAKS DB
K.IFGGEVK.N	N	59.09	748.4119	7	4.2	375.2134	2	21.69	1	F1:13485	20210611-08_2021021-OK192A_815_5.3.236.mgf	0	0	0	0	0	248	254			PEAKS DB

total 39 peptides

CebrOK016_E_159_PDI

back to list

| Protein Coverage | Supporting Peptides |

Protein Coverage:

Oxidation (M) (+15.99)

1 MKFSSCLVLT LLVFVSAEEV KREEDVYVLT TKNFDSFIAD NEFVLVEFYA PWCGHCKALA PEYAKAATAL EEQNLNVKLG

81 KVDATVEDSL AAKFEVRGYP TIKFFRKDKP KSPADYTGGR QSDDIINWLK KKTGPPAKEL KEKDDVKSFV EKDEVVVIGF

198

161 FKDQESTGAL AFKKAAASID DIPFAITSED HVFKEYKMDK DGVVLLKKFD EGRNDFEGEF EEEAIVKHVR ENQLPLVVEF

241 TQESAQKIFG GEVKNHILLF LKKEGGEDTI EKFRGAAEDF KGKVLFIYLD TDNEENGRIT EFFGLKDDEI PAVRLIQLAE

321 DMSKYKPESS DLETATIKKF VQDFLDGKLK PHLMSEDVPD DWDAKPVKIL VGKNFKEVAM DKSKAVFVEF YAPWCGHCKQ

423

401 LAPIWDELGE KYKDSKDIVV AKMDATANEI EEVKVQSFPT LKYFPKDSEE AVDYNGERTL DAFVKFLESG GTEGAGVPED

481 EEEEEEDEEG DDEDLPRDEL

Supporting Peptides:

Peptide	Uniq	-10lgP	Mass	Length	ppm	m/z	z	RT	Fraction	Scan	Source File	Area ebraeus	Area judaeus	#Feature	#Feature ebraeus	#Feature judaeus	Start	End	PTM	AScore	Found By
K.AAASIDDIPFAITSEDHVFK.E	N	139.79	2146.0581	20	-1.5	716.3559	3	51.84	2	F2:121947	20210611-09_2021021-OK160A_816_5.3.236.mgf	0	0	0	0	0	175	194			PEAKS DB
K.YKPESSDLETATIK.K	N	126.50	1580.7933	14	3.8	791.4036	2	25.19	2	F2:75149	20210611-09_2021021-OK160A_816_5.3.236.mgf	0	0	0	0	0	325	338			PEAKS DB
R.ENQLPLVVEFTQESAQK.I	N	122.23	1958.9949	17	-2.6	654.0012	3	51.77	2	F2:121715	20210611-09_2021021-OK160A_816_5.3.236.mgf	0	0	0	0	0	231	247			PEAKS DB
K.AATALEEQNLNVK.L	N	120.90	1399.7307	13	4.4	700.8728	2	27.82	2	F2:79371	20210611-09_2021021-OK160A_816_5.3.236.mgf	0	0	0	0	0	66	78			PEAKS DB
K.LKPHLM(+15.99)SEDVPDDWDAKPVK.I	N	120.27	2335.1519	20	3.0	779.3903	3	29.56	2	F2:82514	20210611-09_2021021-OK160A_816_5.3.236.mgf	0	0	0	0	0	349	368		M6:Oxidation (M):1000.00	PEAKS DB
R.NDFEGEFEEEAIVK.H	N	117.74	1654.7362	14	1.2	828.3729	2	42.82	2	F2:106304	20210611-09_2021021-OK160A_816_5.3.236.mgf	0	0	0	0	0	214	227			PEAKS DB
K.DSEEAVDYNGER.T	N	116.33	1382.5586	12	-6.2	692.2794	2	19.77	2	F2:65987	20210611-09_2021021-OK160A_816_5.3.236.mgf	0	0	0	0	0	447	458			PEAKS DB
K.MDATANEIEEVK.V	N	113.62	1348.6180	12	2.7	675.3153	2	28.09	2	F2:79934	20210611-09_2021021-OK160A_816_5.3.236.mgf		0	0	0	0	423	434			PEAKS DB
R.ITEFFGLKDDEIPAVR.L	N	113.32	1848.9620	16	-0.6	617.3253	3	47.36	1	F1:45510	20210611-08_2021021-OK192A_815_5.3.236.mgf	0	0	0	0	0	299	314			PEAKS DB
K.KAAASIDDIPFAITSEDHVFK.E	N	110.26	2274.1531	21	0.5	759.0555	3	47.20	2	F2:113862	20210611-09_2021021-OK160A_816_5.3.236.mgf		0	0	0	0	174	194			PEAKS DB
K.LKPHLMSEDVPDDWDAKPVK.I	N	106.07	2319.1567	20	7.8	774.0623	3	33.33	2	F2:89240	20210611-09_2021021-OK160A_816_5.3.236.mgf	0	0	0	0	0	349	368			PEAKS DB
K.M(+15.99)DATANEIEEVK.V	N	99.38	1364.6129	12	-1.0	683.3102	2	25.80	2	F2:75953	20210611-09_2021021-OK160A_816_5.3.236.mgf	0	0	0	0	0	423	434	Oxidation (M)	M1:Oxidation (M):1000.00	PEAKS DB
R.LIQLAEDMSK.Y	N	97.87	1146.5955	10	2.2	574.3038	2	34.62	2	F2:91986	20210611-09_2021021-OK160A_816_5.3.236.mgf	0	0	0	0	0	315	324			PEAKS DB
K.KFVQDFLDGK.L	N	95.51	1195.6237	10	2.0	598.8178	2	34.51	2	F2:91583	20210611-09_2021021-OK160A_816_5.3.236.mgf		0	0	0	0	339	348			PEAKS DB
K.Q(-17.03)LAPIWDELGEK.Y	N	95.23	1380.6925	12	-1.5	691.3496	2	59.13	2	F2:131431	20210611-09_2021021-OK160A_816_5.3.236.mgf	0	0	0	0	0	400	411		Q1:Pyro-glu from Q:1000.00	PEAKS DB
R.LIQLAEDM(+15.99)SK.Y	N	94.94	1162.5903	10	1.4	582.3008	2	26.69	2	F2:77509	20210611-09_2021021-OK160A_816_5.3.236.mgf	0	0	0	0	0	315	324		M8:Oxidation (M):1000.00	PEAKS DB
K.DQESTGALAFK.K	N	94.65	1165.5615	11	4.3	583.7881	2	28.45	2	F2:80728	20210611-09_2021021-OK160A_816_5.3.236.mgf	0	0	0	0	0	163	173			PEAKS DB
K.REEDVYVLTTK.N	N	89.94	1351.6984	11	1.1	676.8544	2	26.32	2	F2:77138	20210611-09_2021021-OK160A_816_5.3.236.mgf	0	0	0	0	0	22	32			PEAKS DB
R.EEDVYVLTTK.N	N	89.68	1195.5972	10	1.5	598.8045	2	31.54	1	F1:27383	20210611-08_2021021-OK192A_815_5.3.236.mgf	0	0	0	0	0	23	32			PEAKS DB
K.VDATVEDSLAAK.F	Y	88.57	1217.6139	12	1.7	609.8129	2	26.56	1	F1:20640	20210611-08_2021021-OK192A_815_5.3.236.mgf	0		0	0	0	82	93			PEAKS DB
K.MDKDGVVLLK.K	N	87.62	1116.6212	10	1.6	559.3164	2	27.37	2	F2:78640	20210611-09_2021021-OK160A_816_5.3.236.mgf		0	0	0	0	198	207			PEAKS DB
K.NHILLFLK.K	N	86.51	996.6120	8	3.6	499.3132	2	39.74	1	F1:37317	20210611-08_2021021-OK192A_815_5.3.236.mgf	0	0	0	0	0	255	262			PEAKS DB
R.E(-18.01)NQLPLVVEFTQESAQK.I	N	86.27	1940.9843	17	-6.1	971.4894	2	58.57	2	F2:130865	20210611-09_2021021-OK160A_816_5.3.236.mgf		0	0	0	0	231	247		E1:Pyro-glu from E:1000.00	PEAKS DB
K.QLAPIWDELGEK.Y	N	82.76	1397.7190	12	0.5	699.8645	2	48.72	1	F1:46550	20210611-08_2021021-OK192A_815_5.3.236.mgf	0	0	0	0	0	400	411			PEAKS DB
K.M(+15.99)DKDGVVLLK.K	N	80.81	1132.6162	10	0.4	567.3132	2	24.19	2	F2:73149	20210611-09_2021021-OK160A_816_5.3.236.mgf		0	0	0	0	198	207	Oxidation (M)	M1:Oxidation (M):1000.00	PEAKS DB
K.FVQDFLDGK.L	N	79.93	1067.5287	9	1.0	534.7699	2	37.97	2	F2:97970	20210611-09_2021021-OK160A_816_5.3.236.mgf	0	0	0	0	0	340	348			PEAKS DB
K.NHILLFLKK.E	N	79.31	1124.7069	9	5.4	375.9102	3	30.94	1	F1:26438	20210611-08_2021021-OK192A_815_5.3.236.mgf	0	0	0	0	0	255	263			PEAKS DB
K.VQSFPTLK.Y	N	75.55	918.5175	8	3.7	460.2658	2	29.78	2	F2:82955	20210611-09_2021021-OK160A_816_5.3.236.mgf	0	0	0	0	0	435	442			PEAKS DB
K.FLESGGTEGAGVPEDEEEEEEDEEGDDEDLPR.D	N	73.31	3508.3921	32	13.2	1170.4823	3	38.27	1	F1:35747	20210611-08_2021021-OK192A_815_5.3.236.mgf	0		0	0	0	466	497			PEAKS DB
K.SPADYTGGR.Q	Y	73.22	922.4144	9	0.9	462.2132	2	14.63	1	F1:5853	20210611-08_2021021-OK192A_815_5.3.236.mgf	0		0	0	0	112	120			PEAKS DB
K.DDEIPAVR.L	N	71.69	913.4504	8	0.8	457.7310	2	24.89	2	F2:74398	20210611-09_2021021-OK160A_816_5.3.236.mgf	0	0	0	0	0	307	314			PEAKS DB
R.E(-18.01)EDVYVLTTK.N	N	69.76	1177.5867	10	4.8	589.8010	2	31.52	2	F2:85902	20210611-09_2021021-OK160A_816_5.3.236.mgf		0	0	0	0	23	32		E1:Pyro-glu from E:1000.00	PEAKS DB
R.ITEFFGLK.D	N	66.91	953.5222	8	0.9	477.7668	2	42.32	2	F2:105336	20210611-09_2021021-OK160A_816_5.3.236.mgf	0	0	0	0	0	299	306			PEAKS DB
R.QSDDIINWLK.K	Y	64.68	1230.6244	10	-0.3	616.3170	2	48.47	1	F1:46302	20210611-08_2021021-OK192A_815_5.3.236.mgf	0		0	0	0	121	130			PEAKS DB
R.TLDAFVK.F	N	64.57	792.4381	7	2.8	397.2258	2	28.98	2	F2:81609	20210611-09_2021021-OK160A_816_5.3.236.mgf	0	0	0	0	0	459	465			PEAKS DB

total 39 peptides

Peptide	Uniq	-10lgP	Mass	Length	ppm	m/z	z	RT	Fraction	Scan	Source File	Area ebraeus	Area judaeus	#Feature	#Feature ebraeus	#Feature judaeus	Start	End	PTM	AScore	Found By
K.ALAPEYAK.A	N	62.34	861.4596	8	3.5	431.7368	2	19.50	2	F2:65275	20210611-09_2021021-OK160A_816_5.3.236.mgf	0	0	0	0	0	58	65			PEAKS DB
K.DGVVLLK.K	N	61.62	742.4589	7	1.0	372.2355	2	30.45	2	F2:84009	20210611-09_2021021-OK160A_816_5.3.236.mgf	0	0	0	0	0	201	207			PEAKS DB
K.EGGEDTIEK.F	N	59.47	976.4349	9	3.4	489.2243	2	12.93	2	F2:56083	20210611-09_2021021-OK160A_816_5.3.236.mgf		0	0	0	0	264	272			PEAKS DB
K.IFGGEVK.N	N	59.09	748.4119	7	4.2	375.2134	2	21.69	1	F1:13485	20210611-08_2021021-OK192A_815_5.3.236.mgf	0	0	0	0	0	248	254			PEAKS DB

total 39 peptides

CebrOK014_E_145_PDI

back to list

| **Protein Coverage** | **Supporting Peptides** |

Protein Coverage:

Oxidation (M) (+15.99)

```
  1 MKFSSCLVLT LLVFVSAEEV KREEDVYVLT TKNFDSFIAD NEFVLVEFYA PWCGHCKALA PEYAKAATAL EEQNLNVKLG
 81 KVDATVEDSL AAKFEVRGYP TIKFFRKDKP KSPADYTGGR QSDDIINWLK KKTGPPAKEL KEKDDVKSFV EKDEVVVIGF
161 FKDQESTGAL AFKKAAASID DIPFAITSED HVFKEYKMDK DGVVLLKKFD EGRNDFEGEF EEEAIVKHVR ENQLPLVVEF
241 TQESAQKIFG GEVKNHILLF LKKEGGEDTI EKFRGAAEDF KGKVLFIYLD TDNEENGRIT EFFGLKDDEI PAVRLIQLAE
321 DMSKYKPESS DLETATIKKF VQDFLDGKLK PHLMSEDVPD DWDAKPVKIL VGKNFKEVAM DKSKAVFVEF YAPWCGHCKQ
401 LAPIWDELGE KYKDSKDIVV AKMDATANEI EEVKVQSFPT LKYFPKDSEE AVDYNGERTL DAFVKFLESG GTEGAGVPED
481 EEEEEEDEEG DDEDLPRDEL
```

(198: Oxidation (M); 423: Oxidation (M))

Supporting Peptides:

Peptide	Uniq	-10lgP	Mass	Length	ppm	m/z	z	RT	Fraction	Scan	Source File	Area ebraeus	Area judaeus	#Feature	#Feature ebraeus	#Feature judaeus	Start	End	PTM	AScore	Found By
K.AAASIDDIPFAITSEDHVFK.E	N	139.79	2146.0581	20	-1.5	716.3559	3	51.84	2	F2:121947	20210611-09_2021021-OK160A_816_5.3.236.mgf	0	0	0	0	0	175	194			PEAKS DB
K.YKPESSDLETATIK.K	N	126.50	1580.7933	14	3.8	791.4036	2	25.19	2	F2:75149	20210611-09_2021021-OK160A_816_5.3.236.mgf	0	0	0	0	0	325	338			PEAKS DB
R.ENQLPLVVEFTQESAQK.I	N	122.23	1958.9949	17	-2.6	654.0012	3	51.77	2	F2:121715	20210611-09_2021021-OK160A_816_5.3.236.mgf	0	0	0	0	0	231	247			PEAKS DB
K.AATALEEQNLNVK.L	N	120.90	1399.7307	13	4.4	700.8728	2	27.82	2	F2:79371	20210611-09_2021021-OK160A_816_5.3.236.mgf	0	0	0	0	0	66	78			PEAKS DB
K.LKPHLM(+15.99)SEDVPDDWDAKPVK.I	N	120.27	2335.1519	20	3.0	779.3903	3	29.56	2	F2:82514	20210611-09_2021021-OK160A_816_5.3.236.mgf	0	0	0	0	0	349	368		M6:Oxidation (M):1000.00	PEAKS DB
R.NDFEGEFEEEAIVK.H	N	117.74	1654.7362	14	1.2	828.3729	2	42.82	2	F2:106304	20210611-09_2021021-OK160A_816_5.3.236.mgf	0	0	0	0	0	214	227			PEAKS DB
K.DSEEAVDYNGER.T	N	116.33	1382.5586	12	-6.2	692.2794	2	19.77	2	F2:65987	20210611-09_2021021-OK160A_816_5.3.236.mgf	0	0	0	0	0	447	458			PEAKS DB
K.MDATANEIEEVK.V	N	113.62	1348.6180	12	2.7	675.3153	2	28.09	2	F2:79934	20210611-09_2021021-OK160A_816_5.3.236.mgf		0	0	0	0	423	434			PEAKS DB
R.ITEFFGLKDDEIPAVR.L	N	113.32	1848.9620	16	-0.6	617.3253	3	47.36	1	F1:45510	20210611-08_2021021-OK192A_815_5.3.236.mgf	0	0	0	0	0	299	314			PEAKS DB
K.KAAASIDDIPFAITSEDHVFK.E	N	110.26	2274.1531	21	0.5	759.0555	3	47.20	2	F2:113862	20210611-09_2021021-OK160A_816_5.3.236.mgf		0	0	0	0	174	194			PEAKS DB
K.LKPHLMSEDVPDDWDAKPVK.I	N	106.07	2319.1567	20	7.8	774.0623	3	33.33	2	F2:89240	20210611-09_2021021-OK160A_816_5.3.236.mgf	0	0	0	0	0	349	368			PEAKS DB
K.M(+15.99)DATANEIEEVK.V	N	99.38	1364.6129	12	-1.0	683.3102	2	25.80	2	F2:75953	20210611-09_2021021-OK160A_816_5.3.236.mgf	0	0	0	0	0	423	434	Oxidation (M)	M1:Oxidation (M):1000.00	PEAKS DB
R.LIQLAEDMSK.Y	N	97.87	1146.5955	10	2.2	574.3038	2	34.62	2	F2:91986	20210611-09_2021021-OK160A_816_5.3.236.mgf	0	0	0	0	0	315	324			PEAKS DB
K.KFVQDFLDGK.L	N	95.51	1195.6237	10	2.0	598.8178	2	34.51	2	F2:91583	20210611-09_2021021-OK160A_816_5.3.236.mgf		0	0	0	0	339	348			PEAKS DB
K.Q(-17.03)LAPIWDELGEK.Y	N	95.23	1380.6925	12	-1.5	691.3496	2	59.13	2	F2:131431	20210611-09_2021021-OK160A_816_5.3.236.mgf	0	0	0	0	0	400	411		Q1:Pyro-glu from Q:1000.00	PEAKS DB
R.LIQLAEDM(+15.99)SK.Y	N	94.94	1162.5903	10	1.4	582.3008	2	26.69	2	F2:77509	20210611-09_2021021-OK160A_816_5.3.236.mgf	0	0	0	0	0	315	324		M8:Oxidation (M):1000.00	PEAKS DB
K.DQESTGALAFK.K	N	94.65	1165.5615	11	4.3	583.7881	2	28.45	2	F2:80728	20210611-09_2021021-OK160A_816_5.3.236.mgf	0	0	0	0	0	163	173			PEAKS DB
K.REEDVYVLTTK.N	N	89.94	1351.6984	11	1.1	676.8544	2	26.32	2	F2:77138	20210611-09_2021021-OK160A_816_5.3.236.mgf	0	0	0	0	0	22	32			PEAKS DB
R.EEDVYVLTTK.N	N	89.68	1195.5972	10	1.5	598.8045	2	31.54	1	F1:27383	20210611-08_2021021-OK192A_815_5.3.236.mgf	0	0	0	0	0	23	32			PEAKS DB
K.VDATVEDSLAAK.F	Y	88.57	1217.6139	12	1.7	609.8129	2	26.56	1	F1:20640	20210611-08_2021021-OK192A_815_5.3.236.mgf	0		0	0	0	82	93			PEAKS DB
K.MDKDGVVLLK.K	N	87.62	1116.6212	10	1.6	559.3164	2	27.37	2	F2:78640	20210611-09_2021021-OK160A_816_5.3.236.mgf		0	0	0	0	198	207			PEAKS DB
K.NHILLFLK.K	N	86.51	996.6120	8	3.6	499.3132	2	39.74	1	F1:37317	20210611-08_2021021-OK192A_815_5.3.236.mgf	0	0	0	0	0	255	262			PEAKS DB
R.E(-18.01)NQLPLVVEFTQESAQK.I	N	86.27	1940.9843	17	-6.1	971.4894	2	58.57	2	F2:130865	20210611-09_2021021-OK160A_816_5.3.236.mgf		0	0	0	0	231	247		E1:Pyro-glu from E:1000.00	PEAKS DB
K.QLAPIWDELGEK.Y	N	82.76	1397.7190	12	0.5	699.8645	2	48.72	1	F1:46550	20210611-08_2021021-OK192A_815_5.3.236.mgf	0	0	0	0	0	400	411			PEAKS DB
K.M(+15.99)DKDGVVLLK.K	N	80.81	1132.6162	10	0.4	567.3132	2	24.19	2	F2:73149	20210611-09_2021021-OK160A_816_5.3.236.mgf		0	0	0	0	198	207	Oxidation (M)	M1:Oxidation (M):1000.00	PEAKS DB
K.FVQDFLDGK.L	N	79.93	1067.5287	9	1.0	534.7699	2	37.97	2	F2:97970	20210611-09_2021021-OK160A_816_5.3.236.mgf	0	0	0	0	0	340	348			PEAKS DB
K.NHILLFLKK.E	N	79.31	1124.7069	9	5.4	375.9102	3	30.94	1	F1:26438	20210611-08_2021021-OK192A_815_5.3.236.mgf	0	0	0	0	0	255	263			PEAKS DB
K.VQSFPTLK.Y	N	75.55	918.5175	8	3.7	460.2658	2	29.78	2	F2:82955	20210611-09_2021021-OK160A_816_5.3.236.mgf	0	0	0	0	0	435	442			PEAKS DB
K.FLESGGTEGAGVPEDEEEEEEDEEGDDEDLPR.D	N	73.31	3508.3921	32	13.2	1170.4823	3	38.27	1	F1:35747	20210611-08_2021021-OK192A_815_5.3.236.mgf	0		0	0	0	466	497			PEAKS DB
K.SPADYTGGR.Q	Y	73.22	922.4144	9	0.9	462.2132	2	14.63	1	F1:5853	20210611-08_2021021-OK192A_815_5.3.236.mgf	0		0	0	0	112	120			PEAKS DB
K.DDEIPAVR.L	N	71.69	913.4504	8	0.8	457.7310	2	24.89	2	F2:74398	20210611-09_2021021-OK160A_816_5.3.236.mgf	0	0	0	0	0	307	314			PEAKS DB
R.E(-18.01)EDVYVLTTK.N	N	69.76	1177.5867	10	4.8	589.8010	2	31.52	2	F2:85902	20210611-09_2021021-OK160A_816_5.3.236.mgf		0	0	0	0	23	32		E1:Pyro-glu from E:1000.00	PEAKS DB
R.ITEFFGLK.D	N	66.91	953.5222	8	0.9	477.7668	2	42.32	2	F2:105336	20210611-09_2021021-OK160A_816_5.3.236.mgf	0	0	0	0	0	299	306			PEAKS DB
R.QSDDIINWLK.K	Y	64.68	1230.6244	10	-0.3	616.3170	2	48.47	1	F1:46302	20210611-08_2021021-OK192A_815_5.3.236.mgf	0		0	0	0	121	130			PEAKS DB
R.TLDAFVK.F	N	64.57	792.4381	7	2.8	397.2258	2	28.98	2	F2:81609	20210611-09_2021021-OK160A_816_5.3.236.mgf	0	0	0	0	0	459	465			PEAKS DB

total 39 peptides

Peptide	Uniq	-10lgP	Mass	Length	ppm	m/z	z	RT	Fraction	Scan	Source File	Area ebraeus	Area judaeus	#Feature	#Feature ebraeus	#Feature judaeus	Start	End	PTM	AScore	Found By
K.ALAPEYAK.A	N	62.34	861.4596	8	3.5	431.7368	2	19.50	2	F2:65275	20210611-09_2021021-OK160A_816_5.3.236.mgf	0	0	0	0	0	58	65			PEAKS DB
K.DGVVLLK.K	N	61.62	742.4589	7	1.0	372.2355	2	30.45	2	F2:84009	20210611-09_2021021-OK160A_816_5.3.236.mgf	0	0	0	0	0	201	207			PEAKS DB
K.EGGEDTIEK.F	N	59.47	976.4349	9	3.4	489.2243	2	12.93	2	F2:56083	20210611-09_2021021-OK160A_816_5.3.236.mgf		0	0	0	0	264	272			PEAKS DB
K.IFGGEVK.N	N	59.09	748.4119	7	4.2	375.2134	2	21.69	1	F1:13485	20210611-08_2021021-OK192A_815_5.3.236.mgf	0	0	0	0	0	248	254			PEAKS DB
**total 39 peptides**																					

V_1278_121_Protein

back to list

| Protein Coverage | Supporting Peptides |

Protein Coverage:

```
  1  MKFSSCLVLT LLVFVSAEDV KQEEGVHVLT TKNFDSFIAD NEFVLVEFYA PWCGHCKALA PEYAKAATTL EEEKLNVKLG
 81  KVDATVEESL ASKFEVRGYP TIKFFRKEKP DGPADYNGGR QAADIVNWLK KKTGPPAKEL KEKDEVKSFV EKDEVVVIGF
161  FKDQESTGAL AFKKAAAGID DIPFAITSED HVFKEYKMDK DGIVLLKKFD EGRNDFEGEF EEEAIVKHVR ENQLPLVVEF
241  TQESAQKIFG GEVKNHILLF LKKEGGEDTI EKFRGAAGDF KGKVLFIYLD TDNEENGRIT EFFGLKDDEI PAVRLIQLAE
321  DMSKYKPESL DLETATIKKF VQDFLDGKLK PHLMSEDVPD DWDAKPVKVL VGKNFKEVAM DKSKAVFVEF YAPWCGHCKQ
                                    423
401  LAPIWDELGE KYKDSKDIVV AKMDATANEI EEVKVQSFPT LKYFPKDSEE AVDYNGERTL DAFIKFLESG GTEGAGVPED
481  EEEEEEDEGD DEDLPRDEL
```

O Oxidation (M) (+15.99)

Supporting Peptides:

Peptide	Uniq	-10lgP	Mass	Length	ppm	m/z	z	RT	Fraction	Scan	Source File	Area ebraeus	Area judaeus	#Feature	#Feature ebraeus	#Feature judaeus	Start	End	PTM	AScore	Found By
R.ENQLPLVVEFTQESAQK.I	N	122.23	1958.9949	17	-2.6	654.0012	3	51.77	2	F2:121715	20210611-09_2021021-OK160A_816_5.3.236.mgf	0	0	0	0	0	231	247			PEAKS DB
K.LKPHLM(+15.99)SEDVPDDWDAKPVK.V	N	120.27	2335.1519	20	3.0	779.3903	3	29.56	2	F2:82514	20210611-09_2021021-OK160A_816_5.3.236.mgf	0	0	0	0	0	349	368		M6:Oxidation (M):1000.00	PEAKS DB
K.AAAGIDDIPFAITSEDHVFK.E	N	119.10	2116.0476	20	-1.9	706.3525	3	51.54	1	F1:48186	20210611-08_2021021-OK192A_815_5.3.236.mgf	0		0	0	0	175	194			PEAKS DB
R.NDFEGEFEEEAIVK.H	N	117.74	1654.7362	14	1.2	828.3729	2	42.82	2	F2:106304	20210611-09_2021021-OK160A_816_5.3.236.mgf	0	0	0	0	0	214	227			PEAKS DB
K.DSEEAVDYNGER.T	N	116.33	1382.5586	12	-6.2	692.2794	2	19.77	2	F2:65987	20210611-09_2021021-OK160A_816_5.3.236.mgf	0	0	0	0	0	447	458			PEAKS DB
K.MDATANEIEEVK.V	N	113.62	1348.6180	12	2.7	675.3153	2	28.09	2	F2:79934	20210611-09_2021021-OK160A_816_5.3.236.mgf		0	0	0	0	423	434			PEAKS DB
R.ITEFFGLKDDEIPAVR.L	N	113.32	1848.9620	16	-0.6	617.3253	3	47.36	1	F1:45510	20210611-08_2021021-OK192A_815_5.3.236.mgf	0	0	0	0	0	299	314			PEAKS DB
K.LKPHLMSEDVPDDWDAKPVK.V	N	106.07	2319.1567	20	7.8	774.0623	3	33.33	2	F2:89240	20210611-09_2021021-OK160A_816_5.3.236.mgf	0	0	0	0	0	349	368			PEAKS DB
K.VDATVEESLASK.F	N	104.50	1247.6245	12	3.3	624.8192	2	26.76	1	F1:20877	20210611-08_2021021-OK192A_815_5.3.236.mgf	0		0	0	0	82	93			PEAKS DB
K.M(+15.99)DATANEIEEVK.V	N	99.38	1364.6129	12	-1.0	683.3102	2	25.80	2	F2:75953	20210611-09_2021021-OK160A_816_5.3.236.mgf	0	0	0	0	0	423	434	Oxidation (M)	M1:Oxidation (M):1000.00	PEAKS DB
R.LIQLAEDMSK.Y	N	97.87	1146.5955	10	2.2	574.3038	2	34.62	2	F2:91986	20210611-09_2021021-OK160A_816_5.3.236.mgf	0	0	0	0	0	315	324			PEAKS DB
K.KFVQDFLDGK.L	N	95.51	1195.6237	10	2.0	598.8178	2	34.51	2	F2:91583	20210611-09_2021021-OK160A_816_5.3.236.mgf		0	0	0	0	339	348			PEAKS DB
K.Q(-17.03)LAPIWDELGEK.Y	N	95.23	1380.6925	12	-1.5	691.3496	2	59.13	2	F2:131431	20210611-09_2021021-OK160A_816_5.3.236.mgf	0	0	0	0	0	400	411		Q1:Pyro-glu from Q:1000.00	PEAKS DB
R.LIQLAEDM(+15.99)SK.Y	N	94.94	1162.5903	10	1.4	582.3008	2	26.69	2	F2:77509	20210611-09_2021021-OK160A_816_5.3.236.mgf	0	0	0	0	0	315	324		M8:Oxidation (M):1000.00	PEAKS DB
K.DQESTGALAFK.K	N	94.65	1165.5615	11	4.3	583.7881	2	28.45	2	F2:80728	20210611-09_2021021-OK160A_816_5.3.236.mgf	0	0	0	0	0	163	173			PEAKS DB
K.YKPESLDLETATIK.K	N	92.19	1606.8453	14	-0.3	536.6202	3	34.14	1	F1:30794	20210611-08_2021021-OK192A_815_5.3.236.mgf	0		0	0	0	325	338			PEAKS DB
R.QAADIVNWLK.K	N	90.48	1156.6240	10	2.2	579.3184	2	45.94	1	F1:44192	20210611-08_2021021-OK192A_815_5.3.236.mgf	0		0	0	0	121	130			PEAKS DB
K.NHILLFLK.K	N	86.51	996.6120	8	3.6	499.3132	2	39.74	1	F1:37317	20210611-08_2021021-OK192A_815_5.3.236.mgf	0	0	0	0	0	255	262			PEAKS DB
R.E(-18.01)NQLPLVVEFTQESAQK.I	N	86.27	1940.9843	17	-6.1	971.4894	2	58.57	2	F2:130865	20210611-09_2021021-OK160A_816_5.3.236.mgf		0	0	0	0	231	247		E1:Pyro-glu from E:1000.00	PEAKS DB
K.QLAPIWDELGEK.Y	N	82.76	1397.7190	12	0.5	699.8645	2	48.72	1	F1:46550	20210611-08_2021021-OK192A_815_5.3.236.mgf	0	0	0	0	0	400	411			PEAKS DB
K.FVQDFLDGK.L	N	79.93	1067.5287	9	1.0	534.7699	2	37.97	2	F2:97970	20210611-09_2021021-OK160A_816_5.3.236.mgf	0	0	0	0	0	340	348			PEAKS DB
K.NHILLFLKK.E	N	79.31	1124.7069	9	5.4	375.9102	3	30.94	1	F1:26438	20210611-08_2021021-OK192A_815_5.3.236.mgf	0	0	0	0	0	255	263			PEAKS DB
K.VQSFPTLK.Y	N	75.55	918.5175	8	3.7	460.2658	2	29.78	2	F2:82955	20210611-09_2021021-OK160A_816_5.3.236.mgf	0	0	0	0	0	435	442			PEAKS DB
K.EKPDGPADYNGGR.Q	N	73.49	1374.6163	13	-1.1	688.3121	2	13.78	1	F1:5004	20210611-08_2021021-OK192A_815_5.3.236.mgf	0		0	0	0	108	120			PEAKS DB
K.DDEIPAVR.L	N	71.69	913.4504	8	0.8	457.7310	2	24.89	2	F2:74398	20210611-09_2021021-OK160A_816_5.3.236.mgf	0	0	0	0	0	307	314			PEAKS DB
R.ITEFFGLK.D	N	66.91	953.5222	8	0.9	477.7668	2	42.32	2	F2:105336	20210611-09_2021021-OK160A_816_5.3.236.mgf	0	0	0	0	0	299	306			PEAKS DB
K.ALAPEYAK.A	N	62.34	861.4596	8	3.5	431.7368	2	19.50	2	F2:65275	20210611-09_2021021-OK160A_816_5.3.236.mgf	0	0	0	0	0	58	65			PEAKS DB
R.TLDAFIK.F	N	61.58	806.4538	7	2.3	404.2336	2	33.58	1	F1:29977	20210611-08_2021021-OK192A_815_5.3.236.mgf	0		0	0	0	459	465			PEAKS DB
K.AATTLEEEKLNVK.L	N	59.94	1444.7773	13	-0.8	482.5975	3	24.76	1	F1:17934	20210611-08_2021021-OK192A_815_5.3.236.mgf	0		0	0	0	66	78			PEAKS DB
K.EGGEDTIEK.F	N	59.47	976.4349	9	3.4	489.2243	2	12.93	2	F2:56083	20210611-09_2021021-OK160A_816_5.3.236.mgf		0	0	0	0	264	272			PEAKS DB
K.IFGGEVK.N	N	59.09	748.4119	7	4.2	375.2134	2	21.69	1	F1:13485	20210611-08_2021021-OK192A_815_5.3.236.mgf	0	0	0	0	0	248	254			PEAKS DB
K.DGIVLLK.K	N	56.48	756.4745	7	0.5	379.2433	2	35.60	1	F1:32687	20210611-08_2021021-OK192A_815_5.3.236.mgf	0		0	0	0	201	207			PEAKS DB
K.QEEGVHVLTTK.N	Y	56.41	1239.6459	11	-1.2	620.8271	2	19.69	1	F1:11068	20210611-08_2021021-OK192A_815_5.3.236.mgf	0		0	0	0	22	32			PEAKS DB
**total 33 peptides**																					

V_1302_228_Protein

back to list

| Protein Coverage | Supporting Peptides |

Protein Coverage:

```
  1  MKFSSCLVLT LLVFVSAEDV KQEEGVHVLT TKNFDSFIAD NEFVLVEFYA PWCGHCKALA PEYAKAATTL EEEKLNVKLG
 81  KVDATVEESL ASKFEVRGYP TIKFFRKEKP DGPADYNGGR QAADIVNWLK KKTGPPAKEL KEKDEVKSFV EKDEVVVIGF
161  FKDQESTGAL AFKKAAAGID DIPFAITSED HVFKEYKMDK DGIVLLKKFD EGRNDFEGEF EEEAIVKHVR ENQLPLVVEF
241  TQESAQKIFG GEVKNHILLF LKKEGGEDTI EKFRGAAGDF KGKVLFIYLD TDNEENGRIT EFFGLKDDEI PAVRLIQLAE
321  DMSKYKPESL DLETATIKKF VQDFLDGKLK PHLMSEDVPD DWDAKPVKVL VGKNFKEVAM DKSKAVFVEF YAPWCGHCKQ
                                  423
401  LAPIWDELGE KYKDSKDIVV AKMDATANEI EEVKVQSFPT LKYFPKDSEE AVDYNGERTL DAFIKFLESG GTEGAGVPED
481  EEEEEEDEGD DEDLPRDEL
```

O Oxidation (M) (+15.99)

Supporting Peptides:

Peptide	Uniq	-10lgP	Mass	Length	ppm	m/z	z	RT	Fraction	Scan	Source File	Area ebraeus	Area judaeus	#Feature	#Feature ebraeus	#Feature judaeus	Start	End	PTM	AScore	Found By
R.ENQLPLVVEFTQESAQK.I	N	122.23	1958.9949	17	-2.6	654.0012	3	51.77	2	F2:121715	20210611-09_2021021-OK160A_816_5.3.236.mgf	0	0	0	0	0	231	247			PEAKS DB
K.LKPHLM(+15.99)SEDVPDDWDAKPVK.V	N	120.27	2335.1519	20	3.0	779.3903	3	29.56	2	F2:82514	20210611-09_2021021-OK160A_816_5.3.236.mgf	0	0	0	0	0	349	368		M6:Oxidation (M):1000.00	PEAKS DB
K.AAAGIDDIPFAITSEDHVFK.E	N	119.10	2116.0476	20	-1.9	706.3525	3	51.54	1	F1:48186	20210611-08_2021021-OK192A_815_5.3.236.mgf	0		0	0	0	175	194			PEAKS DB
R.NDFEGEFEEEAIVK.H	N	117.74	1654.7362	14	1.2	828.3729	2	42.82	2	F2:106304	20210611-09_2021021-OK160A_816_5.3.236.mgf	0	0	0	0	0	214	227			PEAKS DB
K.DSEEAVDYNGER.T	N	116.33	1382.5586	12	-6.2	692.2794	2	19.77	2	F2:65987	20210611-09_2021021-OK160A_816_5.3.236.mgf	0	0	0	0	0	447	458			PEAKS DB
K.MDATANEIEEVK.V	N	113.62	1348.6180	12	2.7	675.3153	2	28.09	2	F2:79934	20210611-09_2021021-OK160A_816_5.3.236.mgf		0	0	0	0	423	434			PEAKS DB
R.ITEFFGLKDDEIPAVR.L	N	113.32	1848.9620	16	-0.6	617.3253	3	47.36	1	F1:45510	20210611-08_2021021-OK192A_815_5.3.236.mgf	0	0	0	0	0	299	314			PEAKS DB
K.LKPHLMSEDVPDDWDAKPVK.V	N	106.07	2319.1567	20	7.8	774.0623	3	33.33	2	F2:89240	20210611-09_2021021-OK160A_816_5.3.236.mgf	0	0	0	0	0	349	368			PEAKS DB
K.VDATVEESLASK.F	N	104.50	1247.6245	12	3.3	624.8192	2	26.76	1	F1:20877	20210611-08_2021021-OK192A_815_5.3.236.mgf	0		0	0	0	82	93			PEAKS DB
K.M(+15.99)DATANEIEEVK.V	N	99.38	1364.6129	12	-1.0	683.3102	2	25.80	2	F2:75953	20210611-09_2021021-OK160A_816_5.3.236.mgf	0	0	0	0	0	423	434	Oxidation (M)	M1:Oxidation (M):1000.00	PEAKS DB
R.LIQLAEDMSK.Y	N	97.87	1146.5955	10	2.2	574.3038	2	34.62	2	F2:91986	20210611-09_2021021-OK160A_816_5.3.236.mgf	0	0	0	0	0	315	324			PEAKS DB
K.KFVQDFLDGK.L	N	95.51	1195.6237	10	2.0	598.8178	2	34.51	2	F2:91583	20210611-09_2021021-OK160A_816_5.3.236.mgf		0	0	0	0	339	348			PEAKS DB
K.Q(-17.03)LAPIWDELGEK.Y	N	95.23	1380.6925	12	-1.5	691.3496	2	59.13	2	F2:131431	20210611-09_2021021-OK160A_816_5.3.236.mgf	0	0	0	0	0	400	411		Q1:Pyro-glu from Q:1000.00	PEAKS DB
R.LIQLAEDM(+15.99)SK.Y	N	94.94	1162.5903	10	1.4	582.3008	2	26.69	2	F2:77509	20210611-09_2021021-OK160A_816_5.3.236.mgf	0	0	0	0	0	315	324		M8:Oxidation (M):1000.00	PEAKS DB
K.DQESTGALAFK.K	N	94.65	1165.5615	11	4.3	583.7881	2	28.45	2	F2:80728	20210611-09_2021021-OK160A_816_5.3.236.mgf	0	0	0	0	0	163	173			PEAKS DB
K.YKPESLDLETATIK.K	N	92.19	1606.8453	14	-0.3	536.6202	3	34.14	1	F1:30794	20210611-08_2021021-OK192A_815_5.3.236.mgf	0		0	0	0	325	338			PEAKS DB
R.QAADIVNWLK.K	N	90.48	1156.6240	10	2.2	579.3184	2	45.94	1	F1:44192	20210611-08_2021021-OK192A_815_5.3.236.mgf	0		0	0	0	121	130			PEAKS DB
K.NHILLFLK.K	N	86.51	996.6120	8	3.6	499.3132	2	39.74	1	F1:37317	20210611-08_2021021-OK192A_815_5.3.236.mgf	0	0	0	0	0	255	262			PEAKS DB
R.E(-18.01)NQLPLVVEFTQESAQK.I	N	86.27	1940.9843	17	-6.1	971.4894	2	58.57	2	F2:130865	20210611-09_2021021-OK160A_816_5.3.236.mgf		0	0	0	0	231	247		E1:Pyro-glu from E:1000.00	PEAKS DB
K.QLAPIWDELGEK.Y	N	82.76	1397.7190	12	0.5	699.8645	2	48.72	1	F1:46550	20210611-08_2021021-OK192A_815_5.3.236.mgf	0	0	0	0	0	400	411			PEAKS DB
K.FVQDFLDGK.L	N	79.93	1067.5287	9	1.0	534.7699	2	37.97	2	F2:97970	20210611-09_2021021-OK160A_816_5.3.236.mgf	0	0	0	0	0	340	348			PEAKS DB
K.NHILLFLKK.E	N	79.31	1124.7069	9	5.4	375.9102	3	30.94	1	F1:26438	20210611-08_2021021-OK192A_815_5.3.236.mgf	0	0	0	0	0	255	263			PEAKS DB
K.VQSFPTLK.Y	N	75.55	918.5175	8	3.7	460.2658	2	29.78	2	F2:82955	20210611-09_2021021-OK160A_816_5.3.236.mgf	0	0	0	0	0	435	442			PEAKS DB
K.EKPDGPADYNGGR.Q	N	73.49	1374.6163	13	-1.1	688.3121	2	13.78	1	F1:5004	20210611-08_2021021-OK192A_815_5.3.236.mgf	0		0	0	0	108	120			PEAKS DB
K.DDEIPAVR.L	N	71.69	913.4504	8	0.8	457.7310	2	24.89	2	F2:74398	20210611-09_2021021-OK160A_816_5.3.236.mgf	0	0	0	0	0	307	314			PEAKS DB
R.ITEFFGLK.D	N	66.91	953.5222	8	0.9	477.7668	2	42.32	2	F2:105336	20210611-09_2021021-OK160A_816_5.3.236.mgf	0	0	0	0	0	299	306			PEAKS DB
K.ALAPEYAK.A	N	62.34	861.4596	8	3.5	431.7368	2	19.50	2	F2:65275	20210611-09_2021021-OK160A_816_5.3.236.mgf	0	0	0	0	0	58	65			PEAKS DB
R.TLDAFIK.F	N	61.58	806.4538	7	2.3	404.2336	2	33.58	1	F1:29977	20210611-08_2021021-OK192A_815_5.3.236.mgf	0		0	0	0	459	465			PEAKS DB
K.AATTLEEEKLNVK.L	N	59.94	1444.7773	13	-0.8	482.5975	3	24.76	1	F1:17934	20210611-08_2021021-OK192A_815_5.3.236.mgf	0		0	0	0	66	78			PEAKS DB
K.EGGEDTIEK.F	N	59.47	976.4349	9	3.4	489.2243	2	12.93	2	F2:56083	20210611-09_2021021-OK160A_816_5.3.236.mgf		0	0	0	0	264	272			PEAKS DB
K.IFGGEVK.N	N	59.09	748.4119	7	4.2	375.2134	2	21.69	1	F1:13485	20210611-08_2021021-OK192A_815_5.3.236.mgf	0	0	0	0	0	248	254			PEAKS DB
K.DGIVLLK.K	N	56.48	756.4745	7	0.5	379.2433	2	35.60	1	F1:32687	20210611-08_2021021-OK192A_815_5.3.236.mgf	0		0	0	0	201	207			PEAKS DB
K.QEEGVHVLTTK.N	Y	56.41	1239.6459	11	-1.2	620.8271	2	19.69	1	F1:11068	20210611-08_2021021-OK192A_815_5.3.236.mgf	0		0	0	0	22	32			PEAKS DB
**total 33 peptides**																					

V_1258_220_Protein

back to list

| Protein Coverage | Supporting Peptides |

Protein Coverage:

```
  1  MKFSSCLVLT LLVFVSAEDV KQEEGVHVLT TKNFDSFIAD NEFVLVEFYA PWCGHCKALA PEYAKAATTL EEEKLNVKLG
 81  KVDATVEESL ASKFEVRGYP TIKFFRKEKP DGPADYNGGR QAADIVNWLK KKTGPPAKEL KEKDEVKSFV EKDEVVVIGF
161  FKDQESTGAL AFKKAAAGID DIPFAITSED HVFKEYKMDK DGIVLLKKFD EGRNDFEGEF EEEAIVKHVR ENQLPLVVEF
241  TQESAQKIFG GEVKNHILLF LKKEGGEDTI EKFRGAAGDF KGKVLFIYLD TDNEENGRIT EFFGLKDDEI PAVRLIQLAE
321  DMSKYKPESL DLETATIKKF VQDFLDGKLK PHLMSEDVPD DWDAKPVKVL VGKNFKEVAM DKSKAVFVEF YAPWCGHCKQ
                                  423
401  LAPIWDELGE KYKDSKDIVV AKMDATANEI EEVKVQSFPT LKYFPKDSEE AVDYNGERTL DAFIKFLESG GTEGAGVPED
481  EEEEEEDEGD DEDLPRDEL
```

O Oxidation (M) (+15.99)

Supporting Peptides:

Peptide	Uniq	-10lgP	Mass	Length	ppm	m/z	z	RT	Fraction	Scan	Source File	Area ebraeus	Area judaeus	#Feature	#Feature ebraeus	#Feature judaeus	Start	End	PTM	AScore	Found By
R.ENQLPLVVEFTQESAQK.I	N	122.23	1958.9949	17	-2.6	654.0012	3	51.77	2	F2:121715	20210611-09_2021021-OK160A_816_5.3.236.mgf	0	0	0	0	0	231	247			PEAKS DB
**total 33 peptides**																					

Peptide	Uniq	-10lgP	Mass	Length	ppm	m/z	z	RT	Fraction	Scan	Source File	Area ebraeus	Area judaeus	#Feature	#Feature ebraeus	#Feature judaeus	Start	End	PTM	AScore	Found By
K.LKPHLM(+15.99)SEDVPDDWDAKPVK.V	N	120.27	2335.1519	20	3.0	779.3903	3	29.56	2	F2:82514	20210611-09_2021021-OK160A_816_5.3.236.mgf	0	0	0	0	0	349	368		M6:Oxidation (M):1000.00	PEAKS DB
K.AAAGIDDIPFAITSEDHVFK.E	N	119.10	2116.0476	20	-1.9	706.3525	3	51.54	1	F1:48186	20210611-08_2021021-OK192A_815_5.3.236.mgf	0		0	0	0	175	194			PEAKS DB
R.NDFEGEFEEEAIVK.H	N	117.74	1654.7362	14	1.2	828.3729	2	42.82	2	F2:106304	20210611-09_2021021-OK160A_816_5.3.236.mgf	0	0	0	0	0	214	227			PEAKS DB
K.DSEEAVDYNGER.T	N	116.33	1382.5586	12	-6.2	692.2794	2	19.77	2	F2:65987	20210611-09_2021021-OK160A_816_5.3.236.mgf	0	0	0	0	0	447	458			PEAKS DB
K.MDATANEIEEVK.V	N	113.62	1348.6180	12	2.7	675.3153	2	28.09	2	F2:79934	20210611-09_2021021-OK160A_816_5.3.236.mgf		0	0	0	0	423	434			PEAKS DB
R.ITEFFGLKDDEIPAVR.L	N	113.32	1848.9620	16	-0.6	617.3253	3	47.36	1	F1:45510	20210611-08_2021021-OK192A_815_5.3.236.mgf	0	0	0	0	0	299	314			PEAKS DB
K.LKPHLMSEDVPDDWDAKPVK.V	N	106.07	2319.1567	20	7.8	774.0623	3	33.33	2	F2:89240	20210611-09_2021021-OK160A_816_5.3.236.mgf	0	0	0	0	0	349	368			PEAKS DB
K.VDATVEESLASK.F	N	104.50	1247.6245	12	3.3	624.8192	2	26.76	1	F1:20877	20210611-08_2021021-OK192A_815_5.3.236.mgf	0		0	0	0	82	93			PEAKS DB
K.M(+15.99)DATANEIEEVK.V	N	99.38	1364.6129	12	-1.0	683.3102	2	25.80	2	F2:75953	20210611-09_2021021-OK160A_816_5.3.236.mgf	0	0	0	0	0	423	434	Oxidation (M)	M1:Oxidation (M):1000.00	PEAKS DB
R.LIQLAEDMSK.Y	N	97.87	1146.5955	10	2.2	574.3038	2	34.62	2	F2:91986	20210611-09_2021021-OK160A_816_5.3.236.mgf	0	0	0	0	0	315	324			PEAKS DB
K.KFVQDFLDGK.L	N	95.51	1195.6237	10	2.0	598.8178	2	34.51	2	F2:91583	20210611-09_2021021-OK160A_816_5.3.236.mgf		0	0	0	0	339	348			PEAKS DB
K.Q(-17.03)LAPIWDELGEK.Y	N	95.23	1380.6925	12	-1.5	691.3496	2	59.13	2	F2:131431	20210611-09_2021021-OK160A_816_5.3.236.mgf	0	0	0	0	0	400	411		Q1:Pyro-glu from Q:1000.00	PEAKS DB
R.LIQLAEDM(+15.99)SK.Y	N	94.94	1162.5903	10	1.4	582.3008	2	26.69	2	F2:77509	20210611-09_2021021-OK160A_816_5.3.236.mgf	0	0	0	0	0	315	324		M8:Oxidation (M):1000.00	PEAKS DB
K.DQESTGALAFK.K	N	94.65	1165.5615	11	4.3	583.7881	2	28.45	2	F2:80728	20210611-09_2021021-OK160A_816_5.3.236.mgf	0	0	0	0	0	163	173			PEAKS DB
K.YKPESLDLETATIK.K	N	92.19	1606.8453	14	-0.3	536.6202	3	34.14	1	F1:30794	20210611-08_2021021-OK192A_815_5.3.236.mgf	0		0	0	0	325	338			PEAKS DB
R.QAADIVNWLK.K	N	90.48	1156.6240	10	2.2	579.3184	2	45.94	1	F1:44192	20210611-08_2021021-OK192A_815_5.3.236.mgf	0		0	0	0	121	130			PEAKS DB
K.NHILLFLK.K	N	86.51	996.6120	8	3.6	499.3132	2	39.74	1	F1:37317	20210611-08_2021021-OK192A_815_5.3.236.mgf	0	0	0	0	0	255	262			PEAKS DB
R.E(-18.01)NQLPLVVEFTQESAQK.I	N	86.27	1940.9843	17	-6.1	971.4894	2	58.57	2	F2:130865	20210611-09_2021021-OK160A_816_5.3.236.mgf		0	0	0	0	231	247		E1:Pyro-glu from E:1000.00	PEAKS DB
K.QLAPIWDELGEK.Y	N	82.76	1397.7190	12	0.5	699.8645	2	48.72	1	F1:46550	20210611-08_2021021-OK192A_815_5.3.236.mgf	0	0	0	0	0	400	411			PEAKS DB
K.FVQDFLDGK.L	N	79.93	1067.5287	9	1.0	534.7699	2	37.97	2	F2:97970	20210611-09_2021021-OK160A_816_5.3.236.mgf	0	0	0	0	0	340	348			PEAKS DB
K.NHILLFLKK.E	N	79.31	1124.7069	9	5.4	375.9102	3	30.94	1	F1:26438	20210611-08_2021021-OK192A_815_5.3.236.mgf	0	0	0	0	0	255	263			PEAKS DB
K.VQSFPTLK.Y	N	75.55	918.5175	8	3.7	460.2658	2	29.78	2	F2:82955	20210611-09_2021021-OK160A_816_5.3.236.mgf	0	0	0	0	0	435	442			PEAKS DB
K.EKPDGPADYNGGR.Q	N	73.49	1374.6163	13	-1.1	688.3121	2	13.78	1	F1:5004	20210611-08_2021021-OK192A_815_5.3.236.mgf	0		0	0	0	108	120			PEAKS DB
K.DDEIPAVR.L	N	71.69	913.4504	8	0.8	457.7310	2	24.89	2	F2:74398	20210611-09_2021021-OK160A_816_5.3.236.mgf	0	0	0	0	0	307	314			PEAKS DB
R.ITEFFGLK.D	N	66.91	953.5222	8	0.9	477.7668	2	42.32	2	F2:105336	20210611-09_2021021-OK160A_816_5.3.236.mgf	0	0	0	0	0	299	306			PEAKS DB
K.ALAPEYAK.A	N	62.34	861.4596	8	3.5	431.7368	2	19.50	2	F2:65275	20210611-09_2021021-OK160A_816_5.3.236.mgf	0	0	0	0	0	58	65			PEAKS DB
R.TLDAFIK.F	N	61.58	806.4538	7	2.3	404.2336	2	33.58	1	F1:29977	20210611-08_2021021-OK192A_815_5.3.236.mgf	0		0	0	0	459	465			PEAKS DB
K.AATTLEEEKLNVK.L	N	59.94	1444.7773	13	-0.8	482.5975	3	24.76	1	F1:17934	20210611-08_2021021-OK192A_815_5.3.236.mgf	0		0	0	0	66	78			PEAKS DB
K.EGGEDTIEK.F	N	59.47	976.4349	9	3.4	489.2243	2	12.93	2	F2:56083	20210611-09_2021021-OK160A_816_5.3.236.mgf		0	0	0	0	264	272			PEAKS DB
K.IFGGEVK.N	N	59.09	748.4119	7	4.2	375.2134	2	21.69	1	F1:13485	20210611-08_2021021-OK192A_815_5.3.236.mgf	0	0	0	0	0	248	254			PEAKS DB
K.DGIVLLK.K	N	56.48	756.4745	7	0.5	379.2433	2	35.60	1	F1:32687	20210611-08_2021021-OK192A_815_5.3.236.mgf	0		0	0	0	201	207			PEAKS DB
K.QEEGVHVLTTK.N	Y	56.41	1239.6459	11	-1.2	620.8271	2	19.69	1	F1:11068	20210611-08_2021021-OK192A_815_5.3.236.mgf	0		0	0	0	22	32			PEAKS DB
**total 33 peptides**																					

V_CG13_261_Protein

back to list

| Protein Coverage | Supporting Peptides |

Protein Coverage:

Oxidation (M) (+15.99)

1 MKFSSCLVLT LLVFVSAEDV K**QEEGVHVLT TK**NFDSFIAD NEFVLVEFYA PWCGHCK**ALA PEYAKAATTL EEEKLNVK**LG

81 K**VDATVEESL ASK**FEVRGYP TIKFFRK**EKP DGPADYNGGR QAADIVNWLK** KKTGPPAKEL KEKDEVKSFV EKDEVVVIGF

161 FK**DQESTGAL AFK**K**AAAGID DIPFAITSED HVFK**EYKMDK **DGIVLLK**KFD EGR**NDFEGEF EEEAIVK**HVR **ENQLPLVVEF**

241 **TQESAQKIFG GEVKNHILLF LKKEGGEDTI EK**FRGAAGDF KGKVLFIYLD TDNEENGR**IT EFFGLKDDEI PAVRLIQLAE**

321 **DMSKYKPESL DLETATIKKF VQDFLDGKLK PHLMSEDVPD DWDAKPVK**VL VGKNFKEVAM DKSKAVFVEF YAPWCGHCK**Q**

423

401 **LAPIWDELGE K**YKDSKDIVV AK**MDATANEI EEVKVQSFPT LK**YFPK**DSEE AVDYNGERTL DAFIK**FLESG GTEGAGVPED

481 EEEEEEDEGD DEDLPRDEL

Supporting Peptides:

Peptide	Uniq	-10lgP	Mass	Length	ppm	m/z	z	RT	Fraction	Scan	Source File	Area ebraeus	Area judaeus	#Feature	#Feature ebraeus	#Feature judaeus	Start	End	PTM	AScore	Found By
R.ENQLPLVVEFTQESAQK.I	N	122.23	1958.9949	17	-2.6	654.0012	3	51.77	2	F2:121715	20210611-09_2021021-OK160A_816_5.3.236.mgf	0	0	0	0	0	231	247			PEAKS DB
K.LKPHLM(+15.99)SEDVPDDWDAKPVK.V	N	120.27	2335.1519	20	3.0	779.3903	3	29.56	2	F2:82514	20210611-09_2021021-OK160A_816_5.3.236.mgf	0	0	0	0	0	349	368		M6:Oxidation (M):1000.00	PEAKS DB
K.AAAGIDDIPFAITSEDHVFK.E	N	119.10	2116.0476	20	-1.9	706.3525	3	51.54	1	F1:48186	20210611-08_2021021-OK192A_815_5.3.236.mgf	0		0	0	0	175	194			PEAKS DB
R.NDFEGEFEEEAIVK.H	N	117.74	1654.7362	14	1.2	828.3729	2	42.82	2	F2:106304	20210611-09_2021021-OK160A_816_5.3.236.mgf	0	0	0	0	0	214	227			PEAKS DB
K.DSEEAVDYNGER.T	N	116.33	1382.5586	12	-6.2	692.2794	2	19.77	2	F2:65987	20210611-09_2021021-OK160A_816_5.3.236.mgf	0	0	0	0	0	447	458			PEAKS DB
K.MDATANEIEEVK.V	N	113.62	1348.6180	12	2.7	675.3153	2	28.09	2	F2:79934	20210611-09_2021021-OK160A_816_5.3.236.mgf		0	0	0	0	423	434			PEAKS DB
R.ITEFFGLKDDEIPAVR.L	N	113.32	1848.9620	16	-0.6	617.3253	3	47.36	1	F1:45510	20210611-08_2021021-OK192A_815_5.3.236.mgf	0	0	0	0	0	299	314			PEAKS DB
K.LKPHLMSEDVPDDWDAKPVK.V	N	106.07	2319.1567	20	7.8	774.0623	3	33.33	2	F2:89240	20210611-09_2021021-OK160A_816_5.3.236.mgf	0	0	0	0	0	349	368			PEAKS DB
K.VDATVEESLASK.F	N	104.50	1247.6245	12	3.3	624.8192	2	26.76	1	F1:20877	20210611-08_2021021-OK192A_815_5.3.236.mgf	0		0	0	0	82	93			PEAKS DB
K.M(+15.99)DATANEIEEVK.V	N	99.38	1364.6129	12	-1.0	683.3102	2	25.80	2	F2:75953	20210611-09_2021021-OK160A_816_5.3.236.mgf	0	0	0	0	0	423	434	Oxidation (M)	M1:Oxidation (M):1000.00	PEAKS DB
R.LIQLAEDMSK.Y	N	97.87	1146.5955	10	2.2	574.3038	2	34.62	2	F2:91986	20210611-09_2021021-OK160A_816_5.3.236.mgf	0	0	0	0	0	315	324			PEAKS DB
K.KFVQDFLDGK.L	N	95.51	1195.6237	10	2.0	598.8178	2	34.51	2	F2:91583	20210611-09_2021021-OK160A_816_5.3.236.mgf		0	0	0	0	339	348			PEAKS DB
**total 33 peptides**																					

Peptide	Uniq	-10lgP	Mass	Length	ppm	m/z	z	RT	Fraction	Scan	Source File	Area ebraeus	Area judaeus	#Feature	#Feature ebraeus	#Feature judaeus	Start	End	PTM	AScore	Found By
K.Q(-17.03)LAPIWDELGEK.Y	N	95.23	1380.6925	12	-1.5	691.3496	2	59.13	2	F2:131431	20210611-09_2021021-OK160A_816_5.3.236.mgf	0	0	0	0	0	400	411		Q1:Pyro-glu from Q:1000.00	PEAKS DB
R.LIQLAEDM(+15.99)SK.Y	N	94.94	1162.5903	10	1.4	582.3008	2	26.69	2	F2:77509	20210611-09_2021021-OK160A_816_5.3.236.mgf	0	0	0	0	0	315	324		M8:Oxidation (M):1000.00	PEAKS DB
K.DQESTGALAFK.K	N	94.65	1165.5615	11	4.3	583.7881	2	28.45	2	F2:80728	20210611-09_2021021-OK160A_816_5.3.236.mgf	0	0	0	0	0	163	173			PEAKS DB
K.YKPESLDLETATIK.K	N	92.19	1606.8453	14	-0.3	536.6202	3	34.14	1	F1:30794	20210611-08_2021021-OK192A_815_5.3.236.mgf	0		0	0	0	325	338			PEAKS DB
R.QAADIVNWLK.K	N	90.48	1156.6240	10	2.2	579.3184	2	45.94	1	F1:44192	20210611-08_2021021-OK192A_815_5.3.236.mgf	0		0	0	0	121	130			PEAKS DB
K.NHILLFLK.K	N	86.51	996.6120	8	3.6	499.3132	2	39.74	1	F1:37317	20210611-08_2021021-OK192A_815_5.3.236.mgf	0	0	0	0	0	255	262			PEAKS DB
R.E(-18.01)NQLPLVVEFTQESAQK.I	N	86.27	1940.9843	17	-6.1	971.4894	2	58.57	2	F2:130865	20210611-09_2021021-OK160A_816_5.3.236.mgf		0	0	0	0	231	247		E1:Pyro-glu from E:1000.00	PEAKS DB
K.QLAPIWDELGEK.Y	N	82.76	1397.7190	12	0.5	699.8645	2	48.72	1	F1:46550	20210611-08_2021021-OK192A_815_5.3.236.mgf	0	0	0	0	0	400	411			PEAKS DB
K.FVQDFLDGK.L	N	79.93	1067.5287	9	1.0	534.7699	2	37.97	2	F2:97970	20210611-09_2021021-OK160A_816_5.3.236.mgf	0	0	0	0	0	340	348			PEAKS DB
K.NHILLFLKK.E	N	79.31	1124.7069	9	5.4	375.9102	3	30.94	1	F1:26438	20210611-08_2021021-OK192A_815_5.3.236.mgf	0	0	0	0	0	255	263			PEAKS DB
K.VQSFPTLK.Y	N	75.55	918.5175	8	3.7	460.2658	2	29.78	2	F2:82955	20210611-09_2021021-OK160A_816_5.3.236.mgf	0	0	0	0	0	435	442			PEAKS DB
K.EKPDGPADYNGGR.Q	N	73.49	1374.6163	13	-1.1	688.3121	2	13.78	1	F1:5004	20210611-08_2021021-OK192A_815_5.3.236.mgf	0		0	0	0	108	120			PEAKS DB
K.DDEIPAVR.L	N	71.69	913.4504	8	0.8	457.7310	2	24.89	2	F2:74398	20210611-09_2021021-OK160A_816_5.3.236.mgf	0	0	0	0	0	307	314			PEAKS DB
R.ITEFFGLK.D	N	66.91	953.5222	8	0.9	477.7668	2	42.32	2	F2:105336	20210611-09_2021021-OK160A_816_5.3.236.mgf	0	0	0	0	0	299	306			PEAKS DB
K.ALAPEYAK.A	N	62.34	861.4596	8	3.5	431.7368	2	19.50	2	F2:65275	20210611-09_2021021-OK160A_816_5.3.236.mgf	0	0	0	0	0	58	65			PEAKS DB
R.TLDAFIK.F	N	61.58	806.4538	7	2.3	404.2336	2	33.58	1	F1:29977	20210611-08_2021021-OK192A_815_5.3.236.mgf	0		0	0	0	459	465			PEAKS DB
K.AATTLEEEKLNVK.L	N	59.94	1444.7773	13	-0.8	482.5975	3	24.76	1	F1:17934	20210611-08_2021021-OK192A_815_5.3.236.mgf	0		0	0	0	66	78			PEAKS DB
K.EGGEDTIEK.F	N	59.47	976.4349	9	3.4	489.2243	2	12.93	2	F2:56083	20210611-09_2021021-OK160A_816_5.3.236.mgf		0	0	0	0	264	272			PEAKS DB
K.IFGGEVK.N	N	59.09	748.4119	7	4.2	375.2134	2	21.69	1	F1:13485	20210611-08_2021021-OK192A_815_5.3.236.mgf	0	0	0	0	0	248	254			PEAKS DB
K.DGIVLLK.K	N	56.48	756.4745	7	0.5	379.2433	2	35.60	1	F1:32687	20210611-08_2021021-OK192A_815_5.3.236.mgf	0		0	0	0	201	207			PEAKS DB
K.QEEGVHVLTTK.N	Y	56.41	1239.6459	11	-1.2	620.8271	2	19.69	1	F1:11068	20210611-08_2021021-OK192A_815_5.3.236.mgf	0		0	0	0	22	32			PEAKS DB
**total 33 peptides**																					

P_145_Actin

back to list

| Protein Coverage | Supporting Peptides |

Protein Coverage:

1 DPLFRQRRHK GPAVGRLLFR LFLPPVFQEL ADYYSLTFNM CDDEVAALVV DNGSGMCK**AG FAGDDAPRAV FPSIVGRPRH**

Oxidation (M) (+15.99)

84

81 **QGVMVGMGQK DSYVGDEAQS K**RGILTLK**YP IEHGIVTNWD DMEKIWHHTF YNELRVAPEE HPVLLTEAPL NPK**ANREKMT

161 QIMFETFNSP AMYVAIQAVL SLYASGR**TTG IVLDSGDGVT HTVPIYEGYA LPHAIMR**LDL AGR**DLTDYLM K**ILTER**GYSF**

241 **TTTAER**EIVR DIKEKLCYVA LDFEQEMQTA STSSSLEK**SY ELPDGQVITI GNER**FRCPEA LFQPSFLGME SAGTHETTYN

345

321 SIMKCDVDIR K**DLYANTVLS GGSTMYPGIA DR**MQK**EITAL APPTMK**IK**II APPER**KYSVW IGGSILASLS TFQQMWISK**Q**

401 **EYDESGPSIV HR**KCFLGTVE AKPGHIFRRT RRWLHLESVA LKTNYFCSES MFMFFISLEV HCFHVINMPI SNGCIIFDFF

481 YAGNK

Supporting Peptides:

Peptide	Uniq	-10lgP	Mass	Length	ppm	m/z	z	RT	Fraction	Scan	Source File	Area ebraeus	Area judaeus	#Feature	#Feature ebraeus	#Feature judaeus	Start	End	PTM	AScore	Fou B
K.SYELPDGQVITIGNER.F	Y	149.39	1789.8846	16	-1.7	895.9443	2	45.37	2	F2:111319	20210611-09_2021021-OK160A_816_5.3.236.mgf	0	0	0	0	0	279	294			PEA DI
K.DLYANTVLSGGSTMYPGIADR.M	Y	148.28	2200.0469	21	9.6	1101.0366	2	49.26	2	F2:117636	20210611-09_2021021-OK160A_816_5.3.236.mgf	0	0	0	0	0	332	352			PEA DI
K.DLYANTVLSGGSTM(+15.99)YPGIADR.M	Y	128.96	2216.0420	21	8.1	1109.0331	2	45.34	1	F1:43445	20210611-08_2021021-OK192A_815_5.3.236.mgf	0	0	0	0	0	332	352	Oxidation (M)	M14:Oxidation (M):1000.00	PEA DI
K.QEYDESGPSIVHR.K	Y	119.30	1515.6953	13	-0.8	758.8511	2	22.48	2	F2:70248	20210611-09_2021021-OK160A_816_5.3.236.mgf	0	0	0	0	0	400	412			PEA DI
R.VAPEEHPVLLTEAPLNPK.A	Y	119.08	1953.0570	18	3.7	652.0259	3	37.81	2	F2:97467	20210611-09_2021021-OK160A_816_5.3.236.mgf	0	0	0	0	0	136	153			PEA DI
R.TTGIVLDSGDGVTHTVPIYEGYALPHAIM(+15.99)R.L	Y	117.86	3198.6018	30	0.9	800.6551	4	47.63	2	F2:114753	20210611-09_2021021-OK160A_816_5.3.236.mgf		0	0	0	0	188	217		M29:Oxidation (M):1000.00	PEA DI
R.HQGVMVGMGQK.D	Y	110.56	1170.5638	11	0.3	586.2872	2	18.09	1	F1:9417	20210611-08_2021021-OK192A_815_5.3.236.mgf	0	0	0	0	0	80	90			PEA DI
K.DSYVGDEAQSK.R	Y	107.14	1197.5149	11	0.1	599.7623	2	16.16	2	F2:60394	20210611-09_2021021-OK160A_816_5.3.236.mgf	0	0	0	0	0	91	101			PEA DI
R.GYSFTTTAER.E	Y	106.28	1131.5197	10	4.2	566.7671	2	25.23	2	F2:75101	20210611-09_2021021-OK160A_816_5.3.236.mgf	0	0	0	0	0	237	246			PEA DI
K.Q(-17.03)EYDESGPSIVHR.K	Y	103.01	1498.6688	13	1.1	750.3394	2	28.64	2	F2:81266	20210611-09_2021021-OK160A_816_5.3.236.mgf	0	0	0	0	0	400	412		Q1:Pyro-glu from Q:1000.00	PEA DI
R.TTGIVLDSGDGVTHTVPIYEGYALPHAIMR.L	Y	100.28	3182.6069	30	-1.0	796.6549	4	50.51	2	F2:119802	20210611-09_2021021-OK160A_816_5.3.236.mgf		0	0	0	0	188	217			PEA DI
R.HQGVM(+15.99)VGMGQK.D	Y	96.88	1186.5587	11	1.2	594.2851	2	14.84	1	F1:6000	20210611-08_2021021-OK192A_815_5.3.236.mgf	0	0	0	0	0	80	90	Oxidation (M)	M5:Oxidation (M):62.26	PEA DI
R.HQGVMVGM(+15.99)GQK.D	Y	90.04	1186.5587	11	-2.1	594.2829	2	12.35	2	F2:55322	20210611-09_2021021-OK160A_816_5.3.236.mgf	0	0	0	0	0	80	90		M8:Oxidation (M):32.69	PEA DI
R.AVFPSIVGRPR.H	Y	88.53	1197.6981	11	1.6	599.8548	2	32.67	2	F2:88257	20210611-09_2021021-OK160A_816_5.3.236.mgf	0	0	0	0	0	69	79			PEA DI
K.EITALAPPTMK.I	Y	86.49	1170.6318	11	0.7	586.3212	2	35.31	2	F2:93027	20210611-09_2021021-OK160A_816_5.3.236.mgf	0	0	0	0	0	356	366			PEA DI
K.AGFAGDDAPR.A	Y	85.75	975.4410	10	2.6	488.7272	2	18.67	1	F1:9951	20210611-08_2021021-OK192A_815_5.3.236.mgf	0	0	0	0	0	59	68			PEA DI
K.IWHHTFYNELR.V	Y	83.00	1514.7418	11	3.8	505.9212	3	28.52	1	F1:23270	20210611-08_2021021-OK192A_815_5.3.236.mgf	0	0	0	0	0	125	135			PEA DI
R.DLTDYLMK.I	Y	81.70	997.4790	8	-1.1	499.7441	2	43.74	2	F2:107943	20210611-09_2021021-OK160A_816_5.3.236.mgf	0	0	0	0	0	224	231			PEA DI

total 22 peptides

Peptide	Uniq	-10lgP	Mass	Length	ppm	m/z	z	RT	Fraction	Scan	Source File	Area ebraeus	Area judaeus	#Feature	#Feature ebraeus	#Feature judaeus	Start	End	PTM	AScore	Fou By
R.HQGVM(+15.99)VGM(+15.99)GQK.D	Y	74.72	1202.5536	11	-0.2	602.2817	2	10.25	1	F1:1455	20210611-08_2021021-OK192A_815_5.3.236.mgf	0	0	0	0	0	80	90		M5:Oxidation (M):1000.00; M8:Oxidation (M):1000.00	PEA DI
K.YP(+13.98)IEHGIVTNWDDMEK.I	Y	68.74	1959.8673	16	13.2	654.3026	3	40.68	1	F1:38400	20210611-08_2021021-OK192A_815_5.3.236.mgf	0		0	0	0	109	124		P2:Proline oxidation to pyroglutamic acid:1000.00	PEA DI
K.IIAPPER.K	Y	64.70	794.4650	7	5.1	398.2401	2	20.32	2	F2:66482	20210611-09_2021021-OK160A_816_5.3.236.mgf	0	0	0	0	0	369	375			PEA DI
R.DLTDYLM(+15.99)K.I	Y	61.39	1013.4739	8	-2.0	507.7413	2	37.02	1	F1:34442	20210611-08_2021021-OK192A_815_5.3.236.mgf	0		0	0	0	224	231		M7:Oxidation (M):1000.00	PEA DI
**total 22 peptides**																					

OK212_J_286_Conoporin

back to list

| Protein Coverage | Supporting Peptides |

Protein Coverage:

67 C

1 MRVPFPALKT MVTVFLLLMG NMSPVVPRSS TPLHKVKVLA YK**VVTPGNSL NGVTLQDLAA TNYKVTCVIQ VENWTRYPLI**

C Carbamidomethylation (+57.02)
O Oxidation (M) (+15.99)

151 O

81 **APR**LR**IANDG AATTSPVAIL PGKR**EAFAVR KASNVR**KGVY GTVSWELLGA R**RRFVIMWSA PYSFNK**YSNW MGLGMTR**EGL

161 MDVAPDNTWF NQMYYGSSSG NLNFKR**KEFY YNTDPFIYSN EKFEAEGDMT NGHHAQVR**VI IRPTR**NNWED LADEIR**QNMV

Supporting Peptides:

Peptide	Uniq	-10lgP	Mass	Length	ppm	m/z	z	RT	Fraction	Scan	Source File	Area ebraeus	Area judaeus	#Feature	#Feature ebraeus	#Feature judaeus	Start	End	PTM	AScore	Fou By
K.VVTPGNSLNGVTLQDLAATNYK.V	N	159.55	2274.1855	22	12.3	1138.1093	2	47.99	2	F2:115425	20210611-09_2021021-OK160A_816_5.3.236.mgf		0	0	0	0	43	64			PEAI DE
R.IANDGAATTSPVAILPGK.R	Y	134.75	1694.9203	18	-0.1	848.4637	2	37.25	2	F2:96503	20210611-09_2021021-OK160A_816_5.3.236.mgf		0	0	0	0	86	103			PEAI DE
R.IANDGAATTSPVAILPGKR.E	Y	119.82	1851.0214	19	3.1	618.0137	3	32.78	2	F2:88130	20210611-09_2021021-OK160A_816_5.3.236.mgf		0	0	0	0	86	104			PEAI DE
K.FEAEGDMTNGHHAQVR.V	N	118.42	1797.7853	16	2.2	600.2678	3	18.47	2	F2:63606	20210611-09_2021021-OK160A_816_5.3.236.mgf		0	0	0	0	203	218			PEAI DE
K.VTC(+57.02)VIQVENWTR.Y	N	117.26	1503.7504	12	0.3	752.8795	2	40.90	2	F2:103099	20210611-09_2021021-OK160A_816_5.3.236.mgf		0	0	0	0	65	76	Carbamidomethylation	C3:Carbamidomethylation:1000.00	PEAI DE
K.EFYYNTDPFIYSNEK.F	N	116.83	1928.8468	15	1.6	965.4282	2	46.01	2	F2:112287	20210611-09_2021021-OK160A_816_5.3.236.mgf		0	0	0	0	188	202			PEAI DE
R.KGVYGTVSWELLGAR.R	N	113.88	1634.8781	15	-1.9	545.9633	3	44.01	2	F2:108515	20210611-09_2021021-OK160A_816_5.3.236.mgf		0	0	0	0	117	131			PEAI DE
K.GVYGTVSWELLGAR.R	N	109.86	1506.7831	14	-0.7	754.3951	2	50.27	2	F2:119430	20210611-09_2021021-OK160A_816_5.3.236.mgf		0	0	0	0	118	131			PEAI DE
K.FEAEGDM(+15.99)TNGHHAQVR.V	N	104.09	1813.7802	16	-0.5	605.5978	3	13.73	2	F2:57220	20210611-09_2021021-OK160A_816_5.3.236.mgf		0	0	0	0	203	218		M7:Oxidation (M):1000.00	PEAI DE
R.NNWEDLADEIR.Q	N	99.52	1373.6211	11	0.4	687.8152	2	45.04	2	F2:110772	20210611-09_2021021-OK160A_816_5.3.236.mgf		0	0	0	0	226	236			PEAI DE
R.KEFYYNTDPFIYSNEK.F	N	96.57	2056.9417	16	2.0	686.6530	3	40.01	2	F2:101239	20210611-09_2021021-OK160A_816_5.3.236.mgf		0	0	0	0	187	202			PEAI DE
R.YPLIAPR.L	N	89.94	828.4857	7	3.7	415.2499	2	29.51	2	F2:82428	20210611-09_2021021-OK160A_816_5.3.236.mgf		0	0	0	0	77	83			PEAI DE
K.YSNWM(+15.99)GLGM(+15.99)TR.E	N	88.85	1346.5747	11	1.8	674.2933	2	30.49	1	F1:25789	20210611-08_2021021-OK192A_815_5.3.236.mgf	0	0	0	0	0	147	157	Oxidation (M)	M5:Oxidation (M):1000.00; M9:Oxidation (M):1000.00	PEAI DE
K.YSNWMGLGMTR.E	N	84.79	1314.5850	11	-0.4	658.2970	2	42.62	1	F1:40524	20210611-08_2021021-OK192A_815_5.3.236.mgf	0	0	0	0	0	147	157			PEAI DE
K.YSNWM(+15.99)GLGMTR.E	N	80.81	1330.5798	11	5.0	666.2980	2	35.70	1	F1:32686	20210611-08_2021021-OK192A_815_5.3.236.mgf	0	0	0	0	0	147	157	Oxidation (M)	M5:Oxidation (M):55.36	PEAI DE
K.YSNWMGLGM(+15.99)TR.E	N	80.30	1330.5798	11	4.5	666.2974	2	37.39	2	F2:96896	20210611-09_2021021-OK160A_816_5.3.236.mgf	0	0	0	0	0	147	157		M9:Oxidation (M):41.04	PEAI DE
**total 16 peptides**																					

CebrOK033_J_240_Conoporin

back to list

| Protein Coverage | Supporting Peptides |

Protein Coverage:

67 C

1 MRVPFPALKT MVTVFLLLMG NMSPVVPRSS TPLHKVKVLA YK**VVTPGNSL NGVTLQDLAA TNYKVTCVIQ VENWTRYPLI**

C Carbamidomethylation (+57.02)
O Oxidation (M) (+15.99)

151 O

81 **APR**LRIAK**DG AVTTSPVAIL PGKR**EAFAVR KASYVR**KGVY GTVSWELLGA R**RRFVIMWSA PYSFNK**YSNW MGLGMTR**EGL

161 MDVAPDNTWF NQMYYGSSSG NLTFKR**KEFY YNTDPFIYSN EKFEAEGDMT NGHHAQVR**VI IRPTR**NNWED LADEIR**QNMV

Supporting Peptides:

Peptide	Uniq	-10lgP	Mass	Length	ppm	m/z	z	RT	Fraction	Scan	Source File	Area ebraeus	Area judaeus	#Feature	#Feature ebraeus	#Feature judaeus	Start	End	PTM	AScore	Fou By
K.VVTPGNSLNGVTLQDLAATNYK.V	N	159.55	2274.1855	22	12.3	1138.1093	2	47.99	2	F2:115425	20210611-09_2021021-OK160A_816_5.3.236.mgf		0	0	0	0	43	64			PEAI DE
K.DGAVTTSPVAILPGK.R	Y	123.00	1424.7875	15	0.7	713.3985	2	41.62	2	F2:104154	20210611-09_2021021-OK160A_816_5.3.236.mgf		0	0	0	0	89	103			PEAI DE
K.DGAVTTSPVAILPGKR.E	Y	121.42	1580.8885	16	0.3	791.4485	2	34.49	2	F2:91442	20210611-09_2021021-OK160A_816_5.3.236.mgf		0	0	0	0	89	104			PEAI DE
K.FEAEGDMTNGHHAQVR.V	N	118.42	1797.7853	16	2.2	600.2678	3	18.47	2	F2:63606	20210611-09_2021021-OK160A_816_5.3.236.mgf		0	0	0	0	203	218			PEAI DE
K.VTC(+57.02)VIQVENWTR.Y	N	117.26	1503.7504	12	0.3	752.8795	2	40.90	2	F2:103099	20210611-09_2021021-OK160A_816_5.3.236.mgf		0	0	0	0	65	76	Carbamidomethylation	C3:Carbamidomethylation:1000.00	PEAI DE
K.EFYYNTDPFIYSNEK.F	N	116.83	1928.8468	15	1.6	965.4282	2	46.01	2	F2:112287	20210611-09_2021021-OK160A_816_5.3.236.mgf		0	0	0	0	188	202			PEAI DE
R.KGVYGTVSWELLGAR.R	N	113.88	1634.8781	15	-1.9	545.9633	3	44.01	2	F2:108515	20210611-09_2021021-OK160A_816_5.3.236.mgf		0	0	0	0	117	131			PEAI DE

total 16 peptides

Peptide	Uniq	-10lgP	Mass	Length	ppm	m/z	z	RT	Fraction	Scan	Source File	Area ebraeus	Area judaeus	#Feature	#Feature ebraeus	#Feature judaeus	Start	End	PTM	AScore	Fou By
K.GVYGTVSWELLGAR.R	N	109.86	1506.7831	14	-0.7	754.3951	2	50.27	2	F2:119430	20210611-09_2021021-OK160A_816_5.3.236.mgf		0	0	0	0	118	131			PEAI DE
K.FEAEGDM(+15.99)TNGHHAQVR.V	N	104.09	1813.7802	16	-0.5	605.5978	3	13.73	2	F2:57220	20210611-09_2021021-OK160A_816_5.3.236.mgf		0	0	0	0	203	218		M7:Oxidation (M):1000.00	PEAI DE
R.NNWEDLADEIR.Q	N	99.52	1373.6211	11	0.4	687.8152	2	45.04	2	F2:110772	20210611-09_2021021-OK160A_816_5.3.236.mgf		0	0	0	0	226	236			PEAI DE
R.KEFYYNTDPFIYSNEK.F	N	96.57	2056.9417	16	2.0	686.6530	3	40.01	2	F2:101239	20210611-09_2021021-OK160A_816_5.3.236.mgf		0	0	0	0	187	202			PEAI DE
R.YPLIAPR.L	N	89.94	828.4857	7	3.7	415.2499	2	29.51	2	F2:82428	20210611-09_2021021-OK160A_816_5.3.236.mgf		0	0	0	0	77	83			PEAI DE
K.YSNWM(+15.99)GLGM(+15.99)TR.E	N	88.85	1346.5747	11	1.8	674.2933	2	30.49	1	F1:25789	20210611-08_2021021-OK192A_815_5.3.236.mgf	0	0	0	0	0	147	157	Oxidation (M)	M5:Oxidation (M):1000.00; M9:Oxidation (M):1000.00	PEAI DE
K.YSNWMGLGMTR.E	N	84.79	1314.5850	11	-0.4	658.2970	2	42.62	1	F1:40524	20210611-08_2021021-OK192A_815_5.3.236.mgf	0	0	0	0	0	147	157			PEAI DE
K.YSNWM(+15.99)GLGMTR.E	N	80.81	1330.5798	11	5.0	666.2980	2	35.70	1	F1:32686	20210611-08_2021021-OK192A_815_5.3.236.mgf	0	0	0	0	0	147	157	Oxidation (M)	M5:Oxidation (M):55.36	PEAI DE
K.YSNWMGLGM(+15.99)TR.E	N	80.30	1330.5798	11	4.5	666.2974	2	37.39	2	F2:96896	20210611-09_2021021-OK160A_816_5.3.236.mgf	0	0	0	0	0	147	157		M9:Oxidation (M):41.04	PEAI DE
total 16 peptides																					

OK213_E_299_Conoporin

back to list

| Protein Coverage | Supporting Peptides |

Protein Coverage:

```
  1 MRVPFPALKT MVTVFLLLMG NMSPVVPRSS IPLDKVKVLA YEVVTSGNSL NGVTLQDLAE TEYDVTCVVQ VENWTRYPLM
                                                 137             151
 81 TPRQRIANDG AWTTYPVAIL PGKREAFAVR KASGVKKGVY GIVSWELLGA RRRFVIMWSA PYDLNKYSNW MGLGMTREGL
161 TDVAPGDTWF DQMYSDKSSG SLTFERKEFY HNTDPVIYRN AKFEAEGAMT NGNHAQVKIV VRPTRNNWKD LADEIRQKLV
```

o Oxidation (M) (+15.99)

Supporting Peptides:

Peptide	Uniq	-10lgP	Mass	Length	ppm	m/z	z	RT	Fraction	Scan	Source File	Area ebraeus	Area judaeus	#Feature	#Feature ebraeus	#Feature judaeus	Start	End	PTM	AScore	Found By
R.IANDGAWTTYPVAILPGK.R	Y	143.54	1885.9938	18	1.6	944.0021	2	50.62	1	F1:47729	20210611-08_2021021-OK192A_815_5.3.236.mgf	0	0	0	0	0	86	103			PEAKS DB
R.IANDGAWTTYPVAILPGKR.E	Y	118.82	2042.0948	19	-1.1	681.7022	3	45.81	1	F1:44012	20210611-08_2021021-OK192A_815_5.3.236.mgf	0		0	0	0	86	104			PEAKS DB
K.EFYHNTDPVIYR.N	Y	114.40	1552.7310	12	2.0	777.3714	2	30.56	1	F1:25946	20210611-08_2021021-OK192A_815_5.3.236.mgf	0		0	0	0	188	199			PEAKS DB
K.FEAEGAMTNGNHAQVK.I	Y	110.64	1702.7733	16	0.6	852.3912	2	20.95	1	F1:12500	20210611-08_2021021-OK192A_815_5.3.236.mgf	0		0	0	0	203	218			PEAKS DB
R.KEFYHNTDPVIYR.N	Y	109.38	1680.8259	13	-0.4	561.2802	3	25.39	1	F1:18789	20210611-08_2021021-OK192A_815_5.3.236.mgf	0		0	0	0	187	199			PEAKS DB
R.FVIM(+15.99)WSAPYDLNK.Y	N	105.41	1598.7803	13	0.4	800.3947	2	51.09	1	F1:47971	20210611-08_2021021-OK192A_815_5.3.236.mgf	0		0	0	0	134	146	Oxidation (M)	M4:Oxidation (M):1000.00	PEAKS DB
R.FVIMWSAPYDLNK.Y	N	102.70	1582.7854	13	-2.0	792.3954	2	54.03	1	F1:49661	20210611-08_2021021-OK192A_815_5.3.236.mgf	0		0	0	0	134	146			PEAKS DB
K.E(-18.01)FYHNTDPVIYR.N	Y	88.94	1534.7205	12	2.7	768.3667	2	38.67	1	F1:36170	20210611-08_2021021-OK192A_815_5.3.236.mgf	0		0	0	0	188	199		E1:Pyro-glu from E:1000.00	PEAKS DB
K.YSNWM(+15.99)GLGM(+15.99)TR.E	N	88.85	1346.5747	11	1.8	674.2933	2	30.49	1	F1:25789	20210611-08_2021021-OK192A_815_5.3.236.mgf	0	0	0	0	0	147	157	Oxidation (M)	M5:Oxidation (M):1000.00; M9:Oxidation (M):1000.00	PEAKS DB
K.GVYGIVSWELLGAR.R	Y	87.81	1518.8195	14	-1.4	760.4131	2	56.52	1	F1:50680	20210611-08_2021021-OK192A_815_5.3.236.mgf	0		0	0	0	118	131			PEAKS DB
K.KGVYGIVSWELLGAR.R	Y	86.85	1646.9144	15	0.7	549.9771	3	50.31	1	F1:47548	20210611-08_2021021-OK192A_815_5.3.236.mgf	0		0	0	0	117	131			PEAKS DB
R.EGLTDVAPGDTWFDQMYSDK.S	Y	85.64	2273.9785	20	8.7	1138.0021	2	53.76	1	F1:49528	20210611-08_2021021-OK192A_815_5.3.236.mgf	0		0	0	0	158	177			PEAKS DB
R.EGLTDVAPGDTWFDQM(+15.99)YSDK.S	Y	85.13	2289.9736	20	5.9	1145.9965	2	48.12	1	F1:46085	20210611-08_2021021-OK192A_815_5.3.236.mgf	0		0	0	0	158	177		M16:Oxidation (M):1000.00	PEAKS DB
K.YSNWMGLGMTR.E	N	84.79	1314.5850	11	-0.4	658.2970	2	42.62	1	F1:40524	20210611-08_2021021-OK192A_815_5.3.236.mgf	0	0	0	0	0	147	157			PEAKS DB
K.SSGSLTFER.K	Y	84.61	982.4719	9	5.0	492.2438	2	23.61	1	F1:16053	20210611-08_2021021-OK192A_815_5.3.236.mgf	0		0	0	0	178	186			PEAKS DB
K.YSNWM(+15.99)GLGMTR.E	N	80.81	1330.5798	11	5.0	666.2980	2	35.70	1	F1:32686	20210611-08_2021021-OK192A_815_5.3.236.mgf	0	0	0	0	0	147	157	Oxidation (M)	M5:Oxidation (M):55.36	PEAKS DB
K.YSNWMGLGM(+15.99)TR.E	N	80.30	1330.5798	11	4.5	666.2974	2	37.39	2	F2:96896	20210611-09_2021021-OK160A_816_5.3.236.mgf	0	0	0	0	0	147	157		M9:Oxidation (M):41.04	PEAKS DB
K.FEAEGAM(+15.99)TNGNHAQVK.I	Y	79.05	1718.7682	16	2.8	573.9294	3	15.91	1	F1:7008	20210611-08_2021021-OK192A_815_5.3.236.mgf	0		0	0	0	203	218		M7:Oxidation (M):1000.00	PEAKS DB
R.YPLMTPR.Q	Y	59.21	876.4528	7	1.3	439.2326	2	27.92	1	F1:22536	20210611-08_2021021-OK192A_815_5.3.236.mgf	0		0	0	0	77	83			PEAKS DB
K.DLADEIR.Q	Y	57.73	830.4133	7	2.7	416.2135	2	28.49	1	F1:23171	20210611-08_2021021-OK192A_815_5.3.236.mgf	0		0	0	0	230	236			PEAKS DB
total 20 peptides																					

OK193_E_246_Conoporin

back to list

| Protein Coverage | Supporting Peptides |

Protein Coverage:

```
  1 MRVPFPALKT MVTVFLLLMG NMSPVVPRSS IPLDKVKVLA YEVVTSGNSL NGVTLQDLAE TEYDVTCVVQ VENWTRYPLM
                                                 137             151
 81 TPRQRIANDG AWTTYPVAIL PGKREAFAVR KASGVKKGVY GIVSWELLGA RRRFVIMWSA PYDLNKYSNW MGLGMTREGL
161 TDVAPGDTWF DQMYSDKSSG SLTFERKEFY HNTDPVIYRN AKFEAEGAMT NGNHAQVKIV VRPTRNNWKD LADEIRQKLV
```

o Oxidation (M) (+15.99)

Supporting Peptides:

Peptide	Uniq	-10lgP	Mass	Length	ppm	m/z	z	RT	Fraction	Scan	Source File	Area ebraeus	Area judaeus	#Feature	#Feature ebraeus	#Feature judaeus	Start	End	PTM	AScore	Found By
R.IANDGAWTTYPVAILPGK.R	Y	143.54	1885.9938	18	1.6	944.0021	2	50.62	1	F1:47729	20210611-08_2021021-OK192A_815_5.3.236.mgf	0	0	0	0	0	86	103			PEAKS DB
R.IANDGAWTTYPVAILPGKR.E	Y	118.82	2042.0948	19	-1.1	681.7022	3	45.81	1	F1:44012	20210611-08_2021021-OK192A_815_5.3.236.mgf	0		0	0	0	86	104			PEAKS DB
K.EFYHNTDPVIYR.N	Y	114.40	1552.7310	12	2.0	777.3714	2	30.56	1	F1:25946	20210611-08_2021021-OK192A_815_5.3.236.mgf	0		0	0	0	188	199			PEAKS DB
K.FEAEGAMTNGNHAQVK.I	Y	110.64	1702.7733	16	0.6	852.3912	2	20.95	1	F1:12500	20210611-08_2021021-OK192A_815_5.3.236.mgf	0		0	0	0	203	218			PEAKS DB
R.KEFYHNTDPVIYR.N	Y	109.38	1680.8259	13	-0.4	561.2802	3	25.39	1	F1:18789	20210611-08_2021021-OK192A_815_5.3.236.mgf	0		0	0	0	187	199			PEAKS DB

total 20 peptides

Peptide	Uniq	-10lgP	Mass	Length	ppm	m/z	z	RT	Fraction	Scan	Source File	Area ebraeus	Area judaeus	#Feature	#Feature ebraeus	#Feature judaeus	Start	End	PTM	AScore	Found By
R.FVIM(+15.99)WSAPYDLNK.Y	N	105.41	1598.7803	13	0.4	800.3947	2	51.09	1	F1:47971	20210611-08_2021021-OK192A_815_5.3.236.mgf	0		0	0	0	134	146	Oxidation (M)	M4:Oxidation (M):1000.00	PEAKS DB
R.FVIMWSAPYDLNK.Y	N	102.70	1582.7854	13	-2.0	792.3954	2	54.03	1	F1:49661	20210611-08_2021021-OK192A_815_5.3.236.mgf	0		0	0	0	134	146			PEAKS DB
K.E(-18.01)FYHNTDPVIYR.N	Y	88.94	1534.7205	12	2.7	768.3667	2	38.67	1	F1:36170	20210611-08_2021021-OK192A_815_5.3.236.mgf	0		0	0	0	188	199		E1:Pyro-glu from E:1000.00	PEAKS DB
K.YSNWM(+15.99)GLGM(+15.99)TR.E	N	88.85	1346.5747	11	1.8	674.2933	2	30.49	1	F1:25789	20210611-08_2021021-OK192A_815_5.3.236.mgf	0	0	0	0	0	147	157	Oxidation (M)	M5:Oxidation (M):1000.00; M9:Oxidation (M):1000.00	PEAKS DB
K.GVYGIVSWELLGAR.R	Y	87.81	1518.8195	14	-1.4	760.4131	2	56.52	1	F1:50680	20210611-08_2021021-OK192A_815_5.3.236.mgf	0		0	0	0	118	131			PEAKS DB
K.KGVYGIVSWELLGAR.R	Y	86.85	1646.9144	15	0.7	549.9771	3	50.31	1	F1:47548	20210611-08_2021021-OK192A_815_5.3.236.mgf	0		0	0	0	117	131			PEAKS DB
R.EGLTDVAPGDTWFDQMYSDK.S	Y	85.64	2273.9785	20	8.7	1138.0021	2	53.76	1	F1:49528	20210611-08_2021021-OK192A_815_5.3.236.mgf	0		0	0	0	158	177			PEAKS DB
R.EGLTDVAPGDTWFDQM(+15.99)YSDK.S	Y	85.13	2289.9736	20	5.9	1145.9965	2	48.12	1	F1:46085	20210611-08_2021021-OK192A_815_5.3.236.mgf	0		0	0	0	158	177		M16:Oxidation (M):1000.00	PEAKS DB
K.YSNWMGLGMTR.E	N	84.79	1314.5850	11	-0.4	658.2970	2	42.62	1	F1:40524	20210611-08_2021021-OK192A_815_5.3.236.mgf	0	0	0	0	0	147	157			PEAKS DB
K.SSGSLTFER.K	Y	84.61	982.4719	9	5.0	492.2438	2	23.61	1	F1:16053	20210611-08_2021021-OK192A_815_5.3.236.mgf	0		0	0	0	178	186			PEAKS DB
K.YSNWM(+15.99)GLGMTR.E	N	80.81	1330.5798	11	5.0	666.2980	2	35.70	1	F1:32686	20210611-08_2021021-OK192A_815_5.3.236.mgf	0	0	0	0	0	147	157	Oxidation (M)	M5:Oxidation (M):55.36	PEAKS DB
K.YSNWMGLGM(+15.99)TR.E	N	80.30	1330.5798	11	4.5	666.2974	2	37.39	2	F2:96896	20210611-09_2021021-OK160A_816_5.3.236.mgf	0	0	0	0	0	147	157		M9:Oxidation (M):41.04	PEAKS DB
K.FEAEGAM(+15.99)TNGNHAQVK.I	Y	79.05	1718.7682	16	2.8	573.9294	3	15.91	1	F1:7008	20210611-08_2021021-OK192A_815_5.3.236.mgf	0		0	0	0	203	218		M7:Oxidation (M):1000.00	PEAKS DB
R.YPLMTPR.Q	Y	59.21	876.4528	7	1.3	439.2326	2	27.92	1	F1:22536	20210611-08_2021021-OK192A_815_5.3.236.mgf	0		0	0	0	77	83			PEAKS DB
K.DLADEIR.Q	Y	57.73	830.4133	7	2.7	416.2135	2	28.49	1	F1:23171	20210611-08_2021021-OK192A_815_5.3.236.mgf	0		0	0	0	230	236			PEAKS DB
**total 20 peptides**																					

CebrOK035_J_368_PDI

back to list

| Protein Coverage | Supporting Peptides |

Protein Coverage:

Oxidation (M) (+15.99)

```
  1  MLLAYFRPSA FTSPATMRRA LILFSCIVLA VQASDVLEFT DSNFESKIKE LDVALVEFYA PWCGHCKRLA PEYEKAATKL
 81  KTSDPPVPLV KVDCTAETKT CQEYGVSGYP TLKIFKGGEL TKDYTGPRES NGIISTMLKE AGPVSKELED FKQYEDFIGR
                                                     202
161  GTVCVVGFFE DADSEMAKTF SKLADTNEAD IRFGHTTASA VMDKAKYKDE IVLFRPKAMQ NKFEDSVVKF TGDAKKKSAV
241  KEFLTQEHLG MCGIRTINNA GDFSKPTFVA FYDVDYVKNP KGSNYWRNRV MKVGKKLRDE GLKVFFGVSN KEEHSHELGE
321  CGLQDAEGDK PIVCAYDEKN RKFKMEEEFS VDSFEKFVRG VLNGEVEPYL KSEPVPENND QPVKVVVAKN FEELVNDPEK
401  DVLIEFYAPW CGHCKSLAPK YDELAEKLKD ETDIVIAKMD ATANDPPSQY EVRGFPTIYF APKGSKSSPK KYEGGREVND
481  FIKYLAKEAT DELQGFDRKG KKKGGQKQQK KKKTEL
```

Supporting Peptides:

Peptide	Uniq	-10lgP	Mass	Length	ppm	m/z	z	RT	Fraction	Scan	Source File	Area ebraeus	Area judaeus	#Feature	#Feature ebraeus	#Feature judaeus	Start	End	PTM	AScore	Found By
K.M(+15.99)DATANDPPSQYEVR.G	N	116.71	1708.7362	15	-0.3	855.3715	2	23.22	2	F2:71559	20210611-09_2021021-OK160A_816_5.3.236.mgf		0	0	0	0	439	453		M1:Oxidation (M):1000.00	PEAKS DB
K.SEPVPENNDQPVK.V	N	112.89	1451.6892	13	2.8	726.8509	2	19.00	2	F2:64401	20210611-09_2021021-OK160A_816_5.3.236.mgf	0	0	0	0	0	372	384			PEAKS DB
R.FGHTTASAVMDK.A	Y	110.21	1263.5918	12	0.4	632.8008	2	19.17	2	F2:64682	20210611-09_2021021-OK160A_816_5.3.236.mgf		0	0	0	0	193	204			PEAKS DB
R.FGHTTASAVM(+15.99)DK.A	Y	110.09	1279.5867	12	-4.6	640.7950	2	14.30	2	F2:57824	20210611-09_2021021-OK160A_816_5.3.236.mgf		0	0	0	0	193	204	Oxidation (M)	M10:Oxidation (M):1000.00	PEAKS DB
K.MDATANDPPSQYEVR.G	N	106.16	1692.7413	15	0.4	847.3748	2	26.03	2	F2:76318	20210611-09_2021021-OK160A_816_5.3.236.mgf		0	0	0	0	439	453			PEAKS DB
K.EATDELQGFDR.K	N	95.64	1279.5680	11	0.1	640.7886	2	29.87	2	F2:83300	20210611-09_2021021-OK160A_816_5.3.236.mgf	0	0	0	0	0	488	498			PEAKS DB
K.TSDPPVPLVK.V	Y	89.26	1051.5913	10	2.2	526.8019	2	29.96	2	F2:83364	20210611-09_2021021-OK160A_816_5.3.236.mgf	0	0	0	0	0	82	91			PEAKS DB
R.GFPTIYFAPK.G	N	86.32	1139.6014	10	-0.9	570.8051	2	46.08	2	F2:112594	20210611-09_2021021-OK160A_816_5.3.236.mgf	0	0	0	0	0	454	463			PEAKS DB
K.LADTNEADIR.F	Y	85.89	1116.5411	10	5.7	559.2787	2	19.91	2	F2:65906	20210611-09_2021021-OK160A_816_5.3.236.mgf		0	0	0	0	183	192			PEAKS DB
R.GVLNGEVEPYLK.S	Y	82.43	1316.6975	12	-0.3	659.3531	2	39.10	2	F2:99793	20210611-09_2021021-OK160A_816_5.3.236.mgf		0	0	0	0	360	371			PEAKS DB
K.LKDETDIVIAK.M	N	82.41	1243.7023	11	0.0	622.8558	2	25.53	2	F2:75618	20210611-09_2021021-OK160A_816_5.3.236.mgf		0	0	0	0	428	438			PEAKS DB
K.YKDEIVLFRPK.A	N	82.19	1406.7921	11	-0.6	469.9357	3	31.20	2	F2:85517	20210611-09_2021021-OK160A_816_5.3.236.mgf		0	0	0	0	207	217			PEAKS DB
K.VFFGVSNK.E	N	77.53	896.4756	8	2.5	449.2443	2	30.55	2	F2:84259	20210611-09_2021021-OK160A_816_5.3.236.mgf	0	0	0	0	0	304	311			PEAKS DB
K.YDELAEK.L	N	64.80	866.4021	7	4.6	434.2085	2	16.53	2	F2:60959	20210611-09_2021021-OK160A_816_5.3.236.mgf		0	0	0	0	421	427			PEAKS DB
K.QYEDFIGR.G	N	64.14	1026.4771	8	3.1	514.2452	2	30.02	2	F2:83542	20210611-09_2021021-OK160A_816_5.3.236.mgf		0	0	0	0	153	160			PEAKS DB
K.RLAPEYEK.A	N	61.68	1004.5291	8	1.7	503.2705	2	15.00	2	F2:59037	20210611-09_2021021-OK160A_816_5.3.236.mgf		0	0	0	0	68	75			PEAKS DB
K.FEDSVVK.F	Y	59.31	822.4123	7	2.4	412.2127	2	20.11	2	F2:66181	20210611-09_2021021-OK160A_816_5.3.236.mgf		0	0	0	0	223	229			PEAKS DB
K.TC(+57.02)QEYGVSGYPTLK.I	N	58.68	1601.7395	14	0.4	801.8740	2	32.78	2	F2:88088	20210611-09_2021021-OK160A_816_5.3.236.mgf		0	0	0	0	100	113		C2:Carbamidomethylation:1000.00	PEAKS DB
**total 18 peptides**																					

OK212_J_325_PDI

back to list

| Protein Coverage | Supporting Peptides |

Protein Coverage:

1 MLLAYFRPSA FTSPATMRRA LILFSCIVLA VQASDVLEFT DSNFESKIKE LDVALVEFYA PWCGHCK**RLA PEYEK**AATKL   Oxidation (M) (+15.99)

81 K**TSDPPVPLV K**VDCTAETK**T CQEYGVSGYP TLK**IFKGGEL TKDYTGPRES NGIISTMLKE AGPVSKELED FK**QYEDFIGR**

202

161 GTVCVVGFFE DADSEMAKTF SK**LADTNEAD IRFGHTTASA VMDK**AK**YKDE IVLFRPK**AMQ NK**FEDSVVK**F TGDAKKKSAV

241 KEFLTQEHLG MCGIRTINNA GDFSKPTFVA FYDVDYVKNP KGSNYWRNRV MKVGKKLRDE GLK**VFFGVSN K**EEHSHELGE

321 CGLQDAEGDK PIVCAYDEKN RKFKMEEEFS VDSFEKFVR**G VLNGEVEPYL KSEPVPENND QPVK**VVVAKN FEELVNDPEK

401 DVLIEFYAPW CGHCKSLAPK **YDELAEKLKD ETDIVIAKMD ATANDPPSQY EVRGFPTIYF APK**GSKSSPK KYEGGREVND

481 FIKYLAK**EAT DELQGFDR**KG KKKGGQKQQK KKKTEL

Supporting Peptides:

Peptide	Uniq	-10lgP	Mass	Length	ppm	m/z	z	RT	Fraction	Scan	Source File	Area ebraeus	Area judaeus	#Feature	#Feature ebraeus	#Feature judaeus	Start	End	PTM	AScore	Found By
K.M(+15.99)DATANDPPSQYEVR.G	N	116.71	1708.7362	15	-0.3	855.3715	2	23.22	2	F2:71559	20210611-09_2021021-OK160A_816_5.3.236.mgf		0	0	0	0	439	453		M1:Oxidation (M):1000.00	PEAKS DB
K.SEPVPENNDQPVK.V	N	112.89	1451.6892	13	2.8	726.8509	2	19.00	2	F2:64401	20210611-09_2021021-OK160A_816_5.3.236.mgf	0	0	0	0	0	372	384			PEAKS DB
R.FGHTTASAVMDK.A	Y	110.21	1263.5918	12	0.4	632.8008	2	19.17	2	F2:64682	20210611-09_2021021-OK160A_816_5.3.236.mgf		0	0	0	0	193	204			PEAKS DB
R.FGHTTASAVM(+15.99)DK.A	Y	110.09	1279.5867	12	-4.6	640.7950	2	14.30	2	F2:57824	20210611-09_2021021-OK160A_816_5.3.236.mgf		0	0	0	0	193	204	Oxidation (M)	M10:Oxidation (M):1000.00	PEAKS DB
K.MDATANDPPSQYEVR.G	N	106.16	1692.7413	15	0.4	847.3748	2	26.03	2	F2:76318	20210611-09_2021021-OK160A_816_5.3.236.mgf		0	0	0	0	439	453			PEAKS DB
K.EATDELQGFDR.K	N	95.64	1279.5680	11	0.1	640.7886	2	29.87	2	F2:83300	20210611-09_2021021-OK160A_816_5.3.236.mgf	0	0	0	0	0	488	498			PEAKS DB
K.TSDPPVPLVK.V	Y	89.26	1051.5913	10	2.2	526.8019	2	29.96	2	F2:83364	20210611-09_2021021-OK160A_816_5.3.236.mgf	0	0	0	0	0	82	91			PEAKS DB
R.GFPTIYFAPK.G	N	86.32	1139.6014	10	-0.9	570.8051	2	46.08	2	F2:112594	20210611-09_2021021-OK160A_816_5.3.236.mgf	0	0	0	0	0	454	463			PEAKS DB
K.LADTNEADIR.F	Y	85.89	1116.5411	10	5.7	559.2787	2	19.91	2	F2:65906	20210611-09_2021021-OK160A_816_5.3.236.mgf		0	0	0	0	183	192			PEAKS DB
R.GVLNGEVEPYLK.S	Y	82.43	1316.6975	12	-0.3	659.3531	2	39.10	2	F2:99793	20210611-09_2021021-OK160A_816_5.3.236.mgf		0	0	0	0	360	371			PEAKS DB
K.LKDETDIVIAK.M	N	82.41	1243.7023	11	0.0	622.8558	2	25.53	2	F2:75618	20210611-09_2021021-OK160A_816_5.3.236.mgf		0	0	0	0	428	438			PEAKS DB
K.YKDEIVLFRPK.A	N	82.19	1406.7921	11	-0.6	469.9357	3	31.20	2	F2:85517	20210611-09_2021021-OK160A_816_5.3.236.mgf		0	0	0	0	207	217			PEAKS DB
K.VFFGVSNK.E	N	77.53	896.4756	8	2.5	449.2443	2	30.55	2	F2:84259	20210611-09_2021021-OK160A_816_5.3.236.mgf	0	0	0	0	0	304	311			PEAKS DB
K.YDELAEK.L	N	64.80	866.4021	7	4.6	434.2085	2	16.53	2	F2:60959	20210611-09_2021021-OK160A_816_5.3.236.mgf		0	0	0	0	421	427			PEAKS DB
K.QYEDFIGR.G	N	64.14	1026.4771	8	3.1	514.2452	2	30.02	2	F2:83542	20210611-09_2021021-OK160A_816_5.3.236.mgf		0	0	0	0	153	160			PEAKS DB
K.RLAPEYEK.A	N	61.68	1004.5291	8	1.7	503.2705	2	15.00	2	F2:59037	20210611-09_2021021-OK160A_816_5.3.236.mgf		0	0	0	0	68	75			PEAKS DB
K.FEDSVVK.F	Y	59.31	822.4123	7	2.4	412.2127	2	20.11	2	F2:66181	20210611-09_2021021-OK160A_816_5.3.236.mgf		0	0	0	0	223	229			PEAKS DB
K.TC(+57.02)QEYGVSGYPTLK.I	N	58.68	1601.7395	14	0.4	801.8740	2	32.78	2	F2:88088	20210611-09_2021021-OK160A_816_5.3.236.mgf		0	0	0	0	100	113		C2:Carbamidomethylation:1000.00	PEAKS DB
**total 18 peptides**																					

CebrOK035_J_344_Conoporin

back to list

| Protein Coverage | Supporting Peptides |

Protein Coverage:

67

1 MRVPFPALKT MVTVFLLLMG NMSPVVPRSS TPLHKVKVLA YK**VVTPGNSL NGVTLQDLAA TNYKVTCVIQ VENWTR**YALM   Carbamidomethylation (+57.02)

81 YPTVLTLGSP AVSSAPTAIL PTKK**EAFATH KPR**STATGVS GTVSWELQGA ERRFVLMWAA PYNFNHYSNW MGVGMTREGL

161 VDVAPGNTWF NQMYYGSSSG NLNFKR**KEFY YNTDPFIYSN EKFEAEGDMT NGHHAQVR**VI IRPTR**NNWED LADEIR**QNMV

Supporting Peptides:

Peptide	Uniq	-10lgP	Mass	Length	ppm	m/z	z	RT	Fraction	Scan	Source File	Area ebraeus	Area judaeus	#Feature	#Feature ebraeus	#Feature judaeus	Start	End	PTM	AScore	Found By
K.VVTPGNSLNGVTLQDLAATNYK.V	N	159.55	2274.1855	22	12.3	1138.1093	2	47.99	2	F2:115425	20210611-09_2021021-OK160A_816_5.3.236.mgf		0	0	0	0	43	64			PEAKS DB
K.FEAEGDMTNGHHAQVR.V	N	118.42	1797.7853	16	2.2	600.2678	3	18.47	2	F2:63606	20210611-09_2021021-OK160A_816_5.3.236.mgf		0	0	0	0	203	218			PEAKS DB
K.VTC(+57.02)VIQVENWTR.Y	N	117.26	1503.7504	12	0.3	752.8795	2	40.90	2	F2:103099	20210611-09_2021021-OK160A_816_5.3.236.mgf		0	0	0	0	65	76	Carbamidomethylation	C3:Carbamidomethylation:1000.00	PEAKS DB
K.EFYYNTDPFIYSNEK.F	N	116.83	1928.8468	15	1.6	965.4282	2	46.01	2	F2:112287	20210611-09_2021021-OK160A_816_5.3.236.mgf		0	0	0	0	188	202			PEAKS DB
K.FEAEGDM(+15.99)TNGHHAQVR.V	N	104.09	1813.7802	16	-0.5	605.5978	3	13.73	2	F2:57220	20210611-09_2021021-OK160A_816_5.3.236.mgf		0	0	0	0	203	218		M7:Oxidation (M):1000.00	PEAKS DB
R.NNWEDLADEIR.Q	N	99.52	1373.6211	11	0.4	687.8152	2	45.04	2	F2:110772	20210611-09_2021021-OK160A_816_5.3.236.mgf		0	0	0	0	226	236			PEAKS DB
R.KEFYYNTDPFIYSNEK.F	N	96.57	2056.9417	16	2.0	686.6530	3	40.01	2	F2:101239	20210611-09_2021021-OK160A_816_5.3.236.mgf		0	0	0	0	187	202			PEAKS DB
K.EAFATHKPR.S	Y	76.19	1055.5511	9	4.2	352.8577	3	10.56	2	F2:53584	20210611-09_2021021-OK160A_816_5.3.236.mgf		0	0	0	0	105	113			PEAKS DB
**total 8 peptides**																					

P_033D_033_Arginine

back to list

| Protein Coverage | Supporting Peptides |

Protein Coverage:

1 GEGEGSASAP AAAAAAEEPN EAEEMYKKLS DAESKSVLKK QLTKGVLDKL KAKKTEK**GAT LADCIRSGVE LPDSK**IGVYA

81 PDPEAYTVFA PLLDGVIKNA HGLPEKKQIN QPALDFGDLE K**VKLDDLDPD GKYVVSTSVS AGR**SLEGYAF LSLLTK**ENLE**

161 **EVEQK**VTEAL KTLSEDLEGT YQALADLDSD VSEQLQKEGK LFADSDKGVQ AAGGYKEWPR GRGLFHNADK **TLVAWVNQEE**

241 **HLR**VISKQAG GDVGAVYGRL VKALKALGEK LTFARDDK**LG YVTFSPVNLG TSLR**ATLTLN IPTAAQQANF QATLDKLHLQ

321 CEAAEEGAYN ISNKR**SVGVT EIQLITEVYN GAK**DLIKMEQ DLTWKPESLQ EMVDHVTKTK SCKSLMKKHL TKDVADRLKD

401 KKTTLGATLA DCIRSGSWNL DSGVGIYAAD QESYTMFADI LDPVIKDYHK LKAIDAIAHP ASDFGDPETL DLPDLDPENE

481 LIVSTRIRVG RSHK**EYGFPP VLTK**EDREAM EKKTMEALQS LTDELKGSYH PLTGMTKETQ DQLTK**DHFLF NDSDR**FLK**AA**

561 **GGYDDWPTGR GIFFNEAK**TF LVWVNEEDHL RIISMQSGGD LGAVYKRLIK AVKALEEKLT FARDDRLGYL TFCPSNLGTT

641 LRASVHIKIP K**LAATPEFK**E FCDKYNIQAR **GIHGEHTESV GGVYDISNK**R RLGLTEFEAI QEMRKGVEAV IQKEKELSGQ

Supporting Peptides:

Peptide	Uniq	-10lgP	Mass	Length	ppm	m/z	z	RT	Fraction	Scan	Source File	Area ebraeus	Area judaeus	#Feature	#Feature ebraeus	#Feature judaeus	Start	End	PTM	AScore	Found By
R.SVGVTEIQLITEVYNGAK.D	Y	119.69	1920.0204	18	6.5	961.0197	2	55.93	2	F2:127543	20210611-09_2021021-OK160A_816_5.3.236.mgf		0	0	0	0	336	353			PEAKS DB
R.GIHGEHTESVGGVYDISNK.R	Y	118.70	1997.9442	19	3.0	666.9879	3	24.40	2	F2:73534	20210611-09_2021021-OK160A_816_5.3.236.mgf		0	0	0	0	671	689			PEAKS DB
K.LGYVTFSPVNLGTSLR.A	Y	104.95	1722.9304	16	-1.8	862.4673	2	52.97	2	F2:123584	20210611-09_2021021-OK160A_816_5.3.236.mgf		0	0	0	0	279	294			PEAKS DB
K.TLVAWVNQEEHLR.V	Y	99.69	1593.8263	13	-0.3	797.9169	2	40.00	2	F2:101275	20210611-09_2021021-OK160A_816_5.3.236.mgf		0	0	0	0	231	243			PEAKS DB
K.VKLDDLDPDGK.Y	Y	99.02	1213.6190	11	1.9	607.8154	2	27.19	2	F2:78312	20210611-09_2021021-OK160A_816_5.3.236.mgf		0	0	0	0	122	132			PEAKS DB
K.DHFLFNDSDR.F	Y	92.27	1264.5472	10	2.9	633.2801	2	30.22	2	F2:83716	20210611-09_2021021-OK160A_816_5.3.236.mgf		0	0	0	0	546	555			PEAKS DB
K.AAGGYDDWPTGR.G	Y	85.17	1264.5472	12	3.5	633.2805	2	28.87	2	F2:81362	20210611-09_2021021-OK160A_816_5.3.236.mgf		0	0	0	0	559	570			PEAKS DB
K.YVVSTSVSAGR.S	Y	75.60	1124.5825	11	1.6	563.2971	2	23.49	2	F2:72026	20210611-09_2021021-OK160A_816_5.3.236.mgf		0	0	0	0	133	143			PEAKS DB
R.SGVELPDSK.I	Y	73.64	930.4658	9	2.9	466.2396	2	22.11	2	F2:69541	20210611-09_2021021-OK160A_816_5.3.236.mgf	0	0	0	0	0	67	75			PEAKS DB
K.EYGFPPVLTK.E	Y	69.49	1149.6069	10	-2.5	575.8069	2	42.58	2	F2:105841	20210611-09_2021021-OK160A_816_5.3.236.mgf		0	0	0	0	495	504			PEAKS DB
K.ENLEEVEQK.V	Y	67.27	1116.5298	9	1.9	559.2709	2	19.59	2	F2:65466	20210611-09_2021021-OK160A_816_5.3.236.mgf		0	0	0	0	157	165			PEAKS DB
R.GIFFNEAK.T	Y	64.60	924.4705	8	5.1	463.2429	2	33.34	2	F2:89231	20210611-09_2021021-OK160A_816_5.3.236.mgf		0	0	0	0	571	578			PEAKS DB
K.GATLADC(+57.02)IR.S	Y	61.97	975.4808	9	2.6	488.7469	2	25.57	2	F2:75526	20210611-09_2021021-OK160A_816_5.3.236.mgf		0	0	0	0	58	66		C7:Carbamidomethylation:1000.00	PEAKS DB
K.LAATPEFK.E	Y	57.78	875.4752	8	-0.6	438.7428	2	24.96	2	F2:74549	20210611-09_2021021-OK160A_816_5.3.236.mgf		0	0	0	0	652	659			PEAKS DB

total 14 peptides

CebrOK035_J_343_Prohormone-4a

back to list

| Protein Coverage | Supporting Peptides |

Protein Coverage:

1 NPVTNLAHPM NPAPYSPMNP VTNLAHPMNP ANPYSPMNPV TNLAHPMNPA NPYNPMNPVN SWYNPMNFQY PFHPPMQVAS

81 YYLTPPLGSS YLFPSNGYGH FHLAPTQAAA PLNLHMHFPY SYQAGGMYNP YMYPYQSSAL YQPYGYAAGV PGAMAWNQFG

161 GAAWNPYSAM GYGVYPMWPA QFAHMWPLHP YYQTVMPAQE GMCSYDRPFK CWGSATCITI EAICDKNFDC NNHADEDPRI

241 CVAGRRPSYG ILVSYLMLPN QQWMLQK**LFN NVDPYTAAHA LKLATSLGDL RWSLALTDEN INNTKTVLEA VENDNKGPLV**

321 **SLGMQASAWE SVK**FLMK**NLL STDFFLD**

Supporting Peptides:

Peptide	Uniq	-10lgP	Mass	Length	ppm	m/z	z	RT	Fraction	Scan	Source File	Area ebraeus	Area judaeus	#Feature	#Feature ebraeus	#Feature judaeus	Start	End	PTM	AScore	Found By
K.GPLVSLGMQASAWESVK.F	Y	138.35	1758.8975	17	2.1	880.4542	2	50.02	2	F2:118803	20210611-09_2021021-OK160A_816_5.3.236.mgf		0	0	0	0	317	333			PEAKS DB
K.GPLVSLGM(+15.99)QASAWESVK.F	Y	135.06	1774.8923	17	0.3	888.4500	2	45.86	2	F2:112235	20210611-09_2021021-OK160A_816_5.3.236.mgf		0	0	0	0	317	333		M8:Oxidation (M):1000.00	PEAKS DB
R.WSLALTDENINNTK.T	N	121.92	1617.7998	14	-1.3	809.9027	2	42.21	2	F2:105115	20210611-09_2021021-OK160A_816_5.3.236.mgf	0	0	0	0	0	292	305			PEAKS DB
K.LFNNVDPYTAAHALK.L	N	118.88	1672.8573	15	-0.1	837.4324	2	38.61	2	F2:99023	20210611-09_2021021-OK160A_816_5.3.236.mgf	0	0	0	0	0	268	282			PEAKS DB
K.TVLEAVENDNK.G	Y	105.21	1230.6091	11	1.9	616.3104	2	26.66	2	F2:77659	20210611-09_2021021-OK160A_816_5.3.236.mgf		0	0	0	0	306	316			PEAKS DB
K.TVLEAVENDNKGPLVSLGMQASAWESVK.F	Y	94.51	2971.4961	28	5.6	991.5074	3	54.88	2	F2:126206	20210611-09_2021021-OK160A_816_5.3.236.mgf		0	0	0	0	306	333			PEAKS DB
K.LATSLGDLR.W	N	77.79	944.5291	9	3.7	473.2715	2	31.22	2	F2:85544	20210611-09_2021021-OK160A_816_5.3.236.mgf	0	0	0	0	0	283	291			PEAKS DB
K.NLLSTDFFLD	Y	56.00	1183.5760	10	-1.0	592.7922	2	60.15	2	F2:132237	20210611-09_2021021-OK160A_816_5.3.236.mgf		0	0	0	0	338	347			PEAKS DB

total 8 peptides

OK214_J_305_Prohormone-4a

back to list

| Protein Coverage | Supporting Peptides |

Protein Coverage:

1 MNSFYPYSAA MMPFNSFRPP MHPISYLHPP MGYSFVYPAM NTMSPYNLMH PMYNPAHPMN HANPYSPMNP VTNLAHPMNP

81 ATPYNPMNPV NSWYNPMNFQ YPFHPPMQVA SYYLTPPLGS SYLFPSNGYG HFHLAPTQAA APLNLHMHFP YSYQAGGMYN

161 PYMYPYQSSA LYQPYGYAAG VPGAMAWNQF GGAAWNPYSA MGYGVYPMWP AQFAHMWPLH PYYQTVMPAQ EGMCSYDRPF

241 KCWGSATCIT IEAICDKNFD CNNHADEDPR ICVAGRRPSY GILVSYLMLP NQQWMLQK**LF NNVDPYTAAH ALKLATSLGD**

321 **LRWSLALTDE NINNTKTVLE AVENDNKGPL VSLGMQASAW ESVK**FLMK**NL LSTDFFLD**

Supporting Peptides:

Peptide	Uniq	-10lgP	Mass	Length	ppm	m/z	z	RT	Fraction	Scan	Source File	Area ebraeus	Area judaeus	#Feature	#Feature ebraeus	#Feature judaeus	Start	End	PTM	AScore	Found By
K.GPLVSLGMQASAWESVK.F	Y	138.35	1758.8975	17	2.1	880.4542	2	50.02	2	F2:118803	20210611-09_2021021-OK160A_816_5.3.236.mgf		0	0	0	0	348	364			PEAKS DB
K.GPLVSLGM(+15.99)QASAWESVK.F	Y	135.06	1774.8923	17	0.3	888.4500	2	45.86	2	F2:112235	20210611-09_2021021-OK160A_816_5.3.236.mgf		0	0	0	0	348	364		M8:Oxidation (M):1000.00	PEAKS DB
R.WSLALTDENINNTK.T	N	121.92	1617.7998	14	-1.3	809.9027	2	42.21	2	F2:105115	20210611-09_2021021-OK160A_816_5.3.236.mgf	0	0	0	0	0	323	336			PEAKS DB
K.LFNNVDPYTAAHALK.L	N	118.88	1672.8573	15	-0.1	837.4324	2	38.61	2	F2:99023	20210611-09_2021021-OK160A_816_5.3.236.mgf	0	0	0	0	0	299	313			PEAKS DB
K.TVLEAVENDNK.G	Y	105.21	1230.6091	11	1.9	616.3104	2	26.66	2	F2:77659	20210611-09_2021021-OK160A_816_5.3.236.mgf		0	0	0	0	337	347			PEAKS DB

total 8 peptides

Peptide	Uniq	-10lgP	Mass	Length	ppm	m/z	z	RT	Fraction	Scan	Source File	Area ebraeus	Area judaeus	#Feature	#Feature ebraeus	#Feature judaeus	Start	End	PTM	AScore	Found By
K.TVLEAVENDNKGPLVSLGMQASAWESVK.F	Y	94.51	2971.4961	28	5.6	991.5074	3	54.88	2	F2:126206	20210611-09_2021021-OK160A_816_5.3.236.mgf		0	0	0	0	337	364			PEAKS DB
K.LATSLGDLR.W	N	77.79	944.5291	9	3.7	473.2715	2	31.22	2	F2:85544	20210611-09_2021021-OK160A_816_5.3.236.mgf	0	0	0	0	0	314	322			PEAKS DB
K.NLLSTDFFLD	Y	56.00	1183.5760	10	-1.0	592.7922	2	60.15	2	F2:132237	20210611-09_2021021-OK160A_816_5.3.236.mgf		0	0	0	0	369	378			PEAKS DB
total 8 peptides																					

OK212_J_281_Prohormone-4a

back to list

| Protein Coverage | Supporting Peptides |

Protein Coverage:

1 MNSFYPYSAA MMPFNSFRPP MHPISYLHPP MGYSFVYPAM NTMSPYNLMH PMYNPAHPMN HANPYSPMNP VTNLAHPMNP
81 ANPYNPMNPV NSWYNPMNFQ YPFHPPMQVA SYYLTPPLGS SYLFPSNGYG HFHLAPTQAA APLNLHMHFP YSYQAGGMYN
161 PYMYPYQSSA LYQPYGYAAG VPGAMAWNQF GGAAWNPYSA MGYGVYPMWP AQFAHMWPLH PYYQTVMPAQ EGMCSYDRPF
241 KCWGSATCIT IEAICDKNFD CNNHADEDPR ICVAGRRPSY GILVSYLMLP NQQWMLQK**LF NNVDPYTAAH ALKLATSLGD**
321 **LRWSLALTDE NINNTKTVLE AVENDNKGPL VSLGMQASAW ESVK**FLMK**NL LSTDFFLD**

Supporting Peptides:

Peptide	Uniq	-10lgP	Mass	Length	ppm	m/z	z	RT	Fraction	Scan	Source File	Area ebraeus	Area judaeus	#Feature	#Feature ebraeus	#Feature judaeus	Start	End	PTM	AScore	Found By
K.GPLVSLGMQASAWESVK.F	Y	138.35	1758.8975	17	2.1	880.4542	2	50.02	2	F2:118803	20210611-09_2021021-OK160A_816_5.3.236.mgf		0	0	0	0	348	364			PEAKS DB
K.GPLVSLGM(+15.99)QASAWESVK.F	Y	135.06	1774.8923	17	0.3	888.4500	2	45.86	2	F2:112235	20210611-09_2021021-OK160A_816_5.3.236.mgf		0	0	0	0	348	364		M8:Oxidation (M):1000.00	PEAKS DB
R.WSLALTDENINNTK.T	N	121.92	1617.7998	14	-1.3	809.9027	2	42.21	2	F2:105115	20210611-09_2021021-OK160A_816_5.3.236.mgf	0	0	0	0	0	323	336			PEAKS DB
K.LFNNVDPYTAAHALK.L	N	118.88	1672.8573	15	-0.1	837.4324	2	38.61	2	F2:99023	20210611-09_2021021-OK160A_816_5.3.236.mgf	0	0	0	0	0	299	313			PEAKS DB
K.TVLEAVENDNK.G	Y	105.21	1230.6091	11	1.9	616.3104	2	26.66	2	F2:77659	20210611-09_2021021-OK160A_816_5.3.236.mgf		0	0	0	0	337	347			PEAKS DB
K.TVLEAVENDNKGPLVSLGMQASAWESVK.F	Y	94.51	2971.4961	28	5.6	991.5074	3	54.88	2	F2:126206	20210611-09_2021021-OK160A_816_5.3.236.mgf		0	0	0	0	337	364			PEAKS DB
K.LATSLGDLR.W	N	77.79	944.5291	9	3.7	473.2715	2	31.22	2	F2:85544	20210611-09_2021021-OK160A_816_5.3.236.mgf	0	0	0	0	0	314	322			PEAKS DB
K.NLLSTDFFLD	Y	56.00	1183.5760	10	-1.0	592.7922	2	60.15	2	F2:132237	20210611-09_2021021-OK160A_816_5.3.236.mgf		0	0	0	0	369	378			PEAKS DB
total 8 peptides																					

OK212_J_284_Prohormone-4a

back to list

| Protein Coverage | Supporting Peptides |

Protein Coverage:

1 MNSFYPYSAA MMPFNSFRPP MHPISYLHPP MGYSFVYPAM NTMSPYNLMH PMYNPAHPMN HANPHSPLNP VTNLAHPMNP
81 ANPYSPINPV TNLAHPMNPA NPYNPMNPVN SWYNPMNFQY PFHPPMQVAS YYLTPPLGSS YLFPSNGYGH FHLAPTQAAA
161 PLNLHMHFPY SYQAGGMYNP YMYPYQSSAL YQPYGYAAGV PGAMAWNQFG GAAWNPYSAM GYGVYPMWPA QFAHMWPLHP
241 YYQTVMPAQE GMCSYDRPFK CWGSATCITI EAICDKNFDC NNHADEDPRI CVAGRRPSYG ILVSYLMLPN QQWMLQK**LFN**
321 **NVDPYTAAHA LKLATSLGDL RWSLALTDEN INNTKTVLEA VENDNKGPLV SLGMQASAWE SVK**FLMK**NLL STDFFLD**

Supporting Peptides:

Peptide	Uniq	-10lgP	Mass	Length	ppm	m/z	z	RT	Fraction	Scan	Source File	Area ebraeus	Area judaeus	#Feature	#Feature ebraeus	#Feature judaeus	Start	End	PTM	AScore	Found By
K.GPLVSLGMQASAWESVK.F	Y	138.35	1758.8975	17	2.1	880.4542	2	50.02	2	F2:118803	20210611-09_2021021-OK160A_816_5.3.236.mgf		0	0	0	0	367	383			PEAKS DB
K.GPLVSLGM(+15.99)QASAWESVK.F	Y	135.06	1774.8923	17	0.3	888.4500	2	45.86	2	F2:112235	20210611-09_2021021-OK160A_816_5.3.236.mgf		0	0	0	0	367	383		M8:Oxidation (M):1000.00	PEAKS DB
R.WSLALTDENINNTK.T	N	121.92	1617.7998	14	-1.3	809.9027	2	42.21	2	F2:105115	20210611-09_2021021-OK160A_816_5.3.236.mgf	0	0	0	0	0	342	355			PEAKS DB
K.LFNNVDPYTAAHALK.L	N	118.88	1672.8573	15	-0.1	837.4324	2	38.61	2	F2:99023	20210611-09_2021021-OK160A_816_5.3.236.mgf	0	0	0	0	0	318	332			PEAKS DB
K.TVLEAVENDNK.G	Y	105.21	1230.6091	11	1.9	616.3104	2	26.66	2	F2:77659	20210611-09_2021021-OK160A_816_5.3.236.mgf		0	0	0	0	356	366			PEAKS DB
K.TVLEAVENDNKGPLVSLGMQASAWESVK.F	Y	94.51	2971.4961	28	5.6	991.5074	3	54.88	2	F2:126206	20210611-09_2021021-OK160A_816_5.3.236.mgf		0	0	0	0	356	383			PEAKS DB
K.LATSLGDLR.W	N	77.79	944.5291	9	3.7	473.2715	2	31.22	2	F2:85544	20210611-09_2021021-OK160A_816_5.3.236.mgf	0	0	0	0	0	333	341			PEAKS DB
K.NLLSTDFFLD	Y	56.00	1183.5760	10	-1.0	592.7922	2	60.15	2	F2:132237	20210611-09_2021021-OK160A_816_5.3.236.mgf		0	0	0	0	388	397			PEAKS DB
total 8 peptides																					

OK212_J_282_Prohormone-4a

back to list

| Protein Coverage | Supporting Peptides |

Protein Coverage:

1 MNSFYPYSAA MMPFNSFRPP MHPISYLHPP MGYSFVYPAM NTMSPYNLMH PMYNPAHPMN HANPYSPMNP VTNLAHPMNP
81 ANSHSPLNPV TNLAHPMNPA NPYSPMNPVT NLAHPMNPAN PYNPMNPVNS WYNPMNFQYP FHPPMQVASY YLTPPLGSSY
161 LFPSNGYGHF HLAPTQAAAP LNLHMHFPYS YQAGGMYNPY MYPYQSSALY QPYGYAAGVP GAMAWNQFGG AAWNPYSAMG
241 YGVYPMWPAQ FAHMWPLHPY YQTVMPAQEG MCSYDRPFKC WGSATCITIE AICDKNFDCN NHADEDPRIC VAGRRPSYGI
321 LVSYLMLPNQ QWMLQK**LFNN VDPYTAAHAL KLATSLGDLR WSLALTDENI NNTKTVLEAV ENDNKGPLVS LGMQASAWES**
401 **VK**FLMK**NLLS TDFFLD**

Supporting Peptides:

Peptide	Uniq	-10lgP	Mass	Length	ppm	m/z	z	RT	Fraction	Scan	Source File	Area ebraeus	Area judaeus	#Feature	#Feature ebraeus	#Feature judaeus	Start	End	PTM	AScore	Found By
K.GPLVSLGMQASAWESVK.F	Y	138.35	1758.8975	17	2.1	880.4542	2	50.02	2	F2:118803	20210611-09_2021021-OK160A_816_5.3.236.mgf		0	0	0	0	386	402			PEAKS DB
K.GPLVSLGM(+15.99)QASAWESVK.F	Y	135.06	1774.8923	17	0.3	888.4500	2	45.86	2	F2:112235	20210611-09_2021021-OK160A_816_5.3.236.mgf		0	0	0	0	386	402		M8:Oxidation (M):1000.00	PEAKS DB
R.WSLALTDENINNTK.T	N	121.92	1617.7998	14	-1.3	809.9027	2	42.21	2	F2:105115	20210611-09_2021021-OK160A_816_5.3.236.mgf	0	0	0	0	0	361	374			PEAKS DB
K.LFNNVDPYTAAHALK.L	N	118.88	1672.8573	15	-0.1	837.4324	2	38.61	2	F2:99023	20210611-09_2021021-OK160A_816_5.3.236.mgf	0	0	0	0	0	337	351			PEAKS DB
K.TVLEAVENDNK.G	Y	105.21	1230.6091	11	1.9	616.3104	2	26.66	2	F2:77659	20210611-09_2021021-OK160A_816_5.3.236.mgf		0	0	0	0	375	385			PEAKS DB
K.TVLEAVENDNKGPLVSLGMQASAWESVK.F	Y	94.51	2971.4961	28	5.6	991.5074	3	54.88	2	F2:126206	20210611-09_2021021-OK160A_816_5.3.236.mgf		0	0	0	0	375	402			PEAKS DB
K.LATSLGDLR.W	N	77.79	944.5291	9	3.7	473.2715	2	31.22	2	F2:85544	20210611-09_2021021-OK160A_816_5.3.236.mgf	0	0	0	0	0	352	360			PEAKS DB
K.NLLSTDFFLD	Y	56.00	1183.5760	10	-1.0	592.7922	2	60.15	2	F2:132237	20210611-09_2021021-OK160A_816_5.3.236.mgf		0	0	0	0	407	416			PEAKS DB
total 8 peptides																					

CebrOK035_J_340_Prohormone-4a

back to list

| Protein Coverage | Supporting Peptides |

Protein Coverage:

1 MNSFYPYSAA MMPFNSFRPP MHPISYLHPP MGYSFVYPAM NTMSPYNLMH PMYNPAHPMN HANPYSPMNP VTNLAHPMNP
81 ANPHSPLNPV TNLAHPMNPA PMNPANPYSP MNPVTNLAHP MNPALAHPMN PANPYNPMNP VNSWYNPMNF QYPFHPPMQV
161 ASYYLTPPLG SSYLFPSNGY GHFHLAPTQA AAPLNLHMHF PYSYQAGGMY NPYMYPYQSS ALYQPYGYAA GVPGAMAWNQ
241 FGGAAWNPYS AMGYGVYPMW PAQFAHMWPL HPYYQTVMPA QEGMCSYDRP FKCWGSATCI TIEAICDKNF DCNNHADEDP
321 RICVAGRRPS YGILVSYLML PNQQWMLQK**L FNNVDPYTAA HALKLATSLG DLRWSLALTD ENINNTKTVL EAVENDNKGP**

401 **LVSLGMQASA WESVK**FLMK**N LLSTDFFLD**

Supporting Peptides:

Peptide	Uniq	-10lgP	Mass	Length	ppm	m/z	z	RT	Fraction	Scan	Source File	Area ebraeus	Area judaeus	#Feature	#Feature ebraeus	#Feature judaeus	Start	End	PTM	AScore	Found By
K.GPLVSLGMQASAWESVK.F	Y	138.35	1758.8975	17	2.1	880.4542	2	50.02	2	F2:118803	20210611-09_2021021-OK160A_816_5.3.236.mgf		0	0	0	0	399	415			PEAKS DB
K.GPLVSLGM(+15.99)QASAWESVK.F	Y	135.06	1774.8923	17	0.3	888.4500	2	45.86	2	F2:112235	20210611-09_2021021-OK160A_816_5.3.236.mgf		0	0	0	0	399	415		M8:Oxidation (M):1000.00	PEAKS DB
R.WSLALTDENINNTK.T	N	121.92	1617.7998	14	-1.3	809.9027	2	42.21	2	F2:105115	20210611-09_2021021-OK160A_816_5.3.236.mgf	0	0	0	0	0	374	387			PEAKS DB
K.LFNNVDPYTAAHALK.L	N	118.88	1672.8573	15	-0.1	837.4324	2	38.61	2	F2:99023	20210611-09_2021021-OK160A_816_5.3.236.mgf	0	0	0	0	0	350	364			PEAKS DB
K.TVLEAVENDNK.G	Y	105.21	1230.6091	11	1.9	616.3104	2	26.66	2	F2:77659	20210611-09_2021021-OK160A_816_5.3.236.mgf		0	0	0	0	388	398			PEAKS DB
K.TVLEAVENDNKGPLVSLGMQASAWESVK.F	Y	94.51	2971.4961	28	5.6	991.5074	3	54.88	2	F2:126206	20210611-09_2021021-OK160A_816_5.3.236.mgf		0	0	0	0	388	415			PEAKS DB
K.LATSLGDLR.W	N	77.79	944.5291	9	3.7	473.2715	2	31.22	2	F2:85544	20210611-09_2021021-OK160A_816_5.3.236.mgf	0	0	0	0	0	365	373			PEAKS DB
K.NLLSTDFFLD	Y	56.00	1183.5760	10	-1.0	592.7922	2	60.15	2	F2:132237	20210611-09_2021021-OK160A_816_5.3.236.mgf		0	0	0	0	420	429			PEAKS DB
**total 8 peptides**																					

OK212_J_283_Prohormone-4a

back to list

| Protein Coverage | Supporting Peptides |

Protein Coverage:

1 MNSFYPYSAA MMPFNSFRPP MHPISYLHPP MGYSFVYPAM NTMSPYNLMH PMYNPAHPMN HANPHSPLNP VTNLAHPMNP
81 ANPYSPMNPV TNLAHPMNPA NSHSPLNPVT NLAHPMNPAN PYSPMNPVTN LAHPMNPANP YNPMNPVNSW YNPMNFQYPF
161 HPPMQVASYY LTPPLGSSYL FPSNGYGHFH LAPTQAAAPL NLHMHFPYSY QAGGMYNPYM YPYQSSALYQ PYGYAAGVPG
241 AMAWNQFGGA AWNPYSAMGY GVYPMWPAQF AHMWPLHPYY QTVMPAQEGM CSYDRPFKCW GSATCITIEA ICDKNFDCNN
321 HADEDPRICV AGRRPSYGIL VSYLMLPNQQ WMLQK**LFNNV DPYTAAHALK LATSLGDLRW SLALTDENIN NTKTVLEAVE**

401 **NDNKGPLVSL GMQASAWESV K**FLMK**NLLST DFFLD**

Supporting Peptides:

Peptide	Uniq	-10lgP	Mass	Length	ppm	m/z	z	RT	Fraction	Scan	Source File	Area ebraeus	Area judaeus	#Feature	#Feature ebraeus	#Feature judaeus	Start	End	PTM	AScore	Found By
K.GPLVSLGMQASAWESVK.F	Y	138.35	1758.8975	17	2.1	880.4542	2	50.02	2	F2:118803	20210611-09_2021021-OK160A_816_5.3.236.mgf		0	0	0	0	405	421			PEAKS DB
K.GPLVSLGM(+15.99)QASAWESVK.F	Y	135.06	1774.8923	17	0.3	888.4500	2	45.86	2	F2:112235	20210611-09_2021021-OK160A_816_5.3.236.mgf		0	0	0	0	405	421		M8:Oxidation (M):1000.00	PEAKS DB
R.WSLALTDENINNTK.T	N	121.92	1617.7998	14	-1.3	809.9027	2	42.21	2	F2:105115	20210611-09_2021021-OK160A_816_5.3.236.mgf	0	0	0	0	0	380	393			PEAKS DB
K.LFNNVDPYTAAHALK.L	N	118.88	1672.8573	15	-0.1	837.4324	2	38.61	2	F2:99023	20210611-09_2021021-OK160A_816_5.3.236.mgf	0	0	0	0	0	356	370			PEAKS DB
K.TVLEAVENDNK.G	Y	105.21	1230.6091	11	1.9	616.3104	2	26.66	2	F2:77659	20210611-09_2021021-OK160A_816_5.3.236.mgf		0	0	0	0	394	404			PEAKS DB
K.TVLEAVENDNKGPLVSLGMQASAWESVK.F	Y	94.51	2971.4961	28	5.6	991.5074	3	54.88	2	F2:126206	20210611-09_2021021-OK160A_816_5.3.236.mgf		0	0	0	0	394	421			PEAKS DB
K.LATSLGDLR.W	N	77.79	944.5291	9	3.7	473.2715	2	31.22	2	F2:85544	20210611-09_2021021-OK160A_816_5.3.236.mgf	0	0	0	0	0	371	379			PEAKS DB
K.NLLSTDFFLD	Y	56.00	1183.5760	10	-1.0	592.7922	2	60.15	2	F2:132237	20210611-09_2021021-OK160A_816_5.3.236.mgf		0	0	0	0	426	435			PEAKS DB
**total 8 peptides**																					

CebrOK033_J_239_Prohormone-4a

back to list

| Protein Coverage | Supporting Peptides |

Protein Coverage:

1 MNSFYPYSAA MMPFNSFRPP MHPISYLHPP MGYSFVYPAM NTMSPYNLMH PMYNPAHPMN HANPYSPMNP VTNLAHPMNP
81 ANPHSPLNPV TNLAHPMNPA NPYSPMNPVT NLAHPMNPAN PYSPMNPVTN LAHPMNPATP YNPMNPVNSW YNPMNFQYPF
161 HPPMQVASYY LTPPLGSSYL FPSNGYGHFH LAPTQAAAPL NLHMHFPYSY QAGGMYNPYM YPYQSSALYQ PYGYAAGVPG
241 AMAWNQFGGA AWNPYSAMGY GVYPMWPAQF AHMWPLHPYY QTVMPAQEGM CSYDRPFKCW GSATCITIEA ICDKNFDCNN
321 HADEDPRICV AGRRPSYGIL VSYLMLPNQQ WMLQK**LFNNV DPYTAAHALK LATSLGDLRW SLALTDENIN NTKTVLEAVE**

401 **NDNKGPLVSL GMQASAWESV K**FLMK**NLLST DFFLD**

Supporting Peptides:

Peptide	Uniq	-10lgP	Mass	Length	ppm	m/z	z	RT	Fraction	Scan	Source File	Area ebraeus	Area judaeus	#Feature	#Feature ebraeus	#Feature judaeus	Start	End	PTM	AScore	Found By
K.GPLVSLGMQASAWESVK.F	Y	138.35	1758.8975	17	2.1	880.4542	2	50.02	2	F2:118803	20210611-09_2021021-OK160A_816_5.3.236.mgf		0	0	0	0	405	421			PEAKS DB
K.GPLVSLGM(+15.99)QASAWESVK.F	Y	135.06	1774.8923	17	0.3	888.4500	2	45.86	2	F2:112235	20210611-09_2021021-OK160A_816_5.3.236.mgf		0	0	0	0	405	421		M8:Oxidation (M):1000.00	PEAKS DB
R.WSLALTDENINNTK.T	N	121.92	1617.7998	14	-1.3	809.9027	2	42.21	2	F2:105115	20210611-09_2021021-OK160A_816_5.3.236.mgf	0	0	0	0	0	380	393			PEAKS DB
K.LFNNVDPYTAAHALK.L	N	118.88	1672.8573	15	-0.1	837.4324	2	38.61	2	F2:99023	20210611-09_2021021-OK160A_816_5.3.236.mgf	0	0	0	0	0	356	370			PEAKS DB
K.TVLEAVENDNK.G	Y	105.21	1230.6091	11	1.9	616.3104	2	26.66	2	F2:77659	20210611-09_2021021-OK160A_816_5.3.236.mgf		0	0	0	0	394	404			PEAKS DB
K.TVLEAVENDNKGPLVSLGMQASAWESVK.F	Y	94.51	2971.4961	28	5.6	991.5074	3	54.88	2	F2:126206	20210611-09_2021021-OK160A_816_5.3.236.mgf		0	0	0	0	394	421			PEAKS DB
K.LATSLGDLR.W	N	77.79	944.5291	9	3.7	473.2715	2	31.22	2	F2:85544	20210611-09_2021021-OK160A_816_5.3.236.mgf	0	0	0	0	0	371	379			PEAKS DB
K.NLLSTDFFLD	Y	56.00	1183.5760	10	-1.0	592.7922	2	60.15	2	F2:132237	20210611-09_2021021-OK160A_816_5.3.236.mgf		0	0	0	0	426	435			PEAKS DB
**total 8 peptides**																					

OK214_J_306_Prohormone-4a

back to list

| Protein Coverage | Supporting Peptides |

Protein Coverage:

1 MNSFYPYSAA MMPFNSFRPP MHPISYLHPP MGYSFVYPAM NTMSPYNLMH PMYNPAHPMN HANPYSPMNP VTNLAHPMNP
81 ANPHSPLNPV TNLAHPMNPA NPYSPMNPVT NLAHPMNPAN PHSPLNPVTN LAHPMNPANP YSPMNPVTNL AHPMNPATPY
161 NPMNPVNSWY NPMNFQYPFH PPMQVASYYL TPPLGSSYLF PSNGYGHFHL APTQAAAPLN LHMHFPYSYQ AGGMYNPYMY
241 PYQSSALYQP YGYAAGVPGA MAWNQFGGAA WNPYSAMGYG VYPMWPAQFA HMWPLHPYYQ TVMPAQEGMC SYDRPFKCWG
321 SATCITIEAI CDKNFDCNNH ADEDPRICVA GRRPSYGILV SYLMLPNQQW MLQK**LFNNVD PYTAAHALKL ATSLGDLRWS**
401 **LALTDENINN TKTVLEAVEN DNKGPLVSLG MQASAWESVK** FLMK**NLLSTD FFLD**

Supporting Peptides:

Peptide	Uniq	-10lgP	Mass	Length	ppm	m/z	z	RT	Fraction	Scan	Source File	Area ebraeus	Area judaeus	#Feature	#Feature ebraeus	#Feature judaeus	Start	End	PTM	AScore	Found By
K.GPLVSLGMQASAWESVK.F	Y	138.35	1758.8975	17	2.1	880.4542	2	50.02	2	F2:118803	20210611-09_2021021-OK160A_816_5.3.236.mgf		0	0	0	0	424	440			PEAKS DB
K.GPLVSLGM(+15.99)QASAWESVK.F	Y	135.06	1774.8923	17	0.3	888.4500	2	45.86	2	F2:112235	20210611-09_2021021-OK160A_816_5.3.236.mgf		0	0	0	0	424	440		M8:Oxidation (M):1000.00	PEAKS DB
R.WSLALTDENINNTK.T	N	121.92	1617.7998	14	-1.3	809.9027	2	42.21	2	F2:105115	20210611-09_2021021-OK160A_816_5.3.236.mgf	0	0	0	0	0	399	412			PEAKS DB
K.LFNNVDPYTAAHALK.L	N	118.88	1672.8573	15	-0.1	837.4324	2	38.61	2	F2:99023	20210611-09_2021021-OK160A_816_5.3.236.mgf	0	0	0	0	0	375	389			PEAKS DB
K.TVLEAVENDNK.G	Y	105.21	1230.6091	11	1.9	616.3104	2	26.66	2	F2:77659	20210611-09_2021021-OK160A_816_5.3.236.mgf		0	0	0	0	413	423			PEAKS DB
K.TVLEAVENDNKGPLVSLGMQASAWESVK.F	Y	94.51	2971.4961	28	5.6	991.5074	3	54.88	2	F2:126206	20210611-09_2021021-OK160A_816_5.3.236.mgf		0	0	0	0	413	440			PEAKS DB
K.LATSLGDLR.W	N	77.79	944.5291	9	3.7	473.2715	2	31.22	2	F2:85544	20210611-09_2021021-OK160A_816_5.3.236.mgf	0	0	0	0	0	390	398			PEAKS DB
K.NLLSTDFFLD	Y	56.00	1183.5760	10	-1.0	592.7922	2	60.15	2	F2:132237	20210611-09_2021021-OK160A_816_5.3.236.mgf		0	0	0	0	445	454			PEAKS DB
**total 8 peptides**																					

OK005_J_296_Prohormone-4a

back to list

| Protein Coverage | Supporting Peptides |

Protein Coverage:

1 MNSFYPYSAA MMPFNSFRPP MHPISYLHPP MGYSFVYPAM NTMSPYNLMH PMYNPAHPMN HANPYSPMNP VTNLAHPMNP
81 ANPHSPLNPV TNLAHPMNPA NPYSPMNPVT NLAHPMNPAN PHSPLNPVTN LAHPMNPANP YSPMNPVTNL AHPMNPANPY
161 NPMNPVNSWY NPMNFQYPFH PPMQVASYYL TPPLGSSYLF PSNGYGHFHL APTQAAAPLN LHMHFPYSYQ AGGMYNPYMY
241 PYQSSALYQP YGYAAGVPGA MAWNQFGGAA WNPYSAMGYG VYPMWPAQFA HMWPLHPYYQ TVMPAQEGMC SYDRPFKCWG
321 SATCITIEAI CDKNFDCNNH ADEDPRICVA GRRPSYGILV SYLMLPNQQW MLQK**LFNNVD PYTAAHALKL ATSLGDLRWS**
401 **LALTDENINN TKTVLEAVEN DNKGPLVSLG MQASAWESVK** FLMK**NLLSTD FFLD**

Supporting Peptides:

Peptide	Uniq	-10lgP	Mass	Length	ppm	m/z	z	RT	Fraction	Scan	Source File	Area ebraeus	Area judaeus	#Feature	#Feature ebraeus	#Feature judaeus	Start	End	PTM	AScore	Found By
K.GPLVSLGMQASAWESVK.F	Y	138.35	1758.8975	17	2.1	880.4542	2	50.02	2	F2:118803	20210611-09_2021021-OK160A_816_5.3.236.mgf		0	0	0	0	424	440			PEAKS DB
K.GPLVSLGM(+15.99)QASAWESVK.F	Y	135.06	1774.8923	17	0.3	888.4500	2	45.86	2	F2:112235	20210611-09_2021021-OK160A_816_5.3.236.mgf		0	0	0	0	424	440		M8:Oxidation (M):1000.00	PEAKS DB
R.WSLALTDENINNTK.T	N	121.92	1617.7998	14	-1.3	809.9027	2	42.21	2	F2:105115	20210611-09_2021021-OK160A_816_5.3.236.mgf	0	0	0	0	0	399	412			PEAKS DB
K.LFNNVDPYTAAHALK.L	N	118.88	1672.8573	15	-0.1	837.4324	2	38.61	2	F2:99023	20210611-09_2021021-OK160A_816_5.3.236.mgf	0	0	0	0	0	375	389			PEAKS DB
K.TVLEAVENDNK.G	Y	105.21	1230.6091	11	1.9	616.3104	2	26.66	2	F2:77659	20210611-09_2021021-OK160A_816_5.3.236.mgf		0	0	0	0	413	423			PEAKS DB
K.TVLEAVENDNKGPLVSLGMQASAWESVK.F	Y	94.51	2971.4961	28	5.6	991.5074	3	54.88	2	F2:126206	20210611-09_2021021-OK160A_816_5.3.236.mgf		0	0	0	0	413	440			PEAKS DB
K.LATSLGDLR.W	N	77.79	944.5291	9	3.7	473.2715	2	31.22	2	F2:85544	20210611-09_2021021-OK160A_816_5.3.236.mgf	0	0	0	0	0	390	398			PEAKS DB
K.NLLSTDFFLD	Y	56.00	1183.5760	10	-1.0	592.7922	2	60.15	2	F2:132237	20210611-09_2021021-OK160A_816_5.3.236.mgf		0	0	0	0	445	454			PEAKS DB
**total 8 peptides**																					

CebrOK035_J_339_Prohormone-4a

back to list

| Protein Coverage | Supporting Peptides |

Protein Coverage:

1 MNSFYPYSAA MMPFNSFRPP MHPISYLHPP MGYSFVYPAM NTMSPYNLMH PMYNPAHPMN HANPHSPLNP VTNLAHPMNP
81 ANPYSPINPV TNLAHPMNPA NPHSPLNPVT NLAHPMNPAN PYSPMNPVTN LAHPMNPANP YSPMNPVTNL AHPMNPANPY
161 NPMNPVNSWY NPMNFQYPFH PPMQVASYYL TPPLGSSYLF PSNGYGHFHL APTQAAAPLN LHMHFPYSYQ AGGMYNPYMY
241 PYQSSALYQP YGYAAGVPGA MAWNQFGGAA WNPYSAMGYG VYPMWPAQFA HMWPLHPYYQ TVMPAQEGMC SYDRPFKCWG
321 SATCITIEAI CDKNFDCNNH ADEDPRICVA GRRPSYGILV SYLMLPNQQW MLQK**LFNNVD PYTAAHALKL ATSLGDLRWS**
401 **LALTDENINN TKTVLEAVEN DNKGPLVSLG MQASAWESVK** FLMK**NLLSTD FFLD**

Supporting Peptides:

Peptide	Uniq	-10lgP	Mass	Length	ppm	m/z	z	RT	Fraction	Scan	Source File	Area ebraeus	Area judaeus	#Feature	#Feature ebraeus	#Feature judaeus	Start	End	PTM	AScore	Found By
K.GPLVSLGMQASAWESVK.F	Y	138.35	1758.8975	17	2.1	880.4542	2	50.02	2	F2:118803	20210611-09_2021021-OK160A_816_5.3.236.mgf		0	0	0	0	424	440			PEAKS DB
K.GPLVSLGM(+15.99)QASAWESVK.F	Y	135.06	1774.8923	17	0.3	888.4500	2	45.86	2	F2:112235	20210611-09_2021021-OK160A_816_5.3.236.mgf		0	0	0	0	424	440		M8:Oxidation (M):1000.00	PEAKS DB
R.WSLALTDENINNTK.T	N	121.92	1617.7998	14	-1.3	809.9027	2	42.21	2	F2:105115	20210611-09_2021021-OK160A_816_5.3.236.mgf	0	0	0	0	0	399	412			PEAKS DB
K.LFNNVDPYTAAHALK.L	N	118.88	1672.8573	15	-0.1	837.4324	2	38.61	2	F2:99023	20210611-09_2021021-OK160A_816_5.3.236.mgf	0	0	0	0	0	375	389			PEAKS DB
K.TVLEAVENDNK.G	Y	105.21	1230.6091	11	1.9	616.3104	2	26.66	2	F2:77659	20210611-09_2021021-OK160A_816_5.3.236.mgf		0	0	0	0	413	423			PEAKS DB
K.TVLEAVENDNKGPLVSLGMQASAWESVK.F	Y	94.51	2971.4961	28	5.6	991.5074	3	54.88	2	F2:126206	20210611-09_2021021-OK160A_816_5.3.236.mgf		0	0	0	0	413	440			PEAKS DB
K.LATSLGDLR.W	N	77.79	944.5291	9	3.7	473.2715	2	31.22	2	F2:85544	20210611-09_2021021-OK160A_816_5.3.236.mgf	0	0	0	0	0	390	398			PEAKS DB
K.NLLSTDFFLD	Y	56.00	1183.5760	10	-1.0	592.7922	2	60.15	2	F2:132237	20210611-09_2021021-OK160A_816_5.3.236.mgf		0	0	0	0	445	454			PEAKS DB
**total 8 peptides**																					

CebrOK035_J_341_Prohormone-4a

back to list

| Protein Coverage | Supporting Peptides |

Protein Coverage:

1 MNSFYPYSAA MMPFNSFRPP MHPISYLHPP MGYSFVYPAM NTMSPYNLMH PMYNPAHPMN HANPYSPMPP IVLYSYGRFI
81 LQSNPVTNLA HPMNPANPHS PLNPVTNLAH PMNPANPYSP MNPVTNLAHP MNPANPYSPM NPVTNLAHPM NPANPHSPLN
161 PVTNLAHPMN PANPYSPINP VTNLAHPMNP ANPYNPMNPV NSWYNPMNFQ YPFHPPMQVA SYYLTPPLGS SYLFPSNGYG
241 HFHLAPTQAA APLNLHMHFP YSYQAGGMYN PYMYPYQSSA LYQPYGYAAG VPGAMAWNQF GGAAWNPYSA MGYGVYPMWP
321 AQFAHMWPLH PYYQTVMPAQ EGMCSYDRPF KCWGSATCIT IEAICDKNFD CNNHADEDPR ICVAGRRPSY GILVSYLMLP
401 NQQWMLQK**LF NNVDPYTAAH ALKLATSLGD LRWSLALTDE NINNTKTVLE AVENDNKGPL VSLGMQASAW ESVK**FLMK**NL**

481 **LSTDFFLD**

Supporting Peptides:

Peptide	Uniq	-10lgP	Mass	Length	ppm	m/z	z	RT	Fraction	Scan	Source File	Area ebraeus	Area judaeus	#Feature	#Feature ebraeus	#Feature judaeus	Start	End	PTM	AScore	Found By
K.GPLVSLGMQASAWESVK.F	Y	138.35	1758.8975	17	2.1	880.4542	2	50.02	2	F2:118803	20210611-09_2021021-OK160A_816_5.3.236.mgf		0	0	0	0	458	474			PEAKS DB
K.GPLVSLGM(+15.99)QASAWESVK.F	Y	135.06	1774.8923	17	0.3	888.4500	2	45.86	2	F2:112235	20210611-09_2021021-OK160A_816_5.3.236.mgf		0	0	0	0	458	474		M8:Oxidation (M):1000.00	PEAKS DB
R.WSLALTDENINNTK.T	N	121.92	1617.7998	14	-1.3	809.9027	2	42.21	2	F2:105115	20210611-09_2021021-OK160A_816_5.3.236.mgf	0	0	0	0	0	433	446			PEAKS DB
K.LFNNVDPYTAAHALK.L	N	118.88	1672.8573	15	-0.1	837.4324	2	38.61	2	F2:99023	20210611-09_2021021-OK160A_816_5.3.236.mgf	0	0	0	0	0	409	423			PEAKS DB
K.TVLEAVENDNK.G	Y	105.21	1230.6091	11	1.9	616.3104	2	26.66	2	F2:77659	20210611-09_2021021-OK160A_816_5.3.236.mgf		0	0	0	0	447	457			PEAKS DB
K.TVLEAVENDNKGPLVSLGMQASAWESVK.F	Y	94.51	2971.4961	28	5.6	991.5074	3	54.88	2	F2:126206	20210611-09_2021021-OK160A_816_5.3.236.mgf		0	0	0	0	447	474			PEAKS DB
K.LATSLGDLR.W	N	77.79	944.5291	9	3.7	473.2715	2	31.22	2	F2:85544	20210611-09_2021021-OK160A_816_5.3.236.mgf	0	0	0	0	0	424	432			PEAKS DB
K.NLLSTDFFLD	Y	56.00	1183.5760	10	-1.0	592.7922	2	60.15	2	F2:132237	20210611-09_2021021-OK160A_816_5.3.236.mgf		0	0	0	0	479	488			PEAKS DB
total 8 peptides																					

CebrOK035_J_342_Prohormone-4a

back to list

| Protein Coverage | Supporting Peptides |

Protein Coverage:

1 MNSFYPYSAA MMPFNSFRPP MHPISYLHPP MGYSFVYPAM NTMSPYNLMH PMYNPAHPMN HANPHSPLNP VTNLAHPMNP
81 ANPYSPINPV TNLAHPMNPA NPHSPLNPVT NLAHPMNPAN PYSPMNPVTN LAHPMNPANP YSPMNPVTNL AHPMNPANPH
161 SPLNPVTNLA HPMNPANPYS PINPVTNLAH PMNPANPYNP MNPVNSWYNP MNFQYPFHPP MQVASYYLTP PLGSSYLFPS
241 NGYGHFHLAP TQAAAPLNLH MHFPYSYQAG GMYNPYMYPY QSSALYQPYG YAAGVPGAMA WNQFGGAAWN PYSAMGYGVY
321 PMWPAQFAHM WPLHPYYQTV MPAQEGMCSY DRPFKCWGSA TCITIEAICD KNFDCNNHAD EDPRICVAGR RPSYGILVSY
401 LMLPNQQWML QK**LFNNVDPY TAAHALKLAT SLGDLRWSLA LTDENINNTK TVLEAVENDN KGPLVSLGMQ ASAWESVK**FL

481 MK**NLLSTDFF LD**

Supporting Peptides:

Peptide	Uniq	-10lgP	Mass	Length	ppm	m/z	z	RT	Fraction	Scan	Source File	Area ebraeus	Area judaeus	#Feature	#Feature ebraeus	#Feature judaeus	Start	End	PTM	AScore	Found By
K.GPLVSLGMQASAWESVK.F	Y	138.35	1758.8975	17	2.1	880.4542	2	50.02	2	F2:118803	20210611-09_2021021-OK160A_816_5.3.236.mgf		0	0	0	0	462	478			PEAKS DB
K.GPLVSLGM(+15.99)QASAWESVK.F	Y	135.06	1774.8923	17	0.3	888.4500	2	45.86	2	F2:112235	20210611-09_2021021-OK160A_816_5.3.236.mgf		0	0	0	0	462	478		M8:Oxidation (M):1000.00	PEAKS DB
R.WSLALTDENINNTK.T	N	121.92	1617.7998	14	-1.3	809.9027	2	42.21	2	F2:105115	20210611-09_2021021-OK160A_816_5.3.236.mgf	0	0	0	0	0	437	450			PEAKS DB
K.LFNNVDPYTAAHALK.L	N	118.88	1672.8573	15	-0.1	837.4324	2	38.61	2	F2:99023	20210611-09_2021021-OK160A_816_5.3.236.mgf	0	0	0	0	0	413	427			PEAKS DB
K.TVLEAVENDNK.G	Y	105.21	1230.6091	11	1.9	616.3104	2	26.66	2	F2:77659	20210611-09_2021021-OK160A_816_5.3.236.mgf		0	0	0	0	451	461			PEAKS DB
K.TVLEAVENDNKGPLVSLGMQASAWESVK.F	Y	94.51	2971.4961	28	5.6	991.5074	3	54.88	2	F2:126206	20210611-09_2021021-OK160A_816_5.3.236.mgf		0	0	0	0	451	478			PEAKS DB
K.LATSLGDLR.W	N	77.79	944.5291	9	3.7	473.2715	2	31.22	2	F2:85544	20210611-09_2021021-OK160A_816_5.3.236.mgf	0	0	0	0	0	428	436			PEAKS DB
K.NLLSTDFFLD	Y	56.00	1183.5760	10	-1.0	592.7922	2	60.15	2	F2:132237	20210611-09_2021021-OK160A_816_5.3.236.mgf		0	0	0	0	483	492			PEAKS DB
total 8 peptides																					

P_109D_104_Gelsolin-like

back to list

| Protein Coverage | Supporting Peptides |

Protein Coverage:

1 PGLKIWR**IEQ FEVK**DWPEED YGEFYNGDSY IILNTYKDEE TEDLNYDVHF WIGK**NSTQDE YGTAAYKTVE LDTLLDDKPV** O Oxidation (M) (+15.99)

104 O

81 **QHREVQGHES SLFK**SYFPK**G IQTMEGGANS GFR**SVPDKEK YEPRLMRFAK DKGGNIVVRE IPRACSLVTS GDVFILDAGL

O

161 KLYQFNGCSC SPMEKSKAMQ YIGDIKSK**RG EAESEVLDEA DTSK**NHEFYT YLDEEPEDDG CTSDDEDGDD AKKLLRVNTD

241 TRDFEEVKDG DVTQDDFKSD DVFIYDSGDT VFVWVGCNAS SAEKKNGLPL AHNYLCKCGS PWKSITVVKE GAKSKQFASA
321 LAA

Supporting Peptides:

Peptide	Uniq	-10lgP	Mass	Length	ppm	m/z	z	RT	Fraction	Scan	Source File	Area ebraeus	Area judaeus	#Feature	#Feature ebraeus	#Feature judaeus	Start	End	PTM	AScore	Found By
R.GEAESEVLDEADTSK.N	Y	131.43	1578.6897	15	0.6	790.3493	2	27.29	2	F2:78750	20210611-09_2021021-OK160A_816_5.3.236.mgf		0	0	0	0	190	204			PEAKS DB
K.TVELDTLLDDKPVQHR.E	Y	118.89	1877.9846	16	-2.1	626.9982	3	37.06	2	F2:96177	20210611-09_2021021-OK160A_816_5.3.236.mgf	0	0	0	0	0	68	83			PEAKS DB
K.RGEAESEVLDEADTSK.N	Y	112.45	1734.7908	16	1.7	579.2694	3	23.57	2	F2:72105	20210611-09_2021021-OK160A_816_5.3.236.mgf		0	0	0	0	189	204			PEAKS DB
K.GIQTM(+15.99)EGGANSGFR.S	Y	109.24	1439.6464	14	-0.4	720.8271	2	21.32	2	F2:68285	20210611-09_2021021-OK160A_816_5.3.236.mgf		0	0	0	0	100	113	Oxidation (M)	M5:Oxidation (M):1000.00	PEAKS DB
K.GIQTMEGGANSGFR.S	Y	108.25	1423.6514	14	1.4	712.8310	2	27.59	2	F2:79152	20210611-09_2021021-OK160A_816_5.3.236.mgf		0	0	0	0	100	113			PEAKS DB
K.NSTQDEYGTAAYK.T	Y	107.72	1446.6262	13	1.1	724.3181	2	19.51	2	F2:65106	20210611-09_2021021-OK160A_816_5.3.236.mgf	0	0	0	0	0	55	67			PEAKS DB
R.EVQGHESSLFK.S	Y	101.21	1259.6146	11	0.9	630.8125	2	20.74	2	F2:67101	20210611-09_2021021-OK160A_816_5.3.236.mgf		0	0	0	0	84	94			PEAKS DB
R.E(-18.01)VQGHESSLFK.S	Y	59.64	1241.6040	11	4.9	414.8756	3	20.82	2	F2:67499	20210611-09_2021021-OK160A_816_5.3.236.mgf		0	0	0	0	84	94		E1:Pyro-glu from E:1000.00	PEAKS DB
R.IEQFEVK.D	Y	57.17	891.4702	7	3.7	446.7423	2	24.97	1	F1:18135	20210611-08_2021021-OK192A_815_5.3.236.mgf	0	0	0	0	0	8	14			PEAKS DB
total 9 peptides																					

gene:107296774

back to list

| Protein Coverage | Supporting Peptides |

Protein Coverage:

1 MKYAIVCLLL VGSVFAAEDD DKREEVGTVV GIDLGTTYSC VGVFKNGR**VE IIANDQGNR**I TPSYVAFTPE GERLIGDAAK

O Oxidation (M) (+15.99)

81 **NQLTSNPENT VFDAK**RLIGR TWNDPSVQQD IKYLPFKVIE KKTKPYIQVD IGGGQTKTFA PEEISAMVLT KMKETAEAYL

161 GKKVTHAVVT VPAYFNDAQR QATK**DAGTIA GLNVMRIINE PTAAAIAYGL DKR**EGEKNIL VFDLGGGTFD VSLLTIDNGV

241 FEVVATNGDT HLGGEDFDQR VMEHFIKLYK KKTGKDVRKD NRAVQKLRRE VEKAKRALSA QHQARIEIES FFEGEDFSET

331

321 LTR**AKFEELN MDLFR**STMKP VQKVLEDADL K**KPDIDEIVL VGGSTR**IPKI QQLVKEFFNG KEPSRGINPD EAVAYGAAVQ

401 AGVLSGDQDT GDLVLLDVCP LTLGIETVGG VMTKLIPRNT VVPTKKSQIF STASDNQPTV TIKVYEGERP LTKDNHLLGT

481 FDLTGIPPAP RGVPQIEVTF EIDVNGILRV TAEDKGTGNK NKITITNDQN RLTPEEIERM VNDAEKFAEE DKRLKERIDA

561 R**NELESYAYS LK**NQIGDKEK LGGKLSSEEK ETIEKAVEEK IEWLESHQEA DIEDFKAQKK ELEEVVQPIV GKLYGGAGPP

641 PGGEEETAEK DEL

Supporting Peptides:

Peptide	Uniq	-10lgP	Mass	Length	ppm	m/z	z	RT	Fraction	Scan	Source File	Area ebraeus	Area judaeus	#Feature	#Feature ebraeus	#Feature judaeus	Start	End	PTM	AScore	Found By
R.IINEPTAAAIAYGLDK.R	Y	125.19	1658.8879	16	-1.5	830.4465	2	45.24	2	F2:110815	20210611-09_2021021-OK160A_816_5.3.236.mgf	0	0	0	0	0	197	212			PEAKS DB
R.IINEPTAAAIAYGLDKR.E	Y	112.63	1814.9890	17	1.0	908.4988	2	41.27	2	F2:103773	20210611-09_2021021-OK160A_816_5.3.236.mgf		0	0	0	0	197	213			PEAKS DB
R.VEIIANDQGNR.I	Y	100.83	1227.6207	11	3.2	614.8173	2	21.76	1	F1:13608	20210611-08_2021021-OK192A_815_5.3.236.mgf	0	0	0	0	0	49	59			PEAKS DB
K.DAGTIAGLNVMR.I	Y	94.30	1216.6234	12	0.9	609.3172	2	39.41	1	F1:36977	20210611-08_2021021-OK192A_815_5.3.236.mgf	0	0	0	0	0	185	196			PEAKS DB
K.KPDIDEIVLVGGSTR.I	Y	93.79	1597.8674	15	-1.5	533.6270	3	34.67	1	F1:31464	20210611-08_2021021-OK192A_815_5.3.236.mgf	0	0	0	0	0	352	366			PEAKS DB
R.NELESYAYSLK.N	Y	89.48	1315.6295	11	0.1	658.8196	2	37.72	1	F1:35070	20210611-08_2021021-OK192A_815_5.3.236.mgf	0	0	0	0	0	562	572			PEAKS DB
K.NQLTSNPENTVFDAK.R	Y	84.61	1676.8005	15	-11.7	839.3942	2	35.70	2	F2:93731	20210611-09_2021021-OK160A_816_5.3.236.mgf		0	0	0	0	81	95			PEAKS DB
R.AKFEELNMDLFR.S	Y	79.97	1511.7441	12	-4.9	504.9174	3	45.77	2	F2:112135	20210611-09_2021021-OK160A_816_5.3.236.mgf		0	0	0	0	324	335			PEAKS DB
R.AKFEELNM(+15.99)DLFR.S	Y	64.00	1527.7391	12	-0.3	510.2514	3	38.94	2	F2:99566	20210611-09_2021021-OK160A_816_5.3.236.mgf		0	0	0	0	324	335	Oxidation (M)	M8:Oxidation (M):1000.00	PEAKS DB
K.FEELNMDLFR.S	Y	53.75	1312.6122	10	-2.6	657.3091	2	50.88	1	F1:47911	20210611-08_2021021-OK192A_815_5.3.236.mgf	0		0	0	0	326	335			PEAKS DB
**total 10 peptides**																					

CebrOK033_J_270_PDI

back to list

| Protein Coverage | Supporting Peptides |

Protein Coverage:

1 MKMTGTGLVL FSLAVAGVSA MYSPSDDVVD LTPTNFHSKV INGDELWIVE FYAPWCGHCQ SLAPEWKKAA SALKGIVK**VG**

81 **AVNADEHNAL GGQYGVR**GFP TIKFFGLDK**F KPQDYQGQRT AQGIVDFSIN QAK**SVASARL SGKKSGDSKS KSGGSGK**AGD**

161 **PADVVELTDS NFEK**EVLEHD GLVMVEFYAP WCGHCK**NLAP QWAK**AASELK GKVKLCALDA TVHTVMANRY GVRGYPTIKA

241 FPAGKKDGEA EDYDGGR**TAS DIVQWAMEK**A AANIPSPDVY EITSGEVLTD NCEGHQLCII SVLPQIYDCQ SECRNNYISI

321 LKTLGEKFKK NQWGWLWAEA GAQPALEQSL EVGGFGYPAL AAVNTRKMKF SLLRGQFSEK **GINEFLR**DLS YGKGSTSPLR

401 NAELPKIEAR EPWDGK**DAEL PVEEDIDLSD VDFDDKDEL**

Supporting Peptides:

Peptide	Uniq	-10lgP	Mass	Length	ppm	m/z	z	RT	Fraction	Scan	Source File	Area ebraeus	Area judaeus	#Feature	#Feature ebraeus	#Feature judaeus	Start	End	PTM	AScore	Found By
K.VGAVNADEHNALGGQYGVR.G	N	137.07	1925.9343	19	3.3	642.9848	3	27.94	2	F2:79590	20210611-09_2021021-OK160A_816_5.3.236.mgf	0	0	0	0	0	79	97			PEAKS DB
K.AGDPADVVELTDSNFEK.E	N	114.88	1805.8319	17	0.1	903.9195	2	42.83	2	F2:106592	20210611-09_2021021-OK160A_816_5.3.236.mgf	0	0	0	0	0	158	174			PEAKS DB
R.TAQGIVDFSINQAK.S	N	101.65	1490.7728	14	-1.6	746.3893	2	39.25	2	F2:100088	20210611-09_2021021-OK160A_816_5.3.236.mgf		0	0	0	0	120	133			PEAKS DB
K.DAELPVEEDIDLSDVDFDDKDEL	N	98.43	2635.1548	23	14.0	1318.5975	2	57.62	2	F2:130012	20210611-09_2021021-OK160A_816_5.3.236.mgf		0	0	0	0	417	439			PEAKS DB
K.FKPQDYQGQR.T	Y	80.78	1265.6152	10	0.8	422.8776	3	17.52	2	F2:62434	20210611-09_2021021-OK160A_816_5.3.236.mgf		0	0	0	0	110	119			PEAKS DB
K.NLAPQWAK.A	N	74.58	926.4974	8	1.6	464.2548	2	27.41	2	F2:78699	20210611-09_2021021-OK160A_816_5.3.236.mgf	0	0	0	0	0	197	204			PEAKS DB
R.TASDIVQWAM(+15.99)EK.A	N	73.10	1393.6548	12	2.4	697.8334	2	38.40	2	F2:98771	20210611-09_2021021-OK160A_816_5.3.236.mgf		0	0	0	0	258	269		M10:Oxidation (M):1000.00	PEAKS DB
K.GINEFLR.D	N	60.54	847.4552	7	0.1	424.7332	2	34.12	2	F2:90614	20210611-09_2021021-OK160A_816_5.3.236.mgf		0	0	0	0	381	387			PEAKS DB
**total 8 peptides**																					

OK214_J_340_PDI

back to list

| Protein Coverage | Supporting Peptides |

Protein Coverage:

1 MKMTGTGLVL FSLAVAGVSA MYSPSDDVVD LTPTNFHSKV INGDELWIVE FYAPWCGHCQ SLAPEWKKAA SALKGIVK**VG**

81 **AVNADEHNAL GGQYGVR**GFP TIKFFGLDK**F KPQDYQGQRT AQGIVDFSIN QAK**SVASARL SGKKSGDSKS KSGGSGK**AGD**

161 **PADVVELTDS NFEK**EVLEHD GLVMVEFYAP WCGHCK**NLAP QWAK**AASELK GKVKLCALDA TVHTVMANRY GVRGYPTIKA

241 FPAGKKDGEA EDYDGGR**TAS DIVQWAMEK**A AANIPSPDVY EITSGEVLTD NCEGHQLCII SVLPQIYDCQ SECRNNYISI

321 LKTLGEKFKK NQWGWLWAEA GAQPALEQSL EVGGFGYPAL AAVNTRKMKF SLLRGQFSEK **GINEFLR**DLS YGKGSTSPLR

401 NAELPKIEAR EPWDGK**DAEL PVEEDIDLSD VDFDDKDEL**

Supporting Peptides:

Peptide	Uniq	-10lgP	Mass	Length	ppm	m/z	z	RT	Fraction	Scan	Source File	Area ebraeus	Area judaeus	#Feature	#Feature ebraeus	#Feature judaeus	Start	End	PTM	AScore	Found By
K.VGAVNADEHNALGGQYGVR.G	N	137.07	1925.9343	19	3.3	642.9848	3	27.94	2	F2:79590	20210611-09_2021021-OK160A_816_5.3.236.mgf	0	0	0	0	0	79	97			PEAKS DB
K.AGDPADVVELTDSNFEK.E	N	114.88	1805.8319	17	0.1	903.9195	2	42.83	2	F2:106592	20210611-09_2021021-OK160A_816_5.3.236.mgf	0	0	0	0	0	158	174			PEAKS DB
R.TAQGIVDFSINQAK.S	N	101.65	1490.7728	14	-1.6	746.3893	2	39.25	2	F2:100088	20210611-09_2021021-OK160A_816_5.3.236.mgf		0	0	0	0	120	133			PEAKS DB
K.DAELPVEEDIDLSDVDFDDKDEL	N	98.43	2635.1548	23	14.0	1318.5975	2	57.62	2	F2:130012	20210611-09_2021021-OK160A_816_5.3.236.mgf		0	0	0	0	417	439			PEAKS DB
K.FKPQDYQGQR.T	Y	80.78	1265.6152	10	0.8	422.8776	3	17.52	2	F2:62434	20210611-09_2021021-OK160A_816_5.3.236.mgf		0	0	0	0	110	119			PEAKS DB

total 8 peptides

Peptide	Uniq	-10lgP	Mass	Length	ppm	m/z	z	RT	Fraction	Scan	Source File	Area ebraeus	Area judaeus	#Feature	#Feature ebraeus	#Feature judaeus	Start	End	PTM	AScore	Found By
K.NLAPQWAK.A	N	74.58	926.4974	8	1.6	464.2548	2	27.41	2	F2:78699	20210611-09_2021021-OK160A_816_5.3.236.mgf	0	0	0	0	0	197	204			PEAKS DB
R.TASDIVQWAM(+15.99)EK.A	N	73.10	1393.6548	12	2.4	697.8334	2	38.40	2	F2:98771	20210611-09_2021021-OK160A_816_5.3.236.mgf		0	0	0	0	258	269		M10:Oxidation (M):1000.00	PEAKS DB
K.GINEFLR.D	N	60.54	847.4552	7	0.1	424.7332	2	34.12	2	F2:90614	20210611-09_2021021-OK160A_816_5.3.236.mgf		0	0	0	0	381	387			PEAKS DB
**total 8 peptides**																					

OK005_J_322_PDI

back to list

| Protein Coverage | Supporting Peptides |

Protein Coverage:

1 MKMTGTGLVL FSLAVAGVSA MYSPSDDVVD LTPTNFHSKV INGDELWIVE FYAPWCGHCQ SLAPEWKKAA SALKGIVK**VG**

81 **AVNADEHNAL GGQYGVR**GFP TIKFFGLDK**F KPQDYQGQRT AQGIVDFSIN QAK**SVASARL SGKKSGDSKS KSGGSGK**AGD**

161 **PADVVELTDS NFEK**EVLEHD GLVMVEFYAP WCGHCK**NLAP QWAK**AASELK GKVKLCALDA TVHTVMANRY GVRGYPTIKA

241 FPAGKKDGEA EDYDGGR**TAS DIVQWAMEK**A AANIPSPDVY EITSGEVLTD NCEGHQLCII SVLPQIYDCQ SECRNNYISI

321 LKTLGEKFKK NQWGWLWAEA GAQPALEQSL EVGGFGYPAL AAVNTRKMKF SLLRGQFSEK **GINEFLR**DLS YGKGSTSPLR

401 NAELPKIEAR EAWDGK**DAEL PVEEDIDLSD VDFDDKDEL**

Supporting Peptides:

Peptide	Uniq	-10lgP	Mass	Length	ppm	m/z	z	RT	Fraction	Scan	Source File	Area ebraeus	Area judaeus	#Feature	#Feature ebraeus	#Feature judaeus	Start	End	PTM	AScore	Found By
K.VGAVNADEHNALGGQYGVR.G	N	137.07	1925.9343	19	3.3	642.9848	3	27.94	2	F2:79590	20210611-09_2021021-OK160A_816_5.3.236.mgf	0	0	0	0	0	79	97			PEAKS DB
K.AGDPADVVELTDSNFEK.E	N	114.88	1805.8319	17	0.1	903.9195	2	42.83	2	F2:106592	20210611-09_2021021-OK160A_816_5.3.236.mgf	0	0	0	0	0	158	174			PEAKS DB
R.TAQGIVDFSINQAK.S	N	101.65	1490.7728	14	-1.6	746.3893	2	39.25	2	F2:100088	20210611-09_2021021-OK160A_816_5.3.236.mgf		0	0	0	0	120	133			PEAKS DB
K.DAELPVEEDIDLSDVDFDDKDEL	N	98.43	2635.1548	23	14.0	1318.5975	2	57.62	2	F2:130012	20210611-09_2021021-OK160A_816_5.3.236.mgf		0	0	0	0	417	439			PEAKS DB
K.FKPQDYQGQR.T	Y	80.78	1265.6152	10	0.8	422.8776	3	17.52	2	F2:62434	20210611-09_2021021-OK160A_816_5.3.236.mgf		0	0	0	0	110	119			PEAKS DB
K.NLAPQWAK.A	N	74.58	926.4974	8	1.6	464.2548	2	27.41	2	F2:78699	20210611-09_2021021-OK160A_816_5.3.236.mgf	0	0	0	0	0	197	204			PEAKS DB
R.TASDIVQWAM(+15.99)EK.A	N	73.10	1393.6548	12	2.4	697.8334	2	38.40	2	F2:98771	20210611-09_2021021-OK160A_816_5.3.236.mgf		0	0	0	0	258	269		M10:Oxidation (M):1000.00	PEAKS DB
K.GINEFLR.D	N	60.54	847.4552	7	0.1	424.7332	2	34.12	2	F2:90614	20210611-09_2021021-OK160A_816_5.3.236.mgf		0	0	0	0	381	387			PEAKS DB
**total 8 peptides**																					

OK212_J_328_PDI

back to list

| Protein Coverage | Supporting Peptides |

Protein Coverage:

1 MKMTGTGLVL FSLAVAGVSA MYSPSDDVVD LTPTNFHSKV INGDELWIVE FYAPWCGHCQ SLAPEWKKAA SALKGIVK**VG**

81 **AVNADEHNAL GGQYGVR**GFP TIKFFGLDK**F KPQDYQGQRT AQGIVDFSIN QAK**SVASARL SGKKSGDSKS KSGGSGK**AGD**

161 **PADVVELTDS NFEK**EVLEHD GLVMVEFYAP WCGHCK**NLAP QWAK**AASELK GKVKLCALDA TVHTVMANRY GVRGYPTIKA

241 FPAGKKDGEA EDYDGGR**TAS DIVQWAMEK**A AANIPSPDVY EITSGEVLTD NCEGHQLCII SVLPQIYDCQ SECRYNYISI

321 LKTLGEKFKK NQWGWLWAEA GAQPALEQSL EVGGFGYPAL AAVNTRKMKF SLLRGQFSEK **GINEFLR**DLS YGKGSTSPLR

401 NAELPKIEAR EAWDGK**DAEL PVEEDIDLSD VDFDDKDEL**

Supporting Peptides:

Peptide	Uniq	-10lgP	Mass	Length	ppm	m/z	z	RT	Fraction	Scan	Source File	Area ebraeus	Area judaeus	#Feature	#Feature ebraeus	#Feature judaeus	Start	End	PTM	AScore	Found By
K.VGAVNADEHNALGGQYGVR.G	N	137.07	1925.9343	19	3.3	642.9848	3	27.94	2	F2:79590	20210611-09_2021021-OK160A_816_5.3.236.mgf	0	0	0	0	0	79	97			PEAKS DB
K.AGDPADVVELTDSNFEK.E	N	114.88	1805.8319	17	0.1	903.9195	2	42.83	2	F2:106592	20210611-09_2021021-OK160A_816_5.3.236.mgf	0	0	0	0	0	158	174			PEAKS DB
R.TAQGIVDFSINQAK.S	N	101.65	1490.7728	14	-1.6	746.3893	2	39.25	2	F2:100088	20210611-09_2021021-OK160A_816_5.3.236.mgf		0	0	0	0	120	133			PEAKS DB
K.DAELPVEEDIDLSDVDFDDKDEL	N	98.43	2635.1548	23	14.0	1318.5975	2	57.62	2	F2:130012	20210611-09_2021021-OK160A_816_5.3.236.mgf		0	0	0	0	417	439			PEAKS DB
K.FKPQDYQGQR.T	Y	80.78	1265.6152	10	0.8	422.8776	3	17.52	2	F2:62434	20210611-09_2021021-OK160A_816_5.3.236.mgf		0	0	0	0	110	119			PEAKS DB
K.NLAPQWAK.A	N	74.58	926.4974	8	1.6	464.2548	2	27.41	2	F2:78699	20210611-09_2021021-OK160A_816_5.3.236.mgf	0	0	0	0	0	197	204			PEAKS DB
R.TASDIVQWAM(+15.99)EK.A	N	73.10	1393.6548	12	2.4	697.8334	2	38.40	2	F2:98771	20210611-09_2021021-OK160A_816_5.3.236.mgf		0	0	0	0	258	269		M10:Oxidation (M):1000.00	PEAKS DB
K.GINEFLR.D	N	60.54	847.4552	7	0.1	424.7332	2	34.12	2	F2:90614	20210611-09_2021021-OK160A_816_5.3.236.mgf		0	0	0	0	381	387			PEAKS DB
**total 8 peptides**																					

OK214_J_341_PDI

back to list

| Protein Coverage | Supporting Peptides |

Protein Coverage:

1 MKMTGTGLVL FSLAVAGVSA MYSASDDVVD LTPTNFHSKV INGDELWIVE FYAPWCGHCQ SLAPEWKKAA SALKGIVK**VG**

81 **AVNADEHNAL GGQYGVR**GFP TIKFFGLDK**F KPQDYQGQRT AQGIVDFSIN QAK**SVASARL SGKKSGDSKS KSGGSGK**AGD**

161 **PADVVELTDS NFEK**EVLEHD GLVMVEFYAP WCGHCK**NLAP QWAK**AASELK GKVKLCALDA TVHTVMANRY GVRGYPTIKA

241 FPAGKKDGEA EDYDGGR**TAS DIVQWAMEK**A AANIPSPDVY EITSGEVLTD NCEGHQLCII SVLPQIYDCQ SECRNNYISI

321 LKTLGEKFKK NQWGWLWAEA GAQPALEQSL EVGGFGYPAL AAVNTRKMKF SLLRGQFSEK **GINEFLR**DLS YGKGSTSPLR

401 NAELPKIEAR EPWDGK**DAEL PVEEDIDLSD VDFDDKDEL**

Supporting Peptides:

Peptide	Uniq	-10lgP	Mass	Length	ppm	m/z	z	RT	Fraction	Scan	Source File	Area ebraeus	Area judaeus	#Feature	#Feature ebraeus	#Feature judaeus	Start	End	PTM	AScore	Found By
K.VGAVNADEHNALGGQYGVR.G	N	137.07	1925.9343	19	3.3	642.9848	3	27.94	2	F2:79590	20210611-09_2021021-OK160A_816_5.3.236.mgf	0	0	0	0	0	79	97			PEAKS DB
K.AGDPADVVELTDSNFEK.E	N	114.88	1805.8319	17	0.1	903.9195	2	42.83	2	F2:106592	20210611-09_2021021-OK160A_816_5.3.236.mgf	0	0	0	0	0	158	174			PEAKS DB
R.TAQGIVDFSINQAK.S	N	101.65	1490.7728	14	-1.6	746.3893	2	39.25	2	F2:100088	20210611-09_2021021-OK160A_816_5.3.236.mgf		0	0	0	0	120	133			PEAKS DB
**total 8 peptides**																					

Peptide	Uniq	-10lgP	Mass	Length	ppm	m/z	z	RT	Fraction	Scan	Source File	Area ebraeus	Area judaeus	#Feature	#Feature ebraeus	#Feature judaeus	Start	End	PTM	AScore	Found By
K.DAELPVEEDIDLSDVDFDDKDEL	N	98.43	2635.1548	23	14.0	1318.5975	2	57.62	2	F2:130012	20210611-09_2021021-OK160A_816_5.3.236.mgf		0	0	0	0	417	439			PEAKS DB
K.FKPQDYQGQR.T	Y	80.78	1265.6152	10	0.8	422.8776	3	17.52	2	F2:62434	20210611-09_2021021-OK160A_816_5.3.236.mgf		0	0	0	0	110	119			PEAKS DB
K.NLAPQWAK.A	N	74.58	926.4974	8	1.6	464.2548	2	27.41	2	F2:78699	20210611-09_2021021-OK160A_816_5.3.236.mgf	0	0	0	0	0	197	204			PEAKS DB
R.TASDIVQWAM(+15.99)EK.A	N	73.10	1393.6548	12	2.4	697.8334	2	38.40	2	F2:98771	20210611-09_2021021-OK160A_816_5.3.236.mgf		0	0	0	0	258	269		M10:Oxidation (M):1000.00	PEAKS DB
K.GINEFLR.D	N	60.54	847.4552	7	0.1	424.7332	2	34.12	2	F2:90614	20210611-09_2021021-OK160A_816_5.3.236.mgf		0	0	0	0	381	387			PEAKS DB
total 8 peptides																					

OK006_E_298_PDI

back to list

| Protein Coverage | Supporting Peptides |

Protein Coverage:

1 MKMTGTGLVL FSLAVAGVSA MYSPSDDVVD LTPTNFHSKV INGDELWIVE FYAPWCGHCQ SLAPEWKKAA SALKGIVK**VG**

81 **AVNADEHNAL GGQYGVR**GFP TIKFFGQDK**F NPQDYQGQRT AQGIVDFSIN QAK**SVASARL SGKKSGDSKS KSGGSGK**AGD**

161 **PADVVELTDS NFEK**EVLEHD GLVMVEFYAP WCGHCK**NLAP QWAK**AASELK GKVKLCALDA TVHTVMANRY GVRGYPTIKA

241 FPAGKKDGEA EDYDGGR**TAS DIVQWAMEK**A AANIPSPDVY EITSGEVLTD NCEGHQLCII SVLPQIYDCQ SECRNNYISI

321 LKTLGEKFKK NQWGWLWAEA GAQPALEQSL EVGGFGYPAL AAVNTRKMKF SLLRGQFSEK **GINEFLR**DLS YGKGSTSPLR

401 NAELPKIEAR EPWDGK**DAEL PVEEDIDLSD VDFDDKDEL**

Supporting Peptides:

Peptide	Uniq	-10lgP	Mass	Length	ppm	m/z	z	RT	Fraction	Scan	Source File	Area ebraeus	Area judaeus	#Feature	#Feature ebraeus	#Feature judaeus	Start	End	PTM	AScore	Found By
K.VGAVNADEHNALGGQYGVR.G	N	137.07	1925.9343	19	3.3	642.9848	3	27.94	2	F2:79590	20210611-09_2021021-OK160A_816_5.3.236.mgf	0	0	0	0	0	79	97			PEAKS DB
K.AGDPADVVELTDSNFEK.E	N	114.88	1805.8319	17	0.1	903.9195	2	42.83	2	F2:106592	20210611-09_2021021-OK160A_816_5.3.236.mgf	0	0	0	0	0	158	174			PEAKS DB
R.TAQGIVDFSINQAK.S	N	101.65	1490.7728	14	-1.6	746.3893	2	39.25	2	F2:100088	20210611-09_2021021-OK160A_816_5.3.236.mgf		0	0	0	0	120	133			PEAKS DB
K.DAELPVEEDIDLSDVDFDDKDEL	N	98.43	2635.1548	23	14.0	1318.5975	2	57.62	2	F2:130012	20210611-09_2021021-OK160A_816_5.3.236.mgf		0	0	0	0	417	439			PEAKS DB
K.NLAPQWAK.A	N	74.58	926.4974	8	1.6	464.2548	2	27.41	2	F2:78699	20210611-09_2021021-OK160A_816_5.3.236.mgf	0	0	0	0	0	197	204			PEAKS DB
R.TASDIVQWAM(+15.99)EK.A	N	73.10	1393.6548	12	2.4	697.8334	2	38.40	2	F2:98771	20210611-09_2021021-OK160A_816_5.3.236.mgf		0	0	0	0	258	269		M10:Oxidation (M):1000.00	PEAKS DB
K.FNPQDYQGQR.T	Y	70.07	1251.5632	10	4.8	626.7896	2	22.75	1	F1:14753	20210611-08_2021021-OK192A_815_5.3.236.mgf	0		0	0	0	110	119			PEAKS DB
K.GINEFLR.D	N	60.54	847.4552	7	0.1	424.7332	2	34.12	2	F2:90614	20210611-09_2021021-OK160A_816_5.3.236.mgf		0	0	0	0	381	387			PEAKS DB
total 8 peptides																					

OK193_E_275_PDI

back to list

| Protein Coverage | Supporting Peptides |

Protein Coverage:

1 MKMTGTGLVL FSLAVAGVSA MYSPSDDVVD LTPTNFHSKV INGDELWIVE FYAPWCGHCQ SLAPEWKKAA SALKGIVK**VG**

81 **AVNADEHNAL GGQYGVR**GFP TIKFFGQDK**F NPQDYQGQRT AQGIVDFSIN QAK**SVASARL SGKKSGDSKS KSGGSGK**AGD**

161 **PADVVELTDS NFEK**EVLEHD GLVMVEFYAP WCGHCK**NLAP QWAK**AASELK GKVKLCALDA TVHTVMANRY GVRGYPTIKA

241 FPAGKKDGEA EDYDGGR**TAS DIVQWAMEK**A AANIPSPDVY EITSGEVLTD NCEGHQLCII SVLPQIYDCQ SECRNNYISI

321 LKTLGEKFKK NQWGWLWAEA GAQPALEQSL EVGGFGYPAL AAVNTRKMKF SLLRGQFSEK **GINEFLR**DLS YGKGSTSPLR

401 NAELPKIEAR EPWDGK**DAEL PVEEDIDLSD VDFDDKDEL**

Supporting Peptides:

Peptide	Uniq	-10lgP	Mass	Length	ppm	m/z	z	RT	Fraction	Scan	Source File	Area ebraeus	Area judaeus	#Feature	#Feature ebraeus	#Feature judaeus	Start	End	PTM	AScore	Found By
K.VGAVNADEHNALGGQYGVR.G	N	137.07	1925.9343	19	3.3	642.9848	3	27.94	2	F2:79590	20210611-09_2021021-OK160A_816_5.3.236.mgf	0	0	0	0	0	79	97			PEAKS DB
K.AGDPADVVELTDSNFEK.E	N	114.88	1805.8319	17	0.1	903.9195	2	42.83	2	F2:106592	20210611-09_2021021-OK160A_816_5.3.236.mgf	0	0	0	0	0	158	174			PEAKS DB
R.TAQGIVDFSINQAK.S	N	101.65	1490.7728	14	-1.6	746.3893	2	39.25	2	F2:100088	20210611-09_2021021-OK160A_816_5.3.236.mgf		0	0	0	0	120	133			PEAKS DB
K.DAELPVEEDIDLSDVDFDDKDEL	N	98.43	2635.1548	23	14.0	1318.5975	2	57.62	2	F2:130012	20210611-09_2021021-OK160A_816_5.3.236.mgf		0	0	0	0	417	439			PEAKS DB
K.NLAPQWAK.A	N	74.58	926.4974	8	1.6	464.2548	2	27.41	2	F2:78699	20210611-09_2021021-OK160A_816_5.3.236.mgf	0	0	0	0	0	197	204			PEAKS DB
R.TASDIVQWAM(+15.99)EK.A	N	73.10	1393.6548	12	2.4	697.8334	2	38.40	2	F2:98771	20210611-09_2021021-OK160A_816_5.3.236.mgf		0	0	0	0	258	269		M10:Oxidation (M):1000.00	PEAKS DB
K.FNPQDYQGQR.T	Y	70.07	1251.5632	10	4.8	626.7896	2	22.75	1	F1:14753	20210611-08_2021021-OK192A_815_5.3.236.mgf	0		0	0	0	110	119			PEAKS DB
K.GINEFLR.D	N	60.54	847.4552	7	0.1	424.7332	2	34.12	2	F2:90614	20210611-09_2021021-OK160A_816_5.3.236.mgf		0	0	0	0	381	387			PEAKS DB
total 8 peptides																					

SRX1323887_E_251_PDI

back to list

| Protein Coverage | Supporting Peptides |

Protein Coverage:

1 MKMTGTGLVL FSLAVAGVSA MYSPSDDVVD LTPTNFHTKV INGDELWIVE FYAPWCGHCQ SLAPEWKKAA SALKGIVK**VG**

81 **AVNADEHNAL GGQYGVR**GFP TIKFFGQDK**F NPQDYQGQRT AQGIVDFSIN QAK**SVASARL SGKKSGDSKS KSGGSGK**AGD**

161 **PADVVELTDS NFEK**EVLEHD GLVMVEFYAP WCGHCK**NLAP QWAK**AASELK GKVKLCALDA TVHTVMANRY GVRGYPTIKA

241 FPAGKKDGEA EDYDGGR**TAS DIVQWAMEK**A AANIPSPDVY EITSGEVLTD NCEGHQLCII SVLPQIYDCQ SECRNNYISI

321 LKTLGEKFKK NQWGWLWAEA GAQPALEQSL EVGGFGYPAL AAVNTRKMKF SLLRGQFSEK **GINEFLR**DLS YGKGSTSPLR

401 NAELPKIEAR EPWDGK**DAEL PVEEDIDLSD VDFDDKDEL**

Supporting Peptides:

Peptide	Uniq	-10lgP	Mass	Length	ppm	m/z	z	RT	Fraction	Scan	Source File	Area ebraeus	Area judaeus	#Feature	#Feature ebraeus	#Feature judaeus	Start	End	PTM	AScore	Found By
K.VGAVNADEHNALGGQYGVR.G	N	137.07	1925.9343	19	3.3	642.9848	3	27.94	2	F2:79590	20210611-09_2021021-OK160A_816_5.3.236.mgf	0	0	0	0	0	79	97			PEAKS DB
total 8 peptides																					

Peptide	Uniq	-10lgP	Mass	Length	ppm	m/z	z	RT	Fraction	Scan	Source File	Area ebraeus	Area judaeus	#Feature	#Feature ebraeus	#Feature judaeus	Start	End	PTM	AScore	Found By
K.AGDPADVVELTDSNFEK.E	N	114.88	1805.8319	17	0.1	903.9195	2	42.83	2	F2:106592	20210611-09_2021021-OK160A_816_5.3.236.mgf	0	0	0	0	0	158	174			PEAKS DB
R.TAQGIVDFSINQAK.S	N	101.65	1490.7728	14	-1.6	746.3893	2	39.25	2	F2:100088	20210611-09_2021021-OK160A_816_5.3.236.mgf		0	0	0	0	120	133			PEAKS DB
K.DAELPVEEDIDLSDVDFDDKDEL	N	98.43	2635.1548	23	14.0	1318.5975	2	57.62	2	F2:130012	20210611-09_2021021-OK160A_816_5.3.236.mgf		0	0	0	0	417	439			PEAKS DB
K.NLAPQWAK.A	N	74.58	926.4974	8	1.6	464.2548	2	27.41	2	F2:78699	20210611-09_2021021-OK160A_816_5.3.236.mgf	0	0	0	0	0	197	204			PEAKS DB
R.TASDIVQWAM(+15.99)EK.A	N	73.10	1393.6548	12	2.4	697.8334	2	38.40	2	F2:98771	20210611-09_2021021-OK160A_816_5.3.236.mgf		0	0	0	0	258	269		M10:Oxidation (M):1000.00	PEAKS DB
K.FNPQDYQGQR.T	Y	70.07	1251.5632	10	4.8	626.7896	2	22.75	1	F1:14753	20210611-08_2021021-OK192A_815_5.3.236.mgf	0		0	0	0	110	119			PEAKS DB
K.GINEFLR.D	N	60.54	847.4552	7	0.1	424.7332	2	34.12	2	F2:90614	20210611-09_2021021-OK160A_816_5.3.236.mgf		0	0	0	0	381	387			PEAKS DB
**total 8 peptides**																					

CV8_286_Protein

back to list

| Protein Coverage | Supporting Peptides |

Protein Coverage:

1 MLLAYFRPSA FTSPATMRRA VILFSCIVLA VQASDVLEFT DSNFVSKIKE LDVALVEFYA PWCGHCKRLA PEYEVAATKL
81 KKSDPPVPLV KVDCTAETK**T CQEYGVSGYP TLK**IFKGGEF AKDYQGPRES KGIISTMQKE AGPASKELED LK**QYEDFLGR**
161 DTVCVVGFFA DADSEMAKTF SKLADTHEGD IRFGHTTASA IMDKAK**YKDE VVLFRPK**AMQ NKFEDSAVKF TGDATKKSAI
241 KEFLTQEHLG MCGIRTMNNA GDFNKPTFVA FYDVDYVKNP KGSNYWRNRV MKVGKKLRDE GLK**VFFGVSN K**EEHSHELGE
321 CGLQDAEGDK PIVCAYDEKN RKFKMEEEFS VDSFEKFVRG VLNGEVEAYL K**SEPVPENND QPVK**VVVAKN FEELVNDPEK
401 DVLIEFYAPW CGHCKSLAPK **YDELAEKLKD ETDIVIAKMD ATANDPPSQY EVRGFPTIYF APK**GSKSSPK KYEGGREVND
481 FIKYLAK**EAT DELQGFDR**KG KKKGGQKQKK KTEL

Supporting Peptides:

Peptide	Uniq	-10lgP	Mass	Length	ppm	m/z	z	RT	Fraction	Scan	Source File	Area ebraeus	Area judaeus	#Feature	#Feature ebraeus	#Feature judaeus	Start	End	PTM	AScore	Found By
K.M(+15.99)DATANDPPSQYEVR.G	N	116.71	1708.7362	15	-0.3	855.3715	2	23.22	2	F2:71559	20210611-09_2021021-OK160A_816_5.3.236.mgf		0	0	0	0	439	453		M1:Oxidation (M):1000.00	PEAKS DB
K.SEPVPENNDQPVK.V	N	112.89	1451.6892	13	2.8	726.8509	2	19.00	2	F2:64401	20210611-09_2021021-OK160A_816_5.3.236.mgf	0	0	0	0	0	372	384			PEAKS DB
K.MDATANDPPSQYEVR.G	N	106.16	1692.7413	15	0.4	847.3748	2	26.03	2	F2:76318	20210611-09_2021021-OK160A_816_5.3.236.mgf		0	0	0	0	439	453			PEAKS DB
K.EATDELQGFDR.K	N	95.64	1279.5680	11	0.1	640.7886	2	29.87	2	F2:83300	20210611-09_2021021-OK160A_816_5.3.236.mgf	0	0	0	0	0	488	498			PEAKS DB
R.GFPTIYFAPK.G	N	86.32	1139.6014	10	-0.9	570.8051	2	46.08	2	F2:112594	20210611-09_2021021-OK160A_816_5.3.236.mgf	0	0	0	0	0	454	463			PEAKS DB
K.LKDETDIVIAK.M	N	82.41	1243.7023	11	0.0	622.8558	2	25.53	2	F2:75618	20210611-09_2021021-OK160A_816_5.3.236.mgf		0	0	0	0	428	438			PEAKS DB
K.YKDEVVLFRPK.A	Y	80.21	1392.7765	11	-2.0	465.2634	3	28.43	1	F1:23179	20210611-08_2021021-OK192A_815_5.3.236.mgf	0		0	0	0	207	217			PEAKS DB
K.VFFGVSNK.E	N	77.53	896.4756	8	2.5	449.2443	2	30.55	2	F2:84259	20210611-09_2021021-OK160A_816_5.3.236.mgf	0	0	0	0	0	304	311			PEAKS DB
K.YDELAEK.L	N	64.80	866.4021	7	4.6	434.2085	2	16.53	2	F2:60959	20210611-09_2021021-OK160A_816_5.3.236.mgf		0	0	0	0	421	427			PEAKS DB
K.QYEDFLGR.D	N	64.14	1026.4771	8	3.1	514.2452	2	30.02	2	F2:83542	20210611-09_2021021-OK160A_816_5.3.236.mgf		0	0	0	0	153	160			PEAKS DB
K.TC(+57.02)QEYGVSGYPTLK.I	N	58.68	1601.7395	14	0.4	801.8740	2	32.78	2	F2:88088	20210611-09_2021021-OK160A_816_5.3.236.mgf		0	0	0	0	100	113		C2:Carbamidomethylation:1000.00	PEAKS DB
**total 11 peptides**																					

V_CG13_228_Protein

back to list

| Protein Coverage | Supporting Peptides |

Protein Coverage:

1 MLLAYFRPSA FTSPATMRRA VILFSCIVLA VQASDVLEFT DLNFVSKIKE LDVALVEFYA PWCGHCKRLA PEYEVAATKL
81 KKSDPPVSLV KVDCTAETK**T CQEYGVSGYP TLK**IFKGGEF AKDYQGHRES KGIISTMQKE AGPASKELQD LK**QYEDFLGR**
161 DTVCVVGFFA DADSEMAKTF RKLADTHEGD VRFGHTTASA IMDKAK**YKDE VVLFRPK**AMQ NKFEDSAVKF TGDATKKSAI
241 KEFLTQEHLG MCGIRTMNNA GDFNKPTFVV FYDVDYVKNP KGSNYWRNRV MKVGKKLRDE GLK**VFFGVSN K**EEHSQELGE
321 CGLQDAEGDK PIVCAYDEKN RKFKMEEEFS VDSFEKFVRG VLNGEVEAYL K**SEPVPENND QPVK**VVVAKN FEELVNDPEK
401 DVLIEFYAPW CGHCKSLAPK **YDELAEKLKD ETDIVIAKMD ATANDPPSQY EVRGFPTIYF APK**GSKSSPK KYEGGREVND
481 FIKYLAK**EAT DELQGFDR**KG KKKGGQKQKK KTEL

Supporting Peptides:

Peptide	Uniq	-10lgP	Mass	Length	ppm	m/z	z	RT	Fraction	Scan	Source File	Area ebraeus	Area judaeus	#Feature	#Feature ebraeus	#Feature judaeus	Start	End	PTM	AScore	Found By
K.M(+15.99)DATANDPPSQYEVR.G	N	116.71	1708.7362	15	-0.3	855.3715	2	23.22	2	F2:71559	20210611-09_2021021-OK160A_816_5.3.236.mgf		0	0	0	0	439	453		M1:Oxidation (M):1000.00	PEAKS DB
K.SEPVPENNDQPVK.V	N	112.89	1451.6892	13	2.8	726.8509	2	19.00	2	F2:64401	20210611-09_2021021-OK160A_816_5.3.236.mgf	0	0	0	0	0	372	384			PEAKS DB
K.MDATANDPPSQYEVR.G	N	106.16	1692.7413	15	0.4	847.3748	2	26.03	2	F2:76318	20210611-09_2021021-OK160A_816_5.3.236.mgf		0	0	0	0	439	453			PEAKS DB
K.EATDELQGFDR.K	N	95.64	1279.5680	11	0.1	640.7886	2	29.87	2	F2:83300	20210611-09_2021021-OK160A_816_5.3.236.mgf	0	0	0	0	0	488	498			PEAKS DB
R.GFPTIYFAPK.G	N	86.32	1139.6014	10	-0.9	570.8051	2	46.08	2	F2:112594	20210611-09_2021021-OK160A_816_5.3.236.mgf	0	0	0	0	0	454	463			PEAKS DB
K.LKDETDIVIAK.M	N	82.41	1243.7023	11	0.0	622.8558	2	25.53	2	F2:75618	20210611-09_2021021-OK160A_816_5.3.236.mgf		0	0	0	0	428	438			PEAKS DB
K.YKDEVVLFRPK.A	Y	80.21	1392.7765	11	-2.0	465.2634	3	28.43	1	F1:23179	20210611-08_2021021-OK192A_815_5.3.236.mgf	0		0	0	0	207	217			PEAKS DB
K.VFFGVSNK.E	N	77.53	896.4756	8	2.5	449.2443	2	30.55	2	F2:84259	20210611-09_2021021-OK160A_816_5.3.236.mgf	0	0	0	0	0	304	311			PEAKS DB
K.YDELAEK.L	N	64.80	866.4021	7	4.6	434.2085	2	16.53	2	F2:60959	20210611-09_2021021-OK160A_816_5.3.236.mgf		0	0	0	0	421	427			PEAKS DB
K.QYEDFLGR.D	N	64.14	1026.4771	8	3.1	514.2452	2	30.02	2	F2:83542	20210611-09_2021021-OK160A_816_5.3.236.mgf		0	0	0	0	153	160			PEAKS DB
K.TC(+57.02)QEYGVSGYPTLK.I	N	58.68	1601.7395	14	0.4	801.8740	2	32.78	2	F2:88088	20210611-09_2021021-OK160A_816_5.3.236.mgf		0	0	0	0	100	113		C2:Carbamidomethylation:1000.00	PEAKS DB
**total 11 peptides**																					

A_1387_037

back to list

| Protein Coverage | Supporting Peptides |

Protein Coverage:

1 MSPATMRRAV ILFSCIVLAV QASDVLEFTD SNFVSKIKEL DVALVEFYAP WCGHCKRLAP EYEVAATKLK KSDPPVPLVK
81 VDCTAETKTC QEYGVSGYPT LKIFKGGEFA KDYQGPRESK GIISTMQKEA GPASKELEDL KQYEDFLGRD TVCVVGFFAD
161 ADSQMAKTFS KLADTHEGDI RFGHTTASAI MDKAKYKDEV VLFRPKAMQN KFEDSAVKFT GDATKKSAIK EFLTQEHLGM
241 CGIRTMNNAG DFNKPTFVAF YDVDYVKNPK GSNYWRNRVM KVGKKLRDEG LKVFFGVSNK EEHSQELGEC GLQDAEGDKP
321 IVCAYDEKNR KFKMEEEFSV DSFEKFVRGV LNGEVEAYLK SEPVPENNDQ PVKVVVAKNF EELVNDPEKD VLIEFYAPWC
401 GHCKSLAPKY DELAEKLKDE TDIVIAKMDA TANDPPSQYE VRGFPTIYFA PKGSKSSPKK YEGGREVNDF IKYLAKEATD
481 ELQGFDRKGK KKGGQKQKKK TEL

Supporting Peptides:

Peptide	Uniq	-10lgP	Mass	Length	ppm	m/z	z	RT	Fraction	Scan	Source File	Area ebraeus	Area judaeus	#Feature	#Feature ebraeus	#Feature judaeus	Start	End	PTM	AScore	Found By
K.M(+15.99)DATANDPPSQYEVR.G	N	116.71	1708.7362	15	-0.3	855.3715	2	23.22	2	F2:71559	20210611-09_2021021-OK160A_816_5.3.236.mgf		0	0	0	0	428	442		M1:Oxidation (M):1000.00	PEAKS DB
K.SEPVPENNDQPVK.V	N	112.89	1451.6892	13	2.8	726.8509	2	19.00	2	F2:64401	20210611-09_2021021-OK160A_816_5.3.236.mgf	0	0	0	0	0	361	373			PEAKS DB
K.MDATANDPPSQYEVR.G	N	106.16	1692.7413	15	0.4	847.3748	2	26.03	2	F2:76318	20210611-09_2021021-OK160A_816_5.3.236.mgf		0	0	0	0	428	442			PEAKS DB
K.EATDELQGFDR.K	N	95.64	1279.5680	11	0.1	640.7886	2	29.87	2	F2:83300	20210611-09_2021021-OK160A_816_5.3.236.mgf	0	0	0	0	0	477	487			PEAKS DB
R.GFPTIYFAPK.G	N	86.32	1139.6014	10	-0.9	570.8051	2	46.08	2	F2:112594	20210611-09_2021021-OK160A_816_5.3.236.mgf	0	0	0	0	0	443	452			PEAKS DB
K.LKDETDIVIAK.M	N	82.41	1243.7023	11	0.0	622.8558	2	25.53	2	F2:75618	20210611-09_2021021-OK160A_816_5.3.236.mgf		0	0	0	0	417	427			PEAKS DB
K.YKDEVVLFRPK.A	Y	80.21	1392.7765	11	-2.0	465.2634	3	28.43	1	F1:23179	20210611-08_2021021-OK192A_815_5.3.236.mgf	0		0	0	0	196	206			PEAKS DB
K.VFFGVSNK.E	N	77.53	896.4756	8	2.5	449.2443	2	30.55	2	F2:84259	20210611-09_2021021-OK160A_816_5.3.236.mgf	0	0	0	0	0	293	300			PEAKS DB
K.YDELAEK.L	N	64.80	866.4021	7	4.6	434.2085	2	16.53	2	F2:60959	20210611-09_2021021-OK160A_816_5.3.236.mgf		0	0	0	0	410	416			PEAKS DB
K.QYEDFLGR.D	N	64.14	1026.4771	8	3.1	514.2452	2	30.02	2	F2:83542	20210611-09_2021021-OK160A_816_5.3.236.mgf		0	0	0	0	142	149			PEAKS DB
K.TC(+57.02)QEYGVSGYPTLK.I	N	58.68	1601.7395	14	0.4	801.8740	2	32.78	2	F2:88088	20210611-09_2021021-OK160A_816_5.3.236.mgf		0	0	0	0	89	102		C2:Carbamidomethylation:1000.00	PEAKS DB
**total 11 peptides**																					

A_0025_374_Protein

back to list

| Protein Coverage | Supporting Peptides |

Protein Coverage:

1 MRRAVILFSC IVLAVQASDV LEFTDSNFVS KIKELDVALV EFYAPWCGHC KRLAPEYEVA ATKLKKSDPP VPLVKVDCTA
81 ETKTCQEYGV SGYPTLKIFK GGEFAKDYQG PRESKGIIST MQKEAGPASK ELEDLKQYED FLGRDTVCVV GFFADADSQM
161 AKTFSKLADT HEGDIRFGHT TASAIMDKAK YKDEVVLFRP KAMQNKFEDS AVKFTGNATK KSAIKEFLTQ EHLGICGIRT
241 MNNAGDFNKP TFVAFYDVDY VKNPKGSNYW RNRVMKVGKK LRDEGLKVFF GVSNKEEHSH ELGECGLQDA EGDKPIVCAY
321 DEKNRKFKME EEFSVDSFEK FVRGVLNGEV EAYLKSEPVP ENNDQPVKVV VAKNFEELVN DPEKDVLIEF YAPWCGHCKS
401 LAPKYDELAE KLKDETDIVI AKMDATANDP PSQYEVRGFP TIYFAPKGSK SSPKKYEGGR EVNDFIKYLA KEATDELQGF
481 DRKGKKKGGQ KQKKKTEL

Supporting Peptides:

Peptide	Uniq	-10lgP	Mass	Length	ppm	m/z	z	RT	Fraction	Scan	Source File	Area ebraeus	Area judaeus	#Feature	#Feature ebraeus	#Feature judaeus	Start	End	PTM	AScore	Found By
K.M(+15.99)DATANDPPSQYEVR.G	N	116.71	1708.7362	15	-0.3	855.3715	2	23.22	2	F2:71559	20210611-09_2021021-OK160A_816_5.3.236.mgf		0	0	0	0	423	437		M1:Oxidation (M):1000.00	PEAKS DB
K.SEPVPENNDQPVK.V	N	112.89	1451.6892	13	2.8	726.8509	2	19.00	2	F2:64401	20210611-09_2021021-OK160A_816_5.3.236.mgf	0	0	0	0	0	356	368			PEAKS DB
K.MDATANDPPSQYEVR.G	N	106.16	1692.7413	15	0.4	847.3748	2	26.03	2	F2:76318	20210611-09_2021021-OK160A_816_5.3.236.mgf		0	0	0	0	423	437			PEAKS DB
K.EATDELQGFDR.K	N	95.64	1279.5680	11	0.1	640.7886	2	29.87	2	F2:83300	20210611-09_2021021-OK160A_816_5.3.236.mgf	0	0	0	0	0	472	482			PEAKS DB
R.GFPTIYFAPK.G	N	86.32	1139.6014	10	-0.9	570.8051	2	46.08	2	F2:112594	20210611-09_2021021-OK160A_816_5.3.236.mgf	0	0	0	0	0	438	447			PEAKS DB
K.LKDETDIVIAK.M	N	82.41	1243.7023	11	0.0	622.8558	2	25.53	2	F2:75618	20210611-09_2021021-OK160A_816_5.3.236.mgf		0	0	0	0	412	422			PEAKS DB
K.YKDEVVLFRPK.A	Y	80.21	1392.7765	11	-2.0	465.2634	3	28.43	1	F1:23179	20210611-08_2021021-OK192A_815_5.3.236.mgf	0		0	0	0	191	201			PEAKS DB
K.VFFGVSNK.E	N	77.53	896.4756	8	2.5	449.2443	2	30.55	2	F2:84259	20210611-09_2021021-OK160A_816_5.3.236.mgf	0	0	0	0	0	288	295			PEAKS DB
K.YDELAEK.L	N	64.80	866.4021	7	4.6	434.2085	2	16.53	2	F2:60959	20210611-09_2021021-OK160A_816_5.3.236.mgf		0	0	0	0	405	411			PEAKS DB
K.QYEDFLGR.D	N	64.14	1026.4771	8	3.1	514.2452	2	30.02	2	F2:83542	20210611-09_2021021-OK160A_816_5.3.236.mgf		0	0	0	0	137	144			PEAKS DB
K.TC(+57.02)QEYGVSGYPTLK.I	N	58.68	1601.7395	14	0.4	801.8740	2	32.78	2	F2:88088	20210611-09_2021021-OK160A_816_5.3.236.mgf		0	0	0	0	84	97		C2:Carbamidomethylation:1000.00	PEAKS DB
**total 11 peptides**																					

A_0885_194_Protein

back to list

| Protein Coverage | Supporting Peptides |

Protein Coverage:

1 MLLAYFRPSA FTSPATMRRA VILFSCIVLA VQASDVLEFT DSNFVSKIKE LDVALVEFYA PWCGHCKRLA PEYEVAATKL
81 KKSDPPVPLV KVDCTAETKT CQEYGVSGYP TLKIFKGGEF AKDYQGPRES KGIINTMQKE AGPASKELED LKQYEDFLGR
161 DTVCVVGFFA DADSQMAKTF SKLADTHEGD IRFGHTTASA IMDKAKYKDE VVLFRPKAMQ NKFEDSAVKF TGDATKKSAI
241 KEFLTQEHLG ICGIRTMNNA GDFNKPTFVA FYDVDYVKNP KGSNYWRNRV MKVGKKLRDE GLKVFFGVSN KEEHSHELGE
321 CGLQDAEGDK PIVCAYDEKN RKFKMEEEFS VDSFEKFVRG VLNGEVEAYL KSEPVPENND QPVKVVVAKN FEELVNDPEK
401 DVLIEFYAPW CGHCKSLAPK YDELAEKLKD ETDIVIAKMD ATANDPPSQY EVRGFPTIYF APKGSKSSPK KYEGGREVND
481 FIKYLAKEAT DELQGFDRKG KKKGGQKQKK KTEL

Supporting Peptides:

Peptide	Uniq	-10lgP	Mass	Length	ppm	m/z	z	RT	Fraction	Scan	Source File	Area ebraeus	Area judaeus	#Feature	#Feature ebraeus	#Feature judaeus	Start	End	PTM	AScore	Found By
K.M(+15.99)DATANDPPSQYEVR.G	N	116.71	1708.7362	15	-0.3	855.3715	2	23.22	2	F2:71559	20210611-09_2021021-OK160A_816_5.3.236.mgf		0	0	0	0	439	453		M1:Oxidation (M):1000.00	PEAKS DB
K.SEPVPENNDQPVK.V	N	112.89	1451.6892	13	2.8	726.8509	2	19.00	2	F2:64401	20210611-09_2021021-OK160A_816_5.3.236.mgf	0	0	0	0	0	372	384			PEAKS DB
**total 11 peptides**																					

Peptide	Uniq	-10lgP	Mass	Length	ppm	m/z	z	RT	Fraction	Scan	Source File	Area ebraeus	Area judaeus	#Feature	#Feature ebraeus	#Feature judaeus	Start	End	PTM	AScore	Found By
K.MDATANDPPSQYEVR.G	N	106.16	1692.7413	15	0.4	847.3748	2	26.03	2	F2:76318	20210611-09_2021021-OK160A_816_5.3.236.mgf		0	0	0	0	439	453			PEAKS DB
K.EATDELQGFDR.K	N	95.64	1279.5680	11	0.1	640.7886	2	29.87	2	F2:83300	20210611-09_2021021-OK160A_816_5.3.236.mgf	0	0	0	0	0	488	498			PEAKS DB
R.GFPTIYFAPK.G	N	86.32	1139.6014	10	-0.9	570.8051	2	46.08	2	F2:112594	20210611-09_2021021-OK160A_816_5.3.236.mgf	0	0	0	0	0	454	463			PEAKS DB
K.LKDETDIVIAK.M	N	82.41	1243.7023	11	0.0	622.8558	2	25.53	2	F2:75618	20210611-09_2021021-OK160A_816_5.3.236.mgf		0	0	0	0	428	438			PEAKS DB
K.YKDEVVLFRPK.A	Y	80.21	1392.7765	11	-2.0	465.2634	3	28.43	1	F1:23179	20210611-08_2021021-OK192A_815_5.3.236.mgf	0		0	0	0	207	217			PEAKS DB
K.VFFGVSNK.E	N	77.53	896.4756	8	2.5	449.2443	2	30.55	2	F2:84259	20210611-09_2021021-OK160A_816_5.3.236.mgf	0	0	0	0	0	304	311			PEAKS DB
K.YDELAEK.L	N	64.80	866.4021	7	4.6	434.2085	2	16.53	2	F2:60959	20210611-09_2021021-OK160A_816_5.3.236.mgf		0	0	0	0	421	427			PEAKS DB
K.QYEDFLGR.D	N	64.14	1026.4771	8	3.1	514.2452	2	30.02	2	F2:83542	20210611-09_2021021-OK160A_816_5.3.236.mgf		0	0	0	0	153	160			PEAKS DB
K.TC(+57.02)QEYGVSGYPTLK.I	N	58.68	1601.7395	14	0.4	801.8740	2	32.78	2	F2:88088	20210611-09_2021021-OK160A_816_5.3.236.mgf		0	0	0	0	100	113		C2:Carbamidomethylation:100 0.00	PEAKS DB
total 11 peptides																					

A_0855_188_Protein

back to list

| Protein Coverage | Supporting Peptides |

Protein Coverage:

```
  1 LLAYFRPSAF MSPATMRRAV ILFSCIVLAV QASDVLEFTD SNFVSKIKEL DVALVEFYAP WCGHCKRLAP EYEVAATKLK
 81 KSDPPVPLVK VDCTAETKTC QEYGVSGYPT LKIFKGGEFA KDYQGPRESK GIISTMQKEA GPASKELEDL KQYEDFLGRD
161 TVCVVGFFAD ADSQMAKTFS KLADTHEGDI RFGHTTASAI MDKAKYKDEV VLFRPKAMQN KFEDSAVKFT GDATKKSAIK
241 EFLTQEHLGM CGIRTMNNAG DFNKPTFVAF YDVDYVKNPK GSNYWRNRVM KVGKKLRDEG LKVFFGVSNK EEHSHELGEC
321 GLQDAEGDKP IVCAYDEKNR KFKMEEEFSV DSFEKFVRGV LNGEVEAYLK SEPVPENNDQ PVKVVVAKNF EELVNDPEKD
401 VLIEFYAPWC GHCKSLAPKY DELAEKLKDE TDIVIAKMDA TANDPPSQYE VRGFPTIYFA PKGSKSSPKK YEGGREVNDF
481 IKYLAKEATD ELQGFDRKGK KKGGQKQKKK TEL
```

Supporting Peptides:

Peptide	Uniq	-10lgP	Mass	Length	ppm	m/z	z	RT	Fraction	Scan	Source File	Area ebraeus	Area judaeus	#Feature	#Feature ebraeus	#Feature judaeus	Start	End	PTM	AScore	Found By
K.M(+15.99)DATANDPPSQYEVR.G	N	116.71	1708.7362	15	-0.3	855.3715	2	23.22	2	F2:71559	20210611-09_2021021-OK160A_816_5.3.236.mgf		0	0	0	0	438	452		M1:Oxidation (M):1000.00	PEAKS DB
K.SEPVPENNDQPVK.V	N	112.89	1451.6892	13	2.8	726.8509	2	19.00	2	F2:64401	20210611-09_2021021-OK160A_816_5.3.236.mgf	0	0	0	0	0	371	383			PEAKS DB
K.MDATANDPPSQYEVR.G	N	106.16	1692.7413	15	0.4	847.3748	2	26.03	2	F2:76318	20210611-09_2021021-OK160A_816_5.3.236.mgf		0	0	0	0	438	452			PEAKS DB
K.EATDELQGFDR.K	N	95.64	1279.5680	11	0.1	640.7886	2	29.87	2	F2:83300	20210611-09_2021021-OK160A_816_5.3.236.mgf	0	0	0	0	0	487	497			PEAKS DB
R.GFPTIYFAPK.G	N	86.32	1139.6014	10	-0.9	570.8051	2	46.08	2	F2:112594	20210611-09_2021021-OK160A_816_5.3.236.mgf	0	0	0	0	0	453	462			PEAKS DB
K.LKDETDIVIAK.M	N	82.41	1243.7023	11	0.0	622.8558	2	25.53	2	F2:75618	20210611-09_2021021-OK160A_816_5.3.236.mgf		0	0	0	0	427	437			PEAKS DB
K.YKDEVVLFRPK.A	Y	80.21	1392.7765	11	-2.0	465.2634	3	28.43	1	F1:23179	20210611-08_2021021-OK192A_815_5.3.236.mgf	0		0	0	0	206	216			PEAKS DB
K.VFFGVSNK.E	N	77.53	896.4756	8	2.5	449.2443	2	30.55	2	F2:84259	20210611-09_2021021-OK160A_816_5.3.236.mgf	0	0	0	0	0	303	310			PEAKS DB
K.YDELAEK.L	N	64.80	866.4021	7	4.6	434.2085	2	16.53	2	F2:60959	20210611-09_2021021-OK160A_816_5.3.236.mgf		0	0	0	0	420	426			PEAKS DB
K.QYEDFLGR.D	N	64.14	1026.4771	8	3.1	514.2452	2	30.02	2	F2:83542	20210611-09_2021021-OK160A_816_5.3.236.mgf		0	0	0	0	152	159			PEAKS DB
K.TC(+57.02)QEYGVSGYPTLK.I	N	58.68	1601.7395	14	0.4	801.8740	2	32.78	2	F2:88088	20210611-09_2021021-OK160A_816_5.3.236.mgf		0	0	0	0	99	112		C2:Carbamidomethylation:100 0.00	PEAKS DB
total 11 peptides																					

A_0048_031_Protein

back to list

| Protein Coverage | Supporting Peptides |

Protein Coverage:

```
  1 MLLAYFRPSA FMSPATMRRA VILFSCIVLA VQASDVLEFT DSNFVSKIKE LDVALVEFYA PWCGHCKRLA PEYEVAATKL
 81 KKSDPPVPLV KVDCTAETKT CQEYGVSGYP TLKIFKGGEF AKDYQGPRES KGIISTMQKE AGPASKELED LKQYEDFLGR
161 DTVCVVGFFA DADSQMAKTF SKLADTHEGD IRFGHTTASA IMDKAKYKDE VVLFRPKAMQ NKFEDSAVKF TGNATKKSAI
241 KEFLTQEHLG ICGIRTMNNA GDFNKPTFVA FYDVDYVKNP KGSNYWRNRV MKVGKKLRDE GLKVFFGVSN KEEHSHELGE
321 CGLQDAEGDK PIVCAYDEKN RKFKMEEEFS VDSFEKFVRG VLNGEVEAYL KSEPVPENND QPVKVVVAKN FEELVNDPEK
401 DVLIEFYAPW CGHCKSLAPK YDELAEKLKD ETDIVIAKMD ATANDPPSQY EVRGFPTIYF APKGSKSSPK KYEGGREVND
481 FIKYLAKEAT DELQGFDRKG KKKGGQKQKK KTEL
```

Supporting Peptides:

Peptide	Uniq	-10lgP	Mass	Length	ppm	m/z	z	RT	Fraction	Scan	Source File	Area ebraeus	Area judaeus	#Feature	#Feature ebraeus	#Feature judaeus	Start	End	PTM	AScore	Found By
K.M(+15.99)DATANDPPSQYEVR.G	N	116.71	1708.7362	15	-0.3	855.3715	2	23.22	2	F2:71559	20210611-09_2021021-OK160A_816_5.3.236.mgf		0	0	0	0	439	453		M1:Oxidation (M):1000.00	PEAKS DB
K.SEPVPENNDQPVK.V	N	112.89	1451.6892	13	2.8	726.8509	2	19.00	2	F2:64401	20210611-09_2021021-OK160A_816_5.3.236.mgf	0	0	0	0	0	372	384			PEAKS DB
K.MDATANDPPSQYEVR.G	N	106.16	1692.7413	15	0.4	847.3748	2	26.03	2	F2:76318	20210611-09_2021021-OK160A_816_5.3.236.mgf		0	0	0	0	439	453			PEAKS DB
K.EATDELQGFDR.K	N	95.64	1279.5680	11	0.1	640.7886	2	29.87	2	F2:83300	20210611-09_2021021-OK160A_816_5.3.236.mgf	0	0	0	0	0	488	498			PEAKS DB
R.GFPTIYFAPK.G	N	86.32	1139.6014	10	-0.9	570.8051	2	46.08	2	F2:112594	20210611-09_2021021-OK160A_816_5.3.236.mgf	0	0	0	0	0	454	463			PEAKS DB
K.LKDETDIVIAK.M	N	82.41	1243.7023	11	0.0	622.8558	2	25.53	2	F2:75618	20210611-09_2021021-OK160A_816_5.3.236.mgf		0	0	0	0	428	438			PEAKS DB
K.YKDEVVLFRPK.A	Y	80.21	1392.7765	11	-2.0	465.2634	3	28.43	1	F1:23179	20210611-08_2021021-OK192A_815_5.3.236.mgf	0		0	0	0	207	217			PEAKS DB
K.VFFGVSNK.E	N	77.53	896.4756	8	2.5	449.2443	2	30.55	2	F2:84259	20210611-09_2021021-OK160A_816_5.3.236.mgf	0	0	0	0	0	304	311			PEAKS DB
K.YDELAEK.L	N	64.80	866.4021	7	4.6	434.2085	2	16.53	2	F2:60959	20210611-09_2021021-OK160A_816_5.3.236.mgf		0	0	0	0	421	427			PEAKS DB
K.QYEDFLGR.D	N	64.14	1026.4771	8	3.1	514.2452	2	30.02	2	F2:83542	20210611-09_2021021-OK160A_816_5.3.236.mgf		0	0	0	0	153	160			PEAKS DB
K.TC(+57.02)QEYGVSGYPTLK.I	N	58.68	1601.7395	14	0.4	801.8740	2	32.78	2	F2:88088	20210611-09_2021021-OK160A_816_5.3.236.mgf		0	0	0	0	100	113		C2:Carbamidomethylation:100 0.00	PEAKS DB
total 11 peptides																					

D_070_Calreticulin-like

back to list

| Protein Coverage | Supporting Peptides |

Protein Coverage:

1 MKTFVFCLAL YGVTFCDATK YFTEDFTENY EGRWVESTHK GSDQGKFKMA LGKFYGDEKV SYGLQTSQDA HFYGISAKFD

81 KFSNKGKDLI IQFTVK**HEQN IDCGGGYVK**L FSSDLDQSDM HGDTPYLIMF GPDICGPGTK **KVHVIFNYK**G KNLLTKKDIR

161 CKDDVYTHVY TLIVRPDNTY EVRIDTEKVE SGELEADWDF LPPKKIKDPE AK**KPEDWDER EKIDDPDDTK PEDWDKPEHI**

241 **PDPDAK**KPDD WDDEMDGEWE PPMIDNPDYK GEWKPKQIDN PDYKGK**WIHP EIDNPDYTPD DELYKYDDIG AIGFDLWQVK**

321 SGTIFDNVLI TDSEDDAKQH AEETWEATKD KEKKMKEEQD EEDRKKREEE EKKRKEEEGS KQSDDDEEEE EEEEEEPADD

401 ADAHDEL

Supporting Peptides:

Peptide	Uniq	-10lgP	Mass	Length	ppm	m/z	z	RT	Fraction	Scan	Source File	Area ebraeus	Area judaeus	#Feature	#Feature ebraeus	#Feature judaeus	Start	End	PTM	AScore	Found By
K.IDDPDDTKPEDWDKPEHIPDPDAK.K	Y	118.19	2787.2510	24	3.3	697.8194	4	32.09	2	F2:86820	20210611-09_2021021-OK160A_816_5.3.236.mgf		0	0	0	0	223	246			PEAKS DB
R.EKIDDPDDTKPEDWDKPEHIPDPDAK.K	Y	110.90	3044.3887	26	3.5	762.1039	4	30.59	2	F2:84334	20210611-09_2021021-OK160A_816_5.3.236.mgf		0	0	0	0	221	246			PEAKS DB
K.YDDIGAIGFDLWQVK.S	Y	110.71	1738.8566	15	-0.4	870.4316	2	58.42	2	F2:130731	20210611-09_2021021-OK160A_816_5.3.236.mgf		0	0	0	0	306	320			PEAKS DB
K.WIHPEIDNPDYTPDDELYK.Y	Y	94.73	2359.0645	19	-0.6	787.3583	3	42.30	2	F2:105539	20210611-09_2021021-OK160A_816_5.3.236.mgf		0	0	0	0	287	305			PEAKS DB
K.HEQNIDC(+57.02)GGGYVK.L	Y	93.35	1475.6464	13	-2.7	738.8254	2	17.81	2	F2:62817	20210611-09_2021021-OK160A_816_5.3.236.mgf		0	0	0	0	97	109		C7:Carbamidomethylation:1000.00	PEAKS DB
K.VHVIFNYK.G	Y	75.44	1018.5600	8	6.2	510.2883	2	28.46	2	F2:80756	20210611-09_2021021-OK160A_816_5.3.236.mgf		0	0	0	0	142	149			PEAKS DB
K.KPEDWDER.E	Y	71.81	1073.4778	8	3.2	537.7456	2	14.88	2	F2:58653	20210611-09_2021021-OK160A_816_5.3.236.mgf		0	0	0	0	213	220			PEAKS DB
K.KVHVIFNYK.G	Y	70.41	1146.6549	9	4.5	383.2258	3	23.71	1	F1:16254	20210611-08_2021021-OK192A_815_5.3.236.mgf	0	0	0	0	0	141	149			PEAKS DB
**total 8 peptides**																					

OK212_J_296_Ferritin

back to list

| Protein Coverage | Supporting Peptides |

Protein Coverage:

1 MAHNVNQCR**Q NYHAESEAGV NR**QINMELYA SYCYQSMSFY FDR**DDVALPG FAK**FFKEASD EEREHAEKLM KFQNKRGGRV

81 VLQDIKKPDR **DEWGTGLDAM QVALGLEKSV NQALLDLHEV AASHKDAQMT DFLEGEYLQE QVESIK**K**IGD HITNLK**R**VGT**

161 **GLGEYMFDK**N LS

Supporting Peptides:

Peptide	Uniq	-10lgP	Mass	Length	ppm	m/z	z	RT	Fraction	Scan	Source File	Area ebraeus	Area judaeus	#Feature	#Feature ebraeus	#Feature judaeus	Start	End	PTM	AScore	Found By
K.DAQMTDFLEGEYLQEQVESIK.K	Y	116.93	2472.1365	21	13.6	1237.0872	2	57.77	2	F2:130150	20210611-09_2021021-OK160A_816_5.3.236.mgf		0	0	0	0	126	146			PEAKS DB
R.DEWGTGLDAMQVALGLEK.S	N	116.51	1931.9298	18	1.8	966.9699	2	59.74	2	F2:131980	20210611-09_2021021-OK160A_816_5.3.236.mgf		0	0	0	0	91	108			PEAKS DB
K.SVNQALLDLHEVAASHK.D	Y	115.94	1830.9587	17	2.0	611.3255	3	37.13	2	F2:96468	20210611-09_2021021-OK160A_816_5.3.236.mgf	0	0	0	0	0	109	125			PEAKS DB
R.QNYHAESEAGVNR.Q	N	96.97	1473.6597	13	1.1	737.8348	2	13.59	2	F2:56908	20210611-09_2021021-OK160A_816_5.3.236.mgf	0	0	0	0	0	10	22			PEAKS DB
R.Q(-17.03)NYHAESEAGVNR.Q	N	91.36	1456.6331	13	-0.3	729.3205	2	18.75	2	F2:64244	20210611-09_2021021-OK160A_816_5.3.236.mgf		0	0	0	0	10	22		Q1:Pyro-glu from Q:1000.00	PEAKS DB
R.DDVALPGFAK.F	N	90.93	1031.5287	10	2.5	516.7708	2	38.01	2	F2:97999	20210611-09_2021021-OK160A_816_5.3.236.mgf	0	0	0	0	0	44	53			PEAKS DB
R.VGTGLGEYMFDK.N	Y	89.63	1315.6118	12	0.0	658.8104	2	40.56	2	F2:102477	20210611-09_2021021-OK160A_816_5.3.236.mgf		0	0	0	0	158	169			PEAKS DB
K.IGDHITNLK.R	N	89.25	1009.5556	9	5.8	505.7859	2	19.85	2	F2:65725	20210611-09_2021021-OK160A_816_5.3.236.mgf		0	0	0	0	148	156			PEAKS DB
**total 8 peptides**																					

CebrOK033_J_246_Ferritin

back to list

| Protein Coverage | Supporting Peptides |

Protein Coverage:

1 MAHNVSQCR**Q NYHAESEAGV NR**QINMELYA SYCYQSMSFY FDR**DDVALPG FAK**FFKEASD EEREHAEKLM KFQNKRGGRV

81 VLQDIKKPDR **DEWGTGLDAM QVALGLEKSV NQALLDLHEV AASHKDAQMT DFLEGEYLQE QVESIK**K**IGD HITNLK**R**VGT**

161 **GLGEYMFDK**N LS

Supporting Peptides:

Peptide	Uniq	-10lgP	Mass	Length	ppm	m/z	z	RT	Fraction	Scan	Source File	Area ebraeus	Area judaeus	#Feature	#Feature ebraeus	#Feature judaeus	Start	End	PTM	AScore	Found By
K.DAQMTDFLEGEYLQEQVESIK.K	Y	116.93	2472.1365	21	13.6	1237.0872	2	57.77	2	F2:130150	20210611-09_2021021-OK160A_816_5.3.236.mgf		0	0	0	0	126	146			PEAKS DB
R.DEWGTGLDAMQVALGLEK.S	N	116.51	1931.9298	18	1.8	966.9699	2	59.74	2	F2:131980	20210611-09_2021021-OK160A_816_5.3.236.mgf		0	0	0	0	91	108			PEAKS DB
K.SVNQALLDLHEVAASHK.D	Y	115.94	1830.9587	17	2.0	611.3255	3	37.13	2	F2:96468	20210611-09_2021021-OK160A_816_5.3.236.mgf	0	0	0	0	0	109	125			PEAKS DB
R.QNYHAESEAGVNR.Q	N	96.97	1473.6597	13	1.1	737.8348	2	13.59	2	F2:56908	20210611-09_2021021-OK160A_816_5.3.236.mgf	0	0	0	0	0	10	22			PEAKS DB
R.Q(-17.03)NYHAESEAGVNR.Q	N	91.36	1456.6331	13	-0.3	729.3205	2	18.75	2	F2:64244	20210611-09_2021021-OK160A_816_5.3.236.mgf		0	0	0	0	10	22		Q1:Pyro-glu from Q:1000.00	PEAKS DB
R.DDVALPGFAK.F	N	90.93	1031.5287	10	2.5	516.7708	2	38.01	2	F2:97999	20210611-09_2021021-OK160A_816_5.3.236.mgf	0	0	0	0	0	44	53			PEAKS DB
R.VGTGLGEYMFDK.N	Y	89.63	1315.6118	12	0.0	658.8104	2	40.56	2	F2:102477	20210611-09_2021021-OK160A_816_5.3.236.mgf		0	0	0	0	158	169			PEAKS DB
K.IGDHITNLK.R	N	89.25	1009.5556	9	5.8	505.7859	2	19.85	2	F2:65725	20210611-09_2021021-OK160A_816_5.3.236.mgf		0	0	0	0	148	156			PEAKS DB
**total 8 peptides**																					

OK214_J_315_Ferritin

back to list

| Protein Coverage | Supporting Peptides |

Protein Coverage:

1 MAHNVSQCR**Q NYHAESEAGV NR**QINMELYA SYCYQSMSFY FDR**DDVALPG FAK**FFKEASD EEREHAEKLM KFQNKRGGRV

81 VLQDIKKPDR **DEWGTGLDAM QVALGLEKSV NQALLDLHEV AASHKDAQMT DFLEGEYLQE QVESIK**K**IGD HITNLK**R**VGT**

161 **GLGEYMFDK**N LS

Supporting Peptides:

Peptide	Uniq	-10lgP	Mass	Length	ppm	m/z	z	RT	Fraction	Scan	Source File	Area ebraeus	Area judaeus	#Feature	#Feature ebraeus	#Feature judaeus	Start	End	PTM	AScore	Found By
**total 8 peptides**																					

Peptide	Uniq	-10lgP	Mass	Length	ppm	m/z	z	RT	Fraction	Scan	Source File	Area ebraeus	Area judaeus	#Feature	#Feature ebraeus	#Feature judaeus	Start	End	PTM	AScore	Found By
K.DAQMTDFLEGEYLQEQVESIK.K	Y	116.93	2472.1365	21	13.6	1237.0872	2	57.77	2	F2:130150	20210611-09_2021021-OK160A_816_5.3.236.mgf		0	0	0	0	126	146			PEAKS DB
R.DEWGTGLDAMQVALGLEK.S	N	116.51	1931.9298	18	1.8	966.9699	2	59.74	2	F2:131980	20210611-09_2021021-OK160A_816_5.3.236.mgf		0	0	0	0	91	108			PEAKS DB
K.SVNQALLDLHEVAASHK.D	Y	115.94	1830.9587	17	2.0	611.3255	3	37.13	2	F2:96468	20210611-09_2021021-OK160A_816_5.3.236.mgf	0	0	0	0	0	109	125			PEAKS DB
R.QNYHAESEAGVNR.Q	N	96.97	1473.6597	13	1.1	737.8348	2	13.59	2	F2:56908	20210611-09_2021021-OK160A_816_5.3.236.mgf	0	0	0	0	0	10	22			PEAKS DB
R.Q(-17.03)NYHAESEAGVNR.Q	N	91.36	1456.6331	13	-0.3	729.3205	2	18.75	2	F2:64244	20210611-09_2021021-OK160A_816_5.3.236.mgf		0	0	0	0	10	22		Q1:Pyro-glu from Q:1000.00	PEAKS DB
R.DDVALPGFAK.F	N	90.93	1031.5287	10	2.5	516.7708	2	38.01	2	F2:97999	20210611-09_2021021-OK160A_816_5.3.236.mgf	0	0	0	0	0	44	53			PEAKS DB
R.VGTGLGEYMFDK.N	Y	89.63	1315.6118	12	0.0	658.8104	2	40.56	2	F2:102477	20210611-09_2021021-OK160A_816_5.3.236.mgf		0	0	0	0	158	169			PEAKS DB
K.IGDHITNLK.R	N	89.25	1009.5556	9	5.8	505.7859	2	19.85	2	F2:65725	20210611-09_2021021-OK160A_816_5.3.236.mgf		0	0	0	0	148	156			PEAKS DB
total 8 peptides																					

OK005_J_302_Ferritin

back to list

| Protein Coverage | Supporting Peptides |

Protein Coverage:

1 MAHNVNQCRQ NYHAESEAGV NRQINMELYA SYCYQSMSFY FDRDDVALPG FAKFFKEASD EEREHAEKLM KFQNKRGGRV

81 VLQDIKKPDR DEWGTGLDAM QVALGLEKSV NQALLDLHEV AASHKDAQMT DFLEGEYLQE QVESIKKIGD HITNLKRVGT

161 GLGEYMFDKN LS

Supporting Peptides:

Peptide	Uniq	-10lgP	Mass	Length	ppm	m/z	z	RT	Fraction	Scan	Source File	Area ebraeus	Area judaeus	#Feature	#Feature ebraeus	#Feature judaeus	Start	End	PTM	AScore	Found By
K.DAQMTDFLEGEYLQEQVESIK.K	Y	116.93	2472.1365	21	13.6	1237.0872	2	57.77	2	F2:130150	20210611-09_2021021-OK160A_816_5.3.236.mgf		0	0	0	0	126	146			PEAKS DB
R.DEWGTGLDAMQVALGLEK.S	N	116.51	1931.9298	18	1.8	966.9699	2	59.74	2	F2:131980	20210611-09_2021021-OK160A_816_5.3.236.mgf		0	0	0	0	91	108			PEAKS DB
K.SVNQALLDLHEVAASHK.D	Y	115.94	1830.9587	17	2.0	611.3255	3	37.13	2	F2:96468	20210611-09_2021021-OK160A_816_5.3.236.mgf	0	0	0	0	0	109	125			PEAKS DB
R.QNYHAESEAGVNR.Q	N	96.97	1473.6597	13	1.1	737.8348	2	13.59	2	F2:56908	20210611-09_2021021-OK160A_816_5.3.236.mgf	0	0	0	0	0	10	22			PEAKS DB
R.Q(-17.03)NYHAESEAGVNR.Q	N	91.36	1456.6331	13	-0.3	729.3205	2	18.75	2	F2:64244	20210611-09_2021021-OK160A_816_5.3.236.mgf		0	0	0	0	10	22		Q1:Pyro-glu from Q:1000.00	PEAKS DB
R.DDVALPGFAK.F	N	90.93	1031.5287	10	2.5	516.7708	2	38.01	2	F2:97999	20210611-09_2021021-OK160A_816_5.3.236.mgf	0	0	0	0	0	44	53			PEAKS DB
R.VGTGLGEYMFDK.N	Y	89.63	1315.6118	12	0.0	658.8104	2	40.56	2	F2:102477	20210611-09_2021021-OK160A_816_5.3.236.mgf		0	0	0	0	158	169			PEAKS DB
K.IGDHITNLK.R	N	89.25	1009.5556	9	5.8	505.7859	2	19.85	2	F2:65725	20210611-09_2021021-OK160A_816_5.3.236.mgf		0	0	0	0	148	156			PEAKS DB
total 8 peptides																					

CebrOK035_J_351_Ferritin

back to list

| Protein Coverage | Supporting Peptides |

Protein Coverage:

1 MAHNVSQCRQ NYHAESEAGV NRQINMELYA SYCYQSMSFY FDRDDVALPG FAKFFKEASE EEREHAEKLM KFQNKRGGRV

81 VLQDIKKPDR DEWGTGLDAM QVALGLEKSV NQALLDLHEV AASHKDAQMT DFLEGEYLQE QVESIKKIGD HITNLKRVGT

161 GLGEYMFDKN LS

Supporting Peptides:

Peptide	Uniq	-10lgP	Mass	Length	ppm	m/z	z	RT	Fraction	Scan	Source File	Area ebraeus	Area judaeus	#Feature	#Feature ebraeus	#Feature judaeus	Start	End	PTM	AScore	Found By
K.DAQMTDFLEGEYLQEQVESIK.K	Y	116.93	2472.1365	21	13.6	1237.0872	2	57.77	2	F2:130150	20210611-09_2021021-OK160A_816_5.3.236.mgf		0	0	0	0	126	146			PEAKS DB
R.DEWGTGLDAMQVALGLEK.S	N	116.51	1931.9298	18	1.8	966.9699	2	59.74	2	F2:131980	20210611-09_2021021-OK160A_816_5.3.236.mgf		0	0	0	0	91	108			PEAKS DB
K.SVNQALLDLHEVAASHK.D	Y	115.94	1830.9587	17	2.0	611.3255	3	37.13	2	F2:96468	20210611-09_2021021-OK160A_816_5.3.236.mgf	0	0	0	0	0	109	125			PEAKS DB
R.QNYHAESEAGVNR.Q	N	96.97	1473.6597	13	1.1	737.8348	2	13.59	2	F2:56908	20210611-09_2021021-OK160A_816_5.3.236.mgf	0	0	0	0	0	10	22			PEAKS DB
R.Q(-17.03)NYHAESEAGVNR.Q	N	91.36	1456.6331	13	-0.3	729.3205	2	18.75	2	F2:64244	20210611-09_2021021-OK160A_816_5.3.236.mgf		0	0	0	0	10	22		Q1:Pyro-glu from Q:1000.00	PEAKS DB
R.DDVALPGFAK.F	N	90.93	1031.5287	10	2.5	516.7708	2	38.01	2	F2:97999	20210611-09_2021021-OK160A_816_5.3.236.mgf	0	0	0	0	0	44	53			PEAKS DB
R.VGTGLGEYMFDK.N	Y	89.63	1315.6118	12	0.0	658.8104	2	40.56	2	F2:102477	20210611-09_2021021-OK160A_816_5.3.236.mgf		0	0	0	0	158	169			PEAKS DB
K.IGDHITNLK.R	N	89.25	1009.5556	9	5.8	505.7859	2	19.85	2	F2:65725	20210611-09_2021021-OK160A_816_5.3.236.mgf		0	0	0	0	148	156			PEAKS DB
total 8 peptides																					

SRX1323887_E_233_Ferritin

back to list

| Protein Coverage | Supporting Peptides |

Protein Coverage:

1 MAHNVSQCRQ NYHAESEAGV NRQINMELYA SYCYQSMSFY FDRDDVALPG FAKFFKEASE EEREHAEKLM KFQNKRGGRV

81 VLQDIKKPDR DEWGTGLDAM QVALGLEKSV NQALLDLHEV AASHKDAQMT DFLEGEYLQE QVESIKKIGD HITNLKRVGT

161 GLGEYMFDKN LS

Supporting Peptides:

Peptide	Uniq	-10lgP	Mass	Length	ppm	m/z	z	RT	Fraction	Scan	Source File	Area ebraeus	Area judaeus	#Feature	#Feature ebraeus	#Feature judaeus	Start	End	PTM	AScore	Found By
K.DAQMTDFLEGEYLQEQVESIK.K	Y	116.93	2472.1365	21	13.6	1237.0872	2	57.77	2	F2:130150	20210611-09_2021021-OK160A_816_5.3.236.mgf		0	0	0	0	126	146			PEAKS DB
R.DEWGTGLDAMQVALGLEK.S	N	116.51	1931.9298	18	1.8	966.9699	2	59.74	2	F2:131980	20210611-09_2021021-OK160A_816_5.3.236.mgf		0	0	0	0	91	108			PEAKS DB
K.SVNQALLDLHEVAASHK.D	Y	115.94	1830.9587	17	2.0	611.3255	3	37.13	2	F2:96468	20210611-09_2021021-OK160A_816_5.3.236.mgf	0	0	0	0	0	109	125			PEAKS DB
R.QNYHAESEAGVNR.Q	N	96.97	1473.6597	13	1.1	737.8348	2	13.59	2	F2:56908	20210611-09_2021021-OK160A_816_5.3.236.mgf	0	0	0	0	0	10	22			PEAKS DB
R.Q(-17.03)NYHAESEAGVNR.Q	N	91.36	1456.6331	13	-0.3	729.3205	2	18.75	2	F2:64244	20210611-09_2021021-OK160A_816_5.3.236.mgf		0	0	0	0	10	22		Q1:Pyro-glu from Q:1000.00	PEAKS DB
R.DDVALPGFAK.F	N	90.93	1031.5287	10	2.5	516.7708	2	38.01	2	F2:97999	20210611-09_2021021-OK160A_816_5.3.236.mgf	0	0	0	0	0	44	53			PEAKS DB
R.VGTGLGEYMFDK.N	Y	89.63	1315.6118	12	0.0	658.8104	2	40.56	2	F2:102477	20210611-09_2021021-OK160A_816_5.3.236.mgf		0	0	0	0	158	169			PEAKS DB
K.IGDHITNLK.R	N	89.25	1009.5556	9	5.8	505.7859	2	19.85	2	F2:65725	20210611-09_2021021-OK160A_816_5.3.236.mgf		0	0	0	0	148	156			PEAKS DB
total 8 peptides																					

OK006_E_274_Ferritin

back to list

| Protein Coverage | Supporting Peptides |

Protein Coverage:

```
  1  MAHNVSQCRQ NYHAESEAGV NRQINMELYA SYCYQSMSFY FDRDDVALPG FAKFFKEASE EEREHAEKLM KFQNKRGGRV
 81  VLQDIKKPDR DEWGTGLDAM QVALGLEKSV NQALLDLHEV AASHKDAQMT DFLEGEYLQE QVESIKKIGD HITNLKRVGT
161  GLGEYMFDKN LS
```

Supporting Peptides:

Peptide	Uniq	-10lgP	Mass	Length	ppm	m/z	z	RT	Fraction	Scan	Source File	Area ebraeus	Area judaeus	#Feature	#Feature ebraeus	#Feature judaeus	Start	End	PTM	AScore	Found By
K.DAQMTDFLEGEYLQEQVESIK.K	Y	116.93	2472.1365	21	13.6	1237.0872	2	57.77	2	F2:130150	20210611-09_2021021-OK160A_816_5.3.236.mgf		0	0	0	0	126	146			PEAKS DB
R.DEWGTGLDAMQVALGLEK.S	N	116.51	1931.9298	18	1.8	966.9699	2	59.74	2	F2:131980	20210611-09_2021021-OK160A_816_5.3.236.mgf		0	0	0	0	91	108			PEAKS DB
K.SVNQALLDLHEVAASHK.D	Y	115.94	1830.9587	17	2.0	611.3255	3	37.13	2	F2:96468	20210611-09_2021021-OK160A_816_5.3.236.mgf	0	0	0	0	0	109	125			PEAKS DB
R.QNYHAESEAGVNR.Q	N	96.97	1473.6597	13	1.1	737.8348	2	13.59	2	F2:56908	20210611-09_2021021-OK160A_816_5.3.236.mgf	0	0	0	0	0	10	22			PEAKS DB
R.Q(-17.03)NYHAESEAGVNR.Q	N	91.36	1456.6331	13	-0.3	729.3205	2	18.75	2	F2:64244	20210611-09_2021021-OK160A_816_5.3.236.mgf		0	0	0	0	10	22		Q1:Pyro-glu from Q:1000.00	PEAKS DB
R.DDVALPGFAK.F	N	90.93	1031.5287	10	2.5	516.7708	2	38.01	2	F2:97999	20210611-09_2021021-OK160A_816_5.3.236.mgf	0	0	0	0	0	44	53			PEAKS DB
R.VGTGLGEYMFDK.N	Y	89.63	1315.6118	12	0.0	658.8104	2	40.56	2	F2:102477	20210611-09_2021021-OK160A_816_5.3.236.mgf		0	0	0	0	158	169			PEAKS DB
K.IGDHITNLK.R	N	89.25	1009.5556	9	5.8	505.7859	2	19.85	2	F2:65725	20210611-09_2021021-OK160A_816_5.3.236.mgf		0	0	0	0	148	156			PEAKS DB

total 8 peptides

OK193_E_251_Ferritin

back to list

| Protein Coverage | Supporting Peptides |

Protein Coverage:

```
  1  MAHNVSQCRQ NYHAESEAGV NRQINMELYA SYCYQSMSFY FDRDDVALPG FAKFFKEASE EEREHAEKLM KFQNKRGGRV
 81  VLQDIKKPDR DEWGTGLDAM QVALGLEKSV NQALLDLHEV AASHKDAQMT DFLEGEYLQE QVESIKKIGD HITNLKRVGT
161  GLGEYMFDKN LS
```

Supporting Peptides:

Peptide	Uniq	-10lgP	Mass	Length	ppm	m/z	z	RT	Fraction	Scan	Source File	Area ebraeus	Area judaeus	#Feature	#Feature ebraeus	#Feature judaeus	Start	End	PTM	AScore	Found By
K.DAQMTDFLEGEYLQEQVESIK.K	Y	116.93	2472.1365	21	13.6	1237.0872	2	57.77	2	F2:130150	20210611-09_2021021-OK160A_816_5.3.236.mgf		0	0	0	0	126	146			PEAKS DB
R.DEWGTGLDAMQVALGLEK.S	N	116.51	1931.9298	18	1.8	966.9699	2	59.74	2	F2:131980	20210611-09_2021021-OK160A_816_5.3.236.mgf		0	0	0	0	91	108			PEAKS DB
K.SVNQALLDLHEVAASHK.D	Y	115.94	1830.9587	17	2.0	611.3255	3	37.13	2	F2:96468	20210611-09_2021021-OK160A_816_5.3.236.mgf	0	0	0	0	0	109	125			PEAKS DB
R.QNYHAESEAGVNR.Q	N	96.97	1473.6597	13	1.1	737.8348	2	13.59	2	F2:56908	20210611-09_2021021-OK160A_816_5.3.236.mgf	0	0	0	0	0	10	22			PEAKS DB
R.Q(-17.03)NYHAESEAGVNR.Q	N	91.36	1456.6331	13	-0.3	729.3205	2	18.75	2	F2:64244	20210611-09_2021021-OK160A_816_5.3.236.mgf		0	0	0	0	10	22		Q1:Pyro-glu from Q:1000.00	PEAKS DB
R.DDVALPGFAK.F	N	90.93	1031.5287	10	2.5	516.7708	2	38.01	2	F2:97999	20210611-09_2021021-OK160A_816_5.3.236.mgf	0	0	0	0	0	44	53			PEAKS DB
R.VGTGLGEYMFDK.N	Y	89.63	1315.6118	12	0.0	658.8104	2	40.56	2	F2:102477	20210611-09_2021021-OK160A_816_5.3.236.mgf		0	0	0	0	158	169			PEAKS DB
K.IGDHITNLK.R	N	89.25	1009.5556	9	5.8	505.7859	2	19.85	2	F2:65725	20210611-09_2021021-OK160A_816_5.3.236.mgf		0	0	0	0	148	156			PEAKS DB

total 8 peptides

OK213_E_305_Ferritin

back to list

| Protein Coverage | Supporting Peptides |

Protein Coverage:

```
  1  MAHNVSQCRQ NYHAESEAGV NRQINMELYA SYCYQSMSFY FDRDDVALPG FAKFFKEASE EEREHAEKLM KFQNKRGGRV
 81  VLQDVKKPDR DEWGTGLDAM QVALGLEKSV NQALLDLHEV AASHKDAQMT DFLEGEYLQE QVESIKKIGD HITNLKRVGT
161  GLGEYMFDKN LS
```

Supporting Peptides:

Peptide	Uniq	-10lgP	Mass	Length	ppm	m/z	z	RT	Fraction	Scan	Source File	Area ebraeus	Area judaeus	#Feature	#Feature ebraeus	#Feature judaeus	Start	End	PTM	AScore	Found By
K.DAQMTDFLEGEYLQEQVESIK.K	Y	116.93	2472.1365	21	13.6	1237.0872	2	57.77	2	F2:130150	20210611-09_2021021-OK160A_816_5.3.236.mgf		0	0	0	0	126	146			PEAKS DB
R.DEWGTGLDAMQVALGLEK.S	N	116.51	1931.9298	18	1.8	966.9699	2	59.74	2	F2:131980	20210611-09_2021021-OK160A_816_5.3.236.mgf		0	0	0	0	91	108			PEAKS DB
K.SVNQALLDLHEVAASHK.D	Y	115.94	1830.9587	17	2.0	611.3255	3	37.13	2	F2:96468	20210611-09_2021021-OK160A_816_5.3.236.mgf	0	0	0	0	0	109	125			PEAKS DB
R.QNYHAESEAGVNR.Q	N	96.97	1473.6597	13	1.1	737.8348	2	13.59	2	F2:56908	20210611-09_2021021-OK160A_816_5.3.236.mgf	0	0	0	0	0	10	22			PEAKS DB
R.Q(-17.03)NYHAESEAGVNR.Q	N	91.36	1456.6331	13	-0.3	729.3205	2	18.75	2	F2:64244	20210611-09_2021021-OK160A_816_5.3.236.mgf		0	0	0	0	10	22		Q1:Pyro-glu from Q:1000.00	PEAKS DB
R.DDVALPGFAK.F	N	90.93	1031.5287	10	2.5	516.7708	2	38.01	2	F2:97999	20210611-09_2021021-OK160A_816_5.3.236.mgf	0	0	0	0	0	44	53			PEAKS DB
R.VGTGLGEYMFDK.N	Y	89.63	1315.6118	12	0.0	658.8104	2	40.56	2	F2:102477	20210611-09_2021021-OK160A_816_5.3.236.mgf		0	0	0	0	158	169			PEAKS DB
K.IGDHITNLK.R	N	89.25	1009.5556	9	5.8	505.7859	2	19.85	2	F2:65725	20210611-09_2021021-OK160A_816_5.3.236.mgf		0	0	0	0	148	156			PEAKS DB

total 8 peptides

CebrOK014_E_138_Ferritin

back to list

| Protein Coverage | Supporting Peptides |

Protein Coverage:

```
  1  MAHNVSQCRQ NYHAESEAGV NRQINMELYA SYCYQSMSFY FDRDDVALPG FAKFFKEASE EEREHAEKLM KFQNKRGGRV
 81  VLQDVKKPDR DEWGTGLDAM QVALGLEKSV NQALLDLHEV AASHKDAQMT DFLEGEYLQE QVESIKKIGD HITNLKRVGT
161  GLGEYMFDKN LS
```

Supporting Peptides:

Peptide	Uniq	-10lgP	Mass	Length	ppm	m/z	z	RT	Fraction	Scan	Source File	Area ebraeus	Area judaeus	#Feature	#Feature ebraeus	#Feature judaeus	Start	End	PTM	AScore	Found By
K.DAQMTDFLEGEYLQEQVESIK.K	Y	116.93	2472.1365	21	13.6	1237.0872	2	57.77	2	F2:130150	20210611-09_2021021-OK160A_816_5.3.236.mgf		0	0	0	0	126	146			PEAKS DB
R.DEWGTGLDAMQVALGLEK.S	N	116.51	1931.9298	18	1.8	966.9699	2	59.74	2	F2:131980	20210611-09_2021021-OK160A_816_5.3.236.mgf		0	0	0	0	91	108			PEAKS DB

total 8 peptides

Peptide	Uniq	-10lgP	Mass	Length	ppm	m/z	z	RT	Fraction	Scan	Source File	Area ebraeus	Area judaeus	#Feature	#Feature ebraeus	#Feature judaeus	Start	End	PTM	AScore	Found By
K.SVNQALLDLHEVAASHK.D	Y	115.94	1830.9587	17	2.0	611.3255	3	37.13	2	F2:96468	20210611-09_2021021-OK160A_816_5.3.236.mgf	0	0	0	0	0	109	125			PEAKS DB
R.QNYHAESEAGVNR.Q	N	96.97	1473.6597	13	1.1	737.8348	2	13.59	2	F2:56908	20210611-09_2021021-OK160A_816_5.3.236.mgf	0	0	0	0	0	10	22			PEAKS DB
R.Q(-17.03)NYHAESEAGVNR.Q	N	91.36	1456.6331	13	-0.3	729.3205	2	18.75	2	F2:64244	20210611-09_2021021-OK160A_816_5.3.236.mgf		0	0	0	0	10	22		Q1:Pyro-glu from Q:1000.00	PEAKS DB
R.DDVALPGFAK.F	N	90.93	1031.5287	10	2.5	516.7708	2	38.01	2	F2:97999	20210611-09_2021021-OK160A_816_5.3.236.mgf	0	0	0	0	0	44	53			PEAKS DB
R.VGTGLGEYMFDK.N	Y	89.63	1315.6118	12	0.0	658.8104	2	40.56	2	F2:102477	20210611-09_2021021-OK160A_816_5.3.236.mgf		0	0	0	0	158	169			PEAKS DB
K.IGDHITNLK.R	N	89.25	1009.5556	9	5.8	505.7859	2	19.85	2	F2:65725	20210611-09_2021021-OK160A_816_5.3.236.mgf		0	0	0	0	148	156			PEAKS DB
**total 8 peptides**																					

D_041_Thioredoxin

back to list

| Protein Coverage | Supporting Peptides |

Protein Coverage:

1 MAQIGQLAIQ KPAPEFSTQA VVNGEFQTVK LSQYRGKYVV LFFYPLDFTF VCPTEIIAFS DRVEEFRSIN CEVLGASTDS

C Carbamidomethylation (+57.02)
O Oxidation (M) (+15.99)

144 O

81 VFSHLAWVNT PR**KQGGLGNM NIPLLADK**TL EISRQYGCLK EDEGISYR**AL YIIDDK**GNLR **QITMNDLPVG R**SVDETLR**LV**

173 C

161 **QAFQFTDKHG EVCPAGWKPG SDTIKPTVK**D SKNYFTKAN

Supporting Peptides:

Peptide	Uniq	-10lgP	Mass	Length	ppm	m/z	z	RT	Fraction	Scan	Source File	Area ebraeus	Area judaeus	#Feature	#Feature ebraeus	#Feature judaeus	Start	End	PTM	AScore	Fo
K.HGEVC(+57.02)PAGWKPGSDTIKPTVK.D	Y	139.43	2263.1418	21	2.7	566.7919	4	26.70	2	F2:77717	20210611-09_2021021-OK160A_816_5.3.236.mgf		0	0	0	0	169	189	Carbamidomethylation	C5:Carbamidomethylation:1000.00	PE
K.QGGLGNMNIPLLADK.T	N	116.97	1539.8079	15	-0.5	770.9076	2	49.54	2	F2:118008	20210611-09_2021021-OK160A_816_5.3.236.mgf		0	0	0	0	94	108			PE
R.Q(-17.03)ITMNDLPVGR.S	N	105.67	1225.6125	11	-0.1	613.8109	2	46.97	2	F2:113637	20210611-09_2021021-OK160A_816_5.3.236.mgf		0	0	0	0	141	151		Q1:Pyro-glu from Q:1000.00	PE
R.QITMNDLPVGR.S	N	94.09	1242.6390	11	0.8	622.3250	2	36.64	1	F1:34092	20210611-08_2021021-OK192A_815_5.3.236.mgf	0	0	0	0	0	141	151			PE
R.QITM(+15.99)NDLPVGR.S	N	91.16	1258.6339	11	1.7	630.3226	2	27.61	2	F2:79161	20210611-09_2021021-OK160A_816_5.3.236.mgf		0	0	0	0	141	151	Oxidation (M)	M4:Oxidation (M):1000.00	PE
R.LVQAFQFTDK.H	N	90.87	1195.6237	10	0.1	598.8167	2	37.25	2	F2:96539	20210611-09_2021021-OK160A_816_5.3.236.mgf	0	0	0	0	0	159	168			PE
R.KQGGLGNMNIPLLADK.T	N	73.18	1667.9028	16	-0.3	556.9726	3	43.41	1	F1:41299	20210611-08_2021021-OK192A_815_5.3.236.mgf	0	0	0	0	0	93	108			PE
R.ALYIIDDK.G	N	72.37	949.5120	8	1.0	475.7617	2	33.17	2	F2:89147	20210611-09_2021021-OK160A_816_5.3.236.mgf	0	0	0	0	0	129	136			PE
**total 8 peptides**																					

D_085_FK506-binding

back to list

| Protein Coverage | Supporting Peptides |

Protein Coverage:

1 CVRPCVVCSL YLGRFRHVEF SVHFIPRQPR APVNLEEGST SSHTTHTCTS FLLRMSSQSR SVAVFAVGLL VCSLGFPSRA

P Pyro-glu from Q (-17.03)

81 DDEGELVREV LLKPDSCDRL TK**KHDLLSMH YTGTLTATGV K**FDSSHDRNQ PFEFQLGVGQ VIRGWEEGLL DMCVGEKRKL

161 VVPPNMAYGD QGAGEVIPPK ASLTFEVELM GIK**DGPPPPN VFK**EIDANED KLLSQDEVSS YLKDQQKRAD PSTPPVNDDQ

247 P

241 HNTMLK**QIFD HEDKDKDGFI SHDEFSGPK**H DEL

Supporting Peptides:

Peptide	Uniq	-10lgP	Mass	Length	ppm	m/z	z	RT	Fraction	Scan	Source File	Area ebraeus	Area judaeus	#Feature	#Feature ebraeus	#Feature judaeus	Start	End	PTM	AScore	Found By
K.DGFISHDEFSGPK.H	Y	116.11	1434.6415	13	-0.6	718.3246	2	31.32	2	F2:85481	20210611-09_2021021-OK160A_816_5.3.236.mgf	0	0	0	0	0	257	269			PEAKS DB
K.KHDLLSMHYTGTLTATGVK.F	Y	106.37	2072.0725	19	3.9	691.6979	3	33.21	2	F2:89037	20210611-09_2021021-OK160A_816_5.3.236.mgf		0	0	0	0	103	121			PEAKS DB
K.Q(-17.03)IFDHEDKDKDGFISHDEFSGPK.H	Y	102.63	2673.1982	23	-1.3	669.3032	4	36.92	2	F2:95954	20210611-09_2021021-OK160A_816_5.3.236.mgf		0	0	0	0	247	269		Q1:Pyro-glu from Q:1000.00	PEAKS DB
K.QIFDHEDKDKDGFISHDEFSGPK.H	Y	100.45	2690.2249	23	3.4	539.0518	5	30.99	2	F2:84921	20210611-09_2021021-OK160A_816_5.3.236.mgf		0	0	0	0	247	269			PEAKS DB
K.DGPPPPNVFK.E	Y	99.23	1066.5447	10	2.1	534.2785	2	29.14	2	F2:81685	20210611-09_2021021-OK160A_816_5.3.236.mgf	0	0	0	0	0	194	203			PEAKS DB
K.HDLLSMHYTGTLTATGVK.F	Y	91.23	1943.9775	18	0.2	648.9972	3	37.37	2	F2:96751	20210611-09_2021021-OK160A_816_5.3.236.mgf		0	0	0	0	104	121			PEAKS DB
K.KHDLLSM(+15.99)HYTGTLTATGVK.F	Y	83.04	2088.0674	19	-0.5	697.0265	3	28.81	2	F2:81290	20210611-09_2021021-OK160A_816_5.3.236.mgf	0	0	0	0	0	103	121		M7:Oxidation (M):1000.00	PEAKS DB
K.DKDGFISHDEFSGPK.H	Y	81.47	1677.7634	15	2.5	560.2608	3	27.88	2	F2:79578	20210611-09_2021021-OK160A_816_5.3.236.mgf		0	0	0	0	255	269			PEAKS DB
K.Q(-17.03)IFDHEDKDK.D	Y	74.23	1256.5673	10	1.0	419.8617	3	21.43	2	F2:68510	20210611-09_2021021-OK160A_816_5.3.236.mgf		0	0	0	0	247	256		Q1:Pyro-glu from Q:1000.00	PEAKS DB
K.Q(-17.03)IFDHEDK.D	Y	73.52	1013.4454	8	5.5	507.7306	2	25.88	2	F2:76140	20210611-09_2021021-OK160A_816_5.3.236.mgf	0	0	0	0	0	247	254	Pyro-glu from Q	Q1:Pyro-glu from Q:1000.00	PEAKS DB
K.QIFDHEDKDK.D	Y	67.29	1273.5939	10	1.9	637.8027	2	13.23	2	F2:56456	20210611-09_2021021-OK160A_816_5.3.236.mgf		0	0	0	0	247	256			PEAKS DB
K.Q(-17.03)IFDHEDKDK(-.98).D	Y	66.34	1255.5833	10	3.1	419.5345	3	20.44	2	F2:66639	20210611-09_2021021-OK160A_816_5.3.236.mgf	0	0	0	0	0	247	256	Pyro-glu from Q	Q1:Pyro-glu from Q:1000.00;K10:Amidation:1000.00	PEAKS DB
K.QIFDHEDK.D	Y	58.58	1030.4719	8	2.8	516.2425	2	16.00	2	F2:60116	20210611-09_2021021-OK160A_816_5.3.236.mgf	0	0	0	0	0	247	254			PEAKS DB
**total 13 peptides**																					

P_002D_003_Thioredoxin-like

back to list

| Protein Coverage | Supporting Peptides |

Protein Coverage:

Oxidation (M) (+15.99)

1 INLIDLAFFF KRLDWPRVMY KGRDVEETHP CEVGTKVGCV IVLHSPSAKG EADKIIDSLQ FDIHGMVGLI VEHVDDGPGG

81 SKVLDIVLLK QDGDTVTLNG SLELDQIEGE ITSHLLPASQ TKTGYHDEDY DFEPTNIDDM VVHMAYLERE HLADVPLLPA

161 LTDKTFPATT KDNKLAMVLF YLPFDAEGML FLKIFEDAGK KIAAMKGDDP KAHPLARVNC FDWTDVCRKE SVTTYPALYI

273

241 YRKGVKRDLY RLNLDVDIIV RTVMLLQAEQ PLMLTSEKEV NDFLSGQLPT EAYKDIDNLV LLKLSKSRKE TEMYIDVVKA

321 LEAQMLFAVV APDVTDLVPN GGVLHKRPKD PIQPMETLTA PRDAATIIEF LLKRQFALFP ELTFMNFPTL YARKQPFAIL

401 FYEPSVPSAN DMHQTIVSLL LSEKVHDMVF CKMTVGDKNI EAKTLLMNYT QTETVPAFTI VNHQKGQVYV LQDKDMKGSA

481 LLPWIQAVQK GEVAPTKILD KGEWKEPGQH YDFLALMDRE KEMDEEKKKA SEPATATPED TLEERQDVET RNALLELQRS

561 RLYSGKDRKM HKEAADTVTA TVKPKTSTDM GNGRKSPKHS EL

Supporting Peptides:

Peptide	Uniq	-10lgP	Mass	Length	ppm	m/z	z	RT	Fraction	Scan	Source File	Area ebraeus	Area judaeus	#Feature	#Feature ebraeus	#Feature judaeus	Start	End	PTM	AScore	Found By
R.TVMLLQAEQPLMLTSEK.E	Y	131.44	1931.0107	17	3.3	966.5118	2	51.75	2	F2:121721	20210611-09_2021021-OK160A_816_5.3.236.mgf		0	0	0	0	262	278			PEAKS DB
K.EAADTVTATVKPK.T	Y	107.01	1329.7140	13	-0.4	665.8612	2	18.99	2	F2:64388	20210611-09_2021021-OK160A_816_5.3.236.mgf		0	0	0	0	573	585			PEAKS DB
R.TVMLLQAEQPLM(+15.99)LTSEK.E	Y	98.91	1947.0056	17	0.7	974.5067	2	48.41	2	F2:115999	20210611-09_2021021-OK160A_816_5.3.236.mgf		0	0	0	0	262	278	Oxidation (M)	M12:Oxidation (M):123.34	PEAKS DB
K.DPIQPM(+15.99)ETLTAPR.D	Y	97.50	1483.7340	13	-1.4	742.8701	2	30.81	2	F2:84833	20210611-09_2021021-OK160A_816_5.3.236.mgf		0	0	0	0	350	362		M6:Oxidation (M):1000.00	PEAKS DB
R.E(-18.01)HLADVPLLPALTDK.T	Y	95.20	1612.8824	15	-1.0	807.4443	2	54.26	2	F2:125340	20210611-09_2021021-OK160A_816_5.3.236.mgf		0	0	0	0	150	164		E1:Pyro-glu from E:1000.00	PEAKS DB
K.E(-18.01)AADTVTATVKPK.T	Y	94.26	1311.7034	13	0.0	438.2399	3	19.03	2	F2:64574	20210611-09_2021021-OK160A_816_5.3.236.mgf		0	0	0	0	573	585		E1:Pyro-glu from E:1000.00	PEAKS DB
R.TVM(+15.99)LLQAEQPLM(+15.99)LTSEK.E	Y	92.97	1963.0006	17	5.8	982.5092	2	44.91	2	F2:110344	20210611-09_2021021-OK160A_816_5.3.236.mgf		0	0	0	0	262	278		M3:Oxidation (M):1000.00;M12:Oxidation (M):1000.00	PEAKS DB
K.GQVYVLQDK.D	Y	75.89	1048.5553	9	4.1	525.2849	2	25.34	2	F2:75184	20210611-09_2021021-OK160A_816_5.3.236.mgf	0	0	0	0	0	466	474			PEAKS DB
K.GQVYVLQDKDM(+15.99)K.G	Y	73.79	1438.7126	12	-1.8	720.3593	2	21.10	2	F2:67788	20210611-09_2021021-OK160A_816_5.3.236.mgf		0	0	0	0	466	477		M11:Oxidation (M):1000.00	PEAKS DB
INLIDLAFFFKR.L	Y	58.40	1495.8551	12	-11.7	748.9229	2	45.08	2	F2:110565	20210611-09_2021021-OK160A_816_5.3.236.mgf		0	0	0	0	1	12			PEAKS DB
**total 10 peptides**																					

K_0010_152_Protein

back to list

| Protein Coverage | Supporting Peptides |

Protein Coverage:

1 MLLAYFRPSA FTSPATMRRA AILFSCIVLA VQASDVLEFT DSNFKSKIKE LDVALVEFYA PWCGHCKRLA PEYEVAATKL

81 MKSDPPVPLV KVDCTAESET CKEYGVSGYP TLKIFKGGEF AKDYQGPRES KGIVNTMQKE AGPASKELED FKQYEDFVGR

161 DTVCVVGFFA DADSEMAKTF SKLADTHEGD IRFGHTTASA IMDKANYKDE IVLFRPKAMQ NKFEDSAVKF TGDATKKPAI

241 KEFLTQEHLG MCGIRTMNNA GDFGKPTFVA FYNVDYVKNP KGSNYWRNRV MKVGKKLCDE GLKVFFGVSN KEEHDQELGE

321 CGLQNAEGDK PIVCAYDEKN RKFKMEEEFS VDSFEKFVRG VLNGEVEAYL KSEPVPENND QPVKVVVAKN FDDLVNDPEK

401 DVLIEFYAPW CGHCKSLAPK YDELAEKLKD ETDIVIAKMD ATANDPPSQY EVRGFPTIYF APKGSKSSPK KYEGGREVND

481 FIKYLAKEAT DELQGFDRKG KKKGGQKKKT EL

Supporting Peptides:

Peptide	Uniq	-10lgP	Mass	Length	ppm	m/z	z	RT	Fraction	Scan	Source File	Area ebraeus	Area judaeus	#Feature	#Feature ebraeus	#Feature judaeus	Start	End	PTM	AScore	Found By
K.M(+15.99)DATANDPPSQYEVR.G	N	116.71	1708.7362	15	-0.3	855.3715	2	23.22	2	F2:71559	20210611-09_2021021-OK160A_816_5.3.236.mgf		0	0	0	0	439	453		M1:Oxidation (M):1000.00	PEAKS DB
K.SEPVPENNDQPVK.V	N	112.89	1451.6892	13	2.8	726.8509	2	19.00	2	F2:64401	20210611-09_2021021-OK160A_816_5.3.236.mgf	0	0	0	0	0	372	384			PEAKS DB
K.MDATANDPPSQYEVR.G	N	106.16	1692.7413	15	0.4	847.3748	2	26.03	2	F2:76318	20210611-09_2021021-OK160A_816_5.3.236.mgf		0	0	0	0	439	453			PEAKS DB
K.EATDELQGFDR.K	N	95.64	1279.5680	11	0.1	640.7886	2	29.87	2	F2:83300	20210611-09_2021021-OK160A_816_5.3.236.mgf	0	0	0	0	0	488	498			PEAKS DB
R.GFPTIYFAPK.G	N	86.32	1139.6014	10	-0.9	570.8051	2	46.08	2	F2:112594	20210611-09_2021021-OK160A_816_5.3.236.mgf	0	0	0	0	0	454	463			PEAKS DB
K.LKDETDIVIAK.M	N	82.41	1243.7023	11	0.0	622.8558	2	25.53	2	F2:75618	20210611-09_2021021-OK160A_816_5.3.236.mgf		0	0	0	0	428	438			PEAKS DB
K.VFFGVSNK.E	N	77.53	896.4756	8	2.5	449.2443	2	30.55	2	F2:84259	20210611-09_2021021-OK160A_816_5.3.236.mgf	0	0	0	0	0	304	311			PEAKS DB
K.NFDDLVNDPEK.D	Y	77.42	1304.5884	11	2.9	653.3006	2	33.46	2	F2:89357	20210611-09_2021021-OK160A_816_5.3.236.mgf		0	0	0	0	390	400			PEAKS DB
K.YDELAEK.L	N	64.80	866.4021	7	4.6	434.2085	2	16.53	2	F2:60959	20210611-09_2021021-OK160A_816_5.3.236.mgf		0	0	0	0	421	427			PEAKS DB
**total 9 peptides**																					

CebrOK035_J_332_Tpra06

back to list

| Protein Coverage | Supporting Peptides |

Protein Coverage:

44

1 QFCPTMTDSR PGENKCLHDN YLCGKEDDSG SCTSLCHCKS GQMCFRDNDH TITAVPRIIN DRPVEITYYT CVALSSLNEC

Carbamidomethylation (+57.02)

81 SGIQEALKVL SPEAEEGESS VEVLCRCPSP KKYRFVFIRP DINGDNPRYV CANAP

Supporting Peptides:

Peptide	Uniq	-10lgP	Mass	Length	ppm	m/z	z	RT	Fraction	Scan	Source File	Area ebraeus	Area judaeus	#Feature	#Feature ebraeus	#Feature judaeus	Start	End	PTM	AScore	Four By
K.VLSPEAEEGESSVEVLC(+57.02)R.C	Y	135.66	1988.9360	18	1.3	995.4724	2	37.98	2	F2:97922	20210611-09_2021021-OK160A_816_5.3.236.mgf		0	0	0	0	89	106		C17:Carbamidomethylation:1000.00	PEAK DB
R.FVFIRPDINGDNPR.Y	Y	120.21	1658.8528	14	0.3	553.9561	3	38.51	2	F2:99034	20210611-09_2021021-OK160A_816_5.3.236.mgf		0	0	0	0	115	128			PEAK DB
R.DNDHTITAVPR.I	Y	112.12	1237.6051	11	0.6	619.8076	2	20.41	2	F2:66510	20210611-09_2021021-OK160A_816_5.3.236.mgf		0	0	0	0	47	57			PEAK DB

total 5 peptides

Peptide	Uniq	-10lgP	Mass	Length	ppm	m/z	z	RT	Fraction	Scan	Source File	Area ebraeus	Area judaeus	#Feature	#Feature ebraeus	#Feature judaeus	Start	End	PTM	AScore	Four By
Q(-17.03)FC(+57.02)PTMTDSRPGENK.C	Y	94.46	1749.7450	15	1.6	875.8775	2	31.08	2	F2:85086	20210611-09_2021021-OK160A_816_5.3.236.mgf		0	0	0	0	1	15		Q1:Pyro-glu from Q:1000.00; C3:Carbamidomethylation:1000.00	PEAK DB
K.SGQMC(+57.02)FR.D	Y	69.48	884.3633	7	4.7	443.1891	2	19.22	2	F2:64755	20210611-09_2021021-OK160A_816_5.3.236.mgf		0	0	0	0	40	46	Carbamidomethylation	C5:Carbamidomethylation:1000.00	PEAK DB

total 5 peptides

OK212_J_274_Tpra06

back to list

| Protein Coverage | Supporting Peptides |

Protein Coverage:

44 C

1 **QFCPTMTDSR PGENK**CLHDN YLCGKEDDSG SCTSLCYCK**S GQMCFRDNDH TITAVPR**IIN DRPVEITYYT CVALSSLNEC

81 SGIQEALK**VL SPEAEEGESS VEVLCR**CPSP KKYR**FVFIRP DINGDNPR**YV CANAP

C Carbamidomethylation (+57.02)

Supporting Peptides:

Peptide	Uniq	-10lgP	Mass	Length	ppm	m/z	z	RT	Fraction	Scan	Source File	Area ebraeus	Area judaeus	#Feature	#Feature ebraeus	#Feature judaeus	Start	End	PTM	AScore	Four By
K.VLSPEAEEGESSVEVLC(+57.02)R.C	Y	135.66	1988.9360	18	1.3	995.4724	2	37.98	2	F2:97922	20210611-09_2021021-OK160A_816_5.3.236.mgf		0	0	0	0	89	106		C17:Carbamidomethylation:1000.00	PEAK DB
R.FVFIRPDINGDNPR.Y	Y	120.21	1658.8528	14	0.3	553.9561	3	38.51	2	F2:99034	20210611-09_2021021-OK160A_816_5.3.236.mgf		0	0	0	0	115	128			PEAK DB
R.DNDHTITAVPR.I	Y	112.12	1237.6051	11	0.6	619.8076	2	20.41	2	F2:66510	20210611-09_2021021-OK160A_816_5.3.236.mgf		0	0	0	0	47	57			PEAK DB
Q(-17.03)FC(+57.02)PTMTDSRPGENK.C	Y	94.46	1749.7450	15	1.6	875.8775	2	31.08	2	F2:85086	20210611-09_2021021-OK160A_816_5.3.236.mgf		0	0	0	0	1	15		Q1:Pyro-glu from Q:1000.00; C3:Carbamidomethylation:1000.00	PEAK DB
K.SGQMC(+57.02)FR.D	Y	69.48	884.3633	7	4.7	443.1891	2	19.22	2	F2:64755	20210611-09_2021021-OK160A_816_5.3.236.mgf		0	0	0	0	40	46	Carbamidomethylation	C5:Carbamidomethylation:1000.00	PEAK DB

total 5 peptides

CebrOK033_J_231_Tpra06

back to list

| Protein Coverage | Supporting Peptides |

Protein Coverage:

44 C

1 **QFCPTMTDSR PGENK**CLHDN YLCGKEDDSG SCTSLCYCK**S GQMCFRDNDH TITAVPR**IIN DRPVEITYYT CVALSSLNEC

81 SGIQEALK**VL SPEAEEGESS VEVLCR**CPSP KKYR**FVFIRP DINGDNPR**YV CANAP

C Carbamidomethylation (+57.02)

Supporting Peptides:

Peptide	Uniq	-10lgP	Mass	Length	ppm	m/z	z	RT	Fraction	Scan	Source File	Area ebraeus	Area judaeus	#Feature	#Feature ebraeus	#Feature judaeus	Start	End	PTM	AScore	Four By
K.VLSPEAEEGESSVEVLC(+57.02)R.C	Y	135.66	1988.9360	18	1.3	995.4724	2	37.98	2	F2:97922	20210611-09_2021021-OK160A_816_5.3.236.mgf		0	0	0	0	89	106		C17:Carbamidomethylation:1000.00	PEAK DB
R.FVFIRPDINGDNPR.Y	Y	120.21	1658.8528	14	0.3	553.9561	3	38.51	2	F2:99034	20210611-09_2021021-OK160A_816_5.3.236.mgf		0	0	0	0	115	128			PEAK DB
R.DNDHTITAVPR.I	Y	112.12	1237.6051	11	0.6	619.8076	2	20.41	2	F2:66510	20210611-09_2021021-OK160A_816_5.3.236.mgf		0	0	0	0	47	57			PEAK DB
Q(-17.03)FC(+57.02)PTMTDSRPGENK.C	Y	94.46	1749.7450	15	1.6	875.8775	2	31.08	2	F2:85086	20210611-09_2021021-OK160A_816_5.3.236.mgf		0	0	0	0	1	15		Q1:Pyro-glu from Q:1000.00; C3:Carbamidomethylation:1000.00	PEAK DB
K.SGQMC(+57.02)FR.D	Y	69.48	884.3633	7	4.7	443.1891	2	19.22	2	F2:64755	20210611-09_2021021-OK160A_816_5.3.236.mgf		0	0	0	0	40	46	Carbamidomethylation	C5:Carbamidomethylation:1000.00	PEAK DB

total 5 peptides

CebrOK016_E_148_Ferritin

back to list

| Protein Coverage | Supporting Peptides |

Protein Coverage:

14 C

1 MAVSQCR**QNY HAECEAGINR** QINMELHASY CYQSMAFYFD R**DDVALPGFA K**FFRKSSEEE REHAEK**LMTF QNQR**GGR**IVL**

81 **QDIK**KPDR**DE WGSGLDAMQV ALALEK**SVNQ SLLDLHEVAT NHNDAQMTDF LEGNYLQEQV RSIK**ELGDYI TNLK**R**VGPGL**

161 **GEYMFDKETL QE**

C Carbamidomethylation (+57.02)

Supporting Peptides:

Peptide	Uniq	-10lgP	Mass	Length	ppm	m/z	z	RT	Fraction	Scan	Source File	Area ebraeus	Area judaeus	#Feature	#Feature ebraeus	#Feature judaeus	Start	End	PTM	AScore	Found By
R.DEWGSGLDAMQVALALEK.S	Y	119.77	1931.9298	18	2.9	966.9709	2	59.33	2	F2:131653	20210611-09_2021021-OK160A_816_5.3.236.mgf		0	0	0	0	89	106			PEAKS DB
R.DDVALPGFAK.F	N	90.93	1031.5287	10	2.5	516.7708	2	38.01	2	F2:97999	20210611-09_2021021-OK160A_816_5.3.236.mgf	0	0	0	0	0	42	51			PEAKS DB
R.VGPGLGEYMFDK.E	Y	89.25	1311.6169	12	2.3	656.8145	2	42.16	2	F2:105049	20210611-09_2021021-OK160A_816_5.3.236.mgf		0	0	0	0	156	167			PEAKS DB
K.ELGDYITNLK.R	Y	84.29	1164.6027	10	2.4	583.3076	2	39.25	2	F2:100121	20210611-09_2021021-OK160A_816_5.3.236.mgf		0	0	0	0	145	154			PEAKS DB
R.VGPGLGEYMFDKETLQE	Y	80.78	1911.8923	17	3.6	956.9529	2	47.16	2	F2:113799	20210611-09_2021021-OK160A_816_5.3.236.mgf		0	0	0	0	156	172			PEAKS DB
R.QNYHAEC(+57.02)EAGINR.Q	Y	69.96	1560.6740	13	2.9	521.2313	3	16.74	2	F2:61108	20210611-09_2021021-OK160A_816_5.3.236.mgf		0	0	0	0	8	20	Carbamidomethylation	C7:Carbamidomethylation:1000.00	PEAKS DB

total 8 peptides

Peptide	Uniq	-10lgP	Mass	Length	ppm	m/z	z	RT	Fraction	Scan	Source File	Area ebraeus	Area judaeus	#Feature	#Feature ebraeus	#Feature judaeus	Start	End	PTM	AScore	Found By
K.LMTFQNQR.G	Y	63.53	1036.5125	8	1.5	519.2621	2	24.34	2	F2:73402	20210611-09_2021021-OK160A_816_5.3.236.mgf		0	0	0	0	67	74			PEAKS DB
R.IVLQDIK.K	N	58.66	827.5116	7	-0.5	414.7611	2	28.93	2	F2:81450	20210611-09_2021021-OK160A_816_5.3.236.mgf		0	0	0	0	78	84			PEAKS DB
total 8 peptides																					

OK213_E_304_Ferritin

back to list

| Protein Coverage | Supporting Peptides |

Protein Coverage:

14 C

1 MAVSQCRQNY HAECEAGINR QINMELHASY CYQSMAFYFD RDDVALPGFA KFFRKSSEEE REHAEKLMTF QNQRGGRIVL

C Carbamidomethylation (+57.02)

81 QDIKKPDRDE WGSGLDAMQV ALALEKSVNQ SLLDLHEVAT NHNDAQMTDF LEGNYLQEQV RSIKELGDYI TNLKRVGPGL

161 GEYMFDKETL QE

Supporting Peptides:

Peptide	Uniq	-10lgP	Mass	Length	ppm	m/z	z	RT	Fraction	Scan	Source File	Area ebraeus	Area judaeus	#Feature	#Feature ebraeus	#Feature judaeus	Start	End	PTM	AScore	Found By
R.DEWGSGLDAMQVALALEK.S	Y	119.77	1931.9298	18	2.9	966.9709	2	59.33	2	F2:131653	20210611-09_2021021-OK160A_816_5.3.236.mgf		0	0	0	0	89	106			PEAKS DB
R.DDVALPGFAK.F	N	90.93	1031.5287	10	2.5	516.7708	2	38.01	2	F2:97999	20210611-09_2021021-OK160A_816_5.3.236.mgf	0	0	0	0	0	42	51			PEAKS DB
R.VGPGLGEYMFDK.E	Y	89.25	1311.6169	12	2.3	656.8145	2	42.16	2	F2:105049	20210611-09_2021021-OK160A_816_5.3.236.mgf		0	0	0	0	156	167			PEAKS DB
K.ELGDYITNLK.R	Y	84.29	1164.6027	10	2.4	583.3076	2	39.25	2	F2:100121	20210611-09_2021021-OK160A_816_5.3.236.mgf		0	0	0	0	145	154			PEAKS DB
R.VGPGLGEYMFDKETLQE	Y	80.78	1911.8923	17	3.6	956.9529	2	47.16	2	F2:113799	20210611-09_2021021-OK160A_816_5.3.236.mgf		0	0	0	0	156	172			PEAKS DB
R.QNYHAEC(+57.02)EAGINR.Q	Y	69.96	1560.6740	13	2.9	521.2313	3	16.74	2	F2:61108	20210611-09_2021021-OK160A_816_5.3.236.mgf		0	0	0	0	8	20	Carbamidomethylation	C7:Carbamidomethylation:1000.00	PEAKS DB
K.LMTFQNQR.G	Y	63.53	1036.5125	8	1.5	519.2621	2	24.34	2	F2:73402	20210611-09_2021021-OK160A_816_5.3.236.mgf		0	0	0	0	67	74			PEAKS DB
R.IVLQDIK.K	N	58.66	827.5116	7	-0.5	414.7611	2	28.93	2	F2:81450	20210611-09_2021021-OK160A_816_5.3.236.mgf		0	0	0	0	78	84			PEAKS DB
total 8 peptides																					

OK006_E_273_Ferritin

back to list

| Protein Coverage | Supporting Peptides |

Protein Coverage:

14 C

1 MAVSQCRQNY HAECEAGINR QINMELHASY CYQSMAFYFD RDDVALPGFA KFFRKSSEEE REHAEKLMTF QNQRGGRIVL

C Carbamidomethylation (+57.02)

81 QDIKKPDRDE WGSGLDAMQV ALALEKSVNQ SLLDLHEVAT NHNDAQMTDF LEGNYLQEQV RSIKELGDYI TNLKRVGPGL

161 GEYMFDKETL QE

Supporting Peptides:

Peptide	Uniq	-10lgP	Mass	Length	ppm	m/z	z	RT	Fraction	Scan	Source File	Area ebraeus	Area judaeus	#Feature	#Feature ebraeus	#Feature judaeus	Start	End	PTM	AScore	Found By
R.DEWGSGLDAMQVALALEK.S	Y	119.77	1931.9298	18	2.9	966.9709	2	59.33	2	F2:131653	20210611-09_2021021-OK160A_816_5.3.236.mgf		0	0	0	0	89	106			PEAKS DB
R.DDVALPGFAK.F	N	90.93	1031.5287	10	2.5	516.7708	2	38.01	2	F2:97999	20210611-09_2021021-OK160A_816_5.3.236.mgf	0	0	0	0	0	42	51			PEAKS DB
R.VGPGLGEYMFDK.E	Y	89.25	1311.6169	12	2.3	656.8145	2	42.16	2	F2:105049	20210611-09_2021021-OK160A_816_5.3.236.mgf		0	0	0	0	156	167			PEAKS DB
K.ELGDYITNLK.R	Y	84.29	1164.6027	10	2.4	583.3076	2	39.25	2	F2:100121	20210611-09_2021021-OK160A_816_5.3.236.mgf		0	0	0	0	145	154			PEAKS DB
R.VGPGLGEYMFDKETLQE	Y	80.78	1911.8923	17	3.6	956.9529	2	47.16	2	F2:113799	20210611-09_2021021-OK160A_816_5.3.236.mgf		0	0	0	0	156	172			PEAKS DB
R.QNYHAEC(+57.02)EAGINR.Q	Y	69.96	1560.6740	13	2.9	521.2313	3	16.74	2	F2:61108	20210611-09_2021021-OK160A_816_5.3.236.mgf		0	0	0	0	8	20	Carbamidomethylation	C7:Carbamidomethylation:1000.00	PEAKS DB
K.LMTFQNQR.G	Y	63.53	1036.5125	8	1.5	519.2621	2	24.34	2	F2:73402	20210611-09_2021021-OK160A_816_5.3.236.mgf		0	0	0	0	67	74			PEAKS DB
R.IVLQDIK.K	N	58.66	827.5116	7	-0.5	414.7611	2	28.93	2	F2:81450	20210611-09_2021021-OK160A_816_5.3.236.mgf		0	0	0	0	78	84			PEAKS DB
total 8 peptides																					

OK005_J_301_Ferritin

back to list

| Protein Coverage | Supporting Peptides |

Protein Coverage:

14 C

1 MAVSQCRQNY HAECEAGINR QINMELHASY CYQSMAFYFD RDDVALPGFA KFFRKSSEEE REHAEKLMTF QNQRGGRIVL

C Carbamidomethylation (+57.02)

81 QDIKKPDRDE WGSGLDAMQV ALALEKSVNQ SLLDLHEVAT NHNDAQMTDF LEGNYLQEQV RSIKELGDYI TNLKRVGPGL

161 GEYMFDKETL QE

Supporting Peptides:

Peptide	Uniq	-10lgP	Mass	Length	ppm	m/z	z	RT	Fraction	Scan	Source File	Area ebraeus	Area judaeus	#Feature	#Feature ebraeus	#Feature judaeus	Start	End	PTM	AScore	Found By
R.DEWGSGLDAMQVALALEK.S	Y	119.77	1931.9298	18	2.9	966.9709	2	59.33	2	F2:131653	20210611-09_2021021-OK160A_816_5.3.236.mgf		0	0	0	0	89	106			PEAKS DB
R.DDVALPGFAK.F	N	90.93	1031.5287	10	2.5	516.7708	2	38.01	2	F2:97999	20210611-09_2021021-OK160A_816_5.3.236.mgf	0	0	0	0	0	42	51			PEAKS DB
R.VGPGLGEYMFDK.E	Y	89.25	1311.6169	12	2.3	656.8145	2	42.16	2	F2:105049	20210611-09_2021021-OK160A_816_5.3.236.mgf		0	0	0	0	156	167			PEAKS DB
K.ELGDYITNLK.R	Y	84.29	1164.6027	10	2.4	583.3076	2	39.25	2	F2:100121	20210611-09_2021021-OK160A_816_5.3.236.mgf		0	0	0	0	145	154			PEAKS DB
R.VGPGLGEYMFDKETLQE	Y	80.78	1911.8923	17	3.6	956.9529	2	47.16	2	F2:113799	20210611-09_2021021-OK160A_816_5.3.236.mgf		0	0	0	0	156	172			PEAKS DB
R.QNYHAEC(+57.02)EAGINR.Q	Y	69.96	1560.6740	13	2.9	521.2313	3	16.74	2	F2:61108	20210611-09_2021021-OK160A_816_5.3.236.mgf		0	0	0	0	8	20	Carbamidomethylation	C7:Carbamidomethylation:1000.00	PEAKS DB
K.LMTFQNQR.G	Y	63.53	1036.5125	8	1.5	519.2621	2	24.34	2	F2:73402	20210611-09_2021021-OK160A_816_5.3.236.mgf		0	0	0	0	67	74			PEAKS DB
R.IVLQDIK.K	N	58.66	827.5116	7	-0.5	414.7611	2	28.93	2	F2:81450	20210611-09_2021021-OK160A_816_5.3.236.mgf		0	0	0	0	78	84			PEAKS DB
total 8 peptides																					

OK212_J_294_Ferritin

back to list

| Protein Coverage | Supporting Peptides |

Protein Coverage:

```
1   MAVSQCRQNY HAECEAGINR QINMELHASY CYQSMAFYFD RDDVALPGFA KFFRKSSEEE REHAEKLMTF QNQRGGRIVL
81  QDIKKPDRDE WGSGLDAMQV ALALEKSVNQ SLLDLHEVAT NHNDAQMTDF LEGNYLQEQV RSIKELGDYI TNLKRVGPGL
161 GEYMFDKETL QE
```

C (14): Carbamidomethylation (+57.02)

Supporting Peptides:

Peptide	Uniq	-10lgP	Mass	Length	ppm	m/z	z	RT	Fraction	Scan	Source File	Area ebraeus	Area judaeus	#Feature	#Feature ebraeus	#Feature judaeus	Start	End	PTM	AScore	Found By
R.DEWGSGLDAMQVALALEK.S	Y	119.77	1931.9298	18	2.9	966.9709	2	59.33	2	F2:131653	20210611-09_2021021-OK160A_816_5.3.236.mgf		0	0	0	0	89	106			PEAKS DB
R.DDVALPGFAK.F	N	90.93	1031.5287	10	2.5	516.7708	2	38.01	2	F2:97999	20210611-09_2021021-OK160A_816_5.3.236.mgf	0	0	0	0	0	42	51			PEAKS DB
R.VGPGLGEYMFDK.E	Y	89.25	1311.6169	12	2.3	656.8145	2	42.16	2	F2:105049	20210611-09_2021021-OK160A_816_5.3.236.mgf		0	0	0	0	156	167			PEAKS DB
K.ELGDYITNLK.R	Y	84.29	1164.6027	10	2.4	583.3076	2	39.25	2	F2:100121	20210611-09_2021021-OK160A_816_5.3.236.mgf		0	0	0	0	145	154			PEAKS DB
R.VGPGLGEYMFDKETLQE	Y	80.78	1911.8923	17	3.6	956.9529	2	47.16	2	F2:113799	20210611-09_2021021-OK160A_816_5.3.236.mgf		0	0	0	0	156	172			PEAKS DB
R.QNYHAEC(+57.02)EAGINR.Q	Y	69.96	1560.6740	13	2.9	521.2313	3	16.74	2	F2:61108	20210611-09_2021021-OK160A_816_5.3.236.mgf		0	0	0	0	8	20	Carbamidomethylation	C7:Carbamidomethylation:1000.00	PEAKS DB
K.LMTFQNQR.G	Y	63.53	1036.5125	8	1.5	519.2621	2	24.34	2	F2:73402	20210611-09_2021021-OK160A_816_5.3.236.mgf		0	0	0	0	67	74			PEAKS DB
R.IVLQDIK.K	N	58.66	827.5116	7	-0.5	414.7611	2	28.93	2	F2:81450	20210611-09_2021021-OK160A_816_5.3.236.mgf		0	0	0	0	78	84			PEAKS DB

total 8 peptides

OK193_E_250_Ferritin

back to list

| Protein Coverage | Supporting Peptides |

Protein Coverage:

```
1   MAVSQCRQNY HAECEAGINR QINMELHASY CYQSMAFYFD RDDVALPGFA KFFRKSSEEE REHAEKLMTF QNQRGGRIVL
81  QDIKKPDRDE WGSGLDAMQV ALALEKSVNQ SLLDLHEVAT NHNDAQMTDF LEGNYLQEQV RSIKELGDYI TNLKRVGPGL
161 GEYMFDKETL QE
```

C (14): Carbamidomethylation (+57.02)

Supporting Peptides:

Peptide	Uniq	-10lgP	Mass	Length	ppm	m/z	z	RT	Fraction	Scan	Source File	Area ebraeus	Area judaeus	#Feature	#Feature ebraeus	#Feature judaeus	Start	End	PTM	AScore	Found By
R.DEWGSGLDAMQVALALEK.S	Y	119.77	1931.9298	18	2.9	966.9709	2	59.33	2	F2:131653	20210611-09_2021021-OK160A_816_5.3.236.mgf		0	0	0	0	89	106			PEAKS DB
R.DDVALPGFAK.F	N	90.93	1031.5287	10	2.5	516.7708	2	38.01	2	F2:97999	20210611-09_2021021-OK160A_816_5.3.236.mgf	0	0	0	0	0	42	51			PEAKS DB
R.VGPGLGEYMFDK.E	Y	89.25	1311.6169	12	2.3	656.8145	2	42.16	2	F2:105049	20210611-09_2021021-OK160A_816_5.3.236.mgf		0	0	0	0	156	167			PEAKS DB
K.ELGDYITNLK.R	Y	84.29	1164.6027	10	2.4	583.3076	2	39.25	2	F2:100121	20210611-09_2021021-OK160A_816_5.3.236.mgf		0	0	0	0	145	154			PEAKS DB
R.VGPGLGEYMFDKETLQE	Y	80.78	1911.8923	17	3.6	956.9529	2	47.16	2	F2:113799	20210611-09_2021021-OK160A_816_5.3.236.mgf		0	0	0	0	156	172			PEAKS DB
R.QNYHAEC(+57.02)EAGINR.Q	Y	69.96	1560.6740	13	2.9	521.2313	3	16.74	2	F2:61108	20210611-09_2021021-OK160A_816_5.3.236.mgf		0	0	0	0	8	20	Carbamidomethylation	C7:Carbamidomethylation:1000.00	PEAKS DB
K.LMTFQNQR.G	Y	63.53	1036.5125	8	1.5	519.2621	2	24.34	2	F2:73402	20210611-09_2021021-OK160A_816_5.3.236.mgf		0	0	0	0	67	74			PEAKS DB
R.IVLQDIK.K	N	58.66	827.5116	7	-0.5	414.7611	2	28.93	2	F2:81450	20210611-09_2021021-OK160A_816_5.3.236.mgf		0	0	0	0	78	84			PEAKS DB

total 8 peptides

K_0010_064_Ferritin

back to list

| Protein Coverage | Supporting Peptides |

Protein Coverage:

```
1   MAVSQCRQNY HAECEAGINR QINMELHASY CYQSMAFYFD RDDVALPGFA KFFRKSSEEE REHAEKLMTF QNQRGGRIVL
81  QDIKKPDRDE WGSGLDAMQV ALALEKSVNQ SLLELHDVAT NHNDAQMTDF LEGNYLQEQV RSIKELGDYI TNLKRVGPGL
161 GEYMFDKETL QE
```

C (14): Carbamidomethylation (+57.02)

Supporting Peptides:

Peptide	Uniq	-10lgP	Mass	Length	ppm	m/z	z	RT	Fraction	Scan	Source File	Area ebraeus	Area judaeus	#Feature	#Feature ebraeus	#Feature judaeus	Start	End	PTM	AScore	Found By
R.DEWGSGLDAMQVALALEK.S	Y	119.77	1931.9298	18	2.9	966.9709	2	59.33	2	F2:131653	20210611-09_2021021-OK160A_816_5.3.236.mgf		0	0	0	0	89	106			PEAKS DB
R.DDVALPGFAK.F	N	90.93	1031.5287	10	2.5	516.7708	2	38.01	2	F2:97999	20210611-09_2021021-OK160A_816_5.3.236.mgf	0	0	0	0	0	42	51			PEAKS DB
R.VGPGLGEYMFDK.E	Y	89.25	1311.6169	12	2.3	656.8145	2	42.16	2	F2:105049	20210611-09_2021021-OK160A_816_5.3.236.mgf		0	0	0	0	156	167			PEAKS DB
K.ELGDYITNLK.R	Y	84.29	1164.6027	10	2.4	583.3076	2	39.25	2	F2:100121	20210611-09_2021021-OK160A_816_5.3.236.mgf		0	0	0	0	145	154			PEAKS DB
R.VGPGLGEYMFDKETLQE	Y	80.78	1911.8923	17	3.6	956.9529	2	47.16	2	F2:113799	20210611-09_2021021-OK160A_816_5.3.236.mgf		0	0	0	0	156	172			PEAKS DB
R.QNYHAEC(+57.02)EAGINR.Q	Y	69.96	1560.6740	13	2.9	521.2313	3	16.74	2	F2:61108	20210611-09_2021021-OK160A_816_5.3.236.mgf		0	0	0	0	8	20	Carbamidomethylation	C7:Carbamidomethylation:1000.00	PEAKS DB
K.LMTFQNQR.G	Y	63.53	1036.5125	8	1.5	519.2621	2	24.34	2	F2:73402	20210611-09_2021021-OK160A_816_5.3.236.mgf		0	0	0	0	67	74			PEAKS DB
R.IVLQDIK.K	N	58.66	827.5116	7	-0.5	414.7611	2	28.93	2	F2:81450	20210611-09_2021021-OK160A_816_5.3.236.mgf		0	0	0	0	78	84			PEAKS DB

total 8 peptides

CebrOK014_E_137_Ferritin

back to list

| Protein Coverage | Supporting Peptides |

Protein Coverage:

1 MAVSQCRQNY HAECEAGINR QINMELHASY CYQSMAFYFD RDDVALPGFA KFFRKSSEEE REHAEKLMTF QNQRGGRIVL

81 QDIKKPDRDE WGSGLDAMQV ALALEKSVNQ SLLDLHEVAT NHNDAQMTDF LEGNYLQEQV RSIKELGDYI TNLKRVGPGL

161 GEYMFDKETL QE

C Carbamidomethylation (+57.02)

Supporting Peptides:

Peptide	Uniq	-10lgP	Mass	Length	ppm	m/z	z	RT	Fraction	Scan	Source File	Area ebraeus	Area judaeus	#Feature	#Feature ebraeus	#Feature judaeus	Start	End	PTM	AScore	Found By
R.DEWGSGLDAMQVALALEK.S	Y	119.77	1931.9298	18	2.9	966.9709	2	59.33	2	F2:131653	20210611-09_2021021-OK160A_816_5.3.236.mgf		0	0	0	0	89	106			PEAKS DB
R.DDVALPGFAK.F	N	90.93	1031.5287	10	2.5	516.7708	2	38.01	2	F2:97999	20210611-09_2021021-OK160A_816_5.3.236.mgf	0	0	0	0	0	42	51			PEAKS DB
R.VGPGLGEYMFDK.E	Y	89.25	1311.6169	12	2.3	656.8145	2	42.16	2	F2:105049	20210611-09_2021021-OK160A_816_5.3.236.mgf		0	0	0	0	156	167			PEAKS DB
K.ELGDYITNLK.R	Y	84.29	1164.6027	10	2.4	583.3076	2	39.25	2	F2:100121	20210611-09_2021021-OK160A_816_5.3.236.mgf		0	0	0	0	145	154			PEAKS DB
R.VGPGLGEYMFDKETLQE	Y	80.78	1911.8923	17	3.6	956.9529	2	47.16	2	F2:113799	20210611-09_2021021-OK160A_816_5.3.236.mgf		0	0	0	0	156	172			PEAKS DB
R.QNYHAEC(+57.02)EAGINR.Q	Y	69.96	1560.6740	13	2.9	521.2313	3	16.74	2	F2:61108	20210611-09_2021021-OK160A_816_5.3.236.mgf		0	0	0	0	8	20	Carbamidomethylation	C7:Carbamidomethylation:1000.00	PEAKS DB
K.LMTFQNQR.G	Y	63.53	1036.5125	8	1.5	519.2621	2	24.34	2	F2:73402	20210611-09_2021021-OK160A_816_5.3.236.mgf		0	0	0	0	67	74			PEAKS DB
R.IVLQDIK.K	N	58.66	827.5116	7	-0.5	414.7611	2	28.93	2	F2:81450	20210611-09_2021021-OK160A_816_5.3.236.mgf		0	0	0	0	78	84			PEAKS DB
**total 8 peptides**																					

SRX1323887_E_232_Ferritin

back to list

| Protein Coverage | Supporting Peptides |

Protein Coverage:

1 MAVSQCRQNY HAECEAGINR QINMELHASY CYQSMAFYFD RDDVALPGFA KFFRKSSEEE REHAEKLMTF QNQRGGRIVL

81 QDIKKPDRDE WGSGLDAMQV ALALEKSVNQ SLLDLHEVAT NHNDAQMTDF LEGNYLQEQV RSIKELGDYI TNLKRVGPGL

161 GEYMFDKETL QE

C Carbamidomethylation (+57.02)

Supporting Peptides:

Peptide	Uniq	-10lgP	Mass	Length	ppm	m/z	z	RT	Fraction	Scan	Source File	Area ebraeus	Area judaeus	#Feature	#Feature ebraeus	#Feature judaeus	Start	End	PTM	AScore	Found By
R.DEWGSGLDAMQVALALEK.S	Y	119.77	1931.9298	18	2.9	966.9709	2	59.33	2	F2:131653	20210611-09_2021021-OK160A_816_5.3.236.mgf		0	0	0	0	89	106			PEAKS DB
R.DDVALPGFAK.F	N	90.93	1031.5287	10	2.5	516.7708	2	38.01	2	F2:97999	20210611-09_2021021-OK160A_816_5.3.236.mgf	0	0	0	0	0	42	51			PEAKS DB
R.VGPGLGEYMFDK.E	Y	89.25	1311.6169	12	2.3	656.8145	2	42.16	2	F2:105049	20210611-09_2021021-OK160A_816_5.3.236.mgf		0	0	0	0	156	167			PEAKS DB
K.ELGDYITNLK.R	Y	84.29	1164.6027	10	2.4	583.3076	2	39.25	2	F2:100121	20210611-09_2021021-OK160A_816_5.3.236.mgf		0	0	0	0	145	154			PEAKS DB
R.VGPGLGEYMFDKETLQE	Y	80.78	1911.8923	17	3.6	956.9529	2	47.16	2	F2:113799	20210611-09_2021021-OK160A_816_5.3.236.mgf		0	0	0	0	156	172			PEAKS DB
R.QNYHAEC(+57.02)EAGINR.Q	Y	69.96	1560.6740	13	2.9	521.2313	3	16.74	2	F2:61108	20210611-09_2021021-OK160A_816_5.3.236.mgf		0	0	0	0	8	20	Carbamidomethylation	C7:Carbamidomethylation:1000.00	PEAKS DB
K.LMTFQNQR.G	Y	63.53	1036.5125	8	1.5	519.2621	2	24.34	2	F2:73402	20210611-09_2021021-OK160A_816_5.3.236.mgf		0	0	0	0	67	74			PEAKS DB
R.IVLQDIK.K	N	58.66	827.5116	7	-0.5	414.7611	2	28.93	2	F2:81450	20210611-09_2021021-OK160A_816_5.3.236.mgf		0	0	0	0	78	84			PEAKS DB
**total 8 peptides**																					

OK214_J_314_Ferritin

back to list

| Protein Coverage | Supporting Peptides |

Protein Coverage:

1 MAVSQCRQNY HAECEAGINR QINMELHASY CYQSMAFYFD RDDVALPGFA KFFRKSSEEE REHAEKLMTF QNQRGGRIVL

81 QDIKKPDRDE WGSGLDAMQV ALALEKSVNQ SLLDLHEVAT NHNDAQMTDF LEGNYLQEQV RSIKELGDYI TNLKRVGPGL

161 GEYMFDKETL QE

C Carbamidomethylation (+57.02)

Supporting Peptides:

Peptide	Uniq	-10lgP	Mass	Length	ppm	m/z	z	RT	Fraction	Scan	Source File	Area ebraeus	Area judaeus	#Feature	#Feature ebraeus	#Feature judaeus	Start	End	PTM	AScore	Found By
R.DEWGSGLDAMQVALALEK.S	Y	119.77	1931.9298	18	2.9	966.9709	2	59.33	2	F2:131653	20210611-09_2021021-OK160A_816_5.3.236.mgf		0	0	0	0	89	106			PEAKS DB
R.DDVALPGFAK.F	N	90.93	1031.5287	10	2.5	516.7708	2	38.01	2	F2:97999	20210611-09_2021021-OK160A_816_5.3.236.mgf	0	0	0	0	0	42	51			PEAKS DB
R.VGPGLGEYMFDK.E	Y	89.25	1311.6169	12	2.3	656.8145	2	42.16	2	F2:105049	20210611-09_2021021-OK160A_816_5.3.236.mgf		0	0	0	0	156	167			PEAKS DB
K.ELGDYITNLK.R	Y	84.29	1164.6027	10	2.4	583.3076	2	39.25	2	F2:100121	20210611-09_2021021-OK160A_816_5.3.236.mgf		0	0	0	0	145	154			PEAKS DB
R.VGPGLGEYMFDKETLQE	Y	80.78	1911.8923	17	3.6	956.9529	2	47.16	2	F2:113799	20210611-09_2021021-OK160A_816_5.3.236.mgf		0	0	0	0	156	172			PEAKS DB
R.QNYHAEC(+57.02)EAGINR.Q	Y	69.96	1560.6740	13	2.9	521.2313	3	16.74	2	F2:61108	20210611-09_2021021-OK160A_816_5.3.236.mgf		0	0	0	0	8	20	Carbamidomethylation	C7:Carbamidomethylation:1000.00	PEAKS DB
K.LMTFQNQR.G	Y	63.53	1036.5125	8	1.5	519.2621	2	24.34	2	F2:73402	20210611-09_2021021-OK160A_816_5.3.236.mgf		0	0	0	0	67	74			PEAKS DB
R.IVLQDIK.K	N	58.66	827.5116	7	-0.5	414.7611	2	28.93	2	F2:81450	20210611-09_2021021-OK160A_816_5.3.236.mgf		0	0	0	0	78	84			PEAKS DB
**total 8 peptides**																					

CebrOK035_J_350_Ferritin

back to list

| Protein Coverage | Supporting Peptides |

Protein Coverage:

1 MAVSQCR**QNY HAECEAGINR** QINMELHASY CYQSMAFYFD R**DDVALPGFA K**FFRKSSEEE REHAEK**LMTF QNQR**GGR**IVL**

81 **QDIK**KPDR**DE WGSGLDAMQV ALALEK**SVNQ SLLDLHEVAT NHNDAQMTDF LEGNYLQEQV RSIK**ELGDYI TNLK**R**VGPGL**

161 **GEYMFDKETL QE**

C Carbamidomethylation (+57.02)

Supporting Peptides:

Peptide	Uniq	-10lgP	Mass	Length	ppm	m/z	z	RT	Fraction	Scan	Source File	Area ebraeus	Area judaeus	#Feature	#Feature ebraeus	#Feature judaeus	Start	End	PTM	AScore	Found By
R.DEWGSGLDAMQVALALEK.S	Y	119.77	1931.9298	18	2.9	966.9709	2	59.33	2	F2:131653	20210611-09_2021021-OK160A_816_5.3.236.mgf		0	0	0	0	89	106			PEAKS DB
R.DDVALPGFAK.F	N	90.93	1031.5287	10	2.5	516.7708	2	38.01	2	F2:97999	20210611-09_2021021-OK160A_816_5.3.236.mgf	0	0	0	0	0	42	51			PEAKS DB
R.VGPGLGEYMFDK.E	Y	89.25	1311.6169	12	2.3	656.8145	2	42.16	2	F2:105049	20210611-09_2021021-OK160A_816_5.3.236.mgf		0	0	0	0	156	167			PEAKS DB
K.ELGDYITNLK.R	Y	84.29	1164.6027	10	2.4	583.3076	2	39.25	2	F2:100121	20210611-09_2021021-OK160A_816_5.3.236.mgf		0	0	0	0	145	154			PEAKS DB
R.VGPGLGEYMFDKETLQE	Y	80.78	1911.8923	17	3.6	956.9529	2	47.16	2	F2:113799	20210611-09_2021021-OK160A_816_5.3.236.mgf		0	0	0	0	156	172			PEAKS DB
R.QNYHAEC(+57.02)EAGINR.Q	Y	69.96	1560.6740	13	2.9	521.2313	3	16.74	2	F2:61108	20210611-09_2021021-OK160A_816_5.3.236.mgf		0	0	0	0	8	20	Carbamidomethylation	C7:Carbamidomethylation:1000.00	PEAKS DB
K.LMTFQNQR.G	Y	63.53	1036.5125	8	1.5	519.2621	2	24.34	2	F2:73402	20210611-09_2021021-OK160A_816_5.3.236.mgf		0	0	0	0	67	74			PEAKS DB
R.IVLQDIK.K	N	58.66	827.5116	7	-0.5	414.7611	2	28.93	2	F2:81450	20210611-09_2021021-OK160A_816_5.3.236.mgf		0	0	0	0	78	84			PEAKS DB
**total 8 peptides**																					

CebrOK033_J_243_Ferritin

back to list

| Protein Coverage | Supporting Peptides |

Protein Coverage:

1 MAVSQCR**QNY HAECEAGINR** QINMELHASY CYQSMAFYFD R**DDVALPGFA K**FFRKSSEEE REHAEK**LMTF QNQR**GGR**IVL**

81 **QDIK**KPDR**DE WGSGLDAMQV ALALEK**SVNQ SLLDLHEVAT NHNDAQMTDF LEGNYLQEQV RSIK**ELGDYI TNLK**R**VGPGL**

161 **GEYMFDKETL QE**

C Carbamidomethylation (+57.02)

Supporting Peptides:

Peptide	Uniq	-10lgP	Mass	Length	ppm	m/z	z	RT	Fraction	Scan	Source File	Area ebraeus	Area judaeus	#Feature	#Feature ebraeus	#Feature judaeus	Start	End	PTM	AScore	Found By
R.DEWGSGLDAMQVALALEK.S	Y	119.77	1931.9298	18	2.9	966.9709	2	59.33	2	F2:131653	20210611-09_2021021-OK160A_816_5.3.236.mgf		0	0	0	0	89	106			PEAKS DB
R.DDVALPGFAK.F	N	90.93	1031.5287	10	2.5	516.7708	2	38.01	2	F2:97999	20210611-09_2021021-OK160A_816_5.3.236.mgf	0	0	0	0	0	42	51			PEAKS DB
R.VGPGLGEYMFDK.E	Y	89.25	1311.6169	12	2.3	656.8145	2	42.16	2	F2:105049	20210611-09_2021021-OK160A_816_5.3.236.mgf		0	0	0	0	156	167			PEAKS DB
K.ELGDYITNLK.R	Y	84.29	1164.6027	10	2.4	583.3076	2	39.25	2	F2:100121	20210611-09_2021021-OK160A_816_5.3.236.mgf		0	0	0	0	145	154			PEAKS DB
R.VGPGLGEYMFDKETLQE	Y	80.78	1911.8923	17	3.6	956.9529	2	47.16	2	F2:113799	20210611-09_2021021-OK160A_816_5.3.236.mgf		0	0	0	0	156	172			PEAKS DB
R.QNYHAEC(+57.02)EAGINR.Q	Y	69.96	1560.6740	13	2.9	521.2313	3	16.74	2	F2:61108	20210611-09_2021021-OK160A_816_5.3.236.mgf		0	0	0	0	8	20	Carbamidomethylation	C7:Carbamidomethylation:1000.00	PEAKS DB
K.LMTFQNQR.G	Y	63.53	1036.5125	8	1.5	519.2621	2	24.34	2	F2:73402	20210611-09_2021021-OK160A_816_5.3.236.mgf		0	0	0	0	67	74			PEAKS DB
R.IVLQDIK.K	N	58.66	827.5116	7	-0.5	414.7611	2	28.93	2	F2:81450	20210611-09_2021021-OK160A_816_5.3.236.mgf		0	0	0	0	78	84			PEAKS DB
**total 8 peptides**																					

OK212_J_295_Ferritin

back to list

| Protein Coverage | Supporting Peptides |

Protein Coverage:

1 MAVSQCR**QNY HAECEAGINR** QINMEFHASY CYQSMAFYFD R**DDVALPGFA K**FFRKSSEEE REHAEK**LMTF QNQR**GGR**IVL**

81 **QDIK**KPDR**DE WGSGLDAMQV ALALEK**SVNQ SLLDLHEVAT NHNDAQMTDF LEGNYLQEQV RSIK**ELGDYI TNLK**R**VGPGL**

161 **GEYMFDKETL QE**

C Carbamidomethylation (+57.02)

Supporting Peptides:

Peptide	Uniq	-10lgP	Mass	Length	ppm	m/z	z	RT	Fraction	Scan	Source File	Area ebraeus	Area judaeus	#Feature	#Feature ebraeus	#Feature judaeus	Start	End	PTM	AScore	Found By
R.DEWGSGLDAMQVALALEK.S	Y	119.77	1931.9298	18	2.9	966.9709	2	59.33	2	F2:131653	20210611-09_2021021-OK160A_816_5.3.236.mgf		0	0	0	0	89	106			PEAKS DB
R.DDVALPGFAK.F	N	90.93	1031.5287	10	2.5	516.7708	2	38.01	2	F2:97999	20210611-09_2021021-OK160A_816_5.3.236.mgf	0	0	0	0	0	42	51			PEAKS DB
R.VGPGLGEYMFDK.E	Y	89.25	1311.6169	12	2.3	656.8145	2	42.16	2	F2:105049	20210611-09_2021021-OK160A_816_5.3.236.mgf		0	0	0	0	156	167			PEAKS DB
K.ELGDYITNLK.R	Y	84.29	1164.6027	10	2.4	583.3076	2	39.25	2	F2:100121	20210611-09_2021021-OK160A_816_5.3.236.mgf		0	0	0	0	145	154			PEAKS DB
R.VGPGLGEYMFDKETLQE	Y	80.78	1911.8923	17	3.6	956.9529	2	47.16	2	F2:113799	20210611-09_2021021-OK160A_816_5.3.236.mgf		0	0	0	0	156	172			PEAKS DB
R.QNYHAEC(+57.02)EAGINR.Q	Y	69.96	1560.6740	13	2.9	521.2313	3	16.74	2	F2:61108	20210611-09_2021021-OK160A_816_5.3.236.mgf		0	0	0	0	8	20	Carbamidomethylation	C7:Carbamidomethylation:1000.00	PEAKS DB
K.LMTFQNQR.G	Y	63.53	1036.5125	8	1.5	519.2621	2	24.34	2	F2:73402	20210611-09_2021021-OK160A_816_5.3.236.mgf		0	0	0	0	67	74			PEAKS DB
R.IVLQDIK.K	N	58.66	827.5116	7	-0.5	414.7611	2	28.93	2	F2:81450	20210611-09_2021021-OK160A_816_5.3.236.mgf		0	0	0	0	78	84			PEAKS DB
**total 8 peptides**																					

V_1278_120_Ferritin

back to list

| Protein Coverage | Supporting Peptides |

Protein Coverage:

14
C

1 MAVSQCRQNY HAECEAGINR QINMELHASY CYQSMAFFFD RDDVALPGFA KFFRKSSEEE REHAEKLMTF QNQRGGRIVL   C Carbamidomethylation (+57.02)

81 QDIKKPDRDE WGSGLDAMQV ALALEKSVNQ SLLDLHEVAT NHNDAQMTDF LEGNYLQEQV RSIKELGDYI TNLKRVGPGL

161 GEYMFDKETL QE

Supporting Peptides:

Peptide	Uniq	-10lgP	Mass	Length	ppm	m/z	z	RT	Fraction	Scan	Source File	Area ebraeus	Area judaeus	#Feature	#Feature ebraeus	#Feature judaeus	Start	End	PTM	AScore	Found By
R.DEWGSGLDAMQVALALEK.S	Y	119.77	1931.9298	18	2.9	966.9709	2	59.33	2	F2:131653	20210611-09_2021021-OK160A_816_5.3.236.mgf		0	0	0	0	89	106			PEAKS DB
R.DDVALPGFAK.F	N	90.93	1031.5287	10	2.5	516.7708	2	38.01	2	F2:97999	20210611-09_2021021-OK160A_816_5.3.236.mgf	0	0	0	0	0	42	51			PEAKS DB
R.VGPGLGEYMFDK.E	Y	89.25	1311.6169	12	2.3	656.8145	2	42.16	2	F2:105049	20210611-09_2021021-OK160A_816_5.3.236.mgf		0	0	0	0	156	167			PEAKS DB
K.ELGDYITNLK.R	Y	84.29	1164.6027	10	2.4	583.3076	2	39.25	2	F2:100121	20210611-09_2021021-OK160A_816_5.3.236.mgf		0	0	0	0	145	154			PEAKS DB
R.VGPGLGEYMFDKETLQE	Y	80.78	1911.8923	17	3.6	956.9529	2	47.16	2	F2:113799	20210611-09_2021021-OK160A_816_5.3.236.mgf		0	0	0	0	156	172			PEAKS DB
R.QNYHAEC(+57.02)EAGINR.Q	Y	69.96	1560.6740	13	2.9	521.2313	3	16.74	2	F2:61108	20210611-09_2021021-OK160A_816_5.3.236.mgf		0	0	0	0	8	20	Carbamidomethylation	C7:Carbamidomethylation:1000.00	PEAKS DB
K.LMTFQNQR.G	Y	63.53	1036.5125	8	1.5	519.2621	2	24.34	2	F2:73402	20210611-09_2021021-OK160A_816_5.3.236.mgf		0	0	0	0	67	74			PEAKS DB
R.IVLQDIK.K	N	58.66	827.5116	7	-0.5	414.7611	2	28.93	2	F2:81450	20210611-09_2021021-OK160A_816_5.3.236.mgf		0	0	0	0	78	84			PEAKS DB
total 8 peptides																					

V_1302_266_Ferritin

back to list

| Protein Coverage | Supporting Peptides |

Protein Coverage:

14
C

1 MAVSQCRQNY HAECEAGINR QINMELHASY CYQSMAFFFD RDDVALPGFA KFFRKSSEEE REHAEKLMTF QNQRGGRIVL   C Carbamidomethylation (+57.02)

81 QDIKKPDRDE WGSGLDAMQV ALALEKSVNQ SLLDLHEVAT NHNDAQMTDF LEGNYLQEQV RSIKELGDYI TNLKRVGPGL

161 GEYMFDKETL QE

Supporting Peptides:

Peptide	Uniq	-10lgP	Mass	Length	ppm	m/z	z	RT	Fraction	Scan	Source File	Area ebraeus	Area judaeus	#Feature	#Feature ebraeus	#Feature judaeus	Start	End	PTM	AScore	Found By
R.DEWGSGLDAMQVALALEK.S	Y	119.77	1931.9298	18	2.9	966.9709	2	59.33	2	F2:131653	20210611-09_2021021-OK160A_816_5.3.236.mgf		0	0	0	0	89	106			PEAKS DB
R.DDVALPGFAK.F	N	90.93	1031.5287	10	2.5	516.7708	2	38.01	2	F2:97999	20210611-09_2021021-OK160A_816_5.3.236.mgf	0	0	0	0	0	42	51			PEAKS DB
R.VGPGLGEYMFDK.E	Y	89.25	1311.6169	12	2.3	656.8145	2	42.16	2	F2:105049	20210611-09_2021021-OK160A_816_5.3.236.mgf		0	0	0	0	156	167			PEAKS DB
K.ELGDYITNLK.R	Y	84.29	1164.6027	10	2.4	583.3076	2	39.25	2	F2:100121	20210611-09_2021021-OK160A_816_5.3.236.mgf		0	0	0	0	145	154			PEAKS DB
R.VGPGLGEYMFDKETLQE	Y	80.78	1911.8923	17	3.6	956.9529	2	47.16	2	F2:113799	20210611-09_2021021-OK160A_816_5.3.236.mgf		0	0	0	0	156	172			PEAKS DB
R.QNYHAEC(+57.02)EAGINR.Q	Y	69.96	1560.6740	13	2.9	521.2313	3	16.74	2	F2:61108	20210611-09_2021021-OK160A_816_5.3.236.mgf		0	0	0	0	8	20	Carbamidomethylation	C7:Carbamidomethylation:1000.00	PEAKS DB
K.LMTFQNQR.G	Y	63.53	1036.5125	8	1.5	519.2621	2	24.34	2	F2:73402	20210611-09_2021021-OK160A_816_5.3.236.mgf		0	0	0	0	67	74			PEAKS DB
R.IVLQDIK.K	N	58.66	827.5116	7	-0.5	414.7611	2	28.93	2	F2:81450	20210611-09_2021021-OK160A_816_5.3.236.mgf		0	0	0	0	78	84			PEAKS DB
total 8 peptides																					

V_1258_223_Ferritin

back to list

| Protein Coverage | Supporting Peptides |

Protein Coverage:

14
C

1 MAVSQCRQNY HAECEAGINR QINMELHASY CYQSMAFFFD RDDVALPGFA KFFRKSSEEE REHAEKLMTF QNQRGGRIVL   C Carbamidomethylation (+57.02)

81 QDIKKPDRDE WGSGLDAMQV ALALEKSVNQ SLLDLHEVAT NHNDAQMTDF LEGNYLQEQV RSIKELGDYI TNLKRVGPGL

161 GEYMFDKETL QE

Supporting Peptides:

Peptide	Uniq	-10lgP	Mass	Length	ppm	m/z	z	RT	Fraction	Scan	Source File	Area ebraeus	Area judaeus	#Feature	#Feature ebraeus	#Feature judaeus	Start	End	PTM	AScore	Found By
R.DEWGSGLDAMQVALALEK.S	Y	119.77	1931.9298	18	2.9	966.9709	2	59.33	2	F2:131653	20210611-09_2021021-OK160A_816_5.3.236.mgf		0	0	0	0	89	106			PEAKS DB
R.DDVALPGFAK.F	N	90.93	1031.5287	10	2.5	516.7708	2	38.01	2	F2:97999	20210611-09_2021021-OK160A_816_5.3.236.mgf	0	0	0	0	0	42	51			PEAKS DB
R.VGPGLGEYMFDK.E	Y	89.25	1311.6169	12	2.3	656.8145	2	42.16	2	F2:105049	20210611-09_2021021-OK160A_816_5.3.236.mgf		0	0	0	0	156	167			PEAKS DB
K.ELGDYITNLK.R	Y	84.29	1164.6027	10	2.4	583.3076	2	39.25	2	F2:100121	20210611-09_2021021-OK160A_816_5.3.236.mgf		0	0	0	0	145	154			PEAKS DB
R.VGPGLGEYMFDKETLQE	Y	80.78	1911.8923	17	3.6	956.9529	2	47.16	2	F2:113799	20210611-09_2021021-OK160A_816_5.3.236.mgf		0	0	0	0	156	172			PEAKS DB
R.QNYHAEC(+57.02)EAGINR.Q	Y	69.96	1560.6740	13	2.9	521.2313	3	16.74	2	F2:61108	20210611-09_2021021-OK160A_816_5.3.236.mgf		0	0	0	0	8	20	Carbamidomethylation	C7:Carbamidomethylation:1000.00	PEAKS DB
K.LMTFQNQR.G	Y	63.53	1036.5125	8	1.5	519.2621	2	24.34	2	F2:73402	20210611-09_2021021-OK160A_816_5.3.236.mgf		0	0	0	0	67	74			PEAKS DB
R.IVLQDIK.K	N	58.66	827.5116	7	-0.5	414.7611	2	28.93	2	F2:81450	20210611-09_2021021-OK160A_816_5.3.236.mgf		0	0	0	0	78	84			PEAKS DB
total 8 peptides																					

V_CG13_283_Ferritin

back to list

| Protein Coverage | Supporting Peptides |

Protein Coverage:

1 MAVSQCRQNY HAECEAGINR QINMELHASY CYQSMAFFFD RDDVALPGFA KFFRKSSEEE REHAEKLMTF QNQRGGRIVL

81 QDIKKPDRDE WGSGLDAMQV ALALEKSVNQ SLLDLHEVAT NHNDAQMTDF LEGNYLQEQV RSIKELGDYI TNLKRVGPGL

161 GEYMFDKETL QE

C Carbamidomethylation (+57.02)

Supporting Peptides:

Peptide	Uniq	-10lgP	Mass	Length	ppm	m/z	z	RT	Fraction	Scan	Source File	Area ebraeus	Area judaeus	#Feature	#Feature ebraeus	#Feature judaeus	Start	End	PTM	AScore	Found By
R.DEWGSGLDAMQVALALEK.S	Y	119.77	1931.9298	18	2.9	966.9709	2	59.33	2	F2:131653	20210611-09_2021021-OK160A_816_5.3.236.mgf		0	0	0	0	89	106			PEAKS DB
R.DDVALPGFAK.F	N	90.93	1031.5287	10	2.5	516.7708	2	38.01	2	F2:97999	20210611-09_2021021-OK160A_816_5.3.236.mgf	0	0	0	0	0	42	51			PEAKS DB
R.VGPGLGEYMFDK.E	Y	89.25	1311.6169	12	2.3	656.8145	2	42.16	2	F2:105049	20210611-09_2021021-OK160A_816_5.3.236.mgf		0	0	0	0	156	167			PEAKS DB
K.ELGDYITNLK.R	Y	84.29	1164.6027	10	2.4	583.3076	2	39.25	2	F2:100121	20210611-09_2021021-OK160A_816_5.3.236.mgf		0	0	0	0	145	154			PEAKS DB
R.VGPGLGEYMFDKETLQE	Y	80.78	1911.8923	17	3.6	956.9529	2	47.16	2	F2:113799	20210611-09_2021021-OK160A_816_5.3.236.mgf		0	0	0	0	156	172			PEAKS DB
R.QNYHAEC(+57.02)EAGINR.Q	Y	69.96	1560.6740	13	2.9	521.2313	3	16.74	2	F2:61108	20210611-09_2021021-OK160A_816_5.3.236.mgf		0	0	0	0	8	20	Carbamidomethylation	C7:Carbamidomethylation:1000.00	PEAKS DB
K.LMTFQNQR.G	Y	63.53	1036.5125	8	1.5	519.2621	2	24.34	2	F2:73402	20210611-09_2021021-OK160A_816_5.3.236.mgf		0	0	0	0	67	74			PEAKS DB
R.IVLQDIK.K	N	58.66	827.5116	7	-0.5	414.7611	2	28.93	2	F2:81450	20210611-09_2021021-OK160A_816_5.3.236.mgf		0	0	0	0	78	84			PEAKS DB
**total 8 peptides**																					

OK212_J_014_B2

back to list

| Protein Coverage | Supporting Peptides |

Protein Coverage:

1 YPQRRDGAPA NAAPNLQPFN GEMAMPNMQP MHALPMSMSG MSSPQLMPLN PNLGMGFKRE NLEKRRQHSQ FSDDNKSPFD

81 SEDGLGNFMN FMKENGNNLP FANMDSAATD LGHFEPSAEN EDGKFRFFDK EQ

a Amidation (-0.98)
P Pyro-glu from Q (-17.03)

Supporting Peptides:

Peptide	Uniq	-10lgP	Mass	Length	ppm	m/z	z	RT	Fraction	Scan	Source File	Area ebraeus	Area judaeus	#Feature	#Feature ebraeus	#Feature judaeus	Start	End	PTM	AScore
K.ENGNNLPFANMDSAATDLGHFEPSAENEDGK.F	Y	125.47	3290.4058	31	10.2	1097.8158	3	50.09	2	F2:119029	20210611-09_2021021-OK160A_816_5.3.236.mgf		0	0	0	0	94	124		
R.Q(-17.03)HSQFSDDNK.S	N	112.62	1187.4843	10	2.1	594.7482	2	13.73	2	F2:57082	20210611-09_2021021-OK160A_816_5.3.236.mgf	0	0	0	0	0	67	76	Pyro-glu from Q	Q1:Pyro-glu from Q:1000.00
K.ENGNNLPFANM(+15.99)DSAATDLGHFEPSAENEDGK.F	Y	108.80	3306.4006	31	11.4	1103.1488	3	43.68	2	F2:107793	20210611-09_2021021-OK160A_816_5.3.236.mgf		0	0	0	0	94	124		M11:Oxidation (M):1000.00
K.SPFDSEDGLGNFM(+15.99)NFM(+15.99)K.E	N	104.89	1966.8076	17	2.8	984.4097	2	45.96	2	F2:112336	20210611-09_2021021-OK160A_816_5.3.236.mgf	0	0	0	0	0	77	93		M13:Oxidation (M):1000.00; M16:Oxidation (M):1000.00
R.RQHSQFSDDNK.S	N	100.18	1360.6119	11	0.4	454.5429	3	6.44	2	F2:52336	20210611-09_2021021-OK160A_816_5.3.236.mgf		0	0	0	0	66	76		
R.Q(-17.03)HSQFSDDNK(-.98).S	N	90.51	1186.5002	10	2.1	594.2562	2	12.84	2	F2:55863	20210611-09_2021021-OK160A_816_5.3.236.mgf		0	0	0	0	67	76	Amidation	Q1:Pyro-glu from Q:1000.00; K10:Amidation:1000.00
R.QHSQFSDDNK.S	N	87.11	1204.5109	10	-2.8	603.2585	2	8.72	2	F2:52560	20210611-09_2021021-OK160A_816_5.3.236.mgf		0	0	0	0	67	76		
K.SPFDSEDGLGNFMNFMK.E	N	83.66	1934.8179	17	0.7	968.4128	2	59.14	2	F2:131486	20210611-09_2021021-OK160A_816_5.3.236.mgf		0	0	0	0	77	93		
K.E(-18.01)NGNNLPFANMDSAATDLGHFEPSAENEDGK.F	Y	83.49	3272.3953	31	0.6	1091.8018	3	55.58	2	F2:127016	20210611-09_2021021-OK160A_816_5.3.236.mgf		0	0	0	0	94	124		E1:Pyro-glu from E:1000.00
K.SPFDSEDGLGNFM(+15.99)NFMK.E	N	76.38	1950.8127	17	2.4	976.4119	2	53.56	2	F2:124496	20210611-09_2021021-OK160A_816_5.3.236.mgf		0	0	0	0	77	93		M13:Oxidation (M):0.00
K.RRQHSQFSDDNK.S	N	72.54	1516.7130	12	10.8	506.5816	3	19.31	2	F2:64986	20210611-09_2021021-OK160A_816_5.3.236.mgf		0	0	0	0	65	76		
**total 11 peptides**																				

OK214_J_010_B2

back to list

| Protein Coverage | Supporting Peptides |

Protein Coverage:

1 YPQRRDGAPA NAAPNLQPFN GEMAMPNMQP MHALPMSMSG MSSPQLMPLN PNLGMGFKRE NLEKRRQHSQ FSDDNKSPFD

81 SEDGLGNFMN FMKENGNNLP FANMDSAATD LGHFEPSAEN EDGKFRFFDK EQ

a Amidation (-0.98)
P Pyro-glu from Q (-17.03)

Supporting Peptides:

Peptide	Uniq	-10lgP	Mass	Length	ppm	m/z	z	RT	Fraction	Scan	Source File	Area ebraeus	Area judaeus	#Feature	#Feature ebraeus	#Feature judaeus	Start	End	PTM	AScore
K.ENGNNLPFANMDSAATDLGHFEPSAENEDGK.F	Y	125.47	3290.4058	31	10.2	1097.8158	3	50.09	2	F2:119029	20210611-09_2021021-OK160A_816_5.3.236.mgf		0	0	0	0	94	124		
R.Q(-17.03)HSQFSDDNK.S	N	112.62	1187.4843	10	2.1	594.7482	2	13.73	2	F2:57082	20210611-09_2021021-OK160A_816_5.3.236.mgf	0	0	0	0	0	67	76	Pyro-glu from Q	Q1:Pyro-glu from Q:1000.00
K.ENGNNLPFANM(+15.99)DSAATDLGHFEPSAENEDGK.F	Y	108.80	3306.4006	31	11.4	1103.1488	3	43.68	2	F2:107793	20210611-09_2021021-OK160A_816_5.3.236.mgf		0	0	0	0	94	124		M11:Oxidation (M):1000.00
K.SPFDSEDGLGNFM(+15.99)NFM(+15.99)K.E	N	104.89	1966.8076	17	2.8	984.4097	2	45.96	2	F2:112336	20210611-09_2021021-OK160A_816_5.3.236.mgf	0	0	0	0	0	77	93		M13:Oxidation (M):1000.00; M16:Oxidation (M):1000.00
**total 11 peptides**																				

Peptide	Uniq	-10lgP	Mass	Length	ppm	m/z	z	RT	Fraction	Scan	Source File	Area ebraeus	Area judaeus	#Feature	#Feature ebraeus	#Feature judaeus	Start	End	PTM	AScore
R.RQHSQFSDDNK.S	N	100.18	1360.6119	11	0.4	454.5429	3	6.44	2	F2:52336	20210611-09_2021021-OK160A_816_5.3.236.mgf		0	0	0	0	66	76		
R.Q(-17.03)HSQFSDDNK(-.98).S	N	90.51	1186.5002	10	2.1	594.2562	2	12.84	2	F2:55863	20210611-09_2021021-OK160A_816_5.3.236.mgf		0	0	0	0	67	76	Amidation	Q1:Pyro-glu from Q:1000.00; K10:Amidation:1000.00
R.QHSQFSDDNK.S	N	87.11	1204.5109	10	-2.8	603.2585	2	8.72	2	F2:52560	20210611-09_2021021-OK160A_816_5.3.236.mgf		0	0	0	0	67	76		
K.SPFDSEDGLGNFMNFMK.E	N	83.66	1934.8179	17	0.7	968.4128	2	59.14	2	F2:131486	20210611-09_2021021-OK160A_816_5.3.236.mgf		0	0	0	0	77	93		
K.E(-18.01)NGNNLPFANMDSAATDLGHFEPSAENEDGK.F	Y	83.49	3272.3953	31	0.6	1091.8018	3	55.58	2	F2:127016	20210611-09_2021021-OK160A_816_5.3.236.mgf		0	0	0	0	94	124		E1:Pyro-glu from E:1000.00
K.SPFDSEDGLGNFM(+15.99)NFMK.E	N	76.38	1950.8127	17	2.4	976.4119	2	53.56	2	F2:124496	20210611-09_2021021-OK160A_816_5.3.236.mgf		0	0	0	0	77	93		M13:Oxidation (M):0.00
K.RRQHSQFSDDNK.S	N	72.54	1516.7130	12	10.8	506.5816	3	19.31	2	F2:64986	20210611-09_2021021-OK160A_816_5.3.236.mgf		0	0	0	0	65	76		

total 11 peptides

OK005_J_013_B2

back to list

| Protein Coverage | Supporting Peptides |

Protein Coverage:

1 YPQRRDGAPA NAAPNLQPFN GEMAMPNMQP MHALPMSMSG MSSPQLMPLN PNLGMGFKRE NLEKRRQHSQ FSDDNKSPFD

81 SEDGLGNFMN FMKENGNNLP FANMDSAATD LGHFEPSAEN EDGKFRFFDK EQ

a Amidation (-0.98)
P Pyro-glu from Q (-17.03)

Supporting Peptides:

Peptide	Uniq	-10lgP	Mass	Length	ppm	m/z	z	RT	Fraction	Scan	Source File	Area ebraeus	Area judaeus	#Feature	#Feature ebraeus	#Feature judaeus	Start	End	PTM	AScore
K.ENGNNLPFANMDSAATDLGHFEPSAENEDGK.F	Y	125.47	3290.4058	31	10.2	1097.8158	3	50.09	2	F2:119029	20210611-09_2021021-OK160A_816_5.3.236.mgf		0	0	0	0	94	124		
R.Q(-17.03)HSQFSDDNK.S	N	112.62	1187.4843	10	2.1	594.7482	2	13.73	2	F2:57082	20210611-09_2021021-OK160A_816_5.3.236.mgf	0	0	0	0	0	67	76	Pyro-glu from Q	Q1:Pyro-glu from Q:1000.00
K.ENGNNLPFANM(+15.99)DSAATDLGHFEPSAENEDGK.F	Y	108.80	3306.4006	31	11.4	1103.1488	3	43.68	2	F2:107793	20210611-09_2021021-OK160A_816_5.3.236.mgf		0	0	0	0	94	124		M11:Oxidation (M):1000.00
K.SPFDSEDGLGNFM(+15.99)NFM(+15.99)K.E	N	104.89	1966.8076	17	2.8	984.4097	2	45.96	2	F2:112336	20210611-09_2021021-OK160A_816_5.3.236.mgf	0	0	0	0	0	77	93		M13:Oxidation (M):1000.00; M16:Oxidation (M):1000.00
R.RQHSQFSDDNK.S	N	100.18	1360.6119	11	0.4	454.5429	3	6.44	2	F2:52336	20210611-09_2021021-OK160A_816_5.3.236.mgf		0	0	0	0	66	76		
R.Q(-17.03)HSQFSDDNK(-.98).S	N	90.51	1186.5002	10	2.1	594.2562	2	12.84	2	F2:55863	20210611-09_2021021-OK160A_816_5.3.236.mgf		0	0	0	0	67	76	Amidation	Q1:Pyro-glu from Q:1000.00; K10:Amidation:1000.00
R.QHSQFSDDNK.S	N	87.11	1204.5109	10	-2.8	603.2585	2	8.72	2	F2:52560	20210611-09_2021021-OK160A_816_5.3.236.mgf		0	0	0	0	67	76		
K.SPFDSEDGLGNFMNFMK.E	N	83.66	1934.8179	17	0.7	968.4128	2	59.14	2	F2:131486	20210611-09_2021021-OK160A_816_5.3.236.mgf		0	0	0	0	77	93		
K.E(-18.01)NGNNLPFANMDSAATDLGHFEPSAENEDGK.F	Y	83.49	3272.3953	31	0.6	1091.8018	3	55.58	2	F2:127016	20210611-09_2021021-OK160A_816_5.3.236.mgf		0	0	0	0	94	124		E1:Pyro-glu from E:1000.00
K.SPFDSEDGLGNFM(+15.99)NFMK.E	N	76.38	1950.8127	17	2.4	976.4119	2	53.56	2	F2:124496	20210611-09_2021021-OK160A_816_5.3.236.mgf		0	0	0	0	77	93		M13:Oxidation (M):0.00
K.RRQHSQFSDDNK.S	N	72.54	1516.7130	12	10.8	506.5816	3	19.31	2	F2:64986	20210611-09_2021021-OK160A_816_5.3.236.mgf		0	0	0	0	65	76		

total 11 peptides

CebrOK035_J_018_B2

back to list

| Protein Coverage | Supporting Peptides |

Protein Coverage:

1 YPQRRDGAPA NAAPNLQPFN GEMAMPNMQP MHALPMSMSG MSSPQLMPLN PNLGMGFKRE NLEKRRQHSQ FSDDNKSPFD

81 SEDGLGNFMN FMKENGNNLP FANMDSAATD LGHFEPSAEN EDGKFRFFDK EQ

a Amidation (-0.98)
P Pyro-glu from Q (-17.03)

Supporting Peptides:

Peptide	Uniq	-10lgP	Mass	Length	ppm	m/z	z	RT	Fraction	Scan	Source File	Area ebraeus	Area judaeus	#Feature	#Feature ebraeus	#Feature judaeus	Start	End	PTM	AScore
K.ENGNNLPFANMDSAATDLGHFEPSAENEDGK.F	Y	125.47	3290.4058	31	10.2	1097.8158	3	50.09	2	F2:119029	20210611-09_2021021-OK160A_816_5.3.236.mgf		0	0	0	0	94	124		
R.Q(-17.03)HSQFSDDNK.S	N	112.62	1187.4843	10	2.1	594.7482	2	13.73	2	F2:57082	20210611-09_2021021-OK160A_816_5.3.236.mgf	0	0	0	0	0	67	76	Pyro-glu from Q	Q1:Pyro-glu from Q:1000.00
K.ENGNNLPFANM(+15.99)DSAATDLGHFEPSAENEDGK.F	Y	108.80	3306.4006	31	11.4	1103.1488	3	43.68	2	F2:107793	20210611-09_2021021-OK160A_816_5.3.236.mgf		0	0	0	0	94	124		M11:Oxidation (M):1000.00
K.SPFDSEDGLGNFM(+15.99)NFM(+15.99)K.E	N	104.89	1966.8076	17	2.8	984.4097	2	45.96	2	F2:112336	20210611-09_2021021-OK160A_816_5.3.236.mgf	0	0	0	0	0	77	93		M13:Oxidation (M):1000.00; M16:Oxidation (M):1000.00
R.RQHSQFSDDNK.S	N	100.18	1360.6119	11	0.4	454.5429	3	6.44	2	F2:52336	20210611-09_2021021-OK160A_816_5.3.236.mgf		0	0	0	0	66	76		

total 11 peptides

Peptide	Uniq	-10lgP	Mass	Length	ppm	m/z	z	RT	Fraction	Scan	Source File	Area ebraeus	Area judaeus	#Feature	#Feature ebraeus	#Feature judaeus	Start	End	PTM	AScore
R.Q(-17.03)HSQFSDDNK(-.98).S	N	90.51	1186.5002	10	2.1	594.2562	2	12.84	2	F2:55863	20210611-09_2021021-OK160A_816_5.3.236.mgf		0	0	0	0	67	76	Amidation	Q1:Pyro-glu from Q:1000.00;K10:Amidation:1000.00
R.QHSQFSDDNK.S	N	87.11	1204.5109	10	-2.8	603.2585	2	8.72	2	F2:52560	20210611-09_2021021-OK160A_816_5.3.236.mgf		0	0	0	0	67	76		
K.SPFDSEDGLGNFMNFMK.E	N	83.66	1934.8179	17	0.7	968.4128	2	59.14	2	F2:131486	20210611-09_2021021-OK160A_816_5.3.236.mgf		0	0	0	0	77	93		
K.E(-18.01)NGNNLPFANMDSAATDLGHFEPSAENEDGK.F	Y	83.49	3272.3953	31	0.6	1091.8018	3	55.58	2	F2:127016	20210611-09_2021021-OK160A_816_5.3.236.mgf		0	0	0	0	94	124		E1:Pyro-glu from E:1000.00
K.SPFDSEDGLGNFM(+15.99)NFMK.E	N	76.38	1950.8127	17	2.4	976.4119	2	53.56	2	F2:124496	20210611-09_2021021-OK160A_816_5.3.236.mgf		0	0	0	0	77	93		M13:Oxidation (M):0.00
K.RRQHSQFSDDNK.S	N	72.54	1516.7130	12	10.8	506.5816	3	19.31	2	F2:64986	20210611-09_2021021-OK160A_816_5.3.236.mgf		0	0	0	0	65	76		
**total 11 peptides**																				

CebrOK033_J_011_B2

back to list

| **Protein Coverage** | **Supporting Peptides** |

Protein Coverage:

1 YPQRRDGAPA NAAPNLQPFN GEMAMPNMQP MHALPMSMSG MSSPQLMPLN PNLGMGFKRE NLEKRRQHSQ FSDDNKSPFD

a Amidation (-0.98)
P Pyro-glu from Q (-17.03)

81 SEDGLGNFMN FMKENGNNLP FANMDSAATD LGHFEPSAEN EDGKFRFFDK EQ

Supporting Peptides:

Peptide	Uniq	-10lgP	Mass	Length	ppm	m/z	z	RT	Fraction	Scan	Source File	Area ebraeus	Area judaeus	#Feature	#Feature ebraeus	#Feature judaeus	Start	End	PTM	AScore
K.ENGNNLPFANMDSAATDLGHFEPSAENEDGK.F	Y	125.47	3290.4058	31	10.2	1097.8158	3	50.09	2	F2:119029	20210611-09_2021021-OK160A_816_5.3.236.mgf		0	0	0	0	94	124		
R.Q(-17.03)HSQFSDDNK.S	N	112.62	1187.4843	10	2.1	594.7482	2	13.73	2	F2:57082	20210611-09_2021021-OK160A_816_5.3.236.mgf	0	0	0	0	0	67	76	Pyro-glu from Q	Q1:Pyro-glu from Q:1000.00
K.ENGNNLPFANM(+15.99)DSAATDLGHFEPSAENEDGK.F	Y	108.80	3306.4006	31	11.4	1103.1488	3	43.68	2	F2:107793	20210611-09_2021021-OK160A_816_5.3.236.mgf		0	0	0	0	94	124		M11:Oxidation (M):1000.00
K.SPFDSEDGLGNFM(+15.99)NFM(+15.99)K.E	N	104.89	1966.8076	17	2.8	984.4097	2	45.96	2	F2:112336	20210611-09_2021021-OK160A_816_5.3.236.mgf	0	0	0	0	0	77	93		M13:Oxidation (M):1000.00;M16:Oxidation (M):1000.00
R.RQHSQFSDDNK.S	N	100.18	1360.6119	11	0.4	454.5429	3	6.44	2	F2:52336	20210611-09_2021021-OK160A_816_5.3.236.mgf		0	0	0	0	66	76		
R.Q(-17.03)HSQFSDDNK(-.98).S	N	90.51	1186.5002	10	2.1	594.2562	2	12.84	2	F2:55863	20210611-09_2021021-OK160A_816_5.3.236.mgf		0	0	0	0	67	76	Amidation	Q1:Pyro-glu from Q:1000.00;K10:Amidation:1000.00
R.QHSQFSDDNK.S	N	87.11	1204.5109	10	-2.8	603.2585	2	8.72	2	F2:52560	20210611-09_2021021-OK160A_816_5.3.236.mgf		0	0	0	0	67	76		
K.SPFDSEDGLGNFMNFMK.E	N	83.66	1934.8179	17	0.7	968.4128	2	59.14	2	F2:131486	20210611-09_2021021-OK160A_816_5.3.236.mgf		0	0	0	0	77	93		
K.E(-18.01)NGNNLPFANMDSAATDLGHFEPSAENEDGK.F	Y	83.49	3272.3953	31	0.6	1091.8018	3	55.58	2	F2:127016	20210611-09_2021021-OK160A_816_5.3.236.mgf		0	0	0	0	94	124		E1:Pyro-glu from E:1000.00
K.SPFDSEDGLGNFM(+15.99)NFMK.E	N	76.38	1950.8127	17	2.4	976.4119	2	53.56	2	F2:124496	20210611-09_2021021-OK160A_816_5.3.236.mgf		0	0	0	0	77	93		M13:Oxidation (M):0.00
K.RRQHSQFSDDNK.S	N	72.54	1516.7130	12	10.8	506.5816	3	19.31	2	F2:64986	20210611-09_2021021-OK160A_816_5.3.236.mgf		0	0	0	0	65	76		
**total 11 peptides**																				

OK213_E_318_PPI

back to list

| **Protein Coverage** | **Supporting Peptides** |

Protein Coverage:

1 MLASGIVLIL LVHAFALADD KPKGPKVTEK VYFDIEIGGK AAGRIVIGLF GKTVPKTVEN FKQLAIGEKE QDGKKLTYKG

81 SKFHRVIKDF MIQGGDFTRG DGTGGRSIYG EKFPDENFKL KHYGSGWLSM ANSGKDTNGS QFFITTKKTS WLDGRHVVFG

161 TVLEGMDVVR KIEASKTDAR DKPEKDVVIA DCGALEVTKQ FSVEKSDAPE

Supporting Peptides:

Peptide	Uniq	-10lgP	Mass	Length	ppm	m/z	z	RT	Fraction	Scan	Source File	Area ebraeus	Area judaeus	#Feature	#Feature ebraeus	#Feature judaeus	Start	End	PTM	AScore	Found By
R.HVVFGTVLEGMDVVR.K	Y	113.44	1656.8657	15	-3.0	553.2919	3	47.54	2	F2:114501	20210611-09_2021021-OK160A_816_5.3.236.mgf		0	0	0	0	156	170			PEAKS DB
K.HYGSGWLSMANSGK.D	Y	102.18	1493.6721	14	-1.9	747.8388	2	32.18	2	F2:87133	20210611-09_2021021-OK160A_816_5.3.236.mgf		0	0	0	0	122	135			PEAKS DB
K.DTNGSQFFITTK.K	N	100.10	1357.6514	12	-0.1	679.8300	2	36.35	2	F2:94879	20210611-09_2021021-OK160A_816_5.3.236.mgf		0	0	0	0	136	147			PEAKS DB
K.DFMIQGGDFTR.G	Y	81.64	1285.5760	11	1.3	643.7935	2	41.06	2	F2:103152	20210611-09_2021021-OK160A_816_5.3.236.mgf		0	0	0	0	89	99			PEAKS DB
K.HYGSGWLSM(+15.99)ANSGK.D	Y	76.18	1509.6670	14	-0.9	755.8369	2	25.39	2	F2:75315	20210611-09_2021021-OK160A_816_5.3.236.mgf		0	0	0	0	122	135		M9:Oxidation (M):1000.00	PEAKS DB
K.VYFDIEIGGK.A	Y	74.78	1139.5862	10	-2.7	570.7964	2	44.43	2	F2:109368	20210611-09_2021021-OK160A_816_5.3.236.mgf		0	0	0	0	31	40			PEAKS DB
R.IVIGLFGK.T	N	64.69	845.5374	8	-0.9	423.7738	2	44.37	2	F2:109266	20210611-09_2021021-OK160A_816_5.3.236.mgf		0	0	0	0	45	52			PEAKS DB
K.KTSWLDGR.H	Y	59.35	961.4981	8	3.7	481.7561	2	20.75	2	F2:67312	20210611-09_2021021-OK160A_816_5.3.236.mgf		0	0	0	0	148	155			PEAKS DB
**total 8 peptides**																					

SRX1323887_E_240_PPI

back to list

| **Protein Coverage** | **Supporting Peptides** |

Protein Coverage:

1 MLASGVVLIL LVHAFALADD KPKGPKVTEK **VYFDIEIGGK** AAGR**IVIGLF GK**TVPKTVEN FKQLAIGEKE QDGKKLTYKG

81 SKFHRVIK**DF MIQGGDFTR**G DGTGGRSIYG EKFPDENFKL K**HYGSGWLSM ANSGKDTNGS QFFITTKKTS WLDGRHVVFG**

161 **TVLEGMDVVR** KIEASKTDAR DKPEKDVVIA DCGALEVTKQ FSVEKRDAPE

Supporting Peptides:

Peptide	Uniq	-10lgP	Mass	Length	ppm	m/z	z	RT	Fraction	Scan	Source File	Area ebraeus	Area judaeus	#Feature	#Feature ebraeus	#Feature judaeus	Start	End	PTM	AScore	Found By
R.HVVFGTVLEGMDVVR.K	Y	113.44	1656.8657	15	-3.0	553.2919	3	47.54	2	F2:114501	20210611-09_2021021-OK160A_816_5.3.236.mgf		0	0	0	0	156	170			PEAKS DB
K.HYGSGWLSMANSGK.D	Y	102.18	1493.6721	14	-1.9	747.8388	2	32.18	2	F2:87133	20210611-09_2021021-OK160A_816_5.3.236.mgf		0	0	0	0	122	135			PEAKS DB
K.DTNGSQFFITTK.K	N	100.10	1357.6514	12	-0.1	679.8300	2	36.35	2	F2:94879	20210611-09_2021021-OK160A_816_5.3.236.mgf		0	0	0	0	136	147			PEAKS DB
K.DFMIQGGDFTR.G	Y	81.64	1285.5760	11	1.3	643.7935	2	41.06	2	F2:103152	20210611-09_2021021-OK160A_816_5.3.236.mgf		0	0	0	0	89	99			PEAKS DB
K.HYGSGWLSM(+15.99)ANSGK.D	Y	76.18	1509.6670	14	-0.9	755.8369	2	25.39	2	F2:75315	20210611-09_2021021-OK160A_816_5.3.236.mgf		0	0	0	0	122	135		M9:Oxidation (M):1000.00	PEAKS DB
K.VYFDIEIGGK.A	Y	74.78	1139.5862	10	-2.7	570.7964	2	44.43	2	F2:109368	20210611-09_2021021-OK160A_816_5.3.236.mgf		0	0	0	0	31	40			PEAKS DB
R.IVIGLFGK.T	N	64.69	845.5374	8	-0.9	423.7738	2	44.37	2	F2:109266	20210611-09_2021021-OK160A_816_5.3.236.mgf		0	0	0	0	45	52			PEAKS DB
K.KTSWLDGR.H	Y	59.35	961.4981	8	3.7	481.7561	2	20.75	2	F2:67312	20210611-09_2021021-OK160A_816_5.3.236.mgf		0	0	0	0	148	155			PEAKS DB
**total 8 peptides**																					

CebrOK033_J_257_PPI

back to list

| Protein Coverage | Supporting Peptides |

Protein Coverage:

1 MLASGLVLIL LVHAFALADD KPKGPKVTEK **VYFDIEIGGK** AAGR**IVIGLF GK**TVPKTVEN FKQLAIGEKE QDGKKLTYKG

81 SKFHRVIK**DF MIQGGDFTR**G DGTGGRSIYG EKFPDENFKL K**HYGSGWLSM ANSGKDTNGS QFFITTKKTS WLDGRHVVFG**

161 **TVLEGMDVVR** KIEASKTDAR DKPENGCGAE D

Supporting Peptides:

Peptide	Uniq	-10lgP	Mass	Length	ppm	m/z	z	RT	Fraction	Scan	Source File	Area ebraeus	Area judaeus	#Feature	#Feature ebraeus	#Feature judaeus	Start	End	PTM	AScore	Found By
R.HVVFGTVLEGMDVVR.K	Y	113.44	1656.8657	15	-3.0	553.2919	3	47.54	2	F2:114501	20210611-09_2021021-OK160A_816_5.3.236.mgf		0	0	0	0	156	170			PEAKS DB
K.HYGSGWLSMANSGK.D	Y	102.18	1493.6721	14	-1.9	747.8388	2	32.18	2	F2:87133	20210611-09_2021021-OK160A_816_5.3.236.mgf		0	0	0	0	122	135			PEAKS DB
K.DTNGSQFFITTK.K	N	100.10	1357.6514	12	-0.1	679.8300	2	36.35	2	F2:94879	20210611-09_2021021-OK160A_816_5.3.236.mgf		0	0	0	0	136	147			PEAKS DB
K.DFMIQGGDFTR.G	Y	81.64	1285.5760	11	1.3	643.7935	2	41.06	2	F2:103152	20210611-09_2021021-OK160A_816_5.3.236.mgf		0	0	0	0	89	99			PEAKS DB
K.HYGSGWLSM(+15.99)ANSGK.D	Y	76.18	1509.6670	14	-0.9	755.8369	2	25.39	2	F2:75315	20210611-09_2021021-OK160A_816_5.3.236.mgf		0	0	0	0	122	135		M9:Oxidation (M):1000.00	PEAKS DB
K.VYFDIEIGGK.A	Y	74.78	1139.5862	10	-2.7	570.7964	2	44.43	2	F2:109368	20210611-09_2021021-OK160A_816_5.3.236.mgf		0	0	0	0	31	40			PEAKS DB
R.IVIGLFGK.T	N	64.69	845.5374	8	-0.9	423.7738	2	44.37	2	F2:109266	20210611-09_2021021-OK160A_816_5.3.236.mgf		0	0	0	0	45	52			PEAKS DB
K.KTSWLDGR.H	Y	59.35	961.4981	8	3.7	481.7561	2	20.75	2	F2:67312	20210611-09_2021021-OK160A_816_5.3.236.mgf		0	0	0	0	148	155			PEAKS DB
**total 8 peptides**																					

CebrOK033_J_256_PPI

back to list

| Protein Coverage | Supporting Peptides |

Protein Coverage:

1 MLASGLVLIL LVHAFALADD KPKGPKVTEK **VYFDIEIGGK** AAGR**IVIGLF GK**TVPKTVEN FKQLAIGEKE QDGKKLTYKG

81 SKFHRVIK**DF MIQGGDFTR**G DGTGGRSIYG EKFPDENFKL K**HYGSGWLSM ANSGKDTNGS QFFITTKKTS WLDGRHVVFG**

161 **TVLEGMDVVR** KIEASKTDAR DKPEKDVVIA DCGALEVTKQ FSVEKRDAPE

Supporting Peptides:

Peptide	Uniq	-10lgP	Mass	Length	ppm	m/z	z	RT	Fraction	Scan	Source File	Area ebraeus	Area judaeus	#Feature	#Feature ebraeus	#Feature judaeus	Start	End	PTM	AScore	Found By
R.HVVFGTVLEGMDVVR.K	Y	113.44	1656.8657	15	-3.0	553.2919	3	47.54	2	F2:114501	20210611-09_2021021-OK160A_816_5.3.236.mgf		0	0	0	0	156	170			PEAKS DB
K.HYGSGWLSMANSGK.D	Y	102.18	1493.6721	14	-1.9	747.8388	2	32.18	2	F2:87133	20210611-09_2021021-OK160A_816_5.3.236.mgf		0	0	0	0	122	135			PEAKS DB
K.DTNGSQFFITTK.K	N	100.10	1357.6514	12	-0.1	679.8300	2	36.35	2	F2:94879	20210611-09_2021021-OK160A_816_5.3.236.mgf		0	0	0	0	136	147			PEAKS DB
K.DFMIQGGDFTR.G	Y	81.64	1285.5760	11	1.3	643.7935	2	41.06	2	F2:103152	20210611-09_2021021-OK160A_816_5.3.236.mgf		0	0	0	0	89	99			PEAKS DB
K.HYGSGWLSM(+15.99)ANSGK.D	Y	76.18	1509.6670	14	-0.9	755.8369	2	25.39	2	F2:75315	20210611-09_2021021-OK160A_816_5.3.236.mgf		0	0	0	0	122	135		M9:Oxidation (M):1000.00	PEAKS DB
K.VYFDIEIGGK.A	Y	74.78	1139.5862	10	-2.7	570.7964	2	44.43	2	F2:109368	20210611-09_2021021-OK160A_816_5.3.236.mgf		0	0	0	0	31	40			PEAKS DB
R.IVIGLFGK.T	N	64.69	845.5374	8	-0.9	423.7738	2	44.37	2	F2:109266	20210611-09_2021021-OK160A_816_5.3.236.mgf		0	0	0	0	45	52			PEAKS DB
K.KTSWLDGR.H	Y	59.35	961.4981	8	3.7	481.7561	2	20.75	2	F2:67312	20210611-09_2021021-OK160A_816_5.3.236.mgf		0	0	0	0	148	155			PEAKS DB
**total 8 peptides**																					

OK005_J_311_PPI

back to list

| Protein Coverage | Supporting Peptides |

Protein Coverage:

1 MLASGLVLIL LVHAFALADD KPKGPKVTEK **VYFDIEIGGK** AAGR**IVIGLF GK**TVPKTVEN FKQLAIGEKE QDGKKLTYKG

81 SKFHRVIK**DF MIQGGDFTR**G DGTGGRSIYG EKFPDENFKL K**HYGSGWLSM ANSGKDTNGS QFFITTKKTS WLDGRHVVFG**

161 **TVLEGMDVVR** KIEASKTDAR DKPEKDVVIA DCGALEVTKQ FSVEKRDAPE

Supporting Peptides:

Peptide	Uniq	-10lgP	Mass	Length	ppm	m/z	z	RT	Fraction	Scan	Source File	Area ebraeus	Area judaeus	#Feature	#Feature ebraeus	#Feature judaeus	Start	End	PTM	AScore	Found By
R.HVVFGTVLEGMDVVR.K	Y	113.44	1656.8657	15	-3.0	553.2919	3	47.54	2	F2:114501	20210611-09_2021021-OK160A_816_5.3.236.mgf		0	0	0	0	156	170			PEAKS DB
K.HYGSGWLSMANSGK.D	Y	102.18	1493.6721	14	-1.9	747.8388	2	32.18	2	F2:87133	20210611-09_2021021-OK160A_816_5.3.236.mgf		0	0	0	0	122	135			PEAKS DB
K.DTNGSQFFITTK.K	N	100.10	1357.6514	12	-0.1	679.8300	2	36.35	2	F2:94879	20210611-09_2021021-OK160A_816_5.3.236.mgf		0	0	0	0	136	147			PEAKS DB
K.DFMIQGGDFTR.G	Y	81.64	1285.5760	11	1.3	643.7935	2	41.06	2	F2:103152	20210611-09_2021021-OK160A_816_5.3.236.mgf		0	0	0	0	89	99			PEAKS DB

total 8 peptides

Peptide	Uniq	-10lgP	Mass	Length	ppm	m/z	z	RT	Fraction	Scan	Source File	Area ebraeus	Area judaeus	#Feature	#Feature ebraeus	#Feature judaeus	Start	End	PTM	AScore	Found By
K.HYGSGWLSM(+15.99)ANSGK.D	Y	76.18	1509.6670	14	-0.9	755.8369	2	25.39	2	F2:75315	20210611-09_2021021-OK160A_816_5.3.236.mgf		0	0	0	0	122	135		M9:Oxidation (M):1000.00	PEAKS DB
K.VYFDIEIGGK.A	Y	74.78	1139.5862	10	-2.7	570.7964	2	44.43	2	F2:109368	20210611-09_2021021-OK160A_816_5.3.236.mgf		0	0	0	0	31	40			PEAKS DB
R.IVIGLFGK.T	N	64.69	845.5374	8	-0.9	423.7738	2	44.37	2	F2:109266	20210611-09_2021021-OK160A_816_5.3.236.mgf		0	0	0	0	45	52			PEAKS DB
K.KTSWLDGR.H	Y	59.35	961.4981	8	3.7	481.7561	2	20.75	2	F2:67312	20210611-09_2021021-OK160A_816_5.3.236.mgf		0	0	0	0	148	155			PEAKS DB
**total 8 peptides**																					

OK006_E_286_PPI

back to list

| Protein Coverage | Supporting Peptides |

Protein Coverage:

1 MLASGIVLIL LVHAFALADD KPKGPKVTEK **VYFDIEIGGK** AAGR**IVIGLF GK**TVPKTVEN FKQLAIGEKE QDGKKLTYKG

81 SKFHRVIK**DF MIQGGDFTR**G DGTGGRSIYG EKFPDENFKL K**HYGSGWLSM ANSGKDTNGS QFFITTKKTS WLDGRHVVFG**

161 **TVLEGMDVVR** KIEASKTDAR DKPEKDVVIA DCGALEVTKQ FSVEKRDAPE

Supporting Peptides:

Peptide	Uniq	-10lgP	Mass	Length	ppm	m/z	z	RT	Fraction	Scan	Source File	Area ebraeus	Area judaeus	#Feature	#Feature ebraeus	#Feature judaeus	Start	End	PTM	AScore	Found By
R.HVVFGTVLEGMDVVR.K	Y	113.44	1656.8657	15	-3.0	553.2919	3	47.54	2	F2:114501	20210611-09_2021021-OK160A_816_5.3.236.mgf		0	0	0	0	156	170			PEAKS DB
K.HYGSGWLSMANSGK.D	Y	102.18	1493.6721	14	-1.9	747.8388	2	32.18	2	F2:87133	20210611-09_2021021-OK160A_816_5.3.236.mgf		0	0	0	0	122	135			PEAKS DB
K.DTNGSQFFITTK.K	N	100.10	1357.6514	12	-0.1	679.8300	2	36.35	2	F2:94879	20210611-09_2021021-OK160A_816_5.3.236.mgf		0	0	0	0	136	147			PEAKS DB
K.DFMIQGGDFTR.G	Y	81.64	1285.5760	11	1.3	643.7935	2	41.06	2	F2:103152	20210611-09_2021021-OK160A_816_5.3.236.mgf		0	0	0	0	89	99			PEAKS DB
K.HYGSGWLSM(+15.99)ANSGK.D	Y	76.18	1509.6670	14	-0.9	755.8369	2	25.39	2	F2:75315	20210611-09_2021021-OK160A_816_5.3.236.mgf		0	0	0	0	122	135		M9:Oxidation (M):1000.00	PEAKS DB
K.VYFDIEIGGK.A	Y	74.78	1139.5862	10	-2.7	570.7964	2	44.43	2	F2:109368	20210611-09_2021021-OK160A_816_5.3.236.mgf		0	0	0	0	31	40			PEAKS DB
R.IVIGLFGK.T	N	64.69	845.5374	8	-0.9	423.7738	2	44.37	2	F2:109266	20210611-09_2021021-OK160A_816_5.3.236.mgf		0	0	0	0	45	52			PEAKS DB
K.KTSWLDGR.H	Y	59.35	961.4981	8	3.7	481.7561	2	20.75	2	F2:67312	20210611-09_2021021-OK160A_816_5.3.236.mgf		0	0	0	0	148	155			PEAKS DB
**total 8 peptides**																					

OK193_E_264_PPI

back to list

| Protein Coverage | Supporting Peptides |

Protein Coverage:

1 MLASGIVLIL LVHAFALADD KPKGPKVTEK **VYFDIEIGGK** AAGR**IVIGLF GK**TVPKTVEN FKQLAIGEKE QDGKKLTYKG

81 SKFHRVIK**DF MIQGGDFTR**G DGTGGRSIYG EKFPDENFKL K**HYGSGWLSM ANSGKDTNGS QFFITTKKTS WLDGRHVVFG**

161 **TVLEGMDVVR** KIEASKTDAR DKPEKDVVIA DCGALEVTKQ FSVEKRDAPE

Supporting Peptides:

Peptide	Uniq	-10lgP	Mass	Length	ppm	m/z	z	RT	Fraction	Scan	Source File	Area ebraeus	Area judaeus	#Feature	#Feature ebraeus	#Feature judaeus	Start	End	PTM	AScore	Found By
R.HVVFGTVLEGMDVVR.K	Y	113.44	1656.8657	15	-3.0	553.2919	3	47.54	2	F2:114501	20210611-09_2021021-OK160A_816_5.3.236.mgf		0	0	0	0	156	170			PEAKS DB
K.HYGSGWLSMANSGK.D	Y	102.18	1493.6721	14	-1.9	747.8388	2	32.18	2	F2:87133	20210611-09_2021021-OK160A_816_5.3.236.mgf		0	0	0	0	122	135			PEAKS DB
K.DTNGSQFFITTK.K	N	100.10	1357.6514	12	-0.1	679.8300	2	36.35	2	F2:94879	20210611-09_2021021-OK160A_816_5.3.236.mgf		0	0	0	0	136	147			PEAKS DB
K.DFMIQGGDFTR.G	Y	81.64	1285.5760	11	1.3	643.7935	2	41.06	2	F2:103152	20210611-09_2021021-OK160A_816_5.3.236.mgf		0	0	0	0	89	99			PEAKS DB
K.HYGSGWLSM(+15.99)ANSGK.D	Y	76.18	1509.6670	14	-0.9	755.8369	2	25.39	2	F2:75315	20210611-09_2021021-OK160A_816_5.3.236.mgf		0	0	0	0	122	135		M9:Oxidation (M):1000.00	PEAKS DB
K.VYFDIEIGGK.A	Y	74.78	1139.5862	10	-2.7	570.7964	2	44.43	2	F2:109368	20210611-09_2021021-OK160A_816_5.3.236.mgf		0	0	0	0	31	40			PEAKS DB
R.IVIGLFGK.T	N	64.69	845.5374	8	-0.9	423.7738	2	44.37	2	F2:109266	20210611-09_2021021-OK160A_816_5.3.236.mgf		0	0	0	0	45	52			PEAKS DB
K.KTSWLDGR.H	Y	59.35	961.4981	8	3.7	481.7561	2	20.75	2	F2:67312	20210611-09_2021021-OK160A_816_5.3.236.mgf		0	0	0	0	148	155			PEAKS DB
**total 8 peptides**																					

OK214_J_328_PPI

back to list

| Protein Coverage | Supporting Peptides |

Protein Coverage:

1 MLASGLVLIL LVHAFALADD KPKGPKVTEK **VYFDIEIGGK** AAGR**IVIGLF GK**TVPKTVEN FKQLAIGEKE QDGKKLTYKG

81 SKFHRVIK**DF MIQGGDFTR**G DGTGGRSIYG EKFPDENFKL K**HYGSGWLSM ANSGKDTNGS QFFITTKKTS WLDGRHVVFG**

161 **TVLEGMDVVR** KIEASKTDAR DKPEKDVVIA DCGALEVTKQ FSVEKRDAPE

Supporting Peptides:

Peptide	Uniq	-10lgP	Mass	Length	ppm	m/z	z	RT	Fraction	Scan	Source File	Area ebraeus	Area judaeus	#Feature	#Feature ebraeus	#Feature judaeus	Start	End	PTM	AScore	Found By
R.HVVFGTVLEGMDVVR.K	Y	113.44	1656.8657	15	-3.0	553.2919	3	47.54	2	F2:114501	20210611-09_2021021-OK160A_816_5.3.236.mgf		0	0	0	0	156	170			PEAKS DB
K.HYGSGWLSMANSGK.D	Y	102.18	1493.6721	14	-1.9	747.8388	2	32.18	2	F2:87133	20210611-09_2021021-OK160A_816_5.3.236.mgf		0	0	0	0	122	135			PEAKS DB
K.DTNGSQFFITTK.K	N	100.10	1357.6514	12	-0.1	679.8300	2	36.35	2	F2:94879	20210611-09_2021021-OK160A_816_5.3.236.mgf		0	0	0	0	136	147			PEAKS DB
K.DFMIQGGDFTR.G	Y	81.64	1285.5760	11	1.3	643.7935	2	41.06	2	F2:103152	20210611-09_2021021-OK160A_816_5.3.236.mgf		0	0	0	0	89	99			PEAKS DB
K.HYGSGWLSM(+15.99)ANSGK.D	Y	76.18	1509.6670	14	-0.9	755.8369	2	25.39	2	F2:75315	20210611-09_2021021-OK160A_816_5.3.236.mgf		0	0	0	0	122	135		M9:Oxidation (M):1000.00	PEAKS DB
K.VYFDIEIGGK.A	Y	74.78	1139.5862	10	-2.7	570.7964	2	44.43	2	F2:109368	20210611-09_2021021-OK160A_816_5.3.236.mgf		0	0	0	0	31	40			PEAKS DB
R.IVIGLFGK.T	N	64.69	845.5374	8	-0.9	423.7738	2	44.37	2	F2:109266	20210611-09_2021021-OK160A_816_5.3.236.mgf		0	0	0	0	45	52			PEAKS DB
K.KTSWLDGR.H	Y	59.35	961.4981	8	3.7	481.7561	2	20.75	2	F2:67312	20210611-09_2021021-OK160A_816_5.3.236.mgf		0	0	0	0	148	155			PEAKS DB
**total 8 peptides**																					

OK212_J_315_PPI

back to list

| Protein Coverage | Supporting Peptides |

Protein Coverage:

1 MLASGLVLIL LVHAFALADD KPKGPKVTEK VYFDIEIGGK AAGRIVIGLF GKTVPKTVEN FKQLAVGEKE QDGKKLTYKG

81 SKFHRVIKDF MIQGGDFTRG DGTGGRSIYG EKFPDENFKL KHYGSGWLSM ANSGKDTNGS QFFITTKKTS WLDGRHVVFG

161 TVLEGMDVVR KIEASKTDAR DKPEKDVVIA DCGALEVTKQ FSVEKRDAPE

Supporting Peptides:

Peptide	Uniq	-10lgP	Mass	Length	ppm	m/z	z	RT	Fraction	Scan	Source File	Area ebraeus	Area judaeus	#Feature	#Feature ebraeus	#Feature judaeus	Start	End	PTM	AScore	Found By
R.HVVFGTVLEGMDVVR.K	Y	113.44	1656.8657	15	-3.0	553.2919	3	47.54	2	F2:114501	20210611-09_2021021-OK160A_816_5.3.236.mgf		0	0	0	0	156	170			PEAKS DB
K.HYGSGWLSMANSGK.D	Y	102.18	1493.6721	14	-1.9	747.8388	2	32.18	2	F2:87133	20210611-09_2021021-OK160A_816_5.3.236.mgf		0	0	0	0	122	135			PEAKS DB
K.DTNGSQFFITTK.K	N	100.10	1357.6514	12	-0.1	679.8300	2	36.35	2	F2:94879	20210611-09_2021021-OK160A_816_5.3.236.mgf		0	0	0	0	136	147			PEAKS DB
K.DFMIQGGDFTR.G	Y	81.64	1285.5760	11	1.3	643.7935	2	41.06	2	F2:103152	20210611-09_2021021-OK160A_816_5.3.236.mgf		0	0	0	0	89	99			PEAKS DB
K.HYGSGWLSM(+15.99)ANSGK.D	Y	76.18	1509.6670	14	-0.9	755.8369	2	25.39	2	F2:75315	20210611-09_2021021-OK160A_816_5.3.236.mgf		0	0	0	0	122	135		M9:Oxidation (M):1000.00	PEAKS DB
K.VYFDIEIGGK.A	Y	74.78	1139.5862	10	-2.7	570.7964	2	44.43	2	F2:109368	20210611-09_2021021-OK160A_816_5.3.236.mgf		0	0	0	0	31	40			PEAKS DB
R.IVIGLFGK.T	N	64.69	845.5374	8	-0.9	423.7738	2	44.37	2	F2:109266	20210611-09_2021021-OK160A_816_5.3.236.mgf		0	0	0	0	45	52			PEAKS DB
K.KTSWLDGR.H	Y	59.35	961.4981	8	3.7	481.7561	2	20.75	2	F2:67312	20210611-09_2021021-OK160A_816_5.3.236.mgf		0	0	0	0	148	155			PEAKS DB
**total 8 peptides**																					

gene:107297086

back to list

| Protein Coverage | Supporting Peptides |

Protein Coverage:

1 MSGRGKGGKG LGKGGAKRHR KVLRDNIQGI TKPAIRRLAR RGGVKRISGL IYEETRGVLK VFLENVIRDA VTYTEHAKRK

Oxidation (M) (+15.99)

81 TVTAMDVVYA LKRQGRTLPT SLQLRLQLPE MARTKQTARK STGGKAPRKQ LATKAARKSA PATGGVKKPH RYRPGTVALR

161 EIRRYQKSTE LLIRKLPFQR LVREIAQDFK TDLRFQSSAV MALQEASEAY LVGLFEDTNL CAIHAKRVTI MPKDIQLARR

241 IRGERA

Supporting Peptides:

Peptide	Uniq	-10lgP	Mass	Length	ppm	m/z	z	RT	Fraction	Scan	Source File	Area ebraeus	Area judaeus	#Feature	#Feature ebraeus	#Feature judaeus	Start	End	PTM	AScore	Found By
R.DNIQGITKPAIR.R	Y	108.08	1324.7462	12	-0.2	663.3777	2	26.45	1	F1:20382	20210611-08_2021021-OK192A_815_5.3.236.mgf	0	0	0	0	0	25	36			PEAKS DB
K.TVTAM(+15.99)DVVYALK.R	Y	94.21	1325.6901	12	1.3	663.8504	2	41.63	2	F2:104257	20210611-09_2021021-OK160A_816_5.3.236.mgf	0	0	0	0	0	81	92	Oxidation (M)	M5:Oxidation (M):1000.00	PEAKS DB
K.TVTAMDVVYALK.R	Y	92.03	1309.6952	12	-2.5	655.8508	2	49.31	1	F1:46803	20210611-08_2021021-OK192A_815_5.3.236.mgf	0	0	0	0	0	81	92			PEAKS DB
R.ISGLIYEETR.G	Y	86.83	1179.6135	10	2.0	590.8127	2	32.27	2	F2:87322	20210611-09_2021021-OK160A_816_5.3.236.mgf	0	0	0	0	0	47	56			PEAKS DB
R.DAVTYTEHAK.R	Y	81.33	1133.5353	10	-0.7	567.7722	2	14.83	2	F2:58952	20210611-09_2021021-OK160A_816_5.3.236.mgf	0	0	0	0	0	69	78			PEAKS DB
K.VFLENVIR.D	Y	78.88	988.5706	8	0.0	495.2905	2	40.54	2	F2:102326	20210611-09_2021021-OK160A_816_5.3.236.mgf	0	0	0	0	0	61	68			PEAKS DB
R.YRPGTVALR.E	Y	76.80	1031.5875	9	3.5	344.8697	3	20.19	1	F1:11651	20210611-08_2021021-OK192A_815_5.3.236.mgf	0	0	0	0	0	152	160			PEAKS DB
K.STELLIR.K	Y	63.42	830.4861	7	1.0	416.2490	2	28.31	2	F2:80330	20210611-09_2021021-OK160A_816_5.3.236.mgf	0	0	0	0	0	168	174			PEAKS DB
R.EIAQDFK.T	Y	53.31	849.4232	7	4.6	425.7192	2	20.63	1	F1:12095	20210611-08_2021021-OK192A_815_5.3.236.mgf	0		0	0	0	184	190			PEAKS DB
**total 9 peptides**																					

OK193_E_277_Thioredoxin-like

back to list

| Protein Coverage | Supporting Peptides |

Protein Coverage:

1 MAQIGQLAIQ KPAPEFSTQA VVNGEFQTVK LSQYRGKYVV LFFYPLDFTF VCPTEIIAFS DRAEEFRSIN CEVLAASTDS

Oxidation (M) (+15.99)

81 VFSHLAWVNT PRKQGGLGNM NIPLLADKTL DISRQYGCLK EDEGVSYRAL YIIDDKGNLR QITMNDLPVG RSVDETLRLV

161 QAFQFTDKHG DSDVRVVCPA GWKPGSDTIK PTVKDSKNYF TKAN

Supporting Peptides:

Peptide	Uniq	-10lgP	Mass	Length	ppm	m/z	z	RT	Fraction	Scan	Source File	Area ebraeus	Area judaeus	#Feature	#Feature ebraeus	#Feature judaeus	Start	End	PTM	AScore	Found By
K.QGGLGNMNIPLLADK.T	N	116.97	1539.8079	15	-0.5	770.9076	2	49.54	2	F2:118008	20210611-09_2021021-OK160A_816_5.3.236.mgf		0	0	0	0	94	108			PEAKS DB
R.Q(-17.03)ITMNDLPVGR.S	N	105.67	1225.6125	11	-0.1	613.8109	2	46.97	2	F2:113637	20210611-09_2021021-OK160A_816_5.3.236.mgf		0	0	0	0	141	151		Q1:Pyro-glu from Q:1000.00	PEAKS DB
R.QITMNDLPVGR.S	N	94.09	1242.6390	11	0.8	622.3250	2	36.64	1	F1:34092	20210611-08_2021021-OK192A_815_5.3.236.mgf	0	0	0	0	0	141	151			PEAKS DB
R.QITM(+15.99)NDLPVGR.S	N	91.16	1258.6339	11	1.7	630.3226	2	27.61	2	F2:79161	20210611-09_2021021-OK160A_816_5.3.236.mgf		0	0	0	0	141	151	Oxidation (M)	M4:Oxidation (M):1000.00	PEAKS DB
R.LVQAFQFTDK.H	N	90.87	1195.6237	10	0.1	598.8167	2	37.25	2	F2:96539	20210611-09_2021021-OK160A_816_5.3.236.mgf	0	0	0	0	0	159	168			PEAKS DB
R.KQGGLGNMNIPLLADK.T	N	73.18	1667.9028	16	-0.3	556.9726	3	43.41	1	F1:41299	20210611-08_2021021-OK192A_815_5.3.236.mgf	0	0	0	0	0	93	108			PEAKS DB
R.ALYIIDDK.G	N	72.37	949.5120	8	1.0	475.7617	2	33.17	2	F2:89147	20210611-09_2021021-OK160A_816_5.3.236.mgf	0	0	0	0	0	129	136			PEAKS DB
K.EDEGVSYR.A	Y	67.66	953.4090	8	5.4	477.7123	2	15.59	2	F2:59546	20210611-09_2021021-OK160A_816_5.3.236.mgf		0	0	0	0	121	128			PEAKS DB
**total 8 peptides**																					

gene:107292429

back to list

| Protein Coverage | Supporting Peptides |

Protein Coverage:

1 MADQLTEEQI AEFKEAFSLF DKDGDGTITT KELGTVMRSL GQNPTEAELQ DMINEVDADG NGTIDFPEFL TMMARKMKDT

Oxidation (M) (+15.99)

81 DSEEEIREAF RVFDKDGNGY ISSAELRHVM TNLGEKLTDE EVDEMIREAD IDGDGQVNYE EFVQMMTAK

Supporting Peptides:

Peptide	Uniq	-10lgP	Mass	Length	ppm	m/z	z	RT	Fraction	Scan	Source File	Area ebraeus	Area judaeus	#Feature	#Feature ebraeus	#Feature judaeus	Start	End	PTM	AScore	Found By
K.EAFSLFDKDGDGTITTK.E	Y	109.55	1843.8839	17	-1.4	615.6318	3	39.27	2	F2:100164	20210611-09_2021021-OK160A_816_5.3.236.mgf	0	0	0	0	0	15	31			PEAKS DB
**total 9 peptides**																					

Peptide	Uniq	-10lgP	Mass	Length	ppm	m/z	z	RT	Fraction	Scan	Source File	Area ebraeus	Area judaeus	#Feature	#Feature ebraeus	#Feature judaeus	Start	End	PTM	AScore	Found By
R.HVMTNLGEK.L	Y	97.43	1027.5121	9	1.2	514.7618	2	15.04	2	F2:59006	20210611-09_2021021-OK160A_816_5.3.236.mgf	0	0	0	0	0	108	116			PEAKS DB
K.MKDTDSEEEIR.E	Y	92.20	1351.5925	11	-3.8	676.7982	2	14.72	2	F2:58511	20210611-09_2021021-OK160A_816_5.3.236.mgf		0	0	0	0	77	87			PEAKS DB
K.M(+15.99)KDTDSEEEIR.E	Y	87.58	1367.5874	11	-0.8	684.7975	2	12.59	2	F2:55641	20210611-09_2021021-OK160A_816_5.3.236.mgf	0	0	0	0	0	77	87		M1:Oxidation (M):1000.00	PEAKS DB
K.EAFSLFDK.D	Y	85.63	955.4650	8	2.4	478.7391	2	40.32	1	F1:37871	20210611-08_2021021-OK192A_815_5.3.236.mgf	0	0	0	0	0	15	22			PEAKS DB
R.HVM(+15.99)TNLGEK.L	Y	85.36	1043.5070	9	1.4	522.7595	2	11.64	1	F1:2810	20210611-08_2021021-OK192A_815_5.3.236.mgf	0		0	0	0	108	116	Oxidation (M)	M3:Oxidation (M):1000.00	PEAKS DB
K.LTDEEVDEM(+15.99)IR.E	Y	80.09	1364.6129	11	1.6	683.3123	2	27.07	1	F1:21221	20210611-08_2021021-OK192A_815_5.3.236.mgf	0		0	0	0	117	127		M9:Oxidation (M):1000.00	PEAKS DB
K.LTDEEVDEMIR.E	Y	73.97	1348.6180	11	1.6	675.3148	2	36.96	1	F1:34272	20210611-08_2021021-OK192A_815_5.3.236.mgf	0	0	0	0	0	117	127			PEAKS DB
K.DTDSEEEIR.E	Y	72.34	1092.4570	9	0.6	547.2341	2	15.71	1	F1:6943	20210611-08_2021021-OK192A_815_5.3.236.mgf	0	0	0	0	0	79	87			PEAKS DB
total 9 peptides																					

P_096_Elongation

back to list

| Protein Coverage | Supporting Peptides |

Protein Coverage:

```
  1 MGGKDKTHIN IVVIGHVDSG KSTSTGHLIY KCGGIDKRVI EKFEKEAQEM GKGSFKYAWV LDKLKAERER GITIDIALWK
 81 FETEKYYVTI IDAPGHRDFI KNMITGTSQA DCAVLIVAAG TGEFEAGISK EGQTREHALL AYTLGVKQLI VAVNKMDSTE
161 PPYSEDRFNE IQKEVGTYIK KIGYNPKAVA FVPISGWHGD NMLEASEKMP WNKKGWLIER KEGKAEGRTL LQALDAILPP
241 QRPTDKPLRL PLQDVYKIGG IGTVPVGRVE TGVLKPGTVV TFAPANVTTE VKSVEMHHES LPEALPGDNV GFNVKNVSVK
321 DIRRGNVAGD SKNDPPKEAK SFYAQVIILN HPGEIKNGYA PVLDCHTAHI ACKFTEIKEK CDRRSGKKLE DNPKAVKSGD
401 AAMVELTPSK PMCVEAFSDY PPLGRFAVRD MKQTVAVGVI KSVTKAEATG GKVTKSAQKK K
```

Supporting Peptides:

Peptide	Uniq	-10lgP	Mass	Length	ppm	m/z	z	RT	Fraction	Scan	Source File	Area ebraeus	Area judaeus	#Feature	#Feature ebraeus	#Feature judaeus	Start	End	PTM	AScore	Found By
R.EHALLAYTLGVK.Q	Y	117.36	1313.7343	12	2.5	657.8733	2	38.07	2	F2:98035	20210611-09_2021021-OK160A_816_5.3.236.mgf		0	0	0	0	136	147			PEAKS DB
K.YYVTIIDAPGHR.D	Y	115.80	1403.7197	12	0.2	702.8643	2	33.96	2	F2:90405	20210611-09_2021021-OK160A_816_5.3.236.mgf	0	0	0	0	0	86	97			PEAKS DB
K.IGGIGTVPVGR.V	Y	83.39	1024.6029	11	4.9	513.3091	2	30.73	2	F2:84552	20210611-09_2021021-OK160A_816_5.3.236.mgf	0	0	0	0	0	258	268			PEAKS DB
K.QTVAVGVIK.S	Y	80.56	913.5596	9	2.0	457.7863	2	26.21	1	F1:20012	20210611-08_2021021-OK192A_815_5.3.236.mgf	0	0	0	0	0	433	441			PEAKS DB
R.LPLQDVYK.I	Y	67.87	974.5436	8	6.2	488.2801	2	32.37	2	F2:87332	20210611-09_2021021-OK160A_816_5.3.236.mgf	0	0	0	0	0	250	257			PEAKS DB
total 5 peptides																					

Pvau2_100

back to list

| Protein Coverage | Supporting Peptides |

Protein Coverage:

```
  1 GEEACYSYAG GSVYPQETRR TSGHTIQWSQ AVISKPAPDW NGTAVVNGEF KDIKLTDYRG KYLVFFFYPL DFTFVCPTEI
 81 VAFSDNVDKF NSINTEVVAC SVDSQFTHLA WINTPRNQGG LGPLKIPLLS DLTHDIAKAY GVYLQDLGHS LRGLFIIDPK
161 GILRQITMND LPVGRSVEET LRLVQAFQYT DSHGEVCPVN WKPGADTIIP DPKNAKQYF
```

o Oxidation (M) (+15.99)

Supporting Peptides:

Peptide	Uniq	-10lgP	Mass	Length	ppm	m/z	z	RT	Fraction	Scan	Source File	Area ebraeus	Area judaeus	#Feature	#Feature ebraeus	#Feature judaeus	Start	End	PTM	AScore	Found By
K.IPLLSDLTHDIAK.A	Y	122.43	1434.8082	13	-2.7	718.4064	2	49.50	2	F2:117962	20210611-09_2021021-OK160A_816_5.3.236.mgf	0	0	0	0	0	126	138			PEAKS DB
K.AYGVYLQDLGHSLR.G	Y	112.55	1590.8154	14	2.1	796.4133	2	41.92	2	F2:104705	20210611-09_2021021-OK160A_816_5.3.236.mgf		0	0	0	0	139	152			PEAKS DB
R.Q(-17.03)ITMNDLPVGR.S	N	105.67	1225.6125	11	-0.1	613.8109	2	46.97	2	F2:113637	20210611-09_2021021-OK160A_816_5.3.236.mgf		0	0	0	0	165	175		Q1:Pyro-glu from Q:1000.0 0	PEAKS DB
R.QITMNDLPVGR.S	N	94.09	1242.6390	11	0.8	622.3250	2	36.64	1	F1:34092	20210611-08_2021021-OK192A_815_5.3.236.mgf	0	0	0	0	0	165	175			PEAKS DB
R.QITM(+15.99)NDLPVGR.S	N	91.16	1258.6339	11	1.7	630.3226	2	27.61	2	F2:79161	20210611-09_2021021-OK160A_816_5.3.236.mgf		0	0	0	0	165	175	Oxidation (M)	M4:Oxidation (M):1000.00	PEAKS DB
R.GLFIIDPK.G	Y	76.34	901.5273	8	0.5	451.7693	2	42.96	2	F2:106624	20210611-09_2021021-OK160A_816_5.3.236.mgf	0	0	0	0	0	153	160			PEAKS DB
total 6 peptides																					

OK213_E_018_B2

back to list

| Protein Coverage | Supporting Peptides |

Protein Coverage:

```
  1 FPQRRDGAPA NAAPNLQPFN GEMAMPNMQP MHALPMSMSG MSSPQLMPLN PNLGMGFKRE NLEKRRQHSQ FSDDNKSPFD
 81 SEDGLGNFMN FMKENGNNLP FADVNSAATD LGHFEPSAEN EDGKFRFFD
```

a Amidation (-0.98)
P Pyro-glu from Q (-17.03)

Supporting Peptides:

Peptide	Uniq	-10lgP	Mass	Length	ppm	m/z	z	RT	Fraction	Scan	Source File	Area ebraeus	Area judaeus	#Feature	#Feature ebraeus	#Feature judaeus	Start	End	PTM	AScore	Found By
R.Q(-17.03)HSQFSDDNK.S	N	112.62	1187.4843	10	2.1	594.7482	2	13.73	2	F2:57082	20210611-09_2021021-OK160A_816_5.3.236.mgf	0	0	0	0	0	67	76	Pyro-glu from Q	Q1:Pyro-glu from Q:1000.00	PEAKS DB
K.SPFDSEDGLGNFM(+15.99)NFM(+15.99)K.E	N	104.89	1966.8076	17	2.8	984.4097	2	45.96	2	F2:112336	20210611-09_2021021-OK160A_816_5.3.236.mgf	0	0	0	0	0	77	93		M13:Oxidation(M):1000.00;M16:Oxidation(M):1000.00	PEAKS DB
R.RQHSQFSDDNK.S	N	100.18	1360.6119	11	0.4	454.5429	3	6.44	2	F2:52336	20210611-09_2021021-OK160A_816_5.3.236.mgf		0	0	0	0	66	76			PEAKS DB
R.Q(-17.03)HSQFSDDNK(-.98).S	N	90.51	1186.5002	10	2.1	594.2562	2	12.84	2	F2:55863	20210611-09_2021021-OK160A_816_5.3.236.mgf		0	0	0	0	67	76	Amidation	Q1:Pyro-glu from Q:1000.00;K10:Amidation:1000.00	PEAKS DB
R.QHSQFSDDNK.S	N	87.11	1204.5109	10	-2.8	603.2585	2	8.72	2	F2:52560	20210611-09_2021021-OK160A_816_5.3.236.mgf		0	0	0	0	67	76			PEAKS DB
K.SPFDSEDGLGNFMNFMK.E	N	83.66	1934.8179	17	0.7	968.4128	2	59.14	2	F2:131486	20210611-09_2021021-OK160A_816_5.3.236.mgf		0	0	0	0	77	93			PEAKS DB
total 9 peptides																					

Peptide	Uniq	-10lgP	Mass	Length	ppm	m/z	z	RT	Fraction	Scan	Source File	Area ebraeus	Area judaeus	#Feature	#Feature ebraeus	#Feature judaeus	Start	End	PTM	AScore	Found By
K.SPFDSEDGLGNFM(+15.99)NFMK.E	N	76.38	1950.8127	17	2.4	976.4119	2	53.56	2	F2:124496	20210611-09_2021021-OK160A_816_5.3.236.mgf		0	0	0	0	77	93		M13:Oxidation (M):0.00	PEAKS DB
K.RRQHSQFSDDNK.S	N	72.54	1516.7130	12	10.8	506.5816	3	19.31	2	F2:64986	20210611-09_2021021-OK160A_816_5.3.236.mgf		0	0	0	0	65	76			PEAKS DB
K.ENGNNLPFADVNSAATDLGHFEPSAENEDGK.F	Y	63.75	3258.4336	31	8.0	1087.1564	3	53.53	1	F1:49273	20210611-08_2021021-OK192A_815_5.3.236.mgf	0		0	0	0	94	124			PEAKS DB
**total 9 peptides**																					

OK006_E_036_F

back to list

| Protein Coverage | Supporting Peptides |

Protein Coverage:

1 EEYDLNDPDV LAIVDEGQKL MHACSIANKY TYDPWWKLNI LAFKEMRVYH SMMSDMVTCL NHFS

C Carbamidomethylation (+57.02)
O Oxidation (M) (+15.99)

Supporting Peptides:

Peptide	Uniq	-10lgP	Mass	Length	ppm	m/z	z	RT	Fraction	Scan	Source File	Area ebraeus	Area judaeus	#Feature	#Feature ebraeus	#Feature judaeus	Start	End	PTM	AScore	Found By
EEYDLNDPDVLAIVDEGQK.L	Y	140.20	2161.0061	19	5.1	1081.5117	2	51.87	1	F1:48359	20210611-08_2021021-OK192A_815_5.3.236.mgf	0	0	0	0	0	1	19			PEAKS DB
K.YTYDPWWK.L	Y	86.54	1157.5182	8	1.7	579.7651	2	41.86	1	F1:39684	20210611-08_2021021-OK192A_815_5.3.236.mgf	0		0	0	0	30	37			PEAKS DB
K.LMHAC(+57.02)SIANK.Y	Y	78.32	1143.5529	10	-0.6	382.1899	3	16.34	1	F1:7558	20210611-08_2021021-OK192A_815_5.3.236.mgf	0		0	0	0	20	29	Carbamidomethylation	C5:Carbamidomethylation:1000.00	PEAKS DB
K.LM(+15.99)HAC(+57.02)SIANK.Y	Y	71.07	1159.5479	10	5.4	387.5238	3	12.10	1	F1:3140	20210611-08_2021021-OK192A_815_5.3.236.mgf	0		0	0	0	20	29	Oxidation (M); Carbamidomethylation	M2:Oxidation (M):1000.00; C5:Carbamidomethylation:1000.00	PEAKS DB
K.LNILAFK.E	Y	61.32	817.5062	7	4.1	409.7605	2	42.63	1	F1:40433	20210611-08_2021021-OK192A_815_5.3.236.mgf	0		0	0	0	38	44			PEAKS DB
**total 5 peptides**																					

OK213_E_040_F

back to list

| Protein Coverage | Supporting Peptides |

Protein Coverage:

1 EEYDLNDPDV LAIVDEGQKL MHACSIANKY TYDPWWKLNI LAFKEMRVYH SMMSDMVTCL NHFS

C Carbamidomethylation (+57.02)
O Oxidation (M) (+15.99)

Supporting Peptides:

Peptide	Uniq	-10lgP	Mass	Length	ppm	m/z	z	RT	Fraction	Scan	Source File	Area ebraeus	Area judaeus	#Feature	#Feature ebraeus	#Feature judaeus	Start	End	PTM	AScore	Found By
EEYDLNDPDVLAIVDEGQK.L	Y	140.20	2161.0061	19	5.1	1081.5117	2	51.87	1	F1:48359	20210611-08_2021021-OK192A_815_5.3.236.mgf	0	0	0	0	0	1	19			PEAKS DB
K.YTYDPWWK.L	Y	86.54	1157.5182	8	1.7	579.7651	2	41.86	1	F1:39684	20210611-08_2021021-OK192A_815_5.3.236.mgf	0		0	0	0	30	37			PEAKS DB
K.LMHAC(+57.02)SIANK.Y	Y	78.32	1143.5529	10	-0.6	382.1899	3	16.34	1	F1:7558	20210611-08_2021021-OK192A_815_5.3.236.mgf	0		0	0	0	20	29	Carbamidomethylation	C5:Carbamidomethylation:1000.00	PEAKS DB
K.LM(+15.99)HAC(+57.02)SIANK.Y	Y	71.07	1159.5479	10	5.4	387.5238	3	12.10	1	F1:3140	20210611-08_2021021-OK192A_815_5.3.236.mgf	0		0	0	0	20	29	Oxidation (M); Carbamidomethylation	M2:Oxidation (M):1000.00; C5:Carbamidomethylation:1000.00	PEAKS DB
K.LNILAFK.E	Y	61.32	817.5062	7	4.1	409.7605	2	42.63	1	F1:40433	20210611-08_2021021-OK192A_815_5.3.236.mgf	0		0	0	0	38	44			PEAKS DB
**total 5 peptides**																					

SRX1323887_E_036_F

back to list

| Protein Coverage | Supporting Peptides |

Protein Coverage:

1 EEYDLNDPDV LAIVDEGQKL MHACSIANKY TYDPWWKLNI LAFKEMRVYH SMMSDMVTCL NHFS

C Carbamidomethylation (+57.02)
O Oxidation (M) (+15.99)

Supporting Peptides:

Peptide	Uniq	-10lgP	Mass	Length	ppm	m/z	z	RT	Fraction	Scan	Source File	Area ebraeus	Area judaeus	#Feature	#Feature ebraeus	#Feature judaeus	Start	End	PTM	AScore	Found By
EEYDLNDPDVLAIVDEGQK.L	Y	140.20	2161.0061	19	5.1	1081.5117	2	51.87	1	F1:48359	20210611-08_2021021-OK192A_815_5.3.236.mgf	0	0	0	0	0	1	19			PEAKS DB
K.YTYDPWWK.L	Y	86.54	1157.5182	8	1.7	579.7651	2	41.86	1	F1:39684	20210611-08_2021021-OK192A_815_5.3.236.mgf	0		0	0	0	30	37			PEAKS DB
K.LMHAC(+57.02)SIANK.Y	Y	78.32	1143.5529	10	-0.6	382.1899	3	16.34	1	F1:7558	20210611-08_2021021-OK192A_815_5.3.236.mgf	0		0	0	0	20	29	Carbamidomethylation	C5:Carbamidomethylation:1000.00	PEAKS DB
K.LM(+15.99)HAC(+57.02)SIANK.Y	Y	71.07	1159.5479	10	5.4	387.5238	3	12.10	1	F1:3140	20210611-08_2021021-OK192A_815_5.3.236.mgf	0		0	0	0	20	29	Oxidation (M); Carbamidomethylation	M2:Oxidation (M):1000.00; C5:Carbamidomethylation:1000.00	PEAKS DB
K.LNILAFK.E	Y	61.32	817.5062	7	4.1	409.7605	2	42.63	1	F1:40433	20210611-08_2021021-OK192A_815_5.3.236.mgf	0		0	0	0	38	44			PEAKS DB
**total 5 peptides**																					

CebrOK014_E_011_F

back to list

| Protein Coverage | Supporting Peptides |

Protein Coverage:

1 EEYDLNDPDV LAIVDEGQKL MHACSIANKY TYDPWWKLNI LAFKEMRVYH SMMSDMVTCL NHFS

C Carbamidomethylation (+57.02)
O Oxidation (M) (+15.99)

Supporting Peptides:

Peptide	Uniq	-10lgP	Mass	Length	ppm	m/z	z	RT	Fraction	Scan	Source File	Area ebraeus	Area judaeus	#Feature	#Feature ebraeus	#Feature judaeus	Start	End	PTM	AScore	Found By
**total 5 peptides**																					

Peptide	Uniq	-10lgP	Mass	Length	ppm	m/z	z	RT	Fraction	Scan	Source File	Area ebraeus	Area judaeus	#Feature	#Feature ebraeus	#Feature judaeus	Start	End	PTM	AScore	Found By
EEYDLNDPDVLAIVDEGQK.L	Y	140.20	2161.0061	19	5.1	1081.5117	2	51.87	1	F1:48359	20210611-08_2021021-OK192A_815_5.3.236.mgf	0	0	0	0	0	1	19			PEAKS DB
K.YTYDPWWK.L	Y	86.54	1157.5182	8	1.7	579.7651	2	41.86	1	F1:39684	20210611-08_2021021-OK192A_815_5.3.236.mgf	0		0	0	0	30	37			PEAKS DB
K.LMHAC(+57.02)SIANK.Y	Y	78.32	1143.5529	10	-0.6	382.1899	3	16.34	1	F1:7558	20210611-08_2021021-OK192A_815_5.3.236.mgf	0		0	0	0	20	29	Carbamidomethylation	C5:Carbamidomethylation:1000.00	PEAKS DB
K.LM(+15.99)HAC(+57.02)SIANK.Y	Y	71.07	1159.5479	10	5.4	387.5238	3	12.10	1	F1:3140	20210611-08_2021021-OK192A_815_5.3.236.mgf	0		0	0	0	20	29	Oxidation (M); Carbamidomethylation	M2:Oxidation (M):1000.00; C5:Carbamidomethylation:1000.00	PEAKS DB
K.LNILAFK.E	Y	61.32	817.5062	7	4.1	409.7605	2	42.63	1	F1:40433	20210611-08_2021021-OK192A_815_5.3.236.mgf	0		0	0	0	38	44			PEAKS DB
**total 5 peptides**																					

OK214_J_037_F

back to list

| Protein Coverage | Supporting Peptides |

Protein Coverage:

21 O, 24 C

1 **EEYDLNDPDV LAIVDEGQKL MHACSIANKY TYDPWWKLNI LAFK**EMRVYH SMMSDMVTCL NHFS

C Carbamidomethylation (+57.02)
O Oxidation (M) (+15.99)

Supporting Peptides:

Peptide	Uniq	-10lgP	Mass	Length	ppm	m/z	z	RT	Fraction	Scan	Source File	Area ebraeus	Area judaeus	#Feature	#Feature ebraeus	#Feature judaeus	Start	End	PTM	AScore	Found By
EEYDLNDPDVLAIVDEGQK.L	Y	140.20	2161.0061	19	5.1	1081.5117	2	51.87	1	F1:48359	20210611-08_2021021-OK192A_815_5.3.236.mgf	0	0	0	0	0	1	19			PEAKS DB
K.YTYDPWWK.L	Y	86.54	1157.5182	8	1.7	579.7651	2	41.86	1	F1:39684	20210611-08_2021021-OK192A_815_5.3.236.mgf	0		0	0	0	30	37			PEAKS DB
K.LMHAC(+57.02)SIANK.Y	Y	78.32	1143.5529	10	-0.6	382.1899	3	16.34	1	F1:7558	20210611-08_2021021-OK192A_815_5.3.236.mgf	0		0	0	0	20	29	Carbamidomethylation	C5:Carbamidomethylation:1000.00	PEAKS DB
K.LM(+15.99)HAC(+57.02)SIANK.Y	Y	71.07	1159.5479	10	5.4	387.5238	3	12.10	1	F1:3140	20210611-08_2021021-OK192A_815_5.3.236.mgf	0		0	0	0	20	29	Oxidation (M); Carbamidomethylation	M2:Oxidation (M):1000.00; C5:Carbamidomethylation:1000.00	PEAKS DB
K.LNILAFK.E	Y	61.32	817.5062	7	4.1	409.7605	2	42.63	1	F1:40433	20210611-08_2021021-OK192A_815_5.3.236.mgf	0		0	0	0	38	44			PEAKS DB
**total 5 peptides**																					

OK005_J_033_F

back to list

| Protein Coverage | Supporting Peptides |

Protein Coverage:

21 O, 24 C

1 **EEYDLNDPDV LAIVDEGQKL MHACSIANKY TYDPWWKLNI LAFK**EMRVYH SMMSDMVTCL NHFS

C Carbamidomethylation (+57.02)
O Oxidation (M) (+15.99)

Supporting Peptides:

Peptide	Uniq	-10lgP	Mass	Length	ppm	m/z	z	RT	Fraction	Scan	Source File	Area ebraeus	Area judaeus	#Feature	#Feature ebraeus	#Feature judaeus	Start	End	PTM	AScore	Found By
EEYDLNDPDVLAIVDEGQK.L	Y	140.20	2161.0061	19	5.1	1081.5117	2	51.87	1	F1:48359	20210611-08_2021021-OK192A_815_5.3.236.mgf	0	0	0	0	0	1	19			PEAKS DB
K.YTYDPWWK.L	Y	86.54	1157.5182	8	1.7	579.7651	2	41.86	1	F1:39684	20210611-08_2021021-OK192A_815_5.3.236.mgf	0		0	0	0	30	37			PEAKS DB
K.LMHAC(+57.02)SIANK.Y	Y	78.32	1143.5529	10	-0.6	382.1899	3	16.34	1	F1:7558	20210611-08_2021021-OK192A_815_5.3.236.mgf	0		0	0	0	20	29	Carbamidomethylation	C5:Carbamidomethylation:1000.00	PEAKS DB
K.LM(+15.99)HAC(+57.02)SIANK.Y	Y	71.07	1159.5479	10	5.4	387.5238	3	12.10	1	F1:3140	20210611-08_2021021-OK192A_815_5.3.236.mgf	0		0	0	0	20	29	Oxidation (M); Carbamidomethylation	M2:Oxidation (M):1000.00; C5:Carbamidomethylation:1000.00	PEAKS DB
K.LNILAFK.E	Y	61.32	817.5062	7	4.1	409.7605	2	42.63	1	F1:40433	20210611-08_2021021-OK192A_815_5.3.236.mgf	0		0	0	0	38	44			PEAKS DB
**total 5 peptides**																					

OK193_E_033_F

back to list

| Protein Coverage | Supporting Peptides |

Protein Coverage:

21 O, 24 C

1 **EEYDLNDPDV LAIVDEGQKL MHACSIANKY TYDPWWKLNI LAFK**EMRVYH SMMSDMVTCL NHFF

C Carbamidomethylation (+57.02)
O Oxidation (M) (+15.99)

Supporting Peptides:

Peptide	Uniq	-10lgP	Mass	Length	ppm	m/z	z	RT	Fraction	Scan	Source File	Area ebraeus	Area judaeus	#Feature	#Feature ebraeus	#Feature judaeus	Start	End	PTM	AScore	Found By
EEYDLNDPDVLAIVDEGQK.L	Y	140.20	2161.0061	19	5.1	1081.5117	2	51.87	1	F1:48359	20210611-08_2021021-OK192A_815_5.3.236.mgf	0	0	0	0	0	1	19			PEAKS DB
K.YTYDPWWK.L	Y	86.54	1157.5182	8	1.7	579.7651	2	41.86	1	F1:39684	20210611-08_2021021-OK192A_815_5.3.236.mgf	0		0	0	0	30	37			PEAKS DB
K.LMHAC(+57.02)SIANK.Y	Y	78.32	1143.5529	10	-0.6	382.1899	3	16.34	1	F1:7558	20210611-08_2021021-OK192A_815_5.3.236.mgf	0		0	0	0	20	29	Carbamidomethylation	C5:Carbamidomethylation:1000.00	PEAKS DB
K.LM(+15.99)HAC(+57.02)SIANK.Y	Y	71.07	1159.5479	10	5.4	387.5238	3	12.10	1	F1:3140	20210611-08_2021021-OK192A_815_5.3.236.mgf	0		0	0	0	20	29	Oxidation (M); Carbamidomethylation	M2:Oxidation (M):1000.00; C5:Carbamidomethylation:1000.00	PEAKS DB
K.LNILAFK.E	Y	61.32	817.5062	7	4.1	409.7605	2	42.63	1	F1:40433	20210611-08_2021021-OK192A_815_5.3.236.mgf	0		0	0	0	38	44			PEAKS DB
**total 5 peptides**																					

CebrOK016_E_013_F

back to list

| Protein Coverage | Supporting Peptides |

Protein Coverage:

21 O 24 C

1 EEYDLNDPDV LAIVDEGQKL MHACSIANKY TYDPWWKLNI LAFKEMRVYH SMMSDMVTCL NHFF

C Carbamidomethylation (+57.02)
O Oxidation (M) (+15.99)

Supporting Peptides:

Peptide	Uniq	-10lgP	Mass	Length	ppm	m/z	z	RT	Fraction	Scan	Source File	Area ebraeus	Area judaeus	#Feature	#Feature ebraeus	#Feature judaeus	Start	End	PTM	AScore	Found By
EEYDLNDPDVLAIVDEGQK.L	Y	140.20	2161.0061	19	5.1	1081.5117	2	51.87	1	F1:48359	20210611-08_2021021-OK192A_815_5.3.236.mgf	0	0	0	0	0	1	19			PEAKS DB
K.YTYDPWWK.L	Y	86.54	1157.5182	8	1.7	579.7651	2	41.86	1	F1:39684	20210611-08_2021021-OK192A_815_5.3.236.mgf	0		0	0	0	30	37			PEAKS DB
K.LMHAC(+57.02)SIANK.Y	Y	78.32	1143.5529	10	-0.6	382.1899	3	16.34	1	F1:7558	20210611-08_2021021-OK192A_815_5.3.236.mgf	0		0	0	0	20	29	Carbamidomethylation	C5:Carbamidomethylation:1000.00	PEAKS DB
K.LM(+15.99)HAC(+57.02)SIANK.Y	Y	71.07	1159.5479	10	5.4	387.5238	3	12.10	1	F1:3140	20210611-08_2021021-OK192A_815_5.3.236.mgf	0		0	0	0	20	29	Oxidation (M); Carbamidomethylation	M2:Oxidation (M):1000.00; C5:Carbamidomethylation:1000.00	PEAKS DB
K.LNILAFK.E	Y	61.32	817.5062	7	4.1	409.7605	2	42.63	1	F1:40433	20210611-08_2021021-OK192A_815_5.3.236.mgf	0		0	0	0	38	44			PEAKS DB

total 5 peptides

OK213_E_041_F

back to list

| Protein Coverage | Supporting Peptides |

Protein Coverage:

21 O 24 C

1 EEYDLNDPDV LAIVDEGQKL MHACSIANKY TYDPWWKLNI LAFKEMRVYH SMMSDMVTCL NHFF

C Carbamidomethylation (+57.02)
O Oxidation (M) (+15.99)

Supporting Peptides:

Peptide	Uniq	-10lgP	Mass	Length	ppm	m/z	z	RT	Fraction	Scan	Source File	Area ebraeus	Area judaeus	#Feature	#Feature ebraeus	#Feature judaeus	Start	End	PTM	AScore	Found By
EEYDLNDPDVLAIVDEGQK.L	Y	140.20	2161.0061	19	5.1	1081.5117	2	51.87	1	F1:48359	20210611-08_2021021-OK192A_815_5.3.236.mgf	0	0	0	0	0	1	19			PEAKS DB
K.YTYDPWWK.L	Y	86.54	1157.5182	8	1.7	579.7651	2	41.86	1	F1:39684	20210611-08_2021021-OK192A_815_5.3.236.mgf	0		0	0	0	30	37			PEAKS DB
K.LMHAC(+57.02)SIANK.Y	Y	78.32	1143.5529	10	-0.6	382.1899	3	16.34	1	F1:7558	20210611-08_2021021-OK192A_815_5.3.236.mgf	0		0	0	0	20	29	Carbamidomethylation	C5:Carbamidomethylation:1000.00	PEAKS DB
K.LM(+15.99)HAC(+57.02)SIANK.Y	Y	71.07	1159.5479	10	5.4	387.5238	3	12.10	1	F1:3140	20210611-08_2021021-OK192A_815_5.3.236.mgf	0		0	0	0	20	29	Oxidation (M); Carbamidomethylation	M2:Oxidation (M):1000.00; C5:Carbamidomethylation:1000.00	PEAKS DB
K.LNILAFK.E	Y	61.32	817.5062	7	4.1	409.7605	2	42.63	1	F1:40433	20210611-08_2021021-OK192A_815_5.3.236.mgf	0		0	0	0	38	44			PEAKS DB

total 5 peptides

CebrOK014_E_010_F

back to list

| Protein Coverage | Supporting Peptides |

Protein Coverage:

21 O 24 C

1 EEYDLNDPDV LAIVDEGQKL MHACSIANKY TYDPWWKLNI LAFKEMRVYH SMMSDMVTCL NHFF

C Carbamidomethylation (+57.02)
O Oxidation (M) (+15.99)

Supporting Peptides:

Peptide	Uniq	-10lgP	Mass	Length	ppm	m/z	z	RT	Fraction	Scan	Source File	Area ebraeus	Area judaeus	#Feature	#Feature ebraeus	#Feature judaeus	Start	End	PTM	AScore	Found By
EEYDLNDPDVLAIVDEGQK.L	Y	140.20	2161.0061	19	5.1	1081.5117	2	51.87	1	F1:48359	20210611-08_2021021-OK192A_815_5.3.236.mgf	0	0	0	0	0	1	19			PEAKS DB
K.YTYDPWWK.L	Y	86.54	1157.5182	8	1.7	579.7651	2	41.86	1	F1:39684	20210611-08_2021021-OK192A_815_5.3.236.mgf	0		0	0	0	30	37			PEAKS DB
K.LMHAC(+57.02)SIANK.Y	Y	78.32	1143.5529	10	-0.6	382.1899	3	16.34	1	F1:7558	20210611-08_2021021-OK192A_815_5.3.236.mgf	0		0	0	0	20	29	Carbamidomethylation	C5:Carbamidomethylation:1000.00	PEAKS DB
K.LM(+15.99)HAC(+57.02)SIANK.Y	Y	71.07	1159.5479	10	5.4	387.5238	3	12.10	1	F1:3140	20210611-08_2021021-OK192A_815_5.3.236.mgf	0		0	0	0	20	29	Oxidation (M); Carbamidomethylation	M2:Oxidation (M):1000.00; C5:Carbamidomethylation:1000.00	PEAKS DB
K.LNILAFK.E	Y	61.32	817.5062	7	4.1	409.7605	2	42.63	1	F1:40433	20210611-08_2021021-OK192A_815_5.3.236.mgf	0		0	0	0	38	44			PEAKS DB

total 5 peptides

SRX1323887_E_035_F

back to list

| Protein Coverage | Supporting Peptides |

Protein Coverage:

21 O 24 C

1 EEYDLNDPDV LAIVDEGQKL MHACSIANKY TYDPWWKLNI LAFKEMRVYH SMMSDMVTCL NHFF

C Carbamidomethylation (+57.02)
O Oxidation (M) (+15.99)

Supporting Peptides:

Peptide	Uniq	-10lgP	Mass	Length	ppm	m/z	z	RT	Fraction	Scan	Source File	Area ebraeus	Area judaeus	#Feature	#Feature ebraeus	#Feature judaeus	Start	End	PTM	AScore	Found By
EEYDLNDPDVLAIVDEGQK.L	Y	140.20	2161.0061	19	5.1	1081.5117	2	51.87	1	F1:48359	20210611-08_2021021-OK192A_815_5.3.236.mgf	0	0	0	0	0	1	19			PEAKS DB
K.YTYDPWWK.L	Y	86.54	1157.5182	8	1.7	579.7651	2	41.86	1	F1:39684	20210611-08_2021021-OK192A_815_5.3.236.mgf	0		0	0	0	30	37			PEAKS DB
K.LMHAC(+57.02)SIANK.Y	Y	78.32	1143.5529	10	-0.6	382.1899	3	16.34	1	F1:7558	20210611-08_2021021-OK192A_815_5.3.236.mgf	0		0	0	0	20	29	Carbamidomethylation	C5:Carbamidomethylation:1000.00	PEAKS DB
K.LM(+15.99)HAC(+57.02)SIANK.Y	Y	71.07	1159.5479	10	5.4	387.5238	3	12.10	1	F1:3140	20210611-08_2021021-OK192A_815_5.3.236.mgf	0		0	0	0	20	29	Oxidation (M); Carbamidomethylation	M2:Oxidation (M):1000.00; C5:Carbamidomethylation:1000.00	PEAKS DB

total 5 peptides

Peptide	Uniq	-10lgP	Mass	Length	ppm	m/z	z	RT	Fraction	Scan	Source File	Area ebraeus	Area judaeus	#Feature	#Feature ebraeus	#Feature judaeus	Start	End	PTM	AScore	Found By
K.LNILAFK.E	Y	61.32	817.5062	7	4.1	409.7605	2	42.63	1	F1:40433	20210611-08_2021021-OK192A_815_5.3.236.mgf	0		0	0	0	38	44			PEAKS DB
total 5 peptides																					

gene:107300483

back to list

| Protein Coverage | Supporting Peptides |

Protein Coverage:

1 MSSMNPEYDY LFK**LLLIGDS GVGK**SCLLLR **FADDTYTESY ISTIGVDFK**I RTIELDGKTI K**LQIWDTAGQ ER**FRTITSSY

81 YRGAHGIIVV YDVTDQESFN NVK**QWLQEID R**YASENVNKL LVGNKCDLTT K**KVVDYTTAK** EFADSLGIPF LETSAKNATN

161 VEQSFMTMAA EIKKRMGPGA TSGGAEKSNV RIQSTPVKQS SGGCC

Supporting Peptides:

Peptide	Uniq	-10lgP	Mass	Length	ppm	m/z	z	RT	Fraction	Scan	Source File	Area ebraeus	Area judaeus	#Feature	#Feature ebraeus	#Feature judaeus	Start	End	PTM	AScore	Found By
R.FADDTYTESYISTIGVDFK.I	Y	123.83	2170.9946	19	5.6	1086.5061	2	53.75	2	F2:124908	20210611-09_2021021-OK160A_816_5.3.236.mgf		0	0	0	0	31	49			PEAKS DB
K.LQIWDTAGQER.F	Y	87.15	1315.6521	11	3.6	658.8332	2	34.94	1	F1:31834	20210611-08_2021021-OK192A_815_5.3.236.mgf	0	0	0	0	0	62	72			PEAKS DB
K.KVVDYTTAK.E	Y	79.72	1023.5601	9	2.7	512.7866	2	14.04	2	F2:57561	20210611-09_2021021-OK160A_816_5.3.236.mgf		0	0	0	0	132	140			PEAKS DB
K.LLLIGDSGVGK.S	Y	69.42	1070.6335	11	2.2	536.3230	2	39.02	2	F2:99655	20210611-09_2021021-OK160A_816_5.3.236.mgf	0	0	0	0	0	14	24			PEAKS DB
K.QWLQEIDR.Y	Y	63.37	1086.5458	8	1.6	544.2787	2	35.15	2	F2:92812	20210611-09_2021021-OK160A_816_5.3.236.mgf		0	0	0	0	104	111			PEAKS DB
total 5 peptides																					

OK214_J_343_CAP

back to list

| Protein Coverage | Supporting Peptides |

Protein Coverage:

1 MPALAPSFAV LLLSLAVVTG HSVSGMEGHG QSLGLSTVER R**AAGAGFSDS EKAVLVEVHN TLR**NGVKPPA TNMKPMTWSK

81 ELEDIAKAFV AKCHFGHNSQ R**SSQSETFDY VGENVYMSSG K**VANPKDVVQ VWYNEVHNYN YNTNSCLAGK MCGHYTQVVW

161 ADSHALGCAR QSCPGGTSLV VCNYGPGGNW R**GERPYQTQE**

Supporting Peptides:

Peptide	Uniq	-10lgP	Mass	Length	ppm	m/z	z	RT	Fraction	Scan	Source File	Area ebraeus	Area judaeus	#Feature	#Feature ebraeus	#Feature judaeus	Start	End	PTM	AScore	Found By
R.SSQSETFDYVGENVYMSSGK.V	Y	129.39	2213.9424	20	9.0	1107.9838	2	40.63	2	F2:102340	20210611-09_2021021-OK160A_816_5.3.236.mgf		0	0	0	0	102	121			PEAKS DB
R.AAGAGFSDSEK.A	Y	94.35	1038.4618	11	5.8	520.2390	2	14.56	2	F2:58531	20210611-09_2021021-OK160A_816_5.3.236.mgf	0	0	0	0	0	42	52			PEAKS DB
K.AVLVEVHNTLR.N	Y	92.95	1249.7142	11	1.0	625.8624	2	28.19	2	F2:80198	20210611-09_2021021-OK160A_816_5.3.236.mgf		0	0	0	0	53	63			PEAKS DB
R.SSQSETFDYVGENVYM(+15.99)SSGK.V	Y	90.88	2229.9373	20	3.7	1115.9753	2	37.15	2	F2:96338	20210611-09_2021021-OK160A_816_5.3.236.mgf		0	0	0	0	102	121		M16:Oxidation (M):1000.00	PEAKS DB
R.GERPYQTQE	Y	65.43	1106.4993	9	0.2	554.2547	2	13.28	2	F2:56610	20210611-09_2021021-OK160A_816_5.3.236.mgf		0	0	0	0	192	200			PEAKS DB
total 5 peptides																					

CebrOK033_J_271_CAP

back to list

| Protein Coverage | Supporting Peptides |

Protein Coverage:

1 MPALAPSFAV LLLSLAVVTG HSVSGMEGHG QSLGLSTVER R**AAGAGFSDS EKAVLVEVHN TLR**NGVKPPA TNMKPMTWSK

81 ELEDIAKAFV AKCHFGHNSQ R**SSQSETFDY VGENVYMSSG K**VANPKDVVQ VWYNEVHNYN YNTNSCLAGK MCGHYTQVVW

161 ADSHALGCAR QSCPGGTSLV VCNYGPGGNW K**GERPYQTQE**

Supporting Peptides:

Peptide	Uniq	-10lgP	Mass	Length	ppm	m/z	z	RT	Fraction	Scan	Source File	Area ebraeus	Area judaeus	#Feature	#Feature ebraeus	#Feature judaeus	Start	End	PTM	AScore	Found By
R.SSQSETFDYVGENVYMSSGK.V	Y	129.39	2213.9424	20	9.0	1107.9838	2	40.63	2	F2:102340	20210611-09_2021021-OK160A_816_5.3.236.mgf		0	0	0	0	102	121			PEAKS DB
R.AAGAGFSDSEK.A	Y	94.35	1038.4618	11	5.8	520.2390	2	14.56	2	F2:58531	20210611-09_2021021-OK160A_816_5.3.236.mgf	0	0	0	0	0	42	52			PEAKS DB
K.AVLVEVHNTLR.N	Y	92.95	1249.7142	11	1.0	625.8624	2	28.19	2	F2:80198	20210611-09_2021021-OK160A_816_5.3.236.mgf		0	0	0	0	53	63			PEAKS DB
R.SSQSETFDYVGENVYM(+15.99)SSGK.V	Y	90.88	2229.9373	20	3.7	1115.9753	2	37.15	2	F2:96338	20210611-09_2021021-OK160A_816_5.3.236.mgf		0	0	0	0	102	121		M16:Oxidation (M):1000.00	PEAKS DB
K.GERPYQTQE	Y	65.43	1106.4993	9	0.2	554.2547	2	13.28	2	F2:56610	20210611-09_2021021-OK160A_816_5.3.236.mgf		0	0	0	0	192	200			PEAKS DB
total 5 peptides																					

K_0010_139_Ferritin

back to list

| Protein Coverage | Supporting Peptides |

Protein Coverage:

1 **FDRDDVALPG FAK**YFKEMSD EEREHAEKLM KYQNKRGGRV VLQDIKKPDR **DEWGTGLDAM QVALGLEK**SV NQALLELHKV

81 ATSHQDAQMT DFLEGEYLQE QVESIKK**IGD HITNLK**RVGS GLGEYMFDKN LS

Supporting Peptides:

Peptide	Uniq	-10lgP	Mass	Length	ppm	m/z	z	RT	Fraction	Scan	Source File	Area ebraeus	Area judaeus	#Feature	#Feature ebraeus	#Feature judaeus	Start	End	PTM	AScore	Found By
R.DEWGTGLDAMQVALGLEK.S	N	116.51	1931.9298	18	1.8	966.9699	2	59.74	2	F2:131980	20210611-09_2021021-OK160A_816_5.3.236.mgf		0	0	0	0	51	68			PEAKS DB
R.DDVALPGFAK.Y	N	90.93	1031.5287	10	2.5	516.7708	2	38.01	2	F2:97999	20210611-09_2021021-OK160A_816_5.3.236.mgf	0	0	0	0	0	4	13			PEAKS DB
K.IGDHITNLK.R	N	89.25	1009.5556	9	5.8	505.7859	2	19.85	2	F2:65725	20210611-09_2021021-OK160A_816_5.3.236.mgf		0	0	0	0	108	116			PEAKS DB
FDRDDVALPGFAK.Y	Y	67.23	1449.7251	13	0.8	484.2473	3	38.48	2	F2:98912	20210611-09_2021021-OK160A_816_5.3.236.mgf		0	0	0	0	1	13			PEAKS DB
total 4 peptides																					

OK212_J_216_Cerm03

back to list

| Protein Coverage | Supporting Peptides |

Protein Coverage:

1 EISR**RTIDGG EAGVLAGDR**A SNK**NALLQQR** LCP

Supporting Peptides:

Peptide	Uniq	-10lgP	Mass	Length	ppm	m/z	z	RT	Fraction	Scan	Source File	Area ebraeus	Area judaeus	#Feature	#Feature ebraeus	#Feature judaeus	Start	End	PTM	AScore	Found By
R.TIDGGEAGVLAGDR.A	N	131.45	1329.6525	14	2.4	665.8323	2	28.62	2	F2:81183	20210611-09_2021021-OK160A_816_5.3.236.mgf	0	0	0	0	0	6	19			PEAKS DB
R.TIDGGEAGVLAGDR(-.98).A	N	105.16	1328.6685	14	2.0	665.3400	2	27.71	2	F2:79347	20210611-09_2021021-OK160A_816_5.3.236.mgf		0	0	0	0	6	19		R14:Amidation:1000.00	PEAKS DB
R.RTIDGGEAGVLAGDR.A	Y	104.64	1485.7535	15	3.6	496.2581	3	24.73	2	F2:74075	20210611-09_2021021-OK160A_816_5.3.236.mgf		0	0	0	0	5	19			PEAKS DB
K.NALLQQR.L	N	64.80	841.4770	7	4.5	421.7459	2	18.33	2	F2:63419	20210611-09_2021021-OK160A_816_5.3.236.mgf	0	0	0	0	0	24	30			PEAKS DB
**total 4 peptides**																					

gene:107288075

back to list

| Protein Coverage | Supporting Peptides |

Protein Coverage:

1 MPEPAKSAPA AKKGSKKAIT KTQKKGDKKR RKTRKESYSI YVYKVLK**QVH PDTGISSKAM SIMNSFVNDI FER**IAAEASR

81 LAHYNKRSTI TSREIQTAVR **LLLPGELAKH AVSEGTK**AVT KYTSSK

O Oxidation (M) (+15.99)

Supporting Peptides:

Peptide	Uniq	-10lgP	Mass	Length	ppm	m/z	z	RT	Fraction	Scan	Source File	Area ebraeus	Area judaeus	#Feature	#Feature ebraeus	#Feature judaeus	Start	End	PTM	AScore	Found By
K.AM(+15.99)SIM(+15.99)NSFVNDIFER.I	Y	106.77	1804.8124	15	2.4	903.4119	2	50.39	2	F2:119379	20210611-09_2021021-OK160A_816_5.3.236.mgf		0	0	0	0	59	73	Oxidation (M)	M2:Oxidation (M):1000.00;M5:Oxidation (M):1000.00	PEAKS DB
K.QVHPDTGISSK.A	Y	106.55	1167.5884	11	2.8	584.8007	2	13.28	2	F2:56502	20210611-09_2021021-OK160A_816_5.3.236.mgf	0	0	0	0	0	48	58			PEAKS DB
K.AM(+15.99)SIMNSFVNDIFER.I	Y	102.03	1788.8175	15	2.1	895.4142	2	58.07	2	F2:130463	20210611-09_2021021-OK160A_816_5.3.236.mgf		0	0	0	0	59	73		M2:Oxidation (M):26.52	PEAKS DB
K.Q(-17.03)VHPDTGISSK.A	Y	99.83	1150.5619	11	3.5	576.2878	2	19.84	2	F2:65995	20210611-09_2021021-OK160A_816_5.3.236.mgf	0	0	0	0	0	48	58		Q1:Pyro-glu from Q:1000.00	PEAKS DB
K.HAVSEGTK.A	Y	86.65	827.4137	8	5.1	414.7145	2	5.27	2	F2:52256	20210611-09_2021021-OK160A_816_5.3.236.mgf		0	0	0	0	110	117			PEAKS DB
R.LLLPGELAK.H	Y	59.89	952.5957	9	-0.1	477.3031	2	39.13	2	F2:99951	20210611-09_2021021-OK160A_816_5.3.236.mgf	0	0	0	0	0	101	109			PEAKS DB
**total 6 peptides**																					

gene:107298957

back to list

| Protein Coverage | Supporting Peptides |

Protein Coverage:

1 MPEPAKSAPA PKKGSKKAVT KTQKKGDKKR KKSRKESYSI YVYKVLK**QVH PDTGISSKAM SIMNSFVNDI FER**IAGEASR

81 LAHYNKRSTI TSREIQTAVR **LLLPGELAKH AVSEGTK**AVT KYTSSK

O Oxidation (M) (+15.99)

Supporting Peptides:

Peptide	Uniq	-10lgP	Mass	Length	ppm	m/z	z	RT	Fraction	Scan	Source File	Area ebraeus	Area judaeus	#Feature	#Feature ebraeus	#Feature judaeus	Start	End	PTM	AScore	Found By
K.AM(+15.99)SIM(+15.99)NSFVNDIFER.I	Y	106.77	1804.8124	15	2.4	903.4119	2	50.39	2	F2:119379	20210611-09_2021021-OK160A_816_5.3.236.mgf		0	0	0	0	59	73	Oxidation (M)	M2:Oxidation (M):1000.00;M5:Oxidation (M):1000.00	PEAKS DB
K.QVHPDTGISSK.A	Y	106.55	1167.5884	11	2.8	584.8007	2	13.28	2	F2:56502	20210611-09_2021021-OK160A_816_5.3.236.mgf	0	0	0	0	0	48	58			PEAKS DB
K.AM(+15.99)SIMNSFVNDIFER.I	Y	102.03	1788.8175	15	2.1	895.4142	2	58.07	2	F2:130463	20210611-09_2021021-OK160A_816_5.3.236.mgf		0	0	0	0	59	73		M2:Oxidation (M):26.52	PEAKS DB
K.Q(-17.03)VHPDTGISSK.A	Y	99.83	1150.5619	11	3.5	576.2878	2	19.84	2	F2:65995	20210611-09_2021021-OK160A_816_5.3.236.mgf	0	0	0	0	0	48	58		Q1:Pyro-glu from Q:1000.00	PEAKS DB
K.HAVSEGTK.A	Y	86.65	827.4137	8	5.1	414.7145	2	5.27	2	F2:52256	20210611-09_2021021-OK160A_816_5.3.236.mgf		0	0	0	0	110	117			PEAKS DB
R.LLLPGELAK.H	Y	59.89	952.5957	9	-0.1	477.3031	2	39.13	2	F2:99951	20210611-09_2021021-OK160A_816_5.3.236.mgf	0	0	0	0	0	101	109			PEAKS DB
**total 6 peptides**																					

gene:107297087

back to list

| Protein Coverage | Supporting Peptides |

Protein Coverage:

1 MPEPAKSAPV PKKGSKKAIT KTQKKGDKKR RKTRKESYSI YVYKVLK**QVH PDTGISSKAM SIMNSFVNDI FER**IAAEASR

81 LAHYNKRSTI TSREIQTAVR **LLLPGELAKH AVSEGTK**AVT KYTSSK

O Oxidation (M) (+15.99)

Supporting Peptides:

Peptide	Uniq	-10lgP	Mass	Length	ppm	m/z	z	RT	Fraction	Scan	Source File	Area ebraeus	Area judaeus	#Feature	#Feature ebraeus	#Feature judaeus	Start	End	PTM	AScore	Found By
K.AM(+15.99)SIM(+15.99)NSFVNDIFER.I	Y	106.77	1804.8124	15	2.4	903.4119	2	50.39	2	F2:119379	20210611-09_2021021-OK160A_816_5.3.236.mgf		0	0	0	0	59	73	Oxidation (M)	M2:Oxidation (M):1000.00;M5:Oxidation (M):1000.00	PEAKS DB
K.QVHPDTGISSK.A	Y	106.55	1167.5884	11	2.8	584.8007	2	13.28	2	F2:56502	20210611-09_2021021-OK160A_816_5.3.236.mgf	0	0	0	0	0	48	58			PEAKS DB
K.AM(+15.99)SIMNSFVNDIFER.I	Y	102.03	1788.8175	15	2.1	895.4142	2	58.07	2	F2:130463	20210611-09_2021021-OK160A_816_5.3.236.mgf		0	0	0	0	59	73		M2:Oxidation (M):26.52	PEAKS DB
K.Q(-17.03)VHPDTGISSK.A	Y	99.83	1150.5619	11	3.5	576.2878	2	19.84	2	F2:65995	20210611-09_2021021-OK160A_816_5.3.236.mgf	0	0	0	0	0	48	58		Q1:Pyro-glu from Q:1000.00	PEAKS DB
K.HAVSEGTK.A	Y	86.65	827.4137	8	5.1	414.7145	2	5.27	2	F2:52256	20210611-09_2021021-OK160A_816_5.3.236.mgf		0	0	0	0	110	117			PEAKS DB
R.LLLPGELAK.H	Y	59.89	952.5957	9	-0.1	477.3031	2	39.13	2	F2:99951	20210611-09_2021021-OK160A_816_5.3.236.mgf	0	0	0	0	0	101	109			PEAKS DB
**total 6 peptides**																					

CebrOK016_E_140_Tpra

back to list

| Protein Coverage | Supporting Peptides |

Protein Coverage:

1 **QVCPTMTDSY AR**ENECHYDH NLCGKEVSGS CSSLCNCK**NS QECSTDSDHT ITVVKRYVNN QPER**KRYYTC VALSSLNECS

81 GTQEALYVLT PEANELKSVK VLCRCPSTKV YLLARNPQRY VCTTA

C Carbamidomethylation (+57.02)
O Oxidation (M) (+15.99)

Supporting Peptides:

Peptide	Uniq	-10lgP	Mass	Length	ppm	m/z	z	RT	Fraction	Scan	Source File	Area ebraeus	Area judaeus	#Feature	#Feature ebraeus	#Feature judaeus	Start	End	PTM	AScore

Peptide	Uniq	-10lgP	Mass	Length	ppm	m/z	z	RT	Fraction	Scan	Source File	Area ebraeus	Area judaeus	#Feature	#Feature ebraeus	#Feature judaeus	Start	End	PTM	AScore
K.NSQEC(+57.02)STDSDHTITVVK.R	Y	103.57	1919.8530	17	2.4	960.9324	2	20.77	1	F1:12236	20210611-08_2021021-OK192A_815_5.3.236.mgf	0		0	0	0	39	55	Carbamidomethylation	C5:Carbamidomethylation:1000.00
Q(-17.03)VC(+57.02)PTMTDSYAR.E	Y	98.74	1410.5908	12	1.7	706.3012	2	33.96	1	F1:30498	20210611-08_2021021-OK192A_815_5.3.236.mgf	0		0	0	0	1	12	Carbamidomethylation	Q1:Pyro-glu from Q:1000.00;C3:Carbamidomethylation:1000.00
K.RYVNNQPER.K	Y	90.62	1174.5842	9	1.9	588.2983	2	10.46	1	F1:1648	20210611-08_2021021-OK192A_815_5.3.236.mgf	0		0	0	0	56	64		
R.YVNNQPER.K	Y	81.98	1018.4832	8	6.8	510.2504	2	11.28	1	F1:2421	20210611-08_2021021-OK192A_815_5.3.236.mgf	0		0	0	0	57	64		
Q(-17.03)VC(+57.02)PTM(+15.99)TDSYAR.E	Y	77.47	1426.5857	12	2.8	714.2994	2	27.52	1	F1:21864	20210611-08_2021021-OK192A_815_5.3.236.mgf	0		0	0	0	1	12	Carbamidomethylation; Oxidation (M)	Q1:Pyro-glu from Q:1000.00;C3:Carbamidomethylation:1000.00;M6:Oxidation (M):1000.00
**total 5 peptides**																				

SRX1323887_E_218_Tpra06

back to list

| Protein Coverage | Supporting Peptides |

Protein Coverage:

3 C, 6 O, 43 C

1 **QVCPTMTDSY AR**ENECHYDH NLCGKEVSGS CSSLCNCK**NS QECSTDSDHT ITVVKRYVNN QPER**KRYYTC VALSSLNECS

81 GTQEALYVLT PEANELKSVK VLCRCPSTKV YLLARNPQRY VCTTAAQLST RTT

C Carbamidomethylation (+57.02)
O Oxidation (M) (+15.99)

Supporting Peptides:

Peptide	Uniq	-10lgP	Mass	Length	ppm	m/z	z	RT	Fraction	Scan	Source File	Area ebraeus	Area judaeus	#Feature	#Feature ebraeus	#Feature judaeus	Start	End	PTM	AScore
K.NSQEC(+57.02)STDSDHTITVVK.R	Y	103.57	1919.8530	17	2.4	960.9324	2	20.77	1	F1:12236	20210611-08_2021021-OK192A_815_5.3.236.mgf	0		0	0	0	39	55	Carbamidomethylation	C5:Carbamidomethylation:1000.00
Q(-17.03)VC(+57.02)PTMTDSYAR.E	Y	98.74	1410.5908	12	1.7	706.3012	2	33.96	1	F1:30498	20210611-08_2021021-OK192A_815_5.3.236.mgf	0		0	0	0	1	12	Carbamidomethylation	Q1:Pyro-glu from Q:1000.00;C3:Carbamidomethylation:1000.00
K.RYVNNQPER.K	Y	90.62	1174.5842	9	1.9	588.2983	2	10.46	1	F1:1648	20210611-08_2021021-OK192A_815_5.3.236.mgf	0		0	0	0	56	64		
R.YVNNQPER.K	Y	81.98	1018.4832	8	6.8	510.2504	2	11.28	1	F1:2421	20210611-08_2021021-OK192A_815_5.3.236.mgf	0		0	0	0	57	64		
Q(-17.03)VC(+57.02)PTM(+15.99)TDSYAR.E	Y	77.47	1426.5857	12	2.8	714.2994	2	27.52	1	F1:21864	20210611-08_2021021-OK192A_815_5.3.236.mgf	0		0	0	0	1	12	Carbamidomethylation; Oxidation (M)	Q1:Pyro-glu from Q:1000.00;C3:Carbamidomethylation:1000.00;M6:Oxidation (M):1000.00
**total 5 peptides**																				

CebrOK014_E_131_Tpra06

back to list

| Protein Coverage | Supporting Peptides |

Protein Coverage:

3 C, 6 O, 43 C

1 **QVCPTMTDSY AR**ENECHYDH NLCGKEVSGS CSSLCNCK**NS QECSTDSDHT ITVVKRYVNN QPER**KRYYTC VALSSLNECS

81 GTQEALYVLT PEANELKSVK VLCRCPSTKV YLLARNPQRY VCTTAAQLST RTT

C Carbamidomethylation (+57.02)
O Oxidation (M) (+15.99)

Supporting Peptides:

Peptide	Uniq	-10lgP	Mass	Length	ppm	m/z	z	RT	Fraction	Scan	Source File	Area ebraeus	Area judaeus	#Feature	#Feature ebraeus	#Feature judaeus	Start	End	PTM	AScore
K.NSQEC(+57.02)STDSDHTITVVK.R	Y	103.57	1919.8530	17	2.4	960.9324	2	20.77	1	F1:12236	20210611-08_2021021-OK192A_815_5.3.236.mgf	0		0	0	0	39	55	Carbamidomethylation	C5:Carbamidomethylation:1000.00
Q(-17.03)VC(+57.02)PTMTDSYAR.E	Y	98.74	1410.5908	12	1.7	706.3012	2	33.96	1	F1:30498	20210611-08_2021021-OK192A_815_5.3.236.mgf	0		0	0	0	1	12	Carbamidomethylation	Q1:Pyro-glu from Q:1000.00;C3:Carbamidomethylation:1000.00
K.RYVNNQPER.K	Y	90.62	1174.5842	9	1.9	588.2983	2	10.46	1	F1:1648	20210611-08_2021021-OK192A_815_5.3.236.mgf	0		0	0	0	56	64		
R.YVNNQPER.K	Y	81.98	1018.4832	8	6.8	510.2504	2	11.28	1	F1:2421	20210611-08_2021021-OK192A_815_5.3.236.mgf	0		0	0	0	57	64		
Q(-17.03)VC(+57.02)PTM(+15.99)TDSYAR.E	Y	77.47	1426.5857	12	2.8	714.2994	2	27.52	1	F1:21864	20210611-08_2021021-OK192A_815_5.3.236.mgf	0		0	0	0	1	12	Carbamidomethylation; Oxidation (M)	Q1:Pyro-glu from Q:1000.00;C3:Carbamidomethylation:1000.00;M6:Oxidation (M):1000.00
**total 5 peptides**																				

OK213_E_279_Tpra06

back to list

| Protein Coverage | Supporting Peptides |

Protein Coverage:

1 **QVCPTMTDSY AR**ENECHYDH NLCGKEVSGS CSSLCNCK**NS QECSTDSDHT ITVVKRYVNN QPER**KRYYTC VALSSLNECS

81 GTQEALYVLT PEANELKSVK VLCRCPSTKV YLLARNPQRY VCTTAAQLST RTT

C Carbamidomethylation (+57.02)
O Oxidation (M) (+15.99)

Supporting Peptides:

Peptide	Uniq	-10lgP	Mass	Length	ppm	m/z	z	RT	Fraction	Scan	Source File	Area ebraeus	Area judaeus	#Feature	#Feature ebraeus	#Feature judaeus	Start	End	PTM	AScore
K.NSQEC(+57.02)STDSDHTITVVK.R	Y	103.57	1919.8530	17	2.4	960.9324	2	20.77	1	F1:12236	20210611-08_2021021-OK192A_815_5.3.236.mgf	0		0	0	0	39	55	Carbamidomethylation	C5:Carbamidomethylation:1000.00
Q(-17.03)VC(+57.02)PTMTDSYAR.E	Y	98.74	1410.5908	12	1.7	706.3012	2	33.96	1	F1:30498	20210611-08_2021021-OK192A_815_5.3.236.mgf	0		0	0	0	1	12	Carbamidomethylation	Q1:Pyro-glu from Q:1000.00;C3:Carbamidomethylation:1000.00
K.RYVNNQPER.K	Y	90.62	1174.5842	9	1.9	588.2983	2	10.46	1	F1:1648	20210611-08_2021021-OK192A_815_5.3.236.mgf	0		0	0	0	56	64		
R.YVNNQPER.K	Y	81.98	1018.4832	8	6.8	510.2504	2	11.28	1	F1:2421	20210611-08_2021021-OK192A_815_5.3.236.mgf	0		0	0	0	57	64		
Q(-17.03)VC(+57.02)PTM(+15.99)TDSYAR.E	Y	77.47	1426.5857	12	2.8	714.2994	2	27.52	1	F1:21864	20210611-08_2021021-OK192A_815_5.3.236.mgf	0		0	0	0	1	12	Carbamidomethylation; Oxidation (M)	Q1:Pyro-glu from Q:1000.00;C3:Carbamidomethylation:1000.00;M6:Oxidation (M):1000.00
**total 5 peptides**																				

OK006_E_261_Tpra06

[back to list]

| **Protein Coverage** | **Supporting Peptides** |

Protein Coverage:

1 **QVCPTMTDSY AR**ENECHYDH NLCGKEVSGS CSSLCNCK**NS QECSTDSDHT ITVVKRYVNN QPER**KRYYTC VALSSLNECS

81 GTQEALYVLT PEANELKSVK VLCRCPSTKV YLLARNPQRY VCTTAAPLST RTT

C Carbamidomethylation (+57.02)
O Oxidation (M) (+15.99)

Supporting Peptides:

Peptide	Uniq	-10lgP	Mass	Length	ppm	m/z	z	RT	Fraction	Scan	Source File	Area ebraeus	Area judaeus	#Feature	#Feature ebraeus	#Feature judaeus	Start	End	PTM	AScore
K.NSQEC(+57.02)STDSDHTITVVK.R	Y	103.57	1919.8530	17	2.4	960.9324	2	20.77	1	F1:12236	20210611-08_2021021-OK192A_815_5.3.236.mgf	0		0	0	0	39	55	Carbamidomethylation	C5:Carbamidomethylation:1000.00
Q(-17.03)VC(+57.02)PTMTDSYAR.E	Y	98.74	1410.5908	12	1.7	706.3012	2	33.96	1	F1:30498	20210611-08_2021021-OK192A_815_5.3.236.mgf	0		0	0	0	1	12	Carbamidomethylation	Q1:Pyro-glu from Q:1000.00;C3:Carbamidomethylation:1000.00
K.RYVNNQPER.K	Y	90.62	1174.5842	9	1.9	588.2983	2	10.46	1	F1:1648	20210611-08_2021021-OK192A_815_5.3.236.mgf	0		0	0	0	56	64		
R.YVNNQPER.K	Y	81.98	1018.4832	8	6.8	510.2504	2	11.28	1	F1:2421	20210611-08_2021021-OK192A_815_5.3.236.mgf	0		0	0	0	57	64		
Q(-17.03)VC(+57.02)PTM(+15.99)TDSYAR.E	Y	77.47	1426.5857	12	2.8	714.2994	2	27.52	1	F1:21864	20210611-08_2021021-OK192A_815_5.3.236.mgf	0		0	0	0	1	12	Carbamidomethylation; Oxidation (M)	Q1:Pyro-glu from Q:1000.00;C3:Carbamidomethylation:1000.00;M6:Oxidation (M):1000.00
**total 5 peptides**																				

OK006_E_262_Tpra06

[back to list]

| **Protein Coverage** | **Supporting Peptides** |

Protein Coverage:

1 **QVCPTMTDSY AR**ENECHYDH NLCGKEVSGS SSSLCNCK**NS QECSTDSDHT ITVVKRYVNN QPER**KRYYTC VALSSLNECT

81 GTQEALYVLT PEANELKSVK VLCRCPSTKV YLLARNPQRY VCTTAAPLST RTT

C Carbamidomethylation (+57.02)
O Oxidation (M) (+15.99)

Supporting Peptides:

Peptide	Uniq	-10lgP	Mass	Length	ppm	m/z	z	RT	Fraction	Scan	Source File	Area ebraeus	Area judaeus	#Feature	#Feature ebraeus	#Feature judaeus	Start	End	PTM	AScore
K.NSQEC(+57.02)STDSDHTITVVK.R	Y	103.57	1919.8530	17	2.4	960.9324	2	20.77	1	F1:12236	20210611-08_2021021-OK192A_815_5.3.236.mgf	0		0	0	0	39	55	Carbamidomethylation	C5:Carbamidomethylation:1000.00
Q(-17.03)VC(+57.02)PTMTDSYAR.E	Y	98.74	1410.5908	12	1.7	706.3012	2	33.96	1	F1:30498	20210611-08_2021021-OK192A_815_5.3.236.mgf	0		0	0	0	1	12	Carbamidomethylation	Q1:Pyro-glu from Q:1000.00;C3:Carbamidomethylation:1000.00
K.RYVNNQPER.K	Y	90.62	1174.5842	9	1.9	588.2983	2	10.46	1	F1:1648	20210611-08_2021021-OK192A_815_5.3.236.mgf	0		0	0	0	56	64		
R.YVNNQPER.K	Y	81.98	1018.4832	8	6.8	510.2504	2	11.28	1	F1:2421	20210611-08_2021021-OK192A_815_5.3.236.mgf	0		0	0	0	57	64		
Q(-17.03)VC(+57.02)PTM(+15.99)TDSYAR.E	Y	77.47	1426.5857	12	2.8	714.2994	2	27.52	1	F1:21864	20210611-08_2021021-OK192A_815_5.3.236.mgf	0		0	0	0	1	12	Carbamidomethylation; Oxidation (M)	Q1:Pyro-glu from Q:1000.00;C3:Carbamidomethylation:1000.00;M6:Oxidation (M):1000.00
**total 5 peptides**																				

V_1278_106_Prohormone-4a

[back to list]

| **Protein Coverage** | **Supporting Peptides** |

Protein Coverage:

Oxidation (M) (+15.99)

1 MPFNSFHPPM HPLSYLHPPM GYPFVYPAMN TMHPHNLMNP MYNPAHPMHP MNLYNPMNPV TYPIHPTNLY NPMNPINGMS
81 FPYPFHPPMR VASYFLTPPL GTSYLLPSNG YGHFHPVPTQ AASKLLQPSL VTAAPLSLHM HFPYSRQAGV YSPYYLPPYH
161 PAAALQTVYP PYGYAAGVPG AMGWNQFGGA AWNPYSAMGY GAYPMWPSQF AHMWPLHPYY QTVVPAQEGM CSYDRPFKCW
241 GSSTCITIEA VCDKTFDCNN HADEDPRICI AGRRPSYTTL VSYLALPDQK WMLEK**LFNNV DPYMAAQALK LATSLGDLR**W

352

321 SLALTDENIK NTK**TVLEAVK NDDKEPLVSL GMQASAWEAV K**FLMNSLLST DFFLD

Supporting Peptides:

Peptide	Uniq	-10lgP	Mass	Length	ppm	m/z	z	RT	Fraction	Scan	Source File	Area ebraeus	Area judaeus	#Feature	#Feature ebraeus	#Feature judaeus	Start	End	PTM	AScore	Found By
K.NDDKEPLVSLGM(+15.99)QASAWEAVK.F	N	119.58	2303.1104	21	2.7	768.7099	3	46.66	1	F1:44929	20210611-08_2021021-OK192A_815_5.3.236.mgf	0		0	0	0	341	361	Oxidation (M)	M12:Oxidation (M): 1000.00	PEAKS DB
K.LFNNVDPYM(+15.99)AAQALK.L	Y	82.82	1709.8446	15	1.1	855.9273	2	44.66	1	F1:42711	20210611-08_2021021-OK192A_815_5.3.236.mgf	0		0	0	0	296	310		M9:Oxidation (M):1000.00	PEAKS DB
K.LATSLGDLR.W	N	77.79	944.5291	9	3.7	473.2715	2	31.22	2	F2:85544	20210611-09_2021021-OK160A_816_5.3.236.mgf	0	0	0	0	0	311	319			PEAKS DB
K.LFNNVDPYMAAQALK.L	Y	63.83	1693.8497	15	1.8	847.9305	2	48.32	1	F1:46204	20210611-08_2021021-OK192A_815_5.3.236.mgf	0		0	0	0	296	310			PEAKS DB
K.TVLEAVK.N	N	63.45	758.4538	7	2.3	380.2336	2	21.88	1	F1:13726	20210611-08_2021021-OK192A_815_5.3.236.mgf	0		0	0	0	334	340			PEAKS DB
**total 5 peptides**																					

V_CG13_205_Prohormone-4a

back to list

| **Protein Coverage** | **Supporting Peptides** |

Protein Coverage:

Oxidation (M) (+15.99)

1 MPFNSFHPPM HPLSYLHPPM GYPFVYPAMN TMNPHNLMNP MYNPAHPMHP MNLYNPMNPV TYPIHPTNLY NPMNPINGMS
81 FPYPFHPPMR VASYFLTPPL GTSYLLPSNG YGHFHPVPTQ AASKLLQPSL VTAAPLSLHM HFPYSHQAGV YSPYYLPPYH
161 PAAALQTVYP PYGYAAGVPG AMGWNQFGGA AWNPYSAMGY GAYPMWPSQF AHMWPLHPYY QTVVPAQEGM CSYDRPFKCW
241 GSSTCITIEA ICDKTFDCNN HADEDPRICI AGRRPSYTTL VSYLALPDQK WMLEK**LFNNV DPYMAAQALK LATSLGDLR**W

352

321 SLALTDENIK NTK**TVLEAVK NDDKEPLVSL GMQASAWEAV K**FLMNNLLST DFFLD

Supporting Peptides:

Peptide	Uniq	-10lgP	Mass	Length	ppm	m/z	z	RT	Fraction	Scan	Source File	Area ebraeus	Area judaeus	#Feature	#Feature ebraeus	#Feature judaeus	Start	End	PTM	AScore	Found By
K.NDDKEPLVSLGM(+15.99)QASAWEAVK.F	N	119.58	2303.1104	21	2.7	768.7099	3	46.66	1	F1:44929	20210611-08_2021021-OK192A_815_5.3.236.mgf	0		0	0	0	341	361	Oxidation (M)	M12:Oxidation (M): 1000.00	PEAKS DB
K.LFNNVDPYM(+15.99)AAQALK.L	Y	82.82	1709.8446	15	1.1	855.9273	2	44.66	1	F1:42711	20210611-08_2021021-OK192A_815_5.3.236.mgf	0		0	0	0	296	310		M9:Oxidation (M):1000.00	PEAKS DB
K.LATSLGDLR.W	N	77.79	944.5291	9	3.7	473.2715	2	31.22	2	F2:85544	20210611-09_2021021-OK160A_816_5.3.236.mgf	0	0	0	0	0	311	319			PEAKS DB
K.LFNNVDPYMAAQALK.L	Y	63.83	1693.8497	15	1.8	847.9305	2	48.32	1	F1:46204	20210611-08_2021021-OK192A_815_5.3.236.mgf	0		0	0	0	296	310			PEAKS DB
K.TVLEAVK.N	N	63.45	758.4538	7	2.3	380.2336	2	21.88	1	F1:13726	20210611-08_2021021-OK192A_815_5.3.236.mgf	0		0	0	0	334	340			PEAKS DB
**total 5 peptides**																					

P_094D_091_Angiotensin-converting

back to list

| **Protein Coverage** | **Supporting Peptides** |

Protein Coverage:

1 MKATHGRCLL VMVMGVVAVT MMMAVAEASE GKRQHGRWAW SHGKHRWLEL LELLEEMLSA TDAELENELV GGNEDDTYTT
81 SYYSSPEAAR TWAERVDKEL LYWNHKSSEA NWDFITNVTE DTKNKYIAIV DQLSNWSKPT TAEGRKFNTE HFDPDLKRWF
161 RIITMSSSPK DPNKTREITK LHTTMMTTYD TARVCLTDHH RHHAHRRQWQ QHQHQQQQQQ QQPLKNDNAS GCLSLEPELQ
241 DKMRESRDPE ELRELWEGWR EVSGKPQR**EL YAQYVDLLNQ GAR**ENGFADY SDYWSQEQFP DDTDLDAMTS DLWRQLRPLY
321 LELHAYVRGQ LRHQYGSGIV GTDGTIPAHL LGDMWAQAWS DIFSLVIPYP EVSQNSSGLD QK**LKDQYDVR** GLFELAEKFY
401 VSVGLFNMTE TFWAKSMLEK PVDREVQCHA SASDMYSPGD FRIKMCSSVT MEYLSTIHHE MGHVQYYMAY EHQPTVFRDS
481 ANQGFHEAVG DTTTLSVMSR THLQELGLAS SRRGHDAHK**Q DINDLLK**MAL AKIAFLPFAL VVDRWRWQVF REQTTPETYN
561 RDWWKLRLEL QGIRSPVPR**S EEDFDPAAK**M HVVNNSPYYC YFISYVVQFQ FFKTLCRVSG HQGPLHLCDF GRSVKAGTLL
641 KKFMAVGASQ SWQDALKDFT GSRDIIAQPM LEYFEPLRKW LKEENHRNGE HTGWEGATIN WQN

Supporting Peptides:

Peptide	Uniq	-10lgP	Mass	Length	ppm	m/z	z	RT	Fraction	Scan	Source File	Area ebraeus	Area judaeus	#Feature	#Feature ebraeus	#Feature judaeus	Start	End	PTM	AScore	Found By
R.ELYAQYVDLLNQGAR.E	Y	131.57	1751.8842	15	2.1	876.9475	2	49.71	2	F2:118355	20210611-09_2021021-OK160A_816_5.3.236.mgf	0	0	0	0	0	269	283			PEAKS DB
K.LKDQYDVR.G	Y	68.16	1035.5349	8	2.8	518.7740	2	15.68	2	F2:59680	20210611-09_2021021-OK160A_816_5.3.236.mgf		0	0	0	0	383	390			PEAKS DB
R.SEEDFDPAAK.M	Y	66.24	1107.4719	10	4.9	554.7438	2	21.79	1	F1:13745	20210611-08_2021021-OK192A_815_5.3.236.mgf	0		0	0	0	580	589			PEAKS DB
K.QDINDLLK.M	Y	58.00	957.5131	8	2.9	479.7632	2	34.20	2	F2:90790	20210611-09_2021021-OK160A_816_5.3.236.mgf		0	0	0	0	520	527			PEAKS DB
**total 4 peptides**																					

P_102D_097_Nucleobinding-2-like

back to list

| **Protein Coverage** | **Supporting Peptides** |

Protein Coverage:

1 MGWKAVLYTL AVLAIMLLPA GGKPVADKR**G PPHPDPVEAG SK**EDDDR**GTG LEYDAYLR**QI VEALESDTEF RKKLEEANVS
81 DIKSGAIAMH LEMVNHSIRN RLDEIKRMEV RRLQELALVR MKSMSGVGVN RMDIPTHLDL VNPHSFEMKD LESLITKATK
161 DLEEMDRKRK EEFKTYEMEK EFEYQQK**LKD LPEDQKK**DVE EKHKELQEKH KKHEKINHPG SK**DQFEEVWE K**DDHLEDQNF
241 DPKTFFMMHD LNGDRMWDIQ EVEAVLQREL DKVYDSR**NSP EEDDPNER**FE EMSRMREHVF KEVDTNKDFM ITMEEFLSYT
321 GQTGDNAKFK EDEGWETLDE SPQFTDDEYE EFIRQHHPDL ITPKPEDLKF VPEGQAHQQG MPQQQPGQFN AQAGQGQQGQ
401 PGQGQGQPAP GPGQPGHA

Supporting Peptides:

Peptide	Uniq	-10lgP	Mass	Length	ppm	m/z	z	RT	Fraction	Scan	Source File	Area ebraeus	Area judaeus	#Feature	#Feature ebraeus	#Feature judaeus	Start	End	PTM	AScore	Found By
**total 6 peptides**																					

Peptide	Uniq	-10lgP	Mass	Length	ppm	m/z	z	RT	Fraction	Scan	Source File	Area ebraeus	Area judaeus	#Feature	#Feature ebraeus	#Feature judaeus	Start	End	PTM	AScore	Found By
R.GPPHPDPVEAGSK.E	Y	93.93	1286.6255	13	4.3	429.8827	3	16.13	1	F1:7354	20210611-08_2021021-OK192A_815_5.3.236.mgf	0		0	0	0	30	42			PEAKS DB
R.NSPEEDDPNER.F	Y	84.27	1300.5167	11	2.2	651.2646	2	11.70	1	F1:2875	20210611-08_2021021-OK192A_815_5.3.236.mgf	0	0	0	0	0	278	288			PEAKS DB
R.GTGLEYDAYLR.Q	Y	75.63	1256.6036	11	0.8	629.3072	2	37.58	1	F1:35001	20210611-08_2021021-OK192A_815_5.3.236.mgf	0		0	0	0	48	58			PEAKS DB
K.LKDLPEDQKK.D	Y	70.08	1212.6714	10	-1.4	607.3396	2	12.20	2	F2:55159	20210611-09_2021021-OK160A_816_5.3.236.mgf		0	0	0	0	188	197			PEAKS DB
K.DQFEEVWEK.D	Y	58.11	1208.5349	9	0.0	605.2722	2	36.89	2	F2:96016	20210611-09_2021021-OK160A_816_5.3.236.mgf	0	0	0	0	0	223	231			PEAKS DB
K.LKDLPEDQK.K	Y	54.27	1084.5764	9	0.1	543.2933	2	15.10	2	F2:58998	20210611-09_2021021-OK160A_816_5.3.236.mgf		0	0	0	0	188	196			PEAKS DB
total 6 peptides																					

OK212_J_009_B1

back to list

| Protein Coverage | Supporting Peptides |

Protein Coverage:

1 DGGALTERRS ADATALKPEP VLLQKSAARS TDDNGKDTLT QMKRTLKKRG NTARGYEEDR EVAETVRELD EHGKR

Supporting Peptides:

Peptide	Uniq	-10lgP	Mass	Length	ppm	m/z	z	RT	Fraction	Scan	Source File	Area ebraeus	Area judaeus	#Feature	#Feature ebraeus	#Feature judaeus	Start	End	PTM	AScore	Found By
R.STDDNGKDTLTQMK.R	N	120.13	1552.7039	14	0.7	777.3565	2	18.22	2	F2:63522	20210611-09_2021021-OK160A_816_5.3.236.mgf		0	0	0	0	30	43			PEAKS DB
R.SADATALKPEPVLLQK.S	Y	107.32	1679.9457	16	-3.6	840.9736	2	33.75	2	F2:89867	20210611-09_2021021-OK160A_816_5.3.236.mgf		0	0	0	0	10	25			PEAKS DB
R.STDDNGKDTLTQM(+15.99)K.R	N	91.87	1568.6989	14	-0.9	785.3527	2	12.52	2	F2:55548	20210611-09_2021021-OK160A_816_5.3.236.mgf		0	0	0	0	30	43		M13:Oxidation (M):1000.00	PEAKS DB
DGGALTER.R	N	70.06	817.3929	8	1.7	409.7029	2	14.28	1	F1:5430	20210611-08_2021021-OK192A_815_5.3.236.mgf	0	0	0	0	0	1	8			PEAKS DB
total 4 peptides																					

OK005_J_007_B1

back to list

| Protein Coverage | Supporting Peptides |

Protein Coverage:

1 DGGALTERRS ADATALKPEP VLLQKSAARS TDDNGKDTLT QMKRTLKKRG NTARGYEEDR EVAETVRELD EHGKR

Supporting Peptides:

Peptide	Uniq	-10lgP	Mass	Length	ppm	m/z	z	RT	Fraction	Scan	Source File	Area ebraeus	Area judaeus	#Feature	#Feature ebraeus	#Feature judaeus	Start	End	PTM	AScore	Found By
R.STDDNGKDTLTQMK.R	N	120.13	1552.7039	14	0.7	777.3565	2	18.22	2	F2:63522	20210611-09_2021021-OK160A_816_5.3.236.mgf		0	0	0	0	30	43			PEAKS DB
R.SADATALKPEPVLLQK.S	Y	107.32	1679.9457	16	-3.6	840.9736	2	33.75	2	F2:89867	20210611-09_2021021-OK160A_816_5.3.236.mgf		0	0	0	0	10	25			PEAKS DB
R.STDDNGKDTLTQM(+15.99)K.R	N	91.87	1568.6989	14	-0.9	785.3527	2	12.52	2	F2:55548	20210611-09_2021021-OK160A_816_5.3.236.mgf		0	0	0	0	30	43		M13:Oxidation (M):1000.00	PEAKS DB
DGGALTER.R	N	70.06	817.3929	8	1.7	409.7029	2	14.28	1	F1:5430	20210611-08_2021021-OK192A_815_5.3.236.mgf	0	0	0	0	0	1	8			PEAKS DB
total 4 peptides																					

OK212_J_010_B1

back to list

| Protein Coverage | Supporting Peptides |

Protein Coverage:

1 DGGALTERRS ADATALKPEP VLLQKSAARS TDDNGKDTLT QMKRTLKKRG NTARGYENDR ETAENIRELE VAGKRKRKRS

81 IKLGVPRDTR QF

Supporting Peptides:

Peptide	Uniq	-10lgP	Mass	Length	ppm	m/z	z	RT	Fraction	Scan	Source File	Area ebraeus	Area judaeus	#Feature	#Feature ebraeus	#Feature judaeus	Start	End	PTM	AScore	Found By
R.STDDNGKDTLTQMK.R	N	120.13	1552.7039	14	0.7	777.3565	2	18.22	2	F2:63522	20210611-09_2021021-OK160A_816_5.3.236.mgf		0	0	0	0	30	43			PEAKS DB
R.SADATALKPEPVLLQK.S	Y	107.32	1679.9457	16	-3.6	840.9736	2	33.75	2	F2:89867	20210611-09_2021021-OK160A_816_5.3.236.mgf		0	0	0	0	10	25			PEAKS DB
R.STDDNGKDTLTQM(+15.99)K.R	N	91.87	1568.6989	14	-0.9	785.3527	2	12.52	2	F2:55548	20210611-09_2021021-OK160A_816_5.3.236.mgf		0	0	0	0	30	43		M13:Oxidation (M):1000.00	PEAKS DB
DGGALTER.R	N	70.06	817.3929	8	1.7	409.7029	2	14.28	1	F1:5430	20210611-08_2021021-OK192A_815_5.3.236.mgf	0	0	0	0	0	1	8			PEAKS DB
total 4 peptides																					

OK212_J_008_B1

back to list

| Protein Coverage | Supporting Peptides |

Protein Coverage:

1 DGGALTERRS ADATALKPEP VLLQKSAARS TDDNGKDTLT QMKRTLKKRG NVATDREEIL ETIRELNESG KR

Supporting Peptides:

Peptide	Uniq	-10lgP	Mass	Length	ppm	m/z	z	RT	Fraction	Scan	Source File	Area ebraeus	Area judaeus	#Feature	#Feature ebraeus	#Feature judaeus	Start	End	PTM	AScore	Found By
R.STDDNGKDTLTQMK.R	N	120.13	1552.7039	14	0.7	777.3565	2	18.22	2	F2:63522	20210611-09_2021021-OK160A_816_5.3.236.mgf		0	0	0	0	30	43			PEAKS DB
R.SADATALKPEPVLLQK.S	Y	107.32	1679.9457	16	-3.6	840.9736	2	33.75	2	F2:89867	20210611-09_2021021-OK160A_816_5.3.236.mgf		0	0	0	0	10	25			PEAKS DB
R.STDDNGKDTLTQM(+15.99)K.R	N	91.87	1568.6989	14	-0.9	785.3527	2	12.52	2	F2:55548	20210611-09_2021021-OK160A_816_5.3.236.mgf		0	0	0	0	30	43		M13:Oxidation (M):1000.00	PEAKS DB
DGGALTER.R	N	70.06	817.3929	8	1.7	409.7029	2	14.28	1	F1:5430	20210611-08_2021021-OK192A_815_5.3.236.mgf	0	0	0	0	0	1	8			PEAKS DB
total 4 peptides																					

OK005_J_006_B1

back to list

| Protein Coverage | Supporting Peptides |

Protein Coverage:

1 DGGALTERRS ADATALKPEP VLLQKSAARS TDDNGKDTLT QMKRTLKKRG NVATDREEIL ETIRELNESG KR

Supporting Peptides:

Peptide	Uniq	-10lgP	Mass	Length	ppm	m/z	z	RT	Fraction	Scan	Source File	Area ebraeus	Area judaeus	#Feature	#Feature ebraeus	#Feature judaeus	Start	End	PTM	AScore	Found By
R.STDDNGKDTLTQMK.R	N	120.13	1552.7039	14	0.7	777.3565	2	18.22	2	F2:63522	20210611-09_2021021-OK160A_816_5.3.236.mgf		0	0	0	0	30	43			PEAKS DB
R.SADATALKPEPVLLQK.S	Y	107.32	1679.9457	16	-3.6	840.9736	2	33.75	2	F2:89867	20210611-09_2021021-OK160A_816_5.3.236.mgf		0	0	0	0	10	25			PEAKS DB
R.STDDNGKDTLTQM(+15.99)K.R	N	91.87	1568.6989	14	-0.9	785.3527	2	12.52	2	F2:55548	20210611-09_2021021-OK160A_816_5.3.236.mgf		0	0	0	0	30	43		M13:Oxidation (M):1000.00	PEAKS DB
total 4 peptides																					

Peptide	Uniq	-10lgP	Mass	Length	ppm	m/z	z	RT	Fraction	Scan	Source File	Area ebraeus	Area judaeus	#Feature	#Feature ebraeus	#Feature judaeus	Start	End	PTM	AScore	Found By
DGGALTER.R	N	70.06	817.3929	8	1.7	409.7029	2	14.28	1	F1:5430	20210611-08_2021021-OK192A_815_5.3.236.mgf	0	0	0	0	0	1	8			PEAKS DB
**total 4 peptides**																					

OK212_J_220_Cerm03

back to list

| Protein Coverage | Supporting Peptides |

Protein Coverage:

1 EISHR**TIDGG EAGVLAGDRA TNK**NAELQQG LCPT

Supporting Peptides:

Peptide	Uniq	-10lgP	Mass	Length	ppm	m/z	z	RT	Fraction	Scan	Source File	Area ebraeus	Area judaeus	#Feature	#Feature ebraeus	#Feature judaeus	Start	End	PTM	AScore	Found By
R.TIDGGEAGVLAGDR.A	N	131.45	1329.6525	14	2.4	665.8323	2	28.62	2	F2:81183	20210611-09_2021021-OK160A_816_5.3.236.mgf	0	0	0	0	0	6	19			PEAKS DB
R.TIDGGEAGVLAGDRATNK.N	Y	129.48	1743.8751	18	0.2	872.9413	2	25.18	2	F2:75165	20210611-09_2021021-OK160A_816_5.3.236.mgf		0	0	0	0	6	23			PEAKS DB
R.TIDGGEAGVLAGDR(-.98).A	N	105.16	1328.6685	14	2.0	665.3400	2	27.71	2	F2:79347	20210611-09_2021021-OK160A_816_5.3.236.mgf		0	0	0	0	6	19		R14:Amidation:1000.00	PEAKS DB
**total 3 peptides**																					

OK214_J_005_B1

back to list

| Protein Coverage | Supporting Peptides |

Protein Coverage:

1 **DGGALTER**R**S ADATALKPEP ILLQK**SAAR**S TDDNGKDTLT QMK**RTLKKRG NTARGYEEDR EVAETVRELD EHGKR

Supporting Peptides:

Peptide	Uniq	-10lgP	Mass	Length	ppm	m/z	z	RT	Fraction	Scan	Source File	Area ebraeus	Area judaeus	#Feature	#Feature ebraeus	#Feature judaeus	Start	End	PTM	AScore	Found By
R.STDDNGKDTLTQMK.R	N	120.13	1552.7039	14	0.7	777.3565	2	18.22	2	F2:63522	20210611-09_2021021-OK160A_816_5.3.236.mgf		0	0	0	0	30	43			PEAKS DB
R.SADATALKPEPILLQK.S	Y	104.64	1693.9614	16	-0.9	847.9836	2	36.16	2	F2:94489	20210611-09_2021021-OK160A_816_5.3.236.mgf		0	0	0	0	10	25			PEAKS DB
R.STDDNGKDTLTQM(+15.99)K.R	N	91.87	1568.6989	14	-0.9	785.3527	2	12.52	2	F2:55548	20210611-09_2021021-OK160A_816_5.3.236.mgf		0	0	0	0	30	43		M13:Oxidation (M):1000.00	PEAKS DB
DGGALTER.R	N	70.06	817.3929	8	1.7	409.7029	2	14.28	1	F1:5430	20210611-08_2021021-OK192A_815_5.3.236.mgf	0	0	0	0	0	1	8			PEAKS DB
**total 4 peptides**																					

OK214_J_004_B1

back to list

| Protein Coverage | Supporting Peptides |

Protein Coverage:

1 **DGGALTER**R**S ADATALKPEP ILLQK**SAAR**S TDDNGKDTLT QMK**RTLKKRG NVATDREEIL ETIRELNESG KR

Supporting Peptides:

Peptide	Uniq	-10lgP	Mass	Length	ppm	m/z	z	RT	Fraction	Scan	Source File	Area ebraeus	Area judaeus	#Feature	#Feature ebraeus	#Feature judaeus	Start	End	PTM	AScore	Found By
R.STDDNGKDTLTQMK.R	N	120.13	1552.7039	14	0.7	777.3565	2	18.22	2	F2:63522	20210611-09_2021021-OK160A_816_5.3.236.mgf		0	0	0	0	30	43			PEAKS DB
R.SADATALKPEPILLQK.S	Y	104.64	1693.9614	16	-0.9	847.9836	2	36.16	2	F2:94489	20210611-09_2021021-OK160A_816_5.3.236.mgf		0	0	0	0	10	25			PEAKS DB
R.STDDNGKDTLTQM(+15.99)K.R	N	91.87	1568.6989	14	-0.9	785.3527	2	12.52	2	F2:55548	20210611-09_2021021-OK160A_816_5.3.236.mgf		0	0	0	0	30	43		M13:Oxidation (M):1000.00	PEAKS DB
DGGALTER.R	N	70.06	817.3929	8	1.7	409.7029	2	14.28	1	F1:5430	20210611-08_2021021-OK192A_815_5.3.236.mgf	0	0	0	0	0	1	8			PEAKS DB
**total 4 peptides**																					

V_1278_048_E-1

back to list

| Protein Coverage | Supporting Peptides |

Protein Coverage:

1 WPR**LNDWDCE LGR**NMHITCK **QLEQCGFIEK** KDGQLTCKLR CRCKPGKRCL RK**EKIDWSDI TTR**IYHCPWP

(C at 9 and 25: Carbamidomethylation (+57.02))

Supporting Peptides:

Peptide	Uniq	-10lgP	Mass	Length	ppm	m/z	z	RT	Fraction	Scan	Source File	Area ebraeus	Area judaeus	#Feature	#Feature ebraeus	#Feature judaeus	Start	End	PTM	AScore	Found By
K.EKIDWSDITTR.I	Y	98.62	1362.6780	11	1.4	682.3446	2	34.31	1	F1:30882	20210611-08_2021021-OK192A_815_5.3.236.mgf	0	0	0	0	0	53	63			PEAKS DB
K.QLEQC(+57.02)GFIEK.K	Y	93.89	1250.5964	10	3.3	626.3052	2	27.67	1	F1:22189	20210611-08_2021021-OK192A_815_5.3.236.mgf	0	0	0	0	0	21	30	Carbamidomethylation	C5:Carbamidomethylation:1000.00	PEAKS DB
K.Q(-17.03)LEQC(+57.02)GFIEK.K	Y	86.02	1233.5699	10	-1.3	617.7891	2	39.10	1	F1:36643	20210611-08_2021021-OK192A_815_5.3.236.mgf	0		0	0	0	21	30	Carbamidomethylation	Q1:Pyro-glu from Q:1000.00;C5:Carbamidomethylation:1000.00	PEAKS DB
K.IDWSDITTR.I	Y	79.29	1105.5404	9	0.1	553.7755	2	38.75	1	F1:36423	20210611-08_2021021-OK192A_815_5.3.236.mgf	0	0	0	0	0	55	63			PEAKS DB
R.LNDWDC(+57.02)ELGR.N	Y	75.44	1276.5507	10	1.6	639.2812	2	33.14	1	F1:29308	20210611-08_2021021-OK192A_815_5.3.236.mgf	0		0	0	0	4	13	Carbamidomethylation	C6:Carbamidomethylation:1000.00	PEAKS DB
**total 5 peptides**																					

V_CG13_066_E-1

back to list

| Protein Coverage | Supporting Peptides |

Protein Coverage:

1 WPR**LNDWDCE LGR**NMHITCK **QLEQCGFIEK** KDGQLTCKLR CRCKPGKRCL RK**EKIDWSDI TTR**IYHCPWP

(C at 9 and 25: Carbamidomethylation (+57.02))

Supporting Peptides:

Peptide	Uniq	-10lgP	Mass	Length	ppm	m/z	z	RT	Fraction	Scan	Source File	Area ebraeus	Area judaeus	#Feature	#Feature ebraeus	#Feature judaeus	Start	End	PTM	AScore	Found By
K.EKIDWSDITTR.I	Y	98.62	1362.6780	11	1.4	682.3446	2	34.31	1	F1:30882	20210611-08_2021021-OK192A_815_5.3.236.mgf	0	0	0	0	0	53	63			PEAKS DB
**total 5 peptides**																					

Peptide	Uniq	-10lgP	Mass	Length	ppm	m/z	z	RT	Fraction	Scan	Source File	Area ebraeus	Area judaeus	#Feature	#Feature ebraeus	#Feature judaeus	Start	End	PTM	AScore	Found By
K.QLEQC(+57.02)GFIEK.K	Y	93.89	1250.5964	10	3.3	626.3052	2	27.67	1	F1:22189	20210611-08_2021021-OK192A_815_5.3.236.mgf	0	0	0	0	0	21	30	Carbamidomethylation	C5:Carbamidomethylation:1000.00	PEAKS DB
K.Q(-17.03)LEQC(+57.02)GFIEK.K	Y	86.02	1233.5699	10	-1.3	617.7891	2	39.10	1	F1:36643	20210611-08_2021021-OK192A_815_5.3.236.mgf	0		0	0	0	21	30	Carbamidomethylation	Q1:Pyro-glu from Q:1000.00;C5:Carbamidomethylation:1000.00	PEAKS DB
K.IDWSDITTR.I	Y	79.29	1105.5404	9	0.1	553.7755	2	38.75	1	F1:36423	20210611-08_2021021-OK192A_815_5.3.236.mgf	0	0	0	0	0	55	63			PEAKS DB
R.LNDWDC(+57.02)ELGR.N	Y	75.44	1276.5507	10	1.6	639.2812	2	33.14	1	F1:29308	20210611-08_2021021-OK192A_815_5.3.236.mgf	0		0	0	0	4	13	Carbamidomethylation	C6:Carbamidomethylation:1000.00	PEAKS DB
**total 5 peptides**																					

SRX1323887_E_199_Pmag02

back to list

| **Protein Coverage** | **Supporting Peptides** |

Protein Coverage:

1 QFFCPDSEDD PLNCLETQAT SATCMKSTED ETYSYVCGYC GKKKEACFGK **KVPSSDYNCQ IHKIPNHCGG PVM**

59 C, 68 C, 73 O

C Carbamidomethylation (+57.02)
O Oxidation (M) (+15.99)

Supporting Peptides:

Peptide	Uniq	-10lgP	Mass	Length	ppm	m/z	z	RT	Fraction	Scan	Source File	Area ebraeus	Area judaeus	#Feature	#Feature ebraeus	#Feature judaeus	Start	End	PTM	AScore	Found By
K.VPSSDYNC(+57.02)QIHK.I	Y	108.78	1446.6561	12	-0.3	724.3321	2	17.48	2	F2:62177	20210611-09_2021021-OK160A_816_5.3.236.mgf		0	0	0	0	52	63	Carbamidomethylation	C8:Carbamidomethylation:1000.00	PEAKS DB
K.KVPSSDYNC(+57.02)QIHK.I	Y	101.53	1574.7511	13	2.5	525.9234	3	15.65	2	F2:59847	20210611-09_2021021-OK160A_816_5.3.236.mgf		0	0	0	0	51	63	Carbamidomethylation	C9:Carbamidomethylation:1000.00	PEAKS DB
K.IPNHC(+57.02)GGPVM(+15.99)	N	90.01	1096.4794	10	-0.1	549.2448	2	16.28	1	F1:7548	20210611-08_2021021-OK192A_815_5.3.236.mgf	0		0	0	0	64	73	Carbamidomethylation; Oxidation (M)	C5:Carbamidomethylation:1000.00;M10:Oxidation (M):1000.00	PEAKS DB
K.IPNHC(+57.02)GGPVM	N	70.78	1080.4845	10	2.7	541.2490	2	22.57	1	F1:14539	20210611-08_2021021-OK192A_815_5.3.236.mgf	0		0	0	0	64	73	Carbamidomethylation	C5:Carbamidomethylation:1000.00	PEAKS DB
**total 4 peptides**																					

SRX1323887_E_200_Pmag02

back to list

| **Protein Coverage** | **Supporting Peptides** |

Protein Coverage:

1 QFFCPDSEDD PLNCLETQAT SATCMKSTED ETYSYVCGYC GKKKEACFGK **KVPSSDYNCQ IHKIPNHCGG PVM**

59 C, 68 C, 73 O

C Carbamidomethylation (+57.02)
O Oxidation (M) (+15.99)

Supporting Peptides:

Peptide	Uniq	-10lgP	Mass	Length	ppm	m/z	z	RT	Fraction	Scan	Source File	Area ebraeus	Area judaeus	#Feature	#Feature ebraeus	#Feature judaeus	Start	End	PTM	AScore	Found By
K.VPSSDYNC(+57.02)QIHK.I	Y	108.78	1446.6561	12	-0.3	724.3321	2	17.48	2	F2:62177	20210611-09_2021021-OK160A_816_5.3.236.mgf		0	0	0	0	52	63	Carbamidomethylation	C8:Carbamidomethylation:1000.00	PEAKS DB
K.KVPSSDYNC(+57.02)QIHK.I	Y	101.53	1574.7511	13	2.5	525.9234	3	15.65	2	F2:59847	20210611-09_2021021-OK160A_816_5.3.236.mgf		0	0	0	0	51	63	Carbamidomethylation	C9:Carbamidomethylation:1000.00	PEAKS DB
K.IPNHC(+57.02)GGPVM(+15.99)	N	90.01	1096.4794	10	-0.1	549.2448	2	16.28	1	F1:7548	20210611-08_2021021-OK192A_815_5.3.236.mgf	0		0	0	0	64	73	Carbamidomethylation; Oxidation (M)	C5:Carbamidomethylation:1000.00;M10:Oxidation (M):1000.00	PEAKS DB
K.IPNHC(+57.02)GGPVM	N	70.78	1080.4845	10	2.7	541.2490	2	22.57	1	F1:14539	20210611-08_2021021-OK192A_815_5.3.236.mgf	0		0	0	0	64	73	Carbamidomethylation	C5:Carbamidomethylation:1000.00	PEAKS DB
**total 4 peptides**																					

OK212_J_265_Pmag02

back to list

| **Protein Coverage** | **Supporting Peptides** |

Protein Coverage:

1 QFFCPDSEND PLNCIETKAS DATCMKSNSD QTYSYVCGYC GKKKETCFGK **KVPTSDYNCQ IHKIPNHCGG PVL**

59 C

C Carbamidomethylation (+57.02)

Supporting Peptides:

Peptide	Uniq	-10lgP	Mass	Length	ppm	m/z	z	RT	Fraction	Scan	Source File	Area ebraeus	Area judaeus	#Feature	#Feature ebraeus	#Feature judaeus	Start	End	PTM	AScore	Found By
K.KVPTSDYNC(+57.02)QIHK.I	N	109.55	1588.7667	13	1.8	530.5949	3	16.58	2	F2:60994	20210611-09_2021021-OK160A_816_5.3.236.mgf	0	0	0	0	0	51	63		C9:Carbamidomethylation:1000.00	PEAKS DB
K.VPTSDYNC(+57.02)QIHK.I	N	103.68	1460.6718	12	1.1	487.8963	3	18.39	2	F2:63597	20210611-09_2021021-OK160A_816_5.3.236.mgf	0	0	0	0	0	52	63	Carbamidomethylation	C8:Carbamidomethylation:1000.00	PEAKS DB
K.IPNHC(+57.02)GGPVL	Y	79.63	1062.5281	10	3.7	532.2711	2	26.38	2	F2:77178	20210611-09_2021021-OK160A_816_5.3.236.mgf		0	0	0	0	64	73		C5:Carbamidomethylation:1000.00	PEAKS DB
**total 3 peptides**																					

CebrOK035_J_309_Pmag02

back to list

| **Protein Coverage** | **Supporting Peptides** |

Protein Coverage:

1 QFFCPDSEND PLNCVETKPT AATCMHSRSD KTYSYVCGYC GKKKETCFGK **KVPTSDYNCQ IHKIPNHCGG PVL** 59 C

C Carbamidomethylation (+57.02)

Supporting Peptides:

Peptide	Uniq	-10lgP	Mass	Length	ppm	m/z	z	RT	Fraction	Scan	Source File	Area ebraeus	Area judaeus	#Feature	#Feature ebraeus	#Feature judaeus	Start	End	PTM	AScore	Found By
K.KVPTSDYNC(+57.02)QIHK.I	N	109.55	1588.7667	13	1.8	530.5949	3	16.58	2	F2:60994	20210611-09_2021021-OK160A_816_5.3.236.mgf	0	0	0	0	0	51	63		C9:Carbamidomethylation:1000.00	PEAKS DB
K.VPTSDYNC(+57.02)QIHK.I	N	103.68	1460.6718	12	1.1	487.8963	3	18.39	2	F2:63597	20210611-09_2021021-OK160A_816_5.3.236.mgf	0	0	0	0	0	52	63	Carbamidomethylation	C8:Carbamidomethylation:1000.00	PEAKS DB
K.IPNHC(+57.02)GGPVL	Y	79.63	1062.5281	10	3.7	532.2711	2	26.38	2	F2:77178	20210611-09_2021021-OK160A_816_5.3.236.mgf		0	0	0	0	64	73		C5:Carbamidomethylation:1000.00	PEAKS DB
**total 3 peptides**																					

CebrOK035_J_308_Pmag02

back to list

| Protein Coverage | Supporting Peptides |

Protein Coverage:

1 QFFCPDSEDD PLNCLETQAT AATCMKSTED ETYSYVCGYC GKKKETCFGK **KVPTSDYNCQ IHKIPNHCGG PVL** 59 C

C Carbamidomethylation (+57.02)

Supporting Peptides:

Peptide	Uniq	-10lgP	Mass	Length	ppm	m/z	z	RT	Fraction	Scan	Source File	Area ebraeus	Area judaeus	#Feature	#Feature ebraeus	#Feature judaeus	Start	End	PTM	AScore	Found By
K.KVPTSDYNC(+57.02)QIHK.I	N	109.55	1588.7667	13	1.8	530.5949	3	16.58	2	F2:60994	20210611-09_2021021-OK160A_816_5.3.236.mgf	0	0	0	0	0	51	63		C9:Carbamidomethylation:1000.00	PEAKS DB
K.VPTSDYNC(+57.02)QIHK.I	N	103.68	1460.6718	12	1.1	487.8963	3	18.39	2	F2:63597	20210611-09_2021021-OK160A_816_5.3.236.mgf	0	0	0	0	0	52	63	Carbamidomethylation	C8:Carbamidomethylation:1000.00	PEAKS DB
K.IPNHC(+57.02)GGPVL	Y	79.63	1062.5281	10	3.7	532.2711	2	26.38	2	F2:77178	20210611-09_2021021-OK160A_816_5.3.236.mgf		0	0	0	0	64	73		C5:Carbamidomethylation:1000.00	PEAKS DB
**total 3 peptides**																					

CebrOK033_J_219_Pmag02

back to list

| Protein Coverage | Supporting Peptides |

Protein Coverage:

1 QFFCPDSEDD PLNCLETQAT AATCMKSTED ETYSYVCGYC GKKKETCFGK **KVPTSDYNCQ IHKIPNHCGG PVL** 59 C

C Carbamidomethylation (+57.02)

Supporting Peptides:

Peptide	Uniq	-10lgP	Mass	Length	ppm	m/z	z	RT	Fraction	Scan	Source File	Area ebraeus	Area judaeus	#Feature	#Feature ebraeus	#Feature judaeus	Start	End	PTM	AScore	Found By
K.KVPTSDYNC(+57.02)QIHK.I	N	109.55	1588.7667	13	1.8	530.5949	3	16.58	2	F2:60994	20210611-09_2021021-OK160A_816_5.3.236.mgf	0	0	0	0	0	51	63		C9:Carbamidomethylation:1000.00	PEAKS DB
K.VPTSDYNC(+57.02)QIHK.I	N	103.68	1460.6718	12	1.1	487.8963	3	18.39	2	F2:63597	20210611-09_2021021-OK160A_816_5.3.236.mgf	0	0	0	0	0	52	63	Carbamidomethylation	C8:Carbamidomethylation:1000.00	PEAKS DB
K.IPNHC(+57.02)GGPVL	Y	79.63	1062.5281	10	3.7	532.2711	2	26.38	2	F2:77178	20210611-09_2021021-OK160A_816_5.3.236.mgf		0	0	0	0	64	73		C5:Carbamidomethylation:1000.00	PEAKS DB
**total 3 peptides**																					

OK214_J_271_Pmag02

back to list

| Protein Coverage | Supporting Peptides |

Protein Coverage:

1 QFFCPDSEDD PLNCLETQAT AATCMKSTED ETYSYVCGYC GKKKETCFGK **KVPTSDYNCQ IHKIPNHCGG PVL** 59 C

C Carbamidomethylation (+57.02)

Supporting Peptides:

Peptide	Uniq	-10lgP	Mass	Length	ppm	m/z	z	RT	Fraction	Scan	Source File	Area ebraeus	Area judaeus	#Feature	#Feature ebraeus	#Feature judaeus	Start	End	PTM	AScore	Found By
K.KVPTSDYNC(+57.02)QIHK.I	N	109.55	1588.7667	13	1.8	530.5949	3	16.58	2	F2:60994	20210611-09_2021021-OK160A_816_5.3.236.mgf	0	0	0	0	0	51	63		C9:Carbamidomethylation:1000.00	PEAKS DB
K.VPTSDYNC(+57.02)QIHK.I	N	103.68	1460.6718	12	1.1	487.8963	3	18.39	2	F2:63597	20210611-09_2021021-OK160A_816_5.3.236.mgf	0	0	0	0	0	52	63	Carbamidomethylation	C8:Carbamidomethylation:1000.00	PEAKS DB
K.IPNHC(+57.02)GGPVL	Y	79.63	1062.5281	10	3.7	532.2711	2	26.38	2	F2:77178	20210611-09_2021021-OK160A_816_5.3.236.mgf		0	0	0	0	64	73		C5:Carbamidomethylation:1000.00	PEAKS DB
**total 3 peptides**																					

OK005_J_275_Pmag02

back to list

| Protein Coverage | Supporting Peptides |

Protein Coverage:

1 QFFCPESEND PLNCLETQPN SATCMQSSDD QSYSYVCGYC GKKKETCFGK **KVPTSDYNCQ IHKIPNHCGG PVL** 59 C

C Carbamidomethylation (+57.02)

Supporting Peptides:

Peptide	Uniq	-10lgP	Mass	Length	ppm	m/z	z	RT	Fraction	Scan	Source File	Area ebraeus	Area judaeus	#Feature	#Feature ebraeus	#Feature judaeus	Start	End	PTM	AScore	Found By
**total 3 peptides**																					

Peptide	Uniq	-10lgP	Mass	Length	ppm	m/z	z	RT	Fraction	Scan	Source File	Area ebraeus	Area judaeus	#Feature	#Feature ebraeus	#Feature judaeus	Start	End	PTM	AScore	Found By
K.KVPTSDYNC(+57.02)QIHK.I	N	109.55	1588.7667	13	1.8	530.5949	3	16.58	2	F2:60994	20210611-09_2021021-OK160A_816_5.3.236.mgf	0	0	0	0	0	51	63		C9:Carbamidomethylation:1000.00	PEAKS DB
K.VPTSDYNC(+57.02)QIHK.I	N	103.68	1460.6718	12	1.1	487.8963	3	18.39	2	F2:63597	20210611-09_2021021-OK160A_816_5.3.236.mgf	0	0	0	0	0	52	63	Carbamidomethylation	C8:Carbamidomethylation:1000.00	PEAKS DB
K.IPNHC(+57.02)GGPVL	Y	79.63	1062.5281	10	3.7	532.2711	2	26.38	2	F2:77178	20210611-09_2021021-OK160A_816_5.3.236.mgf		0	0	0	0	64	73		C5:Carbamidomethylation:1000.00	PEAKS DB
total 3 peptides																					

OK212_J_264_Pmag02

back to list

| Protein Coverage | Supporting Peptides |

Protein Coverage:

59 C

1 QFFCPDSEDD PLNCLETQAT AATCMKSTED ETYSYVCGYC GKKKETCFGK **KVPTSDYNCQ IHKIPNHCGG PVL**

C Carbamidomethylation (+57.02)

Supporting Peptides:

Peptide	Uniq	-10lgP	Mass	Length	ppm	m/z	z	RT	Fraction	Scan	Source File	Area ebraeus	Area judaeus	#Feature	#Feature ebraeus	#Feature judaeus	Start	End	PTM	AScore	Found By
K.KVPTSDYNC(+57.02)QIHK.I	N	109.55	1588.7667	13	1.8	530.5949	3	16.58	2	F2:60994	20210611-09_2021021-OK160A_816_5.3.236.mgf	0	0	0	0	0	51	63		C9:Carbamidomethylation:1000.00	PEAKS DB
K.VPTSDYNC(+57.02)QIHK.I	N	103.68	1460.6718	12	1.1	487.8963	3	18.39	2	F2:63597	20210611-09_2021021-OK160A_816_5.3.236.mgf	0	0	0	0	0	52	63	Carbamidomethylation	C8:Carbamidomethylation:1000.00	PEAKS DB
K.IPNHC(+57.02)GGPVL	Y	79.63	1062.5281	10	3.7	532.2711	2	26.38	2	F2:77178	20210611-09_2021021-OK160A_816_5.3.236.mgf		0	0	0	0	64	73		C5:Carbamidomethylation:1000.00	PEAKS DB
total 3 peptides																					

OK214_J_272_Pmag02

back to list

| Protein Coverage | Supporting Peptides |

Protein Coverage:

59 C

1 QFFCPESENE PLNCLETQPN SATCMQSSDD QSYSYVCGYC GKKKETCFGK **KVPTSDYNCQ IHKIPNHCGG PVL**

C Carbamidomethylation (+57.02)

Supporting Peptides:

Peptide	Uniq	-10lgP	Mass	Length	ppm	m/z	z	RT	Fraction	Scan	Source File	Area ebraeus	Area judaeus	#Feature	#Feature ebraeus	#Feature judaeus	Start	End	PTM	AScore	Found By
K.KVPTSDYNC(+57.02)QIHK.I	N	109.55	1588.7667	13	1.8	530.5949	3	16.58	2	F2:60994	20210611-09_2021021-OK160A_816_5.3.236.mgf	0	0	0	0	0	51	63		C9:Carbamidomethylation:1000.00	PEAKS DB
K.VPTSDYNC(+57.02)QIHK.I	N	103.68	1460.6718	12	1.1	487.8963	3	18.39	2	F2:63597	20210611-09_2021021-OK160A_816_5.3.236.mgf	0	0	0	0	0	52	63	Carbamidomethylation	C8:Carbamidomethylation:1000.00	PEAKS DB
K.IPNHC(+57.02)GGPVL	Y	79.63	1062.5281	10	3.7	532.2711	2	26.38	2	F2:77178	20210611-09_2021021-OK160A_816_5.3.236.mgf		0	0	0	0	64	73		C5:Carbamidomethylation:1000.00	PEAKS DB
total 3 peptides																					

gene:107299479

back to list

| Protein Coverage | Supporting Peptides |

Protein Coverage:

1 MQIFVKTLTG K**TITLEVEPS DTIENVK**AKI QDK**EGIPPDQ QR**LIFAGKQL EDGR**TLSDYN IQKESTLHLV LR**LRGGMQIF

81 VKTLTVSDYN IQKESTLHLV LRLRGGMQIF VKTLTGKTIT LEVEPSDTIE NVKAKIQDKE GIPPDQQRLI FAGKQLEDGR

161 TLSDYNIQKE STLHLVLRLR GGF

Supporting Peptides:

Peptide	Uniq	-10lgP	Mass	Length	ppm	m/z	z	RT	Fraction	Scan	Source File	Area ebraeus	Area judaeus	#Feature	#Feature ebraeus	#Feature judaeus	Start	End	PTM	AScore	Found By
K.TITLEVEPSDTIENVK.A	Y	101.48	1786.9200	16	-0.2	894.4637	2	41.99	1	F1:39768	20210611-08_2021021-OK192A_815_5.3.236.mgf	0	0	0	0	0	12	27			PEAKS DB
K.ESTLHLVLR.L	Y	87.22	1066.6135	9	2.6	534.3134	2	31.53	1	F1:27196	20210611-08_2021021-OK192A_815_5.3.236.mgf	0	0	0	0	0	64	72			PEAKS DB
R.TLSDYNIQK.E	Y	74.97	1080.5452	9	1.1	541.2782	2	23.62	2	F2:72217	20210611-09_2021021-OK160A_816_5.3.236.mgf	0	0	0	0	0	55	63			PEAKS DB
K.EGIPPDQQR.L	Y	63.07	1038.5094	9	3.0	520.2614	2	16.35	2	F2:60614	20210611-09_2021021-OK160A_816_5.3.236.mgf	0	0	0	0	0	34	42			PEAKS DB
total 4 peptides																					

SRX1323887_E_190_Ggeo01

back to list

| Protein Coverage | Supporting Peptides |

Protein Coverage:

1 **FQADDSGLDK K**SRPLSRAAR **DHANSDFFGK** LQAAELDRHR R**ILTETPFNS PNANHYHK**MQ YF

Supporting Peptides:

Peptide	Uniq	-10lgP	Mass	Length	ppm	m/z	z	RT	Fraction	Scan	Source File	Area ebraeus	Area judaeus	#Feature	#Feature ebraeus	#Feature judaeus	Start	End	PTM	AScore	Found By
R.DHANSDFFGK.L	N	108.55	1136.4886	10	2.5	569.2509	2	23.01	1	F1:15072	20210611-08_2021021-OK192A_815_5.3.236.mgf	0		0	0	0	21	30			PEAKS DB
R.ILTETPFNSPNANHYHK.M	Y	107.56	1981.9646	17	0.8	661.6602	3	33.30	1	F1:29581	20210611-08_2021021-OK192A_815_5.3.236.mgf	0		0	0	0	42	58			PEAKS DB
FQADDSGLDKK.S	N	65.78	1222.5829	11	-4.3	612.2938	2	17.28	1	F1:8549	20210611-08_2021021-OK192A_815_5.3.236.mgf	0		0	0	0	1	11			PEAKS DB
total 3 peptides																					

CebrOK014_E_119_Ggeo01

back to list

| Protein Coverage | Supporting Peptides |

Protein Coverage:

1 **FQADDSGLDK K**SRPLSRAAR **DHANSDFFGK** LQAAELDRHR R**ILTETPFNS PNANHYHK**MQ HF

Supporting Peptides:

Peptide	Uniq	-10lgP	Mass	Length	ppm	m/z	z	RT	Fraction	Scan	Source File	Area ebraeus	Area judaeus	#Feature	#Feature ebraeus	#Feature judaeus	Start	End	PTM	AScore	Found By
R.DHANSDFFGK.L	N	108.55	1136.4886	10	2.5	569.2509	2	23.01	1	F1:15072	20210611-08_2021021-OK192A_815_5.3.236.mgf	0		0	0	0	21	30			PEAKS DB
R.ILTETPFNSPNANHYHK.M	Y	107.56	1981.9646	17	0.8	661.6602	3	33.30	1	F1:29581	20210611-08_2021021-OK192A_815_5.3.236.mgf	0		0	0	0	42	58			PEAKS DB
FQADDSGLDKK.S	N	65.78	1222.5829	11	-4.3	612.2938	2	17.28	1	F1:8549	20210611-08_2021021-OK192A_815_5.3.236.mgf	0		0	0	0	1	11			PEAKS DB
total 3 peptides																					

OK005_J_264_Ggeo01

back to list

| Protein Coverage | Supporting Peptides |

Protein Coverage:

1 FQADDSGLDK KSRPLSRAAR DHANSDFFGK LQAAELDRHR RILTETPFNS PNANHYHKVQ HF

Supporting Peptides:

Peptide	Uniq	-10lgP	Mass	Length	ppm	m/z	z	RT	Fraction	Scan	Source File	Area ebraeus	Area judaeus	#Feature	#Feature ebraeus	#Feature judaeus	Start	End	PTM	AScore	Found By
R.DHANSDFFGK.L	N	108.55	1136.4886	10	2.5	569.2509	2	23.01	1	F1:15072	20210611-08_2021021-OK192A_815_5.3.236.mgf	0		0	0	0	21	30			PEAKS DB
R.ILTETPFNSPNANHYHK.V	Y	107.56	1981.9646	17	0.8	661.6602	3	33.30	1	F1:29581	20210611-08_2021021-OK192A_815_5.3.236.mgf	0		0	0	0	42	58			PEAKS DB
FQADDSGLDKK.S	N	65.78	1222.5829	11	-4.3	612.2938	2	17.28	1	F1:8549	20210611-08_2021021-OK192A_815_5.3.236.mgf	0		0	0	0	1	11			PEAKS DB
total 3 peptides																					

OK213_E_251_Ggeo01

back to list

| Protein Coverage | Supporting Peptides |

Protein Coverage:

1 FQADDSGLDK KSRPLSRAAR DHANSDFFGK LQAAELDRHR RILTETPFNS PNANHYHKVQ HF

Supporting Peptides:

Peptide	Uniq	-10lgP	Mass	Length	ppm	m/z	z	RT	Fraction	Scan	Source File	Area ebraeus	Area judaeus	#Feature	#Feature ebraeus	#Feature judaeus	Start	End	PTM	AScore	Found By
R.DHANSDFFGK.L	N	108.55	1136.4886	10	2.5	569.2509	2	23.01	1	F1:15072	20210611-08_2021021-OK192A_815_5.3.236.mgf	0		0	0	0	21	30			PEAKS DB
R.ILTETPFNSPNANHYHK.V	Y	107.56	1981.9646	17	0.8	661.6602	3	33.30	1	F1:29581	20210611-08_2021021-OK192A_815_5.3.236.mgf	0		0	0	0	42	58			PEAKS DB
FQADDSGLDKK.S	N	65.78	1222.5829	11	-4.3	612.2938	2	17.28	1	F1:8549	20210611-08_2021021-OK192A_815_5.3.236.mgf	0		0	0	0	1	11			PEAKS DB
total 3 peptides																					

P_022_Coagulation

back to list

| Protein Coverage | Supporting Peptides |

Protein Coverage:

1 MMLLLSVTLT LLVIASTNAD SPPGVPPHQQ QRQQQFDQQQ MNVQEGHGHA GSKLKFQSEI HNADHIIEHL QDVIKTKPKD

O Oxidation (M) (+15.99)

82 94

81 QMSEEELEFH YFKMHDYDNN NKLDGVEIGK ALTHFHDDDH DANKPETGDS SSSSTSPPAD NQKSTQGSSS HSGGNDPDRV

161 SGGEDVEIVT DEKLAELVDS ILELLDWDND GYVEYFELKV AQFKSKDKE

Supporting Peptides:

Peptide	Uniq	-10lgP	Mass	Length	ppm	m/z	z	RT	Fraction	Scan	Source File	Area ebraeus	Area judaeus	#Feature	#Feature ebraeus	#Feature judaeus	Start	End	PTM	AScore	Found By
K.M(+15.99)HDYDNNNKLDGVEIGK.A	Y	112.80	1976.8898	17	1.9	659.9690	3	24.30	2	F2:73307	20210611-09_2021021-OK160A_816_5.3.236.mgf	0	0	0	0	0	94	110	Oxidation (M)	M1:Oxidation (M):1000.00	PEAKS DB
K.DQMSEEELEFHYFK.M	Y	98.80	1830.7770	14	-1.4	916.3910	2	42.39	1	F1:40251	20210611-08_2021021-OK192A_815_5.3.236.mgf	0		0	0	0	80	93			PEAKS DB
K.DQM(+15.99)SEEELEFHYFK.M	Y	83.64	1846.7720	14	-1.7	616.5945	3	39.39	1	F1:37083	20210611-08_2021021-OK192A_815_5.3.236.mgf	0		0	0	0	80	93	Oxidation (M)	M3:Oxidation (M):1000.00	PEAKS DB
K.LDGVEIGK.A	Y	65.43	829.4545	8	0.5	415.7332	2	25.02	1	F1:18355	20210611-08_2021021-OK192A_815_5.3.236.mgf	0	0	0	0	0	103	110			PEAKS DB
total 4 peptides																					

D_059_Immunoglobulin-like

back to list

| Protein Coverage | Supporting Peptides |

Protein Coverage:

1 ETTPGGESEK SDTVSPKVSP RPPAVITPEL VTVVDGDNVT YICNASCTVN DKVSFKWFRN GAEISDAKEQ KYTFVASLKD

C Carbamidomethylation (+57.02)

81 NINKFSCMAE STLGVESGKS DNVSPKVRPQ PPTITPELVK VVDGENVTYT CNTSYTGNDK VSFKWFKNGD GIPDAKEQKY

175

161 TFVSSLKDNK NKFSCMIETT PGGESEKSDN VSPKVRTRPP NVITSETVTV VDGDNVTYIC NTSYICDDKV TFKWFKNGAE

241 LSDVKGQKYT FVSSLKDNKN KFSCLIIITP GGESEESDNV CLEVRPRPPT IIPEIVTVVD GDNNVSPKVR PQPPTVTPEL

321 VTVVDGENVT YTCNTSYTVN DKVSFKWFEN GARIPDAKEQ IYTFVSSLKD NKKFSCMIET A

Supporting Peptides:

Peptide	Uniq	-10lgP	Mass	Length	ppm	m/z	z	RT	Fraction	Scan	Source File	Area ebraeus	Area judaeus	#Feature	#Feature ebraeus	#Feature judaeus	Start	End	PTM	AScore	Found By
K.FSC(+57.02)MIETTPGGESEK.S	Y	113.87	1671.7120	15	-1.0	836.8589	2	30.27	2	F2:83755	20210611-09_2021021-OK160A_816_5.3.236.mgf		0	0	0	0	173	187	Carbamidomethylation	C3:Carbamidomethylation:1000.00	PEAKS DB
R.NGAEISDAKEQK.Y	Y	86.20	1288.6259	12	1.1	645.3182	2	10.99	2	F2:54000	20210611-09_2021021-OK160A_816_5.3.236.mgf		0	0	0	0	60	71			PEAKS DB
K.YTFVSSLK.D	Y	79.17	943.5015	8	4.0	472.7579	2	32.82	2	F2:88168	20210611-09_2021021-OK160A_816_5.3.236.mgf	0	0	0	0	0	160	167			PEAKS DB
total 3 peptides																					

OK214_J_329_PPI

back to list

| Protein Coverage | Supporting Peptides |

Protein Coverage:

1 MLVCGAIVFL LVQGFAFADK AKGPKVTDKV FFDIEIGGEK AGRIVIGLFG KTVPKTAENF KKLAEGYEES GETLTYKGSK

81 FHRVIKEFMI QGGDFTRGDG TGGRSIYGEK FADENFKLQH YGPGWLSMAN AGKDTNGSQF FITTKKTPWL DGRHVVFGKV

161 LEGMKVVRKI EGTRTGDRDK PVKDVVIVDS GSLPVDEPFS VSKDPVSEEN

Supporting Peptides:

Peptide	Uniq	-10lgP	Mass	Length	ppm	m/z	z	RT	Fraction	Scan	Source File	Area ebraeus	Area judaeus	#Feature	#Feature ebraeus	#Feature judaeus	Start	End	PTM	AScore	Found By
K.LAEGYEESGETLTYK.G	Y	106.39	1688.7781	15	-0.1	845.3927	2	30.74	2	F2:84640	20210611-09_2021021-OK160A_816_5.3.236.mgf		0	0	0	0	63	77			PEAKS DB
K.DTNGSQFFITTK.K	N	100.10	1357.6514	12	-0.1	679.8300	2	36.35	2	F2:94879	20210611-09_2021021-OK160A_816_5.3.236.mgf		0	0	0	0	134	145			PEAKS DB
R.IVIGLFGK.T	N	64.69	845.5374	8	-0.9	423.7738	2	44.37	2	F2:109266	20210611-09_2021021-OK160A_816_5.3.236.mgf		0	0	0	0	44	51			PEAKS DB
**total 3 peptides**																					

OK212_J_316_PPI

back to list

| Protein Coverage | Supporting Peptides |

Protein Coverage:

1 MLVCGAIVFL LVQGFAFADK AKGPKVTDKV FFDIEIGGEK AGR**IVIGLFG K**TVPKTAENF KK**LAEGYEES GETLTYK**GSK

81 FHRVIKEFMI QGGDFTRGDG TGGRSIYGEK FADENFKLQH YGPGWLSMAN AGK**DTNGSQF FITTK**KTPWL DGRHVVFGKV

161 LEGMKVVRKI EGTRTGDRDK PVKDVVIVDS GSLPVDEPFS VSKDPVSEEN

Supporting Peptides:

Peptide	Uniq	-10lgP	Mass	Length	ppm	m/z	z	RT	Fraction	Scan	Source File	Area ebraeus	Area judaeus	#Feature	#Feature ebraeus	#Feature judaeus	Start	End	PTM	AScore	Found By
K.LAEGYEESGETLTYK.G	Y	106.39	1688.7781	15	-0.1	845.3927	2	30.74	2	F2:84640	20210611-09_2021021-OK160A_816_5.3.236.mgf		0	0	0	0	63	77			PEAKS DB
K.DTNGSQFFITTK.K	N	100.10	1357.6514	12	-0.1	679.8300	2	36.35	2	F2:94879	20210611-09_2021021-OK160A_816_5.3.236.mgf		0	0	0	0	134	145			PEAKS DB
R.IVIGLFGK.T	N	64.69	845.5374	8	-0.9	423.7738	2	44.37	2	F2:109266	20210611-09_2021021-OK160A_816_5.3.236.mgf		0	0	0	0	44	51			PEAKS DB
**total 3 peptides**																					

SRX1323887_E_191_Ggeo01

back to list

| Protein Coverage | Supporting Peptides |

Protein Coverage:

1 **FQADDSGLDK K**SRPLSRAAR **DHANSDFFGK** LQAAELDRHR R**FLLDAAFYN PHANHLHK**ML LFQTTSQRSG F

Supporting Peptides:

Peptide	Uniq	-10lgP	Mass	Length	ppm	m/z	z	RT	Fraction	Scan	Source File	Area ebraeus	Area judaeus	#Feature	#Feature ebraeus	#Feature judaeus	Start	End	PTM	AScore	Found By
R.DHANSDFFGK.L	N	108.55	1136.4886	10	2.5	569.2509	2	23.01	1	F1:15072	20210611-08_2021021-OK192A_815_5.3.236.mgf	0		0	0	0	21	30			PEAKS DB
R.FLLDAAFYNPHANHLHK.M	Y	88.49	2007.0115	17	-0.6	670.0082	3	46.57	1	F1:44806	20210611-08_2021021-OK192A_815_5.3.236.mgf	0		0	0	0	42	58			PEAKS DB
FQADDSGLDKK.S	N	65.78	1222.5829	11	-4.3	612.2938	2	17.28	1	F1:8549	20210611-08_2021021-OK192A_815_5.3.236.mgf	0		0	0	0	1	11			PEAKS DB
**total 3 peptides**																					

OK193_E_234_Tand01

back to list

| Protein Coverage | Supporting Peptides |

Protein Coverage:

1 TPDVLCITPV GQLSELCTTI GGTCAEYYHW KGKNNCSRFC ECDVGVACPT DEAHAIVRGT VK**RPLTHYTC EPITEFPKCE**

81 **VDDVAIVNSG PGLR**RVVKCI CPWKYMYDGN SSHICKD

Supporting Peptides:

Peptide	Uniq	-10lgP	Mass	Length	ppm	m/z	z	RT	Fraction	Scan	Source File	Area ebraeus	Area judaeus	#Feature	#Feature ebraeus	#Feature judaeus	Start	End	PTM	AScore	Found By
K.C(+57.02)EVDDVAIVNSGPGLR.R	Y	129.32	1699.8199	16	2.6	850.9163	2	37.78	1	F1:35162	20210611-08_2021021-OK192A_815_5.3.236.mgf	0		0	0	0	79	94		C1:Carbamidomethylation:100.00	PEAKS DB
K.RPLTHYTC(+57.02)EPITEFPK.C	Y	86.41	1987.9825	16	-1.1	663.6649	3	31.42	1	F1:27186	20210611-08_2021021-OK192A_815_5.3.236.mgf	0		0	0	0	63	78		C8:Carbamidomethylation:100.00	PEAKS DB
**total 2 peptides**																					

OK213_E_278_Tand01

back to list

| Protein Coverage | Supporting Peptides |

Protein Coverage:

1 TPDVLCITPV GQLSELCTTI GGTCAEYYHW KGKNNCSRFC ECDVGVACPT DEAHAIVRGT VK**RPLTHYTC EPITEFPKCE**

81 **VDDVAIVNSG PGLR**RVVKCI CPWKYMYDGN SSHICKD

Supporting Peptides:

Peptide	Uniq	-10lgP	Mass	Length	ppm	m/z	z	RT	Fraction	Scan	Source File	Area ebraeus	Area judaeus	#Feature	#Feature ebraeus	#Feature judaeus	Start	End	PTM	AScore	Found By
K.C(+57.02)EVDDVAIVNSGPGLR.R	Y	129.32	1699.8199	16	2.6	850.9163	2	37.78	1	F1:35162	20210611-08_2021021-OK192A_815_5.3.236.mgf	0		0	0	0	79	94		C1:Carbamidomethylation:100.00	PEAKS DB
K.RPLTHYTC(+57.02)EPITEFPK.C	Y	86.41	1987.9825	16	-1.1	663.6649	3	31.42	1	F1:27186	20210611-08_2021021-OK192A_815_5.3.236.mgf	0		0	0	0	63	78		C8:Carbamidomethylation:100.00	PEAKS DB
**total 2 peptides**																					

OK006_E_260_Tand01

back to list

| Protein Coverage | Supporting Peptides |

Protein Coverage:

1 TPDVLCITPV GQLSELCTTI GGTCAEYYHW KGKNNCSRFC ECDVGVACPT DEAHAIVRGT VK**RPLTHYTC EPITEFPKCE**

81 **VDDVAIVNSG PGLR**RVVKCI CPWKYMYDGN SSHICKD

Supporting Peptides:

Peptide	Uniq	-10lgP	Mass	Length	ppm	m/z	z	RT	Fraction	Scan	Source File	Area ebraeus	Area judaeus	#Feature	#Feature ebraeus	#Feature judaeus	Start	End	PTM	AScore	Found By
K.C(+57.02)EVDDVAIVNSGPGLR.R	Y	129.32	1699.8199	16	2.6	850.9163	2	37.78	1	F1:35162	20210611-08_2021021-OK192A_815_5.3.236.mgf	0		0	0	0	79	94		C1:Carbamidomethylation:100.00	PEAKS DB
K.RPLTHYTC(+57.02)EPITEFPK.C	Y	86.41	1987.9825	16	-1.1	663.6649	3	31.42	1	F1:27186	20210611-08_2021021-OK192A_815_5.3.236.mgf	0		0	0	0	63	78		C8:Carbamidomethylation:100.00	PEAKS DB
**total 2 peptides**																					

SRX1323887_E_217_Tand01

back to list

| Protein Coverage | Supporting Peptides |

Protein Coverage:

1 TPDVLCITPV GQLSELCTTI GGTCAEYYHW KGKNNCSRFC ECDVGVACPT DEAHAIVRGT VK**RPLTHYTC EPITEFPKCE**

81 **VDDVAIVNSG PGLR**RVVKCI CPWKYMYDGN SSHICKD

Supporting Peptides:

Peptide	Uniq	-10lgP	Mass	Length	ppm	m/z	z	RT	Fraction	Scan	Source File	Area ebraeus	Area judaeus	#Feature	#Feature ebraeus	#Feature judaeus	Start	End	PTM	AScore	Found By
K.C(+57.02)EVDDVAIVNSGPGLR.R	Y	129.32	1699.8199	16	2.6	850.9163	2	37.78	1	F1:35162	20210611-08_2021021-OK192A_815_5.3.236.mgf	0		0	0	0	79	94		C1:Carbamidomethylation:100 0.00	PEAKS DB
K.RPLTHYTC(+57.02)EPITEFPK.C	Y	86.41	1987.9825	16	-1.1	663.6649	3	31.42	1	F1:27186	20210611-08_2021021-OK192A_815_5.3.236.mgf	0		0	0	0	63	78		C8:Carbamidomethylation:100 0.00	PEAKS DB
**total 2 peptides**																					

P_079D_079_Metallopeptidase

back to list

| Protein Coverage | Supporting Peptides |

Protein Coverage:

1 QGLGMQPWMS LGQSAPPSYD MEAMLVKLAY TGITPFFRVL VDADARNNTK QIVWINPGML GISRQYLVLG RTSLEVITYK
81 KWCYDMVVNV LGLQPSRALN RSVSEAVDFE VKLADIYPRS LRASAVASGN HKTFVLSQMD AVYPNFNWAK YLKLFFQMSQ
161 VKLPPDLNVS VHHEFVQNLT AVIAQTDKR**V LANYLLMR**VV RSSYSYLSKE IR**DLDIPMR**T ALFGMNQSLI HWQSCVTFLT

241 KVMMNAVGSM YVTHHFSPQS KQKATEVVEF IRDAMQEIIR QSRWIDNATK AKALEKSEKL QANVGYPENL LNKTYLEAMY
321 R**NVVFDPK**AL YKNVMQVHFL STSGNAKALL SPAPPRQQWI VSAADVNAYY DSTSNVMFCP AAMLQPPYFG DKFPMSMVFG

401 AIGMVISHEL THAFDDQGRK FDEEGNKASW WTEASKLKFD QQAQCFVNQF NEIVYAGHRI NGNATLSENI ADNGGVKIAF
481 RAFKKWQAQK GPEPVQLPNL SFTPDQLFFI SLSQMWCNVI TNQQAK**ASVL TFDHSPNNIR** ANMVMTNSMN FSQAFNCPAG

561 SKMNPADKCQ LW

Supporting Peptides:

Peptide	Uniq	-10lgP	Mass	Length	ppm	m/z	z	RT	Fraction	Scan	Source File	Area ebraeus	Area judaeus	#Feature	#Feature ebraeus	#Feature judaeus	Start	End	PTM	AScore	Found By
K.ASVLTFDHSPNNIR.A	Y	99.19	1569.7899	14	0.7	524.2687	3	31.24	2	F2:85466	20210611-09_2021021-OK160A_816_5.3.236.mgf	0	0	0	0	0	527	540			PEAKS DB
R.VLANYLLMR.V	Y	75.54	1091.6161	9	-2.6	546.8116	2	43.61	2	F2:107761	20210611-09_2021021-OK160A_816_5.3.236.mgf		0	0	0	0	190	198			PEAKS DB
R.NVVFDPK.A	Y	62.29	817.4333	7	1.6	409.7229	2	24.35	2	F2:73455	20210611-09_2021021-OK160A_816_5.3.236.mgf		0	0	0	0	322	328			PEAKS DB
R.DLDIPMR.T	Y	58.90	858.4269	7	2.4	430.2200	2	34.75	2	F2:92035	20210611-09_2021021-OK160A_816_5.3.236.mgf		0	0	0	0	213	219			PEAKS DB
**total 4 peptides**																					

gene:107297064

back to list

| Protein Coverage | Supporting Peptides |

Protein Coverage:

1 M**VNFTVDQIR** AIMDKKANIR **NMSVIAHVDH GK**STLTDSLV CKAGIIASSR AGETRFTDTR KDEQERCITI KSTAISLFYE

81 LSENDLAFIK QSKDGCGFLI NLIDSPGHVD FSSEVTAALR VTDGALVVVD CVSGVCVQTE TVLRQAIAER IKPVLMMNKM
161 DRALLELQLQ PEELYQTFQR IVENVNVIIS TYGEGETGPM GNIMIDPVLG TVGFGSGLHG WAFTLKQFAE MYVAKFAAKG
241 EGQLNCNERA KKVDDMMKKL WGDRYFDPAT GKFSKSATSP EGKKLPRTFC QLILDPIFKV FDAIMNFKKE ETAKLIEKLD
321 IKLDSEDKGK EGKPLLKVRQ NGHPGIKNCD PKGPLMMYIS KMVPTSDKGR FYAFGRVFSG VVSTGLKCRI MGPNYTPGKK
401 EDLYLKPIQR TILMMGRYTE PIEDVPCGNI VGLVGVDQFL VKTGTITTFE HAHNMRVMK**F SVSPVVR**VAV EAKNPADLPK

481 LVEGLKRLAK SDPMVQCIIE ESGEHIIAGA GELHLEICLK DLEEDHACIP IKKSDPVVSY RETVSEESGT LCLSKSPNKH
561 NRLYMKARPF PDGLAEDIDK GDVSSRQELK QRARYLAEKY EWDVTEARKI WCFGPDGTGP NILTDITKGV QYLNEIKDSV
641 VAGFQWATKE GALCEENLRG VRFDVHDVTL HADAIHRGGG QIIPTARRCL YACMLTAQPR LMEPIYLVEI QCPEQVVGGI
721 YGVLNRKRGH VFEESQVAGT PMFVVK**AYLP VNESFGFTAD LR**SNTGGQAF PQCVFDHWQI LPGDPFDNTT RPCQVVIETR

801 KRKGLKEGIP ALDNFLDKL

Supporting Peptides:

Peptide	Uniq	-10lgP	Mass	Length	ppm	m/z	z	RT	Fraction	Scan	Source File	Area ebraeus	Area judaeus	#Feature	#Feature ebraeus	#Feature judaeus	Start	End	PTM	AScore	Found By
K.AYLPVNESFGFTADLR.S	Y	99.31	1798.8889	16	0.0	900.4480	2	52.73	2	F2:123202	20210611-09_2021021-OK160A_816_5.3.236.mgf		0	0	0	0	747	762			PEAKS DB
K.FSVSPVVR.V	Y	68.20	889.5021	8	0.8	445.7568	2	30.49	2	F2:84306	20210611-09_2021021-OK160A_816_5.3.236.mgf		0	0	0	0	460	467			PEAKS DB
R.NMSVIAHVDHGK.S	Y	61.46	1306.6451	12	2.0	436.5547	3	19.04	2	F2:64665	20210611-09_2021021-OK160A_816_5.3.236.mgf		0	0	0	0	21	32			PEAKS DB
M.VNFTVDQIR.A	Y	59.81	1090.5771	9	1.3	546.2943	2	34.09	2	F2:90876	20210611-09_2021021-OK160A_816_5.3.236.mgf		0	0	0	0	2	10			PEAKS DB
**total 4 peptides**																					

gene:107289560

back to list

| Protein Coverage | Supporting Peptides |

Protein Coverage:

1 MDDREDLVYQ AKLAEQAERY DEMVESMKKV AGMDVELTVE ER**NLLSVAYK** NVIGARRASW RIISSIEQKE ENKGGEDKLK

81 MIREYRQMVE TELKLICCDI LDVLDKHLIP AANTGESKVF YYKMKGDYHR YLAEFATGND RKEAAENSLV AYKAASDIAM
161 TELPPTHPIR LGLALNFSVF YYEILNSPDR ACRLAK**AAFD DAIAELDTLS EESYKDSTLI MQLLR**DNLTL WTSDMQGDGE

241 EQSKEALQDV EDETQ

Supporting Peptides:

Peptide	Uniq	-10lgP	Mass	Length	ppm	m/z	z	RT	Fraction	Scan	Source File	Area ebraeus	Area judaeus	#Feature	#Feature ebraeus	#Feature judaeus	Start	End	PTM	AScore	Found By
K.AAFDDAIAELDTLSEESYK.D	Y	106.31	2086.9583	19	5.3	1044.4875	2	58.44	2	F2:130765	20210611-09_2021021-OK160A_816_5.3.236.mgf		0	0	0	0	197	215			PEAKS DB
K.DSTLIMQLLR.D	Y	79.20	1188.6537	10	-2.2	595.3303	2	56.63	2	F2:128557	20210611-09_2021021-OK160A_816_5.3.236.mgf	0	0	0	0	0	216	225			PEAKS DB
R.NLLSVAYK.N	Y	68.03	906.5175	8	2.8	454.2654	2	33.79	2	F2:90231	20210611-09_2021021-OK160A_816_5.3.236.mgf	0	0	0	0	0	43	50			PEAKS DB
**total 3 peptides**																					

OK193_E_248_Conodipine

back to list

| Protein Coverage | Supporting Peptides |

Protein Coverage:

1 MKMLESALWI LAALALPWIV AQDSSTAELC KINSNGCSVP FAFIPCQKHF LAACDIHDNC YLCGAHFGLT RNNCDNAFLT

C Carbamidomethylation (+57.02)

81 HMTALCAQGT DEGGFCLERR KRR**EASSMFI TTTPLR**QLRL LEK**LMPPNSL SDHDSR**QPQQ R**FFTCTDWAK NYFDAVR**ISG

161 SSYFSDTANA TYCPQFVDCM P

Supporting Peptides:

Peptide	Uniq	-10lgP	Mass	Length	ppm	m/z	z	RT	Fraction	Scan	Source File	Area ebraeus	Area judaeus	#Feature	#Feature ebraeus	#Feature judaeus	Start	End	PTM	AScore	Found By
K.LMPPNSLSDHDSR.Q	N	82.88	1467.6776	13	-0.2	490.2312	3	23.57	1	F1:16022	20210611-08_2021021-OK192A_815_5.3.236.mgf	0		0	0	0	124	136			PEAKS DB
R.EASSMFITTTPLR.Q	N	80.11	1452.7283	13	1.2	727.3695	2	41.94	1	F1:39695	20210611-08_2021021-OK192A_815_5.3.236.mgf	0		0	0	0	104	116			PEAKS DB
K.NYFDAVR.I	Y	78.72	883.4188	7	2.1	442.7159	2	29.12	1	F1:23959	20210611-08_2021021-OK192A_815_5.3.236.mgf	0		0	0	0	151	157			PEAKS DB
R.FFTC(+57.02)TDWAK.N	Y	66.58	1174.5117	9	3.5	588.2629	2	37.66	1	F1:35030	20210611-08_2021021-OK192A_815_5.3.236.mgf	0		0	0	0	142	150	Carbamidomethylation	C4:Carbamidomethylation:1000.00	PEAKS DB
total 4 peptides																					

SRX1323887_E_230_Conodipine

back to list

| Protein Coverage | Supporting Peptides |

Protein Coverage:

1 MKMLESALWI LAALALTWIV AQDSSTAELC KINSNGCSVP FAFIPCQKHF LAACDIHDNC YLCGAHFGLT RNNCDNAFLT

C Carbamidomethylation (+57.02)

81 HMTALCAQGT DEGGFCLERR KRR**EASSMFI TTTPLR**QLRL LEK**LMPPNSL SDHDSR**QPQQ R**FFTCTDWAK NYFDAVR**ISG

161 SSYFSDTANA TYCPQFVDCM P

Supporting Peptides:

Peptide	Uniq	-10lgP	Mass	Length	ppm	m/z	z	RT	Fraction	Scan	Source File	Area ebraeus	Area judaeus	#Feature	#Feature ebraeus	#Feature judaeus	Start	End	PTM	AScore	Found By
K.LMPPNSLSDHDSR.Q	N	82.88	1467.6776	13	-0.2	490.2312	3	23.57	1	F1:16022	20210611-08_2021021-OK192A_815_5.3.236.mgf	0		0	0	0	124	136			PEAKS DB
R.EASSMFITTTPLR.Q	N	80.11	1452.7283	13	1.2	727.3695	2	41.94	1	F1:39695	20210611-08_2021021-OK192A_815_5.3.236.mgf	0		0	0	0	104	116			PEAKS DB
K.NYFDAVR.I	Y	78.72	883.4188	7	2.1	442.7159	2	29.12	1	F1:23959	20210611-08_2021021-OK192A_815_5.3.236.mgf	0		0	0	0	151	157			PEAKS DB
R.FFTC(+57.02)TDWAK.N	Y	66.58	1174.5117	9	3.5	588.2629	2	37.66	1	F1:35030	20210611-08_2021021-OK192A_815_5.3.236.mgf	0		0	0	0	142	150	Carbamidomethylation	C4:Carbamidomethylation:1000.00	PEAKS DB
total 4 peptides																					

OK213_E_301_Conodipine

back to list

| Protein Coverage | Supporting Peptides |

Protein Coverage:

1 MKMLESALWI LAALALTWIV AQDSSTAELC KINSNGCSVP SALIPCQKHF LAACDIHDNC YLCGAHFGLT RNNCDNAFLT

C Carbamidomethylation (+57.02)

81 HMTALCAQGT DEGGFCLERR KRR**EASSMFI TTTPLR**QLRL LEK**LMPPNSL SDHDSR**QPQQ R**FFTCTDWAK NYFDAVR**ISG

161 SSYFSDTANA TYCPQFVDCM PQV

Supporting Peptides:

Peptide	Uniq	-10lgP	Mass	Length	ppm	m/z	z	RT	Fraction	Scan	Source File	Area ebraeus	Area judaeus	#Feature	#Feature ebraeus	#Feature judaeus	Start	End	PTM	AScore	Found By
K.LMPPNSLSDHDSR.Q	N	82.88	1467.6776	13	-0.2	490.2312	3	23.57	1	F1:16022	20210611-08_2021021-OK192A_815_5.3.236.mgf	0		0	0	0	124	136			PEAKS DB
R.EASSMFITTTPLR.Q	N	80.11	1452.7283	13	1.2	727.3695	2	41.94	1	F1:39695	20210611-08_2021021-OK192A_815_5.3.236.mgf	0		0	0	0	104	116			PEAKS DB
K.NYFDAVR.I	Y	78.72	883.4188	7	2.1	442.7159	2	29.12	1	F1:23959	20210611-08_2021021-OK192A_815_5.3.236.mgf	0		0	0	0	151	157			PEAKS DB
R.FFTC(+57.02)TDWAK.N	Y	66.58	1174.5117	9	3.5	588.2629	2	37.66	1	F1:35030	20210611-08_2021021-OK192A_815_5.3.236.mgf	0		0	0	0	142	150	Carbamidomethylation	C4:Carbamidomethylation:1000.00	PEAKS DB
total 4 peptides																					

OK006_E_272_Conodipine

back to list

| Protein Coverage | Supporting Peptides |

Protein Coverage:

1 MKMLESALWI LAALALTWIV AQDSSTAELC KINSNGCSVP SALIPCQKHF LAACDIHDNC YLCGAHFGLT RNNCDNAFLT

C Carbamidomethylation (+57.02)

81 HMTALCAQGT DEGGFCLERR KRR**EASSMFI TTTPLR**QLRL LEK**LMPPNSL SDHDSR**QPQQ R**FFTCTDWAK NYFDAVR**ISG

161 SSYFSDTANA TYCPQFVDCM PQV

Supporting Peptides:

Peptide	Uniq	-10lgP	Mass	Length	ppm	m/z	z	RT	Fraction	Scan	Source File	Area ebraeus	Area judaeus	#Feature	#Feature ebraeus	#Feature judaeus	Start	End	PTM	AScore	Found By
K.LMPPNSLSDHDSR.Q	N	82.88	1467.6776	13	-0.2	490.2312	3	23.57	1	F1:16022	20210611-08_2021021-OK192A_815_5.3.236.mgf	0		0	0	0	124	136			PEAKS DB
R.EASSMFITTTPLR.Q	N	80.11	1452.7283	13	1.2	727.3695	2	41.94	1	F1:39695	20210611-08_2021021-OK192A_815_5.3.236.mgf	0		0	0	0	104	116			PEAKS DB
K.NYFDAVR.I	Y	78.72	883.4188	7	2.1	442.7159	2	29.12	1	F1:23959	20210611-08_2021021-OK192A_815_5.3.236.mgf	0		0	0	0	151	157			PEAKS DB
R.FFTC(+57.02)TDWAK.N	Y	66.58	1174.5117	9	3.5	588.2629	2	37.66	1	F1:35030	20210611-08_2021021-OK192A_815_5.3.236.mgf	0		0	0	0	142	150	Carbamidomethylation	C4:Carbamidomethylation:1000.00	PEAKS DB
total 4 peptides																					

V_1302_329_T-3-WF

back to list

| Protein Coverage | Supporting Peptides |

Protein Coverage:

1 SPAAPNPVER R**IQSDLIRAG LEGADMETEE R**FIGALFGPV VDAVTSLFKG RKK

O Oxidation (M) (+15.99)

Supporting Peptides:

Peptide	Uniq	-10lgP	Mass	Length	ppm	m/z	z	RT	Fraction	Scan	Source File	Area ebraeus	Area judaeus	#Feature	#Feature ebraeus	#Feature judaeus	Start	End	PTM	AScore	Found By
R.AGLEGADMETEER.F	Y	130.51	1406.5984	13	3.6	704.3063	2	24.58	1	F1:17622	20210611-08_2021021-OK192A_815_5.3.236.mgf	0		0	0	0	19	31			PEAKS DB
R.AGLEGADM(+15.99)ETEER.F	Y	123.92	1422.5933	13	-1.7	712.3000	2	18.84	1	F1:10158	20210611-08_2021021-OK192A_815_5.3.236.mgf	0	0	0	0	0	19	31	Oxidation (M)	M8:Oxidation (M):1000.00	PEAKS DB

total 3 peptides

Peptide	Uniq	-10lgP	Mass	Length	ppm	m/z	z	RT	Fraction	Scan	Source File	Area ebraeus	Area judaeus	#Feature	#Feature ebraeus	#Feature judaeus	Start	End	PTM	AScore	Found By
R.IQSDLIR.A	N	59.76	843.4814	7	4.4	422.7482	2	23.53	1	F1:15827	20210611-08_2021021-OK192A_815_5.3.236.mgf	0	0	0	0	0	12	18			PEAKS DB
total 3 peptides																					

V_1302_150_T-3-WF

back to list

| Protein Coverage | Supporting Peptides |

Protein Coverage:

21
1 NPLETRIQSD LIRAGLEGAD METEERFIGA LVGPVVNALT SLFKGRKK

Oxidation (M) (+15.99)

Supporting Peptides:

Peptide	Uniq	-10lgP	Mass	Length	ppm	m/z	z	RT	Fraction	Scan	Source File	Area ebraeus	Area judaeus	#Feature	#Feature ebraeus	#Feature judaeus	Start	End	PTM	AScore	Found By
R.AGLEGADMETEER.F	Y	130.51	1406.5984	13	3.6	704.3063	2	24.58	1	F1:17622	20210611-08_2021021-OK192A_815_5.3.236.mgf	0		0	0	0	14	26			PEAKS DB
R.AGLEGADM(+15.99)ETEER.F	Y	123.92	1422.5933	13	-1.7	712.3000	2	18.84	1	F1:10158	20210611-08_2021021-OK192A_815_5.3.236.mgf	0	0	0	0	0	14	26	Oxidation (M)	M8:Oxidation (M):1000.00	PEAKS DB
R.IQSDLIR.A	N	59.76	843.4814	7	4.4	422.7482	2	23.53	1	F1:15827	20210611-08_2021021-OK192A_815_5.3.236.mgf	0	0	0	0	0	7	13			PEAKS DB
total 3 peptides																					

V_1258_142_T-3-WF

back to list

| Protein Coverage | Supporting Peptides |

Protein Coverage:

21
1 NPLETRIQSD LIRAGLEGAD METEERFIGA LVGPVVNALT SLFKGRKK

Oxidation (M) (+15.99)

Supporting Peptides:

Peptide	Uniq	-10lgP	Mass	Length	ppm	m/z	z	RT	Fraction	Scan	Source File	Area ebraeus	Area judaeus	#Feature	#Feature ebraeus	#Feature judaeus	Start	End	PTM	AScore	Found By
R.AGLEGADMETEER.F	Y	130.51	1406.5984	13	3.6	704.3063	2	24.58	1	F1:17622	20210611-08_2021021-OK192A_815_5.3.236.mgf	0		0	0	0	14	26			PEAKS DB
R.AGLEGADM(+15.99)ETEER.F	Y	123.92	1422.5933	13	-1.7	712.3000	2	18.84	1	F1:10158	20210611-08_2021021-OK192A_815_5.3.236.mgf	0	0	0	0	0	14	26	Oxidation (M)	M8:Oxidation (M):1000.00	PEAKS DB
R.IQSDLIR.A	N	59.76	843.4814	7	4.4	422.7482	2	23.53	1	F1:15827	20210611-08_2021021-OK192A_815_5.3.236.mgf	0	0	0	0	0	7	13			PEAKS DB
total 3 peptides																					

V_1302_151_T-3-WF

back to list

| Protein Coverage | Supporting Peptides |

Protein Coverage:

21
1 NPLETRIQSD LIRAGLEGAD METEERFLGS LPTYATIFYR VVRNNQEALK

Oxidation (M) (+15.99)

Supporting Peptides:

Peptide	Uniq	-10lgP	Mass	Length	ppm	m/z	z	RT	Fraction	Scan	Source File	Area ebraeus	Area judaeus	#Feature	#Feature ebraeus	#Feature judaeus	Start	End	PTM	AScore	Found By
R.AGLEGADMETEER.F	Y	130.51	1406.5984	13	3.6	704.3063	2	24.58	1	F1:17622	20210611-08_2021021-OK192A_815_5.3.236.mgf	0		0	0	0	14	26			PEAKS DB
R.AGLEGADM(+15.99)ETEER.F	Y	123.92	1422.5933	13	-1.7	712.3000	2	18.84	1	F1:10158	20210611-08_2021021-OK192A_815_5.3.236.mgf	0	0	0	0	0	14	26	Oxidation (M)	M8:Oxidation (M):1000.00	PEAKS DB
R.IQSDLIR.A	N	59.76	843.4814	7	4.4	422.7482	2	23.53	1	F1:15827	20210611-08_2021021-OK192A_815_5.3.236.mgf	0	0	0	0	0	7	13			PEAKS DB
total 3 peptides																					

V_1258_074_T-3-WF

back to list

| Protein Coverage | Supporting Peptides |

Protein Coverage:

23
1 APTPVERRIQ SDLIRAGLEG ADMETEERFL GSLPTYATIF YRVVRNNQEA LK

Oxidation (M) (+15.99)

Supporting Peptides:

Peptide	Uniq	-10lgP	Mass	Length	ppm	m/z	z	RT	Fraction	Scan	Source File	Area ebraeus	Area judaeus	#Feature	#Feature ebraeus	#Feature judaeus	Start	End	PTM	AScore	Found By
R.AGLEGADMETEER.F	Y	130.51	1406.5984	13	3.6	704.3063	2	24.58	1	F1:17622	20210611-08_2021021-OK192A_815_5.3.236.mgf	0		0	0	0	16	28			PEAKS DB
R.AGLEGADM(+15.99)ETEER.F	Y	123.92	1422.5933	13	-1.7	712.3000	2	18.84	1	F1:10158	20210611-08_2021021-OK192A_815_5.3.236.mgf	0	0	0	0	0	16	28	Oxidation (M)	M8:Oxidation (M):1000.00	PEAKS DB
R.IQSDLIR.A	N	59.76	843.4814	7	4.4	422.7482	2	23.53	1	F1:15827	20210611-08_2021021-OK192A_815_5.3.236.mgf	0	0	0	0	0	9	15			PEAKS DB
total 3 peptides																					

gene:107292247

back to list

| Protein Coverage | Supporting Peptides |

Protein Coverage:

1 MPLRLDIKRK LTARSDRVKS VDLHPTEPWM LASLYNGSVC VWNHETQTLV KTFEVCDLPV RAAKFVARKN WVVTGADDMQ
81 IKVFNYNTLE RVHMFEAHSD YIRCIAVHPT QPFILTSSDD MLIKLWDWDK KWACSQVFEG HTHYVMQIVI NPKDNNQFAS
161 TSLDRTIKVW QLGSSSPNFT LEGHEKGVNS IDYYSGGDKP YLISGADDRL VKIWDYQNKT CVQILEGHAQ NVSCVSFHPE
241 LPIIITGSED GTVRIWHSST YRLESTLNYG MERVWCVASL RGSNNVALGY DEGSIIVKLG REEPAMSMDA NGKIIWAKHS
321 EIQQANLKAM GDTEIKDGER LPLAVKDMGS CEIYPQTIQH NPNGRFVVVC GDGEYIIYTA MALRNKSFGS AQEFAWAHDS
401 SEYAIRESNS SVKIFKNFKE KKSFKPDFGA EGIYGGFLLG VRSVNGLAFY DWENTELIRR IEIQPKHIFW SDSGELVCIA
481 TEESFFILKY LSEKVATAQD THEGVTEDGI EDAFEVLGEI QEIVKTGLWV GDCFIYTSSV NRLNYYVGGE IVTIAHLDRT
561 MYLLGYIPKD NRLYLGDKEL NIVSYSLLVS VLEYQTAVMR RDFSMADKVL PTIPKEQRTR VAHFLEKQGF KQQALAVSTD
641 PEHRFELALQ LGELKIANQL ALEAESEQKW KQLAELAINK CQFGLAQECL HHAQDYGGLL LLATASGNTT MVNKLAEAAE
721 KDGKNNVAFM SYFLQGELDH CLELLIKTGR LPEAAFLART YLPSQVSRVV KLWRENLSKI NQKAAESLAD PTEYENLFPG
801 LKEAFVAEEY IQKSQTLSRP SSEYPLVTPN EERNILEEAK DFTLQNKVSE ESEDESVPSP RPVIEPPVAE KVPVEIKDEK
881 ILLDLDVDLD NLELEDIDTT DIFLDEEMSD

Supporting Peptides:

Peptide	Uniq	-10lgP	Mass	Length	ppm	m/z	z	RT	Fraction	Scan	Source File	Area ebraeus	Area judaeus	#Feature	#Feature ebraeus	#Feature judaeus	Start	End	PTM	AScore	Found By
K.HSEIQQANLK.A	Y	95.90	1166.6044	10	4.5	584.3096	2	14.53	2	F2:58162	20210611-09_2021021-OK160A_816_5.3.236.mgf		0	0	0	0	319	328			PEAKS DB
K.VFNYNTLER.V	Y	74.51	1154.5720	9	2.9	578.2925	2	31.81	2	F2:86429	20210611-09_2021021-OK160A_816_5.3.236.mgf		0	0	0	0	83	91			PEAKS DB
R.TYLPSQVSR.V	Y	70.11	1049.5505	9	4.9	525.7829	2	25.12	2	F2:74725	20210611-09_2021021-OK160A_816_5.3.236.mgf		0	0	0	0	760	768			PEAKS DB
**total 3 peptides**																					

OK212_J_239_Cerm08

back to list

| Protein Coverage | Supporting Peptides |

Protein Coverage:

53 C

1 **TVTLDTCASA K**VVCAWAGQG ESCSCSATHS CTQDDAHKVE VNMER**GVQAL YTCQQISDFD K**CTATGAVMP ADLSQLYCRC

C Carbamidomethylation (+57.02)

81 SNNKYTIEND DVVC

Supporting Peptides:

Peptide	Uniq	-10lgP	Mass	Length	ppm	m/z	z	RT	Fraction	Scan	Source File	Area ebraeus	Area judaeus	#Feature	#Feature ebraeus	#Feature judaeus	Start	End	PTM	AScore	Found By
R.GVQALYTC(+57.02)QQISDFDK.C	Y	119.48	1871.8723	16	2.9	936.9422	2	40.69	2	F2:102502	20210611-09_2021021-OK160A_816_5.3.236.mgf		0	0	0	0	46	61	Carbamidomethylation	C8:Carbamidomethylation:1000.00	PEAKS DB
TVTLDTC(+57.02)ASAK.V	Y	66.61	1165.5649	11	1.1	583.7880	2	20.65	2	F2:66937	20210611-09_2021021-OK160A_816_5.3.236.mgf		0	0	0	0	1	11		C7:Carbamidomethylation:1000.00	PEAKS DB
**total 2 peptides**																					

CebrOK033_J_197_Cerm08

back to list

| Protein Coverage | Supporting Peptides |

Protein Coverage:

53 C

1 **TVTLDTCASA K**VVCAWAGQG ESCSCSATHS CTQDDAHKVE VNMER**GVQAL YTCQQISDFD K**CTATGAVMP ADLSQLYCRC

C Carbamidomethylation (+57.02)

81 SNNKYTIEND DVVC

Supporting Peptides:

Peptide	Uniq	-10lgP	Mass	Length	ppm	m/z	z	RT	Fraction	Scan	Source File	Area ebraeus	Area judaeus	#Feature	#Feature ebraeus	#Feature judaeus	Start	End	PTM	AScore	Found By
R.GVQALYTC(+57.02)QQISDFDK.C	Y	119.48	1871.8723	16	2.9	936.9422	2	40.69	2	F2:102502	20210611-09_2021021-OK160A_816_5.3.236.mgf		0	0	0	0	46	61	Carbamidomethylation	C8:Carbamidomethylation:1000.00	PEAKS DB
TVTLDTC(+57.02)ASAK.V	Y	66.61	1165.5649	11	1.1	583.7880	2	20.65	2	F2:66937	20210611-09_2021021-OK160A_816_5.3.236.mgf		0	0	0	0	1	11		C7:Carbamidomethylation:1000.00	PEAKS DB
**total 2 peptides**																					

gene:107285995

back to list

| Protein Coverage | Supporting Peptides |

Protein Coverage:

1 MGNIFANLFK GLFGKKEMR**I LMVGLDAAGK** TTILYKLK**LG EIVTTIPTIG FNVETVEYKN ISFTVWDVGG QDK**IRPLWRH

81 YFQNTQGLIF VVDSNDRERV NEAREELMRM LAEDELRDAV LLVFANKQDL PNAMNAAEIT DKLGLHSLRH RNWYIQATCA

161 TSGDGLYEGL DWLSNQLRNQ K

Supporting Peptides:

Peptide	Uniq	-10lgP	Mass	Length	ppm	m/z	z	RT	Fraction	Scan	Source File	Area ebraeus	Area judaeus	#Feature	#Feature ebraeus	#Feature judaeus	Start	End	PTM	AScore	Found By
K.NISFTVWDVGGQDK.I	Y	89.28	1564.7522	14	-0.3	783.3802	2	48.12	1	F1:46181	20210611-08_2021021-OK192A_815_5.3.236.mgf	0	0	0	0	0	60	73			PEAKS DB
K.LGEIVTTIPTIGFNVETVEYK.N	Y	89.20	2322.2358	21	9.9	1162.1318	2	57.68	2	F2:129913	20210611-09_2021021-OK160A_816_5.3.236.mgf		0	0	0	0	39	59			PEAKS DB
R.ILMVGLDAAGK.T	Y	54.33	1086.6107	11	0.2	544.3105	2	39.00	2	F2:99680	20210611-09_2021021-OK160A_816_5.3.236.mgf		0	0	0	0	20	30			PEAKS DB
**total 3 peptides**																					

V_CG13_085_B2

back to list

| Protein Coverage | Supporting Peptides |

Protein Coverage:

1 YPQRREGAPA DAANLQGFDQ GMQAMPNMQG MGMQPMQAMP GMGAGQMLPF NPNFALGYKR AFDENLEKRR EHSK**FNDDNM**

O Oxidation (M) (+15.99)

93 O

81 **SPFDSGDGLG NFMNFMKDNG NNLPFANMDS AATDLGNFQP SAENEDGK**FR FFDQQQ

Supporting Peptides:

Peptide	Uniq	-10lgP	Mass	Length	ppm	m/z	z	RT	Fraction	Scan	Source File	Area ebraeus	Area judaeus	#Feature	#Feature ebraeus	#Feature judaeus	Start	End	PTM	A[illegible]
K.FNDDNM(+15.99)SPFDSGDGLGNFM(+15.99)NFM(+15.99)K.D	Y	105.24	2647.0300	23	4.3	883.3511	3	48.22	1	F1:46215	20210611-08_2021021-OK192A_815_5.3.236.mgf	0		0	0	0	75	97	Oxidation (M)	[illegible]
K.DNGNNLPFANMDSAATDLGNFQPSAENEDGK.F	Y	90.32	3252.3901	31	9.1	1085.1431	3	53.35	1	F1:49178	20210611-08_2021021-OK192A_815_5.3.236.mgf	0		0	0	0	98	128		
K.DNGNNLPFANM(+15.99)DSAATDLGNFQPSAENEDGK.F	Y	89.17	3268.3850	31	7.9	1090.4734	3	46.90	1	F1:45224	20210611-08_2021021-OK192A_815_5.3.236.mgf	0		0	0	0	98	128		[illegible]
**total 3 peptides**																				

OK214_J_237_Cerm03

back to list

| Protein Coverage | Supporting Peptides |

Protein Coverage:

1 LMPKTATK**RK DVAPNDRHAV DSQK**LKRFC

Supporting Peptides:

Peptide	Uniq	-10lgP	Mass	Length	ppm	m/z	z	RT	Fraction	Scan	Source File	Area ebraeus	Area judaeus	#Feature	#Feature ebraeus	#Feature judaeus	Start	End	PTM	AScore	Found By
R.KDVAPNDRHAVDSQK.L	Y	80.06	1678.8386	15	-1.6	560.6169	3	9.36	2	F2:52796	20210611-09_2021021-OK160A_816_5.3.236.mgf		0	0	0	0	10	24			PEAKS DB
R.HAVDSQK.L	Y	67.82	783.3875	7	6.3	392.7018	2	5.11	2	F2:52246	20210611-09_2021021-OK160A_816_5.3.236.mgf		0	0	0	0	18	24			PEAKS DB
K.RKDVAPNDRHAVDSQK.L	Y	67.09	1834.9397	16	1.7	459.7411	4	8.74	2	F2:52569	20210611-09_2021021-OK160A_816_5.3.236.mgf		0	0	0	0	9	24			PEAKS DB
R.KDVAPNDR.H	Y	54.21	913.4617	8	0.6	457.7365	2	6.98	2	F2:52348	20210611-09_2021021-OK160A_816_5.3.236.mgf		0	0	0	0	10	17			PEAKS DB
**total 4 peptides**																					

OK212_J_300_Thioredoxin

back to list

| Protein Coverage | Supporting Peptides |

Protein Coverage:

```
  1 MAANTAHVVC LIALFILVCV SCDNDHGSDA VSYERDAFQS AINDKKHFVM FFAPWCGHCK RLAPTWNELA KVYNNDESSV
 81 TIAKVDCTVE TALCSEHDVT GYPTLKFFHK KADDFQRYKG NRDLDSLKKF VEEQASRTDS EGEQATQAEE PGAGALVELT
161 DDTFDAFIQK GFHFVKFYAP WCGHCKRLAP TWEELASSFA ENQQVSVSKV DCTVSTKLCT SQGVRGYPTL ILFNDGDKFE
241 QYQGSRSLED LKGFVTSKME EVLGFQQQEA VPEEEQEEAV VDLTGDTFEE VISEGLTFVK FFAPWCGHCK RLAPTWEELA
321 RTNMDKDIVI ARVDCTQHKS VCDEQKVQGY PTLVLFQNGK RKNDYKGQRD LGSLQGFVDS HVEHTEL
```

Supporting Peptides:

Peptide	Uniq	-10lgP	Mass	Length	ppm	m/z	z	RT	Fraction	Scan	Source File	Area ebraeus	Area judaeus	#Feature	#Feature ebraeus	#Feature judaeus	Start	End	PTM	AScore	Found By
K.VYNNDESSVTIAK.V	Y	112.20	1438.6940	13	2.0	720.3527	2	22.87	2	F2:70797	20210611-09_2021021-OK160A_816_5.3.236.mgf		0	0	0	0	72	84			PEAKS DB
R.DLGSLQGFVDSHVEHTEL	Y	72.82	1981.9381	18	-5.7	661.6468	3	52.41	2	F2:122758	20210611-09_2021021-OK160A_816_5.3.236.mgf		0	0	0	0	370	387			PEAKS DB
**total 2 peptides**																					

OK005_J_306_Thioredoxin

back to list

| Protein Coverage | Supporting Peptides |

Protein Coverage:

```
  1 MAANTAHVVC LIALFILVCV SCDNDHGSDA VSYERDAFQS AINDKKHFVM FFAPWCGHCK RLAPTWNELA KVYNNDESSV
 81 TIAKVDCTVE TALCSEHDVT GYPTLKFFHK KADDFQRYKG NRDLDSLKKF VEEQASRTDS EGEQATQAEE PGAGALVELT
161 DDTFDAFIQK GFHFVKFYAP WCGHCKRLAP TWEELASSFA ENQQVSVSKV DCTVSTKLCT SQGVRGYPTL ILFNDGDKFE
241 QYQGSRSLED LKGFVTSKME EVLGFQQQEA VPEEEQEEAV VDLTGDTFEE VISEGLTFVK FFAPWCGHCK RLAPTWEELA
321 RTNMDKDIVI ARVDCTQHKS VCDEQKVQGY PTLVLFQNGK RKNDYKGQRD LGSLQGFVDS HVEHTEL
```

Supporting Peptides:

Peptide	Uniq	-10lgP	Mass	Length	ppm	m/z	z	RT	Fraction	Scan	Source File	Area ebraeus	Area judaeus	#Feature	#Feature ebraeus	#Feature judaeus	Start	End	PTM	AScore	Found By
K.VYNNDESSVTIAK.V	Y	112.20	1438.6940	13	2.0	720.3527	2	22.87	2	F2:70797	20210611-09_2021021-OK160A_816_5.3.236.mgf		0	0	0	0	72	84			PEAKS DB
R.DLGSLQGFVDSHVEHTEL	Y	72.82	1981.9381	18	-5.7	661.6468	3	52.41	2	F2:122758	20210611-09_2021021-OK160A_816_5.3.236.mgf		0	0	0	0	370	387			PEAKS DB
**total 2 peptides**																					

SRX1323887_E_229_Conodipine

back to list

| Protein Coverage | Supporting Peptides |

Protein Coverage:

```
  1 MKMLESALWI LAALALTWIV AQDSSTAELC KINSNGCSVP FAFIPCQKHF LAACDIHDNC YLCGAHFGLT RNNCDNAFLT
                                                                          145
 81 HMTALCAQGT DEGGFCLERR KRREASSMFI TTTPLRQLRL LEKLMPPNSL SDHDSRQPQQ RFFTCTEWAE KYFDAVRTSG
161 SSYFSDTANA TYCPQFVDCM P
```

C Carbamidomethylation (+57.02)

Supporting Peptides:

Peptide	Uniq	-10lgP	Mass	Length	ppm	m/z	z	RT	Fraction	Scan	Source File	Area ebraeus	Area judaeus	#Feature	#Feature ebraeus	#Feature judaeus	Start	End	PTM	AScore	Found By
K.LMPPNSLSDHDSR.Q	N	82.88	1467.6776	13	-0.2	490.2312	3	23.57	1	F1:16022	20210611-08_2021021-OK192A_815_5.3.236.mgf	0		0	0	0	124	136			PEAKS DB
R.EASSMFITTTPLR.Q	N	80.11	1452.7283	13	1.2	727.3695	2	41.94	1	F1:39695	20210611-08_2021021-OK192A_815_5.3.236.mgf	0		0	0	0	104	116			PEAKS DB
R.FFTC(+57.02)TEWAEK.Y	Y	68.97	1317.5699	10	0.2	659.7899	2	37.22	1	F1:34554	20210611-08_2021021-OK192A_815_5.3.236.mgf	0		0	0	0	142	151	Carbamidomethylation	C4:Carbamidomethylation:1000.00	PEAKS DB
**total 3 peptides**																					

OK193_E_247_Conodipine

back to list

| Protein Coverage | Supporting Peptides |

Protein Coverage:

```
  1 MKMLESALWI LAALALPWIV AQDSSTAELC KINSNGCSVP FAFIPCQKHF LAACDIHDNC YLCGAHFGLT RNNCDNAFLT
                                                                          145
 81 HMTALCAQGT DEGGFCLERR KRREASSMFI TTTPLRQLRL LEKLMPPNSL SDHDSRQPQQ RFFTCTEWAE KYFDAVRTSG
161 SSYFSDTANA TYCPQFVDCM P
```

C Carbamidomethylation (+57.02)

Supporting Peptides:

Peptide	Uniq	-10lgP	Mass	Length	ppm	m/z	z	RT	Fraction	Scan	Source File	Area ebraeus	Area judaeus	#Feature	#Feature ebraeus	#Feature judaeus	Start	End	PTM	AScore	Found By
K.LMPPNSLSDHDSR.Q	N	82.88	1467.6776	13	-0.2	490.2312	3	23.57	1	F1:16022	20210611-08_2021021-OK192A_815_5.3.236.mgf	0		0	0	0	124	136			PEAKS DB
R.EASSMFITTTPLR.Q	N	80.11	1452.7283	13	1.2	727.3695	2	41.94	1	F1:39695	20210611-08_2021021-OK192A_815_5.3.236.mgf	0		0	0	0	104	116			PEAKS DB
R.FFTC(+57.02)TEWAEK.Y	Y	68.97	1317.5699	10	0.2	659.7899	2	37.22	1	F1:34554	20210611-08_2021021-OK192A_815_5.3.236.mgf	0		0	0	0	142	151	Carbamidomethylation	C4:Carbamidomethylation:1000.00	PEAKS DB
**total 3 peptides**																					

gene:107298953

back to list

| Protein Coverage | Supporting Peptides |

Protein Coverage:

```
  1 MAVTVFLLAC SRVGAGAPVY LAAVLEYLTA EILELAGNAA RDNKKTRIIP RHLQLAIRND EELNKLLGKV TIAQGGVLPN
 81 IQAVLLPKKT ESHKAKGK
```

Supporting Peptides:

Peptide	Uniq	-10lgP	Mass	Length	ppm	m/z	z	RT	Fraction	Scan	Source File	Area ebraeus	Area judaeus	#Feature	#Feature ebraeus	#Feature judaeus	Start	End	PTM	AScore	Found By
R.VGAGAPVYLAAVLEYLTAEILELAGNAAR.D	Y	106.24	2914.5803	29	2.0	972.5319	3	66.71	2	F2:134483	20210611-09_2021021-OK160A_816_5.3.236.mgf		0	0	0	0	13	41			PEAKS DB
R.HLQLAIR.N	Y	73.85	849.5184	7	2.8	425.7659	2	24.26	2	F2:73252	20210611-09_2021021-OK160A_816_5.3.236.mgf	0	0	0	0	0	52	58			PEAKS DB
total 2 peptides																					

OK212_J_224_Cerm03

back to list

| Protein Coverage | Supporting Peptides |

Protein Coverage:

1 EISRR**TIDGG EDGVLAGDR**T TNK**NALLQQR**

Supporting Peptides:

Peptide	Uniq	-10lgP	Mass	Length	ppm	m/z	z	RT	Fraction	Scan	Source File	Area ebraeus	Area judaeus	#Feature	#Feature ebraeus	#Feature judaeus	Start	End	PTM	AScore	Found By
R.TIDGGEDGVLAGDR.T	Y	104.53	1373.6422	14	4.4	687.8285	2	29.96	2	F2:83337	20210611-09_2021021-OK160A_816_5.3.236.mgf		0	0	0	0	6	19			PEAKS DB
K.NALLQQR	N	64.80	841.4770	7	4.5	421.7459	2	18.33	2	F2:63419	20210611-09_2021021-OK160A_816_5.3.236.mgf	0	0	0	0	0	24	30			PEAKS DB
total 2 peptides																					

OK213_E_102_M

back to list

| Protein Coverage | Supporting Peptides |

Protein Coverage:

1 **LDADQPVER**E AENKQDLHSD ER**TGFILPAQ K**

Supporting Peptides:

Peptide	Uniq	-10lgP	Mass	Length	ppm	m/z	z	RT	Fraction	Scan	Source File	Area ebraeus	Area judaeus	#Feature	#Feature ebraeus	#Feature judaeus	Start	End	PTM	AScore	Found By
LDADQPVER.E	Y	92.28	1041.5090	9	3.1	521.7612	2	17.81	2	F2:62607	20210611-09_2021021-OK160A_816_5.3.236.mgf	0	0	0	0	0	1	9			PEAKS DB
R.TGFILPAQK	Y	88.10	973.5596	9	3.3	487.7868	2	33.01	1	F1:29289	20210611-08_2021021-OK192A_815_5.3.236.mgf	0	0	0	0	0	23	31			PEAKS DB
total 2 peptides																					

V_CG13_016_Conkunitzin-12

back to list

| Protein Coverage | Supporting Peptides |

Protein Coverage:

1 ESIHICDK**AK DEGTGSQNIA SYFFDR**DTGT CETFNYTGGN YDTFPNRFNN CPVCENTCKC KLRKDPGTGE RRRTRYYHSS

81 DGSGCLPFEY RGIGGNANNF DSESACINEC EL

Supporting Peptides:

Peptide	Uniq	-10lgP	Mass	Length	ppm	m/z	z	RT	Fraction	Scan	Source File	Area ebraeus	Area judaeus	#Feature	#Feature ebraeus	#Feature judaeus	Start	End	PTM	AScore	Found By
K.DEGTGSQNIASYFFDR.D	Y	97.30	1805.7856	16	-0.2	903.8965	2	51.83	1	F1:48344	20210611-08_2021021-OK192A_815_5.3.236.mgf	0		0	0	0	11	26			PEAKS DB
K.AKDEGTGSQNIASYFFDR.D	Y	74.55	2004.9177	18	-1.1	669.3099	3	44.62	1	F1:42703	20210611-08_2021021-OK192A_815_5.3.236.mgf	0		0	0	0	9	26			PEAKS DB
total 2 peptides																					

V_1302_039_Conkunitzin-12

back to list

| Protein Coverage | Supporting Peptides |

Protein Coverage:

1 ESIHICDK**AK DEGTGSQNIA SYFFDR**DTGT CETFNYTGGN YDMFPNRFNN CPVCENTCKC KLRKDPGTGE RRRIRYYHSS

81 DGSGCLPFEY RGIGGNANNF DSESACINEC EL

Supporting Peptides:

Peptide	Uniq	-10lgP	Mass	Length	ppm	m/z	z	RT	Fraction	Scan	Source File	Area ebraeus	Area judaeus	#Feature	#Feature ebraeus	#Feature judaeus	Start	End	PTM	AScore	Found By
K.DEGTGSQNIASYFFDR.D	Y	97.30	1805.7856	16	-0.2	903.8965	2	51.83	1	F1:48344	20210611-08_2021021-OK192A_815_5.3.236.mgf	0		0	0	0	11	26			PEAKS DB
K.AKDEGTGSQNIASYFFDR.D	Y	74.55	2004.9177	18	-1.1	669.3099	3	44.62	1	F1:42703	20210611-08_2021021-OK192A_815_5.3.236.mgf	0		0	0	0	9	26			PEAKS DB
total 2 peptides																					

OK213_E_009_B1

back to list

| Protein Coverage | Supporting Peptides |

Protein Coverage:

1 **DGGALTER**R**S ADVTALKPEP VLLQK**SAARS TSDNGKDRLT QMRRIFIKRG NTARGYEEDR EVAETVRELD EHGKR

Supporting Peptides:

Peptide	Uniq	-10lgP	Mass	Length	ppm	m/z	z	RT	Fraction	Scan	Source File	Area ebraeus	Area judaeus	#Feature	#Feature ebraeus	#Feature judaeus	Start	End	PTM	AScore	Found By
R.SADVTALKPEPVLLQK.S	Y	98.74	1707.9771	16	2.5	854.9947	2	37.23	1	F1:34574	20210611-08_2021021-OK192A_815_5.3.236.mgf	0		0	0	0	10	25			PEAKS DB
DGGALTER.R	N	70.06	817.3929	8	1.7	409.7029	2	14.28	1	F1:5430	20210611-08_2021021-OK192A_815_5.3.236.mgf	0	0	0	0	0	1	8			PEAKS DB
total 2 peptides																					

OK213_E_008_B1

back to list

| Protein Coverage | Supporting Peptides |

Protein Coverage:

1 **DGGALTER**R**S ADVTALKPEP VLLQK**SAARS TSDNGKDRLT QMKRTLKKRG NVATDREEIL ETIRELNESG KR

Supporting Peptides:

Peptide	Uniq	-10lgP	Mass	Length	ppm	m/z	z	RT	Fraction	Scan	Source File	Area ebraeus	Area judaeus	#Feature	#Feature ebraeus	#Feature judaeus	Start	End	PTM	AScore	Found By
R.SADVTALKPEPVLLQK.S	Y	98.74	1707.9771	16	2.5	854.9947	2	37.23	1	F1:34574	20210611-08_2021021-OK192A_815_5.3.236.mgf	0		0	0	0	10	25			PEAKS DB
DGGALTER.R	N	70.06	817.3929	8	1.7	409.7029	2	14.28	1	F1:5430	20210611-08_2021021-OK192A_815_5.3.236.mgf	0	0	0	0	0	1	8			PEAKS DB
total 2 peptides																					

SRX1323887_E_010_B1

back to list

| Protein Coverage | Supporting Peptides |

Protein Coverage:

1 **DGGALTER**R**S ADVTALKPEP VLLQK**SAARS TSDNGKDRLT QMRRIFIKRG NTARGYEDDA EDVEYIGDLE EL

Supporting Peptides:

Peptide	Uniq	-10lgP	Mass	Length	ppm	m/z	z	RT	Fraction	Scan	Source File	Area ebraeus	Area judaeus	#Feature	#Feature ebraeus	#Feature judaeus	Start	End	PTM	AScore	Found By
R.SADVTALKPEPVLLQK.S	Y	98.74	1707.9771	16	2.5	854.9947	2	37.23	1	F1:34574	20210611-08_2021021-OK192A_815_5.3.236.mgf	0		0	0	0	10	25			PEAKS DB
DGGALTER.R	N	70.06	817.3929	8	1.7	409.7029	2	14.28	1	F1:5430	20210611-08_2021021-OK192A_815_5.3.236.mgf	0	0	0	0	0	1	8			PEAKS DB
total 2 peptides																					

OK193_E_008_B1

back to list

| Protein Coverage | Supporting Peptides |

Protein Coverage:

1 DGGALTERRS ADVTALKPEP VLLQKSAARS TSDNGKDRLT QMRRIFIKRG NTARGYEDDA EDVEYIGDLE EL

Supporting Peptides:

Peptide	Uniq	-10lgP	Mass	Length	ppm	m/z	z	RT	Fraction	Scan	Source File	Area ebraeus	Area judaeus	#Feature	#Feature ebraeus	#Feature judaeus	Start	End	PTM	AScore	Found By
R.SADVTALKPEPVLLQK.S	Y	98.74	1707.9771	16	2.5	854.9947	2	37.23	1	F1:34574	20210611-08_2021021-OK192A_815_5.3.236.mgf	0		0	0	0	10	25			PEAKS DB
DGGALTER.R	N	70.06	817.3929	8	1.7	409.7029	2	14.28	1	F1:5430	20210611-08_2021021-OK192A_815_5.3.236.mgf	0	0	0	0	0	1	8			PEAKS DB
total 2 peptides																					

OK213_E_010_B1

back to list

| Protein Coverage | Supporting Peptides |

Protein Coverage:

1 DGGALTERRS ADVTALKPEP VLLQKSAARS TSDNGKDRLT QMRRIFIKRG NTARGYEDDA EDVEYIGDLE EL

Supporting Peptides:

Peptide	Uniq	-10lgP	Mass	Length	ppm	m/z	z	RT	Fraction	Scan	Source File	Area ebraeus	Area judaeus	#Feature	#Feature ebraeus	#Feature judaeus	Start	End	PTM	AScore	Found By
R.SADVTALKPEPVLLQK.S	Y	98.74	1707.9771	16	2.5	854.9947	2	37.23	1	F1:34574	20210611-08_2021021-OK192A_815_5.3.236.mgf	0		0	0	0	10	25			PEAKS DB
DGGALTER.R	N	70.06	817.3929	8	1.7	409.7029	2	14.28	1	F1:5430	20210611-08_2021021-OK192A_815_5.3.236.mgf	0	0	0	0	0	1	8			PEAKS DB
total 2 peptides																					

CebrOK014_E_003_B1

back to list

| Protein Coverage | Supporting Peptides |

Protein Coverage:

1 DGGALTERRS ADVTALKPEP VLLQKSAARS TSDNGKDRLT QMRRIFIKRG NTARGYEDDA EDVEYIGDLE EL

Supporting Peptides:

Peptide	Uniq	-10lgP	Mass	Length	ppm	m/z	z	RT	Fraction	Scan	Source File	Area ebraeus	Area judaeus	#Feature	#Feature ebraeus	#Feature judaeus	Start	End	PTM	AScore	Found By
R.SADVTALKPEPVLLQK.S	Y	98.74	1707.9771	16	2.5	854.9947	2	37.23	1	F1:34574	20210611-08_2021021-OK192A_815_5.3.236.mgf	0		0	0	0	10	25			PEAKS DB
DGGALTER.R	N	70.06	817.3929	8	1.7	409.7029	2	14.28	1	F1:5430	20210611-08_2021021-OK192A_815_5.3.236.mgf	0	0	0	0	0	1	8			PEAKS DB
total 2 peptides																					

OK193_E_026_Conkunitzin

back to list

| Protein Coverage | Supporting Peptides |

Protein Coverage:

1 CYEPLDSGDS RGPTQSTRFY YNQEKKCQT FQCTECGGNG NNFPDLYACK ECCEWDMTCY

C Carbamidomethylation (+57.02)

Supporting Peptides:

Peptide	Uniq	-10lgP	Mass	Length	ppm	m/z	z	RT	Fraction	Scan	Source File	Area ebraeus	Area judaeus	#Feature	#Feature ebraeus	#Feature judaeus	Start	End	PTM	AScore	Found By
C(+57.02)YEPLDSGDSR.G	Y	97.67	1297.5244	11	0.9	649.7676	2	22.26	1	F1:14164	20210611-08_2021021-OK192A_815_5.3.236.mgf	0		0	0	0	1	11	Carbamidomethylation	C1:Carbamidomethylation:1000.00	PEAKS DB
R.FYYNQEK.K	Y	61.64	990.4446	7	1.4	496.2284	2	20.07	1	F1:11498	20210611-08_2021021-OK192A_815_5.3.236.mgf	0		0	0	0	19	25			PEAKS DB
total 2 peptides																					

OK006_E_028_Conkunitzin

back to list

| Protein Coverage | Supporting Peptides |

Protein Coverage:

1 CYEPLDSGDS RGPTQSTRFY YNQEKKCQT FQCTECGGNG NNFPDLYACK ECCEWDMTCY

C Carbamidomethylation (+57.02)

Supporting Peptides:

Peptide	Uniq	-10lgP	Mass	Length	ppm	m/z	z	RT	Fraction	Scan	Source File	Area ebraeus	Area judaeus	#Feature	#Feature ebraeus	#Feature judaeus	Start	End	PTM	AScore	Found By
C(+57.02)YEPLDSGDSR.G	Y	97.67	1297.5244	11	0.9	649.7676	2	22.26	1	F1:14164	20210611-08_2021021-OK192A_815_5.3.236.mgf	0		0	0	0	1	11	Carbamidomethylation	C1:Carbamidomethylation:1000.00	PEAKS DB
R.FYYNQEK.K	Y	61.64	990.4446	7	1.4	496.2284	2	20.07	1	F1:11498	20210611-08_2021021-OK192A_815_5.3.236.mgf	0		0	0	0	19	25			PEAKS DB
total 2 peptides																					

SRX1323887_E_026_Conkunitzin

back to list

| Protein Coverage | Supporting Peptides |

Protein Coverage:

1 CYEPLDSGDS RGPTQSTRFY YNQEKKCQT FQCTECGGNG NNFPDLYACK ECCEWDMTCY

C Carbamidomethylation (+57.02)

Supporting Peptides:

Peptide	Uniq	-10lgP	Mass	Length	ppm	m/z	z	RT	Fraction	Scan	Source File	Area ebraeus	Area judaeus	#Feature	#Feature ebraeus	#Feature judaeus	Start	End	PTM	AScore	Found By
C(+57.02)YEPLDSGDSR.G	Y	97.67	1297.5244	11	0.9	649.7676	2	22.26	1	F1:14164	20210611-08_2021021-OK192A_815_5.3.236.mgf	0		0	0	0	1	11	Carbamidomethylation	C1:Carbamidomethylation:1000.00	PEAKS DB
R.FYYNQEK.K	Y	61.64	990.4446	7	1.4	496.2284	2	20.07	1	F1:11498	20210611-08_2021021-OK192A_815_5.3.236.mgf	0		0	0	0	19	25			PEAKS DB
total 2 peptides																					

OK213_E_030_Conkunitzin

back to list

| **Protein Coverage** | **Supporting Peptides** |

Protein Coverage:

```
     1
     C
  1  CYEPLDSGDS RGPTQSTRFY YNQEKKKCQT FQCTECGGNG NNFPDLYACK ECCEWDMTCY
     C
```

C Carbamidomethylation (+57.02)

Supporting Peptides:

Peptide	Uniq	-10lgP	Mass	Length	ppm	m/z	z	RT	Fraction	Scan	Source File	Area ebraeus	Area judaeus	#Feature	#Feature ebraeus	#Feature judaeus	Start	End	PTM	AScore	Found By
C(+57.02)YEPLDSGDSR.G	Y	97.67	1297.5244	11	0.9	649.7676	2	22.26	1	F1:14164	20210611-08_2021021-OK192A_815_5.3.236.mgf	0		0	0	0	1	11	Carbamidomethylation	C1:Carbamidomethylation:1000.00	PEAKS DB
R.FYYNQEK.K	Y	61.64	990.4446	7	1.4	496.2284	2	20.07	1	F1:11498	20210611-08_2021021-OK192A_815_5.3.236.mgf	0		0	0	0	19	25			PEAKS DB
**total 2 peptides**																					

gene:107288180

back to list

| **Protein Coverage** | **Supporting Peptides** |

Protein Coverage:

```
  1  MMRKNYRNTS STVRSFSSRS SGPVASSRIS TSYSSAGSGF GGSRYGGGAS FRGTSAGSLG GITAVSVNKS LLAPLNLEID
 81  PAIQQVRIKE KEEIKTLNNK FANLIDKVRF LEQQNKMLET KWSLLQNQKT TRSNMDSMFE AYINNLRRQL ENLGQERARL
161  DAELGNMQGL VEEFKNKYED EINHRTEKEN EFVLLKKDVD EAYMNKVELE SRMECLTDEI NFLRQFYDEE LRVLQSQISD
241  TSVILSMDNN RSLDLDGIIT DVKAQYEEIA NRSRAEAERM YQIKYEELQT VAGKHGDDLR STKNEINELS RMINRIQSEI
321  DGLKGQRASL EAAITEAEER GELAVKDANN KLAELESAMQ RAKQDMARQL REYQELLNTK LALDIEIATY RKLLEGEEIR
401  LESGMQNLSI QTKTTGYSGG LSSGFSGYGG GYNSSYSSGG YSMNMLPMES SAISKTKAIV IKKIETRDGK LVSESSDVLS
481  K
```

Supporting Peptides:

Peptide	Uniq	-10lgP	Mass	Length	ppm	m/z	z	RT	Fraction	Scan	Source File	Area ebraeus	Area judaeus	#Feature	#Feature ebraeus	#Feature judaeus	Start	End	PTM	AScore	Found By
K.LALDIEIATYR.K	Y	89.70	1276.7026	11	1.5	639.3571	2	48.56	1	F1:46398	20210611-08_2021021-OK192A_815_5.3.236.mgf	0	0	0	0	0	381	391			PEAKS DB
K.AQYEEIANR.S	Y	77.22	1092.5199	9	4.7	547.2675	2	19.94	2	F2:65984	20210611-09_2021021-OK160A_816_5.3.236.mgf	0	0	0	0	0	264	272			PEAKS DB
**total 2 peptides**																					

OK212_J_025_Conkunitzin

back to list

| **Protein Coverage** | **Supporting Peptides** |

Protein Coverage:

```
                     15
                     C
  1  SLPAVCLQPV VVGPCAASLQ RYYFNHHYDI CQPFVYGGCN GNGNRFITMA ACVNTCKSPS VAIVVD
                     C
```

C Carbamidomethylation (+57.02)

Supporting Peptides:

Peptide	Uniq	-10lgP	Mass	Length	ppm	m/z	z	RT	Fraction	Scan	Source File	Area ebraeus	Area judaeus	#Feature	#Feature ebraeus	#Feature judaeus	Start	End	PTM	ASc
SLPAVC(+57.02)LQPVVVGPC(+57.02)AASLQR.Y	Y	127.65	2221.1711	21	12.2	1111.6017	2	49.19	2	F2:117561	20210611-09_2021021-OK160A_816_5.3.236.mgf		0	0	0	0	1	21	Carbamidomethylation	C6:(bam met atio 000 C15 rbar ome latic 000
**total 1 peptides**																				

OK212_J_026_Conkunitzin

back to list

| **Protein Coverage** | **Supporting Peptides** |

Protein Coverage:

```
                     15
                     C
  1  SLPAVCLQPV VVGPCAASLQ RYYFNHHFDI CQPFVYGGCN GNGNRFITMA ACVNTCKSPS VAIVVD
                     C
```

C Carbamidomethylation (+57.02)

Supporting Peptides:

Peptide	Uniq	-10lgP	Mass	Length	ppm	m/z	z	RT	Fraction	Scan	Source File	Area ebraeus	Area judaeus	#Feature	#Feature ebraeus	#Feature judaeus	Start	End	PTM	ASc
SLPAVC(+57.02)LQPVVVGPC(+57.02)AASLQR.Y	Y	127.65	2221.1711	21	12.2	1111.6017	2	49.19	2	F2:117561	20210611-09_2021021-OK160A_816_5.3.236.mgf		0	0	0	0	1	21	Carbamidomethylation	C6:(bam met atio 000 C15 rbar ome latic 000
**total 1 peptides**																				

gene:107288688

back to list

| **Protein Coverage** | **Supporting Peptides** |

Protein Coverage:

```
  1  MAAIRKKLVI VGDGACGKTC LLIVFSKDQF PEVYVPTVFE NYVADIEVDG KQVELALWDT AGQEDYDRLR PLSYPDTDVI
                                                            134
                                                            O
 81  LMCFSIDSPD SLENIPEKWT PEVKHFCPNV PIILVGNKKD LRNDEHTRRE LAKMKQEPVK PEEGRDMANR IGAFGYMECS
                                                            O
161  AKTKDGVREV FEMATRAALQ ARRGKKKSGC LLL
```

O Oxidation (M) (+15.99)

Supporting Peptides:

Peptide	Uniq	-10lgP	Mass	Length	ppm	m/z	z	RT	Fraction	Scan	Source File	Area ebraeus	Area judaeus	#Feature	#Feature ebraeus	#Feature judaeus	Start	End	PTM	AScore	Found By
K.M(+15.99)KQEPVKPEEGR.D	Y	85.52	1442.7188	12	1.1	481.9120	3	9.69	2	F2:52973	20210611-09_2021021-OK160A_816_5.3.236.mgf		0	0	0	0	134	145	Oxidation (M)	M1:Oxidation (M):1000.00	PEAKS DB
K.QVELALWDTAGQEDYDR.L	Y	75.66	2007.9174	17	3.0	1004.9647	2	47.26	2	F2:114052	20210611-09_2021021-OK160A_816_5.3.236.mgf		0	0	0	0	52	68			PEAKS DB
**total 2 peptides**																					

Pvau2_22

back to list

| **Protein Coverage** | **Supporting Peptides** |

Protein Coverage:

1 NLTAMHWTNT LAGYCPKPYS QFCRKLSAFL TKNMQWMKEQ IRHDKGGNLH LPYWHHVELF LYQAAGLADG LSGNEKSPNL
81 DIDPMGFFLF QVGGDIEDLE SVLGDSSTQH PLGAGHCSAL VKLLPGNKDL YVSQDTWDVF PSMLRILKVY DFPWKTLASE
161 TETMAGSVQA FSSYPGTIMS GDDYYLMSSG MVSMETTIGN SNSSLWK**FVT PSSVLEGIR**C IVANRLAKSG AEWASIFSQY
241 NSGTYNNEWM LVDYKRFQPG MSKLPPGVLT VLDQIPGMIQ WADLTELLVN QGYFPSYNVA YFPEIFNASG NQPLVEK**YGN**
321 **WFTYDK**TPRA LIYKRDHVKV KDLNSMMKLM RYNDFQHDPL SRCNCTPPYS GENAISARSD LNPKDGTYPF SSLGHRMHGG
401 IDMKLTSSEL FKGLNFVAVG GPTWDQQPPF QWSKSDFSNV SHIGQPDLWK FSPILFNATQ IFGRP

Supporting Peptides:

Peptide	Uniq	-10lgP	Mass	Length	ppm	m/z	z	RT	Fraction	Scan	Source File	Area ebraeus	Area judaeus	#Feature	#Feature ebraeus	#Feature judaeus	Start	End	PTM	AScore	Found By
K.FVTPSSVLEGIR.C	Y	79.82	1303.7136	12	-2.0	652.8600	2	47.38	2	F2:114202	20210611-09_2021021-OK160A_816_5.3.236.mgf		0	0	0	0	208	219			PEAKS DB
K.YGNWFTYDK.T	Y	65.79	1192.5189	9	-1.8	597.2632	2	38.54	2	F2:98999	20210611-09_2021021-OK160A_816_5.3.236.mgf		0	0	0	0	318	326			PEAKS DB
**total 2 peptides**																					

gene:107294617

back to list

| Protein Coverage | Supporting Peptides |

Protein Coverage:

1 MKQLWGLGLL CLLLLAASAQ AEEPDVDGTV EDDLGKSREG SRTDDEVVQR EEESIQLDGL NASQIKQLRE KSERFAFQAE
81 VNRMMKLIIN SLYKNKEIFL RELISNASDA LDKIRLMSLT DETALSDNEE LTIKIKCDKE KNMLHVIDTG IGMTKDELIK
161 NLGTIAKSGT SEFLNKISEM QEENQSTSEL IGQFGVGFYS AFLVADKVIV TSKHNNDSQQ IWESDSNEFS VIEDPRGNTL
241 GRGTTVTLVL KEEASDYLEL DTVKNLVTKY SQFINFPIYV WSSKTETVEE PLEDEEVKDK DETDDEAAVE EEEEEKKPKT
321 KPVQKTVWDW ELMNDIKPIW QRPSKEVEED EYKAFYKSFS KETDEPMTYI HFTAEGEVTF KSILFIPSTA PRGLFDEYGS
401 KKSDFIKLYV RRVFITDDFH DMMPKYLNFV K**GVVDSDDLP LNVSR**ETLQQ HKLLKVIRKK LVRKTLDMIK KIAEEKYNDT
481 FWKEFGTNIK LGVIEDHSNR TRLAKLLRFQ SSNHESNTTS LDQYVERMKE KQDKIYFMAG SSRKEAESSP FVERLLKKGY
561 EVIYLTEPVD EYCIQALPEF DNKRFQNVAK EGVKFDESEK SKEAHEALEK EYEPLLTWMK DKALKDKIEK AVLSQRLTQS
641 PCALVASQYG WSGNMERIMK AQAYQTGKDI ATNYYASQKK IFEINPKHPL IKDMLRRVQE NEDDQIVADL AVVLLETATL
721 RSGYLLPDTK EYGERIERML RLSLNIDPEE KVEDEPEEPE EADEEVEPEE EIEADEDIED SETEKTESTD GKDEL

Supporting Peptides:

Peptide	Uniq	-10lgP	Mass	Length	ppm	m/z	z	RT	Fraction	Scan	Source File	Area ebraeus	Area judaeus	#Feature	#Feature ebraeus	#Feature judaeus	Start	End	PTM	AScore	Found By
K.GVVDSDDLPLNVSR.E	Y	111.81	1484.7471	14	0.6	743.3784	2	39.08	1	F1:36637	20210611-08_2021021-OK192A_815_5.3.236.mgf	0	0	0	0	0	432	445			PEAKS DB
**total 1 peptides**																					

P_027D_029_Neuroendocrine

back to list

| Protein Coverage | Supporting Peptides |

Protein Coverage:

1 MVKKYVVVTV VVIAAALLLR VDPADGKEDE SRYLNEWAVE IEGGLNHARS VAERHGFELL GQMPFLDNHF IVRHVDVPMR
81 SKRSADQHTT RLTEDSEVKW AEQQVVRARS KRGYADNSPP LLLPMAPEET KTCEANTRLR YNDPDYPCQW YMVGDDEHEY
161 TSNVIKVWKQ KITGRGVVVC VVDDGLEHNH TDLFPNYDPY ASWDLNSNDS DPMPHYNEME TNRHGTRCAG EISMAADNGK
241 CGVGIAFNSK IGGIRLLDGN VTDRLEADAL SFNHTHIDIY SLSWGPTDDG KTVEKPLRLV QTAMETAIQK GRGGKGVIYT
321 LASGNGGGSN DDCNCDGYAS SLYTISVSSV GRDGNKTSYG ECCASSLTTT YSSGTRGGVS VVSTDLHNEC TYHHSGTSAS
401 APMASGFIAL ALEANPDLTW RDVQHLVVIT SRHDQLTKKG LYKNAANFCV NPAFGFGLMD PLGMVKWAKI WKRVPKQRVC
481 HSTPIVGTTS RANHWSRDVR SGSGVKVTFD VHNDCCPNNR TAINYLEHVQ FIFTMNYTTR GAISAYATSP SGTRTVLLSP
561 RTRDNSTENF EDWPLMSVHT WGENPCGEWT FQVADEGSFG TNNSGSLVDV K**MILYGTNTA PFYR**KYSTAY CALPTPSSTQ
641 EVQTTHKTGD FIPHYGMGLL KYLWFRDTDE TNKSSE

Supporting Peptides:

Peptide	Uniq	-10lgP	Mass	Length	ppm	m/z	z	RT	Fraction	Scan	Source File	Area ebraeus	Area judaeus	#Feature	#Feature ebraeus	#Feature judaeus	Start	End	PTM	AScore	Found By
K.MILYGTNTAPFYR.K	Y	111.72	1545.7650	13	1.5	773.8877	2	43.58	2	F2:107764	20210611-09_2021021-OK160A_816_5.3.236.mgf		0	0	0	0	612	624			PEAKS DB
K.M(+15.99)ILYGTNTAPFYR.K	Y	98.71	1561.7599	13	-1.7	781.8826	2	38.89	2	F2:99620	20210611-09_2021021-OK160A_816_5.3.236.mgf		0	0	0	0	612	624		M1:Oxidation (M):1000.00	PEAKS DB
**total 2 peptides**																					

OK193_E_214_Ggeo01

back to list

| Protein Coverage | Supporting Peptides |

Protein Coverage:

1 SQTDDSSTEK RSRSMPDVDF NLQFDK**IGDS SDFGQEFPK**G K

Supporting Peptides:

Peptide	Uniq	-10lgP	Mass	Length	ppm	m/z	z	RT	Fraction	Scan	Source File	Area ebraeus	Area judaeus	#Feature	#Feature ebraeus	#Feature judaeus	Start	End	PTM	AScore	Found By
K.IGDSSDFGQEFPK.G	Y	108.40	1425.6411	13	2.1	713.8267	2	34.76	1	F1:31556	20210611-08_2021021-OK192A_815_5.3.236.mgf	0		0	0	0	27	39			PEAKS DB
**total 1 peptides**																					

V_CG13_188_D

back to list

| Protein Coverage | Supporting Peptides |

Protein Coverage:

6 C

1 **AVIAACHPNN PGSPWGR**CCT TVMCGSVCCN YAHCSCIYHK DRGHGCDC

C Carbamidomethylation (+57.02)

Supporting Peptides:

Peptide	Uniq	-10lgP	Mass	Length	ppm	m/z	z	RT	Fraction	Scan	Source File	Area ebraeus	Area judaeus	#Feature	#Feature ebraeus	#Feature judaeus	Start	End	PTM	AScore	Found By
AVIAAC(+57.02)HPNNPGSPWGR.C	Y	105.56	1802.8634	17	0.5	601.9598	3	28.41	1	F1:22979	20210611-08_2021021-OK192A_815_5.3.236.mgf	0		0	0	0	1	17	Carbamidomethylation	C6:Carbamidomethylation:1000.00	PEAKS DB
**total 1 peptides**																					

OK193_E_115_M

back to list

| Protein Coverage | Supporting Peptides |

Protein Coverage:

17

1 CVYSTPCGEC TRRGKICSVQ PGGKGTCGEC VP

C Carbamidomethylation (+57.02)

Supporting Peptides:

Peptide	Uniq	-10lgP	Mass	Length	ppm	m/z	z	RT	Fraction	Scan	Source File	Area ebraeus	Area judaeus	#Feature	#Feature ebraeus	#Feature judaeus	Start	End	PTM	AScore	Found By
R.GKIC(+57.02)SVQPGGK.G	Y	72.55	1129.5913	11	-0.1	565.8007	2	12.34	1	F1:3510	20210611-08_2021021-OK192A_815_5.3.236.mgf	0		0	0	0	14	24		C4:Carbamidomethylation:1000.00	PEAKS DB
K.IC(+57.02)SVQPGGK.G	Y	65.62	944.4749	9	6.0	473.2458	2	13.92	1	F1:5172	20210611-08_2021021-OK192A_815_5.3.236.mgf	0		0	0	0	16	24	Carbamidomethylation	C2:Carbamidomethylation:1000.00	PEAKS DB
total 2 peptides																					

V_CG13_073_Rimp

back to list

| Protein Coverage | Supporting Peptides |

Protein Coverage:

1 AGIYYPPVEK PIGPCETNDI SKTEMYSNRF GRWESPYIYC TCAGGEEHFA ANDTITSYAT VSYRIYVCGA PTSPCSGAMI

81 PVTDSNDGTR RMQCTCGQYK YFVSNPLGWH VRC

Supporting Peptides:

Peptide	Uniq	-10lgP	Mass	Length	ppm	m/z	z	RT	Fraction	Scan	Source File	Area ebraeus	Area judaeus	#Feature	#Feature ebraeus	#Feature judaeus	Start	End	PTM	AScore	Found By
K.YFVSNPLGWHVR.C	Y	104.60	1473.7517	12	0.9	737.8810	2	43.26	1	F1:41157	20210611-08_2021021-OK192A_815_5.3.236.mgf	0	0	0	0	0	101	112			PEAKS DB
total 1 peptides																					

OK213_E_049_H

back to list

| Protein Coverage | Supporting Peptides |

Protein Coverage:

1 HPIANDGDAT RNVGSDGISV ELPKMIERGQ DSGQEKTSAH GRGIVYVYYA PDHDIPFPH

Supporting Peptides:

Peptide	Uniq	-10lgP	Mass	Length	ppm	m/z	z	RT	Fraction	Scan	Source File	Area ebraeus	Area judaeus	#Feature	#Feature ebraeus	#Feature judaeus	Start	End	PTM	AScore	Found By
R.GIVYVYYAPDHDIPFPH	Y	103.98	2001.9624	17	4.5	1001.9892	2	49.85	1	F1:47334	20210611-08_2021021-OK192A_815_5.3.236.mgf	0		0	0	0	43	59			PEAKS DB
total 1 peptides																					

A_0025_010_60S

back to list

| Protein Coverage | Supporting Peptides |

Protein Coverage:

1 AFHLRVRLHP FHIIRINKML SCAGADRLQT GMRGAFGKPQ GTVARVHIGQ PIMSVRAREQ HEASIIEALR RAKFKYPGRQ

81 KIVVSKKWGF TKWAREDYER MRAEGYL

Supporting Peptides:

Peptide	Uniq	-10lgP	Mass	Length	ppm	m/z	z	RT	Fraction	Scan	Source File	Area ebraeus	Area judaeus	#Feature	#Feature ebraeus	#Feature judaeus	Start	End	PTM	AScore	Found By
R.EQHEASIIEALR.R	Y	69.77	1394.7153	12	0.5	465.9107	3	34.28	2	F2:91188	20210611-09_2021021-OK160A_816_5.3.236.mgf		0	0	0	0	59	70			PEAKS DB
R.GAFGKPQGTVAR.V	Y	66.31	1187.6411	12	4.3	396.8877	3	16.39	2	F2:60763	20210611-09_2021021-OK160A_816_5.3.236.mgf		0	0	0	0	34	45			PEAKS DB
total 2 peptides																					

A_1387_362

back to list

| Protein Coverage | Supporting Peptides |

Protein Coverage:

1 AFHLRVRLHP FHIIRINKML SCAGADRLQT GMRGAFGKPQ GTVARVHIGQ PIMSVRAREQ HEASIIEALR RAKFKYPGRQ

81 KIVVSKKWGF TKWAREDYER MRAEGYL

Supporting Peptides:

Peptide	Uniq	-10lgP	Mass	Length	ppm	m/z	z	RT	Fraction	Scan	Source File	Area ebraeus	Area judaeus	#Feature	#Feature ebraeus	#Feature judaeus	Start	End	PTM	AScore	Found By
R.EQHEASIIEALR.R	Y	69.77	1394.7153	12	0.5	465.9107	3	34.28	2	F2:91188	20210611-09_2021021-OK160A_816_5.3.236.mgf		0	0	0	0	59	70			PEAKS DB
R.GAFGKPQGTVAR.V	Y	66.31	1187.6411	12	4.3	396.8877	3	16.39	2	F2:60763	20210611-09_2021021-OK160A_816_5.3.236.mgf		0	0	0	0	34	45			PEAKS DB
total 2 peptides																					

A_0885_182_60S

back to list

| Protein Coverage | Supporting Peptides |

Protein Coverage:

1 AFHLRVRLHP FHIIRINKML SCAGADRLQT GMRGAFGKPQ GTVARVHIGQ PIMSVRAREQ HEASIIEALR RAKFKYPGRQ

81 KIVVSKKWGF TKWAREDYER MRAEGYL

Supporting Peptides:

Peptide	Uniq	-10lgP	Mass	Length	ppm	m/z	z	RT	Fraction	Scan	Source File	Area ebraeus	Area judaeus	#Feature	#Feature ebraeus	#Feature judaeus	Start	End	PTM	AScore	Found By
R.EQHEASIIEALR.R	Y	69.77	1394.7153	12	0.5	465.9107	3	34.28	2	F2:91188	20210611-09_2021021-OK160A_816_5.3.236.mgf		0	0	0	0	59	70			PEAKS DB
R.GAFGKPQGTVAR.V	Y	66.31	1187.6411	12	4.3	396.8877	3	16.39	2	F2:60763	20210611-09_2021021-OK160A_816_5.3.236.mgf		0	0	0	0	34	45			PEAKS DB
total 2 peptides																					

A_0055_259_60S

back to list

| Protein Coverage | Supporting Peptides |

Protein Coverage:

1 AFHLRVRLHP FHIIRINKML SCAGADRLQT GMRGAFGKPQ GTVARVHIGQ PIMSVRAREQ HEASIIEALR RAKFKYPGRQ

81 KIVVSKKWGF TKWAREDYER MRAEGYL

Supporting Peptides:

Peptide	Uniq	-10lgP	Mass	Length	ppm	m/z	z	RT	Fraction	Scan	Source File	Area ebraeus	Area judaeus	#Feature	#Feature ebraeus	#Feature judaeus	Start	End	PTM	AScore	Found By
R.EQHEASIIEALR.R	Y	69.77	1394.7153	12	0.5	465.9107	3	34.28	2	F2:91188	20210611-09_2021021-OK160A_816_5.3.236.mgf		0	0	0	0	59	70			PEAKS DB

total 2 peptides

Peptide	Uniq	-10lgP	Mass	Length	ppm	m/z	z	RT	Fraction	Scan	Source File	Area ebraeus	Area judaeus	#Feature	#Feature ebraeus	#Feature judaeus	Start	End	PTM	AScore	Found By
R.GAFGKPQGTVAR.V	Y	66.31	1187.6411	12	4.3	396.8877	3	16.39	2	F2:60763	20210611-09_2021021-OK160A_816_5.3.236.mgf		0	0	0	0	34	45			PEAKS DB
**total 2 peptides**																					

A_0520_023_60S

back to list

| Protein Coverage | Supporting Peptides |

Protein Coverage:

1 AFHLRVRLHP FHIIRINKML SCAGADRLQT GMR**GAFGKPQ GTVAR**VHIGQ PIMSVRAR**EQ HEASIIEALR** RAKFKYPGRQ

81 KIVVSKKWGF TKWAREDYER MRAEGYL

Supporting Peptides:

Peptide	Uniq	-10lgP	Mass	Length	ppm	m/z	z	RT	Fraction	Scan	Source File	Area ebraeus	Area judaeus	#Feature	#Feature ebraeus	#Feature judaeus	Start	End	PTM	AScore	Found By
R.EQHEASIIEALR.R	Y	69.77	1394.7153	12	0.5	465.9107	3	34.28	2	F2:91188	20210611-09_2021021-OK160A_816_5.3.236.mgf		0	0	0	0	59	70			PEAKS DB
R.GAFGKPQGTVAR.V	Y	66.31	1187.6411	12	4.3	396.8877	3	16.39	2	F2:60763	20210611-09_2021021-OK160A_816_5.3.236.mgf		0	0	0	0	34	45			PEAKS DB
**total 2 peptides**																					

A_0048_318_60S

back to list

| Protein Coverage | Supporting Peptides |

Protein Coverage:

1 AFHLRVRLHP FHIIRINKML SCAGADRLQT GMR**GAFGKPQ GTVAR**VHIGQ PIMSVRAR**EQ HEASIIEALR** RAKFKYPGRQ

81 KIVVSKKWGF TKWAREDYER MRAEGYL

Supporting Peptides:

Peptide	Uniq	-10lgP	Mass	Length	ppm	m/z	z	RT	Fraction	Scan	Source File	Area ebraeus	Area judaeus	#Feature	#Feature ebraeus	#Feature judaeus	Start	End	PTM	AScore	Found By
R.EQHEASIIEALR.R	Y	69.77	1394.7153	12	0.5	465.9107	3	34.28	2	F2:91188	20210611-09_2021021-OK160A_816_5.3.236.mgf		0	0	0	0	59	70			PEAKS DB
R.GAFGKPQGTVAR.V	Y	66.31	1187.6411	12	4.3	396.8877	3	16.39	2	F2:60763	20210611-09_2021021-OK160A_816_5.3.236.mgf		0	0	0	0	34	45			PEAKS DB
**total 2 peptides**																					

A_0031_033_60S

back to list

| Protein Coverage | Supporting Peptides |

Protein Coverage:

1 AFHLRVRLHP FHIIRINKML SCAGADRLQT GMR**GAFGKPQ GTVAR**VHIGQ PIMSVRAR**EQ HEASIIEALR** RAKFKYPGRQ

81 KIVVSKKWGF TKWAREDYER MRAEGYL

Supporting Peptides:

Peptide	Uniq	-10lgP	Mass	Length	ppm	m/z	z	RT	Fraction	Scan	Source File	Area ebraeus	Area judaeus	#Feature	#Feature ebraeus	#Feature judaeus	Start	End	PTM	AScore	Found By
R.EQHEASIIEALR.R	Y	69.77	1394.7153	12	0.5	465.9107	3	34.28	2	F2:91188	20210611-09_2021021-OK160A_816_5.3.236.mgf		0	0	0	0	59	70			PEAKS DB
R.GAFGKPQGTVAR.V	Y	66.31	1187.6411	12	4.3	396.8877	3	16.39	2	F2:60763	20210611-09_2021021-OK160A_816_5.3.236.mgf		0	0	0	0	34	45			PEAKS DB
**total 2 peptides**																					

A_0855_173_60S

back to list

| Protein Coverage | Supporting Peptides |

Protein Coverage:

1 AFHLRVRLHP FHIIRINKML SCAGADRLQT GMR**GAFGKPQ GTVAR**VHIGQ PIMSVRAR**EQ HEASIIEALR** RAKFKYPGRQ

81 KIVVSKKWGF TKWAREDYER MRAEGYL

Supporting Peptides:

Peptide	Uniq	-10lgP	Mass	Length	ppm	m/z	z	RT	Fraction	Scan	Source File	Area ebraeus	Area judaeus	#Feature	#Feature ebraeus	#Feature judaeus	Start	End	PTM	AScore	Found By
R.EQHEASIIEALR.R	Y	69.77	1394.7153	12	0.5	465.9107	3	34.28	2	F2:91188	20210611-09_2021021-OK160A_816_5.3.236.mgf		0	0	0	0	59	70			PEAKS DB
R.GAFGKPQGTVAR.V	Y	66.31	1187.6411	12	4.3	396.8877	3	16.39	2	F2:60763	20210611-09_2021021-OK160A_816_5.3.236.mgf		0	0	0	0	34	45			PEAKS DB
**total 2 peptides**																					

K_0010_065_60S

back to list

| Protein Coverage | Supporting Peptides |

Protein Coverage:

1 AFHLRVRLHP FHIIRINKML SCAGADRLQT GMR**GAFGKPQ GTVAR**VHIGQ PIMSVRAR**EQ HEASIIEALR** RAKFKYPGRQ

81 KIVVSKKWGF TKWPREDYER MRAEGYL

Supporting Peptides:

Peptide	Uniq	-10lgP	Mass	Length	ppm	m/z	z	RT	Fraction	Scan	Source File	Area ebraeus	Area judaeus	#Feature	#Feature ebraeus	#Feature judaeus	Start	End	PTM	AScore	Found By
R.EQHEASIIEALR.R	Y	69.77	1394.7153	12	0.5	465.9107	3	34.28	2	F2:91188	20210611-09_2021021-OK160A_816_5.3.236.mgf		0	0	0	0	59	70			PEAKS DB
R.GAFGKPQGTVAR.V	Y	66.31	1187.6411	12	4.3	396.8877	3	16.39	2	F2:60763	20210611-09_2021021-OK160A_816_5.3.236.mgf		0	0	0	0	34	45			PEAKS DB
**total 2 peptides**																					

A_0239_021_60S

back to list

| Protein Coverage | Supporting Peptides |

Protein Coverage:

1 AFHLRVRLHP FHIIRINKML SCAGADRLQT GMR**GAFGKPQ GTVAR**VHIGQ PIMSVRAR**EQ HEASIIEALR** RAKFKYPGRQ

81 KIVVSKKWGF TKWAREDYER MRAEGYL

Supporting Peptides:

Peptide	Uniq	-10lgP	Mass	Length	ppm	m/z	z	RT	Fraction	Scan	Source File	Area ebraeus	Area judaeus	#Feature	#Feature ebraeus	#Feature judaeus	Start	End	PTM	AScore	Found By
R.EQHEASIIEALR.R	Y	69.77	1394.7153	12	0.5	465.9107	3	34.28	2	F2:91188	20210611-09_2021021-OK160A_816_5.3.236.mgf		0	0	0	0	59	70			PEAKS DB
R.GAFGKPQGTVAR.V	Y	66.31	1187.6411	12	4.3	396.8877	3	16.39	2	F2:60763	20210611-09_2021021-OK160A_816_5.3.236.mgf		0	0	0	0	34	45			PEAKS DB
**total 2 peptides**																					

A_0875_147_60S

back to list

| Protein Coverage | Supporting Peptides |

Protein Coverage:

1 AFHLRVRLHP FHIIRINKML SCAGADRLQT GMR**GAFGKPQ GTVAR**VHIGQ PIMSVRAR**EQ HEASIIEALR** RAKFKYPGRQ

81 KIVVSKKWGF TKWAREDYER MRAEGYL

Supporting Peptides:

Peptide	Uniq	-10lgP	Mass	Length	ppm	m/z	z	RT	Fraction	Scan	Source File	Area ebraeus	Area judaeus	#Feature	#Feature ebraeus	#Feature judaeus	Start	End	PTM	AScore	Found By
R.EQHEASIIEALR.R	Y	69.77	1394.7153	12	0.5	465.9107	3	34.28	2	F2:91188	20210611-09_2021021-OK160A_816_5.3.236.mgf		0	0	0	0	59	70			PEAKS DB
R.GAFGKPQGTVAR.V	Y	66.31	1187.6411	12	4.3	396.8877	3	16.39	2	F2:60763	20210611-09_2021021-OK160A_816_5.3.236.mgf		0	0	0	0	34	45			PEAKS DB
total 2 peptides																					

A_0039_214_60S

back to list

| Protein Coverage | Supporting Peptides |

Protein Coverage:

1 AFHLRVRLHP FHIIRINKML SCAGADRLQT GMR**GAFGKPQ GTVAR**VHIGQ PIMSVRAR**EQ HEASIIEALR** RAKFKYPGRQ

81 KIVVSKKWGF TKWAREDYER MRAEGYL

Supporting Peptides:

Peptide	Uniq	-10lgP	Mass	Length	ppm	m/z	z	RT	Fraction	Scan	Source File	Area ebraeus	Area judaeus	#Feature	#Feature ebraeus	#Feature judaeus	Start	End	PTM	AScore	Found By
R.EQHEASIIEALR.R	Y	69.77	1394.7153	12	0.5	465.9107	3	34.28	2	F2:91188	20210611-09_2021021-OK160A_816_5.3.236.mgf		0	0	0	0	59	70			PEAKS DB
R.GAFGKPQGTVAR.V	Y	66.31	1187.6411	12	4.3	396.8877	3	16.39	2	F2:60763	20210611-09_2021021-OK160A_816_5.3.236.mgf		0	0	0	0	34	45			PEAKS DB
total 2 peptides																					

OK212_J_022_Con-ikot-ikot

back to list

| Protein Coverage | Supporting Peptides |

Protein Coverage:

1 **LECPPPSHGQ NR**DRSSRKCC AVKSYQCLRD R**GCFNPVNSK** CAEPCKTTDT TSCGSTTGDN CCSIYKSCLV ECQIAHGDQR — C Carbamidomethylation (+57.02)

81 EDQLLCCYNI CDQSYPC

Supporting Peptides:

Peptide	Uniq	-10lgP	Mass	Length	ppm	m/z	z	RT	Fraction	Scan	Source File	Area ebraeus	Area judaeus	#Feature	#Feature ebraeus	#Feature judaeus	Start	End	PTM	AScore	Found By
R.GC(+57.02)FNPVNSK.C	Y	70.61	1021.4651	9	5.1	511.7403	2	20.01	2	F2:66038	20210611-09_2021021-OK160A_816_5.3.236.mgf		0	0	0	0	32	40	Carbamidomethylation	C2:Carbamidomethylation:1000.00	PEAKS DB
LEC(+57.02)PPPSHGQNR.D	Y	63.83	1390.6411	12	0.4	464.5526	3	11.89	2	F2:54854	20210611-09_2021021-OK160A_816_5.3.236.mgf		0	0	0	0	1	12	Carbamidomethylation	C3:Carbamidomethylation:1000.00	PEAKS DB
total 2 peptides																					

OK214_J_017_Con-ikot-ikot

back to list

| Protein Coverage | Supporting Peptides |

Protein Coverage:

1 **LECPPPSHGQ NR**DRSSRKCC AVKSYQCLRD R**GCFNPVNSK** CAEPCKTTDT TSCGSTTGDN CCSIYKSCLV ECQIAHGDQR — C Carbamidomethylation (+57.02)

81 EDQLLCCYNI CDQSYPC

Supporting Peptides:

Peptide	Uniq	-10lgP	Mass	Length	ppm	m/z	z	RT	Fraction	Scan	Source File	Area ebraeus	Area judaeus	#Feature	#Feature ebraeus	#Feature judaeus	Start	End	PTM	AScore	Found By
R.GC(+57.02)FNPVNSK.C	Y	70.61	1021.4651	9	5.1	511.7403	2	20.01	2	F2:66038	20210611-09_2021021-OK160A_816_5.3.236.mgf		0	0	0	0	32	40	Carbamidomethylation	C2:Carbamidomethylation:1000.00	PEAKS DB
LEC(+57.02)PPPSHGQNR.D	Y	63.83	1390.6411	12	0.4	464.5526	3	11.89	2	F2:54854	20210611-09_2021021-OK160A_816_5.3.236.mgf		0	0	0	0	1	12	Carbamidomethylation	C3:Carbamidomethylation:1000.00	PEAKS DB
total 2 peptides																					

CebrOK035_J_031_Con-ikot-ikot

back to list

| Protein Coverage | Supporting Peptides |

Protein Coverage:

1 **LECPPPSHGQ NR**DRSSRKCC AVKSYQCLRD R**GCFNPVNSK** CAEPCKTTDT TSCGSTTGDN CCSIYKSCLV ECQIAHGDQR — C Carbamidomethylation (+57.02)

81 EDQLLCCYNI CDQSYPC

Supporting Peptides:

Peptide	Uniq	-10lgP	Mass	Length	ppm	m/z	z	RT	Fraction	Scan	Source File	Area ebraeus	Area judaeus	#Feature	#Feature ebraeus	#Feature judaeus	Start	End	PTM	AScore	Found By
R.GC(+57.02)FNPVNSK.C	Y	70.61	1021.4651	9	5.1	511.7403	2	20.01	2	F2:66038	20210611-09_2021021-OK160A_816_5.3.236.mgf		0	0	0	0	32	40	Carbamidomethylation	C2:Carbamidomethylation:1000.00	PEAKS DB
LEC(+57.02)PPPSHGQNR.D	Y	63.83	1390.6411	12	0.4	464.5526	3	11.89	2	F2:54854	20210611-09_2021021-OK160A_816_5.3.236.mgf		0	0	0	0	1	12	Carbamidomethylation	C3:Carbamidomethylation:1000.00	PEAKS DB
total 2 peptides																					

OK214_J_018_Con-ikot-ikot

back to list

| Protein Coverage | Supporting Peptides |

Protein Coverage:

1 **LECPPPSHGQ NR**DRSSRKCC AVKSYQCLRD R**GCFNPVNSK** CAEPCKTTDT TSCGSTTGDN CCSIYKSCLV ECQIAHGDQR — C Carbamidomethylation (+57.02)

81 EDQLLCCYNI CDQSCTYT

Supporting Peptides:

Peptide	Uniq	-10lgP	Mass	Length	ppm	m/z	z	RT	Fraction	Scan	Source File	Area ebraeus	Area judaeus	#Feature	#Feature ebraeus	#Feature judaeus	Start	End	PTM	AScore	Found By
R.GC(+57.02)FNPVNSK.C	Y	70.61	1021.4651	9	5.1	511.7403	2	20.01	2	F2:66038	20210611-09_2021021-OK160A_816_5.3.236.mgf		0	0	0	0	32	40	Carbamidomethylation	C2:Carbamidomethylation:1000.00	PEAKS DB

total 2 peptides

Peptide	Uniq	-10lgP	Mass	Length	ppm	m/z	z	RT	Fraction	Scan	Source File	Area ebraeus	Area judaeus	#Feature	#Feature ebraeus	#Feature judaeus	Start	End	PTM	AScore	Found By
LEC(+57.02)PPPSHGQNR.D	Y	63.83	1390.6411	12	0.4	464.5526	3	11.89	2	F2:54854	20210611-09_2021021-OK160A_816_5.3.236.mgf		0	0	0	0	1	12	Carbamidomethylation	C3:Carbamidomethylation:1000.00	PEAKS DB
total 2 peptides																					

V_1258_008_M-5-WF

back to list

| Protein Coverage | Supporting Peptides |

Protein Coverage:

1 DDPRRQRDEK QSPQRDIVQD TVITSNDVAR **TVPHGETETE GKK**NSWWTV

Supporting Peptides:

Peptide	Uniq	-10lgP	Mass	Length	ppm	m/z	z	RT	Fraction	Scan	Source File	Area ebraeus	Area judaeus	#Feature	#Feature ebraeus	#Feature judaeus	Start	End	PTM	AScore	Found By
R.TVPHGETETEGKK.N	Y	74.90	1411.6943	13	4.0	471.5722	3	8.52	1	F1:501	20210611-08_2021021-OK192A_815_5.3.236.mgf	0		0	0	0	31	43			PEAKS DB
R.TVPHGETETEGK.K	Y	55.19	1283.5994	12	4.5	428.8740	3	10.36	1	F1:1570	20210611-08_2021021-OK192A_815_5.3.236.mgf	0		0	0	0	31	42			PEAKS DB
total 2 peptides																					

V_1302_055_M-5-WF

back to list

| Protein Coverage | Supporting Peptides |

Protein Coverage:

1 DDPRRQRDEK QSPQRDIVQD TVITSNDVAR **TVPHGETETE GKK**NSWWTLK ERATAGSAFQ HADKTSLTNP

Supporting Peptides:

Peptide	Uniq	-10lgP	Mass	Length	ppm	m/z	z	RT	Fraction	Scan	Source File	Area ebraeus	Area judaeus	#Feature	#Feature ebraeus	#Feature judaeus	Start	End	PTM	AScore	Found By
R.TVPHGETETEGKK.N	Y	74.90	1411.6943	13	4.0	471.5722	3	8.52	1	F1:501	20210611-08_2021021-OK192A_815_5.3.236.mgf	0		0	0	0	31	43			PEAKS DB
R.TVPHGETETEGK.K	Y	55.19	1283.5994	12	4.5	428.8740	3	10.36	1	F1:1570	20210611-08_2021021-OK192A_815_5.3.236.mgf	0		0	0	0	31	42			PEAKS DB
total 2 peptides																					

SRX1323887_E_029_D

back to list

| Protein Coverage | Supporting Peptides |

Protein Coverage:

2
C

1 **ACYTSQPGSE WGK**CCLTKMC GDKCCRTIHC RCVFGDFNGQ GCSC

C Carbamidomethylation (+57.02)

Supporting Peptides:

Peptide	Uniq	-10lgP	Mass	Length	ppm	m/z	z	RT	Fraction	Scan	Source File	Area ebraeus	Area judaeus	#Feature	#Feature ebraeus	#Feature judaeus	Start	End	PTM	AScore	Found By
AC(+57.02)YTSQPGSEWGK.C	Y	101.45	1469.6245	13	3.5	735.8193	2	24.79	1	F1:17774	20210611-08_2021021-OK192A_815_5.3.236.mgf	0		0	0	0	1	13	Carbamidomethylation	C2:Carbamidomethylation:1000.00	PEAKS DB
total 1 peptides																					

OK213_E_034_D

back to list

| Protein Coverage | Supporting Peptides |

Protein Coverage:

2
C

1 **ACYTSQPGSE WGK**CCLTKMC GDKCCHTIHC RCVSGDFKGQ GCSC

C Carbamidomethylation (+57.02)

Supporting Peptides:

Peptide	Uniq	-10lgP	Mass	Length	ppm	m/z	z	RT	Fraction	Scan	Source File	Area ebraeus	Area judaeus	#Feature	#Feature ebraeus	#Feature judaeus	Start	End	PTM	AScore	Found By
AC(+57.02)YTSQPGSEWGK.C	Y	101.45	1469.6245	13	3.5	735.8193	2	24.79	1	F1:17774	20210611-08_2021021-OK192A_815_5.3.236.mgf	0		0	0	0	1	13	Carbamidomethylation	C2:Carbamidomethylation:1000.00	PEAKS DB
total 1 peptides																					

V_CG13_175_T-3-WF

back to list

| Protein Coverage | Supporting Peptides |

Protein Coverage:

1 NPLETR**IQSD LIRAGLEGAD MK**TEERFLGA LVGPALNALT SLLSGRKK

Supporting Peptides:

Peptide	Uniq	-10lgP	Mass	Length	ppm	m/z	z	RT	Fraction	Scan	Source File	Area ebraeus	Area judaeus	#Feature	#Feature ebraeus	#Feature judaeus	Start	End	PTM	AScore	Found By
R.AGLEGADMK.T	Y	69.85	890.4167	9	6.0	446.2166	2	18.69	1	F1:10092	20210611-08_2021021-OK192A_815_5.3.236.mgf	0		0	0	0	14	22			PEAKS DB
R.AGLEGADM(+15.99)K.T	Y	68.12	906.4117	9	5.2	454.2137	2	13.44	1	F1:4690	20210611-08_2021021-OK192A_815_5.3.236.mgf	0		0	0	0	14	22		M8:Oxidation (M):1000.00	PEAKS DB
R.IQSDLIR.A	N	59.76	843.4814	7	4.4	422.7482	2	23.53	1	F1:15827	20210611-08_2021021-OK192A_815_5.3.236.mgf	0	0	0	0	0	7	13			PEAKS DB
total 3 peptides																					

OK006_E_057_I1

back to list

| Protein Coverage | Supporting Peptides |

Protein Coverage:

22 23 39
C C C

1 QFECVEGGEN CRADSNCCSR **VCCQLSTGK**R CAFK**QTVGCD DIHLN**

C Carbamidomethylation (+57.02)

Supporting Peptides:

Peptide	Uniq	-10lgP	Mass	Length	ppm	m/z	z	RT	Fraction	Scan	Source File	Area ebraeus	Area judaeus	#Feature	#Feature ebraeus	#Feature judaeus	Start	End	PTM	AScore	Found By
K.QTVGC(+57.02)DDIHLN	Y	71.49	1270.5612	11	2.9	636.2873	2	28.23	1	F1:22885	20210611-08_2021021-OK192A_815_5.3.236.mgf	0		0	0	0	35	45	Carbamidomethylation	C5:Carbamidomethylation:1000.00	PEAKS DB
R.VC(+57.02)C(+57.02)QLSTGK.R	Y	53.95	1051.4790	9	0.2	526.7449	2	15.04	1	F1:6163	20210611-08_2021021-OK192A_815_5.3.236.mgf	0		0	0	0	21	29	Carbamidomethylation	C2:Carbamidomethylation:1000.00;C3:Carbamidomethylation:1000.00	PEAKS DB
total 2 peptides																					

OK213_E_051_I1

back to list

| Protein Coverage | Supporting Peptides |

Protein Coverage:

22 23 C C; 39 C

1 QFECVEGGEN CRADSNCCSR **VCCQLSTGK**R CAFK**QTVGCD DIHLN**

C Carbamidomethylation (+57.02)

Supporting Peptides:

Peptide	Uniq	-10lgP	Mass	Length	ppm	m/z	z	RT	Fraction	Scan	Source File	Area ebraeus	Area judaeus	#Feature	#Feature ebraeus	#Feature judaeus	Start	End	PTM	AScore	Found By
K.QTVGC(+57.02)DDIHLN	Y	71.49	1270.5612	11	2.9	636.2873	2	28.23	1	F1:22885	20210611-08_2021021-OK192A_815_5.3.236.mgf	0		0	0	0	35	45	Carbamidomethylation	C5:Carbamidomethylation:1000.00	PEAKS DB
R.VC(+57.02)C(+57.02)QLSTGK.R	Y	53.95	1051.4790	9	0.2	526.7449	2	15.04	1	F1:6163	20210611-08_2021021-OK192A_815_5.3.236.mgf	0		0	0	0	21	29	Carbamidomethylation	C2:Carbamidomethylation:1000.00;C3:Carbamidomethylation:1000.00	PEAKS DB
total 2 peptides																					

CebrOK014_E_015_I1

back to list

| Protein Coverage | Supporting Peptides |

Protein Coverage:

22 23 C C; 39 C

1 QFECVEGGEN CRADSNCCSR **VCCQLSTGK**R CAFK**QTVGCD DIHLN**

C Carbamidomethylation (+57.02)

Supporting Peptides:

Peptide	Uniq	-10lgP	Mass	Length	ppm	m/z	z	RT	Fraction	Scan	Source File	Area ebraeus	Area judaeus	#Feature	#Feature ebraeus	#Feature judaeus	Start	End	PTM	AScore	Found By
K.QTVGC(+57.02)DDIHLN	Y	71.49	1270.5612	11	2.9	636.2873	2	28.23	1	F1:22885	20210611-08_2021021-OK192A_815_5.3.236.mgf	0		0	0	0	35	45	Carbamidomethylation	C5:Carbamidomethylation:1000.00	PEAKS DB
R.VC(+57.02)C(+57.02)QLSTGK.R	Y	53.95	1051.4790	9	0.2	526.7449	2	15.04	1	F1:6163	20210611-08_2021021-OK192A_815_5.3.236.mgf	0		0	0	0	21	29	Carbamidomethylation	C2:Carbamidomethylation:1000.00;C3:Carbamidomethylation:1000.00	PEAKS DB
total 2 peptides																					

OK193_E_044_I1

back to list

| Protein Coverage | Supporting Peptides |

Protein Coverage:

22 23 C C; 39 C

1 QFKCVEGGEN CRADSNCCSR **VCCQLSTGK**R CAFK**QTVGCD DIHLN**

C Carbamidomethylation (+57.02)

Supporting Peptides:

Peptide	Uniq	-10lgP	Mass	Length	ppm	m/z	z	RT	Fraction	Scan	Source File	Area ebraeus	Area judaeus	#Feature	#Feature ebraeus	#Feature judaeus	Start	End	PTM	AScore	Found By
K.QTVGC(+57.02)DDIHLN	Y	71.49	1270.5612	11	2.9	636.2873	2	28.23	1	F1:22885	20210611-08_2021021-OK192A_815_5.3.236.mgf	0		0	0	0	35	45	Carbamidomethylation	C5:Carbamidomethylation:1000.00	PEAKS DB
R.VC(+57.02)C(+57.02)QLSTGK.R	Y	53.95	1051.4790	9	0.2	526.7449	2	15.04	1	F1:6163	20210611-08_2021021-OK192A_815_5.3.236.mgf	0		0	0	0	21	29	Carbamidomethylation	C2:Carbamidomethylation:1000.00;C3:Carbamidomethylation:1000.00	PEAKS DB
total 2 peptides																					

SRX1323887_E_041_I1

back to list

| Protein Coverage | Supporting Peptides |

Protein Coverage:

22 23 C C; 39 C

1 QFKCVEGGEN CRADSNCCSR **VCCQLSTGK**R CAFK**QTVGCD DIHLN**

C Carbamidomethylation (+57.02)

Supporting Peptides:

Peptide	Uniq	-10lgP	Mass	Length	ppm	m/z	z	RT	Fraction	Scan	Source File	Area ebraeus	Area judaeus	#Feature	#Feature ebraeus	#Feature judaeus	Start	End	PTM	AScore	Found By
K.QTVGC(+57.02)DDIHLN	Y	71.49	1270.5612	11	2.9	636.2873	2	28.23	1	F1:22885	20210611-08_2021021-OK192A_815_5.3.236.mgf	0		0	0	0	35	45	Carbamidomethylation	C5:Carbamidomethylation:1000.00	PEAKS DB
R.VC(+57.02)C(+57.02)QLSTGK.R	Y	53.95	1051.4790	9	0.2	526.7449	2	15.04	1	F1:6163	20210611-08_2021021-OK192A_815_5.3.236.mgf	0		0	0	0	21	29	Carbamidomethylation	C2:Carbamidomethylation:1000.00;C3:Carbamidomethylation:1000.00	PEAKS DB
total 2 peptides																					

OK006_E_265_Conopressin

back to list

| Protein Coverage | Supporting Peptides |

Protein Coverage:

1 RLTLVLCLLL LLLLTTQACF IRNCPKGGKR DVDERQLVHA CMFCSFGQCV GPHICCGDGG CQMGTPEANR **CSQEDESPIP**

81 **CR**VIGNHCSL NNPGNIHGHC VAYGICCVDD TCTTHSGCL

Supporting Peptides:

Peptide	Uniq	-10lgP	Mass	Length	ppm	m/z	z	RT	Fraction	Scan	Source File	Area ebraeus	Area judaeus	#Feature	#Feature ebraeus	#Feature judaeus	Start	End	PTM	AScore	Found By
R.C(+57.02)SQEDESPIPC(+57.02)R.V	Y	98.21	1476.5973	12	1.0	739.3036	2	21.86	2	F2:69231	20210611-09_2021021-OK160A_816_5.3.236.mgf		0	0	0	0	71	82		C1:Carbamidomethylation:1000.00;C11:Carbamidomethylation:1000.00	PEAKS DB
total 1 peptides																					

CebrOK035_J_333_Conopressin/Conophysin

back to list

| Protein Coverage | Supporting Peptides |

Protein Coverage:

```
 1 MTRSAMQMGR LTLVLCLLLL LLLTTQACFI RNCPKGGKRD VDERQLVHAC MFCSFGQCVG PHICCGDGGC QMGTPEANRC
81 SQEDESPIPC RVIGNHCSLN NPGNIHGHCV AYGICCVDDT CTTHSGCL
```

Supporting Peptides:

Peptide	Uniq	-10lgP	Mass	Length	ppm	m/z	z	RT	Fraction	Scan	Source File	Area ebraeus	Area judaeus	#Feature	#Feature ebraeus	#Feature judaeus	Start	End	PTM	AScore	Found By
R.C(+57.02)SQEDESPIPC(+57.02)R.V	Y	98.21	1476.5973	12	1.0	739.3036	2	21.86	2	F2:69231	20210611-09_2021021-OK160A_816_5.3.236.mgf		0	0	0	0	80	91		C1:Carbamidomethylation:1000.00;C11:Carbamidomethylation:1000.00	PEAKS DB
**total 1 peptides**																					

CebrOK033_J_111_O1

back to list

| Protein Coverage | Supporting Peptides |

Protein Coverage:

```
1 DDSINGPDNR GIWGKPSSKA RDEMKNPKDS RIERVHS
```

Supporting Peptides:

Peptide	Uniq	-10lgP	Mass	Length	ppm	m/z	z	RT	Fraction	Scan	Source File	Area ebraeus	Area judaeus	#Feature	#Feature ebraeus	#Feature judaeus	Start	End	PTM	AScore	Found By
DDSINGPDNR.G	Y	68.61	1101.4686	10	1.8	551.7403	2	14.36	2	F2:58024	20210611-09_2021021-OK160A_816_5.3.236.mgf		0	0	0	0	1	10			PEAKS DB
R.GIWGKPSSK.A	Y	54.17	958.5236	9	3.3	480.2686	2	16.90	2	F2:61420	20210611-09_2021021-OK160A_816_5.3.236.mgf		0	0	0	0	11	19			PEAKS DB
**total 2 peptides**																					

OK213_E_029_Conkunitzin

back to list

| Protein Coverage | Supporting Peptides |

Protein Coverage:

```
1 CYEPFDLGDS TCRTLRIRFY YNQENKKCQT FQYTGCGGNG NNFPDLYACK ECCEWDMTCY
```

Supporting Peptides:

Peptide	Uniq	-10lgP	Mass	Length	ppm	m/z	z	RT	Fraction	Scan	Source File	Area ebraeus	Area judaeus	#Feature	#Feature ebraeus	#Feature judaeus	Start	End	PTM	AScore	Found By
R.FYYNQENK.K	Y	63.26	1104.4875	8	0.1	553.2490	2	19.53	1	F1:10977	20210611-08_2021021-OK192A_815_5.3.236.mgf	0		0	0	0	19	26			PEAKS DB
R.FYYNQENKK.C	Y	61.47	1232.5825	9	-1.5	617.2953	2	15.36	1	F1:6600	20210611-08_2021021-OK192A_815_5.3.236.mgf	0		0	0	0	19	27			PEAKS DB
**total 2 peptides**																					

OK213_E_035_D

back to list

| Protein Coverage | Supporting Peptides |

Protein Coverage:

```
1 ACYYHQPNSP WGRCCPTKMC GSKCCDRANC RCVSHADRGQ GCSC
```

C Carbamidomethylation (+57.02)

Supporting Peptides:

Peptide	Uniq	-10lgP	Mass	Length	ppm	m/z	z	RT	Fraction	Scan	Source File	Area ebraeus	Area judaeus	#Feature	#Feature ebraeus	#Feature judaeus	Start	End	PTM	AScore	Found By
AC(+57.02)YYHQPNSPWGR.C	Y	93.29	1634.7048	13	1.8	545.9078	3	27.32	1	F1:21579	20210611-08_2021021-OK192A_815_5.3.236.mgf	0		0	0	0	1	13	Carbamidomethylation	C2:Carbamidomethylation:1000.00	PEAKS DB
**total 1 peptides**																					

OK193_E_028_D

back to list

| Protein Coverage | Supporting Peptides |

Protein Coverage:

```
1 ACYYHQPNSP WGRCCPTKMC GSKCCDRANC RCVSHADRGQ GCSC
```

C Carbamidomethylation (+57.02)

Supporting Peptides:

Peptide	Uniq	-10lgP	Mass	Length	ppm	m/z	z	RT	Fraction	Scan	Source File	Area ebraeus	Area judaeus	#Feature	#Feature ebraeus	#Feature judaeus	Start	End	PTM	AScore	Found By
AC(+57.02)YYHQPNSPWGR.C	Y	93.29	1634.7048	13	1.8	545.9078	3	27.32	1	F1:21579	20210611-08_2021021-OK192A_815_5.3.236.mgf	0		0	0	0	1	13	Carbamidomethylation	C2:Carbamidomethylation:1000.00	PEAKS DB
**total 1 peptides**																					

CebrOK014_E_009_D

back to list

| Protein Coverage | Supporting Peptides |

Protein Coverage:

```
1 ACYYHQPNSP WGRCCPT
```

C Carbamidomethylation (+57.02)

Supporting Peptides:

Peptide	Uniq	-10lgP	Mass	Length	ppm	m/z	z	RT	Fraction	Scan	Source File	Area ebraeus	Area judaeus	#Feature	#Feature ebraeus	#Feature judaeus	Start	End	PTM	AScore	Found By
AC(+57.02)YYHQPNSPWGR.C	Y	93.29	1634.7048	13	1.8	545.9078	3	27.32	1	F1:21579	20210611-08_2021021-OK192A_815_5.3.236.mgf	0		0	0	0	1	13	Carbamidomethylation	C2:Carbamidomethylation:1000.00	PEAKS DB
**total 1 peptides**																					

V_1258_145_T-3

back to list

| Protein Coverage | Supporting Peptides |

Protein Coverage:

```
1 AGLEDAGAKF EKGLLSAVVQ NLDKIGGTAT KVCCYFTKTC CPGDDE
```

Supporting Peptides:

Peptide	Uniq	-10lgP	Mass	Length	ppm	m/z	z	RT	Fraction	Scan	Source File	Area ebraeus	Area judaeus	#Feature	#Feature ebraeus	#Feature judaeus	Start	End	PTM	AScore	Found By
AGLEDAGAK.F	Y	60.70	830.4133	9	3.6	416.2139	2	14.24	1	F1:5438	20210611-08_2021021-OK192A_815_5.3.236.mgf	0		0	0	0	1	9			PEAKS DB
**total 2 peptides**																					

Peptide	Uniq	-10lgP	Mass	Length	ppm	m/z	z	RT	Fraction	Scan	Source File	Area ebraeus	Area judaeus	#Feature	#Feature ebraeus	#Feature judaeus	Start	End	PTM	AScore	Found By
K.GLLSAVVQNLDK.I	Y	57.90	1255.7136	12	-0.1	628.8616	2	50.98	1	F1:47930	20210611-08_2021021-OK192A_815_5.3.236.mgf	0		0	0	0	13	24			PEAKS DB
total 2 peptides																					

V_1302_153_T-3

back to list

| Protein Coverage | Supporting Peptides |

Protein Coverage:

1 **AGLEDAGAK**F EK**GLLSAVVQ NLDK**IGGTAT KVCCYFTKTC CPGDDE

Supporting Peptides:

Peptide	Uniq	-10lgP	Mass	Length	ppm	m/z	z	RT	Fraction	Scan	Source File	Area ebraeus	Area judaeus	#Feature	#Feature ebraeus	#Feature judaeus	Start	End	PTM	AScore	Found By
AGLEDAGAK.F	Y	60.70	830.4133	9	3.6	416.2139	2	14.24	1	F1:5438	20210611-08_2021021-OK192A_815_5.3.236.mgf	0		0	0	0	1	9			PEAKS DB
K.GLLSAVVQNLDK.I	Y	57.90	1255.7136	12	-0.1	628.8616	2	50.98	1	F1:47930	20210611-08_2021021-OK192A_815_5.3.236.mgf	0		0	0	0	13	24			PEAKS DB
total 2 peptides																					

V_1302_154_T-3

back to list

| Protein Coverage | Supporting Peptides |

Protein Coverage:

1 **AGLEDAGAK**F EK**GLLSAVVQ NLDK**IGGTAT KVCCYFTTSC CPDDDE

Supporting Peptides:

Peptide	Uniq	-10lgP	Mass	Length	ppm	m/z	z	RT	Fraction	Scan	Source File	Area ebraeus	Area judaeus	#Feature	#Feature ebraeus	#Feature judaeus	Start	End	PTM	AScore	Found By
AGLEDAGAK.F	Y	60.70	830.4133	9	3.6	416.2139	2	14.24	1	F1:5438	20210611-08_2021021-OK192A_815_5.3.236.mgf	0		0	0	0	1	9			PEAKS DB
K.GLLSAVVQNLDK.I	Y	57.90	1255.7136	12	-0.1	628.8616	2	50.98	1	F1:47930	20210611-08_2021021-OK192A_815_5.3.236.mgf	0		0	0	0	13	24			PEAKS DB
total 2 peptides																					

V_1258_144_T-3

back to list

| Protein Coverage | Supporting Peptides |

Protein Coverage:

1 **AGLEDAGAK**F EK**GLLSAVVQ NLDK**IGGTAT KVCCYFTTSC CPDDDE

Supporting Peptides:

Peptide	Uniq	-10lgP	Mass	Length	ppm	m/z	z	RT	Fraction	Scan	Source File	Area ebraeus	Area judaeus	#Feature	#Feature ebraeus	#Feature judaeus	Start	End	PTM	AScore	Found By
AGLEDAGAK.F	Y	60.70	830.4133	9	3.6	416.2139	2	14.24	1	F1:5438	20210611-08_2021021-OK192A_815_5.3.236.mgf	0		0	0	0	1	9			PEAKS DB
K.GLLSAVVQNLDK.I	Y	57.90	1255.7136	12	-0.1	628.8616	2	50.98	1	F1:47930	20210611-08_2021021-OK192A_815_5.3.236.mgf	0		0	0	0	13	24			PEAKS DB
total 2 peptides																					

V_CG13_170_T-3

back to list

| Protein Coverage | Supporting Peptides |

Protein Coverage:

1 **AGLEDAGAK**F EK**GLLSAVVQ NLDK**IGGTAT KVCCYFTTSC CPDDDE

Supporting Peptides:

Peptide	Uniq	-10lgP	Mass	Length	ppm	m/z	z	RT	Fraction	Scan	Source File	Area ebraeus	Area judaeus	#Feature	#Feature ebraeus	#Feature judaeus	Start	End	PTM	AScore	Found By
AGLEDAGAK.F	Y	60.70	830.4133	9	3.6	416.2139	2	14.24	1	F1:5438	20210611-08_2021021-OK192A_815_5.3.236.mgf	0		0	0	0	1	9			PEAKS DB
K.GLLSAVVQNLDK.I	Y	57.90	1255.7136	12	-0.1	628.8616	2	50.98	1	F1:47930	20210611-08_2021021-OK192A_815_5.3.236.mgf	0		0	0	0	13	24			PEAKS DB
total 2 peptides																					

V_CG13_174_T-3

back to list

| Protein Coverage | Supporting Peptides |

Protein Coverage:

1 **AGLEDAGAK**F EK**GLLSAVVQ NLDK**IGGTAT KVCCFFTKSC CSDDDE

Supporting Peptides:

Peptide	Uniq	-10lgP	Mass	Length	ppm	m/z	z	RT	Fraction	Scan	Source File	Area ebraeus	Area judaeus	#Feature	#Feature ebraeus	#Feature judaeus	Start	End	PTM	AScore	Found By
AGLEDAGAK.F	Y	60.70	830.4133	9	3.6	416.2139	2	14.24	1	F1:5438	20210611-08_2021021-OK192A_815_5.3.236.mgf	0		0	0	0	1	9			PEAKS DB
K.GLLSAVVQNLDK.I	Y	57.90	1255.7136	12	-0.1	628.8616	2	50.98	1	F1:47930	20210611-08_2021021-OK192A_815_5.3.236.mgf	0		0	0	0	13	24			PEAKS DB
total 2 peptides																					

gene:107288629

back to list

| Protein Coverage | Supporting Peptides |

Protein Coverage:

1 MWCSPFLRSP FVLGQCSPSR AQSALAQLKR LLEGELEGIR GAGTWKNERI ITSKQGPHIN VEGSRGDILN FCANNYLGLS
81 SHPEVIRAGH DALEKYGAGL SSVRFICGTQ NIHKDLEEKI ARFHQREDAI LYISCFDANA GIFEALLTPE DAILSDELNH
161 ASIIDGIRLC KANKYRYKHM DMQDLEVKLQ DAQKHRLRMV ATDGAFSMDG DIAPLKEICR LAQKYKAFVF IDECHATGFL
241 GPNGRGTDEL LGVMDQVTII NSTLGK**ALGG AAGGYTTGPK** ALVDLLRQRS RPYLFSNSLP PAVVGCASKA LDLLMESNAI
321 AQSMAAKTSQ FRSKMAAAGF TISGNNHPIC PVMLGDARLA SVMADDILKR GIYVIGFSYP VVPKGKARIR VQISAVHSEE
401 DIDRCVEAFI EVGRKHGALP

Supporting Peptides:

Peptide	Uniq	-10lgP	Mass	Length	ppm	m/z	z	RT	Fraction	Scan	Source File	Area ebraeus	Area judaeus	#Feature	#Feature ebraeus	#Feature judaeus	Start	End	PTM	AScore	Found By
K.ALGGAAGGYTTGPK.A	Y	86.87	1219.6196	14	2.4	610.8160	2	21.84	2	F2:69280	20210611-09_2021021-OK160A_816_5.3.236.mgf		0	0	0	0	267	280			PEAKS DB
total 1 peptides																					

CebrOK035_J_218_Q

back to list

| Protein Coverage | Supporting Peptides |

Protein Coverage:

35
C

1 MDGSDCTPCG PDLCCSPEKS CGK**STHHEHY GEPACV**

C Carbamidomethylation (+57.02)

Supporting Peptides:

Peptide	Uniq	-10lgP	Mass	Length	ppm	m/z	z	RT	Fraction	Scan	Source File	Area ebraeus	Area judaeus	#Feature	#Feature ebraeus	#Feature judaeus	Start	End	PTM	AScore	Found By
K.STHHEHYGEPAC(+57.02)V	Y	86.20	1522.6259	13	-5.4	762.3129	2	14.73	2	F2:58605	20210611-09_2021021-OK160A_816_5.3.236.mgf		0	0	0	0	24	36	Carbamidomethylation	C12:Carbamidomethylation:1000.00	PEAKS DB
**total 1 peptides**																					

OK214_J_192_Q

back to list

| Protein Coverage | Supporting Peptides |

Protein Coverage:

35
C

1 MDGSDCTPCG PDLCCSPEKS CGK**STHHEHY GEPACV**

C Carbamidomethylation (+57.02)

Supporting Peptides:

Peptide	Uniq	-10lgP	Mass	Length	ppm	m/z	z	RT	Fraction	Scan	Source File	Area ebraeus	Area judaeus	#Feature	#Feature ebraeus	#Feature judaeus	Start	End	PTM	AScore	Found By
K.STHHEHYGEPAC(+57.02)V	Y	86.20	1522.6259	13	-5.4	762.3129	2	14.73	2	F2:58605	20210611-09_2021021-OK160A_816_5.3.236.mgf		0	0	0	0	24	36	Carbamidomethylation	C12:Carbamidomethylation:1000.00	PEAKS DB
**total 1 peptides**																					

gene:107290492

back to list

| Protein Coverage | Supporting Peptides |

Protein Coverage:

1 MAVVAAVRGS RGGRRFWFEI SRKTNFNWHS SSFISSTSVP TTKLFIDGKF VESKSNEWIE IYNPATNEVV GRVPKATQNE
81 MEAAVASCKK ALPSWSEVSI LSRQQIFLRY QQLIKDNLKE ISKLITLEQG KTLADAEGDV FRGLQVVEHA CSVTSLMLGE
161 TLPSITKDMD TCTYRLPLGV CAGIAPFNFP AMIPLWMFPM AMVCGNTFLM KPSERVPGAL MFLAQLLQDA GAPDGTLNII
241 HGQHEAVTFI CDHPDIK**AIS FVGSNQAGEY IYER**GSKHGK RVQANMGAKN HGVVMPDANK ENTLNQLIGA AFGAAGQRCM
321 ALSAAILVGE SQKWLPELVE KAKNLCVNAG DQPGADLGPL ISPQARERVC QLIDSGAKEG ANVLLDGRKV RVKGFENGNF
401 VGPTILGKVK PNMTCYKEEI FGPVLVVLEA NNLDDAIEMV NNNPYGNGTA IFTTNGATAR KYSHLVDVGQ IGVNVPIPVP
481 LPMFSFTGSR GSFRGDTNFY GKQGVQFYTQ LKTVISQWKE EDATVTKPAV VMPTMGN

Supporting Peptides:

Peptide	Uniq	-10lgP	Mass	Length	ppm	m/z	z	RT	Fraction	Scan	Source File	Area ebraeus	Area judaeus	#Feature	#Feature ebraeus	#Feature judaeus	Start	End	PTM	AScore	Found By
K.AISFVGSNQAGEYIYER.G	Y	84.88	1902.9111	17	3.8	952.4625	2	42.85	2	F2:106351	20210611-09_2021021-OK160A_816_5.3.236.mgf		0	0	0	0	258	274			PEAKS DB
**total 1 peptides**																					

CebrOK016_E_005_B2

back to list

| Protein Coverage | Supporting Peptides |

Protein Coverage:

1 FPQRRDGAPE NADPNLQPFG GVIPNMQPMQ GMTGMPSPQF LPFNPNFAVG LKRENLEKRR **HNPQFSDNNQ SPFGSEDDLD**

81 **SFMNFMKENG NNLPFADVNS AATDLGHFEP SDENEAGK**FR FFDKEQ

Supporting Peptides:

Peptide	Uniq	-10lgP	Mass	Length	ppm	m/z	z	RT	Fraction	Scan	Source File	Area ebraeus	Area judaeus	#Feature	#Feature ebraeus	#Feature judaeus	Start	End	PTM	AScore	F[illegible]
K.ENGNNLPFADVNSAATDLGHFEPSDENEAGK.F	Y	57.19	3258.4336	31	7.7	1087.1560	3	49.05	1	F1:46660	20210611-08_2021021-OK192A_815_5.3.236.mgf	0		0	0	0	88	118			[illegible]
R.HNPQFSDNNQSPFGSEDDLDSFM(+15.99)NFM(+15.99)K.E	Y	55.34	3179.2874	27	7.5	1060.7737	3	45.54	1	F1:43754	20210611-08_2021021-OK192A_815_5.3.236.mgf	0		0	0	0	61	87		M23:Oxidation(M):1000.00; M26:Oxidation(M):1000.00	[illegible]
**total 2 peptides**																					

CebrOK014_E_004_B2

back to list

| Protein Coverage | Supporting Peptides |

Protein Coverage:

1 FPQRRDGAPA NVDPNLQPFG GVIPNMQPMQ GMTGMPSPQF LPFNPNFAVG LKRENLEKRR **HNPQFSDNNQ SPFGSEDDLD**

81 **SFMNFMKENG NNLPFADVNS AATDLGHFEP SDENEAGK**FR FFDKEQ

Supporting Peptides:

Peptide	Uniq	-10lgP	Mass	Length	ppm	m/z	z	RT	Fraction	Scan	Source File	Area ebraeus	Area judaeus	#Feature	#Feature ebraeus	#Feature judaeus	Start	End	PTM	AScore	F[illegible]
K.ENGNNLPFADVNSAATDLGHFEPSDENEAGK.F	Y	57.19	3258.4336	31	7.7	1087.1560	3	49.05	1	F1:46660	20210611-08_2021021-OK192A_815_5.3.236.mgf	0		0	0	0	88	118			[illegible]
R.HNPQFSDNNQSPFGSEDDLDSFM(+15.99)NFM(+15.99)K.E	Y	55.34	3179.2874	27	7.5	1060.7737	3	45.54	1	F1:43754	20210611-08_2021021-OK192A_815_5.3.236.mgf	0		0	0	0	61	87		M23:Oxidation(M):1000.00; M26:Oxidation(M):1000.00	[illegible]
**total 2 peptides**																					

CebrOK016_E_004_B2

back to list

| Protein Coverage | Supporting Peptides |

Protein Coverage:

1 FPQRRDGAPA NVDPNLQPFG GVIPNMQPMQ GMTGMPSPQF LPFNPNFAVG LKRENLEKRR **HNPQFSDNNQ SPFGSEDDLD**

81 **SFMNFMKENG NNLPFADVNS AATDLGHFEP SDENEAGK**FR FFDKEQ

Supporting Peptides:

Peptide	Uniq	-10lgP	Mass	Length	ppm	m/z	z	RT	Fraction	Scan	Source File	Area ebraeus	Area judaeus	#Feature	#Feature ebraeus	#Feature judaeus	Start	End	PTM	AScore	F[illegible]
K.ENGNNLPFADVNSAATDLGHFEPSDENEAGK.F	Y	57.19	3258.4336	31	7.7	1087.1560	3	49.05	1	F1:46660	20210611-08_2021021-OK192A_815_5.3.236.mgf	0		0	0	0	88	118			[illegible]
**total 2 peptides**																					

Peptide	Uniq	-10lgP	Mass	Length	ppm	m/z	z	RT	Fraction	Scan	Source File	Area ebraeus	Area judaeus	#Feature	#Feature ebraeus	#Feature judaeus	Start	End	PTM	AScore	Fo
R.HNPQFSDNNQSPFGSEDDLDSFM(+15.99)NFM(+15.99)K.E	Y	55.34	3179.2874	27	7.5	1060.7737	3	45.54	1	F1:43754	20210611-08_2021021-OK192A_815_5.3.236.mgf	0		0	0	0	61	87		M23:Oxidation (M):1000.00; M26:Oxidation (M):1000.00	PE
total 2 peptides																					

V_1278_082_T-3

back to list

| **Protein Coverage** | **Supporting Peptides** |

Protein Coverage:

1 **AGLEDADMK**T DDWGVVSDLL NVFMQICCSA FSTCCAEE

Supporting Peptides:

Peptide	Uniq	-10lgP	Mass	Length	ppm	m/z	z	RT	Fraction	Scan	Source File	Area ebraeus	Area judaeus	#Feature	#Feature ebraeus	#Feature judaeus	Start	End	PTM	AScore	Found By
AGLEDADM(+15.99)K.T	Y	84.64	964.4171	9	2.0	483.2150	2	14.43	1	F1:5669	20210611-08_2021021-OK192A_815_5.3.236.mgf	0		0	0	0	1	9		M8:Oxidation (M):1000.00	PEAKS DB
AGLEDADMK.T	Y	81.44	948.4222	9	3.7	475.2184	2	20.05	1	F1:11485	20210611-08_2021021-OK192A_815_5.3.236.mgf	0	0	0	0	0	1	9			PEAKS DB
total 2 peptides																					

AXL95619_conotoxin_precursor_superfamily_T_Conus_ermineus

back to list

| **Protein Coverage** | **Supporting Peptides** |

Protein Coverage:

1 **AGLEDADMK**T DDWGVVSDLL NVFMQICCSA FSTCCAEE

Supporting Peptides:

Peptide	Uniq	-10lgP	Mass	Length	ppm	m/z	z	RT	Fraction	Scan	Source File	Area ebraeus	Area judaeus	#Feature	#Feature ebraeus	#Feature judaeus	Start	End	PTM	AScore	Found By
AGLEDADM(+15.99)K.T	Y	84.64	964.4171	9	2.0	483.2150	2	14.43	1	F1:5669	20210611-08_2021021-OK192A_815_5.3.236.mgf	0		0	0	0	1	9		M8:Oxidation (M):1000.00	PEAKS DB
AGLEDADMK.T	Y	81.44	948.4222	9	3.7	475.2184	2	20.05	1	F1:11485	20210611-08_2021021-OK192A_815_5.3.236.mgf	0	0	0	0	0	1	9			PEAKS DB
total 2 peptides																					

V_1278_081_T-3

back to list

| **Protein Coverage** | **Supporting Peptides** |

Protein Coverage:

1 **AGLEDADMK**T DDWGVVSDLL NVFMQICCSA FSTCCAEE

Supporting Peptides:

Peptide	Uniq	-10lgP	Mass	Length	ppm	m/z	z	RT	Fraction	Scan	Source File	Area ebraeus	Area judaeus	#Feature	#Feature ebraeus	#Feature judaeus	Start	End	PTM	AScore	Found By
AGLEDADM(+15.99)K.T	Y	84.64	964.4171	9	2.0	483.2150	2	14.43	1	F1:5669	20210611-08_2021021-OK192A_815_5.3.236.mgf	0		0	0	0	1	9		M8:Oxidation (M):1000.00	PEAKS DB
AGLEDADMK.T	Y	81.44	948.4222	9	3.7	475.2184	2	20.05	1	F1:11485	20210611-08_2021021-OK192A_815_5.3.236.mgf	0	0	0	0	0	1	9			PEAKS DB
total 2 peptides																					

MATURECV10_022_Conotoxin_precursor_T_T_3

back to list

| **Protein Coverage** | **Supporting Peptides** |

Protein Coverage:

1 **AGLEDADMK**T DDWGVVSDLL NVFMQICCSA FSTCCAEE

Supporting Peptides:

Peptide	Uniq	-10lgP	Mass	Length	ppm	m/z	z	RT	Fraction	Scan	Source File	Area ebraeus	Area judaeus	#Feature	#Feature ebraeus	#Feature judaeus	Start	End	PTM	AScore	Found By
AGLEDADM(+15.99)K.T	Y	84.64	964.4171	9	2.0	483.2150	2	14.43	1	F1:5669	20210611-08_2021021-OK192A_815_5.3.236.mgf	0		0	0	0	1	9		M8:Oxidation (M):1000.00	PEAKS DB
AGLEDADMK.T	Y	81.44	948.4222	9	3.7	475.2184	2	20.05	1	F1:11485	20210611-08_2021021-OK192A_815_5.3.236.mgf	0	0	0	0	0	1	9			PEAKS DB
total 2 peptides																					

V_1278_083_T-3

back to list

| **Protein Coverage** | **Supporting Peptides** |

Protein Coverage:

1 **AGLEDADMK**T DDWGVVSDLL NVFMQICCSA FSTCCKLS

Supporting Peptides:

Peptide	Uniq	-10lgP	Mass	Length	ppm	m/z	z	RT	Fraction	Scan	Source File	Area ebraeus	Area judaeus	#Feature	#Feature ebraeus	#Feature judaeus	Start	End	PTM	AScore	Found By
AGLEDADM(+15.99)K.T	Y	84.64	964.4171	9	2.0	483.2150	2	14.43	1	F1:5669	20210611-08_2021021-OK192A_815_5.3.236.mgf	0		0	0	0	1	9		M8:Oxidation (M):1000.00	PEAKS DB
AGLEDADMK.T	Y	81.44	948.4222	9	3.7	475.2184	2	20.05	1	F1:11485	20210611-08_2021021-OK192A_815_5.3.236.mgf	0	0	0	0	0	1	9			PEAKS DB
total 2 peptides																					

Pvau2_102

back to list

| **Protein Coverage** | **Supporting Peptides** |

Protein Coverage:

```
  1 YHDYCIIGAG PAGLQMGYFL KKANRDYMIF ERSNVSGHFF VTYPRHRTLI SINKRYTGKT NKEFNLRHDW NSLLSDDESL
 81 LFTKYSKDLF PHADRLLDYF RDYQQKLGLK VQWNTEVSDI HSVHNLSAPD GVLKVFSDQR GQQYSCKVLV MATGMWKPNI
161 PQAEGIEHTV GYENMSIDRN DYEGKTVMIL GHGNSAFEVA QQLYEVTNLV HVFGRSRIRL SWQTHYVGDV RAVNMAVVDS
241 YQLKSLDGVA DASRIELIRI LREGDKLKVL PLGIGNHTYS TFGREAYDVI IRCLGFSFDT SIFRNQTQTG ETKYHSIHHN
321 YESRSMPGVF VAGTASHSLD YRKSSGGFIH GFRYTARTLA RLLEWRYERV PWPSQNLGLP VTQLMNVVLK RLSEASGPYQ
401 MFEMLGDVVV FSEDGKTFDY FEEFPINLLH DFPEHTGRAA SRVLVVVLQY GNDFSGPGED TLRAGRATGY PAKGHLSNFL
481 HPVLYYYHTL PTVAQMKNRS EEEPLPRPDA IHHMVEDFLT RWDHLQFHIM PLRHFLESIL SSDLRHRLDE TCFLETMTRT
561 QASPGCQGYI LQDQVVTPHA SHSM
```

Supporting Peptides:

Peptide	Uniq	-10lgP	Mass	Length	ppm	m/z	z	RT	Fraction	Scan	Source File	Area ebraeus	Area judaeus	#Feature	#Feature ebraeus	#Feature judaeus	Start	End	PTM	AScore	Found By
R.LSWQTHYVGDVR.A	Y	84.51	1459.7208	12	-3.7	730.8619	2	32.22	2	F2:87280	20210611-09_2021021-OK160A_816_5.3.236.mgf	0	0	0	0	0	220	231			PEAKS DB
total 1 peptides																					

Pvau2_101

back to list

| Protein Coverage | Supporting Peptides |

Protein Coverage:

1 YHDYCIIGAG PAGLQMGYFL KKANRDYMIF ERSNVSGHFF VTYPRHRTLI SINKRYTGKT NKEFNLRHDW NSLLSDDESL
81 LFTKYSKDLF PHADRLLDYF RDYQQKLGLK VQWNTEVSDI HSVHNLSAPD GVLKVFSDQR GQQYSCKVLV MATGMWKPNI
161 PQAEGIEHTV GYENMSIDRN DYEGKTVMIL GHGNSAFEVA QQLYEVTNLV HVFGRSRIR**L SWQTHYVGDV R**AVNMAVVDS
241 YQLKSLDGVA DASRIELIRI LREGDKLKVL PLGIGNHTYS TFGREAYDVI IRCLGFSFDT SIFRNQTQTG ETKYHSIHHN
321 YESRSMPGVF VAGTASHSLD YRKSSGGFIH GFRYTARTLA RLLEWRYERV PWPSQNLGLP VTQLMNVVLK RLSEASGPYQ
401 MFEMLGDVVV FSEDGKTFDY FEEFPINLLH DFPEHTGRAA SRVLVVVLQY GNDFSGPGED TLRAGRATGY PAKGHLSNFL
481 HPVLYYYHTL PTVAQMKNRS KEEVLPRPDA IHHMVEDFLT RWDHLQFHIM PLRHFLESIL SSDLRHRLDE ACFLETMTRT
561 QASPGCQGYR IKS

Supporting Peptides:

Peptide	Uniq	-10lgP	Mass	Length	ppm	m/z	z	RT	Fraction	Scan	Source File	Area ebraeus	Area judaeus	#Feature	#Feature ebraeus	#Feature judaeus	Start	End	PTM	AScore	Found By
R.LSWQTHYVGDVR.A	Y	84.51	1459.7208	12	-3.7	730.8619	2	32.22	2	F2:87280	20210611-09_2021021-OK160A_816_5.3.236.mgf	0	0	0	0	0	220	231			PEAKS DB
total 1 peptides																					

OK213_E_130_M

back to list

| Protein Coverage | Supporting Peptides |

Protein Coverage:

1 QMDADQTADR HADERDQDRA EQNRNLMRVL KR**DWFHIDLT PNNK**NTWWTL V

Supporting Peptides:

Peptide	Uniq	-10lgP	Mass	Length	ppm	m/z	z	RT	Fraction	Scan	Source File	Area ebraeus	Area judaeus	#Feature	#Feature ebraeus	#Feature judaeus	Start	End	PTM	AScore	Found By
R.DWFHIDLTPNNK.N	Y	83.34	1498.7205	12	1.7	500.5797	3	48.32	1	F1:46288	20210611-08_2021021-OK192A_815_5.3.236.mgf	0		0	0	0	33	44			PEAKS DB
total 1 peptides																					

OK193_E_113_M

back to list

| Protein Coverage | Supporting Peptides |

Protein Coverage:

1 QMDADQTADR HADERDQDRA EQNRNLMRVL KR**DWFHIDLT PNNK**NTWWTL V

Supporting Peptides:

Peptide	Uniq	-10lgP	Mass	Length	ppm	m/z	z	RT	Fraction	Scan	Source File	Area ebraeus	Area judaeus	#Feature	#Feature ebraeus	#Feature judaeus	Start	End	PTM	AScore	Found By
R.DWFHIDLTPNNK.N	Y	83.34	1498.7205	12	1.7	500.5797	3	48.32	1	F1:46288	20210611-08_2021021-OK192A_815_5.3.236.mgf	0		0	0	0	33	44			PEAKS DB
total 1 peptides																					

SRX1323887_E_086_M

back to list

| Protein Coverage | Supporting Peptides |

Protein Coverage:

1 QMDADQTADR HADERDQDRA EQNRNLMRVL KR**DWFHIDLT PNNK**NTWWTL V

Supporting Peptides:

Peptide	Uniq	-10lgP	Mass	Length	ppm	m/z	z	RT	Fraction	Scan	Source File	Area ebraeus	Area judaeus	#Feature	#Feature ebraeus	#Feature judaeus	Start	End	PTM	AScore	Found By
R.DWFHIDLTPNNK.N	Y	83.34	1498.7205	12	1.7	500.5797	3	48.32	1	F1:46288	20210611-08_2021021-OK192A_815_5.3.236.mgf	0		0	0	0	33	44			PEAKS DB
total 1 peptides																					

OK006_E_111_M

back to list

| Protein Coverage | Supporting Peptides |

Protein Coverage:

1 QMDADQTADR HADERDQDRA EQNRNLMRVL KR**DWFHIDLT PNNK**NTWWTL V

Supporting Peptides:

Peptide	Uniq	-10lgP	Mass	Length	ppm	m/z	z	RT	Fraction	Scan	Source File	Area ebraeus	Area judaeus	#Feature	#Feature ebraeus	#Feature judaeus	Start	End	PTM	AScore	Found By
R.DWFHIDLTPNNK.N	Y	83.34	1498.7205	12	1.7	500.5797	3	48.32	1	F1:46288	20210611-08_2021021-OK192A_815_5.3.236.mgf	0		0	0	0	33	44			PEAKS DB
total 1 peptides																					

SRX1323887_E_022_Con-ikot-ikot

back to list

| Protein Coverage | Supporting Peptides |

Protein Coverage:

16 C

1 KCCATKAYQC LRDHK**CFEPI DYK**CGQPCKN TDTTSCRPTA GDNCCMIYKH CLVECQIFRS DRRGDQLLYC YELCSDSYPC

C Carbamidomethylation (+57.02)

Supporting Peptides:

Peptide	Uniq	-10lgP	Mass	Length	ppm	m/z	z	RT	Fraction	Scan	Source File	Area ebraeus	Area judaeus	#Feature	#Feature ebraeus	#Feature judaeus	Start	End	PTM	AScore	Found By
K.C(+57.02)FEPIDYK.C	Y	82.56	1070.4742	8	4.6	536.2448	2	31.42	1	F1:27261	20210611-08_2021021-OK192A_815_5.3.236.mgf	0		0	0	0	16	23	Carbamidomethylation	C1:Carbamidomethylation:1000.00	PEAKS DB
total 1 peptides																					

OK193_E_023_Con-ikot-ikot

back to list

| Protein Coverage | Supporting Peptides |

Protein Coverage:

16 C

1 KCCATKAYQC LRDHK**CFEPI DYK**CGQPCKN TDTTSCRPTA GDNCCMIYKH CLVDCQIFRS DRRGDQLLYC YELCSDSYPC

C Carbamidomethylation (+57.02)

Supporting Peptides:

Peptide	Uniq	-10lgP	Mass	Length	ppm	m/z	z	RT	Fraction	Scan	Source File	Area ebraeus	Area judaeus	#Feature	#Feature ebraeus	#Feature judaeus	Start	End	PTM	AScore	Found By
K.C(+57.02)FEPIDYK.C	Y	82.56	1070.4742	8	4.6	536.2448	2	31.42	1	F1:27261	20210611-08_2021021-OK192A_815_5.3.236.mgf	0		0	0	0	16	23	Carbamidomethylation	C1:Carbamidomethylation:1000.00	PEAKS DB
total 1 peptides																					

OK006_E_022_Con-ikot-ikot

back to list

| Protein Coverage | Supporting Peptides |

Protein Coverage:

16

1 KCCATKAYQC LRDHK**CFEPI DYK**CGQPCKN TDTTSCRPTA GDNCCMIYKH CLVECQIFRS DRRGDQLLYC YELCSDSYPC

C Carbamidomethylation (+57.02)

Supporting Peptides:

Peptide	Uniq	-10lgP	Mass	Length	ppm	m/z	z	RT	Fraction	Scan	Source File	Area ebraeus	Area judaeus	#Feature	#Feature ebraeus	#Feature judaeus	Start	End	PTM	AScore	Found By
K.C(+57.02)FEPIDYK.C	Y	82.56	1070.4742	8	4.6	536.2448	2	31.42	1	F1:27261	20210611-08_2021021-OK192A_815_5.3.236.mgf	0		0	0	0	16	23	Carbamidomethylation	C1:Carbamidomethylation:1000.00	PEAKS DB
total 1 peptides																					

OK213_E_025_Con-ikot-ikot

back to list

| Protein Coverage | Supporting Peptides |

Protein Coverage:

16

1 KCCATKAYQC LRDHK**CFEPI DYK**CGQPCKN TDTTSCRPTA GDNCCMIYKH CLVECQIFRS DRRGDQLLYC YELCSDSYPC

C Carbamidomethylation (+57.02)

Supporting Peptides:

Peptide	Uniq	-10lgP	Mass	Length	ppm	m/z	z	RT	Fraction	Scan	Source File	Area ebraeus	Area judaeus	#Feature	#Feature ebraeus	#Feature judaeus	Start	End	PTM	AScore	Found By
K.C(+57.02)FEPIDYK.C	Y	82.56	1070.4742	8	4.6	536.2448	2	31.42	1	F1:27261	20210611-08_2021021-OK192A_815_5.3.236.mgf	0		0	0	0	16	23	Carbamidomethylation	C1:Carbamidomethylation:1000.00	PEAKS DB
total 1 peptides																					

OK006_E_021_Con-ikot-ikot

back to list

| Protein Coverage | Supporting Peptides |

Protein Coverage:

16

1 KCCATKAYQC LRDHK**CFEPI DYK**CGQPCKN TDTTSCRPTA GDNCCMIYKH CLVECQIFRS DRRGDQLLYC YELCSDSCTY

81 T

C Carbamidomethylation (+57.02)

Supporting Peptides:

Peptide	Uniq	-10lgP	Mass	Length	ppm	m/z	z	RT	Fraction	Scan	Source File	Area ebraeus	Area judaeus	#Feature	#Feature ebraeus	#Feature judaeus	Start	End	PTM	AScore	Found By
K.C(+57.02)FEPIDYK.C	Y	82.56	1070.4742	8	4.6	536.2448	2	31.42	1	F1:27261	20210611-08_2021021-OK192A_815_5.3.236.mgf	0		0	0	0	16	23	Carbamidomethylation	C1:Carbamidomethylation:1000.00	PEAKS DB
total 1 peptides																					

JAB84712_Conodipine_Vc2_prepropeptide_Conus_victoriae

back to list

| Protein Coverage | Supporting Peptides |

Protein Coverage:

1 **QDSSTAELCK** INSNGCSVPS SWIPCQQHFL AACDRHDTCY LCGAHFNLTQ NDCDNAFLAH MIALCADGTD DEGFCLEKRK

81 RREASSMSLT TPLRQLRLLE KLMPLNSLWD HDSRQPQQRY IYDNCTDWAS TYYNTVQSFG SFYFYDTANA TYCPQFEPCM

161 PEVSSSA

Supporting Peptides:

Peptide	Uniq	-10lgP	Mass	Length	ppm	m/z	z	RT	Fraction	Scan	Source File	Area ebraeus	Area judaeus	#Feature	#Feature ebraeus	#Feature judaeus	Start	End	PTM	AScore	Found By
Q(-17.03)DSSTAELC(+57.02)K.I	Y	81.19	1120.4706	10	3.8	561.2426	2	20.93	1	F1:12489	20210611-08_2021021-OK192A_815_5.3.236.mgf	0		0	0	0	1	10		Q1:Pyro-glu from Q:1000.00;C9:Carbamidomethylation:1000.00	PEAKS DB
total 1 peptides																					

Pneo_78

back to list

| Protein Coverage | Supporting Peptides |

Protein Coverage:

1 QTDEEIPK**IG IIGAGIGGTS AAYFLR**QAFG DKLLVDIFEA ERVGGRLAVI TIDGQGYEAG G

Supporting Peptides:

Peptide	Uniq	-10lgP	Mass	Length	ppm	m/z	z	RT	Fraction	Scan	Source File	Area ebraeus	Area judaeus	#Feature	#Feature ebraeus	#Feature judaeus	Start	End	PTM	AScore	Found By
K.IGIIGAGIGGTSAAYFLR.Q	Y	79.22	1735.9620	18	-4.3	868.9809	2	55.60	2	F2:127079	20210611-09_2021021-OK160A_816_5.3.236.mgf		0	0	0	0	9	26			PEAKS DB
total 1 peptides																					

OK213_E_290_Insulin-related

back to list

| Protein Coverage | Supporting Peptides |

Protein Coverage:

1 MGLLRAVCQR IAMREVTPAL LALLCLLGQA TGQTFCGVDS RPHPKGICGH MLLRAHRTLC FLLSADYPNI FSRSIHKRSL

81 KNIDDFPLQT YAQMDLQGGH SQEQPTGTSP NTETMRKLFV SLFPR**VQTLL PDVGDSVLTQ GGPGSSK**GPS GDHLQAKRAG

161 DDLLPHVQKR GMVCDCCYNV CQPSTLAQYC P

Supporting Peptides:

Peptide	Uniq	-10lgP	Mass	Length	ppm	m/z	z	RT	Fraction	Scan	Source File	Area ebraeus	Area judaeus	#Feature	#Feature ebraeus	#Feature judaeus	Start	End	PTM	AScore	Found By
R.VQTLLPDVGDSVLTQGGPGSSK.G	Y	79.03	2154.1167	22	5.2	1078.0671	2	47.56	1	F1:45753	20210611-08_2021021-OK192A_815_5.3.236.mgf	0		0	0	0	126	147			PEAKS DB
total 1 peptides																					

gene:107291672

back to list

| Protein Coverage | Supporting Peptides |

Protein Coverage:

1 MPGPNPSATS VGASSRSPSK AVAPRATGST VRQRKNASCG TRNAGRSTSA GTGGMWR**FYT EDSPGLK**VGP VPVLVMSLLF

81 IASVFMLHIW GKYTRS

Supporting Peptides:

Peptide	Uniq	-10lgP	Mass	Length	ppm	m/z	z	RT	Fraction	Scan	Source File	Area ebraeus	Area judaeus	#Feature	#Feature ebraeus	#Feature judaeus	Start	End	PTM	AScore	Found By
total 1 peptides																					

Peptide	Uniq	-10lgP	Mass	Length	ppm	m/z	z	RT	Fraction	Scan	Source File	Area ebraeus	Area judaeus	#Feature	#Feature ebraeus	#Feature judaeus	Start	End	PTM	AScore	Found By
R.FYTEDSPGLK.V	Y	77.88	1155.5448	10	2.4	578.7786	2	26.10	2	F2:76464	20210611-09_2021021-OK160A_816_5.3.236.mgf	0	0	0	0	0	58	67			PEAKS DB
total 1 peptides																					

K_0010_325_Conkunitzin-12

back to list

| Protein Coverage | Supporting Peptides |

Protein Coverage:

1 SCDKAKDEGT GNRNITSYFF DSATATCETF FFK**GSGNNDP FPNR**FNSHVD CENICKCKLR KDMGTGTRHR IRYYHNTDGS

81 RCQSFIYYGM GGNTNNFQHD WECQNECEL

Supporting Peptides:

Peptide	Uniq	-10lgP	Mass	Length	ppm	m/z	z	RT	Fraction	Scan	Source File	Area ebraeus	Area judaeus	#Feature	#Feature ebraeus	#Feature judaeus	Start	End	PTM	AScore	Found By
K.GSGNNDPFPNR.F	Y	77.50	1173.5162	11	1.9	587.7643	2	20.32	1	F1:11930	20210611-08_2021021-OK192A_815_5.3.236.mgf	0		0	0	0	34	44			PEAKS DB
total 1 peptides																					

gene:107293627

back to list

| Protein Coverage | Supporting Peptides |

Protein Coverage:

1 MRSVGLTASG AEVLSSISLT CCFNMLVLVL LHFLAAGFGS PVTSQRSFEI DYSRNCFLKD GKPFR**YISGS IHYAR**IPQFY

81 WKDRLLKMKM AGLDAIQTYV PWNFHEPEPG VYNFRDDRDL EFFLKLADEI GLLVILRAGP YICAEWDMGG LPAWLLQKES

161 IVLRSSDSDY LAAVGRWMGI LLPKMKPHLY QNGGPIIMVQ VENEYGSYFA CDFHYLRYLK NLFRQYLGNE VVLFTTDGAT

241 KIFLRCGTLQ GLYSTVDFGP HTNVTAAFLS QRRTEPQGPL VNSEFYTGWL DHWGHTHNVI PSSAITKTLS EILAHGANVN

321 LYMFIGGTNF GYWNGANIPY MAQPTSYDYN APLSEAGDLT EKYFAVREVI GMYKDLPKGP IPPTTPKFAY GEVKLMKVGT

401 VTELLNDFSP SGPIKSMYPL TFVQLKQYFG YVLYRTKLPR NCSKEILFSF DGIRDRGYIS VDGIPQGILE RRKSLTLNIT

481 GNAGANLDLL IENMGRVNYG RYNNDFKGLI ANLTLAKDIL LDWEIYPLDI DRAVQEGLSH KISGRNISTY EGSNVYTGNF

561 SIPSGIPDLP QDTYLKFPGW TKGQVWINGF NLGRYWPSLG PQLTLFVPRN ILVSSSPNNI TILELERSPC GIQNCLVEFV

641 DKPILNETLD YEGNFEKLFS KDL

Supporting Peptides:

Peptide	Uniq	-10lgP	Mass	Length	ppm	m/z	z	RT	Fraction	Scan	Source File	Area ebraeus	Area judaeus	#Feature	#Feature ebraeus	#Feature judaeus	Start	End	PTM	AScore	Found By
R.YISGSIHYAR.I	Y	77.33	1165.5880	10	1.8	583.7999	2	22.11	2	F2:69489	20210611-09_2021021-OK160A_816_5.3.236.mgf		0	0	0	0	66	75			PEAKS DB
total 1 peptides																					

Pvau2_11

back to list

| Protein Coverage | Supporting Peptides |

Protein Coverage:

1 PDDTKCGYES CNPVKDDMIN VHLVPHTHDD VGWLVTVDQY YYEHVQYILD SVIPELVNDP SKRFIYVEIA FFERWFREQT

81 DSMRHIVKKL VNEGRLEFIL GGWCMNDEAA THYNAIIDQH TLGFEYLREN FGKCGKPRVA WQIDPFGHSR EHASIFARFG

161 FDSLFFGRLD YEDKENRENH KTMEMIWRGS PDNLGTPADL FTGALPNGYN PPDGFCFDIF CGDIPIVDDT RLHDVNVKEK

241 VDTFLKEMAE QATHYATNHL ITTMGSDFQY QAAHNWYLNL DKLIKYVNAR QSNGSKVNLL YSTPSCYTYH VNKANKTWTT

321 KEDDFFPYAH RPHSFWTGYF TSRPTLKGYI RRTNNFLQVV KQLDALAMLE DTDNSTFNIE VLAK**AMGVAQ HHDAVSGTAK**

401 QVVTYDYEER LANGIMEGQK VYNDAMKKLE VQSGGVKMDH YFCTLLNISV CSVSEKGNQF MVTVYNPIAR PGFYYVRLPV

481 SSVQNVAFSV MGPDGKQVAS ELIPETPDTF RIPERMGDTS KYELVFPVSL VPLGFTTFLV SRSKGTLCDD ALSVLWMIVR

561 VRIHEMSL

Supporting Peptides:

Peptide	Uniq	-10lgP	Mass	Length	ppm	m/z	z	RT	Fraction	Scan	Source File	Area ebraeus	Area judaeus	#Feature	#Feature ebraeus	#Feature judaeus	Start	End	PTM	AScore	Found By
K.AMGVAQHHDAVSGTAK.Q	Y	76.52	1578.7572	16	-3.2	527.2558	3	12.94	2	F2:56008	20210611-09_2021021-OK160A_816_5.3.236.mgf		0	0	0	0	385	400			PEAKS DB
total 1 peptides																					

Pvau2_17

back to list

| Protein Coverage | Supporting Peptides |

Protein Coverage:

1 PDDTKCGYES CNPVKDDMIN VHLVPHTHDD VGWLVTVDQY YYEHVQYILD SVIPELVNDP SKRFIYVEIA FFERWFREQT

81 DSMRHIVKKL VNEGRLEFIL GGWCMNDEAA THYNAIIDQH TLGFEYLREN FGKCGKPRVA WQIDPFGHSR EHASIFARFG

161 FDSLFFGRLD YEDKENRENH KTMEMIWRGS PDNLGTPADL FTGALPNGYN PPDGFCFDIF CGDIPIVDDT RLHDVNVKEK

241 VDTFLKEMAE QATHYATNHL ITTMGSDFQY QAAHNWYLNL DKLIKYVNAR QSNGSKVNLL YSTPSCYTYH VNKANKTWTT

321 KEDDFFPYAH RPHSFWTGYF TSRPTLKGYI RRTNNFLQVV KQLDALAMLE DTDNSTFNIE VLAK**AMGVAQ HHDAVSGTAK**

401 QVVTYDYEER LANGIMEGQK VYNDAMKKLE VQSGGVKMDH YFCTLLNISV CSVSEKGNQF MVTVYNPIAR PGFYYVRLPV

481 SSVQNVAFSV MGPDGKQVAS ELIPETPDTF RIPERMGDTS KYELVFPVSL VPLGFTTFLV SRSKDKRAKN QQREVTPMNE

561 EDIVIQNEYI AVAFSGKTGT LKNMTNLEKK ISIDLTQSML YYLGDAGNNS QPQFQASGAY IFRPNGTSPI GMGNGRAKLR

641 SKAYFVKGEL VQEVYQEFTP WMTQTVKLYS QQCFVEFQWT VGPIPIQDGR GKEVVSRFES SLNNSEIFYT DANGREILTR

721 QLNHRDTWNF NNTEPIAGNY YPVNSRIFIR DMAKKVQLTV LTDRSQGGTS LQDGQIELMV HRRLLHDDAL GVDQALNETG

801 CDGKGLIVRG SHYLMLEPIA SAAAGHRDLA ERLYMAPQIS FSMPTSKQVA TNWSGVHQAL PDNVHLLTLE QFPSKGPVPS

881 DKQPFLLRLE HFYETGEDPV LSKPATVHLQ KLFRTFDIVK ATELTLGADM ALADL

Supporting Peptides:

Peptide	Uniq	-10lgP	Mass	Length	ppm	m/z	z	RT	Fraction	Scan	Source File	Area ebraeus	Area judaeus	#Feature	#Feature ebraeus	#Feature judaeus	Start	End	PTM	AScore	Found By
K.AMGVAQHHDAVSGTAK.Q	Y	76.52	1578.7572	16	-3.2	527.2558	3	12.94	2	F2:56008	20210611-09_2021021-OK160A_816_5.3.236.mgf		0	0	0	0	385	400			PEAKS DB
total 1 peptides																					

K_0010_003_Con-ikot-ikot-4

back to list

| Protein Coverage | Supporting Peptides |

Protein Coverage:

1 QCCAIKSYQC LQLHECLTPI DSACAVQCKT TDTSSCGSPP SDNCCSTYKS CLVDCQISR**G DQVEDPLR**VC YNQCDQEHPC

Supporting Peptides:

Peptide	Uniq	-10lgP	Mass	Length	ppm	m/z	z	RT	Fraction	Scan	Source File	Area ebraeus	Area judaeus	#Feature	#Feature ebraeus	#Feature judaeus	Start	End	PTM	AScore	Found By
total 1 peptides																					

Peptide	Uniq	-10lgP	Mass	Length	ppm	m/z	z	RT	Fraction	Scan	Source File	Area ebraeus	Area judaeus	#Feature	#Feature ebraeus	#Feature judaeus	Start	End	PTM	AScore	Found By
R.GDQVEDPLR.V	Y	76.39	1027.4934	9	3.9	514.7540	2	24.34	1	F1:17231	20210611-08_2021021-OK192A_815_5.3.236.mgf	0		0	0	0	60	68			PEAKS DB
total 1 peptides																					

V_1278_027_I1-1

back to list

| Protein Coverage | Supporting Peptides |

Protein Coverage:

22 23
C C

1 QFSCSEAGET CMTYSSCCSR **FCCEGSLGK**R CARLSCDDIG V

C C

C Carbamidomethylation (+57.02)

Supporting Peptides:

Peptide	Uniq	-10lgP	Mass	Length	ppm	m/z	z	RT	Fraction	Scan	Source File	Area ebraeus	Area judaeus	#Feature	#Feature ebraeus	#Feature judaeus	Start	End	PTM	AScore	Found By
R.FC(+57.02)C(+57.02)EGSLGK.R	Y	75.34	1056.4369	9	0.9	529.2242	2	19.81	1	F1:11260	20210611-08_2021021-OK192A_815_5.3.236.mgf	0		0	0	0	21	29	Carbamidomethylation	C2:Carbamidomethylation:1000.00;C3:Carbamidomethylation:1000.00	PEAKS DB
total 1 peptides																					

V_1302_121_I1-1

back to list

| Protein Coverage | Supporting Peptides |

Protein Coverage:

22 23
C C

1 QFGCSEAGET CMNYSSCCSR **FCCEGSLGK**R CARLNCDDID I

C C

C Carbamidomethylation (+57.02)

Supporting Peptides:

Peptide	Uniq	-10lgP	Mass	Length	ppm	m/z	z	RT	Fraction	Scan	Source File	Area ebraeus	Area judaeus	#Feature	#Feature ebraeus	#Feature judaeus	Start	End	PTM	AScore	Found By
R.FC(+57.02)C(+57.02)EGSLGK.R	Y	75.34	1056.4369	9	0.9	529.2242	2	19.81	1	F1:11260	20210611-08_2021021-OK192A_815_5.3.236.mgf	0		0	0	0	21	29	Carbamidomethylation	C2:Carbamidomethylation:1000.00;C3:Carbamidomethylation:1000.00	PEAKS DB
total 1 peptides																					

V_1258_040_I1-1

back to list

| Protein Coverage | Supporting Peptides |

Protein Coverage:

22 23
C C

1 QFGCSEAGET CMNYSSCCSR **FCCEGSLGK**R CARLNCDDID I

C C

C Carbamidomethylation (+57.02)

Supporting Peptides:

Peptide	Uniq	-10lgP	Mass	Length	ppm	m/z	z	RT	Fraction	Scan	Source File	Area ebraeus	Area judaeus	#Feature	#Feature ebraeus	#Feature judaeus	Start	End	PTM	AScore	Found By
R.FC(+57.02)C(+57.02)EGSLGK.R	Y	75.34	1056.4369	9	0.9	529.2242	2	19.81	1	F1:11260	20210611-08_2021021-OK192A_815_5.3.236.mgf	0		0	0	0	21	29	Carbamidomethylation	C2:Carbamidomethylation:1000.00;C3:Carbamidomethylation:1000.00	PEAKS DB
total 1 peptides																					

A_0855_013_I1-1

back to list

| Protein Coverage | Supporting Peptides |

Protein Coverage:

22 23
C C

1 QFGCSEAGET CKTYSSCCSR **FCCEGSLGK**R CARLSCDDIG V

C C

C Carbamidomethylation (+57.02)

Supporting Peptides:

Peptide	Uniq	-10lgP	Mass	Length	ppm	m/z	z	RT	Fraction	Scan	Source File	Area ebraeus	Area judaeus	#Feature	#Feature ebraeus	#Feature judaeus	Start	End	PTM	AScore	Found By
R.FC(+57.02)C(+57.02)EGSLGK.R	Y	75.34	1056.4369	9	0.9	529.2242	2	19.81	1	F1:11260	20210611-08_2021021-OK192A_815_5.3.236.mgf	0		0	0	0	21	29	Carbamidomethylation	C2:Carbamidomethylation:1000.00;C3:Carbamidomethylation:1000.00	PEAKS DB
total 1 peptides																					

A_0885_033_I1-1

back to list

| Protein Coverage | Supporting Peptides |

Protein Coverage:

22 23
C C

1 QFGCSEAGET CKTYSSCCSR **FCCEGSLGK**R CARLSCDDIG V

C C

C Carbamidomethylation (+57.02)

Supporting Peptides:

Peptide	Uniq	-10lgP	Mass	Length	ppm	m/z	z	RT	Fraction	Scan	Source File	Area ebraeus	Area judaeus	#Feature	#Feature ebraeus	#Feature judaeus	Start	End	PTM	AScore	Found By
R.FC(+57.02)C(+57.02)EGSLGK.R	Y	75.34	1056.4369	9	0.9	529.2242	2	19.81	1	F1:11260	20210611-08_2021021-OK192A_815_5.3.236.mgf	0		0	0	0	21	29	Carbamidomethylation	C2:Carbamidomethylation:1000.00;C3:Carbamidomethylation:1000.00	PEAKS DB
total 1 peptides																					

A_0031_321_I1-1

back to list

| Protein Coverage | Supporting Peptides |

Protein Coverage:

22 23
C C

1 QFSCSEAGET CKTYSSCCSR **FCCEGSLGK**R CARLSCDDIG V

C C

C Carbamidomethylation (+57.02)

Supporting Peptides:

Peptide	Uniq	-10lgP	Mass	Length	ppm	m/z	z	RT	Fraction	Scan	Source File	Area ebraeus	Area judaeus	#Feature	#Feature ebraeus	#Feature judaeus	Start	End	PTM	AScore	Found By
R.FC(+57.02)C(+57.02)EGSLGK.R	Y	75.34	1056.4369	9	0.9	529.2242	2	19.81	1	F1:11260	20210611-08_2021021-OK192A_815_5.3.236.mgf	0		0	0	0	21	29	Carbamidomethylation	C2:Carbamidomethylation:1000.00;C3:Carbamidomethylation:1000.00	PEAKS DB

total 1 peptides

A_0055_009_I1-1

back to list

| Protein Coverage | Supporting Peptides |

Protein Coverage:

22 23

1 QFGCSEAGET CKTYSSCCSR **FCCEGSLGK**R CARLSCDDIG V

C Carbamidomethylation (+57.02)

Supporting Peptides:

Peptide	Uniq	-10lgP	Mass	Length	ppm	m/z	z	RT	Fraction	Scan	Source File	Area ebraeus	Area judaeus	#Feature	#Feature ebraeus	#Feature judaeus	Start	End	PTM	AScore	Found By
R.FC(+57.02)C(+57.02)EGSLGK.R	Y	75.34	1056.4369	9	0.9	529.2242	2	19.81	1	F1:11260	20210611-08_2021021-OK192A_815_5.3.236.mgf	0		0	0	0	21	29	Carbamidomethylation	C2:Carbamidomethylation:1000.00;C3:Carbamidomethylation:1000.00	PEAKS DB

total 1 peptides

AXL95521_conotoxin_precursor_superfamily_I1_Conus_ermineus

back to list

| Protein Coverage | Supporting Peptides |

Protein Coverage:

22 23

1 QFGCSEAGET CKTYSSCCSR **FCCEGSLGK**R CARLSCDDIG V

C Carbamidomethylation (+57.02)

Supporting Peptides:

Peptide	Uniq	-10lgP	Mass	Length	ppm	m/z	z	RT	Fraction	Scan	Source File	Area ebraeus	Area judaeus	#Feature	#Feature ebraeus	#Feature judaeus	Start	End	PTM	AScore	Found By
R.FC(+57.02)C(+57.02)EGSLGK.R	Y	75.34	1056.4369	9	0.9	529.2242	2	19.81	1	F1:11260	20210611-08_2021021-OK192A_815_5.3.236.mgf	0		0	0	0	21	29	Carbamidomethylation	C2:Carbamidomethylation:1000.00;C3:Carbamidomethylation:1000.00	PEAKS DB

total 1 peptides

V_CG13_054_I1-1

back to list

| Protein Coverage | Supporting Peptides |

Protein Coverage:

22 23

1 QFDCSEAGET CMNYSSCCSR **FCCEGSLGK**R CARLSCDDIG V

C Carbamidomethylation (+57.02)

Supporting Peptides:

Peptide	Uniq	-10lgP	Mass	Length	ppm	m/z	z	RT	Fraction	Scan	Source File	Area ebraeus	Area judaeus	#Feature	#Feature ebraeus	#Feature judaeus	Start	End	PTM	AScore	Found By
R.FC(+57.02)C(+57.02)EGSLGK.R	Y	75.34	1056.4369	9	0.9	529.2242	2	19.81	1	F1:11260	20210611-08_2021021-OK192A_815_5.3.236.mgf	0		0	0	0	21	29	Carbamidomethylation	C2:Carbamidomethylation:1000.00;C3:Carbamidomethylation:1000.00	PEAKS DB

total 1 peptides

A_0855_014_I1-1

back to list

| Protein Coverage | Supporting Peptides |

Protein Coverage:

24 25

1 HRQFGCSEAG ETCKTYSSCC SR**FCCEGSLG K**RCARLSCDD IGV

C Carbamidomethylation (+57.02)

Supporting Peptides:

Peptide	Uniq	-10lgP	Mass	Length	ppm	m/z	z	RT	Fraction	Scan	Source File	Area ebraeus	Area judaeus	#Feature	#Feature ebraeus	#Feature judaeus	Start	End	PTM	AScore	Found By
R.FC(+57.02)C(+57.02)EGSLGK.R	Y	75.34	1056.4369	9	0.9	529.2242	2	19.81	1	F1:11260	20210611-08_2021021-OK192A_815_5.3.236.mgf	0		0	0	0	23	31	Carbamidomethylation	C2:Carbamidomethylation:1000.00;C3:Carbamidomethylation:1000.00	PEAKS DB

total 1 peptides

A_0520_205_I1-1

back to list

| Protein Coverage | Supporting Peptides |

Protein Coverage:

29 30

1 ALLKGDWQFG CSEAGETCKT YSSCCSR**FCC EGSLGK**RCAR LSCDDIGV

C Carbamidomethylation (+57.02)

Supporting Peptides:

Peptide	Uniq	-10lgP	Mass	Length	ppm	m/z	z	RT	Fraction	Scan	Source File	Area ebraeus	Area judaeus	#Feature	#Feature ebraeus	#Feature judaeus	Start	End	PTM	AScore	Found By
R.FC(+57.02)C(+57.02)EGSLGK.R	Y	75.34	1056.4369	9	0.9	529.2242	2	19.81	1	F1:11260	20210611-08_2021021-OK192A_815_5.3.236.mgf	0		0	0	0	28	36	Carbamidomethylation	C2:Carbamidomethylation:1000.00;C3:Carbamidomethylation:1000.00	PEAKS DB

total 1 peptides

CV8_288_Protein

back to list

| Protein Coverage | Supporting Peptides |

Protein Coverage:

1 MALPWKALLF ASFTLLYVIP FRFGAKQNKK DLIIRVDDFK DFKKLLRTRT NLLVIFTQSE KAASKSLALF EEVAEEMRGK
81 ATMAYINCGD DKKLCKKLKV YTTTFELKHY KDGEFNKNYD RKLTVKSMVN FLMDPTGDIP WDEDLTATDV VHIDTEDGLN
161 KLLKKDKSAA LVMFYAPWCG YCKRLKPDFA AAATEVKGKA NLVGIDVDKP HMMGLRTLYN ITGFPTLYYF EKGKVKYRYG
241 GENSKDGIVS WLSNPKPPEE PKKEAEWAEE PSEVVHLEDD NFDDYIKEHP SVLVMFYAPW CGHCKKMKPG YTEAAQLIKD
321 QEIPSVLAAV DATKHRKLAD TYNIKGFPTL KYFKDGEFAF DVNERDKDKI IDFVKDPKEP PPPPAPEPSW EEVDSDVIHL
401 NEENFKPTLK KRKHALIMFY APWCGHCKKA KPEFMAAAAK FRDDGKVMFG AVDCTTQQGI CSAHDVTGYP TFKYFNYNKN
481 SQKYMGGREE NDFVNFMRDP LSPSPSPPTA STKPEEDWAD IPGKDFIGFL TKDTFDSFVL SQGSVLVMFY APWCGHCKRM
561 KPAFGQAASV IVEEKLGALA AVDATIEQQL ADRFEIRGYP TLKYFKGGKY AFEYSGGR**TA DDFIAFLR**NP DAPPTTPSPE
641 AQWSDIVSDV HHLTDTSFDE FIAANKKTLV MFYSPGCGHC KKMKPDYMTA ATRLKEKAPD ARLAAVDATR FKDLARKYHI
721 KGYPTLKYFE DGALKADYKS GRSAEDLVEF FSTASDKKDE L

Supporting Peptides:

Peptide	Uniq	-10lgP	Mass	Length	ppm	m/z	z	RT	Fraction	Scan	Source File	Area ebraeus	Area judaeus	#Feature	#Feature ebraeus	#Feature judaeus	Start	End	PTM	AScore	Found By
R.TADDFIAFLR.N	Y	74.95	1167.5924	10	-3.5	584.7990	2	52.65	2	F2:123233	20210611-09_2021021-OK160A_816_5.3.236.mgf		0	0	0	0	619	628			PEAKS DB
total 1 peptides																					

OK213_E_328_PDI

back to list

| Protein Coverage | Supporting Peptides |

Protein Coverage:

1 MALPWKGLLF ASFTLLYVIP FRFGAKQNKK DLIIRVDDFK DFKKLLRTRT NLLVIFTQSE KAASKSLALF EEVAEEMRGK
81 ATMAYINCGD DKKLCKKLKV YTTTFELKHY KDGEFNKNYD RKLTVKSMVN FLMDPTGDIP WDEDLTATDV VHIDTEDGLN
161 KLLKKDKSAA LVMFYAPWCG YCKRLKPDFA AAATEVKGKA NLVGIDVDKP HMMGLRTLYN ITGFPTLYYF EKGKVKYRYG
241 GENSKEGIVS WLSNPKPPEE PKKEAEWAEE PSEVVHLEDD NFDDYIKEHP SVLVMFYAPW CGHCKKMKPG YTEAAQLIKD
321 QEIPSILAAV DATKHRKLAE TYNIKGFPTL KYFKEGEFAF DVNERDKDKI IDFVKDPKEP PPPPAPEPSW EEVDSDVIHL
401 NEENFKPTLK KRKHALIMFY APWCGHCKKA KPEFMAAAAK FRDDGKVMFG AVDCTTQQGV CSAHDVTGYP TFKYFNYNKN
481 SQKYMGGREE NDFVNFMRDP LSPSPSPPTA STKPEEDWAD IPGKDLIGFL TKDTFDSFVL SQGSVLVMFY APWCGHCKRM
561 KPAFGQAASV IVEEKLGALA AVDATIEQQL ADRFEIRGYP TLKYFRDGKY AFEYSGGR**TA DDFIAFLR**NP DAPPTTPSPE
641 PQWSDIVSDV HHLTDTSFDE FIAANKKTLV MFYSPGCGHC KKMKPDYMTA ATRLKEKVPD ARLAAVDATK FKDLAHKYHI
721 KGYPTLKYFE DGAFKADYKS GRSAEDLVEF FSMASDKKDE L

Supporting Peptides:

Peptide	Uniq	-10lgP	Mass	Length	ppm	m/z	z	RT	Fraction	Scan	Source File	Area ebraeus	Area judaeus	#Feature	#Feature ebraeus	#Feature judaeus	Start	End	PTM	AScore	Found By
R.TADDFIAFLR.N	Y	74.95	1167.5924	10	-3.5	584.7990	2	52.65	2	F2:123233	20210611-09_2021021-OK160A_816_5.3.236.mgf		0	0	0	0	619	628			PEAKS DB
total 1 peptides																					

OK006_E_297_PDI

back to list

| Protein Coverage | Supporting Peptides |

Protein Coverage:

1 MALPWKALLF ASFTLLYVIP FRFGAKQNKK DLIIRVDDFK DFKKLLRTRT NLLVIFTQSE KAASKSLALF EEVAEEMRGK
81 ATMAYINCGD DKKLCKKLKV YTTTFELKHY KDGEFNKNYD RKLTVKSMVN FLMDPTGDIP WDEDLTATDV VHIDTEDGLN
161 KLLKKDKSAA LVMFYAPWCG YCKRLKPDFA AAATEVKGKA NLVGIDVDKP HMMGLRTLYN ITGFPTLYYF EKGKVKYRYG
241 GENSKEGIVS WLSNPKPPEE PKKEAEWAEE PSEVVHLEDD NFDDYIKEHP SVLVMFYAPW CGHCKKMKPG YTEAAQLIKD
321 QEIPSILAAV DATKHRKLAE TYNIKGFPTL KYFKEGEFAF DVNERDKDKI IDFVKDPKEP PPPPAPEPSW EEVDSDVIHL
401 NEENFKPTLK KRKHALIMFY APWCGHCKKA KPEFMAAAAK FRDDGKVMFG AVDCTTQQGV CSAHDVTGYP TFKYFNYNKN
481 SQKYMGGREE NDFVNFMRDP LSPSPSPPTA STKPEEDWAD IPGKDLIGFL TKDTFDSFVL SQGSVLVMFY APWCGHCKRM
561 KPAFGQAASV IVEEKLGALA AVDATIEQQL ADRFEIRGYP TLKYFRDGKY AFEYSGGR**TA DDFIAFLR**NP DAPPTTPSPE
641 PQWSDIVSDV HHLTDTSFDE FIAANKKTLV MFYSPGCGHC KKMKPDYMTA ATRLKEKVPD ARLAAVDATK FKDLAHKYHI
721 KGYPTLKYFE DGAFKADYKS GRSAEDLVEF FSMASDKKDE L

Supporting Peptides:

Peptide	Uniq	-10lgP	Mass	Length	ppm	m/z	z	RT	Fraction	Scan	Source File	Area ebraeus	Area judaeus	#Feature	#Feature ebraeus	#Feature judaeus	Start	End	PTM	AScore	Found By
R.TADDFIAFLR.N	Y	74.95	1167.5924	10	-3.5	584.7990	2	52.65	2	F2:123233	20210611-09_2021021-OK160A_816_5.3.236.mgf		0	0	0	0	619	628			PEAKS DB
total 1 peptides																					

K_0010_438_Protein

back to list

| Protein Coverage | Supporting Peptides |

Protein Coverage:

1 MRGKATMAYI NCGDDKKLCK KLKVYTTTFE LKHYKDGEFN KNYDRKLTVK SMVNFLMDPT GDIPWDEDLT ATDVVHIDTE
81 DGLNKLLKKD KSAALVMFYA PWCGYCKRLK PDFAAAATEV KGKANLVGID VDKPHMMGLR TLYNITGFPT LYYFEKGKVK
161 YRFGGENSKE GIVSWLSNPK PPEEPKKEAE WAEEPSEVVH LEDDNFDDYI KEHPSVLVMF YAPWCGHCKK MKPGYTEAAQ
241 LIKDQEIPSV LAAVDATKHR KLAETYNIKG FPTLKYFKEG EFAFDVNERD KDKIIDFVKD PKEPPPPPAP EPSWEEVDSD
321 VIHLNEENFK PTLKKRKHAL IMFYAPWCGH CKKAKPEFMA AAAKFRDDGK VMFGAVDCTT QQGICSAHDV TGYPTFKYFN
401 YNKNSQKYMG GREENDFVNF MRDPLSPSPS PPTASTKPEE DWADIPGKDL IGFLTKDTFD SFVLSQGSVL VMFYAPWCGH
481 CKRMKPAFGQ AASVIVEEKL GALAAVDATI EQQLADRFEI RGYPTLKYFK GGKYAFEYSG GR**TADDFIAF LR**NPDAPPTT
561 PSPEPQWSDI VSDVHHLTDT SFDEFIAANK KTLVMFYSPG CGHCKKMKPD YMTAATRLKE KVPDARLAAV DATRFKDLAR
641 KYHIKGYPTL KYFEDGAFKA DYKSGRSAED LVEFFSTASD KKDEL

Supporting Peptides:

Peptide	Uniq	-10lgP	Mass	Length	ppm	m/z	z	RT	Fraction	Scan	Source File	Area ebraeus	Area judaeus	#Feature	#Feature ebraeus	#Feature judaeus	Start	End	PTM	AScore	Found By
R.TADDFIAFLR.N	Y	74.95	1167.5924	10	-3.5	584.7990	2	52.65	2	F2:123233	20210611-09_2021021-OK160A_816_5.3.236.mgf		0	0	0	0	543	552			PEAKS DB
total 1 peptides																					

V_1258_178_Protein

back to list

| Protein Coverage | Supporting Peptides |

Protein Coverage:

```
  1  MVNFLMDPTG DIPWDEDLTA TDVVHIDTED GLNKLLKKDK SAALVMFYAP WCGYCKRLKP DFAAAATEVK GKANLVGIDV
 81  DKPHMMGLRT LYNITGFPTL YYFEKGKVKY RYGGENSKDG IVSWLSNPKP PEEPKKEAEW AEEPSEVVHL EDDNFDDYIK
161  EHPSVLVMFY APWCGHCKKM KPGYTEAAQL IKDQEIPSVL AAVDATKHRK LADTYNIKGF PTLKYFKDGE FAFDVNERDK
241  DKIIDFVKDP KEPPPPPAPE PSWEEVDSDV IHLNEENFKP TLKKRKHALI MFYAPWCGHC KKAKPEFMAA AAKFRDDGKV
321  MFGAVDCTTQ QGICSAHDVT GYPTFKYFNY NKNSQKYMGG REENDFVNFM RDPLSPSPSP PTASTKPEED WADIPGKDFI
401  GFLTKDTFDS FVLSQGSVLV MFYAPWCGHC KRMKPAFGQA ASMIVEEKLG ALAAVDATIE QQLADRFEIR GYPTLKYFKG
481  GKYAFEYSGG RTADDFIAFL RNPDAPPTTP SPEPQWSDIV SDVHHLTDTS FDEFIAANKK TLVMFYSPGC GHCKKMKPDY
561  MTAATRLKEK VPDARLAAVD ATRFKDLARK YHIKGYPTLK YFEDGALKAD YKSGRSAEDL VEFFSTASDK KDEL
```

Supporting Peptides:

Peptide	Uniq	-10lgP	Mass	Length	ppm	m/z	z	RT	Fraction	Scan	Source File	Area ebraeus	Area judaeus	#Feature	#Feature ebraeus	#Feature judaeus	Start	End	PTM	AScore	Found By
R.TADDFIAFLR.N	Y	74.95	1167.5924	10	-3.5	584.7990	2	52.65	2	F2:123233	20210611-09_2021021-OK160A_816_5.3.236.mgf		0	0	0	0	492	501			PEAKS DB
total 1 peptides																					

V_1302_231_Protein

back to list

| Protein Coverage | Supporting Peptides |

Protein Coverage:

```
  1  MVNFLMDPTG DIPWDEDLTA TDVVHIDTED GLNKLLKKDK SAALVMFYAP WCGYCKRLKP DFAAAATEVK GKANLVGIDV
 81  DKPHMMGLRT LYNITGFPTL YYFEKGKVKY RYGGENSKDG IVSWLSNPKP PEEPKKEAEW AEEPSEVVHL EDDNFDDYIK
161  EHPSVLVMFY APWCGHCKKM KPGYTEAAQL IKDQEIPSVL AAVDATKHRK LADTYNIKGF PTLKYFKDGE FAFDVNERDK
241  DKIIDFVKDP KEPPPPPAPE PSWEEVDSDV IHLNEENFKP TLKKRKHALI MFYAPWCGHC KKAKPEFMAA AAKFRDDGKV
321  MFGAVDCTTQ QGICSAHDVT GYPTFKYFNY NKNSQKYMGG REENDFVNFM RDPLSPSPSP PTASTKPEED WADIPGKDFI
401  GFLTKDTFDS FVLSQGSVLV MFYAPWCGHC KRMKPAFGQA ASMIVEEKLG ALAAVDATIE QQLADRFEIR GYPTLKYFKG
481  GKYAFEYSGG RTADDFIAFL RNPDAPPTTP SPEPQWSDIV SDVHHLTDTS FDEFIAANKK TLVMFYSPGC GHCKKMKPDY
561  MTAATRLKEK VPDARLAAVD ATRFKDLARK YHIKGYPTLK YFEDGALKAD YKSGRSAEDL VEFFSTASDK KDEL
```

Supporting Peptides:

Peptide	Uniq	-10lgP	Mass	Length	ppm	m/z	z	RT	Fraction	Scan	Source File	Area ebraeus	Area judaeus	#Feature	#Feature ebraeus	#Feature judaeus	Start	End	PTM	AScore	Found By
R.TADDFIAFLR.N	Y	74.95	1167.5924	10	-3.5	584.7990	2	52.65	2	F2:123233	20210611-09_2021021-OK160A_816_5.3.236.mgf		0	0	0	0	492	501			PEAKS DB
total 1 peptides																					

CebrOK033_J_269_PDI

back to list

| Protein Coverage | Supporting Peptides |

Protein Coverage:

```
  1  FKDFKKLLRT RTNLLVIFTQ SEKAASKSLA LFEEVAEEMR GKATMAYINC GDDKKLCKKL KVYTTTFELK HYKDGEFNKN
 81  YDRKLTVKSM VNFLMDPTGD IPWDEDLTAT DVVHIDTEDG LNKLLKKDKS AALVMFYAPW CGYCKRLKPD FAAAATEVKG
161  KANLVGIDVD KPHMMGLRTL YNITGFPTLY YFEKGKVKYR YGGENSKEGI VSWLSNPKPP EEPKKEAEWA EEPSEVVHLE
241  DDNFDDYIKE HPSVLVMFYA PWCGHCKKMK PGYTEAAQLI KDQEIPSVLA AVDATKHRKL AETYNIKGFP TLKYFKEGEF
321  AFDVNERDKD KIIDFVKDPK EPPPPPAPEP SWEEVDSDVI HLNEENFKPT LKKRKHALIM FYAPWCGHCK KAKPEFMAAA
401  AKFRDDGKVM FGAVDCTTQQ GVCSAHDVTG YPTFKYFNYN KNSQKYMGGR EENDFVNFMR DPLSPSPSPP TASTKPEEDW
481  ADIPGKDLIG FLTKDTFDSF VLSQGSVLVM FYAPWCGHCK RMKPAFGQAA SVIVEEKLGA LAAVDATIEQ QLADRFEIRG
561  YPTLKYFRDG KYAFEYSGGR TADDFIAFLR NPDAPPTTPS PEPQWSDIVS DVHHLTDTSF DEFIAANKKT LVMFYSPGCG
641  HCKKMKPDYM TAATRLKEKV PDARLAAVDA TRFKDLARKY HIKGYPTLKY FEDGAFKADY KSGRSAEDLV EFFSMASDKK
721  DEL
```

Supporting Peptides:

Peptide	Uniq	-10lgP	Mass	Length	ppm	m/z	z	RT	Fraction	Scan	Source File	Area ebraeus	Area judaeus	#Feature	#Feature ebraeus	#Feature judaeus	Start	End	PTM	AScore	Found By
R.TADDFIAFLR.N	Y	74.95	1167.5924	10	-3.5	584.7990	2	52.65	2	F2:123233	20210611-09_2021021-OK160A_816_5.3.236.mgf		0	0	0	0	581	590			PEAKS DB
total 1 peptides																					

SRX1323887_E_250_PDI

back to list

| Protein Coverage | Supporting Peptides |

Protein Coverage:

```
  1  MALPWKALLF ASFTLLYVIP FRFGAKQNKK DLIIRVDDFK DFKKLLRTRT NLLVIFTQSE KAASKSLALF EEVAEEMRGK
 81  ATMAYINCGD DKKLCKKLKV YTTTFELKHY KDGEFNKNYD RKLTVKSMVN FLMDPTGDIP WDEDLTATDV VHIDTEDGLN
161  KLLKKDKSAA LVMFYAPWCG YCKRLKPDFA AAATEVKGKA NLVGIDVDKP HMMGLRTLYN ITGFPTLYYF EKGKVKYRYG
241  GENSKEGIVS WLSNPKPPEE PKKEAEWAEE PSEVVHLEDD NFDDYIKEHP SVLVMFYAPW CGHCKKMKPG YTEAAQLIKD
321  QEIPSVLAAV DATKHRKLAE TYNIKGFPTL KYFKEGEFAF DVNERDKDKI IDFVKDPKEP PPPPAPEPSW EEVDSDVIHL
401  NEENFKPTLK KRKHALIMFY APWCGHCKKA KPEFMAAAAK FRDDGKVMFG AVDCTTQQGV CSAHDVTGYP TFKYFNYNKN
481  SQKYMGGREE NDFVNFMRDP LSPSPSPPTA STKPEEDWAD IPGKDLIGFL TKDTFDSFVL SQGSVLVMFY APWCGHCKRM
561  KPAFGQAASV IVEEKLGALA AVDATIEQQL ADRFEIRGYP TLKYFRDGKY AFEYSGGRTA DDFIAFLRNP DAPPTTPSPE
641  PQWSDIVSDV HHLTDTSFDE FIAANKKTLV MFYSPGCGHC KKMKPDYMTA ATRLKEKVPD ARLAAVDATK FKDLAHKYHI
721  KGYPTLKYFE DGAFKADYKS GRSAEDLVEF FSMASDKKDE L
```

Supporting Peptides:

Peptide	Uniq	-10lgP	Mass	Length	ppm	m/z	z	RT	Fraction	Scan	Source File	Area ebraeus	Area judaeus	#Feature	#Feature ebraeus	#Feature judaeus	Start	End	PTM	AScore	Found By
R.TADDFIAFLR.N	Y	74.95	1167.5924	10	-3.5	584.7990	2	52.65	2	F2:123233	20210611-09_2021021-OK160A_816_5.3.236.mgf		0	0	0	0	619	628			PEAKS DB
total 1 peptides																					

OK214_J_339_PDI

back to list

| Protein Coverage | Supporting Peptides |

Protein Coverage:

1 MALPWKALLF ASFTLLYVIP FRFGAKQNKK DLIIRVDDFK DFKKLLRTRT NLLVIFTQSE KAASKSLALF EEVAEEMRGK
81 ATMAYINCGD DKKLCKKLKV YTTTFELKHY KDGEFNKNYD RKLTVKSMVN FLMDPTGDIP WDEDLTATDV VHIDTEDGLN
161 KLLKKDKSAA LVMFYAPWCG YCKRLKPDFA AAATEVKGKA NLVGIDVDKP HMMGLRTLYN ITGFPTLYYF EKGKVKYRYG
241 GENSKEGIVS WLSNPKPPEE PKKEAEWAEE PSEVVHLEDD NFDDYIKEHP SVLVMFYAPW CGHCKKMKPG YTEAAQLIKD
321 QEIPSVLAAV DATKHRKLAE TYNIKGFPTL KYFKEGEFAF DVNERDKDKI IDFVKDPKEP PPPPAPEPSW EEVDSDVIHL
401 NEENFKPTLK KRKHALIMFY APWCGHCKKA KPEFMAAAAK FRDDGKVMFG AVDCTTQQGV CSAHDVTGYP TFKYFNYNKN
481 SQKYMGGREE NDFVNFMRDP LSPSPSPPTA STKPEEDWAD IPGKDLIGFL TKDTFDSFVL SQGSVLVMFY APWCGHCKRM
561 KPAFGQAASV IVEEKLGALA AVDATIEQQL ADRFEIRGYP TLKYFRDGKY AFEYSGGR**TA DDFIAFLR**NP DAPPTTPSPE
641 PQWSDIVSDV HHLTDTSFDE FIAANKKTLV MFYSPGCGHC KKMKPDYMTA ATRLKEKVPD ARLAAVDATR FKDLARKYHI
721 KGYPTLKYFE DGAFKADYKS GRSAEDLVEF FSMASDKKDE L

Supporting Peptides:

Peptide	Uniq	-10lgP	Mass	Length	ppm	m/z	z	RT	Fraction	Scan	Source File	Area ebraeus	Area judaeus	#Feature	#Feature ebraeus	#Feature judaeus	Start	End	PTM	AScore	Found By
R.TADDFIAFLR.N	Y	74.95	1167.5924	10	-3.5	584.7990	2	52.65	2	F2:123233	20210611-09_2021021-OK160A_816_5.3.236.mgf		0	0	0	0	619	628			PEAKS DB
**total 1 peptides**																					

OK212_J_327_PDI

back to list

| Protein Coverage | Supporting Peptides |

Protein Coverage:

1 MALPWKALLF ASFTLLYVIP FRFGAKQNKK DLIIRVDDFK DFKKLLRTRT NLLVIFTQSE KAASKSLALF EEVAEEMRGK
81 ATMAYINCGD DKKLCKKLKV YTTTFELKHY KDGEFNKNYD RKLTVKSMVN FLMDPTGDIP WDEDLTATDV VHIDTEDGLN
161 KLLKKDKSAA LVMFYAPWCG YCKRLKPDFA AAATEVKGKA NLVGIDVDKP HMMGLRTLYN ITGFPTLYYF EKGKVKYRYG
241 GENSKEGIVS WLSNPKPPEE PKKEAEWAEE PSEVVHLEDD NFDDYIKEHP SVLVMFYAPW CGHCKKMKPG YTEAAQLIKD
321 QEIPSVLAAV DATKHRKLAE TYNIKGFPTL KYFKEGEFAF DVNERDKDKI IDFVKDPKEP PPPPAPEPSW EEVDSDVIHL
401 NEENFKPTLK KRKHALIMFY APWCGHCKKA KPEFMAAAAK FRDDGKVMFG AVDCTTQQGV CSAHDVTGYP TFKYFNYNKN
481 SQKYMGGREE NDFVNFMRDP LSPSPSPPTA STKPEEDWAD IPGKDLIGFL TKDTFDSFVL SQGSVLVMFY APWCGHCKRM
561 KPAFGQAASV IVEEKLGALA AVDATIEQQL ADRFEIRGYP TLKYFRDGKY AFEYSGGR**TA DDFIAFLR**NP DAPPTTPSPE
641 PQWSDIVSDV HHLTDTSFDE FIAANKKTLV MFYSPGCGHC KKMKPDYMTA ATRLKEKVPD ARLAAVDATR FKDLARKYHI
721 KGYPTLKYFE DGAFKADYKS GRSAEDLVEF FSMASDKKDE L

Supporting Peptides:

Peptide	Uniq	-10lgP	Mass	Length	ppm	m/z	z	RT	Fraction	Scan	Source File	Area ebraeus	Area judaeus	#Feature	#Feature ebraeus	#Feature judaeus	Start	End	PTM	AScore	Found By
R.TADDFIAFLR.N	Y	74.95	1167.5924	10	-3.5	584.7990	2	52.65	2	F2:123233	20210611-09_2021021-OK160A_816_5.3.236.mgf		0	0	0	0	619	628			PEAKS DB
**total 1 peptides**																					

OK193_E_274_PDI

back to list

| Protein Coverage | Supporting Peptides |

Protein Coverage:

1 MALPWKALLF ASFTLLYVIP FRFGAKQNKK DLIIRVDDFK DFKKLLRTRT NLLVIFTQSE KAASKSLALF EEVAEEMRGK
81 ATMAYINCGD DKKLCKKLKV YTTTFELKHY KDGEFNKNYD RKLTVKSMVN FLMDPTGDIP WDEDLTATDV VHIDTEDGLN
161 KLLKKDKSAA LVMFYAPWCG YCKRLKPDFA AAATEVKGKA NLVGIDVDKP HMMGLRTLYN ITGFPTLYYF EKGKVKYRYG
241 GENSKEGIVS WLSNPKPPEE PKKEAEWAEE PSEVVHLEDD NFDDYIKEHP SVLVMFYAPW CGHCKKMKPG YTEAAQLIKD
321 QEIPSVLAAV DATKHRKLAE TYNIKGFPTL KYFKEGEFAF DVNERDKDKI IDFVKDPKEP PPPPAPEPSW EEVDSDVIHL
401 NEENFKPTLK KRKHALIMFY APWCGHCKKA KPEFMAAAAK FRDDGKVMFG AVDCTTQQGV CSAHDVTGYP TFKYFNYNKN
481 SQKYMGGREE NDFVNFMRDP LSPSPSPPTA STKPEEDWAD IPGKDLIGFL TKDTFDSFVL SQGSVLVMFY APWCGHCKRM
561 KPAFGQAASV IVEEKLGALA AVDATIEQQL ADRFEIRGYP TLKYFRDGKY AFEYSGGR**TA DDFIAFLR**NP DAPPTIPSPE
641 PQWSDIVSDV HHLTDTSFDE FIAANKKTLV MFYSPGCGHC KKMKPDYMTA ATRLKEKVPD ARLAAVDATK FKDLAHKYHI
721 KGYPTLKYFE DGAFKADYKS GRSAEDLVEF FSMASDKKDE L

Supporting Peptides:

Peptide	Uniq	-10lgP	Mass	Length	ppm	m/z	z	RT	Fraction	Scan	Source File	Area ebraeus	Area judaeus	#Feature	#Feature ebraeus	#Feature judaeus	Start	End	PTM	AScore	Found By
R.TADDFIAFLR.N	Y	74.95	1167.5924	10	-3.5	584.7990	2	52.65	2	F2:123233	20210611-09_2021021-OK160A_816_5.3.236.mgf		0	0	0	0	619	628			PEAKS DB
**total 1 peptides**																					

OK005_J_321_PDI

back to list

| Protein Coverage | Supporting Peptides |

Protein Coverage:

1 MALPWKALLF ASFTLLYVIP FRFGAKQNKK DLIIRVDDFK DFKKLLRTRT NLLVIFTQSE KAASKSLALF EEVAEEMRGK
81 ATMAYINCGD DKKLCKKLKV YTTTFELKHY KDGEFNKNYD RKLTVKSMVN FLMDPTGDIP WDEDLTATDV VHIDTEDGLN
161 KLLKKDKSAA LVMFYAPWCG YCKRLKPDFA AAATEVKGKA NLVGIDVDKP HMMGLRTLYN ITGFPTLYYF EKGKVKYRYG
241 GENSKEGIVS WLSNPKPPEE PKKEAEWAEE PSEVVHLEDD NFDDYIKEHP SVLVMFYAPW CGHCKKMKPG YTEAAQLIKD
321 QEIPSVLAAV DATKHRKLAE TYNIKGFPTL KYFKEGEFAF DVNERDKDKI IDFVKDPKEP PPPPAPEPSW EEVDSDVIHL
401 NEENFKPTLK KRKHALIMFY APWCGHCKKA KPEFMAAAAK FRDDGKVMFG AVDCTTQQGV CSAHDVTGYP TFKYFNYNKN
481 SQKYMGGREE NDFVNFMRDP LSPSPSPPTA STKPEEDWAD IPGKDLIGFL TKDTFDSFVL SQGSVLVMFY APWCGHCKRM
561 KPAFGQAASV IVEEKLGALA AVDATIEQQL ADRFEIRGYP TLKYFRDGKY AFEYSGGR**TA DDFIAFLR**NP DAPPTTPSPE
641 PQWSDIVSDV HHLTDTSFDE FIAANKKTLV MFYSPGCGHC KKMKPDYMTA ATRLKEKVPD ARLAAVDATR FKDLARKYHI
721 KGYPTLKYFE DGAFKADYKS GRSAEDLVEF FSMASDKKDE L

Supporting Peptides:

Peptide	Uniq	-10lgP	Mass	Length	ppm	m/z	z	RT	Fraction	Scan	Source File	Area ebraeus	Area judaeus	#Feature	#Feature ebraeus	#Feature judaeus	Start	End	PTM	AScore	Found By
R.TADDFIAFLR.N	Y	74.95	1167.5924	10	-3.5	584.7990	2	52.65	2	F2:123233	20210611-09_2021021-OK160A_816_5.3.236.mgf		0	0	0	0	619	628			PEAKS DB
**total 1 peptides**																					

A_0855_170_Protein

back to list

| Protein Coverage | Supporting Peptides |

Protein Coverage:

1 SPKPPEEPKK EAEWAEEPSE VVHLEDDNFD DYIKEHPSVL VMFYAPWCGH CKKMKPGYTE AAQLIKDQEI PSVLAAVDAT
81 KHRKLADTYN IKGFPTLKYF KDGEFAFDVN ERDKDKIIDF VKDPKEPPPP PAPEPSWEEV DSDVIHLNEE NFKPTLKKRK
161 HALIMFYAPW CGHCKKAKPE FMAAAAKFRD DGKVMFGAVD CTTQQGICSA HDVTGYPTFK YFNYNKNSQK YMGGREENDF
241 VNFMRDPLSP SPSPPTASTK PEEDWADIPG KDFSFVLSQG SVLVMFYAPW CGHCKRMKPA FGQAASVIVE
321 EKLGALAAVD ATIEQQLADR FEIRGYPTLK YFKGGKYAFE YSGGR**TADDF IAFLR**NPDAP PTTPSPEPQW SDIVSDVHHL
401 TDTSFDEFIA ANKKTLVMFY SPGCGHCKKM KPDYMTAATR LKEKVPDARL AAVDATRFKD LARKYHIKGY PNLKYFEDGA
481 LKADYKSGRS AEDLVEFFST ASDKKDEL

Supporting Peptides:

Peptide	Uniq	-10lgP	Mass	Length	ppm	m/z	z	RT	Fraction	Scan	Source File	Area ebraeus	Area judaeus	#Feature	#Feature ebraeus	#Feature judaeus	Start	End	PTM	AScore	Found By
R.TADDFIAFLR.N	Y	74.95	1167.5924	10	-3.5	584.7990	2	52.65	2	F2:123233	20210611-09_2021021-OK160A_816_5.3.236.mgf		0	0	0	0	366	375			PEAKS DB
total 1 peptides																					

A_0039_255_Protein

back to list

| Protein Coverage | Supporting Peptides |

Protein Coverage:

1 MHCDSGCLVH SPKPPEEPKK EAEWAEEPSE VVHLEDDNFD DYIKEHPSVL VMFYAPWCGH CKKMKPGYTE AAQLIKDQEI
81 PSVLAAVDAT KHRKLADTYN IKGFPTLKYF KDGEFAFDVN ERDKDKIIDF VKDPKEPPPP PAPEPSWEEV DSDVIHLNEE
161 NFKPTLKKRK HALIMFYAPW CGHCKKAKPE FMAAAAKFRD DGKVMFGAVD CTTQQGICSA HDVTGYPTFK YFNYNKNSQK
241 YMGGREENDF VNFMRDPLSP SPSPPTASTK PEEDWADIPG KDFIGFLTKD TFDSFVLSQG SVLVMFYAPW CGHCKKMKPA
321 FGQAASVIVE EKLGALAAVD ATIEQQLADR FEIRGYPTLK YFKRGKYAFE YSGGR**TADDF IAFLR**NPDAP PTTPSPEPQW
401 SDIVSDVHHL TDTSFDEFIA ANKKTLVMFY SPGCGHCKKM KPDYMTAATR LKEKVPDARL AAVDATRFKD LARKYHIKGY
481 PTLKYFEDGA LKADYKSGRS AEDLVEFFST ASDKKDEL

Supporting Peptides:

Peptide	Uniq	-10lgP	Mass	Length	ppm	m/z	z	RT	Fraction	Scan	Source File	Area ebraeus	Area judaeus	#Feature	#Feature ebraeus	#Feature judaeus	Start	End	PTM	AScore	Found By
R.TADDFIAFLR.N	Y	74.95	1167.5924	10	-3.5	584.7990	2	52.65	2	F2:123233	20210611-09_2021021-OK160A_816_5.3.236.mgf		0	0	0	0	376	385			PEAKS DB
total 1 peptides																					

A_0239_037_Protein

back to list

| Protein Coverage | Supporting Peptides |

Protein Coverage:

1 MHCDSGCLVH SPKPPEEPKK EAEWAEEPSE VVHLEDDNFD DYIKEHPSVL VMFYAPWCGH CKKMKPGYTE AAQLIKDQEI
81 PSVLAAVDAT KHRKLADTYN IKGFPTLKYF KDGEFAFDVN ERDKDKIIDF VKDPKEPPPP PAPEPSWEEV DSDVIHLNEE
161 NFKPTLKKRK HALIMFYAPW CGHCKKAKPE FMAAAAKFRD DGKVMFGAVD CTTQQGICSA HDVTGYPTFK YFNYNKNSQK
241 YMGGREENDF VNFMRDPLSP SPSPPTASTK PEEDWADIPG KDFIGFLTKD TFDSFVLSQG SVLVMFYAPW CGHCKRMKPA
321 FGQAASVIVE EKLGALAAVD ATIEQQLADR FEIRGYPTLK YFKGGKYAFE YSGGR**TADDF IAFLR**NPDAP PTTPSPEPQW
401 SDIVSDVHHL TDTSFDEFIA ANKKTLVMFY SPGCGHCKKM KPDYMTAATR LKEKVPDARL AAVDATRFKD LARKYHIKGY
481 PTLKYFEDGA LKADYKSGRS AEDLVEFFST ASDKKDEL

Supporting Peptides:

Peptide	Uniq	-10lgP	Mass	Length	ppm	m/z	z	RT	Fraction	Scan	Source File	Area ebraeus	Area judaeus	#Feature	#Feature ebraeus	#Feature judaeus	Start	End	PTM	AScore	Found By
R.TADDFIAFLR.N	Y	74.95	1167.5924	10	-3.5	584.7990	2	52.65	2	F2:123233	20210611-09_2021021-OK160A_816_5.3.236.mgf		0	0	0	0	376	385			PEAKS DB
total 1 peptides																					

V_1278_149_Protein

back to list

| Protein Coverage | Supporting Peptides |

Protein Coverage:

1 MVNFLMDPTG DIPWDEDLTA TDVVHIDTED GLNKLLKKDK SAALVMFYAP WCGYCKRLKP DFAAAATEVK GKANLVGIDV
81 DKPHMMGLRT LYNITGFPTL YYFEKGKVKY RYGGENSKDG IVSWLSNPKP PEEPKKEAEW AEEPSEVVHL EDDNFDDYIK
161 EHPSVLVMFY APWCGHCKKM KPGYTEAAQL IKDQEIPSVL AAVDATKHRK LADTYNIKGF PTLKYFKDGE FAFDVNERDK
241 DKIIDFVKDP KEPPPPPAPE PSWEEVDSDV IHLNEENFKP TLKKRKHALI MFYAPWCGHC KKAKPEFMAA AAKFRDDGKV
321 MFGAVDCTTQ QGICSAHDVT GYPTFKYFNY NKNSQKYMGG REENDFVNFM RDPLSPSPSP PTASTKPEED WADIPGKDFI
401 GFLTKDTFDS FVLSQGSVLV MFYAPWCGHC KRMKPAFGQA ASVIVEEKLG ALAAVDATIE QQLADRFEIR GYPTLKYFKG
481 GKYAFEYSGG R**TADDFIAFL R**NPDAPPTTP SPEPQWSDIV SDVHHLTDTS FDEFIAANKK TLVMFYSPGC GHCKKMKPDY
561 MTAATRLKEK VPDARLAAVD ATRFKDLARK YHIKGYPTLK YFEDGALKAD YKSGRSAEDL VEFFSTASDK KDEL

Supporting Peptides:

Peptide	Uniq	-10lgP	Mass	Length	ppm	m/z	z	RT	Fraction	Scan	Source File	Area ebraeus	Area judaeus	#Feature	#Feature ebraeus	#Feature judaeus	Start	End	PTM	AScore	Found By
R.TADDFIAFLR.N	Y	74.95	1167.5924	10	-3.5	584.7990	2	52.65	2	F2:123233	20210611-09_2021021-OK160A_816_5.3.236.mgf		0	0	0	0	492	501			PEAKS DB
total 1 peptides																					

A_0039_254_Protein

back to list

| Protein Coverage | Supporting Peptides |

Protein Coverage:

1 MVNFLMDPTG DIPWDEDLTA TDVVHIYTED GLNKLLKKDK SAALVMFYAP WCGYCKRLKP DFAAAATEVK GKANLVGIDV
81 DKPHMMGLRT LYNITGFPTL YYFEKGKVKY RYGGENSKDG IVSWLSNPKP PEEPKKEAEW AEEPSEVVHL EDDNFDDYIK
161 EHPSVLVMFY APWCGHCKKM KPGYTEAAQL IKDQEIPSVL AAVDATKHRK LADTYNIKGF PTLKYFKDGE FAFDVNERDK
241 DKIIDFVKDP KEPPPPPAPE PSWEEVDSDV IHLNEENFKP TLKKRKHALI MFYAPWCGHC KKAKPEFMAA AAKFRDDGKV
321 MFGAVDCTTQ QGICSAHDVT GYPTFKYFNY NKNSQKYMGG REENDFVNFM RDPLSPSPSP PTASTKPEED WADIPGKDFI
401 GFLTKDTFDS FVLSQGSVLV MFYAPWCGHC KKMKPAFGQA ASVIVEEKLG ALAAVDATIE QQLADRFEIR GYPTLKYFKR
481 GKYAFEYSGG R**TADDFIAFL R**NPDAPPTTP SPEPQWSDIV SDVHHLTDTS FDEFIAANKK TLVMFYSPGC GHCKKMKPDY
561 MTAATRLKEK VPDARLAAVD ATRFKDLARK YHIKGYPTLK YFEDGALKAD YKSGRSAEDL VEFFSTASDK KDEL

Supporting Peptides:

Peptide	Uniq	-10lgP	Mass	Length	ppm	m/z	z	RT	Fraction	Scan	Source File	Area ebraeus	Area judaeus	#Feature	#Feature ebraeus	#Feature judaeus	Start	End	PTM	AScore	Found By
R.TADDFIAFLR.N	Y	74.95	1167.5924	10	-3.5	584.7990	2	52.65	2	F2:123233	20210611-09_2021021-OK160A_816_5.3.236.mgf		0	0	0	0	492	501			PEAKS DB
total 1 peptides																					

V_CG13_297_Protein

back to list

| Protein Coverage | Supporting Peptides |

Protein Coverage:

```
  1 MVNFLMDPTG DIPWDEDLTA TDVVHIDTED GLNKLLKKDK SAALVMFYAP WCGYCKRLKP DFAAAATEVK GKANLVGIDV
 81 DKPHMMGLRT LYNITGFPTL YYLEKGKVKY RYGGENSKDG IVSWLSNPKP PEEPKKEAEW AEEPSEVVHL EDDNFDDYIK
161 EHPSVLVMFY APWCGHCKKM KPGYTEAAQL IKDQEIPSVL AAVDATKHRK LADTYNIKGF PTLKYFKDGE FAFDVNERDK
241 DKIIDFVKDP KEPPPPPAPE PSWEEVDSDV IHLNEENFKP TLKKRKHALI MFYAPWCGHC KKAKPEFMAA AAKFRDDGKV
321 MFGAVDCTTQ QGICSAHDVT GYPTFKYFNY NKNSQKYMGG REENDFVNFM RDPLSPSPSP PTASTKPEED WADIPGKDFI
401 GFLTKDTFDS FVLSQGSVLV MFYAPWCGHC KRMKPAFGQA ASVIVEEKLG ALAAVDATIE QQLADRFEIR GYPTLKYFKG
481 GKYAFEYSGG RTADDFIAFL RNPDAPPTTP SPEPQWSDIV SDVHHLTDTS FDEFIAANKK TLVMFYSPGC GHCKKMKPDY

561 MTAATRLKEK VPDARLAAVD ATRFKDLARK YHIKGYPTLK YFEDGALKAD YKSGRSAEDL VEFFSTASDK KDEL
```

Supporting Peptides:

Peptide	Uniq	-10lgP	Mass	Length	ppm	m/z	z	RT	Fraction	Scan	Source File	Area ebraeus	Area judaeus	#Feature	#Feature ebraeus	#Feature judaeus	Start	End	PTM	AScore	Found By
R.TADDFIAFLR.N	Y	74.95	1167.5924	10	-3.5	584.7990	2	52.65	2	F2:123233	20210611-09_2021021-OK160A_816_5.3.236.mgf		0	0	0	0	492	501			PEAKS DB
**total 1 peptides**																					

A_0855_171_Protein

back to list

| Protein Coverage | Supporting Peptides |

Protein Coverage:

```
  1 MVNFLMDPTG DIPWDEDLTA TDVVHIYTED GLNKLLKKDK SAALVMFYAP WCGYCKRLKP DFAAAATEVK GKANLVGIDV
 81 DKPHMMGLRT LYNITGFPTL YYFEKGKVKY RYGGENSKDG IVSWLSNPKP PEEPKKEAEW AEEPSEVVHL EDDNFDDYIK
161 EHPSVLVMFY APWCGHCKKM KPGYTEAAQL IKDQEIPSVL AAVDATKHRK LADTYNIKGF PTLKYFKDGE FAFDVNERDK
241 DKIIDFVKDP KEPPPPPAPE PSWEEVDSDV IHLNEENFKP TLKKRKHALI MFYAPWCGHC KKAKPEFMAA AAKFRDDGKV
321 MFGAVDCTTQ QGICSAHDVT GYPTFKYFNY NKNSQKYMGG REENDFVNFM RDPLSPSPSP PTASTKPEED WADIPGKDFI
401 GFLTKDTFDS FVLSQGSVLV MFYAPWCGHC KRMKPAFGQA ASVIVEEKLG ALAAVDATIE QQLADRFEIR GYPTLKYFKG
481 GKYAFEYSGG RTADDFIAFL RNPDAPPTTP SPEPQWSDIV SDVHHLTDTS FDEFIAANKK TLVMFYSPGC GHCKKMKPDY

561 MTAATRLKEK VPDARLAAVD ATRFKDLARK YHIKGYPNLK YFEDGALKAD YKSGRSAEDL VEFFSTASDK KDEL
```

Supporting Peptides:

Peptide	Uniq	-10lgP	Mass	Length	ppm	m/z	z	RT	Fraction	Scan	Source File	Area ebraeus	Area judaeus	#Feature	#Feature ebraeus	#Feature judaeus	Start	End	PTM	AScore	Found By
R.TADDFIAFLR.N	Y	74.95	1167.5924	10	-3.5	584.7990	2	52.65	2	F2:123233	20210611-09_2021021-OK160A_816_5.3.236.mgf		0	0	0	0	492	501			PEAKS DB
**total 1 peptides**																					

A_0055_279_Protein

back to list

| Protein Coverage | Supporting Peptides |

Protein Coverage:

```
  1 MVNFLMDPTG DIPWDEDLTA TDVVHIYTED GLNKLLKKDK SAALVMFYAP WCGYCKRLKP DFAAAATEVK GKANLVGIDV
 81 DKPHMMGLRT LYNITGFPTL YYFEKGKVKY RYGGENSKDG IVSWLSNPKP PEEPKKEAEW AEEPSEVVHL EDDNFDDYIK
161 EHPSVLVMFY APWCGHCKKM KPGYTEAAQL IKDQEIPSVL AAVDATKHRK LADTYNIKGF PTLKYFKDGE FAFDVNERDK
241 DKIIDFVKDP KEPPPPPAPE PSWEEVDSDV IHLNEENFKP MLKKRKHALI MFYAPWCGHC KKAKPEFMAA AAKFRDDGKV
321 MFGAVDCTTQ QGICSAHDVT GYPTFKYFNY NKNSQKYMGG REENDFVNFM RDPLSPSPSP PTASTKPEED WADIPGKDFI
401 GFLTKDTFDS FVLSQGSVLV MFYAPWCGHC KRMKPAFGQA ASVIVEEKLG ALAAVDATIE QQLADRFEIR GYPTLKYFKR
481 GKYAFEYSGG RTADDFIAFL RNPDAPPTTP SPEPQWSDIV SDVHHLTDTS FDEFIAANKK TLVMFYSPGC GHCKKMKPDY

561 MTAATRLKEK VPDARLAAVD ATRFKDLARK YHIKGYPNLK YFEDGALKAD YKSGRSAEDL VEFFSTASDK KDEL
```

Supporting Peptides:

Peptide	Uniq	-10lgP	Mass	Length	ppm	m/z	z	RT	Fraction	Scan	Source File	Area ebraeus	Area judaeus	#Feature	#Feature ebraeus	#Feature judaeus	Start	End	PTM	AScore	Found By
R.TADDFIAFLR.N	Y	74.95	1167.5924	10	-3.5	584.7990	2	52.65	2	F2:123233	20210611-09_2021021-OK160A_816_5.3.236.mgf		0	0	0	0	492	501			PEAKS DB
**total 1 peptides**																					

A_0875_155_Protein

back to list

| Protein Coverage | Supporting Peptides |

Protein Coverage:

```
  1 MFYAPWCGHC KKAKPEFMAA AAKFRDDGKV MFGAVDCTTQ QGICSAHDVT GYPTFKYFNY NKNSQKYMGG REENDFVNFM
 81 RDPLSPSPSP PTASTKPEED WADIPGKDFI GFLTKDTFDS FVLSQGSVLV MFYAPWCGHC KRMKPAFGQA ASVIVEEKLG
161 ALAAVDATIE QQLADRFEIR GYPTLKYFKG GKYAFEYSGG RTADDFIAFL RSPDAPPTTP SPEPQWSDIV SDVHHLTDTS

241 FDEFIAANKK TLVMFYSPGC GHCKKMKPDY MTAATRLKEK VPDARLAAVD ATRFKDLARK YHIKGYPNLK YFEDGALKAD
321 YKSGRSAEDL VEFFSTASDK KDEL
```

Supporting Peptides:

Peptide	Uniq	-10lgP	Mass	Length	ppm	m/z	z	RT	Fraction	Scan	Source File	Area ebraeus	Area judaeus	#Feature	#Feature ebraeus	#Feature judaeus	Start	End	PTM	AScore	Found By
R.TADDFIAFLR.S	Y	74.95	1167.5924	10	-3.5	584.7990	2	52.65	2	F2:123233	20210611-09_2021021-OK160A_816_5.3.236.mgf		0	0	0	0	202	211			PEAKS DB
**total 1 peptides**																					

A_0885_186_Protein

back to list

| Protein Coverage | Supporting Peptides |

Protein Coverage:

```
  1 MVNFLMDPTG DIPWDEDLTA TDVVHIYTED GLNKLLKKDK SAALVMFYAP WCGYCKRLKP DFAAAATEVK GKANLVGIDV
 81 DKPHMMGLRT LYNITGFPTL YYFEKGKVKY RYGGENSKDG IVSWLSNPKP PEEPKKEAEW AEEPSEVVHL EDDNFDDYIK
161 EHPSVLVMFY APWCGHCKKM KPGYTEAAQL IKDQEIPSVL AAVDATKHRK LADTYNIKGF PTLKYFKDGE FAFDVNERDK
241 DKIIDFVKDP KEPPPPPAPE PSWEEVDSDV IHLNEENFKP MLKKRKHALI MFYAPWCGHC KKAKPEFMAA AVKFRDDGKV
321 MFGAVDCTTQ QGICSAHDVT GYPTFKYFNY NKNSQKYMGG REENDFVNFM RDPLSPSPSP PTASTKPEED WADIPGKDFI
401 GFLTKDTFDS FVLSQGSVLV MFYAPWCGHC KRMKPAFGQA ASVIVEEKLG ALAAVDATIE QQLADRFEIR GYPTLKYFKG
481 GKYAFEYSGG RTADDFIAFL RNPDAPPTTP SPEPQWSDIV SDVHHLTDTS FDEFIAANKK TLVMFYSPGC GHCKKMKPDY

561 MTAATRLKEK VPDARLAAVD ATRFKDLARK YHIKGYPTLK YFEDGALKAD YKSGRSAEDL VEFFSTASDK KDEL
```

Supporting Peptides:

Peptide	Uniq	-10lgP	Mass	Length	ppm	m/z	z	RT	Fraction	Scan	Source File	Area ebraeus	Area judaeus	#Feature	#Feature ebraeus	#Feature judaeus	Start	End	PTM	AScore	Found By

total 1 peptides

Peptide	Uniq	-10lgP	Mass	Length	ppm	m/z	z	RT	Fraction	Scan	Source File	Area ebraeus	Area judaeus	#Feature	#Feature ebraeus	#Feature judaeus	Start	End	PTM	AScore	Found By
R.TADDFIAFLR.N	Y	74.95	1167.5924	10	-3.5	584.7990	2	52.65	2	F2:123233	20210611-09_2021021-OK160A_816_5.3.236.mgf		0	0	0	0	492	501			PEAKS DB
total 1 peptides																					

OK193_E_118_N

back to list

| Protein Coverage | Supporting Peptides |

Protein Coverage:

1 CPGPKSCGSG EAEVCCESSD CYCK**LLHSQP FSR**LVCVCPD

Supporting Peptides:

Peptide	Uniq	-10lgP	Mass	Length	ppm	m/z	z	RT	Fraction	Scan	Source File	Area ebraeus	Area judaeus	#Feature	#Feature ebraeus	#Feature judaeus	Start	End	PTM	AScore	Found By
K.LLHSQPFSR.L	Y	73.45	1083.5825	9	1.9	362.2008	3	21.01	1	F1:12663	20210611-08_2021021-OK192A_815_5.3.236.mgf	0		0	0	0	25	33			PEAKS DB
total 1 peptides																					

SRX1323887_E_088_N

back to list

| Protein Coverage | Supporting Peptides |

Protein Coverage:

1 CPGPKSCGSG EAEVCCESSD CYCK**LLHSQP FSR**LVCVCPD

Supporting Peptides:

Peptide	Uniq	-10lgP	Mass	Length	ppm	m/z	z	RT	Fraction	Scan	Source File	Area ebraeus	Area judaeus	#Feature	#Feature ebraeus	#Feature judaeus	Start	End	PTM	AScore	Found By
K.LLHSQPFSR.L	Y	73.45	1083.5825	9	1.9	362.2008	3	21.01	1	F1:12663	20210611-08_2021021-OK192A_815_5.3.236.mgf	0		0	0	0	25	33			PEAKS DB
total 1 peptides																					

OK213_E_135_N

back to list

| Protein Coverage | Supporting Peptides |

Protein Coverage:

1 CPGPKSCGSG EAEVCCESSD CYCK**LLHSQP FSR**LVCVCPD

Supporting Peptides:

Peptide	Uniq	-10lgP	Mass	Length	ppm	m/z	z	RT	Fraction	Scan	Source File	Area ebraeus	Area judaeus	#Feature	#Feature ebraeus	#Feature judaeus	Start	End	PTM	AScore	Found By
K.LLHSQPFSR.L	Y	73.45	1083.5825	9	1.9	362.2008	3	21.01	1	F1:12663	20210611-08_2021021-OK192A_815_5.3.236.mgf	0		0	0	0	25	33			PEAKS DB
total 1 peptides																					

OK193_E_117_N

back to list

| Protein Coverage | Supporting Peptides |

Protein Coverage:

1 CPGPKSCGSG EAEVCCESSD CYCK**LLHSQP FSR**LVCVCPD

Supporting Peptides:

Peptide	Uniq	-10lgP	Mass	Length	ppm	m/z	z	RT	Fraction	Scan	Source File	Area ebraeus	Area judaeus	#Feature	#Feature ebraeus	#Feature judaeus	Start	End	PTM	AScore	Found By
K.LLHSQPFSR.L	Y	73.45	1083.5825	9	1.9	362.2008	3	21.01	1	F1:12663	20210611-08_2021021-OK192A_815_5.3.236.mgf	0		0	0	0	25	33			PEAKS DB
total 1 peptides																					

V_CG13_125_A-1

back to list

| Protein Coverage | Supporting Peptides |

Protein Coverage:

17 C

1 DGCCSFPPCK **GIHPHLCA**

C Carbamidomethylation (+57.02)

Supporting Peptides:

Peptide	Uniq	-10lgP	Mass	Length	ppm	m/z	z	RT	Fraction	Scan	Source File	Area ebraeus	Area judaeus	#Feature	#Feature ebraeus	#Feature judaeus	Start	End	PTM	AScore	Found By
K.GIHPHLC(+57.02)A	Y	72.97	903.4385	8	0.9	452.7252	2	18.46	1	F1:9724	20210611-08_2021021-OK192A_815_5.3.236.mgf	0		0	0	0	11	18	Carbamidomethylation	C7:Carbamidomethylation:1000.00	PEAKS DB
total 1 peptides																					

OK006_E_107_M

back to list

| Protein Coverage | Supporting Peptides |

Protein Coverage:

1 K**LDADQPVKR** YVENKENLNP D

Supporting Peptides:

Peptide	Uniq	-10lgP	Mass	Length	ppm	m/z	z	RT	Fraction	Scan	Source File	Area ebraeus	Area judaeus	#Feature	#Feature ebraeus	#Feature judaeus	Start	End	PTM	AScore	Found By
K.LDADQPVKR.Y	Y	72.81	1040.5614	9	1.8	521.2867	2	13.67	2	F2:56990	20210611-09_2021021-OK160A_816_5.3.236.mgf		0	0	0	0	2	10			PEAKS DB
total 1 peptides																					

OK213_E_203_Q

back to list

| Protein Coverage | Supporting Peptides |

Protein Coverage:

35 C

1 MDTNDCTPCA PDLCCRPGLR CATSTR**QGHY DEPACVSY**

C Carbamidomethylation (+57.02)

Supporting Peptides:

Peptide	Uniq	-10lgP	Mass	Length	ppm	m/z	z	RT	Fraction	Scan	Source File	Area ebraeus	Area judaeus	#Feature	#Feature ebraeus	#Feature judaeus	Start	End	PTM	AScore	Found By
R.QGHYDEPAC(+57.02)VSY	Y	72.77	1424.5667	12	3.9	713.2906	2	26.76	1	F1:20862	20210611-08_2021021-OK192A_815_5.3.236.mgf	0		0	0	0	27	38	Carbamidomethylation	C9:Carbamidomethylation:1000.00	PEAKS DB
total 1 peptides																					

OK213_E_192_O2

back to list

| Protein Coverage | Supporting Peptides |

Protein Coverage:

1 DGGSVGRSLK **GPIQLPWGK**S VVLRKDVQSG WDQPADRNAV PRDDNPGGAI GKS

Supporting Peptides:

Peptide	Uniq	-10lgP	Mass	Length	ppm	m/z	z	RT	Fraction	Scan	Source File	Area ebraeus	Area judaeus	#Feature	#Feature ebraeus	#Feature judaeus	Start	End	PTM	AScore	Found By
K.GPIQLPWGK.S	Y	71.13	994.5599	9	0.3	498.2855	2	42.60	1	F1:40496	20210611-08_2021021-OK192A_815_5.3.236.mgf	0		0	0	0	11	19			PEAKS DB
total 1 peptides																					

OK005_J_272_Ggeo01

back to list

| Protein Coverage | Supporting Peptides |

Protein Coverage:

1 SQADDGGTEK R**SWPMSDADL K**GTFDKIGHG SFGGGFGGGK

Oxidation (M) (+15.99)

Supporting Peptides:

Peptide	Uniq	-10lgP	Mass	Length	ppm	m/z	z	RT	Fraction	Scan	Source File	Area ebraeus	Area judaeus	#Feature	#Feature ebraeus	#Feature judaeus	Start	End	PTM	AScore	Found By
R.SWPMSDADLK.G	Y	70.99	1148.5172	10	-0.6	575.2631	2	35.64	2	F2:93830	20210611-09_2021021-OK160A_816_5.3.236.mgf		0	0	0	0	12	21			PEAKS DB
R.SWPM(+15.99)SDADLK.G	Y	53.35	1164.5121	10	2.5	583.2623	2	28.17	2	F2:80256	20210611-09_2021021-OK160A_816_5.3.236.mgf		0	0	0	0	12	21	Oxidation (M)	M4:Oxidation (M):1000.00	PEAKS DB
total 2 peptides																					

CebrOK035_J_299_Ggeo01

back to list

| Protein Coverage | Supporting Peptides |

Protein Coverage:

1 SQADDGGTEK R**SWPMSDADL K**GTFDKIGHG SFGGGFGGGK

Oxidation (M) (+15.99)

Supporting Peptides:

Peptide	Uniq	-10lgP	Mass	Length	ppm	m/z	z	RT	Fraction	Scan	Source File	Area ebraeus	Area judaeus	#Feature	#Feature ebraeus	#Feature judaeus	Start	End	PTM	AScore	Found By
R.SWPMSDADLK.G	Y	70.99	1148.5172	10	-0.6	575.2631	2	35.64	2	F2:93830	20210611-09_2021021-OK160A_816_5.3.236.mgf		0	0	0	0	12	21			PEAKS DB
R.SWPM(+15.99)SDADLK.G	Y	53.35	1164.5121	10	2.5	583.2623	2	28.17	2	F2:80256	20210611-09_2021021-OK160A_816_5.3.236.mgf		0	0	0	0	12	21	Oxidation (M)	M4:Oxidation (M):1000.00	PEAKS DB
total 2 peptides																					

OK214_J_263_Ggeo01

back to list

| Protein Coverage | Supporting Peptides |

Protein Coverage:

1 SQADDGGTEK R**SWPMSDADL K**GTFDKIGHG SFGGGFGGGK

Oxidation (M) (+15.99)

Supporting Peptides:

Peptide	Uniq	-10lgP	Mass	Length	ppm	m/z	z	RT	Fraction	Scan	Source File	Area ebraeus	Area judaeus	#Feature	#Feature ebraeus	#Feature judaeus	Start	End	PTM	AScore	Found By
R.SWPMSDADLK.G	Y	70.99	1148.5172	10	-0.6	575.2631	2	35.64	2	F2:93830	20210611-09_2021021-OK160A_816_5.3.236.mgf		0	0	0	0	12	21			PEAKS DB
R.SWPM(+15.99)SDADLK.G	Y	53.35	1164.5121	10	2.5	583.2623	2	28.17	2	F2:80256	20210611-09_2021021-OK160A_816_5.3.236.mgf		0	0	0	0	12	21	Oxidation (M)	M4:Oxidation (M):1000.00	PEAKS DB
total 2 peptides																					

gene:107285462

back to list

| Protein Coverage | Supporting Peptides |

Protein Coverage:

1 MSPVRAVLAL LAALWAPAAG YFVSIDAHAE ECFLERVPSG TKMGLIFEVA EGGFLDIDVE ITGPDNKGIH KGDRESSGKY
81 TFAAHMDGTY KFCFSNKMST MTPKIVMFTI DIGEAPKGQD METEAHQNKL EEMINELAVA MTAVK**HEQEY MEVR**ERVHRA
161 INDNTNSRVV LWSFFEALVL VAMTLGQIYY LKRFFEVRRV V

Supporting Peptides:

Peptide	Uniq	-10lgP	Mass	Length	ppm	m/z	z	RT	Fraction	Scan	Source File	Area ebraeus	Area judaeus	#Feature	#Feature ebraeus	#Feature judaeus	Start	End	PTM	AScore	Found By
K.HEQEYMEVR.E	Y	70.43	1219.5292	9	-0.1	610.7692	2	18.53	2	F2:63972	20210611-09_2021021-OK160A_816_5.3.236.mgf		0	0	0	0	146	154			PEAKS DB
total 1 peptides																					

K_0010_081_Rimp

back to list

| Protein Coverage | Supporting Peptides |

Protein Coverage:

1 IYYPNGRPIG PCSTNDISKT EVFSIRNARW ELHYSYCTCA SGEDYIEAED RTTSSTTVSY KIYECGMPTY SCSGGTFPVT
81 DADDGPRRMQ CTCGQYK**YFV SPTLGWHVR**C

Supporting Peptides:

Peptide	Uniq	-10lgP	Mass	Length	ppm	m/z	z	RT	Fraction	Scan	Source File	Area ebraeus	Area judaeus	#Feature	#Feature ebraeus	#Feature judaeus	Start	End	PTM	AScore	Found By
K.YFVSPTLGWHVR.C	Y	69.05	1460.7565	12	0.8	487.9246	3	42.46	1	F1:40238	20210611-08_2021021-OK192A_815_5.3.236.mgf	0		0	0	0	98	109			PEAKS DB
total 1 peptides																					

OK006_E_029_Conkunitzin

back to list

| Protein Coverage | Supporting Peptides |

Protein Coverage:

1 GACEKPKVEG TGSQSISSYF FNSASGTCDT FVYK**GGNDDP FPNR**FNSHPV CENVCKCKLR MDPGTGERLR TRYYHNTDGS
81 RCLPFQYRGT GGNANNFLSE SACEMECEL

Supporting Peptides:

Peptide	Uniq	-10lgP	Mass	Length	ppm	m/z	z	RT	Fraction	Scan	Source File	Area ebraeus	Area judaeus	#Feature	#Feature ebraeus	#Feature judaeus	Start	End	PTM	AScore	Found By
K.GGNDDPFPNR.F	Y	69.01	1087.4683	10	1.1	544.7399	2	22.11	1	F1:14025	20210611-08_2021021-OK192A_815_5.3.236.mgf	0		0	0	0	35	44			PEAKS DB
total 1 peptides																					

OK193_E_027_Conkunitzin

back to list

| Protein Coverage | Supporting Peptides |

Protein Coverage:

1 GACEKPKVEG TGSQSISSYF FNSASGTCDT FVYKGGNDDP FPNRFNSHPV CENVCKCKLR MDPGTGERLR TRYYHNTDGS

81 RCLPFQYRGT GGNANNFLSE SACEMECEL

Supporting Peptides:

Peptide	Uniq	-10lgP	Mass	Length	ppm	m/z	z	RT	Fraction	Scan	Source File	Area ebraeus	Area judaeus	#Feature	#Feature ebraeus	#Feature judaeus	Start	End	PTM	AScore	Found By
K.GGNDDPFPNR.F	Y	69.01	1087.4683	10	1.1	544.7399	2	22.11	1	F1:14025	20210611-08_2021021-OK192A_815_5.3.236.mgf	0		0	0	0	35	44			PEAKS DB
total 1 peptides																					

CebrOK016_E_012_Conkunitzin

back to list

| Protein Coverage | Supporting Peptides |

Protein Coverage:

1 GACEKPKVEG TGSQSISSYF FNSASGTCDT FVYKGGNDDP FPNRFNSHPV CENVCKCKLR MDPGTGERLR TRYYHNTDGS

81 RCLPFQYRGT GGNANNFLSE SACEMECEL

Supporting Peptides:

Peptide	Uniq	-10lgP	Mass	Length	ppm	m/z	z	RT	Fraction	Scan	Source File	Area ebraeus	Area judaeus	#Feature	#Feature ebraeus	#Feature judaeus	Start	End	PTM	AScore	Found By
K.GGNDDPFPNR.F	Y	69.01	1087.4683	10	1.1	544.7399	2	22.11	1	F1:14025	20210611-08_2021021-OK192A_815_5.3.236.mgf	0		0	0	0	35	44			PEAKS DB
total 1 peptides																					

SRX1323887_E_028_Conkunitzin

back to list

| Protein Coverage | Supporting Peptides |

Protein Coverage:

1 GACEKPKVEG TGSQSISSYF FNSASGTCDT FVYKGGNDDP FPNRFNSHPV CENVCKCKLR MDPGTGERLR TRYYHNTDGS

81 RCLPFQYRGT GGNANNFLSE SACEMECEL

Supporting Peptides:

Peptide	Uniq	-10lgP	Mass	Length	ppm	m/z	z	RT	Fraction	Scan	Source File	Area ebraeus	Area judaeus	#Feature	#Feature ebraeus	#Feature judaeus	Start	End	PTM	AScore	Found By
K.GGNDDPFPNR.F	Y	69.01	1087.4683	10	1.1	544.7399	2	22.11	1	F1:14025	20210611-08_2021021-OK192A_815_5.3.236.mgf	0		0	0	0	35	44			PEAKS DB
total 1 peptides																					

OK213_E_033_Conkunitzin

back to list

| Protein Coverage | Supporting Peptides |

Protein Coverage:

1 MTPSTSVLHL SFITPYCCSL HPERNMDALR FSAVLLILAM ACFITVSAGA CEKPKVEGTG SQSISSYFFN SASGTCDTFV
81 YKGGNDDPFP NRFNSHPVCE NVCKCKLRMD PGTGERLRTR YYHNTDGSRC LPFQYRGTGG NANNFLSESA CEMECEL

Supporting Peptides:

Peptide	Uniq	-10lgP	Mass	Length	ppm	m/z	z	RT	Fraction	Scan	Source File	Area ebraeus	Area judaeus	#Feature	#Feature ebraeus	#Feature judaeus	Start	End	PTM	AScore	Found By
K.GGNDDPFPNR.F	Y	69.01	1087.4683	10	1.1	544.7399	2	22.11	1	F1:14025	20210611-08_2021021-OK192A_815_5.3.236.mgf	0		0	0	0	83	92			PEAKS DB
total 1 peptides																					

OK212_J_201_T

back to list

| Protein Coverage | Supporting Peptides |

Protein Coverage:

1 AALGDADLKG LTGVFYHRCC HFP

Supporting Peptides:

Peptide	Uniq	-10lgP	Mass	Length	ppm	m/z	z	RT	Fraction	Scan	Source File	Area ebraeus	Area judaeus	#Feature	#Feature ebraeus	#Feature judaeus	Start	End	PTM	AScore	Found By
AALGDADLK.G	Y	66.91	872.4603	9	2.0	437.2365	2	24.13	2	F2:73310	20210611-09_2021021-OK160A_816_5.3.236.mgf		0	0	0	0	1	9			PEAKS DB
total 1 peptides																					

D_092_FK506-binding

back to list

| Protein Coverage | Supporting Peptides |

Protein Coverage:

1 MVSRRNAVKT SVLLLLCSLG GVSRADETEE ELINEVIFKP ETCDRLSKAH DMLSMHYTGT LTATGVKFDS SHDRNQPFEF
81 QLGVGQVIQG WEKGLLDMCV GEKRKLIIPS HLGYGDQGAG EMIPPKSSLT FEVELLNIKD GTPPQNIFKE IDTNEDKLLS
161 KEEISGFLKE QAQKAGPGSG LTDEHHEKII TEIFEHEDKD KDGFISHKEF SGPKHDEL

Supporting Peptides:

Peptide	Uniq	-10lgP	Mass	Length	ppm	m/z	z	RT	Fraction	Scan	Source File	Area ebraeus	Area judaeus	#Feature	#Feature ebraeus	#Feature judaeus	Start	End	PTM	AScore	Found By
K.EEISGFLK.E	Y	66.80	921.4807	8	-0.4	461.7455	2	34.49	2	F2:91692	20210611-09_2021021-OK160A_816_5.3.236.mgf	0	0	0	0	0	162	169			PEAKS DB
total 1 peptides																					

SRX1323887_E_137_P

back to list

| Protein Coverage | Supporting Peptides |

Protein Coverage:

1 ESSSLVLCRS PCSAVRFCPP TCSYCEGTPQ IC

Supporting Peptides:

Peptide	Uniq	-10lgP	Mass	Length	ppm	m/z	z	RT	Fraction	Scan	Source File	Area ebraeus	Area judaeus	#Feature	#Feature ebraeus	#Feature judaeus	Start	End	PTM	AScore	Found By
ESSSLVLC(+57.02)R.S	Y	66.31	1049.5176	9	2.8	525.7656	2	25.17	1	F1:18696	20210611-08_2021021-OK192A_815_5.3.236.mgf	0		0	0	0	1	9		C8:Carbamidomethylation:1000.00	PEAKS DB
total 1 peptides																					

gene:107290974

back to list

| Protein Coverage | Supporting Peptides |

Protein Coverage:

1 MLGFAGRCCA SSASFLLRGA RGASSPLQDL LQLRGGVVAA AAEARRNYAA QTAPAAKAGT ATGHIVAVIG AVVDVQFDEA
81 LPPILNALEV QGRDTRLVLE VAQHLGENTV RTIAMDGTEG LVRGQKVLDS GAPIRIPVGP ETLGRIMNVI GEPIDERGPI
161 KTKQFAAIHA EAPEFVEMSV EQEILVTGIK VVDLLAPYAK GGK**IGLFGGA GVGK**TVLIME LINNVAKAHG GYSVFAGVGE
241 RTREGNDLYH EMIESGVINL KDTTSKVALV YGQMNEPPGA RARVALTGLT VAEYFRDQEG QDVLLFIDNI FRFTQAGSEV
321 SALLGRIPSA VGYQPTLATD MGTMQERITT TRKGSITSVQ AIYVPADDLT DPAPATTFAH LDATTVLSRA IAELGIYPAV
401 DPLDSTSRIM DPNIVGQEHY DVARGVQKIL QDYKSLQDII AILGMDELSE EDKLTVARAR KIQRFLSQPF QVAEVFTGHA
481 GKLVPLKETI KGFQQILIGE YDHLPEQAFY MVGPIEEAVA KAEKLAEEHS

Supporting Peptides:

Peptide	Uniq	-10lgP	Mass	Length	ppm	m/z	z	RT	Fraction	Scan	Source File	Area ebraeus	Area judaeus	#Feature	#Feature ebraeus	#Feature judaeus	Start	End	PTM	AScore	Found By
K.IGLFGGAGVGK.T	Y	65.83	974.5549	11	0.9	488.2831	2	36.87	2	F2:95816	20210611-09_2021021-OK160A_816_5.3.236.mgf		0	0	0	0	204	214			PEAKS DB
total 1 peptides																					

OK214_J_257_Cver07

back to list

| Protein Coverage | Supporting Peptides |

Protein Coverage:

1 SPLFCNTTKV EKLQARDLIG NVYMR**LANLP DEGTR**SQLGF KLSVTEINGK ENLNLQVFFW KHGTCSSWFS DCVVNDNYCE
81 YSSGRKIYFI FHNEDYDVTF GTPGNTVKIL TRYTGGSRID VEKLPQDMLS TIADKCDMEV ITNAFYTRRF DPWYTAPTCD
161 KPQLSFFSFI HLFKSFSKTF I

Supporting Peptides:

Peptide	Uniq	-10lgP	Mass	Length	ppm	m/z	z	RT	Fraction	Scan	Source File	Area ebraeus	Area judaeus	#Feature	#Feature ebraeus	#Feature judaeus	Start	End	PTM	AScore	Found By
R.LANLPDEGTR.S	Y	65.46	1084.5513	10	1.0	543.2812	2	23.80	2	F2:72699	20210611-09_2021021-OK160A_816_5.3.236.mgf		0	0	0	0	26	35			PEAKS DB
total 1 peptides																					

gene:107293638

back to list

| Protein Coverage | Supporting Peptides |

Protein Coverage:

1 MTAIGGQAGY IYPRSDSAAL FLFDLREAFI RRCHLVSCRC FKGKLTMSGG LDVLQMKEED VLKFLAAGTH LGGTNLDFQM
81 EQYIYKRKSD GIYIINLKRT WEKLLLAARA IVAIENPADV SVISSRNTGQ RAVLKFAAAT GATPIAGRFT PGTFTNQIQA
161 AFREPR**LLVV TDPR**ADHQPL TEASYVNIPT IALCNTDSPL RYVDIAIPCN NKGAHSVGLM WWMLAREVLR MRGTISREHP
241 WEVMPDLYFY RDPEEIEKEE QAAADKAVTK EEFQGEWTAP APEFTAPPQP EVSDWSEGVQ VPSVPIQPYP TEDWSSQTAT
321 EDWSAAPTAQ ATEWVGTATE WS

Supporting Peptides:

Peptide	Uniq	-10lgP	Mass	Length	ppm	m/z	z	RT	Fraction	Scan	Source File	Area ebraeus	Area judaeus	#Feature	#Feature ebraeus	#Feature judaeus	Start	End	PTM	AScore	Found By
R.LLVVTDPR.A	Y	65.35	911.5440	8	3.6	456.7790	2	29.59	2	F2:82621	20210611-09_2021021-OK160A_816_5.3.236.mgf		0	0	0	0	167	174			PEAKS DB
total 1 peptides																					

SRX1323887_E_222_Conorfamide

back to list

| Protein Coverage | Supporting Peptides |

Protein Coverage:

1 MSGSGFLLLA LLLLGTVEAT KVEQEKKGMQ TDQLIK**FYDP LPR**LGRQNMQ SPLFSERLRT RALGFRQLSS QKQ

Supporting Peptides:

Peptide	Uniq	-10lgP	Mass	Length	ppm	m/z	z	RT	Fraction	Scan	Source File	Area ebraeus	Area judaeus	#Feature	#Feature ebraeus	#Feature judaeus	Start	End	PTM	AScore	Found By
K.FYDPLPR.L	Y	63.04	906.4599	7	0.8	454.2359	2	35.96	1	F1:32988	20210611-08_2021021-OK192A_815_5.3.236.mgf	0		0	0	0	37	43			PEAKS DB
total 1 peptides																					

OK213_E_288_Conorfamide

back to list

| Protein Coverage | Supporting Peptides |

Protein Coverage:

1 MSGSGFLLLA LLLLGTVEAT KVEQEKKGMQ TDQLIK**FYDP LPR**LGRQNMQ SPLFSERLRT RALGFRQLSS QKQ

Supporting Peptides:

Peptide	Uniq	-10lgP	Mass	Length	ppm	m/z	z	RT	Fraction	Scan	Source File	Area ebraeus	Area judaeus	#Feature	#Feature ebraeus	#Feature judaeus	Start	End	PTM	AScore	Found By
K.FYDPLPR.L	Y	63.04	906.4599	7	0.8	454.2359	2	35.96	1	F1:32988	20210611-08_2021021-OK192A_815_5.3.236.mgf	0		0	0	0	37	43			PEAKS DB
total 1 peptides																					

OK193_E_242_Conorfamide

back to list

| Protein Coverage | Supporting Peptides |

Protein Coverage:

1 MSGSGFLLLA LLLLGTVEAT KVEQEKKGMQ TDQLIK**FYDP LPR**LGRQNMQ SPLFSERLRT RALGFRQLSS QKQ

Supporting Peptides:

Peptide	Uniq	-10lgP	Mass	Length	ppm	m/z	z	RT	Fraction	Scan	Source File	Area ebraeus	Area judaeus	#Feature	#Feature ebraeus	#Feature judaeus	Start	End	PTM	AScore	Found By
K.FYDPLPR.L	Y	63.04	906.4599	7	0.8	454.2359	2	35.96	1	F1:32988	20210611-08_2021021-OK192A_815_5.3.236.mgf	0		0	0	0	37	43			PEAKS DB
total 1 peptides																					

V_1258_138_Q-4

back to list

| Protein Coverage | Supporting Peptides |

Protein Coverage:

1 SLCKTMVCGV ACCPTTNCRC K**LVGAQHFR**Q SVCVC

Supporting Peptides:

Peptide	Uniq	-10lgP	Mass	Length	ppm	m/z	z	RT	Fraction	Scan	Source File	Area ebraeus	Area judaeus	#Feature	#Feature ebraeus	#Feature judaeus	Start	End	PTM	AScore	Found By
K.LVGAQHFR.Q	Y	62.05	926.5086	8	3.0	464.2612	2	17.01	1	F1:8355	20210611-08_2021021-OK192A_815_5.3.236.mgf	0		0	0	0	22	29			PEAKS DB
total 1 peptides																					

V_CG13_043_Q-4

back to list

| Protein Coverage | Supporting Peptides |

Protein Coverage:

1 SSCQTMVCGV ACCPTTNCRC K**LVGAQHFR**K SVCVC

Supporting Peptides:

Peptide	Uniq	-10lgP	Mass	Length	ppm	m/z	z	RT	Fraction	Scan	Source File	Area ebraeus	Area judaeus	#Feature	#Feature ebraeus	#Feature judaeus	Start	End	PTM	AScore	Found By
K.LVGAQHFR.K	Y	62.05	926.5086	8	3.0	464.2612	2	17.01	1	F1:8355	20210611-08_2021021-OK192A_815_5.3.236.mgf	0		0	0	0	22	29			PEAKS DB
total 1 peptides																					

gene:107297725

back to list

| Protein Coverage | Supporting Peptides |

Protein Coverage:

1 MEQAEAHNGS GAAEAPTAGT RPPATAEGMA LAYGSLVVMA LLPIFFGALR SVTCAKGKNA SDMPETITSR DAARFPIVAS
81 CTLLGLYLFF KIFSQEYINL LLSMYFFVLG ILALSHTISP MMNKFFPVNF PNKQYQLLFT QGSGEAKEEI VNYEFDTKDL
161 VCLAMSSIVG VWYLLRKHWI ANNLFGLAFS LNGVELLHLN NVSTGCILLG GLFVYDVFWV FGTNVMVTVA K**SFEAPIK**LV
241 FPQDLLEKGL EADNFAMLGL GDIVIPGIFI ALLLRFDISL KKNTHTYFYT SFVAYILGLS LTIFIMHVFK HAQPALLYLV
321 PACIGFPLLV ALAKGEVTDM FSYEENATSK EVTGDPKEEK TETDPEKKEK

Supporting Peptides:

Peptide	Uniq	-10lgP	Mass	Length	ppm	m/z	z	RT	Fraction	Scan	Source File	Area ebraeus	Area judaeus	#Feature	#Feature ebraeus	#Feature judaeus	Start	End	PTM	AScore	Found By
K.SFEAPIK.L	Y	61.56	790.4225	7	4.1	396.2185	2	25.41	2	F2:75555	20210611-09_2021021-OK160A_816_5.3.236.mgf		0	0	0	0	232	238			PEAKS DB
total 1 peptides																					

OK006_E_160_O2

back to list

| Protein Coverage | Supporting Peptides |

Protein Coverage:

1 CHTNGERCIS DAQCCSNYCR **HTSDYVVSR**C RSLT

Supporting Peptides:

Peptide	Uniq	-10lgP	Mass	Length	ppm	m/z	z	RT	Fraction	Scan	Source File	Area ebraeus	Area judaeus	#Feature	#Feature ebraeus	#Feature judaeus	Start	End	PTM	AScore	Found By
R.HTSDYVVSR.C	Y	61.28	1062.5094	9	0.2	532.2601	2	14.36	1	F1:5671	20210611-08_2021021-OK192A_815_5.3.236.mgf	0		0	0	0	21	29			PEAKS DB
total 1 peptides																					

OK006_E_159_O2

back to list

| Protein Coverage | Supporting Peptides |

Protein Coverage:

1 CHTNGQWCIA DAQCCSNYCR **HTSDYVVSR**C RSLT

Supporting Peptides:

Peptide	Uniq	-10lgP	Mass	Length	ppm	m/z	z	RT	Fraction	Scan	Source File	Area ebraeus	Area judaeus	#Feature	#Feature ebraeus	#Feature judaeus	Start	End	PTM	AScore	Found By
R.HTSDYVVSR.C	Y	61.28	1062.5094	9	0.2	532.2601	2	14.36	1	F1:5671	20210611-08_2021021-OK192A_815_5.3.236.mgf	0		0	0	0	21	29			PEAKS DB
total 1 peptides																					

OK212_J_105_M

back to list

| Protein Coverage | Supporting Peptides |

Protein Coverage:

1 **MTLLLEDGCC TRPR**CTGACS CCQD

Supporting Peptides:

Peptide	Uniq	-10lgP	Mass	Length	ppm	m/z	z	RT	Fraction	Scan	Source File	Area ebraeus	Area judaeus	#Feature	#Feature ebraeus	#Feature judaeus	Start	End	PTM	AScore	Found By
MTLLLEDGC(+57.02)C(+57.02)TRPR.C	Y	61.19	1720.8059	14	1.0	574.6074	3	33.87	2	F2:90467	20210611-09_2021021-OK160A_816_5.3.236.mgf		0	0	0	0	1	14		C9:Carbamidomethylation:1000.00;C10:Carbamidomethylation:1000.00	PEAKS DB
total 1 peptides																					

OK213_E_106_M

back to list

| Protein Coverage | Supporting Peptides |

Protein Coverage:

1 **MTLLLEDGCC TRPR**CTGACS CCQD

Supporting Peptides:

Peptide	Uniq	-10lgP	Mass	Length	ppm	m/z	z	RT	Fraction	Scan	Source File	Area ebraeus	Area judaeus	#Feature	#Feature ebraeus	#Feature judaeus	Start	End	PTM	AScore	Found By
MTLLLEDGC(+57.02)C(+57.02)TRPR.C	Y	61.19	1720.8059	14	1.0	574.6074	3	33.87	2	F2:90467	20210611-09_2021021-OK160A_816_5.3.236.mgf		0	0	0	0	1	14		C9:Carbamidomethylation:1000.00;C10:Carbamidomethylation:1000.00	PEAKS DB
total 1 peptides																					

OK212_J_104_M

back to list

| Protein Coverage | Supporting Peptides |

Protein Coverage:

1 **MTLLLEDGCC TRPR**CTGACS CCQD

Supporting Peptides:

Peptide	Uniq	-10lgP	Mass	Length	ppm	m/z	z	RT	Fraction	Scan	Source File	Area ebraeus	Area judaeus	#Feature	#Feature ebraeus	#Feature judaeus	Start	End	PTM	AScore	Found By
MTLLLEDGC(+57.02)C(+57.02)TRPR.C	Y	61.19	1720.8059	14	1.0	574.6074	3	33.87	2	F2:90467	20210611-09_2021021-OK160A_816_5.3.236.mgf		0	0	0	0	1	14		C9:Carbamidomethylation:1000.00;C10:Carbamidomethylation:1000.00	PEAKS DB
total 1 peptides																					

OK006_E_092_M

back to list

| Protein Coverage | Supporting Peptides |

Protein Coverage:

1 MTLLLEDGCC TRPRCTGACS CCQD

Supporting Peptides:

Peptide	Uniq	-10lgP	Mass	Length	ppm	m/z	z	RT	Fraction	Scan	Source File	Area ebraeus	Area judaeus	#Feature	#Feature ebraeus	#Feature judaeus	Start	End	PTM	AScore	Found By
MTLLLEDGC(+57.02)C(+57.02)TRPR.C	Y	61.19	1720.8059	14	1.0	574.6074	3	33.87	2	F2:90467	20210611-09_2021021-OK160A_816_5.3.236.mgf		0	0	0	0	1	14		C9:Carbamidomethylation:1000.00;C10:Carbamidomethylation:1000.00	PEAKS DB
total 1 peptides																					

OK212_J_106_M

back to list

| Protein Coverage | Supporting Peptides |

Protein Coverage:

1 MTLLLEDGCC TRPRCTGACS CCQD

Supporting Peptides:

Peptide	Uniq	-10lgP	Mass	Length	ppm	m/z	z	RT	Fraction	Scan	Source File	Area ebraeus	Area judaeus	#Feature	#Feature ebraeus	#Feature judaeus	Start	End	PTM	AScore	Found By
MTLLLEDGC(+57.02)C(+57.02)TRPR.C	Y	61.19	1720.8059	14	1.0	574.6074	3	33.87	2	F2:90467	20210611-09_2021021-OK160A_816_5.3.236.mgf		0	0	0	0	1	14		C9:Carbamidomethylation:1000.00;C10:Carbamidomethylation:1000.00	PEAKS DB
total 1 peptides																					

CebrOK033_J_077_M

back to list

| Protein Coverage | Supporting Peptides |

Protein Coverage:

1 MTLLLEDGCC TRPRCTGACS CCQD

Supporting Peptides:

Peptide	Uniq	-10lgP	Mass	Length	ppm	m/z	z	RT	Fraction	Scan	Source File	Area ebraeus	Area judaeus	#Feature	#Feature ebraeus	#Feature judaeus	Start	End	PTM	AScore	Found By
MTLLLEDGC(+57.02)C(+57.02)TRPR.C	Y	61.19	1720.8059	14	1.0	574.6074	3	33.87	2	F2:90467	20210611-09_2021021-OK160A_816_5.3.236.mgf		0	0	0	0	1	14		C9:Carbamidomethylation:1000.00;C10:Carbamidomethylation:1000.00	PEAKS DB
total 1 peptides																					

OK005_J_090_M

back to list

| Protein Coverage | Supporting Peptides |

Protein Coverage:

1 MTLLLEDGCC TRPRCTGACS CCQD

Supporting Peptides:

Peptide	Uniq	-10lgP	Mass	Length	ppm	m/z	z	RT	Fraction	Scan	Source File	Area ebraeus	Area judaeus	#Feature	#Feature ebraeus	#Feature judaeus	Start	End	PTM	AScore	Found By
MTLLLEDGC(+57.02)C(+57.02)TRPR.C	Y	61.19	1720.8059	14	1.0	574.6074	3	33.87	2	F2:90467	20210611-09_2021021-OK160A_816_5.3.236.mgf		0	0	0	0	1	14		C9:Carbamidomethylation:1000.00;C10:Carbamidomethylation:1000.00	PEAKS DB
total 1 peptides																					

OK212_J_101_M

back to list

| Protein Coverage | Supporting Peptides |

Protein Coverage:

1 MTLLLEDGCC TRPRCTGACS CCQD

Supporting Peptides:

Peptide	Uniq	-10lgP	Mass	Length	ppm	m/z	z	RT	Fraction	Scan	Source File	Area ebraeus	Area judaeus	#Feature	#Feature ebraeus	#Feature judaeus	Start	End	PTM	AScore	Found By
MTLLLEDGC(+57.02)C(+57.02)TRPR.C	Y	61.19	1720.8059	14	1.0	574.6074	3	33.87	2	F2:90467	20210611-09_2021021-OK160A_816_5.3.236.mgf		0	0	0	0	1	14		C9:Carbamidomethylation:1000.00;C10:Carbamidomethylation:1000.00	PEAKS DB
total 1 peptides																					

OK005_J_089_M

back to list

| Protein Coverage | Supporting Peptides |

Protein Coverage:

1 MTLLLEDGCC TRPRCTGACS CCQD

Supporting Peptides:

Peptide	Uniq	-10lgP	Mass	Length	ppm	m/z	z	RT	Fraction	Scan	Source File	Area ebraeus	Area judaeus	#Feature	#Feature ebraeus	#Feature judaeus	Start	End	PTM	AScore	Found By
MTLLLEDGC(+57.02)C(+57.02)TRPR.C	Y	61.19	1720.8059	14	1.0	574.6074	3	33.87	2	F2:90467	20210611-09_2021021-OK160A_816_5.3.236.mgf		0	0	0	0	1	14		C9:Carbamidomethylation:1000.00;C10:Carbamidomethylation:1000.00	PEAKS DB
total 1 peptides																					

OK214_J_092_M

back to list

| Protein Coverage | Supporting Peptides |

Protein Coverage:

1 MTLLLEDGCC TRPRCTGACS CCQD

Supporting Peptides:

Peptide	Uniq	-10lgP	Mass	Length	ppm	m/z	z	RT	Fraction	Scan	Source File	Area ebraeus	Area judaeus	#Feature	#Feature ebraeus	#Feature judaeus	Start	End	PTM	AScore	Found By
MTLLLEDGC(+57.02)C(+57.02)TRPR.C	Y	61.19	1720.8059	14	1.0	574.6074	3	33.87	2	F2:90467	20210611-09_2021021-OK160A_816_5.3.236.mgf		0	0	0	0	1	14		C9:Carbamidomethylation:1000.00;C10:Carbamidomethylation:1000.00	PEAKS DB
total 1 peptides																					

OK212_J_103_M

back to list

| Protein Coverage | Supporting Peptides |

Protein Coverage:

1 MTLLLEDGCC TRPRCTGACS CCQD

Supporting Peptides:

Peptide	Uniq	-10lgP	Mass	Length	ppm	m/z	z	RT	Fraction	Scan	Source File	Area ebraeus	Area judaeus	#Feature	#Feature ebraeus	#Feature judaeus	Start	End	PTM	AScore	Found By
MTLLLEDGC(+57.02)C(+57.02)TRPR.C	Y	61.19	1720.8059	14	1.0	574.6074	3	33.87	2	F2:90467	20210611-09_2021021-OK160A_816_5.3.236.mgf		0	0	0	0	1	14		C9:Carbamidomethylation:1000.00;C10:Carbamidomethylation:1000.00	PEAKS DB
total 1 peptides																					

OK214_J_091_M

back to list

| Protein Coverage | Supporting Peptides |

Protein Coverage:

1 MTLLLEDGCC TRPRCTGACS CCQD

Supporting Peptides:

Peptide	Uniq	-10lgP	Mass	Length	ppm	m/z	z	RT	Fraction	Scan	Source File	Area ebraeus	Area judaeus	#Feature	#Feature ebraeus	#Feature judaeus	Start	End	PTM	AScore	Found By
MTLLLEDGC(+57.02)C(+57.02)TRPR.C	Y	61.19	1720.8059	14	1.0	574.6074	3	33.87	2	F2:90467	20210611-09_2021021-OK160A_816_5.3.236.mgf		0	0	0	0	1	14		C9:Carbamidomethylation:1000.00;C10:Carbamidomethylation:1000.00	PEAKS DB
total 1 peptides																					

OK212_J_115_M

back to list

| Protein Coverage | Supporting Peptides |

Protein Coverage:

1 MTLLLEDGCC TRPRCTGSCS CCQD

Supporting Peptides:

Peptide	Uniq	-10lgP	Mass	Length	ppm	m/z	z	RT	Fraction	Scan	Source File	Area ebraeus	Area judaeus	#Feature	#Feature ebraeus	#Feature judaeus	Start	End	PTM	AScore	Found By
MTLLLEDGC(+57.02)C(+57.02)TRPR.C	Y	61.19	1720.8059	14	1.0	574.6074	3	33.87	2	F2:90467	20210611-09_2021021-OK160A_816_5.3.236.mgf		0	0	0	0	1	14		C9:Carbamidomethylation:1000.00;C10:Carbamidomethylation:1000.00	PEAKS DB
total 1 peptides																					

OK212_J_102_M

back to list

| Protein Coverage | Supporting Peptides |

Protein Coverage:

1 MTLLLEDGCC TRPRCTGACS CCQD

Supporting Peptides:

Peptide	Uniq	-10lgP	Mass	Length	ppm	m/z	z	RT	Fraction	Scan	Source File	Area ebraeus	Area judaeus	#Feature	#Feature ebraeus	#Feature judaeus	Start	End	PTM	AScore	Found By
MTLLLEDGC(+57.02)C(+57.02)TRPR.C	Y	61.19	1720.8059	14	1.0	574.6074	3	33.87	2	F2:90467	20210611-09_2021021-OK160A_816_5.3.236.mgf		0	0	0	0	1	14		C9:Carbamidomethylation:1000.00;C10:Carbamidomethylation:1000.00	PEAKS DB
total 1 peptides																					

OK214_J_090_M

back to list

| Protein Coverage | Supporting Peptides |

Protein Coverage:

1 MTLLLEDGCC TRPRCTGACS CCQD

Supporting Peptides:

Peptide	Uniq	-10lgP	Mass	Length	ppm	m/z	z	RT	Fraction	Scan	Source File	Area ebraeus	Area judaeus	#Feature	#Feature ebraeus	#Feature judaeus	Start	End	PTM	AScore	Found By
MTLLLEDGC(+57.02)C(+57.02)TRPR.C	Y	61.19	1720.8059	14	1.0	574.6074	3	33.87	2	F2:90467	20210611-09_2021021-OK160A_816_5.3.236.mgf		0	0	0	0	1	14		C9:Carbamidomethylation:1000.00;C10:Carbamidomethylation:1000.00	PEAKS DB
total 1 peptides																					

OK212_J_109_M

back to list

| Protein Coverage | Supporting Peptides |

Protein Coverage:

1 MTLLLEDGCC TRPRCTGACS CCQD

Supporting Peptides:

Peptide	Uniq	-10lgP	Mass	Length	ppm	m/z	z	RT	Fraction	Scan	Source File	Area ebraeus	Area judaeus	#Feature	#Feature ebraeus	#Feature judaeus	Start	End	PTM	AScore	Found By
MTLLLEDGC(+57.02)C(+57.02)TRPR.C	Y	61.19	1720.8059	14	1.0	574.6074	3	33.87	2	F2:90467	20210611-09_2021021-OK160A_816_5.3.236.mgf		0	0	0	0	1	14		C9:Carbamidomethylation:1000.00;C10:Carbamidomethylation:1000.00	PEAKS DB
total 1 peptides																					

OK212_J_108_M

back to list

| Protein Coverage | Supporting Peptides |

Protein Coverage:

1 MTLLLEDGCC TRPRCTGACS CCQD

Supporting Peptides:

Peptide	Uniq	-10lgP	Mass	Length	ppm	m/z	z	RT	Fraction	Scan	Source File	Area ebraeus	Area judaeus	#Feature	#Feature ebraeus	#Feature judaeus	Start	End	PTM	AScore	Found By
MTLLLEDGC(+57.02)C(+57.02)TRPR.C	Y	61.19	1720.8059	14	1.0	574.6074	3	33.87	2	F2:90467	20210611-09_2021021-OK160A_816_5.3.236.mgf		0	0	0	0	1	14		C9:Carbamidomethylation:1000.00;C10:Carbamidomethylation:1000.00	PEAKS DB
total 1 peptides																					

CebrOK035_J_095_M

back to list

| Protein Coverage | Supporting Peptides |

Protein Coverage:

1 MTLLLEDGCC TRPRCTGACS CCQD

Supporting Peptides:

Peptide	Uniq	-10lgP	Mass	Length	ppm	m/z	z	RT	Fraction	Scan	Source File	Area ebraeus	Area judaeus	#Feature	#Feature ebraeus	#Feature judaeus	Start	End	PTM	AScore	Found By
MTLLLEDGC(+57.02)C(+57.02)TRPR.C	Y	61.19	1720.8059	14	1.0	574.6074	3	33.87	2	F2:90467	20210611-09_2021021-OK160A_816_5.3.236.mgf		0	0	0	0	1	14		C9:Carbamidomethylation:1000.00;C10:Carbamidomethylation:1000.00	PEAKS DB
total 1 peptides																					

OK212_J_087_M

back to list

| Protein Coverage | Supporting Peptides |

Protein Coverage:

1 **MTLLLEDGCC TRPR**CTGACS CCQD

Supporting Peptides:

Peptide	Uniq	-10lgP	Mass	Length	ppm	m/z	z	RT	Fraction	Scan	Source File	Area ebraeus	Area judaeus	#Feature	#Feature ebraeus	#Feature judaeus	Start	End	PTM	AScore	Found By
MTLLLEDGC(+57.02)C(+57.02)TRPR.C	Y	61.19	1720.8059	14	1.0	574.6074	3	33.87	2	F2:90467	20210611-09_2021021-OK160A_816_5.3.236.mgf		0	0	0	0	1	14		C9:Carbamidomethylation:1000.00;C10:Carbamidomethylation:1000.00	PEAKS DB
total 1 peptides																					

OK214_J_095_M

back to list

| Protein Coverage | Supporting Peptides |

Protein Coverage:

1 **MTLLLEDGCC TRPR**FTGACS CCQD

Supporting Peptides:

Peptide	Uniq	-10lgP	Mass	Length	ppm	m/z	z	RT	Fraction	Scan	Source File	Area ebraeus	Area judaeus	#Feature	#Feature ebraeus	#Feature judaeus	Start	End	PTM	AScore	Found By
MTLLLEDGC(+57.02)C(+57.02)TRPR.F	Y	61.19	1720.8059	14	1.0	574.6074	3	33.87	2	F2:90467	20210611-09_2021021-OK160A_816_5.3.236.mgf		0	0	0	0	1	14		C9:Carbamidomethylation:1000.00;C10:Carbamidomethylation:1000.00	PEAKS DB
total 1 peptides																					

DAC80618_TPA_inf_conodipine_partial_Conus_magus

back to list

| Protein Coverage | Supporting Peptides |

Protein Coverage:

1 QDPSTAELCK INSNACSVPF SWIPCQK**HFL AACDR**HDTCY HCGKHFGFKQ DDCDDAFFRD MTALCAHGTD DEGSCPEKRK

81 RREASSMSIT TPLRQLRQLE KLVLPNSLSA RDPRQLHSRA ATCTHWALIY FKTVQLFGWW HFNYQVDATY CPQFQPCMPQ

161 V

Supporting Peptides:

Peptide	Uniq	-10lgP	Mass	Length	ppm	m/z	z	RT	Fraction	Scan	Source File	Area ebraeus	Area judaeus	#Feature	#Feature ebraeus	#Feature judaeus	Start	End	PTM	AScore	Found By
K.HFLAAC(+57.02)DR.H	Y	60.77	988.4548	8	2.3	495.2339	2	17.12	1	F1:8423	20210611-08_2021021-OK192A_815_5.3.236.mgf	0		0	0	0	28	35		C6:Carbamidomethylation:1000.00	PEAKS DB
total 1 peptides																					

OK212_J_293_Conodipine

back to list

| Protein Coverage | Supporting Peptides |

Protein Coverage:

1 MKMLESALWI LAALALPWIV AQDSSTAELC KINSNGCSVP SALIQCRK**HF LAACDR**HDNC YFCGAHFGLT QSDCDNAFLT

81 HMTALCAQGT DDEGFCLERR KRRDASSMFI TSTPLRQLRL LEKLMPPNSL LDRHPRRRRF VTCTEWAEYY YRAVRLAGRW

161 YFYETADATY CPWFKPCVP

Supporting Peptides:

Peptide	Uniq	-10lgP	Mass	Length	ppm	m/z	z	RT	Fraction	Scan	Source File	Area ebraeus	Area judaeus	#Feature	#Feature ebraeus	#Feature judaeus	Start	End	PTM	AScore	Found By
K.HFLAAC(+57.02)DR.H	Y	60.77	988.4548	8	2.3	495.2339	2	17.12	1	F1:8423	20210611-08_2021021-OK192A_815_5.3.236.mgf	0		0	0	0	49	56		C6:Carbamidomethylation:1000.00	PEAKS DB
total 1 peptides																					

OK212_J_292_Conodipine

back to list

| Protein Coverage | Supporting Peptides |

Protein Coverage:

1 MKMLESALWI LAALALPWIV AQDSSTAELC KINSNNCSVP FEWIPCQK**HF LAACDR**HDNC YFCGAHFGLT QSDCDNAFLT

81 HMTALCAQGT DDEGFCLERR KRRDASSMFI TSTPLRQLRL LEKLMPPNSL LDRHPRRRRF VTCTEWAEYY YRAVRLAGRW

161 YFYETADATY CPWFKPCVP

Supporting Peptides:

Peptide	Uniq	-10lgP	Mass	Length	ppm	m/z	z	RT	Fraction	Scan	Source File	Area ebraeus	Area judaeus	#Feature	#Feature ebraeus	#Feature judaeus	Start	End	PTM	AScore	Found By
K.HFLAAC(+57.02)DR.H	Y	60.77	988.4548	8	2.3	495.2339	2	17.12	1	F1:8423	20210611-08_2021021-OK192A_815_5.3.236.mgf	0		0	0	0	49	56		C6:Carbamidomethylation:1000.00	PEAKS DB
total 1 peptides																					

V_CG13_220_Conodipine-2

back to list

| Protein Coverage | Supporting Peptides |

Protein Coverage:

1 NSNGCSVPVS FIPCQK**HFLA ACDR**HDTCYF CGAHFGFTQD DCDNAFLTDM AVLCADGTDD EGFCLEKRKR REASSMSITT

81 PLRQLRLLEK LMPLNSLSDR DPRQPHRRIG TCTTWSEYYF QGVQTMGSWY FYKTANATHC PKFKACMPVV

Supporting Peptides:

Peptide	Uniq	-10lgP	Mass	Length	ppm	m/z	z	RT	Fraction	Scan	Source File	Area ebraeus	Area judaeus	#Feature	#Feature ebraeus	#Feature judaeus	Start	End	PTM	AScore	Found By
K.HFLAAC(+57.02)DR.H	Y	60.77	988.4548	8	2.3	495.2339	2	17.12	1	F1:8423	20210611-08_2021021-OK192A_815_5.3.236.mgf	0		0	0	0	17	24		C6:Carbamidomethylation:1000.00	PEAKS DB
total 1 peptides																					

V_1258_222_Conodipine-2

back to list

| Protein Coverage | Supporting Peptides |

Protein Coverage:

1 MKVLESALWI LAALALPWIA AQDISTAELC KINSNGCSVP VSFIPCQK**HF LAACDR**HDTC YFCGAHFGFT QDDCDNAFLT

81 DMAALCADGT DDEGFCLEKR KRREASSMSI TTPLRQLRLL EKLMPLNSLS DRDPRQPHRR IGTCTTWSEY YFQGVQTMGS

161 WYFYKTSNAT HCPKFKACMP VV

Supporting Peptides:

Peptide	Uniq	-10lgP	Mass	Length	ppm	m/z	z	RT	Fraction	Scan	Source File	Area ebraeus	Area judaeus	#Feature	#Feature ebraeus	#Feature judaeus	Start	End	PTM	AScore	Found By
K.HFLAAC(+57.02)DR.H	Y	60.77	988.4548	8	2.3	495.2339	2	17.12	1	F1:8423	20210611-08_2021021-OK192A_815_5.3.236.mgf	0		0	0	0	49	56		C6:Carbamidomethylation:1000.00	PEAKS DB
total 1 peptides																					

V_1302_262_Conodipine-2

back to list

| Protein Coverage | Supporting Peptides |

Protein Coverage:

1 MKMLESALWI LAALALPWIA AQDSSTAELC KINSNGCSVP VSFIPCQK**HF LAACDR**HDTC YFCGAHFGFT QDDCDNAFLT

81 DMAALCADGT DDEGFCLEKR KRREASSMSI TTPLRQLRLL EKLMPLNSLS DRDPRHLPQR AFTCTEWASH YFEGVQAFGS

161 WHFNNTASAT YCPQFEACMP VV

Supporting Peptides:

Peptide	Uniq	-10lgP	Mass	Length	ppm	m/z	z	RT	Fraction	Scan	Source File	Area ebraeus	Area judaeus	#Feature	#Feature ebraeus	#Feature judaeus	Start	End	PTM	AScore	Found By
K.HFLAAC(+57.02)DR.H	Y	60.77	988.4548	8	2.3	495.2339	2	17.12	1	F1:8423	20210611-08_2021021-OK192A_815_5.3.236.mgf	0		0	0	0	49	56		C6:Carbamidomethylation:1000.00	PEAKS DB
total 1 peptides																					

V_1302_263_Conodipine-2

back to list

| Protein Coverage | Supporting Peptides |

Protein Coverage:

1 MKMLESALWI LAALALPWIA AQDSSTAELC KINSNGCSVP VSFIPCQK**HF LAACDR**HDTC YFCGAHFGFT QDDCDNAFLT

81 DMAALCADGT DDEGFCLEKR KRREASSMSI TTPLRQLRLL EKLMPLNSLS DRDPRQPHRR IGTCTTWSEY YFQGVQTMGS

161 WYFYKTSNAT HCPKFKACMP VV

Supporting Peptides:

Peptide	Uniq	-10lgP	Mass	Length	ppm	m/z	z	RT	Fraction	Scan	Source File	Area ebraeus	Area judaeus	#Feature	#Feature ebraeus	#Feature judaeus	Start	End	PTM	AScore	Found By
K.HFLAAC(+57.02)DR.H	Y	60.77	988.4548	8	2.3	495.2339	2	17.12	1	F1:8423	20210611-08_2021021-OK192A_815_5.3.236.mgf	0		0	0	0	49	56		C6:Carbamidomethylation:1000.00	PEAKS DB
total 1 peptides																					

CV8_259_Conodipine

back to list

| Protein Coverage | Supporting Peptides |

Protein Coverage:

1 MKVLESALWI LAALALPWIA AQDISTAELC KINSNGCSVP VSWIPCQK**HF LAACDR**HDTC YFCGAHFGFT QDDCDNAFLT

81 DMAALCAHGT DDKGFCLEKR KRREASSMSI TTPLRQLRLL EKLMPLNSLS DRDPRQPHRR AAATCTEWAE TYFEGVQNFA

161 SSHFNYTASA TYCPQFEACM PVV

Supporting Peptides:

Peptide	Uniq	-10lgP	Mass	Length	ppm	m/z	z	RT	Fraction	Scan	Source File	Area ebraeus	Area judaeus	#Feature	#Feature ebraeus	#Feature judaeus	Start	End	PTM	AScore	Found By
K.HFLAAC(+57.02)DR.H	Y	60.77	988.4548	8	2.3	495.2339	2	17.12	1	F1:8423	20210611-08_2021021-OK192A_815_5.3.236.mgf	0		0	0	0	49	56		C6:Carbamidomethylation:1000.00	PEAKS DB
total 1 peptides																					

OK212_J_290_Conodipine

back to list

| Protein Coverage | Supporting Peptides |

Protein Coverage:

1 MKMLESALWI LAALALPWIV AQDSSTAELC KINSNGCSVP SALIQCRK**HF LAACDR**HDNC YFCGAHFGLT QSDCDNAFLT

81 HMTALCAQGT DDEGFCLERR KRRDASSMFI TSTPLRQLRL LEKLMPPNSL LDHDSRQPQQ RFFTCTKWAK KYFDAVRTSG

161 SSYFSDTANA TYCPQFVDCM PQV

Supporting Peptides:

Peptide	Uniq	-10lgP	Mass	Length	ppm	m/z	z	RT	Fraction	Scan	Source File	Area ebraeus	Area judaeus	#Feature	#Feature ebraeus	#Feature judaeus	Start	End	PTM	AScore	Found By
K.HFLAAC(+57.02)DR.H	Y	60.77	988.4548	8	2.3	495.2339	2	17.12	1	F1:8423	20210611-08_2021021-OK192A_815_5.3.236.mgf	0		0	0	0	49	56		C6:Carbamidomethylation:1000.00	PEAKS DB
total 1 peptides																					

OK212_J_291_Conodipine

back to list

| Protein Coverage | Supporting Peptides |

Protein Coverage:

1 MKMLESALWI LAALALPWIV AQDSSTAELC KINSNNCSVP FEWIPCQK**HF LAACDR**HDNC YFCGAHFGLT QSDCDNAFLT

81 HMTALCAQGT DDEGFCLERR KRRDASSMFI TSTPLRQLRL LEKLMPPNSL LDHDSRQPQQ RFFTCTKWAK KYFDAVRTSG

161 SSYFSDTANA TYCPQFVDCM PQV

Supporting Peptides:

Peptide	Uniq	-10lgP	Mass	Length	ppm	m/z	z	RT	Fraction	Scan	Source File	Area ebraeus	Area judaeus	#Feature	#Feature ebraeus	#Feature judaeus	Start	End	PTM	AScore	Found By
K.HFLAAC(+57.02)DR.H	Y	60.77	988.4548	8	2.3	495.2339	2	17.12	1	F1:8423	20210611-08_2021021-OK192A_815_5.3.236.mgf	0		0	0	0	49	56		C6:Carbamidomethylation:1000.00	PEAKS DB
total 1 peptides																					

gene:107302264

back to list

| Protein Coverage | Supporting Peptides |

Protein Coverage:

1 SLVIPEKFQH ILR**VLNTNID GR**RKIAFAIT AIKGVGRRYA HVVLRKADID LTKRAGELTE DEVERVITIM QNPRQYKIPD

81 WFLNRQKDVK DGKYSQ

Supporting Peptides:

Peptide	Uniq	-10lgP	Mass	Length	ppm	m/z	z	RT	Fraction	Scan	Source File	Area ebraeus	Area judaeus	#Feature	#Feature ebraeus	#Feature judaeus	Start	End	PTM	AScore	Found By
R.VLNTNIDGR.R	Y	60.44	1000.5302	9	4.6	501.2726	2	21.59	2	F2:68645	20210611-09_2021021-OK160A_816_5.3.236.mgf		0	0	0	0	14	22			PEAKS DB
total 1 peptides																					

gene:107302095

back to list

| Protein Coverage | Supporting Peptides |

Protein Coverage:

1 MSLVIPEKFQ HILRVLNTNI DGRRKIAFAI TAIKGVGRRY AHVVLRKADI DLTKLAGELT EDEVERVITI MQNPRQYKIP

81 DWFLNRQKDV KDGKYSQVLA NGLDNKLRED LERLKKIRAH RGLRHFWGLR VRGQHTKTTG RRGRTVGVSK KK

Supporting Peptides:

Peptide	Uniq	-10lgP	Mass	Length	ppm	m/z	z	RT	Fraction	Scan	Source File	Area ebraeus	Area judaeus	#Feature	#Feature ebraeus	#Feature judaeus	Start	End	PTM	AScore	Found By
R.VLNTNIDGR.R	Y	60.44	1000.5302	9	4.6	501.2726	2	21.59	2	F2:68645	20210611-09_2021021-OK160A_816_5.3.236.mgf		0	0	0	0	15	23			PEAKS DB
total 1 peptides																					

A_0239_128_M-3a-WF

back to list

| Protein Coverage | Supporting Peptides |

Protein Coverage:

1 DQPVERYAEN KQDLNPDERR EIILPALR

Supporting Peptides:

Peptide	Uniq	-10lgP	Mass	Length	ppm	m/z	z	RT	Fraction	Scan	Source File	Area ebraeus	Area judaeus	#Feature	#Feature ebraeus	#Feature judaeus	Start	End	PTM	AScore	Found By
K.QDLNPDER.R	Y	60.04	985.4465	8	4.4	493.7308	2	15.11	1	F1:6285	20210611-08_2021021-OK192A_815_5.3.236.mgf	0		0	0	0	12	19			PEAKS DB
total 1 peptides																					

A_0239_131_M-3a-WF

back to list

| Protein Coverage | Supporting Peptides |

Protein Coverage:

1 DQPVERYAEN KQDLNPDERR EIILPALRQ

Supporting Peptides:

Peptide	Uniq	-10lgP	Mass	Length	ppm	m/z	z	RT	Fraction	Scan	Source File	Area ebraeus	Area judaeus	#Feature	#Feature ebraeus	#Feature judaeus	Start	End	PTM	AScore	Found By
K.QDLNPDER.R	Y	60.04	985.4465	8	4.4	493.7308	2	15.11	1	F1:6285	20210611-08_2021021-OK192A_815_5.3.236.mgf	0		0	0	0	12	19			PEAKS DB
total 1 peptides																					

OK214_J_079_M

back to list

| Protein Coverage | Supporting Peptides |

Protein Coverage:

1 DQPVERYAEN KQDLNPDERG

Supporting Peptides:

Peptide	Uniq	-10lgP	Mass	Length	ppm	m/z	z	RT	Fraction	Scan	Source File	Area ebraeus	Area judaeus	#Feature	#Feature ebraeus	#Feature judaeus	Start	End	PTM	AScore	Found By
K.QDLNPDER.G	Y	60.04	985.4465	8	4.4	493.7308	2	15.11	1	F1:6285	20210611-08_2021021-OK192A_815_5.3.236.mgf	0		0	0	0	12	19			PEAKS DB
total 1 peptides																					

A_1387_079

back to list

| Protein Coverage | Supporting Peptides |

Protein Coverage:

1 DQPVERYAEN KQDLNPDERR E

Supporting Peptides:

Peptide	Uniq	-10lgP	Mass	Length	ppm	m/z	z	RT	Fraction	Scan	Source File	Area ebraeus	Area judaeus	#Feature	#Feature ebraeus	#Feature judaeus	Start	End	PTM	AScore	Found By
K.QDLNPDER.R	Y	60.04	985.4465	8	4.4	493.7308	2	15.11	1	F1:6285	20210611-08_2021021-OK192A_815_5.3.236.mgf	0		0	0	0	12	19			PEAKS DB
total 1 peptides																					

OK006_E_081_M

back to list

| Protein Coverage | Supporting Peptides |

Protein Coverage:

1 QLNADQHVKR YAENKQDLNP DERKG

Supporting Peptides:

Peptide	Uniq	-10lgP	Mass	Length	ppm	m/z	z	RT	Fraction	Scan	Source File	Area ebraeus	Area judaeus	#Feature	#Feature ebraeus	#Feature judaeus	Start	End	PTM	AScore	Found By
K.QDLNPDER.K	Y	60.04	985.4465	8	4.4	493.7308	2	15.11	1	F1:6285	20210611-08_2021021-OK192A_815_5.3.236.mgf	0		0	0	0	16	23			PEAKS DB
total 1 peptides																					

OK212_J_098_M

back to list

| Protein Coverage | Supporting Peptides |

Protein Coverage:

1 DQPVERYAEN KQDLNPDERR GCILPAL

Supporting Peptides:

Peptide	Uniq	-10lgP	Mass	Length	ppm	m/z	z	RT	Fraction	Scan	Source File	Area ebraeus	Area judaeus	#Feature	#Feature ebraeus	#Feature judaeus	Start	End	PTM	AScore	Found By
K.QDLNPDER.R	Y	60.04	985.4465	8	4.4	493.7308	2	15.11	1	F1:6285	20210611-08_2021021-OK192A_815_5.3.236.mgf	0		0	0	0	12	19			PEAKS DB
total 1 peptides																					

K_0010_334_M-3a

back to list

| Protein Coverage | Supporting Peptides |

Protein Coverage:

1 DQPIERYAEN KQDLNPDERL EFILHALGQR

Supporting Peptides:

Peptide	Uniq	-10lgP	Mass	Length	ppm	m/z	z	RT	Fraction	Scan	Source File	Area ebraeus	Area judaeus	#Feature	#Feature ebraeus	#Feature judaeus	Start	End	PTM	AScore	Found By
K.QDLNPDER.L	Y	60.04	985.4465	8	4.4	493.7308	2	15.11	1	F1:6285	20210611-08_2021021-OK192A_815_5.3.236.mgf	0		0	0	0	12	19			PEAKS DB
total 1 peptides																					

A_1387_077

[back to list]

| **Protein Coverage** | **Supporting Peptides** |

Protein Coverage:

1 DQPVERYAEN K**QDLNPDER**M EFILHALGQR R

Supporting Peptides:

Peptide	Uniq	-10lgP	Mass	Length	ppm	m/z	z	RT	Fraction	Scan	Source File	Area ebraeus	Area judaeus	#Feature	#Feature ebraeus	#Feature judaeus	Start	End	PTM	AScore	Found By
K.QDLNPDER.M	Y	60.04	985.4465	8	4.4	493.7308	2	15.11	1	F1:6285	20210611-08_2021021-OK192A_815_5.3.236.mgf	0		0	0	0	12	19			PEAKS DB
total 1 peptides																					

K_0010_335_M-3a-WF

[back to list]

| **Protein Coverage** | **Supporting Peptides** |

Protein Coverage:

1 QLDADQTVER YAENK**QDLNP DER**LEFILHA LGQR

Supporting Peptides:

Peptide	Uniq	-10lgP	Mass	Length	ppm	m/z	z	RT	Fraction	Scan	Source File	Area ebraeus	Area judaeus	#Feature	#Feature ebraeus	#Feature judaeus	Start	End	PTM	AScore	Found By
K.QDLNPDER.L	Y	60.04	985.4465	8	4.4	493.7308	2	15.11	1	F1:6285	20210611-08_2021021-OK192A_815_5.3.236.mgf	0		0	0	0	16	23			PEAKS DB
total 1 peptides																					

AXL95709_conotoxin_precursor_superfamily_M_partial_Conus

[back to list]

| **Protein Coverage** | **Supporting Peptides** |

Protein Coverage:

1 DADQPVERHA ENK**QDLNPDE R**REIILPGLR HGRPSRSYGW RTG

Supporting Peptides:

Peptide	Uniq	-10lgP	Mass	Length	ppm	m/z	z	RT	Fraction	Scan	Source File	Area ebraeus	Area judaeus	#Feature	#Feature ebraeus	#Feature judaeus	Start	End	PTM	AScore	Found By
K.QDLNPDER.R	Y	60.04	985.4465	8	4.4	493.7308	2	15.11	1	F1:6285	20210611-08_2021021-OK192A_815_5.3.236.mgf	0		0	0	0	14	21			PEAKS DB
total 1 peptides																					

DAC80580_TPA_inf_conotoxin_superfamily_M_Conus

[back to list]

| **Protein Coverage** | **Supporting Peptides** |

Protein Coverage:

1 QLDADQPVER HVENK**QDLNP DER**RQIKFDA LRHGRLSRSS GWRTG

Supporting Peptides:

Peptide	Uniq	-10lgP	Mass	Length	ppm	m/z	z	RT	Fraction	Scan	Source File	Area ebraeus	Area judaeus	#Feature	#Feature ebraeus	#Feature judaeus	Start	End	PTM	AScore	Found By
K.QDLNPDER.R	Y	60.04	985.4465	8	4.4	493.7308	2	15.11	1	F1:6285	20210611-08_2021021-OK192A_815_5.3.236.mgf	0		0	0	0	16	23			PEAKS DB
total 1 peptides																					

QFQ61048_conotoxin_superfamily_M_Conus_magus

[back to list]

| **Protein Coverage** | **Supporting Peptides** |

Protein Coverage:

1 QLDADQPVER HVENK**QDLNP DER**RGILLHA LGHLMSSSPN GWWSR

Supporting Peptides:

Peptide	Uniq	-10lgP	Mass	Length	ppm	m/z	z	RT	Fraction	Scan	Source File	Area ebraeus	Area judaeus	#Feature	#Feature ebraeus	#Feature judaeus	Start	End	PTM	AScore	Found By
K.QDLNPDER.R	Y	60.04	985.4465	8	4.4	493.7308	2	15.11	1	F1:6285	20210611-08_2021021-OK192A_815_5.3.236.mgf	0		0	0	0	16	23			PEAKS DB
total 1 peptides																					

gene:107283272

[back to list]

| **Protein Coverage** | **Supporting Peptides** |

Protein Coverage:

1 MAKVTFKVTL TSDPRLPYKV LSVPEKTPFT AVLK**FAAEEF K**VPAATSAII TNDGIGINPD QTAGNVFLKY GSELRLIPRD

81 RVGRYWHMQG PLILREQK

Supporting Peptides:

Peptide	Uniq	-10lgP	Mass	Length	ppm	m/z	z	RT	Fraction	Scan	Source File	Area ebraeus	Area judaeus	#Feature	#Feature ebraeus	#Feature judaeus	Start	End	PTM	AScore	Found By
K.FAAEEFK.V	Y	59.84	840.4017	7	4.4	421.2082	2	24.99	2	F2:74748	20210611-09_2021021-OK160A_816_5.3.236.mgf	0	0	0	0	0	35	41			PEAKS DB
total 1 peptides																					

gene:107295193

[back to list]

| **Protein Coverage** | **Supporting Peptides** |

Protein Coverage:

1 MAAPGGWSSV LWWLLLAAGI APRAWGLYFH LGETERKCFI EEIPDETMVI GNYRTQLYDK QREEYLPATP GLGMFVEVKD

81 PDEKVVLSRQ YGSEGRFIFT SHTPGEHQIC LHSNSTKFSL FAGGMLRVHL DIQVGEHAND YAEIAAK**DKL SELQLR**VRQL

161 LEQIEQIQKE QNYQRWREER FRQTSESTNQ RVLWWSIVQT LILVVIGVWQ MRHLKSFFEA KKLV

Supporting Peptides:

Peptide	Uniq	-10lgP	Mass	Length	ppm	m/z	z	RT	Fraction	Scan	Source File	Area ebraeus	Area judaeus	#Feature	#Feature ebraeus	#Feature judaeus	Start	End	PTM	AScore	Found By
K.DKLSELQLR.V	Y	58.82	1100.6189	9	5.4	551.3174	2	28.68	2	F2:81416	20210611-09_2021021-OK160A_816_5.3.236.mgf		0	0	0	0	148	156			PEAKS DB
total 1 peptides																					

OK214_J_161_O2

[back to list]

| **Protein Coverage** | **Supporting Peptides** |

Protein Coverage:

1 DGVKSPR**EKI NFLSK**RKTNA ESWWE

Supporting Peptides:

Peptide	Uniq	-10lgP	Mass	Length	ppm	m/z	z	RT	Fraction	Scan	Source File	Area ebraeus	Area judaeus	#Feature	#Feature ebraeus	#Feature judaeus	Start	End	PTM	AScore	Found By

Peptide	Uniq	-10lgP	Mass	Length	ppm	m/z	z	RT	Fraction	Scan	Source File	Area ebraeus	Area judaeus	#Feature	#Feature ebraeus	#Feature judaeus	Start	End	PTM	AScore	Found By
R.EKINFLSK.R	Y	58.28	977.5546	8	1.7	489.7833	2	25.75	2	F2:75858	20210611-09_2021021-OK160A_816_5.3.236.mgf		0	0	0	0	8	15			PEAKS DB
**total 1 peptides**																					

OK214_J_169_O2

back to list

| Protein Coverage | Supporting Peptides |

Protein Coverage:

1 LNQGGEVKSP R**EKINFLSK**R ESAAESRWEG EC

Supporting Peptides:

Peptide	Uniq	-10lgP	Mass	Length	ppm	m/z	z	RT	Fraction	Scan	Source File	Area ebraeus	Area judaeus	#Feature	#Feature ebraeus	#Feature judaeus	Start	End	PTM	AScore	Found By
R.EKINFLSK.R	Y	58.28	977.5546	8	1.7	489.7833	2	25.75	2	F2:75858	20210611-09_2021021-OK160A_816_5.3.236.mgf		0	0	0	0	12	19			PEAKS DB
**total 1 peptides**																					

OK006_E_230_Ggeo01

back to list

| Protein Coverage | Supporting Peptides |

Protein Coverage:

1 SQTDDSSTEK RSRSMPDVDF NLQFDK**IGDS SDFGK**GFPKR K

Supporting Peptides:

Peptide	Uniq	-10lgP	Mass	Length	ppm	m/z	z	RT	Fraction	Scan	Source File	Area ebraeus	Area judaeus	#Feature	#Feature ebraeus	#Feature judaeus	Start	End	PTM	AScore	Found By
K.IGDSSDFGK.G	Y	58.10	924.4188	9	2.7	463.2162	2	18.36	1	F1:9680	20210611-08_2021021-OK192A_815_5.3.236.mgf	0		0	0	0	27	35			PEAKS DB
**total 1 peptides**																					

P08581

back to list

| Protein Coverage | Supporting Peptides |

Protein Coverage:

1 **EPMQGMEAEG HFVR**TDCDEG CMKCGSGCCC SPNSCIDSQC RGQVPPQ

O Oxidation (M) (+15.99)

Supporting Peptides:

Peptide	Uniq	-10lgP	Mass	Length	ppm	m/z	z	RT	Fraction	Scan	Source File	Area ebraeus	Area judaeus	#Feature	#Feature ebraeus	#Feature judaeus	Start	End	PTM	AScore	Found By
EPM(+15.99)QGM(+15.99)EAEGHFVR.T	Y	58.09	1648.6974	14	2.9	550.5726	3	19.29	1	F1:10592	20210611-08_2021021-OK192A_815_5.3.236.mgf	0		0	0	0	1	14	Oxidation (M)	M3:Oxidation (M):1000.00;M6:Oxidation (M):1000.00	PEAKS DB
**total 1 peptides**																					

OK193_E_233_Sf-mi2

back to list

| Protein Coverage | Supporting Peptides |

Protein Coverage:

1 **EPMQGMEAEG HFVR**TDCEED CGTCGSGCCC APDSCIDSRC RSHVGPN

O Oxidation (M) (+15.99)

Supporting Peptides:

Peptide	Uniq	-10lgP	Mass	Length	ppm	m/z	z	RT	Fraction	Scan	Source File	Area ebraeus	Area judaeus	#Feature	#Feature ebraeus	#Feature judaeus	Start	End	PTM	AScore	Found By
EPM(+15.99)QGM(+15.99)EAEGHFVR.T	Y	58.09	1648.6974	14	2.9	550.5726	3	19.29	1	F1:10592	20210611-08_2021021-OK192A_815_5.3.236.mgf	0		0	0	0	1	14	Oxidation (M)	M3:Oxidation (M):1000.00;M6:Oxidation (M):1000.00	PEAKS DB
**total 1 peptides**																					

gene:107292229

back to list

| Protein Coverage | Supporting Peptides |

Protein Coverage:

1 MSTAPAGPEY SSFFAVMGAS AAMIFSALGA AYGTAK**SGTG IAAMSVMRPE LIMK**SIIPVV MAGIIAIYGL VVAVLIASSL

81 VHDISLFKSF LQLGAGLSVG LSGLAAGFAI GIVGDAGVRG TAQQPRLFVG MILILIFAEV LGLYGLIVGL ILSTKG

Supporting Peptides:

Peptide	Uniq	-10lgP	Mass	Length	ppm	m/z	z	RT	Fraction	Scan	Source File	Area ebraeus	Area judaeus	#Feature	#Feature ebraeus	#Feature judaeus	Start	End	PTM	AScore	Found By
K.SGTGIAAM(+15.99)SVM(+15.99)RPELIM(+15.99)K.S	Y	58.05	1938.9576	18	2.2	970.4841	2	30.89	2	F2:84821	20210611-09_2021021-OK160A_816_5.3.236.mgf		0	0	0	0	37	54		M8:Oxidation(M):1000.00; M11:Oxidation(M):1000.00; M17:Oxidation(M):1000.00	PEAKS DB
**total 1 peptides**																					

OK214_J_009_B2

back to list

| Protein Coverage | Supporting Peptides |

Protein Coverage:

1 APANVDPNLQ PFGGALTIPN MQPMQGMTGM PSPQFLPFNP NFAVGLKREN LEKRRHNPQF SDNNQSPFGS EGDLDSFMNF

81 MK**ENGNNLPF ADVN**

Supporting Peptides:

Peptide	Uniq	-10lgP	Mass	Length	ppm	m/z	z	RT	Fraction	Scan	Source File	Area ebraeus	Area judaeus	#Feature	#Feature ebraeus	#Feature judaeus	Start	End	PTM	AScore	Found By
K.ENGNNLPFADVN	Y	58.02	1302.5840	12	-0.7	652.2963	2	40.79	1	F1:38394	20210611-08_2021021-OK192A_815_5.3.236.mgf	0		0	0	0	83	94			PEAKS DB
**total 1 peptides**																					

OK214_J_276_Rimp01

back to list

| Protein Coverage | Supporting Peptides |

Protein Coverage:

1 AGIQYSAESP MQQCSTHAVS K**MEVYSNNQG K**YELLDSYCS CDSGEAYFEA QDTTTSSSTV SSYKI

O Oxidation (M) (+15.99)

Supporting Peptides:

Peptide	Uniq	-10lgP	Mass	Length	ppm	m/z	z	RT	Fraction	Scan	Source File	Area ebraeus	Area judaeus	#Feature	#Feature ebraeus	#Feature judaeus	Start	End	PTM	AScore	Found By
K.M(+15.99)EVYSNNQGK.Y	Y	57.67	1184.5132	10	1.4	593.2625	2	11.79	1	F1:2928	20210611-08_2021021-OK192A_815_5.3.236.mgf	0		0	0	0	22	31	Oxidation (M)	M1:Oxidation (M):1000.00	PEAKS DB
total 1 peptides																					

OK006_E_239_Rimp01

back to list

| Protein Coverage | Supporting Peptides |

Protein Coverage:

1 AGIQYSAESP MQQCSTHAVS KMEVYSNNQG KYELLDSYCS CDSGEAYFEA QDTTTSSSTV SSYKIYVCGM PTESCTGGTL

81 PVTDSDNGPR RMQCTCGQYK YLVSASLGWH VRC

Oxidation (M) (+15.99)

Supporting Peptides:

Peptide	Uniq	-10lgP	Mass	Length	ppm	m/z	z	RT	Fraction	Scan	Source File	Area ebraeus	Area judaeus	#Feature	#Feature ebraeus	#Feature judaeus	Start	End	PTM	AScore	Found By
K.M(+15.99)EVYSNNQGK.Y	Y	57.67	1184.5132	10	1.4	593.2625	2	11.79	1	F1:2928	20210611-08_2021021-OK192A_815_5.3.236.mgf	0		0	0	0	22	31	Oxidation (M)	M1:Oxidation (M):1000.00	PEAKS DB
total 1 peptides																					

OK213_E_266_Rimp01

back to list

| Protein Coverage | Supporting Peptides |

Protein Coverage:

1 AGIQYSAESP MQQCSTHAVS KMEVYSNNQG KYELLDSYCS CDSGEAYFEA QDTTTSSSTV SSYKIYVCGM PTESCTGGTL

81 PVTDSDNGPR RMQCTCGQYK YFISASLGWH VRC

Oxidation (M) (+15.99)

Supporting Peptides:

Peptide	Uniq	-10lgP	Mass	Length	ppm	m/z	z	RT	Fraction	Scan	Source File	Area ebraeus	Area judaeus	#Feature	#Feature ebraeus	#Feature judaeus	Start	End	PTM	AScore	Found By
K.M(+15.99)EVYSNNQGK.Y	Y	57.67	1184.5132	10	1.4	593.2625	2	11.79	1	F1:2928	20210611-08_2021021-OK192A_815_5.3.236.mgf	0		0	0	0	22	31	Oxidation (M)	M1:Oxidation (M):1000.00	PEAKS DB
total 1 peptides																					

CebrOK016_E_135_Rimp

back to list

| Protein Coverage | Supporting Peptides |

Protein Coverage:

1 AGIQYSAESP MQQCSTHAVS KMEVYSNNQG KYELLDSYCS CDSGEAYFEA QDTTTSSSTV SSYKIYVCGM PTESCTGGTL

81 PVTDSDNGPR RMQCTCGQYK YLVSASLGWH VRC

Oxidation (M) (+15.99)

Supporting Peptides:

Peptide	Uniq	-10lgP	Mass	Length	ppm	m/z	z	RT	Fraction	Scan	Source File	Area ebraeus	Area judaeus	#Feature	#Feature ebraeus	#Feature judaeus	Start	End	PTM	AScore	Found By
K.M(+15.99)EVYSNNQGK.Y	Y	57.67	1184.5132	10	1.4	593.2625	2	11.79	1	F1:2928	20210611-08_2021021-OK192A_815_5.3.236.mgf	0		0	0	0	22	31	Oxidation (M)	M1:Oxidation (M):1000.00	PEAKS DB
total 1 peptides																					

OK193_E_223_Rimp01

back to list

| Protein Coverage | Supporting Peptides |

Protein Coverage:

1 AGIQYSAESP MQQCSTHAVS KMEVYSNNQG KYELLDSYCS CDSGEAYFDA QDTTTSSSTV SSYKIYVCGM PTESCTGGTL

81 PVTDSDNGPR RMQCTCGQYK YLVSASLGWH VRC

Oxidation (M) (+15.99)

Supporting Peptides:

Peptide	Uniq	-10lgP	Mass	Length	ppm	m/z	z	RT	Fraction	Scan	Source File	Area ebraeus	Area judaeus	#Feature	#Feature ebraeus	#Feature judaeus	Start	End	PTM	AScore	Found By
K.M(+15.99)EVYSNNQGK.Y	Y	57.67	1184.5132	10	1.4	593.2625	2	11.79	1	F1:2928	20210611-08_2021021-OK192A_815_5.3.236.mgf	0		0	0	0	22	31	Oxidation (M)	M1:Oxidation (M):1000.00	PEAKS DB
total 1 peptides																					

SRX1323887_E_204_Rimp01

back to list

| Protein Coverage | Supporting Peptides |

Protein Coverage:

1 AGIQYSAESP MQQCSTHAVS KMEVYSNNQG KYELLDSYCS CDSGEAYFEA QDTTTSSSTV SSYKIYVCGM PTESCTGGTL

81 PVTDSDNGPR RMQCTCGQYK YLVSASLGWH VRC

Oxidation (M) (+15.99)

Supporting Peptides:

Peptide	Uniq	-10lgP	Mass	Length	ppm	m/z	z	RT	Fraction	Scan	Source File	Area ebraeus	Area judaeus	#Feature	#Feature ebraeus	#Feature judaeus	Start	End	PTM	AScore	Found By
K.M(+15.99)EVYSNNQGK.Y	Y	57.67	1184.5132	10	1.4	593.2625	2	11.79	1	F1:2928	20210611-08_2021021-OK192A_815_5.3.236.mgf	0		0	0	0	22	31	Oxidation (M)	M1:Oxidation (M):1000.00	PEAKS DB
total 1 peptides																					

CebrOK014_E_124_Rimp01

back to list

| Protein Coverage | Supporting Peptides |

Protein Coverage:

1 AGIQYSAESP MQQCSTHAVS KMEVYSNNQG KYELLDSYCS CDSGEAYFEA QDTTTSSSTV SSYKIYVCGM PTESCTGGTL

81 PVTDSDNGPR RMQCTCGQYK YLVSASLGWH VRC

Oxidation (M) (+15.99)

Supporting Peptides:

Peptide	Uniq	-10lgP	Mass	Length	ppm	m/z	z	RT	Fraction	Scan	Source File	Area ebraeus	Area judaeus	#Feature	#Feature ebraeus	#Feature judaeus	Start	End	PTM	AScore	Found By
K.M(+15.99)EVYSNNQGK.Y	Y	57.67	1184.5132	10	1.4	593.2625	2	11.79	1	F1:2928	20210611-08_2021021-OK192A_815_5.3.236.mgf	0		0	0	0	22	31	Oxidation (M)	M1:Oxidation (M):1000.00	PEAKS DB
total 1 peptides																					

OK213_E_267_Rimp01

back to list

| Protein Coverage | Supporting Peptides |

Protein Coverage:

1 AGIQYPTVRP IGPCSTNAIS K**TELYSNR**YG RYVLHYSYCS CASGEVYFEA QDTTTSYTTV LYKIYACGAP THSCSGETLP

81 V

Supporting Peptides:

Peptide	Uniq	-10lgP	Mass	Length	ppm	m/z	z	RT	Fraction	Scan	Source File	Area ebraeus	Area judaeus	#Feature	#Feature ebraeus	#Feature judaeus	Start	End	PTM	AScore	Found By
K.TELYSNR.Y	Y	57.66	881.4243	7	5.6	441.7200	2	15.77	2	F2:59839	20210611-09_2021021-OK160A_816_5.3.236.mgf		0	0	0	0	22	28			PEAKS DB
total 1 peptides																					

OK212_J_266_Rimp01

back to list

| Protein Coverage | Supporting Peptides |

Protein Coverage:

1 IQYPTVRPIG PCSTNAISK**T ELYSNR**YGRY VLHYSYCSCA SGEVYFEAQD TTTSYTTVLY KIYACGAPTH SCSGETLPVT

81 DPDDRPRKMQ CTCEQYKYLL YPRLGWLVRC

Supporting Peptides:

Peptide	Uniq	-10lgP	Mass	Length	ppm	m/z	z	RT	Fraction	Scan	Source File	Area ebraeus	Area judaeus	#Feature	#Feature ebraeus	#Feature judaeus	Start	End	PTM	AScore	Found By
K.TELYSNR.Y	Y	57.66	881.4243	7	5.6	441.7200	2	15.77	2	F2:59839	20210611-09_2021021-OK160A_816_5.3.236.mgf		0	0	0	0	20	26			PEAKS DB
total 1 peptides																					

CebrOK035_J_314_Rimp01

back to list

| Protein Coverage | Supporting Peptides |

Protein Coverage:

1 IQYPTVRPIG PCSTNAISK**T ELYSNR**YGRY VLHYSYCSCA SGEVYFEAQD TTTSYTTVLY KIYACGAPTH SCSGETLPVT

81 DPDDRPRKMQ CTCEQYKYLL YPRLGWLVRC

Supporting Peptides:

Peptide	Uniq	-10lgP	Mass	Length	ppm	m/z	z	RT	Fraction	Scan	Source File	Area ebraeus	Area judaeus	#Feature	#Feature ebraeus	#Feature judaeus	Start	End	PTM	AScore	Found By
K.TELYSNR.Y	Y	57.66	881.4243	7	5.6	441.7200	2	15.77	2	F2:59839	20210611-09_2021021-OK160A_816_5.3.236.mgf		0	0	0	0	20	26			PEAKS DB
total 1 peptides																					

OK005_J_279_Rimp01

back to list

| Protein Coverage | Supporting Peptides |

Protein Coverage:

1 IQYPTVRPIG PCSTNAISK**T ELYSNR**YGRY VLHYSYCSCA SGEVYFEAQD TTTSYTTVLY KIYACGAPTH SCSGETLPVT

81 DPDDRPRKMQ CTCEQYKYLL YPRLGWLVRC

Supporting Peptides:

Peptide	Uniq	-10lgP	Mass	Length	ppm	m/z	z	RT	Fraction	Scan	Source File	Area ebraeus	Area judaeus	#Feature	#Feature ebraeus	#Feature judaeus	Start	End	PTM	AScore	Found By
K.TELYSNR.Y	Y	57.66	881.4243	7	5.6	441.7200	2	15.77	2	F2:59839	20210611-09_2021021-OK160A_816_5.3.236.mgf		0	0	0	0	20	26			PEAKS DB
total 1 peptides																					

OK214_J_274_Rimp01

back to list

| Protein Coverage | Supporting Peptides |

Protein Coverage:

1 IQYPTVRPIG PCSTNAISK**T ELYSNR**YGRY VLHYSYCSCA SGEVYFEAQD TTTSSSTVSS YKIYVCGMPT ESCTGGTLPV

81 TDSDNGPRRM QCTCGQYKYF VYSWGWHVRC

Supporting Peptides:

Peptide	Uniq	-10lgP	Mass	Length	ppm	m/z	z	RT	Fraction	Scan	Source File	Area ebraeus	Area judaeus	#Feature	#Feature ebraeus	#Feature judaeus	Start	End	PTM	AScore	Found By
K.TELYSNR.Y	Y	57.66	881.4243	7	5.6	441.7200	2	15.77	2	F2:59839	20210611-09_2021021-OK160A_816_5.3.236.mgf		0	0	0	0	20	26			PEAKS DB
total 1 peptides																					

OK214_J_273_Rimp01

back to list

| Protein Coverage | Supporting Peptides |

Protein Coverage:

1 IQYPTVRPIG PCSTNAISK**T ELYSNR**YGRY VLHYSYCSCA SGEVYFEAQD TTTSYTTVLY KIYACGAPTH SCSGETLPVT

81 DPDDRPRKMQ CTCEQYKYLL YPRLGWLVRC

Supporting Peptides:

Peptide	Uniq	-10lgP	Mass	Length	ppm	m/z	z	RT	Fraction	Scan	Source File	Area ebraeus	Area judaeus	#Feature	#Feature ebraeus	#Feature judaeus	Start	End	PTM	AScore	Found By
K.TELYSNR.Y	Y	57.66	881.4243	7	5.6	441.7200	2	15.77	2	F2:59839	20210611-09_2021021-OK160A_816_5.3.236.mgf		0	0	0	0	20	26			PEAKS DB
total 1 peptides																					

CebrOK033_J_221_Rimp01

back to list

| Protein Coverage | Supporting Peptides |

Protein Coverage:

1 IQYPTVRPIG PCSTNAISK**T ELYSNR**YGRY VLHYSYCSCA SGEVYFEAQD TTTSYTTVLY KIYACGAPTH SCSGETLPVT

81 DPDDRPRKMQ CTCEQYKYLL YPRLGWLVRC

Supporting Peptides:

Peptide	Uniq	-10lgP	Mass	Length	ppm	m/z	z	RT	Fraction	Scan	Source File	Area ebraeus	Area judaeus	#Feature	#Feature ebraeus	#Feature judaeus	Start	End	PTM	AScore	Found By
K.TELYSNR.Y	Y	57.66	881.4243	7	5.6	441.7200	2	15.77	2	F2:59839	20210611-09_2021021-OK160A_816_5.3.236.mgf		0	0	0	0	20	26			PEAKS DB
total 1 peptides																					

Peptide List

Prepared with PEAKS ™ (bioinfor.com)
